# Integrative network pharmacology and molecular modeling approaches reveal the therapeutic potential of *Syzygium samarangense* phytocompounds against diabetes retinopathy

**DOI:** 10.3389/fbinf.2026.1884743

**Published:** 2026-08-18

**Authors:** Arun Kandhan, Bijo Mathew, Sundarrajan Thirugnanasambandam

**Affiliations:** 1 Department of Pharmaceutical Chemistry, SRM College of Pharmacy, Faculty of Medicine and Health Sciences, SRM Institute of Science and Technology, Chengalpattu, Tamil Nadu, India; 2 Department of Pharmaceutical Chemistry, Amrita School of Pharmacy, Amrita Vishwa Vidyapeetham, AIMS Health Sciences Campus, Kochi, India

**Keywords:** diabetic retinopathy, MMGBSA, molecular docking, molecular dynamics, molecular modelling, network pharmacology, Syzygium samarangense

## Abstract

**Introduction:**

Diabetes retinopathy (DR) is a progressive microvascular complication of diabetes mellitus characterized by oxidative stress, inflammation, and neurovascular dysfunction. Current therapies provide limited efficacy, highlighting the need for multi-targeted, natural therapeutic alternatives. *Syzygium samarangense* (SS) is a phytochemical-rich medicinal plant with potential anti-Diabetes properties.

**Methodology:**

A data-driven systems pharmacology approach was employed to explore the anti-DR potential of *S. samarangense* phytochemicals. Twenty-nine active compounds were screened for drug-likeness using Lipinski’s Rule of Five. Overlapping targets between SS and DR were identified, yielding 403 shared targets. Network pharmacology analysis highlighted ten core proteins, including SRC, ALB, GAPDH, and TNF. Gene Ontology (GO) and KEGG pathway enrichment analyses identified key pathways such as AGE-RAGE and HIF-1 signaling. Molecular docking was performed to evaluate ligand–target interactions, followed by 500 ns molecular dynamics (MD) simulations and MM-GBSA binding free energy analysis for validation.

**Results and Discussion:**

Docking studies identified Pinocembrin (SS5) and Stercurensin (SS4) as top-ranked ligands, with strong binding affinities toward SRC and ALB, respectively. MD simulations demonstrated superior structural stability for the SS5–SRC complex, whereas SS4–ALB exhibited moderate stability. MM-GBSA calculations further confirmed favorable binding energies, supporting their drug-like behavior. These multi-target interactions suggest that *S. samarangense* phytochemicals can modulate key DR-associated pathways.

**Conclusion:**

*S. samarangense* exhibits promising multi-target activity against Diabetes retinopathy. Pinocembrin (SS5) and Stercurensin (SS4) emerge as potential lead compounds for further experimental validation and drug development.

## 1 Introduction

Diabetes retinopathy (DR) is one of the most common microvascular complications with high visual impairment and blindness rates among individuals caused by Diabetes mellitus (DM). Recent global epidemiological studies on Diabetes retinopathy (DR) reveal significant insights into its prevalence and burden. As of 2023, it is estimated that 103.12 million people worldwide have Diabetes retinopathy, with projections suggesting an increase to 160.5 million by 2045 due to rising diabetes rates. Among Diabetes patients, nearly one-third develop DR, underscoring its significant public health burden. The condition is divided into two categories: proliferative Diabetes retinopathy (PDR) and non-proliferative Diabetes retinopathy (NPDR), with the latter representing an advanced stage characterized by neovascularization, hemorrhages, and increased risk of retinal detachment (Korkmaz et al., 2025; Rees et al., 2016; Teo et al., 2021).

**FIGURE 1:**
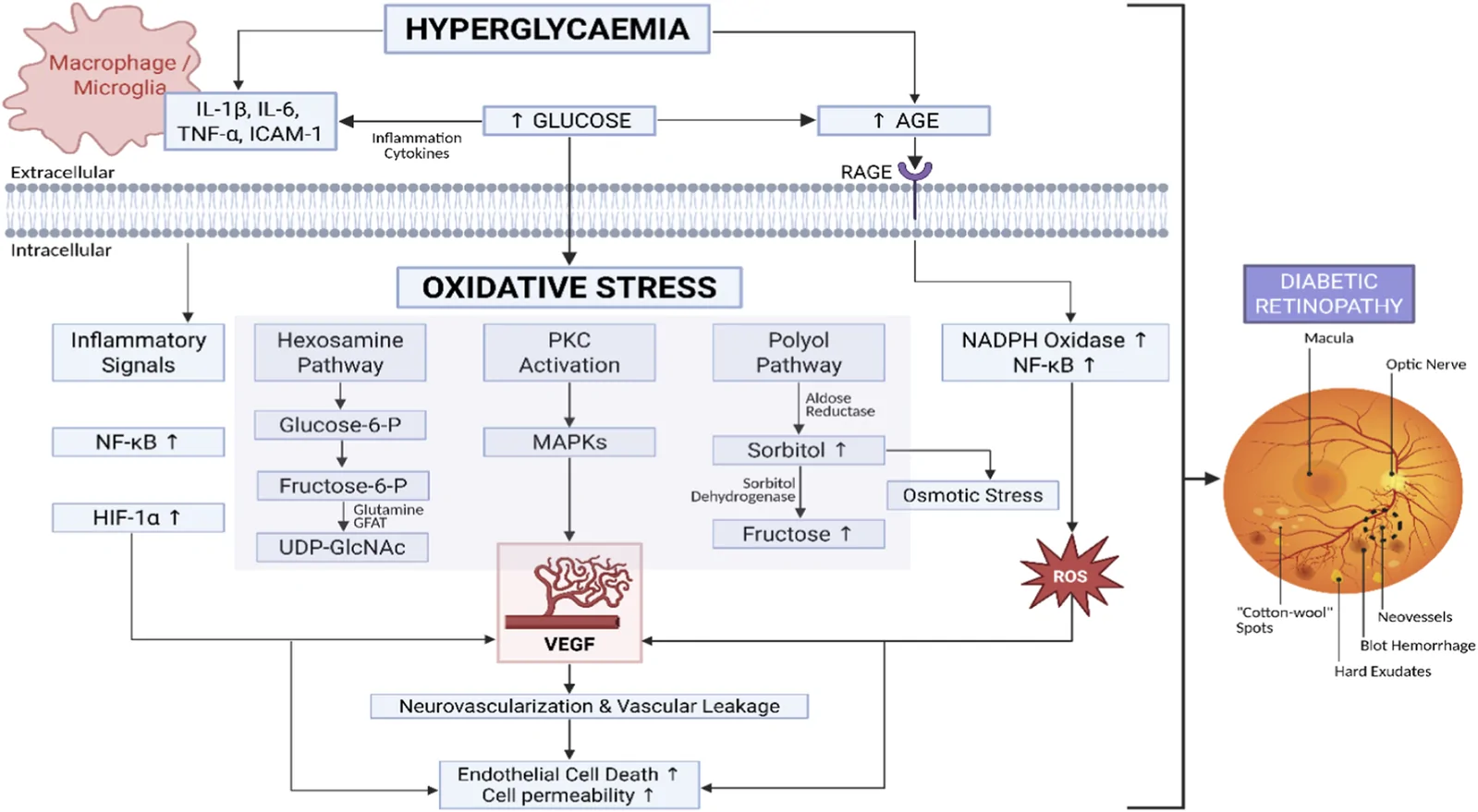
Mechanism involved in the Diabetes retinopathy.

The pathogenesis of Diabetes retinopathy (DR) is multifactorial, encompassing oxidative stress, inflammation, and neurovascular dysfunction induced by chronic hyperglycemia (Al-Namaeh, 2022). Hyperglycemia activates several biochemical pathways, such as the polyol pathway, the formation of advanced glycation end-products (AGEs), the activation of protein kinase C (PKC), and the hexosamine pathway and can leads to the damage of endothelial cells in the retina. Overproduction of reactive oxygen species (ROS) causes mitochondria to stop working, cells to die, and blood vessels to become more permeable. Moreover, inflammatory mediators, including vascular endothelial growth factor (VEGF), interleukins (IL-6, IL-1β), and tumor necrosis factor-alpha (TNF-α), are instrumental in aggravating retinal damage. Despite the availability of current therapeutic strategies such as anti-VEGF injections (e.g., bevacizumab, ranibizumab), corticosteroids, and laser photocoagulation, these treatments have inherent limitations, including high costs, potential side effects, and limited efficacy in preventing disease progression. Therefore, there is an urgent need to explore alternative therapeutic approaches, particularly those derived from natural sources that target multiple pathological mechanisms of DR with minimal side effects (D’Angelo et al., 2025; Chen et al., 2024; Ye et al., 2024). The diagrammatic representation of the mechanism is given in Figure 1.

In India, numerous herbs and natural substances are utilized as adjunctive therapies for Diabetes mellitus (DM). The precise effects and processes of these traditional therapies on Diabetes retinopathy (DR) remain contentious and little comprehended. Comprehensive scientific methodologies are crucial for thoroughly investigating and elucidating these mechanisms. *Syzygium samarangense* (Huang et al., 2024), widely referred to as wax apple, is a member of the Myrtaceae family. Traditionally, nearly all parts of this plant, including roots, leaves, blossoms, and fruits, are utilized to address ailments such as diabetes, bronchitis, fever, asthma, gastrointestinal spasms, infections and renal problems in India and other countries. This plant is rich in polyphenols, which are linked to numerous therapeutic benefits. Leaf extracts of *S. samarangense* are documented to possess antiDiabetes, antihyperglycemic, antiulcer, anti-inflammatory, antifungal, analgesic, spasmolytic, cytotoxic, antidepressant, antioxidant, and hepatoprotective activities. Moreover, these extracts are employed to address ailments like diarrhoea, fever, and eczema (Rashied et al., 2022; Sururi et al., 2024).

**FIGURE 2:**
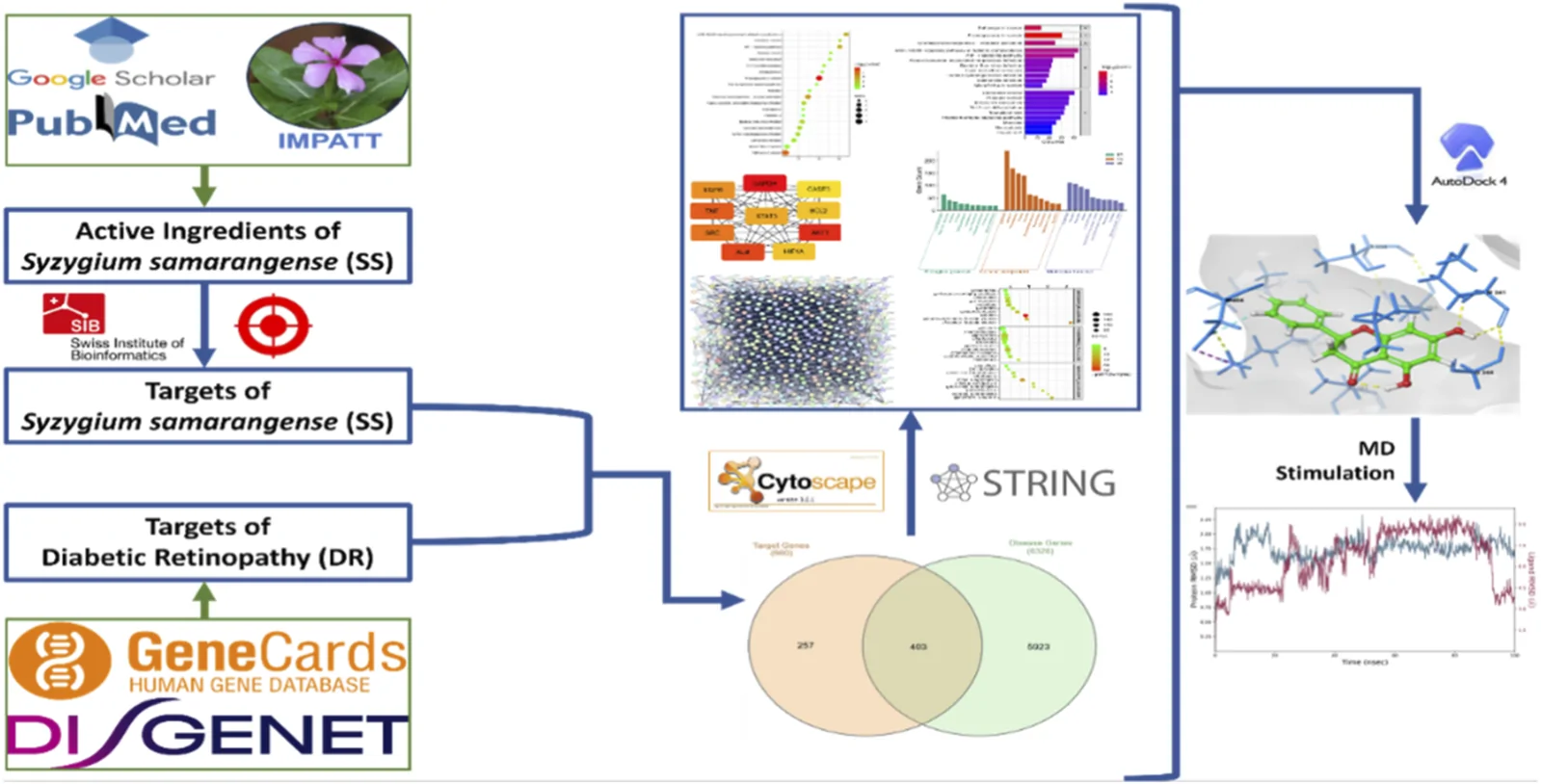
The workflow overview of the current study.

Several research studies have examined the antiDiabetes and antioxidant effects of *Syzygium samarangense* by use of *in-vivo* and *in-vitro* models. Leaf and fruit extracts can lower blood glucose, improve the sensitivity of insulin, and protect the tissues of Diabetes rats against the damage during the oxidative stress. It also downregulated pro-inflammatory cytokines such as TNF-a and IL-6 which are strongly linked to Diabetes microvascular complications and inhibited the production of end-products of glycation (AGE). The direct action of *S. samarangense* on Diabetes retinopathy-specific pathways, such as VEGF, HIF-1a, or NF-kB activation, is, however, evidently under investigated. The lack of a defined molecular mechanism often hinders the translation of traditional botanical knowledge into evidence-based therapeutics. Consequently, the motivation of this study is twofold: first, to systematically decode the anti-DR potential of *S. samarangense* through a high-resolution, multi-database systems pharmacology approach; and second, to identify stable, high-affinity lead molecules that warrant targeted experimental exploration, thus optimizing the trajectory of drug discovery. In recent years, *in silico* approaches have emerged as powerful tools in drug discovery and pharmacology, enabling the systematic exploration of complex biological interactions. The purpose of this study was to evaluate the probable processes and binding interactions of Diabetes retinopathy. To do so, we utilized a combination of network pharmacology, molecular docking, and molecular dynamics simulations. Network pharmacology was utilized to construct and analyze compound-target-disease networks, providing insights into the multi-target effects and therapeutic potential. The binding affinities and interaction patterns between important compounds and their target proteins were subsequently predicted through molecular docking. Lastly, molecular dynamics simulations were implemented to evaluate the stability and conformational dynamics of the protein-ligand complexes over time, thereby providing a more comprehensive comprehension of their atomic-level binding behavior. These computational methods collectively establish a durable framework for the elucidation of the molecular underpinnings of Diabetes retinopathy. The prospective benefits of various components of the *S. samarangense* plant in the treatment of metabolic disorders, particularly Diabetes Mellitus, were investigated. The objective of this insilico investigation was to investigate the potential phytochemicals of the plant *S. samarangense* and their impact on Diabetes retinopathy (Zhang et al., 2021). The entire study workflow overview can be seen in Figure 2.

## 2 Results and discussion

### 2.1 Active phytocompounds and common target screening

**FIGURE 3:**
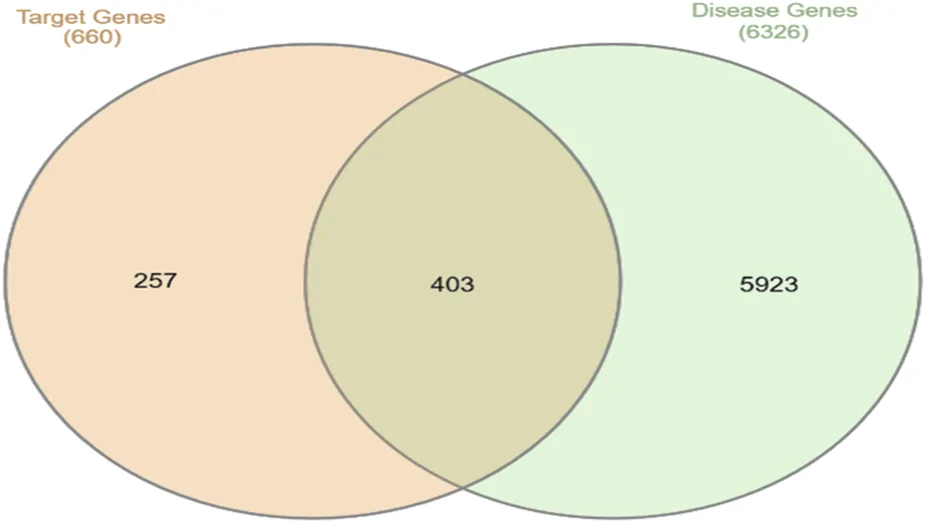
Venn diagram of SS-DR common targets.

We obtained 29 ingredients (phytocompounds) that satisfying the criteria of oral bioavailability ≥30%, Lipinski’s rule of five (ro5), and drug likeness ≥0.18. After removing repetitive gene symbols, we acquired 257 target genes from a total of 660 SS-related targets. A total of 6,326 disease-related targets (disease genes) were obtained from the GeneCards and DisGeNET databases. Following the elimination of duplicated gene symbols, we identified 5923 DR-related targets from the aforementioned databases. Following the intersection of DR-related targets and SS-related targets (Target Genes), we identified 403 common targets associated with both SS and DR are shown in Figure 3.

### 2.2 Diabetes retinopathy-related targets screening

**FIGURE 4:**
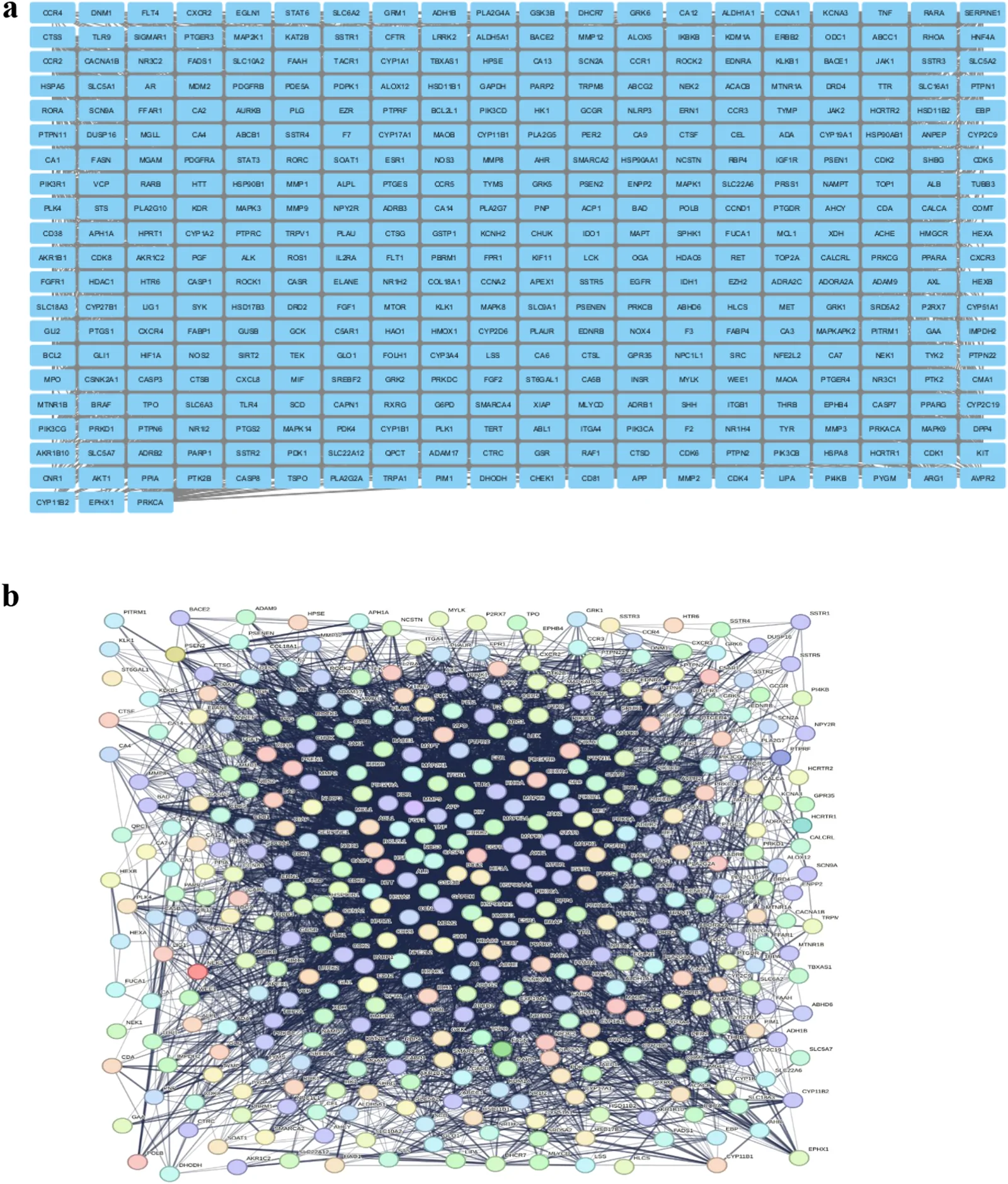
**(a)** PPI network of common targets. **(b)** Summary STRING view of current interactions.

**FIGURE 5:**
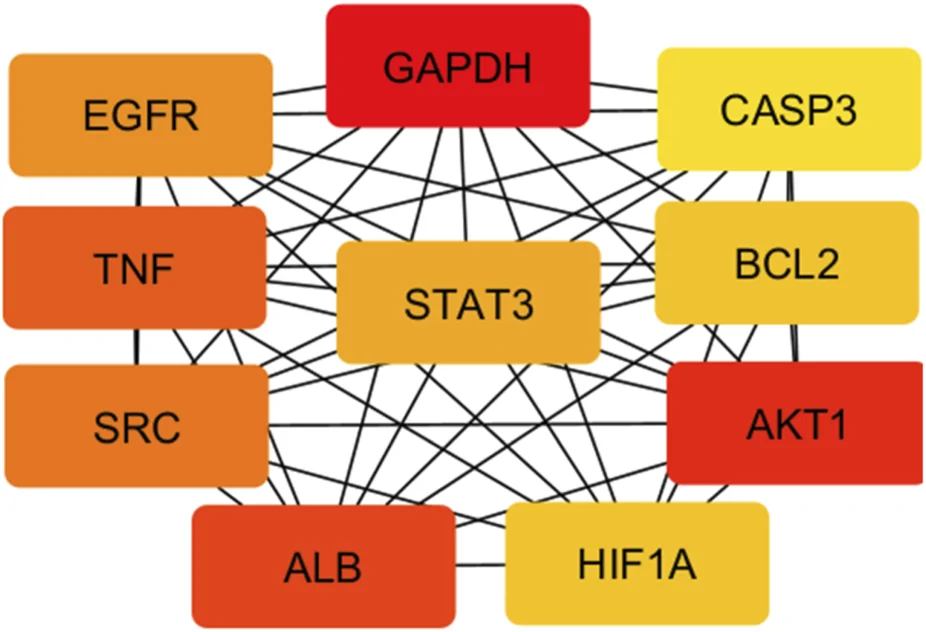
Summary STRING view of current interactions.

The results for each compound were then combined and imported into Cytoscape 3.7.2 software, which used CytoHubba to identify the top ten ranked proteins based on their degree value, resulting in an active ingredient-Diabetes Retinopathy target network shown in Figure 3. The STRING (Search Tool for the Retrieval of Interacting Genes/Proteins) platform was imported into Cytoscape software and used to generate a PPI network based on common targets. The PPI network revealed highly interconnected networks and significant correlations between the targets (Figures 4a,b). The genes GAPDH, AKT1, ALB, TNF, SRC, EGFR, STAT3, BCL2, HIF1A, and CASP3 are identified as potential targets for Diabetes retinopathy based on an analysis of the data and the results are shown in Table 1 and Figure 5.

**TABLE 1:** Top 10 proteins in the network ranked by the degree method.

Rank	Gene name	Description	Uniprot ID	PDB ID	Score
1	GAPDH	Glyceraldehyde-3-phosphate dehydrogenase	P04406	6YND	227
2	AKT1	AKT serine/Threonine kinase 1	P31749	1UNQ	218
3	ALB	Albumin	P02768	6WUW	211
4	TNF	Tumor necrosis factor	P01375	8HXQ	208
5	SRC	Tyrosine-protein kinase SRC	P12931	2H8H	180
6	EGFR	Epidermal growth factor receptor erbB1	P00533	5UGB	179
7	STAT3	Signal transducer and activator of transcription 3	P40763	4ZIA	169
8	BCL2	Apoptosis regulator Bcl-2	P10415	6GL8	165
9	HIF1A	Hypoxia-inducible factor 1 alpha	Q16665	4H6J	165
10	CASP3	Caspase-3	P42574	3KJF	158

### 2.3 GO and KEGG enrichment analysis

**FIGURE 6:**
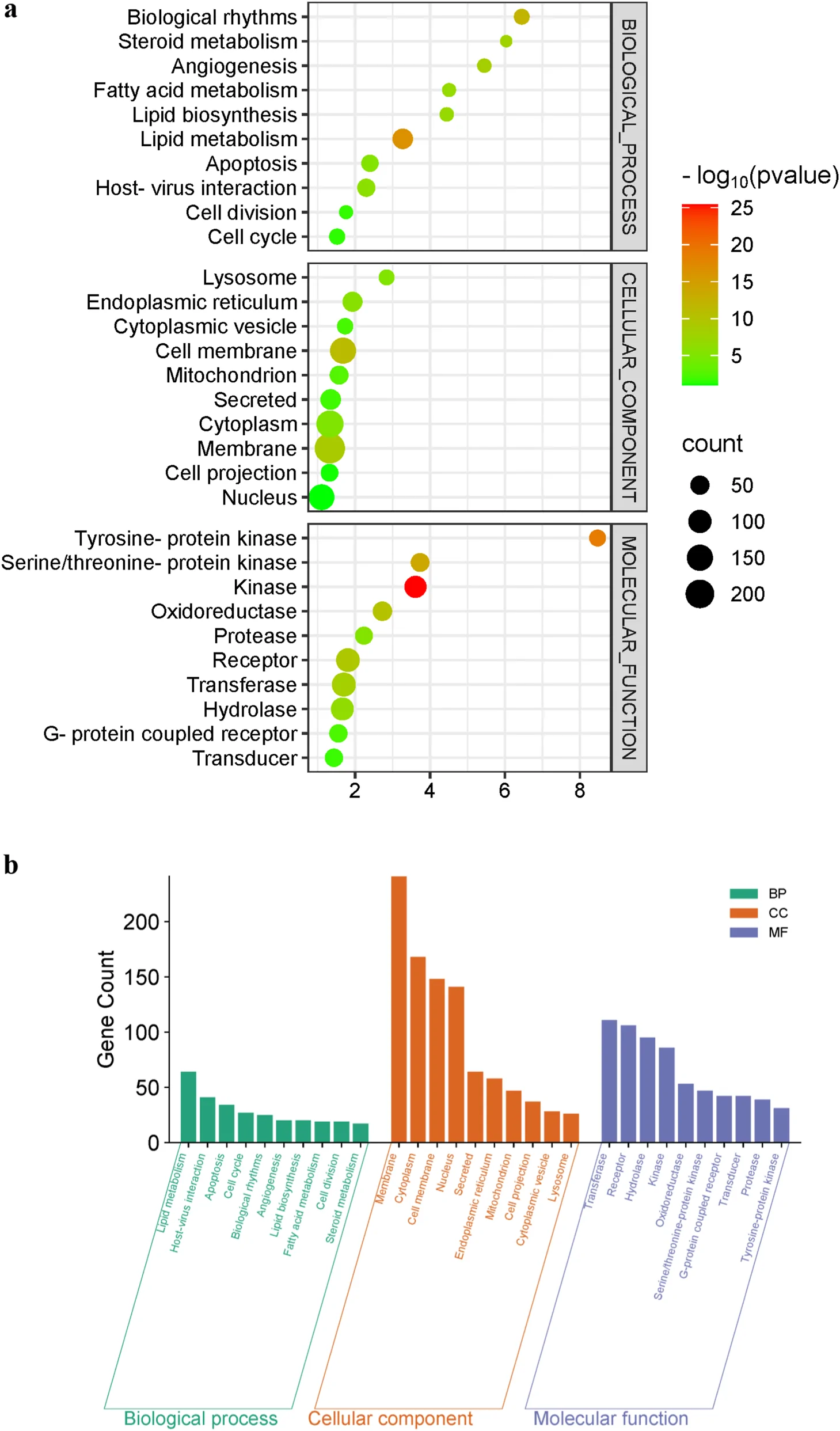
**(a)** The results of the Gene Ontology (GO) enrichment study are shown in a bubble chart for three groups: Molecular Function (MF), Cellular Component (CC), and Biological Process (BP). The titles of more prevalent GO terms are on the Y-axis, while the gene ratio for each term is on the X-axis. The color gradient represents the adjusted p-value, which means that the results are statistically significant. The size of each bubble shows how many genes are implicated. The next bubble charts are formatted using the same guidelines for visualizing. **(b)** Key process from the results for Go categories and the counts of related target genes.

**FIGURE 7:**
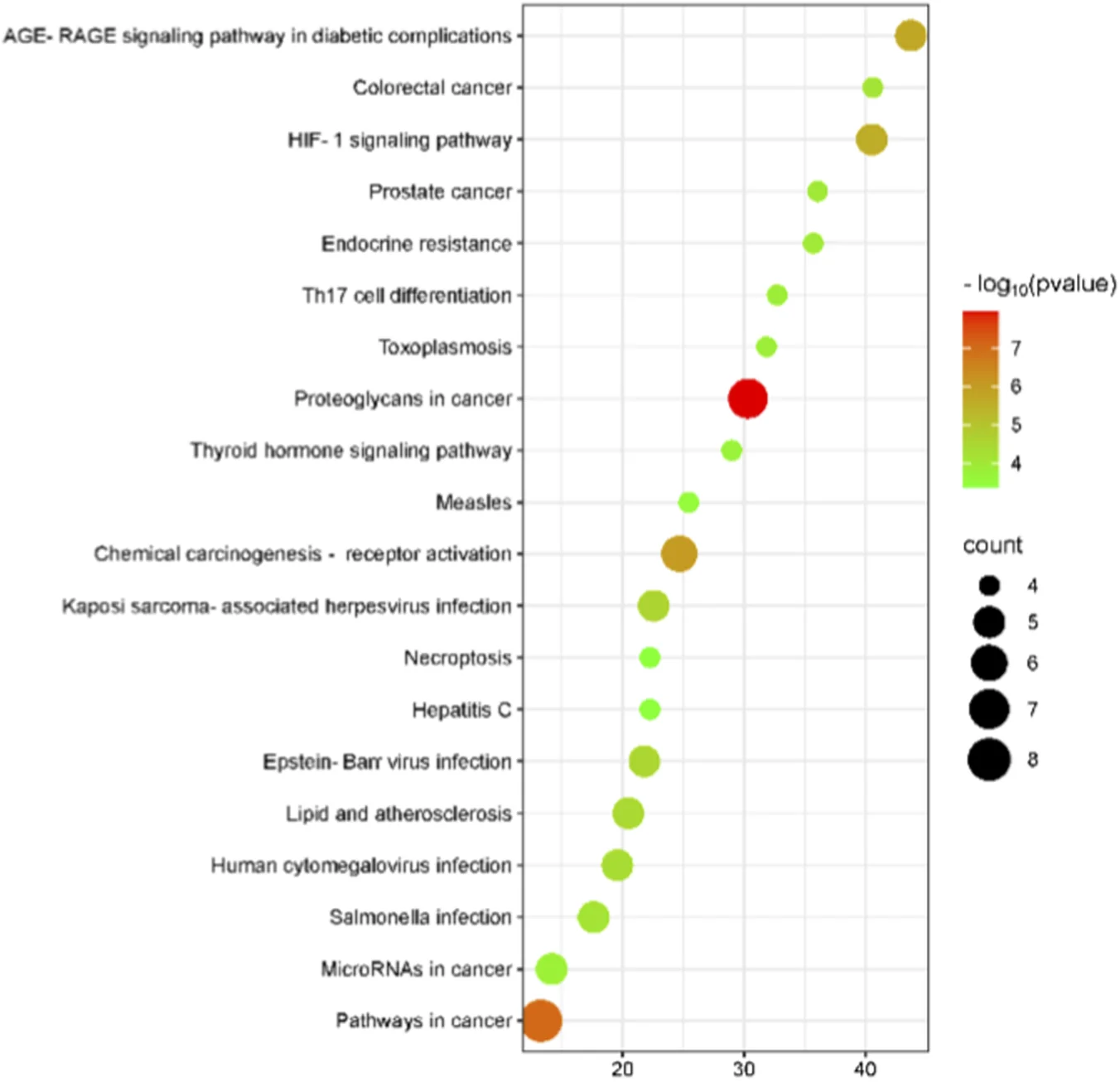
The Top 20 results of KEGG pathway.

**FIGURE 8:**
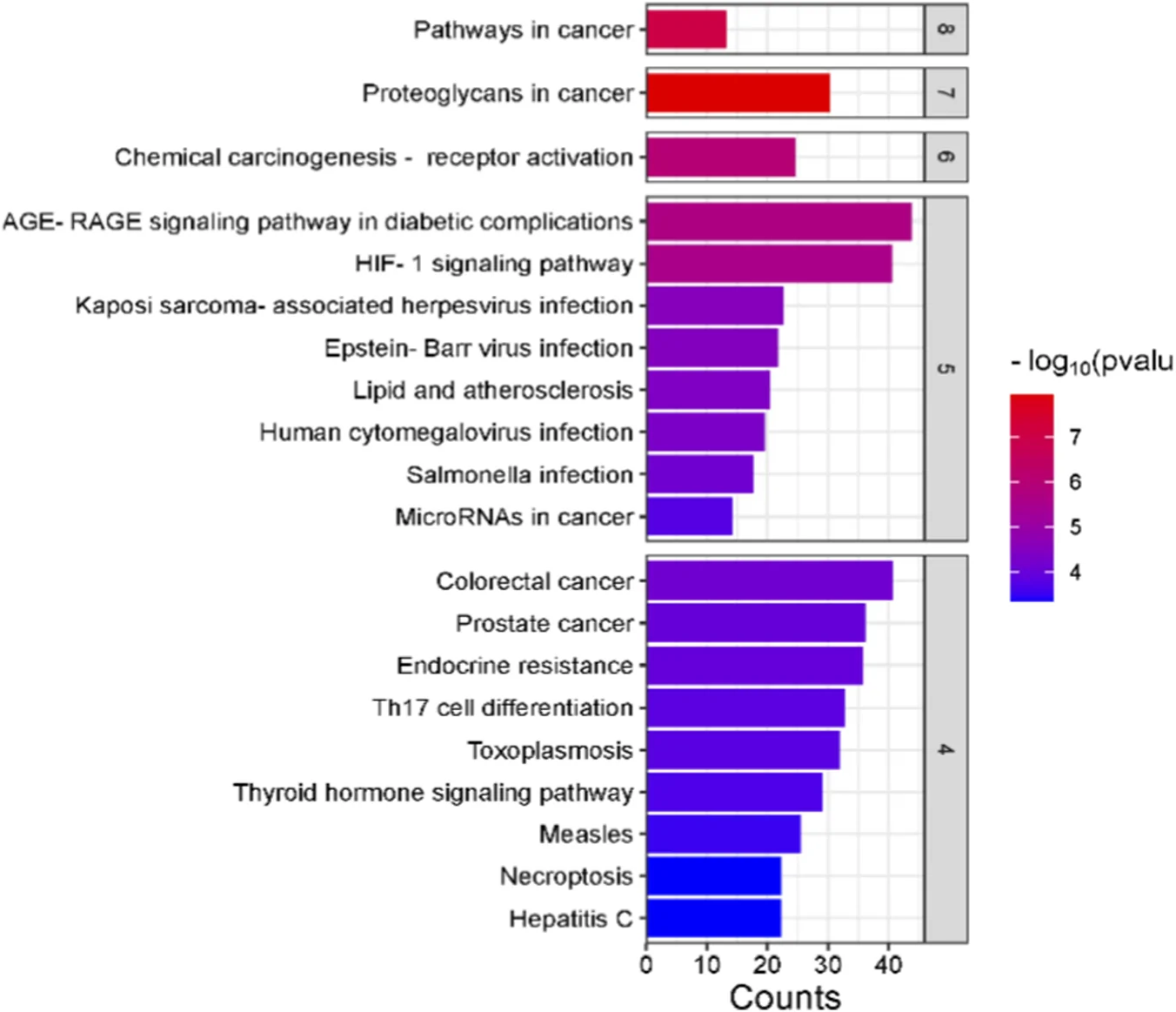
The Top 20 results of KEGG pathway.

Go enrichment (Gene Ontology) analysis of common SS-DR targets found for 29 SS targets on DR. We figured out what these 29 targets do in terms of their biological processes, cellular components, and molecular functions. We did Gene Ontology analysis (GO) and KEGG pathway analysis on 29 targets. The Gene Ontology (GO) results showed that 33 biological processes (BP), 14 cellular components (CC), and 20 molecular functions (MF) were examined. In a bubble chart format, Figure 6a shows the top 10 terms in each of the three categories mentioned above. We also looked at 64 pathways from the KEGG database to see what they showed. A bar chart (Figure 6b) shows the main processes that came out of the results for GO categories and the number of target genes that were related to them. The KEGG analysis found the top 20 pathways and made a scatterplot and bar chart to go with them are shown in Figures 7, 8 respectively. The AGE-RAGE signaling pathway in Diabetes complications, the HIF-1 signaling pathway, and the pathways in cancer and proteoglycans in cancer may all be important for SS’s treatment of Diabetes retinopathy. The size of the dot is related to the count, the color’s brightness shows how red it is, and the p-value is lower. Table 2 shows the top five signaling pathways, along with their target genes and the ratio of genes in each pathway.

**TABLE 2:** Top five signaling pathways by gene ratio and their related target genes.

Pathways	Target genes	Count
hsa05200: Pathways in cancer	HSP90AA1, CCND1, CASP3, STAT3, BCL2, HIF1A, ESR1, MTOR	8
hsa05205: Proteoglycans in cancer	CCND1, CASP3, STAT3, HIF1A, ESR1, TNF, MTOR	7
hsa05207: Chemical carcinogenesis - receptor activation	HSP90AA1, CCND1, STAT3, BCL2, ESR1, MTOR	6
hsa04933: AGE-RAGE signaling pathway in diabetes complications	CCND1, CASP3, STAT3, BCL2, TNF	5
hsa04066: HIF-1 signaling pathway	STAT3, BCL2, HIF1A, GAPDH, MTOR	5

### 2.4 Molecular docking of phytoconstituents and their targets

Molecular docking was used to evaluate the active phytoconstituents and the targets discovered in them. From the top 10 targets in the network study, 7 targets/pathways from the network study consisted for molecular docking analysis which are: GAPDH (6YND), ALB (6WUW), TNF (2AZ5), SRC (2H8H), EGFR (5UGB), BCL2 (6GL8), and STAT3 (6NJS). Table 3 displays the free binding energies (in kcal/mol) of the major targets and active phytoconstituents obtained from molecular docking. The docking results were visualized using PyMOL 3.0 software and Discovery Studio BIOVIA 2024 software.

**TABLE 3:** Molecular docking results of the constituents with the top 7 targeted pathways.

S. No	Code for molecule	Molecules	6YND GAPDF	6WUW ALB	2AZ5 TNF	2H8H SRC	5UGB EGFR	6NJS STAT3	6GL8 BCL2
1	SS1	2′,4′-dihydroxy-6′-methoxy-3′,5′-dimethylchalcone	−2.979	−6.952	−6.884	−6.625	−6.877	−4.117	−5.315
2	SS2	2′-Hydroxy-4′,6′-dimethoxy-3′- methyl dihydrochalcone	-	-	−6.317	−7.9	−5.746	-	-
3	SS3	2′,4′-dihydroxy-6′-methoxy-3′- methyl chalcone	-	−8.538	−6.681	−7.841	−6.683	−3.855	−5.444
4	SS4	Stercurensin	−2.623	−8.983	−6.654	−7.836	−6.684	−4.159	−5.482
5	SS5	Pinocembrin	−3.74	−6.928	−6.757	−9.114	−6.615	−4.993	−4.993
6	SS6	(−)-Strobopinin		−8.31	−6.634	−6.795	−6.036	−4.373	−5.427
7	SS7	8-Methylpinocembrin	−3.324	−6.82	−6.721	−7.471	−7.305	−4.14	−5.53
8	SS8	Demethoxymatteutcinol	−2.207	-	−7.323	−8.688	−8.268	−4.751	−5.405
9	SS9	7-Hydroxy-5-methoxy-6,8- dimethyl-flavanone	−3.74	−8.312	−7.232	−6.933	−6.222	−3.613	-
10	SS10	3,5-di-O-Methyl gossypetin	−3.807	−7.771	−6.807	−8.548	−6.953	−5.36	−6.187
11	SS11	5,7-Dihydroxy-6,8- dimethyl flavanone	−2.631	−8.702	−6.473	−8.757	−7.52	−3.93	−5.468
12	SS12	Aurentiacin	−2.917	−7.907	−6.767	−8.058	−6.506	−3.643	-
13	SS13	Cryptostrobin	−3.036	−8.414	−6.835	−6.914	−6.67	−4.491	−5.845
14	SS14	2-Pentadecyl-5,7- didydroxychromone	−1.394	−6.09	−5.932	−5.789	−4.927	−4.194	-
15	SS15	Samarone D	−1.085	−6.491	−6.066	−6.456	−5.594	−2.744	-
16	SS16	Samarone C	−1.717	−5.939	−6.082	−5.313	−5.08	−4.462	-
17	SS17	Samarone B	−2.443	−4.616	−6.478	−6.513	−5.513	−2.985	−5.724
18	SS18	2R-prunasin	−3.278	−6.879	−5.868	−6.301	−6.485	−3.59	5.429
19	SS19	(3S,5R,6R,7E,9S)- 3,5,6,9-tetrahydroxymegastigman-7-ene	−3.934	-	--5.414	−5.295	−4.1	−3.479	−5.622
20	SS20	Cuminyl aldehyde	−3.648	−7.091	−6.297	−7.01	−6.193	−4.416	−5.912
21	SS21	Spathulenol	−3.166	-	−5.895	−4.318	−4.939	−6.203	−6.203
22	SS22	Anethole	−3.302	−5.441	−5.275	−5.654	−5.482	−3.566	-
23	SS23	Caryophyllene oxide	−2.539	−3.399	−6.345	−4.727	−4.415	-	−5.988
24	SS24	γ-terpinene	-	−5.681	-	−5.36	−4.915	−3.309	−5.362
25	SS25	Limonene	−2.687	−5.237	−5.081	−4.997	−4.956	−4.967	−4.967
26	SS26	Selin-11-en-4-α-ol	-	−5.706	−5.81	−4.482	−3.813	−2.968	−5.372
27	SS27	Epi-ubenol	−2.993	−4.335	−6.226	−5.328	−5.885	−3.51	−5.647
28	SS28	Terpinolene	−3.757	−5.681	−5.574	−5.36	−4.915	−5.362	−5.362
29	SS29	α-terpineol	-	−5.619	−5.171	−5.588	−5.567	−3.569	−5.037

Hereby, the phytoconstituents analyzed targets pathway network and molecular docking score data were combined to select the best ligand for the targets. The findings identified SRC and ALB as the main therapeutic potential targets. Pinocembrin (SS5), Stercurensin (SS4), 5,7-Dihydroxy-6,8- dimethyl flavanone (SS11), Demethoxymatteutcinol (SS8) and 3,5-di-O-Methyl Gossypetin (SS10) were identified as potential active phytoconstituents of SS for treating DR. SS5 exhibited better binding affinity to SRC, with a docking score of −9.114 kcal/mol, while SS4 demonstrated binding affinity to ALB, with a docking score of −8.983 kcal/mol. Furthermore, SS11 exhibited strong interaction with SRC and ALB, obtaining docking scores of −8.757 kcal/mol and −8.702 kcal/mol, respectively, whereas SS8 and SS10 demonstrated docking scores of −8.688 kcal/mol and −8.548 kcal/mol with SRC, so affirming its potential binding affinity.

**FIGURE 9:**
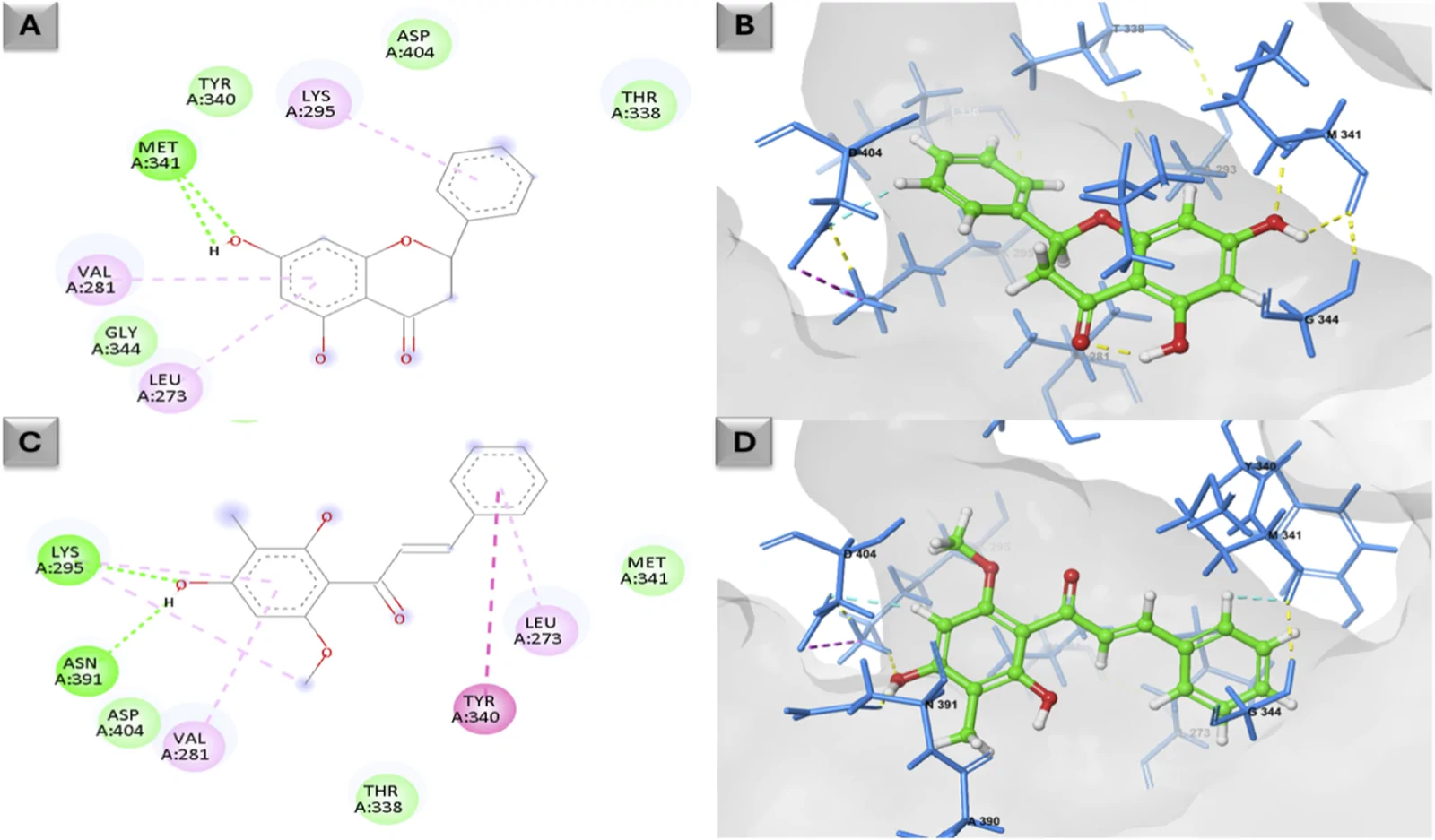
Ligand interaction diagram of the top two compounds, SS4 **(A,B)** and SS5 **(C,D)**, with SRC. Colour-coded legend to specify interaction types: green dashed lines indicate hydrogen bonds, while pink/dashed lines represent hydrophobic and polar contacts, respectively.

**FIGURE 10:**
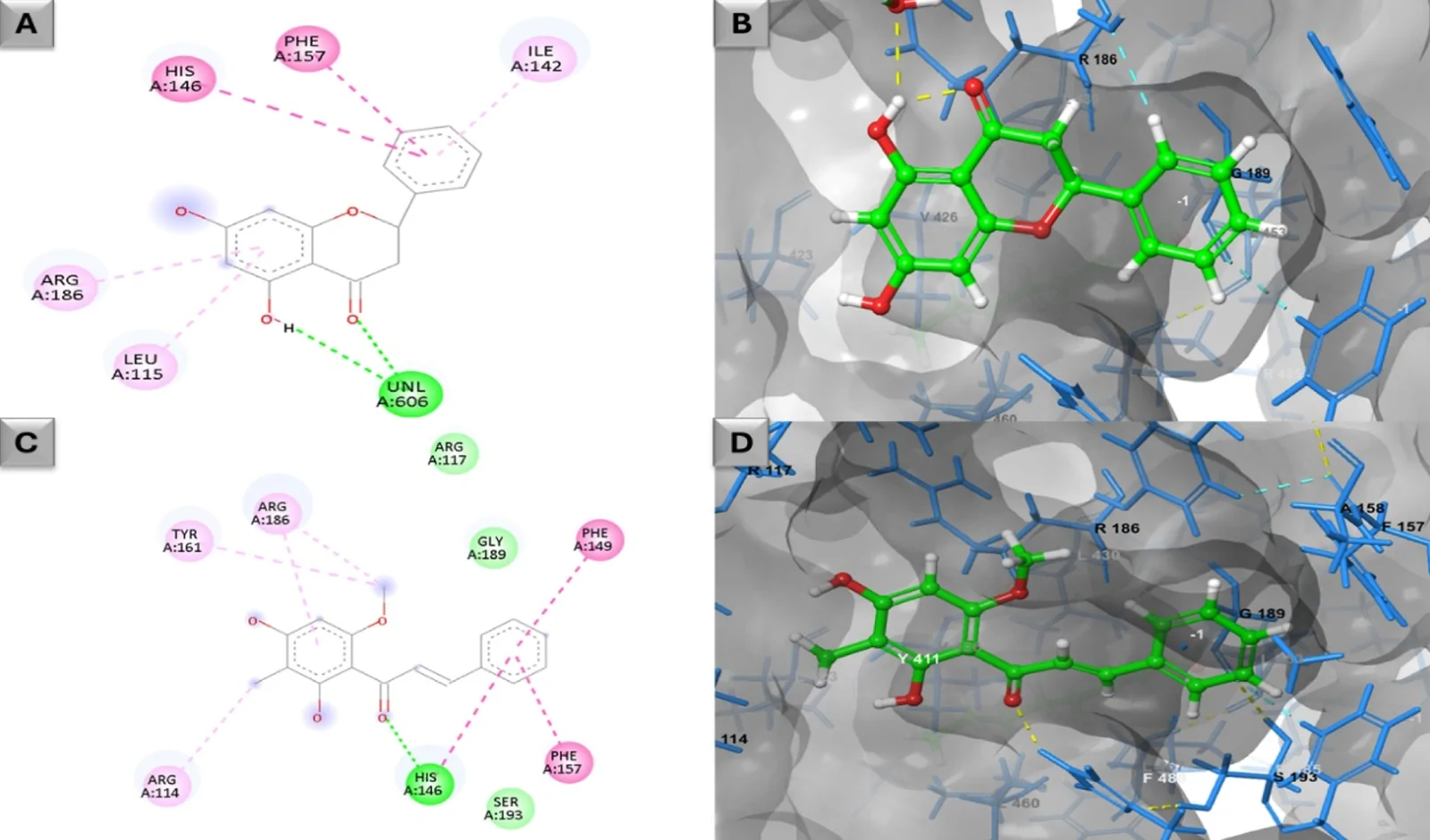
Ligand interaction diagram of the top two compounds, SS4 **(A,B)** and SS5 **(C,D)**, with ALB. Colour-coded legend to specify interaction types: green dashed lines indicate hydrogen bonds, while pink/dashed lines represent hydrophobic and polar contacts, respectively.

After analyzing the data from the KEGG pathway, the SS phytoconstituents–top 10 targets–pathways network, and the results of the molecular docking, it was determined that SRC and ALB are important targets for the therapy of disease. The docking analysis suggested that Pinocembrin (SS5) and Stercurensin (SS4) may potentially interact with SRC and ALB through distinct binding sites and mechanisms. The 2D and 3D ligand interaction diagram of SS5 and SS4 with SRC and ALB are given in Figures 9, 10 and amino acid interaction for compounds and rationale for selecting SRC and ALB for MD are also tabled in Tables 4 and 5, respectively. These targets were chosen for a molecular dynamics (MD) simulation investigation for further analysis.

**TABLE 4:** Amino acid interactions with the target SRC and ALB with SS4 and SS5.

Target	Compound	H-bond residues	Hydrophobic/Polar residues
SRC	SS4	LYS A:295	MET A:341, VAL A:281, LEU A:273
SRC	SS5	LYS A:295, ASN A:391	VAL A:281, TYR A:340, LEU A:273
ALB	SS4	HIS A:146	ILE A:142, PHE A:157, ARG A:186, LEU A:115
ALB	SS5	HIS A:146	PHE A:149, PHE A:157, ARG A:114, ARG A:186, TYR A:161

**TABLE 5:** Rationale for selecting SRC (SS5) and ALB (SS4) for MD evaluation.

DR target	Biological role of DR	Network rank (degree method)	Docking score for ligand	Binding interactions
SRC	VEGF signaling, angiogenesis, BRB breakdown	5th (Score-180)	−9.114 kcal/mol (SS5)	H-bond: LYS A:295, ASN A:391; polar: MET A:341, VAL A:281
ALB	Oxidative stress buffering, retinal osmotic balance	3rd (Score-211)	−8.983 kcal/mol (SS4)	H-bond: HIS A:146, ARG A:186 Polar: ILE A:142 PHE A:157

### 2.5 Molecular dynamics simulation

**FIGURE 11:**
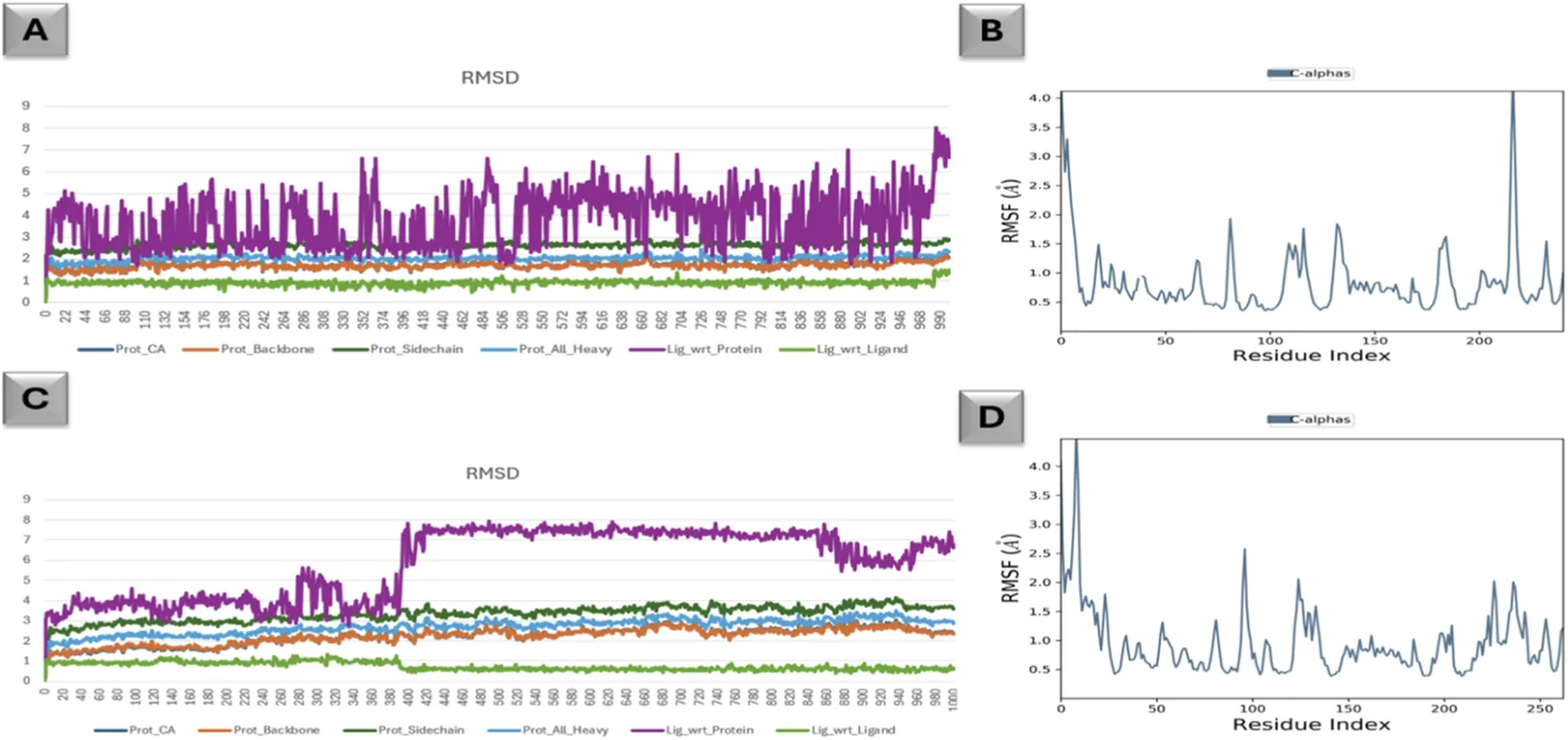
**(A–D)** RMSD and RMSF analysis of SS4 with ALB and SS5 with SRC.

The Root Mean Square Deviation (RMSD) serves as a metric for quantifying the average displacement of designated atoms over a 500 ns simulation period (1,000 frames). This analysis is essential for evaluating the structural stability of the protein-ligand complexes. A deviation of 1–3 Å is generally considered acceptable, indicating that the system has achieved equilibrium. As illustrated in Figure 11A the SS4–ALB complex shows a protein backbone RMSD (orange) and C-alpha RMSD (dark blue) stabilizing between 1.5 Å and 2.0 Å. The ligand-relative-to-protein RMSD (purple) exhibits higher fluctuations, averaging approximately 4.0–5.0 Å, which suggests moderate conformational flexibility of the ligand within the binding pocket. The corresponding RMSF profile Figure 11B highlights stable secondary structural elements, with a notable peak at the C-terminal region reaching 4.0 Å, while the core residues remain well-ordered. In contrast, the SS5–SRC complex Figure 11C demonstrates a distinct transition at approximately 400 ns (frame 800), where the ligand-relative-to-protein RMSD shifts from ∼4.0 Å to a stable plateau at ∼7.5 Å. This transition likely reflects a stable reorientation or adjustment of the ligand within the SRC binding site. The protein backbone and C-alpha RMSD remain remarkably stable throughout the 500 ns duration, hovering near 2.0 Å, indicating that the overall fold of the protein is maintained despite the ligand’s conformational shift. The RMSF analysis for SS5–SRC Figure 11D confirms controlled fluctuations, with a peak around residue 10, underscoring the stability of the binding interface.

**FIGURE 12:**
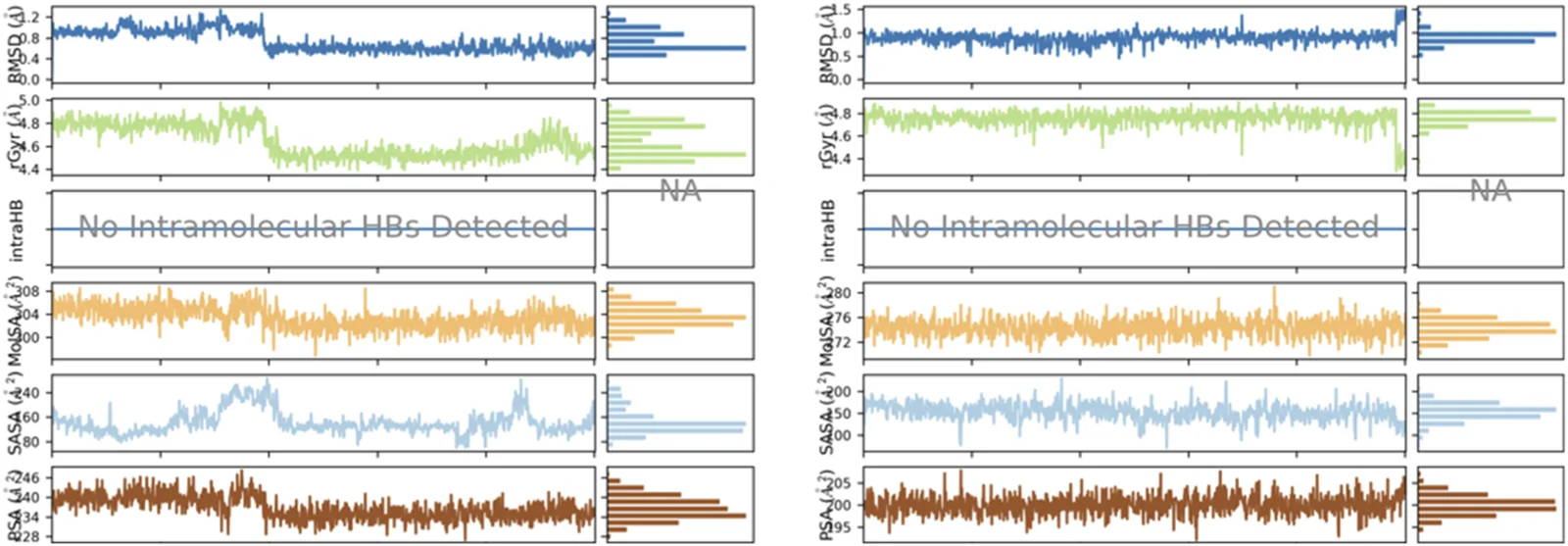
Ligand properties of SS4 with ALB (left) and SS5 with SRC (right).

Further assessment of the radius of gyration (rGyr) and other properties in Figure 12 confirms the compactness and stability of both systems. In the SS4–ALB complex (left column), a structural transition is observed at ∼350 ns (frame 700), evidenced by a drop in rGyr and SASA values, suggesting a more compact state. Conversely, the SS5–SRC complex (right column) maintains a highly consistent profile across all parameters (rGyr, MolSA, SASA, and PSA) over the entire 500 ns trajectory, signifying superior conformational consistency and reduced solvent exposure compared to the SS4–ALB system. No intramolecular hydrogen bonds were detected in either complex, implying that the ligands maintain their binding affinity primarily through intermolecular contacts with the receptor residues. Collectively, these 500 ns simulations provide rigorous evidence for the long-term stability and dynamic interaction patterns of the prioritized lead compounds.

### 2.6 Calculating the binding free energy

The ALB receptor protein’s binding free energy with SS5 was −47.88 kcal/mol, which was comparable to the high binding free energy of −54.38 kcal/mol with SS4. An essential component of the energy that binds a drug and its target together is the van der Waals energy component (ΔE vdw). Precise simulations of these interactions can demonstrate the specificity and adherence of drug molecules to their targets, which can impact their efficacy and safety. With a ΔE vdw value of −35.744 for SS4 and -26.569 for SS5, both compounds demonstrated a high affinity for the target. The ΔG H-bond values for SS5 and SS4 were −0.4937 and −1.3044, respectively. These values indicate the amount of free energy produced by hydrogen bonding interactions shown in Table 6.

**TABLE 6:** Energetic decomposition of MM-GBSA binding free energies for the SS4–ALB and SS5–SRC complexes.

Complex	ΔE_vdW_ (kcal/mol)	ΔE_elec_ (kcal/mol)	ΔG_H bond_ (kcal/mol)	ΔG_gas_ (ΔE_vdW_ + ΔE_elec_) (kcal/mol)	ΔG_sol_ (kcal/mol)	ΔG_bind_ (kcal/mol)
SS4-ALB	−35.744 ± 2.5	−9.5 ± 1.2	−1.304 ± 0.2	−45.24 ± 2.8	−9.14 ± 1.5	−54.38 ± 3.0
SS5-SRC	−26.569 ± 2.1	−8.3 ± 1.0	−0.494 ± 0.1	−34.87 ± 2.3	−13.01 ± 1.6	−47.88 ± 2.8

Previous studies have demonstrated that pinocembrin, a flavanone widely present in several medicinal plants, exhibits significant antiDiabetes, anti-inflammatory, and antioxidant activities (Li et al., 2024; Elbatreek et al., 2023). In experimental models, pinocembrin was shown to reduce hyperglycemia-induced oxidative stress, inhibit NF-κB signaling, and protect vascular endothelium from dysfunction - mechanisms closely aligned with Diabetes retinopathy pathology (Su et al., 2018; Wang et al., 2022). Similarly, stercurensin, a chalcone derivative reported in Syzygium species, has demonstrated free-radical scavenging and glucose-lowering effects in streptozotocin-induced Diabetes models (Adhikari et al., 2025; Uddin et al., 2023). However, their potential roles in Diabetes retinopathy, particularly through modulation of SRC and ALB targets, have not been previously explored. Therefore, our findings provide the first computational evidence linking these bioactive compounds of S. samarangense to molecular mechanisms relevant to retinal oxidative stress and vascular dysfunction, offering new insight for future *in vitro* and *in vivo* validation. The mechanisms by which *Syzygium samarangense* phytochemicals can influence the pathophysiology of DR include several and interconnected ways, suggesting an impact on DR pathology in several different ways., Most importantly, Pinocembrin (SS5) showed a high-affinity with SRC, a non-receptor scaffolding tyrosine kinase that is involved in the vascular endothelial growth factor (VEGF) signaling and the permeability state of the blood-retinal barrier and abnormal angiogenesis, which are the major causes of proliferative DR (Scheppke et al., 2008; Im et al., 2016; Toutounchian et al., 2017). Reduction of SRC activity can decrease pathologic new vascular growth and vascular permeability. In the same way, Stercurensin (SS4) was tightly bound to one of the most abundant plasma proteins that may contribute to antioxidant protection and osmotic regulation, namely, ALB; preventing the destabilization of ALB activity may prevent cellular damage due to oxidative stress in the retina (Medina-Navarro et al., 2014; Watanabe et al., 2025). Hyperglycemia-induced oxidative stress, NF-kB activation and pro-inflammatory cytokine release (ex. TNF-a, IL-6) heavily involve the AGE-RAGE signaling pathway, which was strongly enriched in our KEGG analysis (p < 0.001) (Zong et al., 2011; Jou et al., 2009). The immunoreactivity of the retina with GAPDH, TNF and other inflammatory agents is a sign of potential downregulation of chronic inflammation and retinal apoptosis of neurons. In addition, HIF-1 signaling attenuation implies that it may protect against the overexpression of VEGF induced by hypoxia by reducing pathological angiogenesis (Min et al., 2021; Joussen et al., 2009). Taken together, the above mechanistic considerations relate the described molecular findings to hallmarks of apoptotic potential in DR, including oxidative stress, inflammation, vascular dysfunction, and neovascularization, which would enable a coherent explanation of the potential use of SS5 and SS4 in the therapeutics of DR (Zong et al., 2011).

## 3 Materials and methods

### 3.1 Screening of phytochemicals and drug likeness profiling

We started with the IMPPAT database and literature. From it, we pulled out a group of phytoconstituents found in *S. samarangense* leaves. The PASS online tool let us estimate which of these compounds might have anti-Diabetes effects. For each of the 29 chemicals, we grabbed their standard SMILES notation from PubChem. We wanted clear, consistent chemical identifiers. We checked three key properties: molecular weight (MW), bioavailability (F), and drug-likeness (DL). Molsoft and swissADME handled the virtual verification. Strict cutoffs guided us. A compound qualified as a therapeutic candidate if it had DL greater than 0.18, F above 30%, and MW below 500 Da. Meaning: high promise, good absorption, manageable size. As far as safety is concerned, we looked at sensitive liver biomarkers. This ensures that any dose aiming to treat Diabetes retinopathy will not trigger harmful effects in the liver or set off unwanted biological pathways (Mohanraj et al., 2018; Daina et al., 2017; Kim et al., 2025).

### 3.2 Active phytocompounds of SS-target screening

We gathered data on SS components using “IMPPAT: Indian Medicinal Plants, Phytochemistry and Therapeutics (https://cb.imsc.res.in/imppat)”. The search term was “*S. samarangense*”. Criteria for picking bioactive chemicals were strict: oral bioavailability (OB) of at least 30%. Each compound needed to fit Lipinski’s rule of five (ro5) and have a drug-likeness (DL) score no lower than 0.18. No shortcuts. Next, we pulled information about the targets of these active compounds from the same IMPPAT platform. The chemical composition of the Syzygium samarangense leaf extract (SS-EA) was characterized using gas chromatography-mass spectrometry (GC-MS) on a Shimadzu GCMS-QP2010 Plus system. Compounds were identified by matching mass spectra with the NIST14 and WILEY8 libraries. This experimental profiling confirmed the presence of major bioactive constituents, including various flavonoids and terpenoids such as 5,7-dihydroxy-2-phenyl-2,3-dihydro-4H-chromen-4-one and caryophyllene, which were subsequently used for *in silico* target screening. The compounds were screened for drug-likeness and their biological targets were predicted using the Swiss Target Prediction database. Only the relevant targets made the cut. We chose them as sensitive hepatic biomarkers. Our goal is to rule out any candidates that, at the necessary therapeutic dose for Diabetes retinopathy, could harm the liver or trigger pathways known from literature to connect with adverse effects as interpreted by the PK/PD model.

### 3.3 Diabetes retinopathy related targets screening

For each screened phytochemical, the PubChem database was utilized to obtain the canonical SMILES notation, and the ADME properties of each of the 29 phytocompounds were calculated using SwissADME (http://www.swissadme.ch). Moreover, drug-related targets were identified utilizing the Swiss Target Prediction database (Daina et al., 2019). This strategy is employed to elucidate the molecular pathways that govern a specific phenotype or bioactivity, to justify anticipated side effects, or to anticipate off-target interactions of each molecule. To evaluate the relevance of these protein targets to Diabetes Retinopathy (DM), we employed the DisGeNET database (https://disgenet.com) and the GeneCards database (https://www.genecards.org) (Piñero et al., 2019; Safran et al., 2021).

### 3.4 Active phytocompounds-DR target and PPI network construction

The potential active phytoconstituents of *S. samarangense* (SS) leaves, identified as having overlap between SS targets and Diabetes retinopathy (DR)-associated targets, were defined as SS-DR common targets. This intersection was determined using the InteractiVenn web tool (https://www.interactivenn.net). To construct a compound-target interaction network, Cytoscape software (version 3.7.2) was employed, mapping the active compounds from SS to their respective target proteins. In this network, nodes represent DR-related targets, shared targets, and SS phytocompound targets, while the edges (lines) denote the interactions between them. The significance of each node is indicated by its degree, i.e., the number of edges it connects with. To further analyze protein–protein interactions (PPI), the common targets were uploaded to the STRING database (version 11.0), with the organism set to *Homo sapiens* and the confidence score threshold fixed at 0.950 to ensure high-accuracy data (Szklarczyk et al., 2023). The resulting PPI network was then visualized using Cytoscape (Sahu et al., 2024; Lazzara et al., 2024; Otasek et al., 2019; Shannon et al., 2003).

### 3.5 KEGG and GO enrichment analyses

To gain insight into the functional roles of the SS-DR common targets, Gene Ontology-(GO) enrichment and Kyoto Encyclopedia of Genes and Genomes-(KEGG) pathway analyses were performed using the DAVID-(Database for Annotation, Visualization, and Integrated Discovery) bioinformatics platform (https://david.ncifcrf.gov/tools.jsp) (Kanehisa and Goto, 2000; Kanehisa et al., 2025; Sherman et al., 2022). Gene Ontology enrichment analysis covered three main categories: Biological Process-(BP), Cellular Component-(CC), and Molecular Function-(MF). Both GO terms and KEGG pathways with a significance threshold of P < 0.05 were visualized using bubble and bar charts, which highlighted gene counts, gene ratios, adjusted p-values, and other key annotations. The results of the KEGG analysis were further used to construct a pathway-target interaction network, helping to pinpoint crucial proteins potentially involved in the therapeutic mechanisms of *S. samarangense* (SS) against Diabetes retinopathy (DR). The molecular processes of SS in the setting of DR treatment were further clarified by retrieving more details regarding the enriched pathways and core targets from online databases (Zhang et al., 2023; Liu et al., 2023; Pu et al., 2025; Zhang et al., 2025).

### 3.6 Molecular docking studies

Molecular docking is a useful way to make lead compounds better and find new ones. It’s not new. For over 30 years, researchers have used different search methods and scoring systems to improve how these docking programs work. In this study, after identifying promising compounds, we used AutoDock4 (version 4.2.6) (Morris et al., 2009) to explore these possibilities, targeting DR. Think of molecular docking as a smart computer tool for predicting how drugs and their targets interact. It helps us design new drugs, too. Here’s how: the docking process models how small molecules and proteins might bind together in 3D space. This shows us which docking positions and binding strengths might matter most. We picked out important target proteins linked to Diabetes retinopathy based on where they sit in the protein–protein interaction network and their known role in the disease. Active compounds from *S. samarangense* (SS) and their target proteins were chosen for docking. We pulled the 3D structure of these compounds from the IMPPAT database, and protein structures from the RCSB Protein Data Bank (http://www.rcsb.org/) (Berman et al., 2000). Before docking, we prepped all molecules; that meant adding hydrogens and removing any water. Docking itself used AutoDock version 4.2.6. We did not know the exact binding sites for SS compounds on the Diabetes retinopathy-related targets. So, we predicted likely binding pockets using DeepSite (https://www. playmolecule.com), making sure docking only happened in those areas. Finally, we used PyMOL (version 2.4) for visualizing all the results (Deivasigamani et al., 2024; Mhetre et al., 2024; Nayak et al., 2024).

### 3.7 Molecular dynamics simulation

Molecular dynamics (MD) simulations happened inside Desmond version 5.9, crafted by Schrödinger LLC, running on a Linux-based Ubuntu system. Simple, direct, robust. This method allowed for a close look at how the protein-ligand complexes held up and moved under conditions similar to what they’d face in a living cell. It’s about understanding their dance, not just their pose. To keep things reasonable, simulations ran for 500 nanoseconds (ns)—long enough to spot key molecular interactions without exhausting resources (Majie et al., 2024; Majie et al., 2026; Jha et al., 2025). This window is a standard choice in MD research. Before simulation, the picked protein-ligand complex—saved as a PDB file—underwent energy minimization using the OPLS-2005 force field. You want the most comfortable starting point, not a tangled mess. The minimized structure sat at the center of a predefined orthorhombic box, ready for the next step. Solvation used the TIP3P water model, ensuring at least 10 Å between the complex and box borders. This gap keeps the molecules from bumping into the simulation’s edges. To maintain a neutral system, sodium (Na^+^) and chloride (Cl^−^) counterions were added based on the system’s total charge (Saravanan et al., 2024; Chagaleti et al., 2023; Saravanan et al., 2025).

Equilibration involved both NVT (fixed number of particles, volume, and temperature) and NPT (fixed number of particles, pressure, and temperature) settings. First, restraints held the heavy atoms in place so the solvent could settle around them—think of it as letting the water find its calm before stirring the system. The temperature was carefully raised and held around 300 K, while pressure stabilized at 1 atm, all within a 20-picosecond relaxation period. Once conditions were steady, restraints were lifted. The main MD run—500 ns under NPT conditions—was followed, using standard parameters. After the simulation, the results were not left to guesswork. The trajectory was assessed using the simulation interaction diagram tool inside Desmond. This step measured important features: root mean square deviation (RMSD), root mean square fluctuation (RMSF), hydrogen bonds, and other interactions. Each metric told its part of the story, helping clarify how stable the protein-ligand complex was during its simulated journey (Packiapalavesam et al., 2024; Mukhrish et al., 2025).

### 3.8 Binding free energy calculation

To determine the binding free energy of the ALB and SRC protein receptor-ligand complexes, the MM-GBSA method came into play. We selected MM-GBSA (Prime/VSGB) because it allows efficient ensemble rescoring directly from MD snapshots within the Schrödinger suite, is computationally tractable for multiple complexes and repeated sampling, and produces energetics consistent with our MD/force-field setup. While MM-PBSA can offer slightly different polar solvation estimates (via a Poisson–Boltzmann model), it is considerably more computationally expensive for large ensembles and requires external PB solvers. We therefore used MM-GBSA for the main analysis and report detailed decomposition with statistic. This tool is bundled inside the Prime module of the Schrödinger Suite. Reliable, tried, tested. Before calculating energies, the OPLS 2005 force field minimized the energy of each protein-ligand pairing. It set the stage for accurate results. For capturing the nuances of polar solvation, the VSGB model was used. It accounted for solvent effects—often invisible, yet critical. The method maps the environment around each molecular pair with clarity. Then, both the solvent-accessible surface area (SASA) and van der Waals terms tackled nonpolar solvation energies. Together, these elements formed a complete picture of the MD complex’s binding free energy (ΔGbind). More negative ΔG values mean stronger binding affinity. Simple: the more negative, the tighter the bond, which gives us a clear look at how durable and effective the noncovalent interactions are between receptor and ligand (Shaker et al., 2022; Gupta et al., 2022; Dalal et al., 2021).

## 4 Conclusion

This integrated study demonstrated the potential of *S. samarangense* (SS) as a natural therapeutic agent against Diabetes retinopathy through a network pharmacology and molecular simulation-based approach. By identifying and validating key phytoconstituents and their targets, particularly Pinocembrin (SS5) and Stercurensin (SS4), the study provided molecular evidence of strong binding interactions with key DR-associated proteins, SRC and ALB. Molecular dynamics simulations (MD) confirmed the stability of these complexes, with SS5–SRC exhibiting notable conformational stability and tighter binding, as supported by favorable binding free energy values. These results highlight the multi-pathway and multi-target mechanism of SS in modulating DR-related pathways such as AGE-RAGE and HIF-1 signaling. Collectively, the findings offer a solid foundation for future experimental (*in-vitro* and *in-vivo*) validation and development of SS-derived compounds as safe and effective candidates in DR therapy. To ensure the robustness and reliability of our predictions, the research utilized a multi-layer computational pipeline that incorporated dataset filtering, high-confidence target mapping, pocket-guided docking, molecular dynamics with a duration of 500 nanoseconds, and an evaluation of MM-GBSA free energy. The false-positive docked poses are reduced to a minimum by utilizing this stepwise validation strategy, and it is certain that the identification of lead compounds will be based on converging biophysical evidence instead of relying solely on docking. As a result, the research provides a foundation that is both computationally rigorous and mechanistically justified for prioritizing SS5 and SS4 as promising lead molecules for subsequent biological evaluation in Diabetes retinopathy. Beyond the immediate findings, this work provides a scalable, computationally rigorous, and mechanistically justified platform that significantly reduces the search space for experimentalists. By prioritizing these specific candidates, we establish a reliable foundation for future *in vitro* and *in vivo* studies, such as in ovo CAM angiogenesis assays, to confirm the translational viability of these promising phytochemicals.

## Data Availability

The raw data supporting the conclusions of this article will be made available by the authors, without undue reservation.
