# Supplementary material for: Integrative network pharmacology and molecular modeling approaches reveal the therapeutic potential of Syzygium samarangense phytocompounds against diabetes retinopathy
Source: Front Bioinform. 2026 Aug 18;6:1884743. doi: 10.3389/fbinf.2026.1884743 (PMC13529976; doi:10.3389/fbinf.2026.1884743)

Sample Information

Analyzed by	: Admin
Analyzed	: 28/03/2025 14:58:34
Sample Name	: SS-EA
Sample ID	: 40674
Injection Volume	: 1.00
Data File	: F:\GCMS-2025\MARCH\40674\SS-EA.qgd
Method File	: C:\GCMSsolution\Data\Project1\General\SAMPLE.qgm

Method

[Comment]

===== Analytical Line 1 =====

[GC-2010]

Column Oven Temp.	:50.0 °C
Injection Temp.	:250.00 °C
Injection Mode	:Split
Flow Control Mode	:Linear Velocity
Pressure	:53.5 kPa
Total Flow	:54.0 mL/min
Column Flow	:1.00 mL/min
Linear Velocity	:36.3 cm/sec
Purge Flow	:3.0 mL/min
Split Ratio	:50.0
High Pressure Injection	:OFF
Carrier Gas Saver	:ON
Carrier Gas Saver Split Ratio	:5.0
Carrier Gas Saver Time	:1.00 min
Splitter Hold	:OFF

Oven Temp. Program

Rate	Temperature(°C)	Hold Time(min)
-	50.0	1.00
10.00	280.0	2.00

< Ready Check Heat Unit >

Column Oven	: Yes
SPL1	: Yes
MS	: Yes

< Ready Check Detector(FTD/BID) >
< Ready Check Baseline Drift >
< Ready Check Injection Flow >

SPL1 Carrier	: Yes
SPL1 Purge	: Yes

< Ready Check APC Flow >
< Ready Check Detector APC Flow >

External Wait	:No
Equilibrium Time	:0.3 min

[GC Program]

[GCMS-QP2010 Plus]

IonSourceTemp	:200.00 °C
Interface Temp.	:280.00 °C
Solvent Cut Time	:3.00 min
Detector Gain Mode	:Relative to the Tuning Result
Detector Gain	:1.01 kV +0.10 kV
Threshold	:1000

[MS Table]
--Group 1 - Event 1--

Start Time	:3.50min
End Time	:26.00min
ACQ Mode	:Scan
Event Time	:0.30sec
Scan Speed	:3333
Start m/z	:40.00
End m/z	:900.00

Sample Inlet Unit	:GC

[MS Program]

Use MS Program	:OFF

Chromatogram SS-EA F:\GCMS-2025\MARCH\40674\SS-EA.qgd

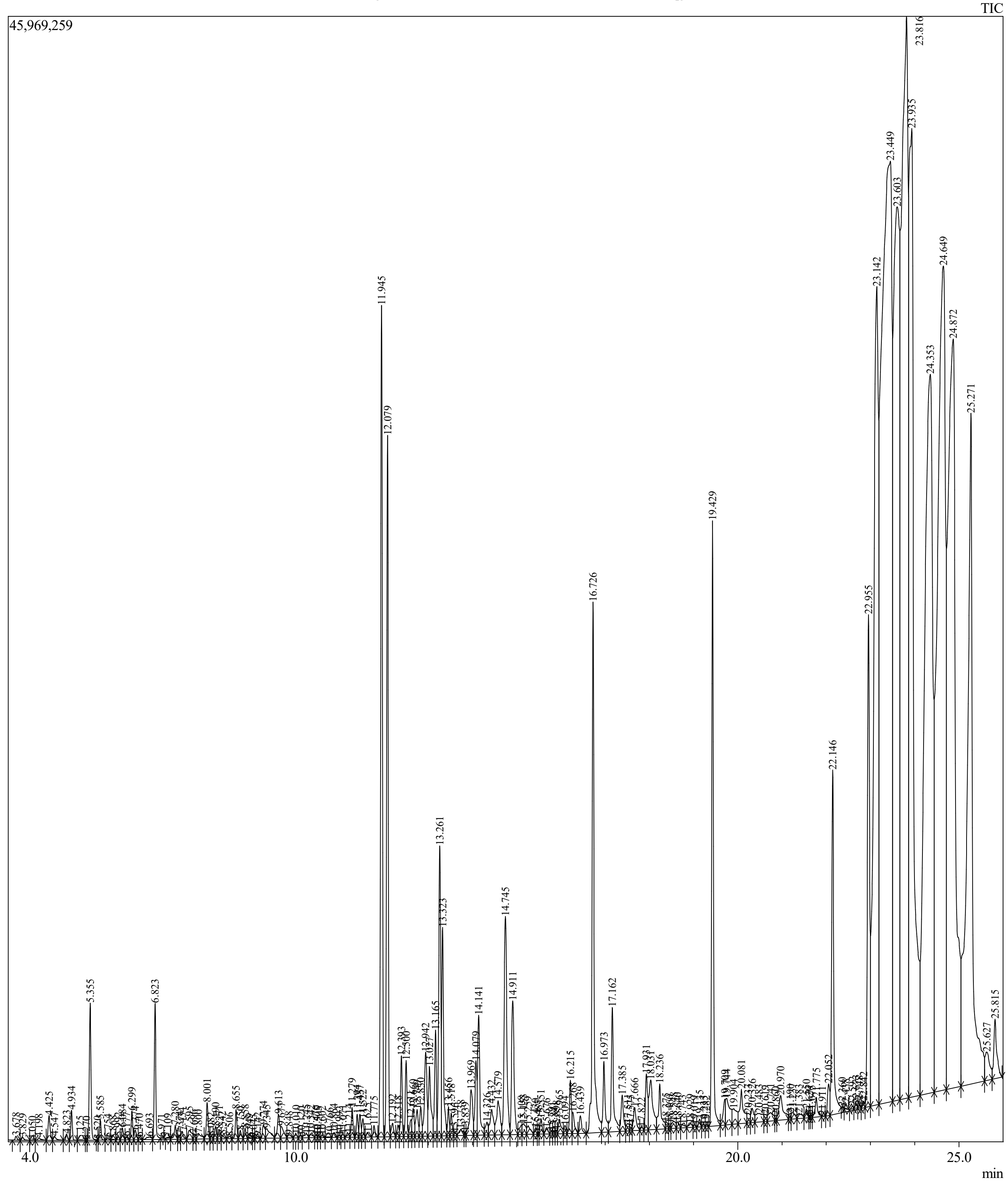

Chromatogram SS-EA F:\GCMS-2025\MARCH\40674\SS-EA.qgd

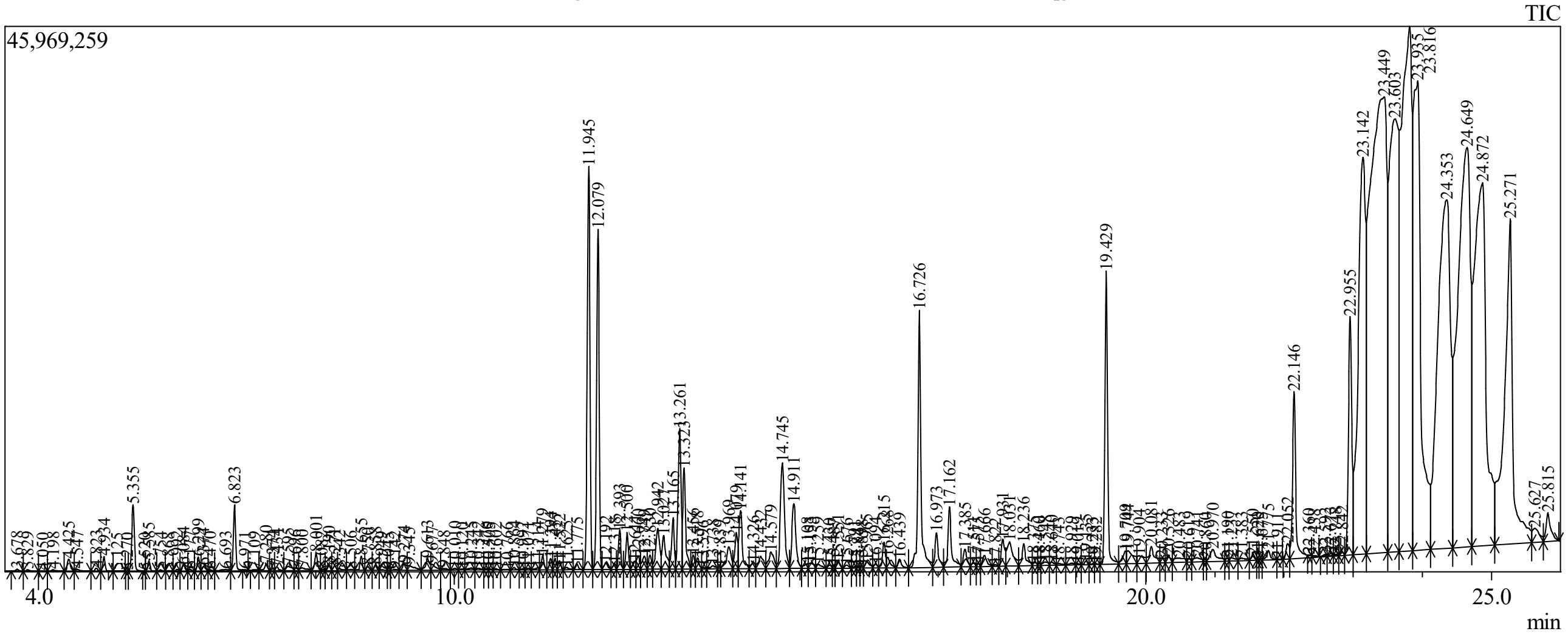

Peak Report TIC

Peak#	R.Time	Area	Area%	Height	Name
1	3.678	467815	0.01	159235	2-PENTANONE, 4-HYDROXY-4-METHYL-
2	3.829	406881	0.01	114698	.alpha.-Phellandrene
3	4.050	46014	0.00	7549	ACETIC ACID ETHYL ESTER
4	4.198	513815	0.01	106412	1,3-CYCLOHEXADIENE, 1-METHYL-4-(1-METHYLETHYL)-
5	4.425	3229729	0.07	1013719	o-Cymene
6	4.547	773983	0.02	163840	CYCLOHEXENE, 1-METHYL-4-(1-METHYLETHENYL)-
7	4.823	715464	0.02	258057	.alpha.-Pinene
8	4.934	2849813	0.07	1234689	.alpha.-Pinene
9	5.125	96177	0.00	17611	CYCLOPROPENE, 1-METHYL-3-(2-METHYLCYCLOPROPYL)-
10	5.270	4410	0.00	2631	BUTANEDIAL
11	5.355	14998339	0.34	5597698	1,4-CYCLOHEXADIENE, 1-METHYL-4-(1-METHYLETHYL)-
12	5.520	4041	0.00	3092	2,5-Dimethyl-1,5-hexadien-3-ol
13	5.585	2019729	0.05	845111	.beta.-Pinene
14	5.754	258629	0.01	90530	1,6-OCTADIENE, 7-METHYL-3-METHYLENE-
15	5.868	84649	0.00	25634	NONANE
16	5.992	360314	0.01	141634	1,3-CYCLOHEXADIENE, 2-METHYL-5-(1-METHYLETHYL)-
17	6.084	1433103	0.03	488198	CYCLOHEXENE, 1-METHYL-4-(1-METHYLETHYLIDENE)-
18	6.177	428856	0.01	134192	1,3-CYCLOHEXADIENE, 1-METHYL-4-(1-METHYLETHYL)-
19	6.299	2862763	0.07	1167054	Benzene, 1-methyl-3-(1-methylethyl)-
20	6.374	1320723	0.03	391438	1,6-OCTADIEN-3-OL, 3,7-DIMETHYL-
21	6.470	282543	0.01	88970	(1S)-2,6,6-Trimethylbicyclo[3.1.1]hept-2-ene
22	6.693	906336	0.02	108468	2,3-Dioxabicyclo[2.2.1]heptane, 1-methyl-
23	6.823	14060777	0.32	5565502	1,4-CYCLOHEXADIENE, 1-METHYL-4-(1-METHYLETHYL)-
24	6.971	35851	0.00	16807	Bicyclo[3.1.0]hexan-2-ol, 2-methyl-5-(1-methylethyl)-, (1.alpha.,2.beta.,5.alph
25	7.109	108038	0.00	28905	2-CYCLOHEXEN-1-ONE, 3,5-DIMETHYL-, O-METHYLOXIME
26	7.280	1887031	0.04	576618	1-METHYL-4-(1-METHYLETHYLIDENE)-1-CYCLOHEXENE
27	7.374	562164	0.01	171146	CYCLOHEXANOL, 5-METHYL-2-(1-METHYLETHENYL)-, [1R-(1.ALPH
28	7.434	1083807	0.02	318499	CYCLOHEXENE, 1-METHYL-4-(1-METHYLETHYLIDENE)-
29	7.595	2131452	0.05	360288	1,2,3-Propanetriol, 1-acetate
30	7.696	783057	0.02	218399	Bicyclo[2.2.1]heptan-2-ol, 1,3,3-trimethyl-, (1R-endo)-
31	7.800	1629805	0.04	227812	BICYCLO[2.2.1]HEPTAN-2-OL, 1,7,7-TRIMETHYL-, (1S-ENDO)-
32	8.001	4365662	0.10	1486495	3-CYCLOHEXEN-1-OL, 4-METHYL-1-(1-METHYLETHYL)-
33	8.082	346560	0.01	106746	4-Isopropyl-1-methylcyclohex-2-enol
34	8.190	1456310	0.03	502471	1-Dodecene
35	8.271	1207287	0.03	387485	Terpineol
36	8.342	620831	0.01	153643	TETRADECANE
37	8.506	389966	0.01	94999	Isoborneol
38	8.655	2963579	0.07	1179316	3-CYCLOHEXEN-1-OL, 4-METHYL-1-(1-METHYLETHYL)-
39	8.750	1045108	0.02	333454	1-Dodecene
40	8.858	1143001	0.03	422106	3-CYCLOHEXENE-1-METHANOL, .ALPHA.,.ALPHA.,4-TRIMETHYL-
41	8.949	174043	0.00	44765	1-Nonene
42	9.045	18866	0.00	10604	BICYCLO[3.1.0]HEXAN-3-OL, 4-METHYLENE-1-(1-METHYLETHYL)-,
43	9.142	258952	0.01	78282	.ALPHA.-IONONE, METHYL-
44	9.274	2106175	0.05	547150	1,2,3-PROPANETRIOL, DIACETATE
45	9.345	2716393	0.06	386678	2-BUTANONE, 3-METHYL-
46	9.613	3842074	0.09	968715	1,2,3-PROPANETRIOL, DIACETATE
47	9.677	2319907	0.05	471943	1,2,3-PROPANETRIOL, 1,2-DIACETATE
48	9.848	740478	0.02	127104	2 OCTENAL
49	10.010	537457	0.01	135199	2,3-DIOXABICYCLO[2.2.2]OCT-5-ENE, 1-METHYL-4-(1-METHYLETHY
50	10.120	358921	0.01	82858	dl-Mevalonic acid lactone

Peak#	R.Time	Area	Area%	Height	Name
51	10.245	1008839	0.02	164218	Ascaridole epoxide
52	10.342	772144	0.02	154531	2-FURANMETHANOL, 5-ETHENYLTETRAHYDRO-.ALPHA.,.ALPHA.,5-
53	10.470	342787	0.01	108052	NAPHTHALENE, 1,2,3,4,4A,5,6,7-OCTAHYDRO-4A-METHYL-
54	10.505	216353	0.00	91628	(1S,2R,4R,7R)-4-Isopropyl-7-methyl-3,8-dioxatricyclo[5.1.0.02,4]octane
55	10.602	283678	0.01	98202	2-FURANMETHANOL, 5-ETHENYLTETRAHYDRO-.ALPHA.,.ALPHA.,5-
56	10.766	164521	0.00	27671	(-)-NEPETALACTOL
57	10.864	592755	0.01	190299	.alpha.-Cubebene
58	10.992	310183	0.01	83267	1,2,3-PROPANETRIOL, TRIACETATE
59	11.071	455726	0.01	141807	1H-CYCLOPENTA[1,3]CYCLOPROPA[1,2]BENZENE, 3A,3B,4,5,6,7-HEX
60	11.214	652751	0.01	162138	.alpha.-ylangene
61	11.279	3286980	0.08	1217076	TRICYCLO[4.4.0.0(2,7)]DEC-3-ENE, 1,3-DIMETHYL-8-(1-METHYLETH
62	11.394	2484348	0.06	935205	1-TETRADECENE
63	11.457	2420518	0.06	937895	TRICYCLO[4.4.0.0(2,7)]DEC-3-ENE, 1,3-DIMETHYL-8-(1-METHYLETH
64	11.522	2021880	0.05	753783	1-TETRADECENE
65	11.625	797164	0.02	191913	TETRADECANE
66	11.775	1290280	0.03	470115	1H-Cycloprop[e]azulene, 1a,2,3,4,4a,5,6,7b-octahydro-1,1,4,7-tetramethyl-, [1
67	11.945	93048550	2.13	33960709	Caryophyllene
68	12.079	73798003	1.69	28648115	Caryophyllene
69	12.192	2209471	0.05	549684	1H-CYCLOPROP[E]AZULENE, DECAHYDRO-1,1,7-TRIMETHYL-4-ME
70	12.318	1453420	0.03	476882	1H-CYCLOPROP[E]AZULENE, DECAHYDRO-1,1,7-TRIMETHYL-4-ME
71	12.393	8297023	0.19	3292685	Humulene
72	12.500	8014827	0.18	3099063	Humulene
73	12.611	2192354	0.05	682479	BICYCLO[7.2.0]UNDEC-4-ENE, 4,11,11-TRIMETHYL-8-METHYLENE-,
74	12.660	3454535	0.08	1136709	Naphthalene, 1,2,3,5,6,7,8,8a-octahydro-1,8a-dimethyl-7-(1-methylethenyl)-, [
75	12.749	4210041	0.10	1097057	NAPHTHALENE, 1,2,3,5,6,7,8,8A-OCTAHYDRO-1,8A-DIMETHYL-7-(1-M
76	12.830	3614447	0.08	1191870	Naphthalene, 1,2,3,4,4a,5,6,8a-octahydro-4a,8-dimethyl-2-(1-methylethenyl)-,
77	12.942	13513128	0.31	3421467	Guaia-1(10),11-diene
78	13.027	11089565	0.25	2838155	1H-CYCLOPROP[E]AZULENE, DECAHYDRO-1,1,7-TRIMETHYL-4-ME
79	13.165	11717940	0.27	4327660	(1S,4aR,8aS)-1-Isopropyl-7-methyl-4-methylene-1,2,3,4,4a,5,6,8a-octahydron
80	13.261	36526779	0.83	11846637	NAPHTHALENE, 1,2,3,5,6,8A-HEXAHYDRO-4,7-DIMETHYL-1-(1-METH
81	13.323	23784694	0.54	8517044	NAPHTHALENE, 1,2,3,5,6,8A-HEXAHYDRO-4,7-DIMETHYL-1-(1-METH
82	13.456	3083278	0.07	1173237	.alpha.-Muurolene
83	13.518	2645607	0.06	926875	NAPHTHALENE, 1,2,4A,5,6,8A-HEXAHYDRO-4,7-DIMETHYL-1-(1-MET
84	13.596	406132	0.01	130599	1-ISOPROPYL-4,7-DIMETHYL-1,2-DIHYDRONAPHTHALENE #
85	13.738	1454482	0.03	264142	DODECANOIC ACID
86	13.839	383352	0.01	154387	1H-Cycloprop[e]azulen-4-ol, decahydro-1,1,4,7-tetramethyl-, [1aR-(1a.alpha.,4
87	13.969	8776473	0.20	1859923	Cetene
88	14.079	9252392	0.21	3042393	(-)-5-OXATRICYCLO[8.2.0.0(4,6)]DODECANE,,12-TRIMETHYL-9-METH
89	14.141	16385775	0.37	4901741	1,2-BENZENEDICARBOXYLIC ACID, DIETHYL ESTER
90	14.326	2250074	0.05	524972	1H-CYCLOPROP[E]AZULEN-4-OL, DECAHYDRO-1,1,4,7-TETRAMETH
91	14.432	5644741	0.13	1046595	Cubenol
92	14.579	7726062	0.18	1391999	Epicubenol
93	14.745	37797002	0.86	8912442	.tau.-Cadinol
94	14.911	21929202	0.50	5457231	.alpha.-Cadinol
95	15.108	1698667	0.04	384109	Androstan-17-one, 3-ethyl-3-hydroxy-, (5.alpha.)-
96	15.153	2314425	0.05	441693	Dodecyl acrylate
97	15.259	1885790	0.04	348095	3-BUTEN-2-OL, 4-(2,6,6-TRIMETHYL-1-CYCLOHEXEN-1-YL)-
98	15.430	963581	0.02	268110	PHENOL, 2,6-DIMETHOXY-4-(2-PROPENYL)-
99	15.487	486368	0.01	210530	3,7,11,15-TETRAMETHYL-2,6,10,14-HEXADECATETRAENYL ACETATE
100	15.551	2105745	0.05	633008	trans-Farnesol
101	15.676	1126441	0.03	256797	1H-Cycloprop[e]azulen-4-ol, decahydro-1,1,4,7-tetramethyl-, [1aR-(1a.alpha.,4
102	15.791	385298	0.01	122961	2,6,10-Dodecatrienal, 3,7,11-trimethyl-, (E,E)-
103	15.841	310638	0.01	111344	(-)-5-OXATRICYCLO[8.2.0.0(4,6)]DODECANE,,12-TRIMETHYL-9-METH
104	15.898	443705	0.01	163912	3-BUTEN-2-ONE, 4-(6,6-DIMETHYL-1-CYCLOHEXEN-1-YL)-
105	15.965	2657071	0.06	597197	OCTADECANOIC ACID
106	16.094	1136456	0.03	338222	Pregn-4-ene-1,20-dione, 16,17-dimethyl-
107	16.215	6342138	0.14	2183461	E-15-Heptadecenal
108	16.268	3120036	0.07	877846	2(4H)-BENZOFURANONE, 5,6,7,7A-TETRAHYDRO-6-HYDROXY-4,4,7A
109	16.439	2546218	0.06	718035	(S,E)-4-Hydroxy-3,5,5-trimethyl-4-(3-oxobut-1-en-1-yl)cyclohex-2-enone
110	16.726	67144073	1.53	21682015	Neophytadiene
111	16.973	10078988	0.23	2893860	3,7,11,15-Tetramethyl-2-hexadecen-1-ol
112	17.162	17903777	0.41	5084080	3,7,11,15-Tetramethyl-2-hexadecen-1-ol
113	17.385	5598739	0.13	1478256	4,4,8-Trimethyltricyclo[6.3.1.0(1,5)]dodecane-2,9-diol
114	17.511	1361482	0.03	306746	5-(3-Hydroxypropyl)-2,3-dimethoxyphenol
115	17.575	864932	0.02	273228	2-Piperidinone, N-[4-bromo-n-butyl]-
116	17.666	4144452	0.09	946276	7-Oxabicyclo[4.1.0]heptan-3-ol, 6-(3-hydroxy-1-butenyl)-1,5,5-trimethyl-
117	17.822	519583	0.01	133249	Isophytol
118	17.931	7860418	0.18	2286767	1-PENTENE, 4-(M-CHLOROPHENYL)-2-METHYL-
119	18.031	12751707	0.29	2030631	n-Hexadecanoic acid
120	18.236	8064991	0.18	1846744	1-Nonadecene
121	18.375	1079863	0.02	273673	2-TRIDECENAL, (Z)-
122	18.460	567474	0.01	191540	NAPHTHALENE, DECAHYDRO-1,4A-DIMETHYL-7-(1-METHYLETHYL
123	18.548	1743179	0.04	288308	2-(2-CARBOXY-ETHYL)-6,6-DIMETHYL-3-OXO-CYCLOHEX-1-ENECA
124	18.640	1169925	0.03	261712	3-PHENYL-PROPIONIC ACID CYCLOPENTYLIDENE-HYDRAZIDE
125	18.743	848097	0.02	146330	3-PHENYL-PROPIONIC ACID CYCLOPENTYLIDENE-HYDRAZIDE
126	18.929	859719	0.02	157432	9-OCTADECEN-1-OL, (Z)-
127	19.019	291311	0.01	100961	3-HEPTYL 2-ETHYLHEXANOATE
128	19.135	931142	0.02	318349	1-Eicosanol
129	19.233	292855	0.01	66139	9-OCTADECYNOIC ACID, METHYL ESTER
130	19.282	193361	0.00	60913	9-Octadecenoic acid (Z)-, methyl ester

Peak#	R.Time	Area	Area%	Height	Name
131	19.429	67150653	1.53	24723569	Phytol
132	19.709	3936535	0.09	1052485	PALMITALDEHYDE, DIALLYL ACETAL
133	19.744	7248432	0.17	1081537	9-OCTADECENOIC ACID (Z)-
134	19.904	4568203	0.10	630732	9,12-Octadecadienoic acid (Z,Z)-
135	20.081	7796156	0.18	1401867	1-Nonadecene
136	20.233	1775538	0.04	391006	Heptadecyl acetate
137	20.326	2539347	0.06	587790	Isophytol, acetate
138	20.483	3935810	0.09	346546	1-ETHOXY-7-(2-PHENYLETHENYLIDENE)BICYCLO[4.1.0]HEPTANE
139	20.619	1224523	0.03	284652	Hexadecanoic acid, 1-(hydroxymethyl)-1,2-ethanediyl ester
140	20.741	2804834	0.06	344478	9-OCTADECEN-1-OL, (Z)-
141	20.860	527355	0.01	195886	CHOLESTAN-3-OL, 2-METHYLENE-, (3.BETA.,5.ALPHA.)-
142	20.970	6961487	0.16	1008340	BENZENE, (2-DECYLDODECYL)-
143	21.190	790301	0.02	237844	1,16-DICHLOROHEXADECANE
144	21.237	1595232	0.04	270638	7-Hexadecenal, (Z)-
145	21.383	1529263	0.03	206794	2,6,6,9,2',6',6',9'-OCTAMETHYL-[8,8']BI[TRICYCLO[5.4.0.0 2,9]UNDECY
146	21.550	1357270	0.03	373703	4,8,12,16-Tetramethylheptadecan-4-olide
147	21.620	198658	0.00	96069	4-DECENOIC ACID, ETHYL ESTER, (Z)-
148	21.675	273174	0.01	106350	Methyl steviol
149	21.775	3215502	0.07	808701	1-HEXACOSENE
150	21.911	89896	0.00	38618	ACETIC ACID, DODECYL ESTER
151	22.052	4569078	0.10	1293996	2,3-Dihydro-5-hydroxy-6,8,8-trimethyl-2-phenyl-4H-1-benzopyran-4,7(8H)-di
152	22.146	48015442	1.10	14082695	5-hydroxy-7-methoxyflavanone
153	22.360	51760	0.00	30249	.alpha.-Cadinol
154	22.438	67741	0.00	26942	<NO NAME>
155	22.592	327881	0.01	112709	Bromoacetic acid, hexadecyl ester
156	22.673	549549	0.01	169928	Alantolactone, 4.alpha.,4A.alpha.-epoxy-
157	22.748	228879	0.01	85937	2,6,6,9,2',6',6',9'-OCTAMETHYL-[8,8']BI[TRICYCLO[5.4.0.0 2,9]UNDECY
158	22.842	875032	0.02	273796	5H-INDEN-5-ONE, 1,2,3,6,7,7A-HEXAHYDRO-1-HYDROXY-7A-METHY
159	22.955	67894780	1.55	20095600	4H-1-Benzopyran-4-one, 2,3-dihydro-5,7-dimethoxy-2-phenyl-
160	23.142	251432223	5.75	33420583	1H-BENZIMIDAZOL-2-YL(DIPHENYL)METHANOL #
161	23.449	644456283	14.73	38423734	4H-1-Benzopyran-4-one, 2,3-dihydro-5,7-dihydroxy-2-phenyl-, (S)-
162	23.603	347614547	7.95	36496924	5,7-DIHYDROXY-6,8-DIMETHYL-2-PHENYL-2,3-DIHYDRO-4H-CHROM
163	23.816	464698852	10.62	44187753	4H-1-Benzopyran-4-one, 2,3-dihydro-5-hydroxy-7-methoxy-2-phenyl-, (S)-
164	23.935	307586042	7.03	39567626	5,7-DIHYDROXY-6,8-DIMETHYL-2-PHENYL-2,3-DIHYDRO-4H-CHROM
165	24.353	380232889	8.69	29358354	1H-BENZOIMIDAZOLE, 5-ETHOXY-2-PHENETHYLSULFANYL-
166	24.649	427564805	9.77	33661545	SPIRO[4H-1-BENZOPYRAN-4,2'-[1,3]DITHIAN]-5'-ONE, 6-FLUORO-2,3-
167	24.872	370274532	8.46	30594280	2,3-Dihydro-5-hydroxy-6,8,8-trimethyl-2-phenyl-4H-1-benzopyran-4,7(8H)-di
168	25.271	232597138	5.32	27397375	(E)-1-(2-Hydroxy-4,6-dimethoxyphenyl)-3-phenylprop-2-en-1-one
169	25.627	8479366	0.19	1169270	.alpha.-Tocospiro B
170	25.815	11454232	0.26	2431682	CELIDONIOL, DEOXY-
		4374999799	100.00	603316113	

Library

<< Target >>
Line#:1 R.Time:3.680(Scan#:37) MassPeaks:25
RawMode:Averaged 3.675-3.685(36-38) BasePeak:43.00(93227)
BG Mode:Calc. from Peak Group 1 - Event 1 Scan

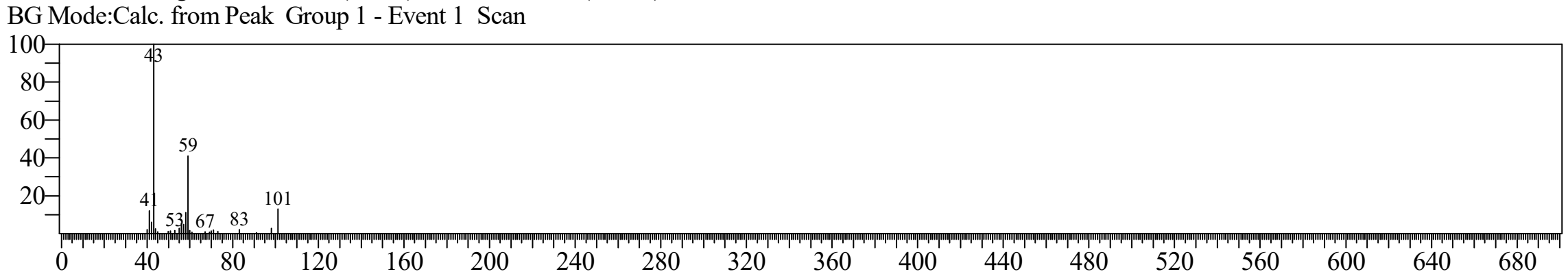

Hit#:1 Entry:14634 Library:WILEY8.LIB
SI:95 Formula:C6H12O2 CAS:123-42-2 MolWeight:116 RetIndex:0
CompName:2-PENTANONE, 4-HYDROXY-4-METHYL- $$ (CH3)2C(OH)CH2C(O)CH3 $$ 2-HYDROXY-2-METHYL-4-PENTANONE $$ 2-HYDRO

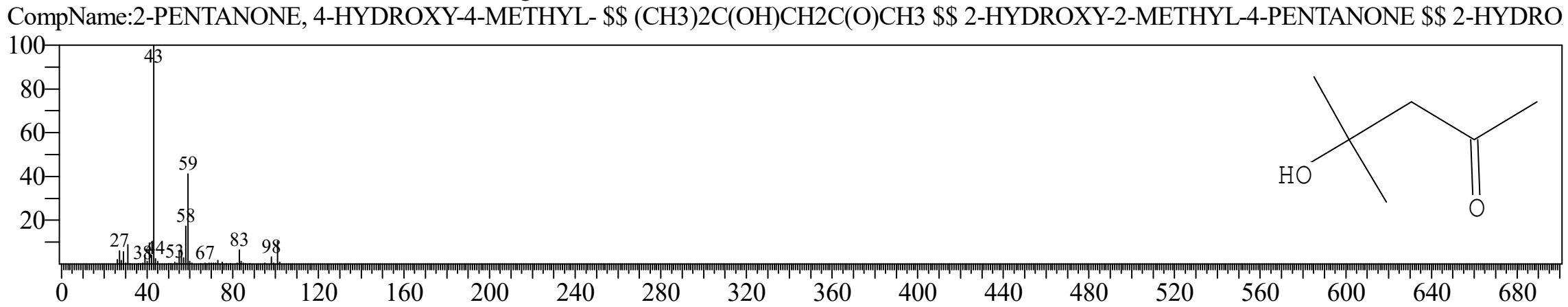

Hit#:2 Entry:14633 Library:WILEY8.LIB
SI:95 Formula:C6H12O2 CAS:123-42-2 MolWeight:116 RetIndex:0
CompName:2-PENTANONE, 4-HYDROXY-4-METHYL- $$ (CH3)2C(OH)CH2C(O)CH3 $$ 2-HYDROXY-2-METHYL-4-PENTANONE $$ 2-HYDRO

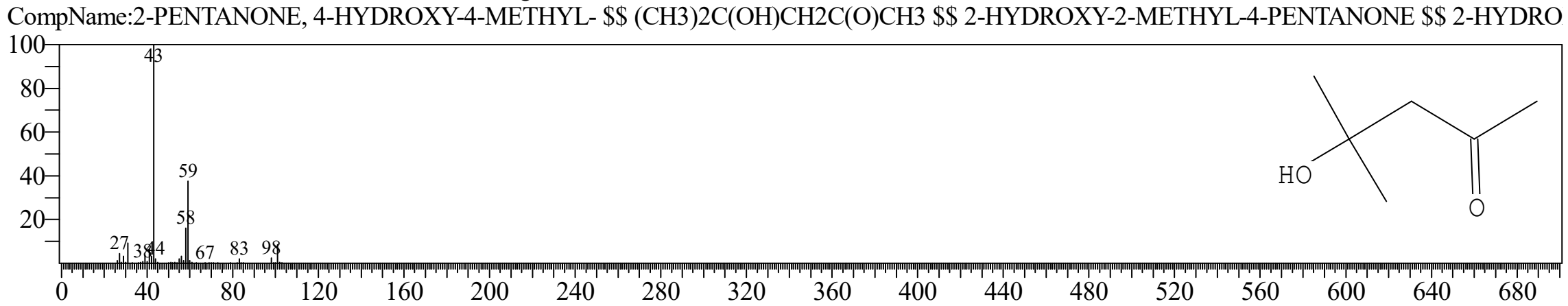

Hit#:3 Entry:14642 Library:WILEY8.LIB
SI:94 Formula:C6H12O2 CAS:123-42-2 MolWeight:116 RetIndex:0
CompName:2-PENTANONE, 4-HYDROXY-4-METHYL- $$ (CH3)2C(OH)CH2C(O)CH3 $$ 2-HYDROXY-2-METHYL-4-PENTANONE $$ 2-HYDRO

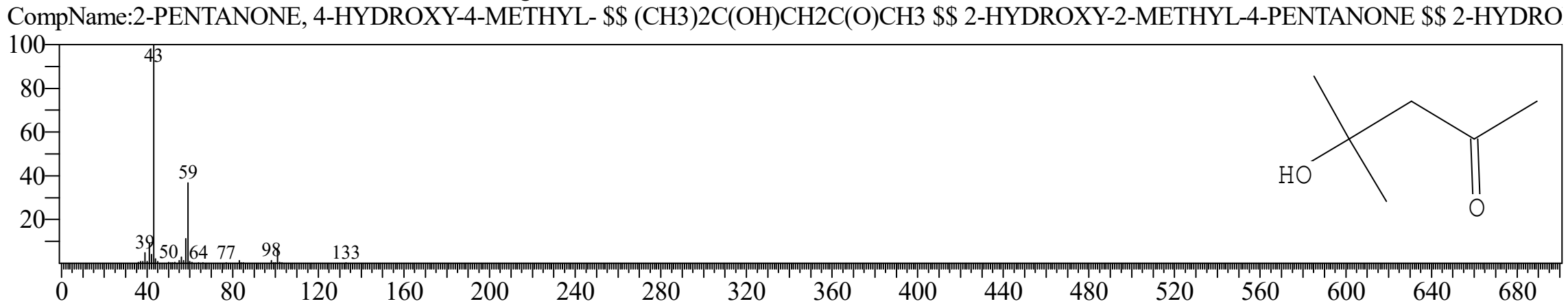

<< Target >>
Line#:2 R.Time:3.830(Scan#:67) MassPeaks:26
RawMode:Averaged 3.825-3.835(66-68) BasePeak:93.05(33728)
BG Mode:Calc. from Peak Group 1 - Event 1 Scan

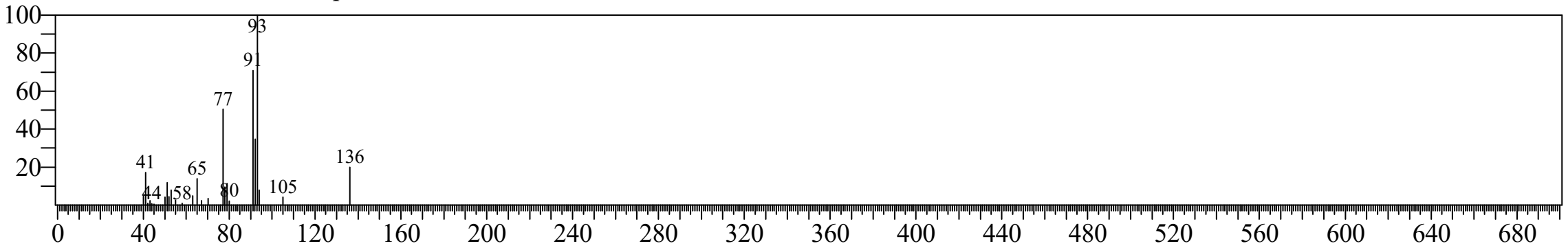

Hit#:1 Entry:6840 Library:NIST14s.lib
SI:93 Formula:C10H16 CAS:99-83-2 MolWeight:136 RetIndex:969
CompName:.alpha.-Phellandrene $$ 1,3-Cyclohexadiene, 2-methyl-5-(1-methylethyl)- $$ .alpha.-Fellandrene $$ p-Mentha-1,5-diene $$ 5-Isopropyl-2-methy

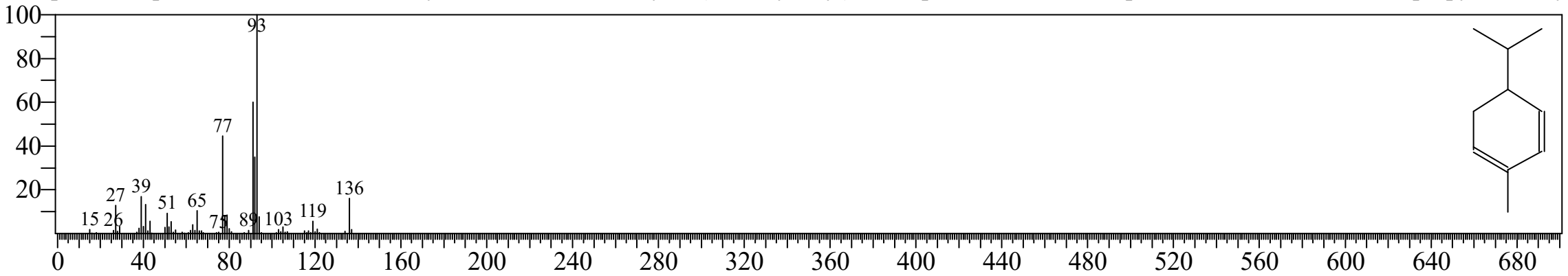

Hit#:2 Entry:28088 Library:WILEY8.LIB
SI:93 Formula:C10H16 CAS:99-83-2 MolWeight:136 RetIndex:0
CompName:1,3-CYCLOHEXADIENE, 2-METHYL-5-(1-METHYLETHYL)- $$ (-)-2-METHYL-5-(1-METHYLETHYL)-1,3-CYCLOHEXADIENE $$ (

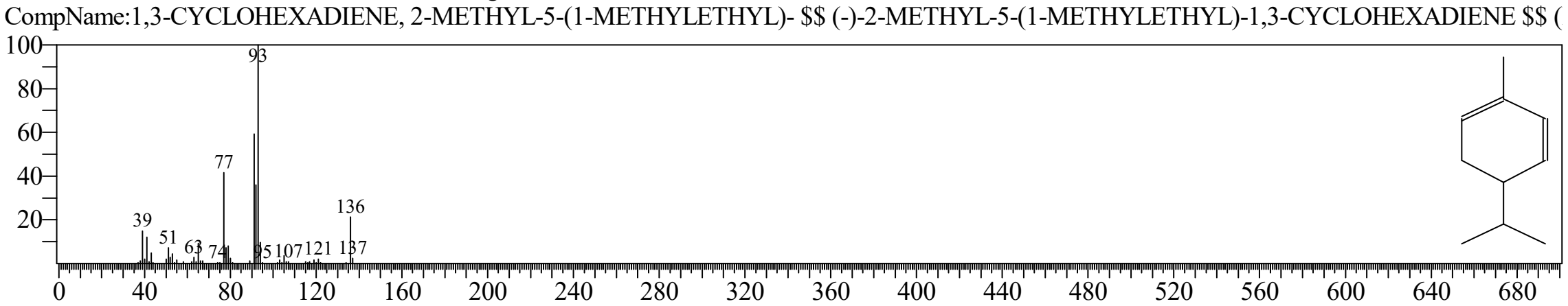

Hit#:3 Entry:28440 Library:WILEY8.LIB
SI:93 Formula:C10H16 CAS:1529-99-3 MolWeight:136 RetIndex:0
CompName:.ALPHA. PHELLANDRENE

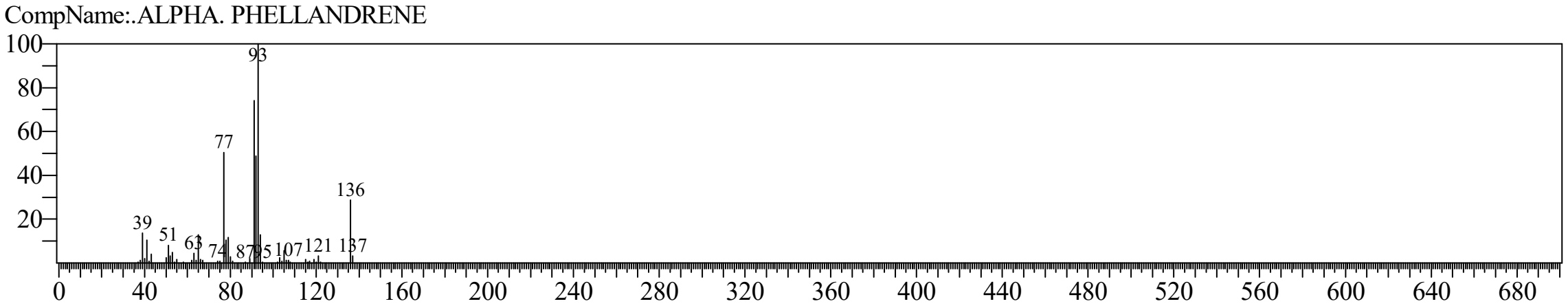

<< Target >>
Line#:3   R.Time:4.050(Scan#:111) MassPeaks:12
RawMode:Averaged 4.045-4.055(110-112) BasePeak:43.00(2521)
BG Mode:Calc. from Peak Group 1 - Event 1 Scan

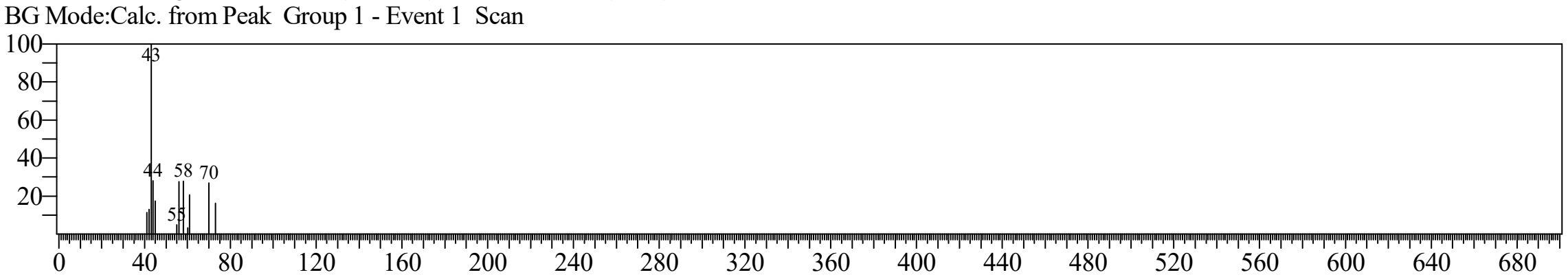

Hit#:1 Entry:4107 Library:WILEY8.LIB
SI:86 Formula:C4H8O2 CAS:141-78-6 MolWeight:88 RetIndex:0
CompName:ACETIC ACID ETHYL ESTER $$ ACETIC ACID, ETHYL ESTER $$ ETHYL ACETATE $$ 1-ACETOXYETHANE $$ ACETATE D'ETHY

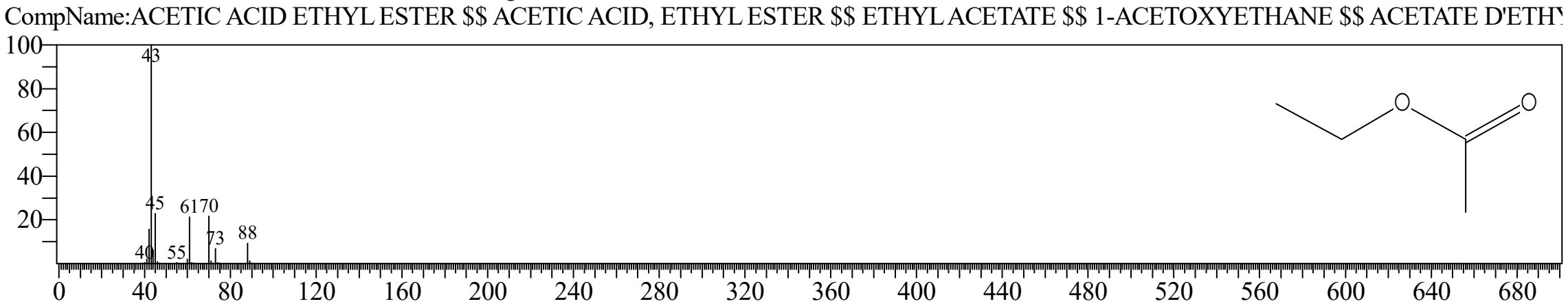

Hit#:2 Entry:23413 Library:WILEY8.LIB
SI:85 Formula:C7H14O2 CAS:628-63-7 MolWeight:130 RetIndex:0
CompName:ACETIC ACID, PENTYL ESTER $$ PENTYL ACETATE $$ 1-ACETOXYPENTANE $$ 1-PENTANOL ACETATE $$ 1-PENTANOL, ACE

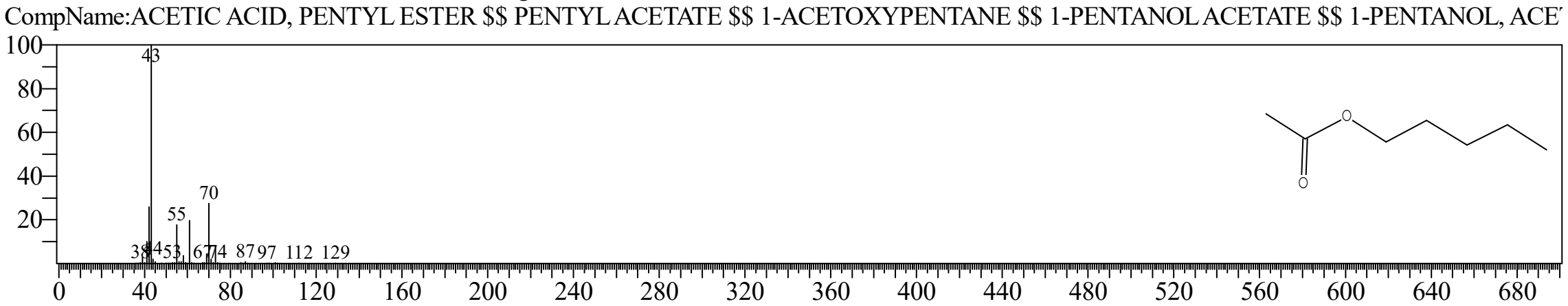

Hit#:3 Entry:14575 Library:WILEY8.LIB
SI:85 Formula:C6H12O2 CAS:123-86-4 MolWeight:116 RetIndex:0
CompName:ACETIC ACID, BUTYL ESTER $$ ACETIC ACID BUTYL ESTER $$ BUTYL ACETATE $$ 1-BUTYL ACETATE $$ ACETATE DE BUTY

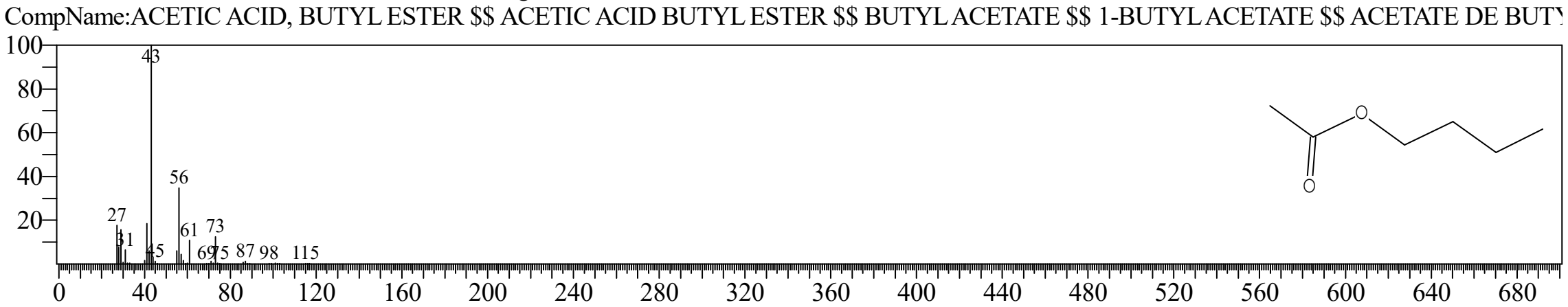

<< Target >>
Line#:4 R.Time:4.200(Scan#:141) MassPeaks:35
RawMode:Averaged 4.195-4.205(140-142) BasePeak:93.10(16888)
BG Mode:Calc. from Peak Group 1 - Event 1 Scan

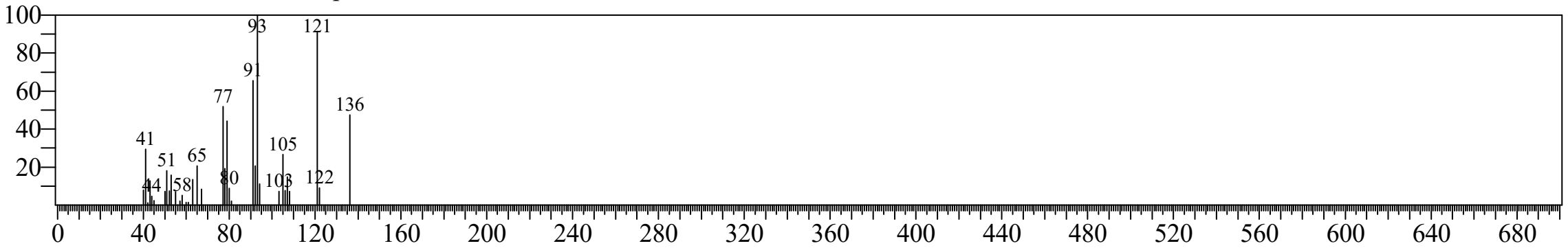

Hit#:1 Entry:28072 Library:WILEY8.LIB
SI:94 Formula:C10H16 CAS:99-86-5 MolWeight:136 RetIndex:0
CompName:1,3-CYCLOHEXADIENE, 1-METHYL-4-(1-METHYLETHYL)- $$ .ALPHA. TERPINENE $$ .ALPHA.-TERPINE $$ .ALPHA.-TERPINEN

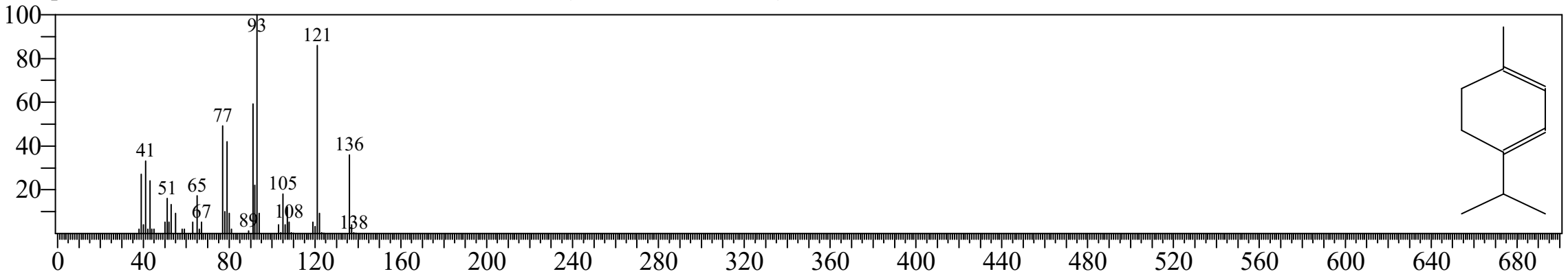

Hit#:2 Entry:28548 Library:WILEY8.LIB
SI:93 Formula:C10H16 CAS:99-86-5 MolWeight:136 RetIndex:0
CompName:1,3-CYCLOHEXADIENE, 1-METHYL-4-(1-METHYLETHYL)- $$ .ALPHA. TERPINENE $$ .ALPHA.-TERPINE $$ .ALPHA.-TERPINEN

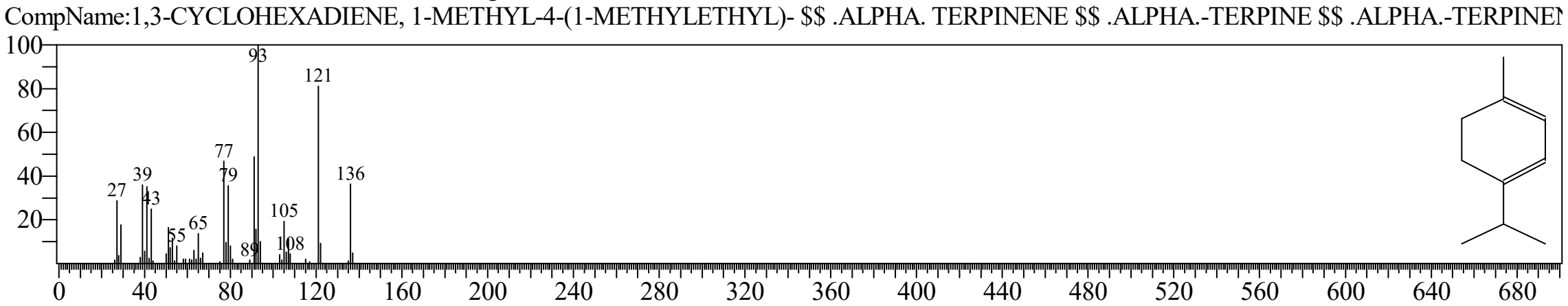

Hit#:3 Entry:10032 Library:NIST14.lib
SI:91 Formula:C10H16 CAS:586-62-9 MolWeight:136 RetIndex:1052
CompName:Cyclohexene, 1-methyl-4-(1-methylethylidene)- $$ p-Mentha-1,4(8)-diene $$ Terpinolene $$ Terpinolen $$ UN 2541 $$ .alpha.- Terpinolen $$

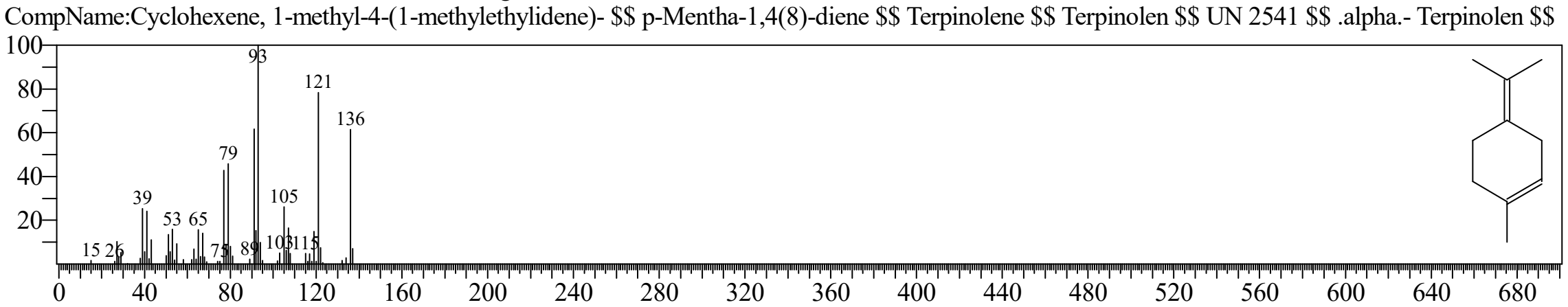

<< Target >>
Line#:5   R.Time:4.425(Scan#:186) MassPeaks:59
RawMode:Averaged 4.420-4.430(185-187) BasePeak:119.10(358752)
BG Mode:Calc. from Peak Group 1 - Event 1 Scan

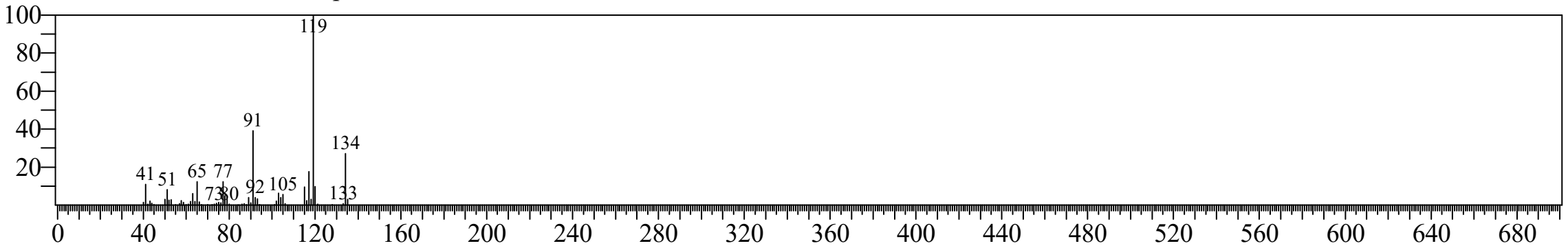

Hit#:1 Entry:9309 Library:NIST14.lib
SI:97 Formula:C10H14 CAS:527-84-4 MolWeight:134 RetIndex:1042
CompName:o-Cymene $$ Benzene, 1-methyl-2-(1-methylethyl)- $$ o-Cymol $$ o-Isopropyltoluene $$ 1-Isopropyl-2-methylbenzene $$ 1-Methyl-2-isoprop

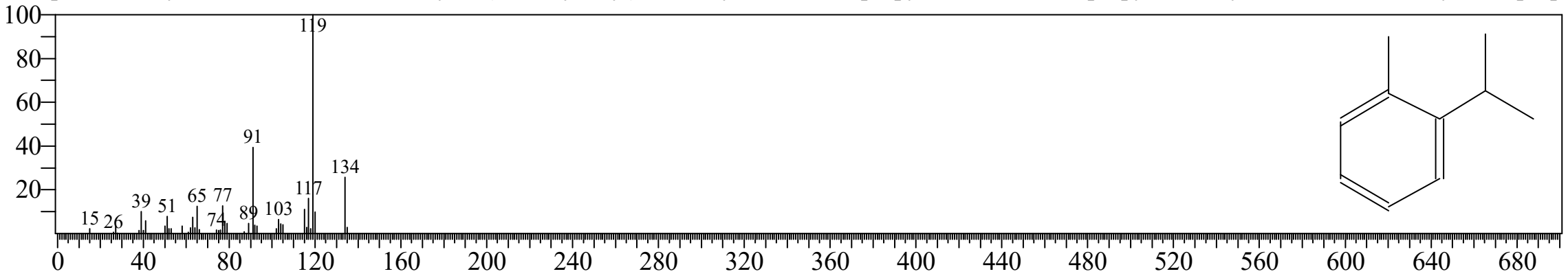

Hit#:2 Entry:6363 Library:NIST14s.lib
SI:96 Formula:C10H14 CAS:535-77-3 MolWeight:134 RetIndex:1042
CompName:Benzene, 1-methyl-3-(1-methylethyl)- $$ m-Cymene $$ .beta.-Cymene $$ m-Cymol $$ m-Isopropyltoluene $$ m-Methylisopropylbenzene $$ 1-

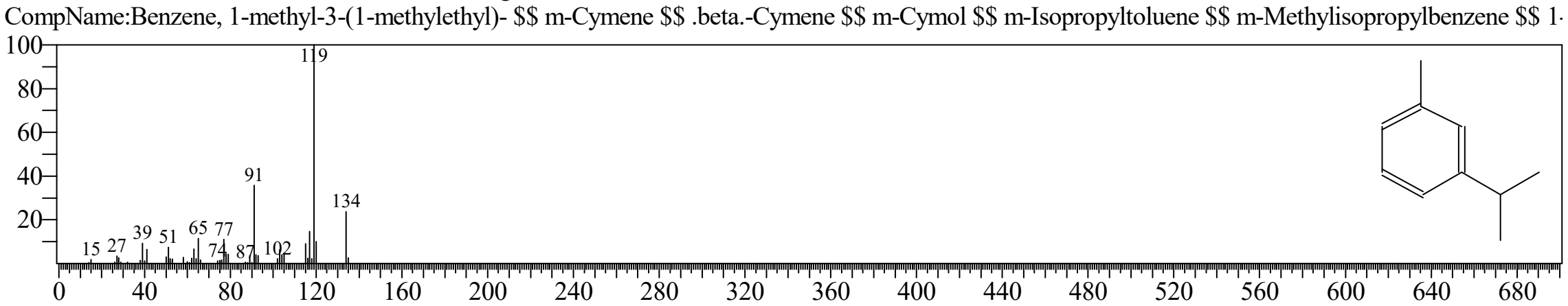

Hit#:3 Entry:6359 Library:NIST14s.lib
SI:96 Formula:C10H14 CAS:99-87-6 MolWeight:134 RetIndex:1042
CompName:p-Cymene $$ Benzene, 1-methyl-4-(1-methylethyl)- $$ p-Cimene $$ p-Cymol $$ p-Isopropyltoluene $$ p-Methylisopropylbenzene $$ Campho

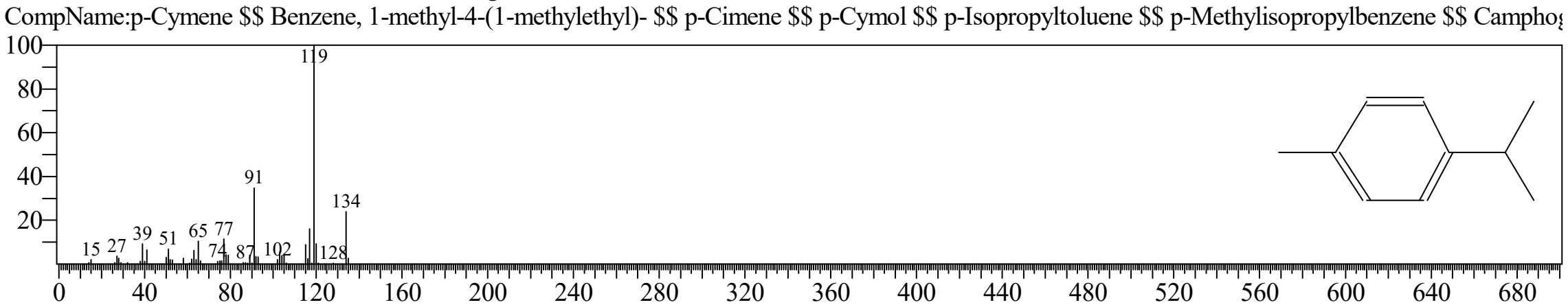

<< Target >>
Line#:6   R.Time:4.545(Scan#:210) MassPeaks:38
RawMode:Averaged 4.540-4.550(209-211) BasePeak:93.05(18017)
BG Mode:Calc. from Peak Group 1 - Event 1 Scan

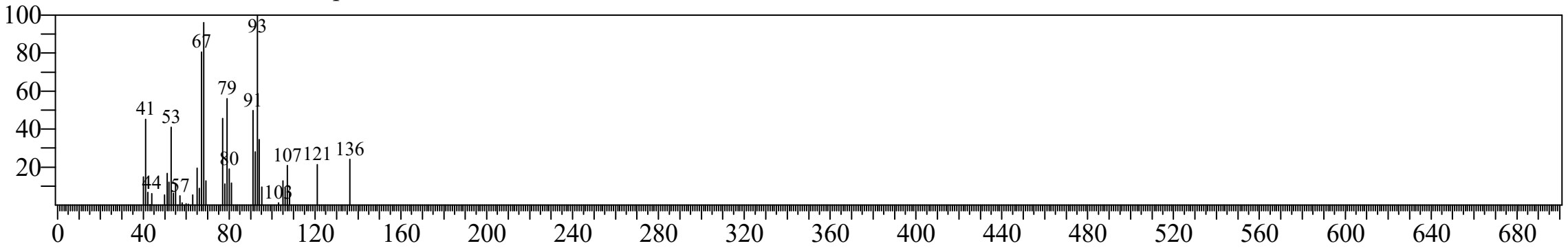

Hit#:1 Entry:28150 Library:WILEY8.LIB
SI:92 Formula:C10H16 CAS:138-86-3 MolWeight:136 RetIndex:0
CompName:CYCLOHEXENE, 1-METHYL-4-(1-METHYLETHENYL)- $$ 1-METHYL-4-(1-METHYLETHENYL)CYCLOHEXENE $$ (+-)-ALPHA-L

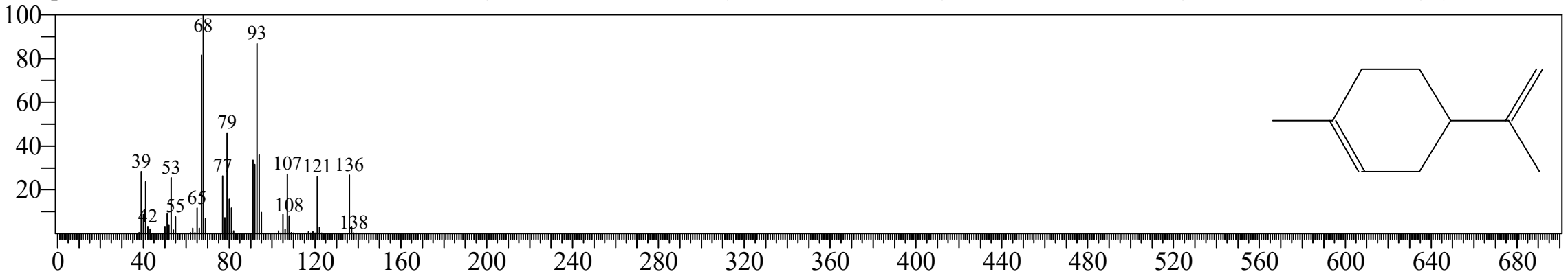

Hit#:2 Entry:28608 Library:WILEY8.LIB
SI:92 Formula:C10H16 CAS:138-86-3 MolWeight:136 RetIndex:0
CompName:CYCLOHEXENE, 1-METHYL-4-(1-METHYLETHENYL)- $$ 1-METHYL-4-(1-METHYLETHENYL)CYCLOHEXENE $$ (+-)-ALPHA-L

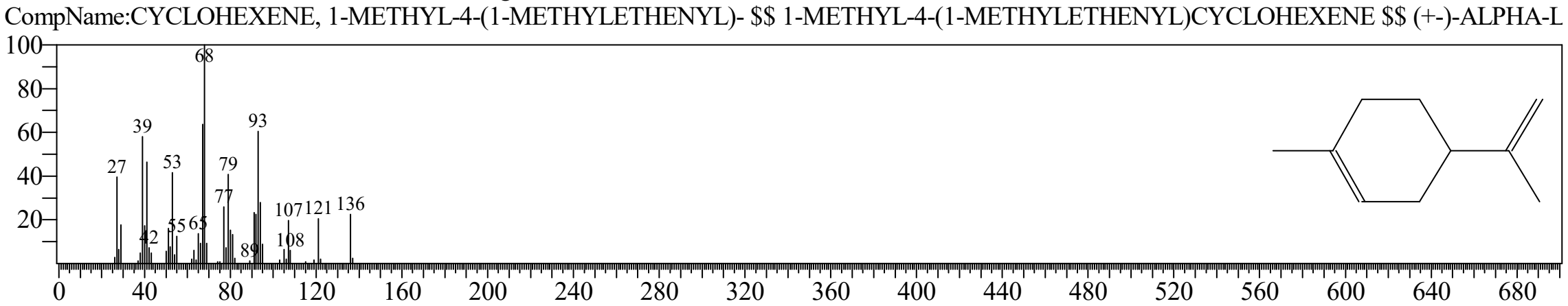

Hit#:3 Entry:28144 Library:WILEY8.LIB
SI:92 Formula:C10H16 CAS:138-86-3 MolWeight:136 RetIndex:0
CompName:CYCLOHEXENE, 1-METHYL-4-(1-METHYLETHENYL)- $$ 1-METHYL-4-(1-METHYLETHENYL)CYCLOHEXENE $$ (+-)-ALPHA-L

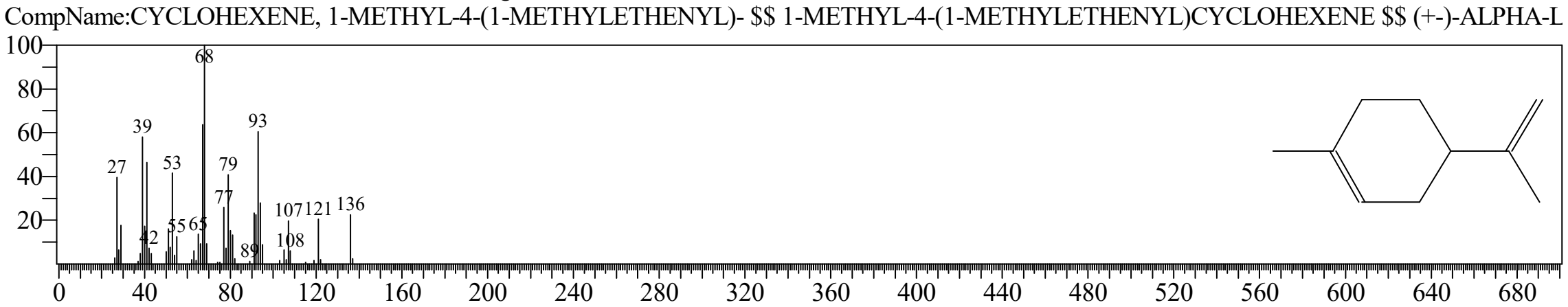

<< Target >>
Line#:7 R.Time:4.825(Scan#:266) MassPeaks:41
RawMode:Averaged 4.820-4.830(265-267) BasePeak:93.05(71508)
BG Mode:Calc. from Peak Group 1 - Event 1 Scan

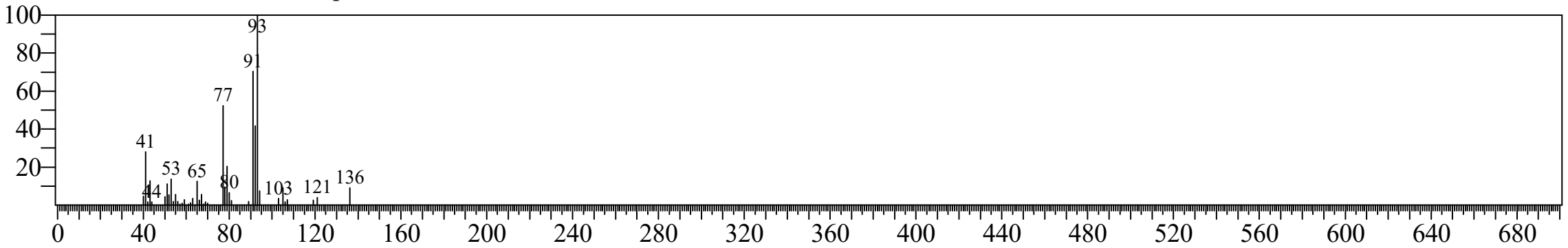

Hit#:1 Entry:10017 Library:NIST14.lib
SI:93 Formula:C10H16 CAS:80-56-8 MolWeight:136 RetIndex:948
CompName:.alpha.-Pinene $$ Bicyclo[3.1.1]hept-2-ene, 2,6,6-trimethyl- $$ 2-Pinene $$ 2,6,6-Trimethylbicyclo[3.1.1]hept-2-ene $$ Pinene, .alpha. $$ 2,6,6

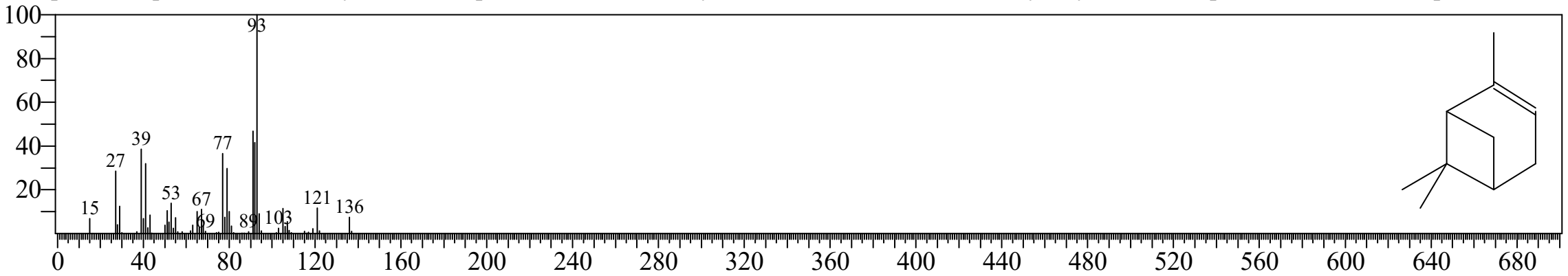

Hit#:2 Entry:6845 Library:NIST14s.lib
SI:92 Formula:C10H16 CAS:7785-70-8 MolWeight:136 RetIndex:948
CompName:(1R)-2,6,6-Trimethylbicyclo[3.1.1]hept-2-ene $$ 1R-.alpha.-Pinene $$ Bicyclo[3.1.1]hept-2-ene, 2,6,6-trimethyl-, (1R)- $$ 2,6,6-Trimethylbicy

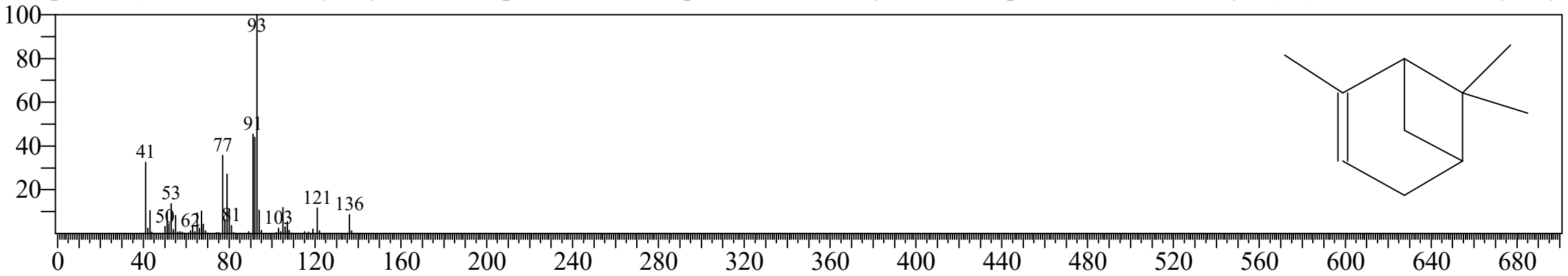

Hit#:3 Entry:28312 Library:WILEY8.LIB
SI:92 Formula:C10H16 CAS:80-56-8 MolWeight:136 RetIndex:0
CompName:BICYCLO[3.1.1]HEPT-2-ENE, 2,6,6-TRIMETHYL- $$,6,6-TRIMETHYL-BICYCLO[3.1.1]HEPT-2-ENE $$ -PINENE $$ .ALPHA.-(+)-PI

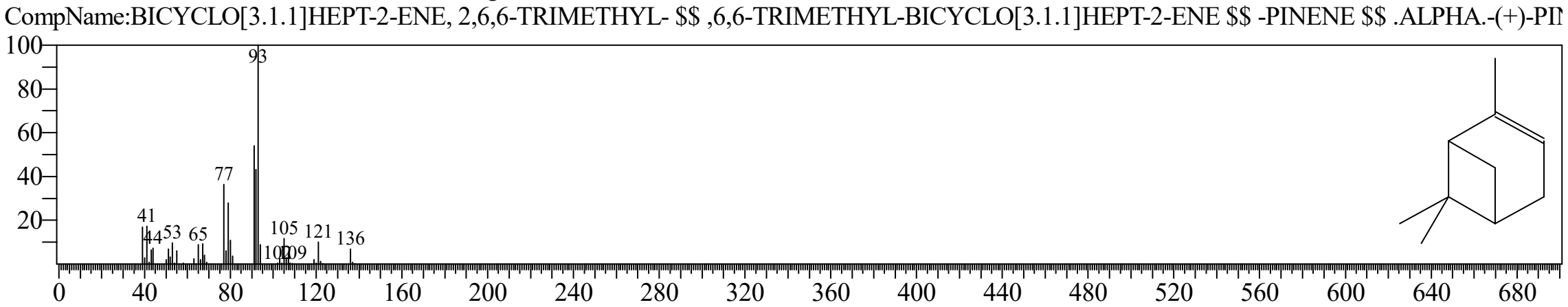

<< Target >>
Line#:8   R.Time:4.935(Scan#:288) MassPeaks:59
RawMode:Averaged 4.930-4.940(287-289) BasePeak:93.10(379475)
BG Mode:Calc. from Peak Group 1 - Event 1 Scan

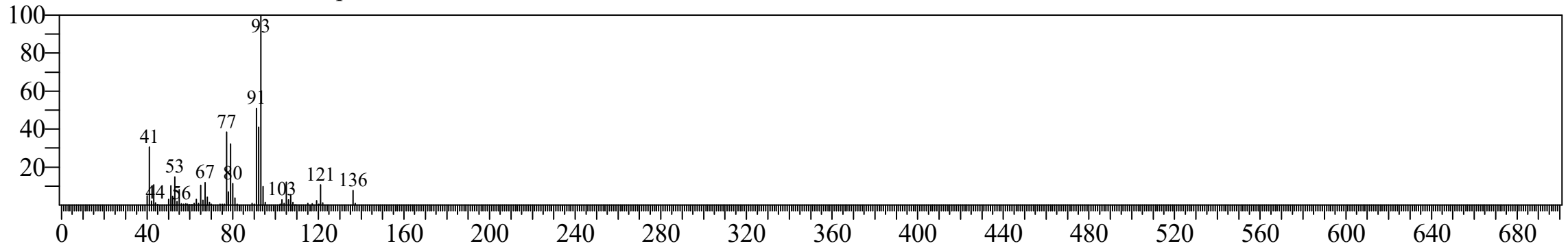

Hit#:1 Entry:10017 Library:NIST14.lib
SI:98 Formula:C10H16 CAS:80-56-8 MolWeight:136 RetIndex:948
CompName:.alpha.-Pinene $$ Bicyclo[3.1.1]hept-2-ene, 2,6,6-trimethyl- $$ 2-Pinene $$ 2,6,6-Trimethylbicyclo[3.1.1]hept-2-ene $$ Pinene, .alpha. $$ 2,6,6

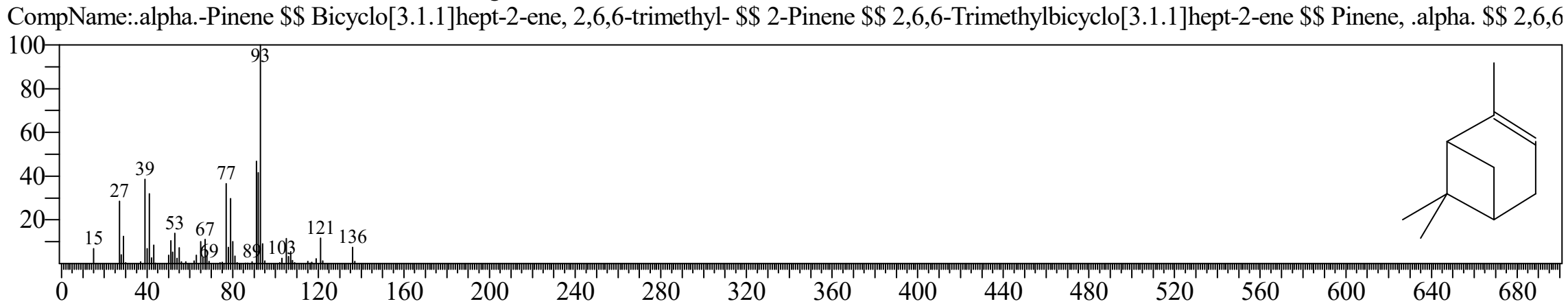

Hit#:2 Entry:6845 Library:NIST14s.lib
SI:98 Formula:C10H16 CAS:7785-70-8 MolWeight:136 RetIndex:948
CompName:(1R)-2,6,6-Trimethylbicyclo[3.1.1]hept-2-ene $$ 1R-.alpha.-Pinene $$ Bicyclo[3.1.1]hept-2-ene, 2,6,6-trimethyl-, (1R)- $$ 2,6,6-Trimethylbicy

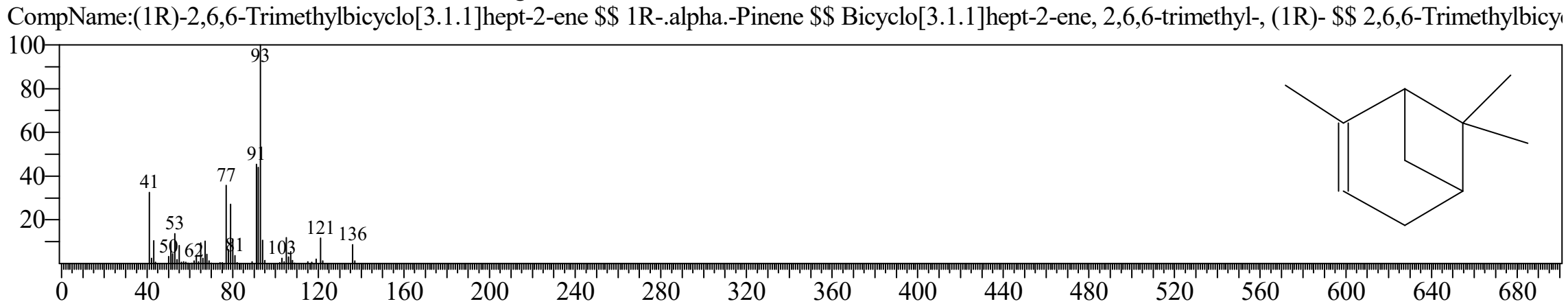

Hit#:3 Entry:6844 Library:NIST14s.lib
SI:96 Formula:C10H16 CAS:3779-61-1 MolWeight:136 RetIndex:976
CompName:trans-.beta.-Ocimene $$ 1,3,6-Octatriene, 3,7-dimethyl-, (E)- $$ .beta.-trans-Ocimene $$ trans-3,7-Dimethyl-1,3,6-Octatriene $$ Ocimene, trans

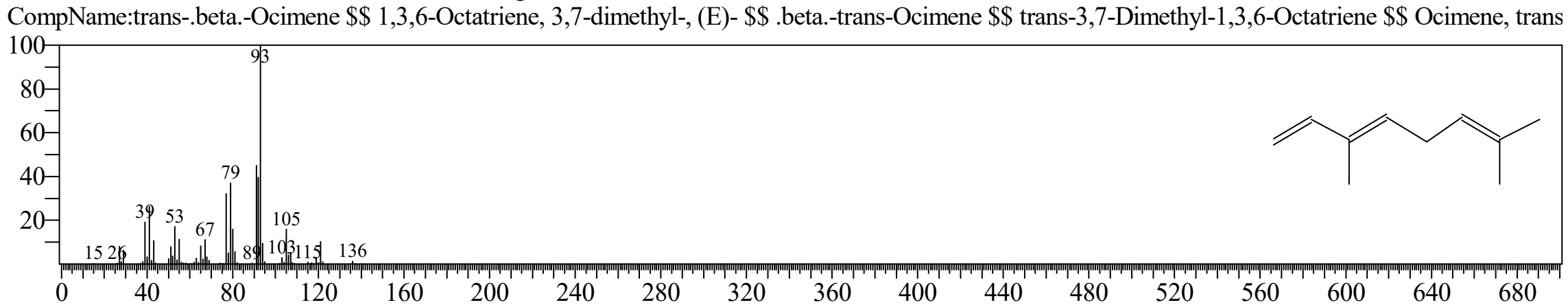

<< Target >>
Line#:9 R.Time:5.125(Scan#:326) MassPeaks:21
RawMode:Averaged 5.120-5.130(325-327) BasePeak:93.00(2121)
BG Mode:Calc. from Peak Group 1 - Event 1 Scan

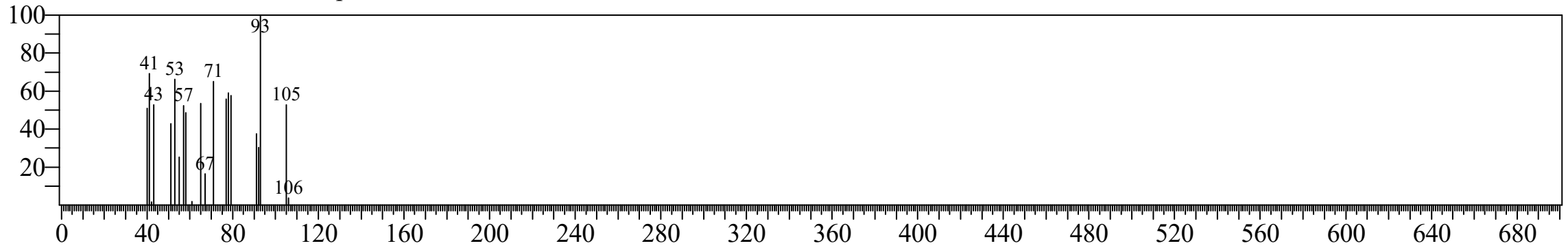

Hit#:1 Entry:10242 Library:WILEY8.LIB
SI:74 Formula:C8H12 CAS:61142-26-5 MolWeight:108 RetIndex:0
CompName:CYCLOPROPENE, 1-METHYL-3-(2-METHYLCYCLOPROPYL)- $$ 1-METHYL-3-(2-METHYLCYCLOPROPYL)-1-CYCLOPROPENE

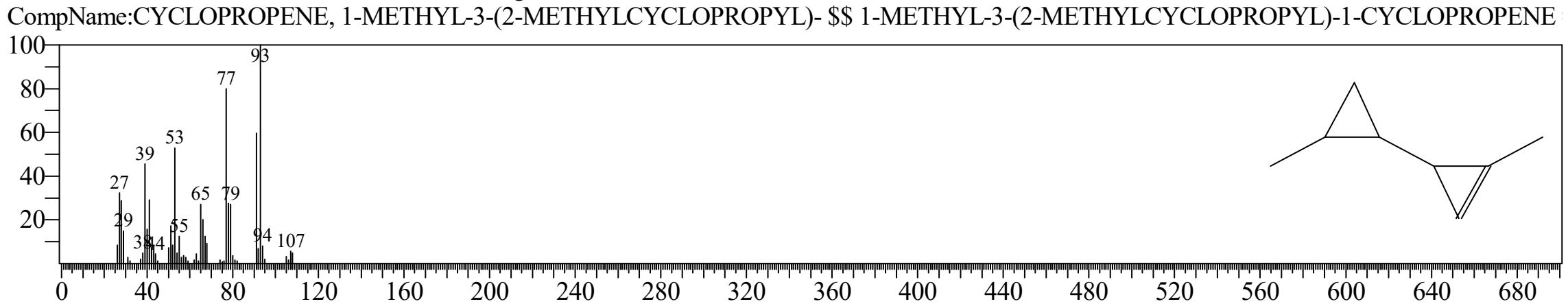

Hit#:2 Entry:10372 Library:WILEY8.LIB
SI:74 Formula:C8H12 CAS:61142-26-5 MolWeight:108 RetIndex:0
CompName:CYCLOPROPENE, 1-METHYL-3-(2-METHYLCYCLOPROPYL)- $$ 1-METHYL-3-(2-METHYLCYCLOPROPYL)-1-CYCLOPROPENE

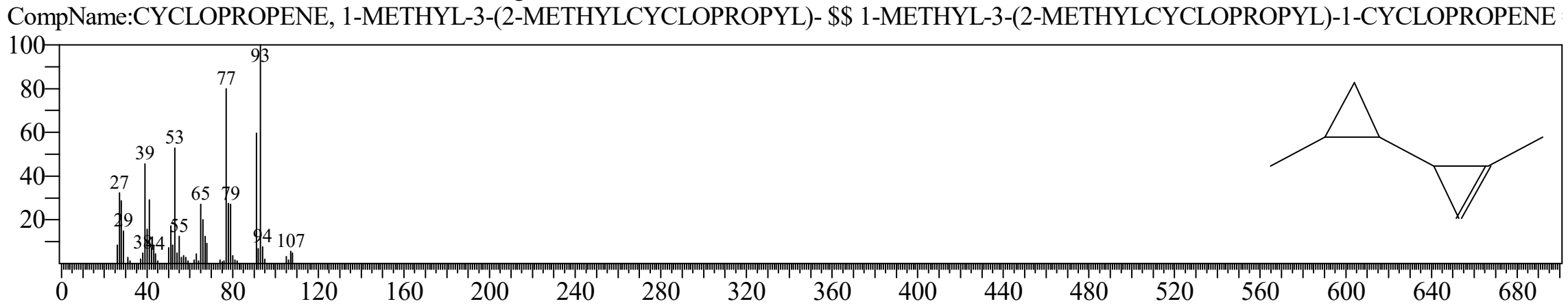

Hit#:3 Entry:2953 Library:NIST14.lib
SI:74 Formula:C8H12 CAS:61142-26-5 MolWeight:108 RetIndex:754
CompName:Cyclopropene, 1-methyl-3-(2-methylcyclopropyl)- $$ 1-Methyl-3-(2-methylcyclopropyl)-1-cyclopropene # $$

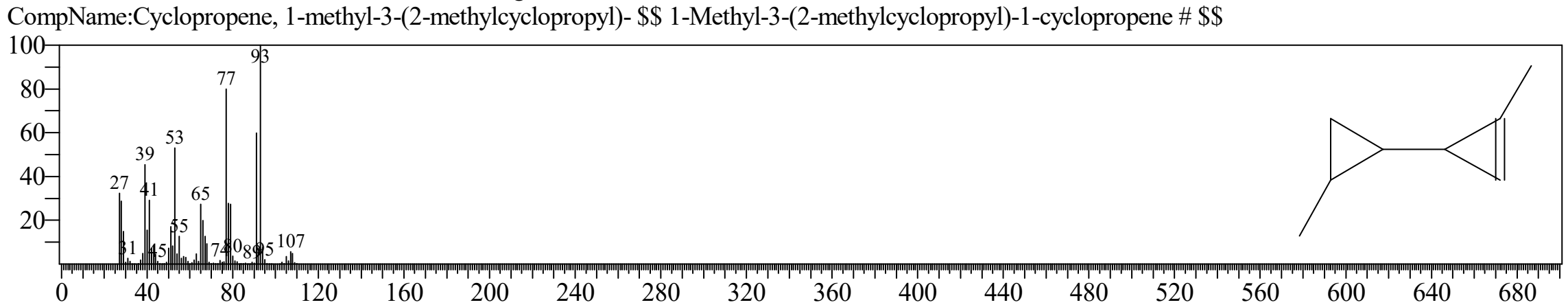

<< Target >>
Line#:10   R.Time:5.270(Scan#:355) MassPeaks:14
RawMode:Averaged 5.265-5.275(354-356) BasePeak:43.00(788)
BG Mode:Calc. from Peak Group 1 - Event 1 Scan

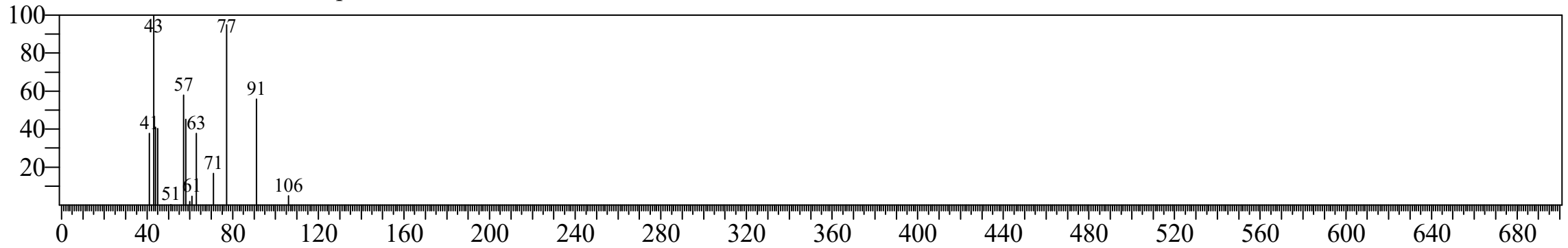

Hit#:1 Entry:3415 Library:WILEY8.LIB
SI:73 Formula:C4H6O2 CAS:638-37-9 MolWeight:86 RetIndex:0
CompName:BUTANEDIAL $$ SUCCINALDEHYDE $$ .BETA.-FORMYLPROPIONALDEHYDE $$ 1,4-BUTANEDIAL $$ 1,4-BUTANEDIONE $$ B

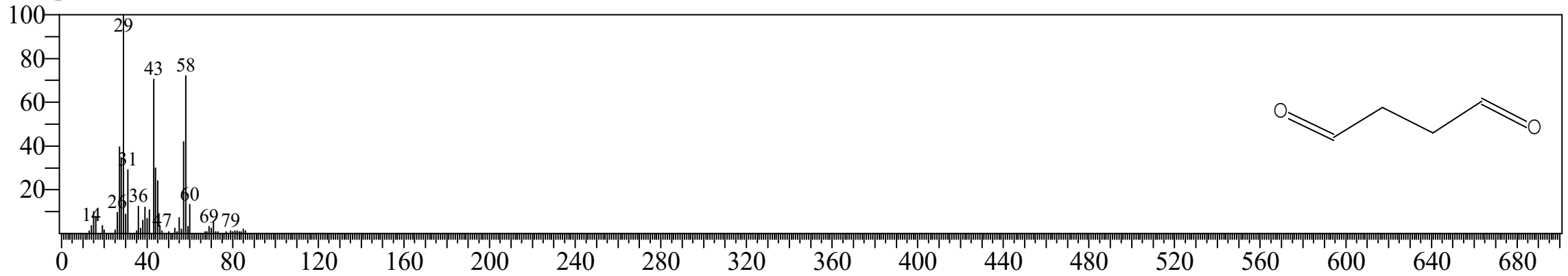

Hit#:2 Entry:839 Library:NIST14s.lib
SI:73 Formula:C4H6O2 CAS:638-37-9 MolWeight:86 RetIndex:796
CompName:Succindialdehyde $$ Butanedial $$ Succinaldehyde $$ Succinic aldehyde $$ Succinic dialdehyde $$ 1,4-Butanedione $$ .beta.-Formylpropiona

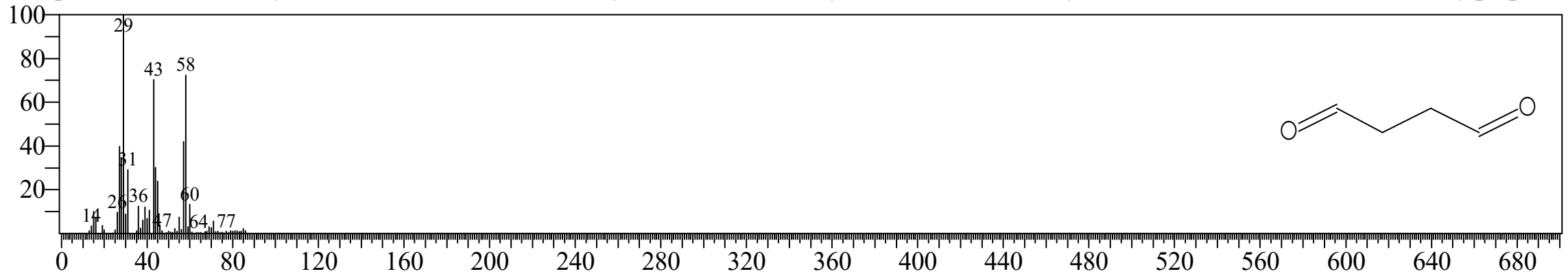

Hit#:3 Entry:3417 Library:WILEY8.LIB
SI:71 Formula:C4H6O2 CAS:638-37-9 MolWeight:86 RetIndex:0
CompName:BUTANEDIAL $$ SUCCINALDEHYDE $$ .BETA.-FORMYLPROPIONALDEHYDE $$ 1,4-BUTANEDIAL $$ 1,4-BUTANEDIONE $$ B

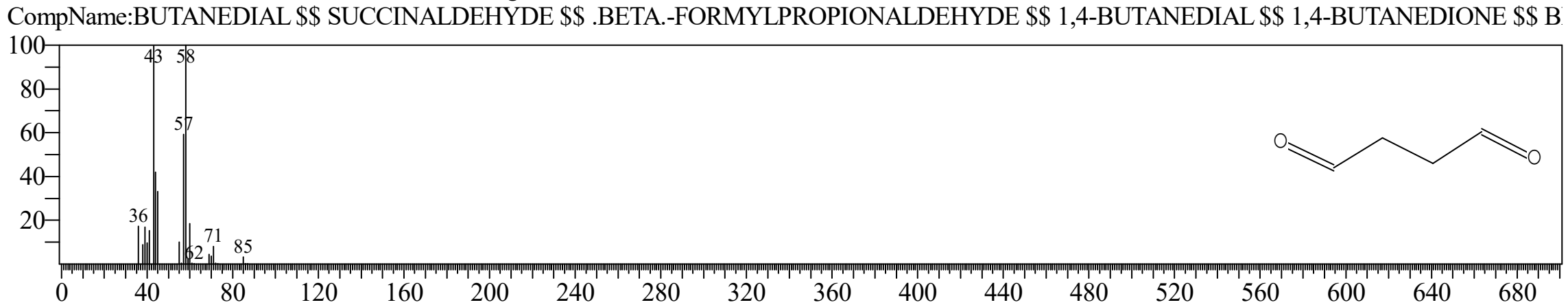

<< Target >>
Line#:11 R.Time:5.355(Scan#:372) MassPeaks:75
RawMode:Averaged 5.350-5.360(371-373) BasePeak:93.05(1435527)
BG Mode:Calc. from Peak Group 1 - Event 1 Scan

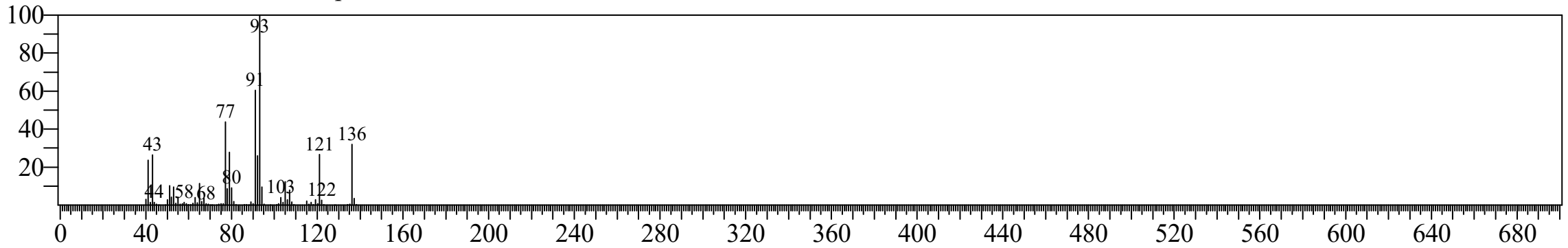

Hit#:1 Entry:28552 Library:WILEY8.LIB
SI:97 Formula:C10H16 CAS:99-85-4 MolWeight:136 RetIndex:0
CompName:1,4-CYCLOHEXADIENE, 1-METHYL-4-(1-METHYLETHYL)- $$ 1-ISOPROPYL-4-METHYL-1,4-CYCLOHEXADIENE # $$ .GAMMA.

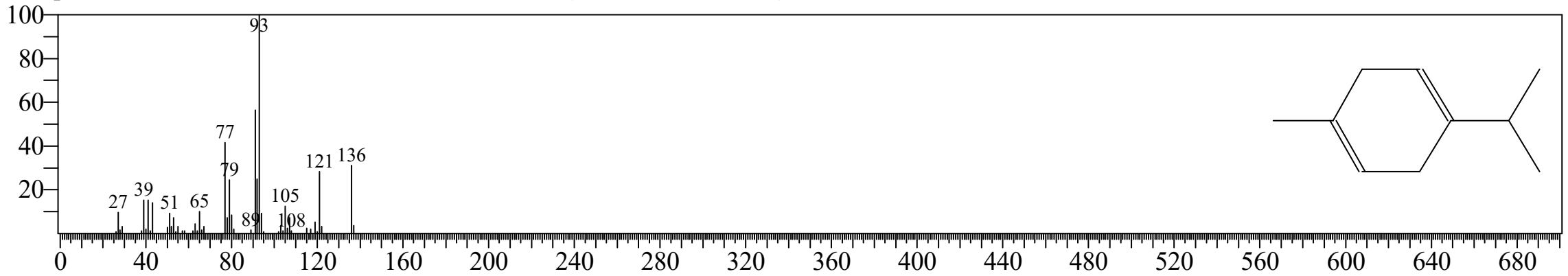

Hit#:2 Entry:6836 Library:NIST14s.lib
SI:97 Formula:C10H16 CAS:99-85-4 MolWeight:136 RetIndex:998
CompName:.gamma.-Terpinene $$ 1,4-Cyclohexadiene, 1-methyl-4-(1-methylethyl)- $$ .gamma.-Terpinen $$ p-Mentha-1,4-diene $$ Crithmene $$ Moslene

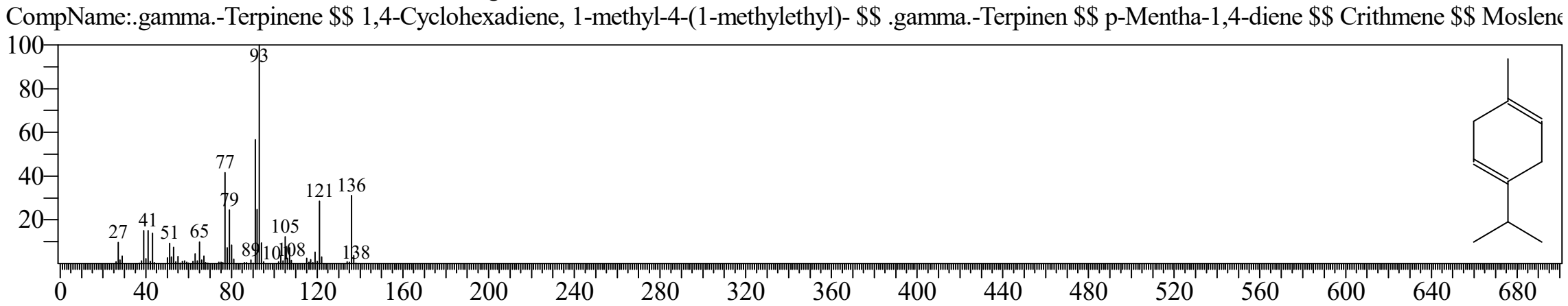

Hit#:3 Entry:28119 Library:WILEY8.LIB
SI:96 Formula:C10H16 CAS:99-85-4 MolWeight:136 RetIndex:0
CompName:1,4-CYCLOHEXADIENE, 1-METHYL-4-(1-METHYLETHYL)- $$ 1-ISOPROPYL-4-METHYL-1,4-CYCLOHEXADIENE # $$ .GAMMA.

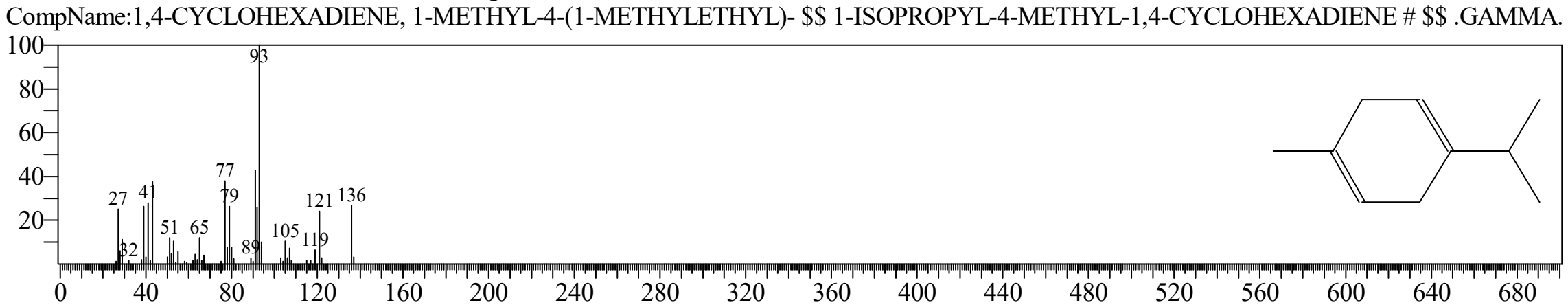

<< Target >>
Line#:12 R.Time:5.520(Scan#:405) MassPeaks:21
RawMode:Averaged 5.515-5.525(404-406) BasePeak:71.10(1287)
BG Mode:Calc. from Peak Group 1 - Event 1 Scan

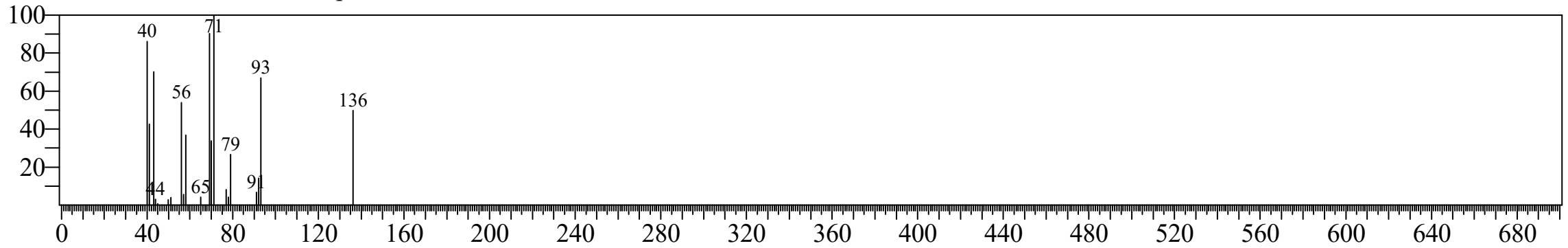

Hit#:1 Entry:6721 Library:NIST14.lib
SI:73 Formula:C8H14O CAS:17123-63-6 MolWeight:126 RetIndex:913
CompName:2,5-Dimethyl-1,5-hexadien-3-ol

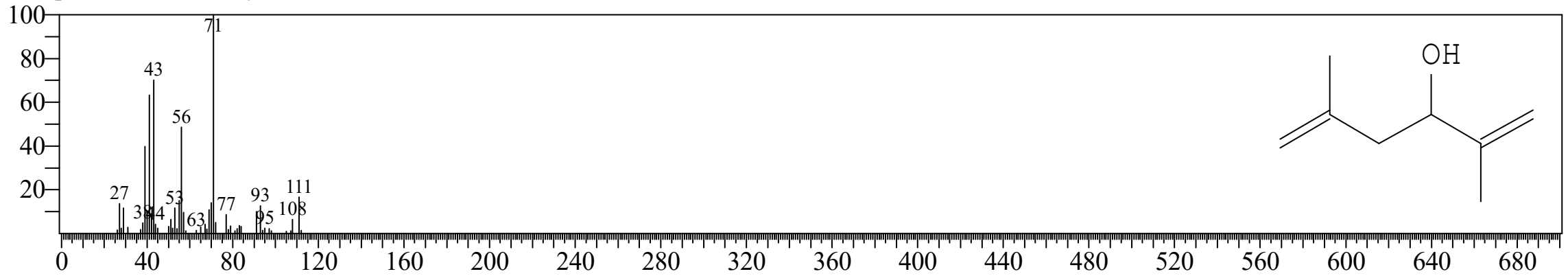

Hit#:2 Entry:18063 Library:NIST14.lib
SI:72 Formula:C10H18O CAS:52939-61-4 MolWeight:154 RetIndex:1087
CompName:1,6-Heptadien-4-ol, 4-propyl- $$ 4-Propyl-1,6-heptadien-4-ol $$

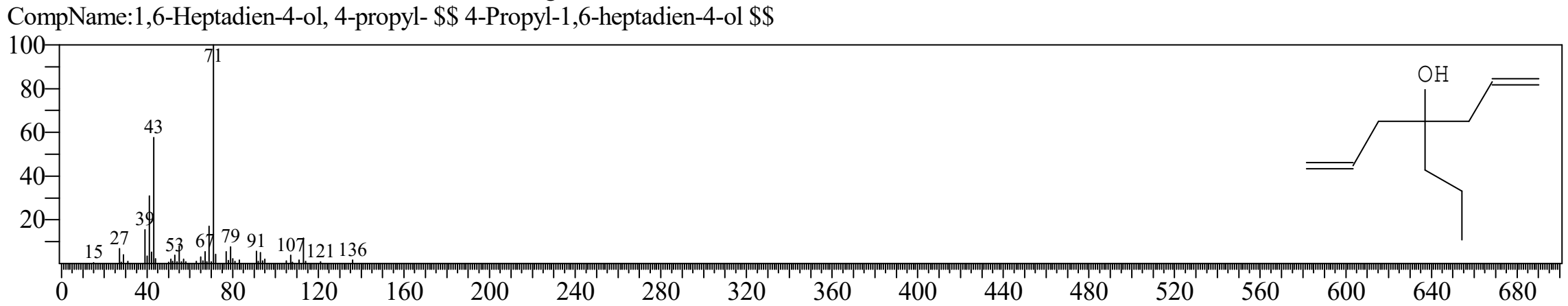

Hit#:3 Entry:46572 Library:WILEY8.LIB
SI:71 Formula:C10H18O CAS:78-70-6 MolWeight:154 RetIndex:0
CompName:1,6-OCTADIEN-3-OL, 3,7-DIMETHYL- $$ 3,7-DIMETHYLOCTA-1,6-DIEN-3-OL $$ (+-)-LINALOOL $$ (1)-3,7-DIMETHYL-1,6-OCTAD

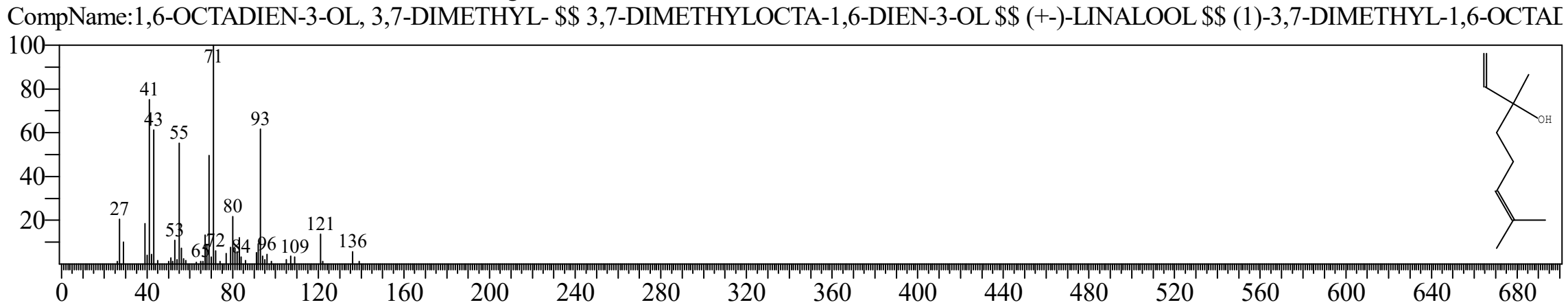

<< Target >>
Line#:13   R.Time:5.585(Scan#:418) MassPeaks:56
RawMode:Averaged 5.580-5.590(417-419) BasePeak:93.10(236594)
BG Mode:Calc. from Peak Group 1 - Event 1 Scan

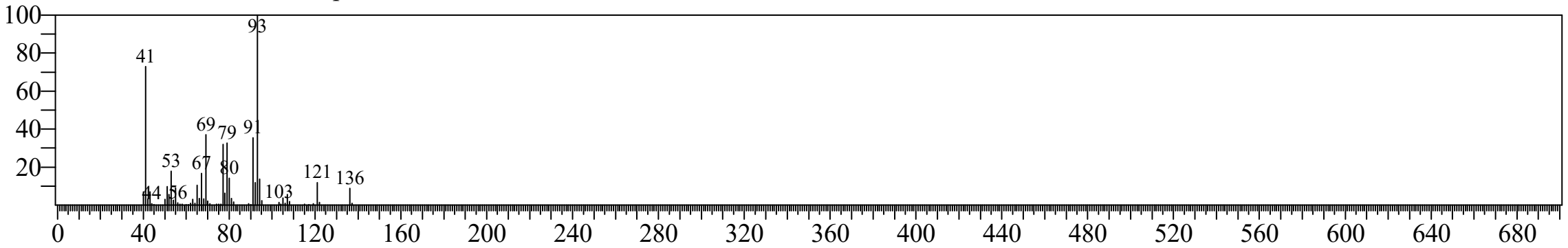

Hit#:1 Entry:9984 Library:NIST14.lib
SI:97 Formula:C10H16 CAS:127-91-3 MolWeight:136 RetIndex:943
CompName:.beta.-Pinene $$ Bicyclo[3.1.1]heptane, 6,6-dimethyl-2-methylene- $$ 2(10)-Pinene $$ Nopinen $$ Nopinene $$ Pseudopinen $$ Pseudopinene

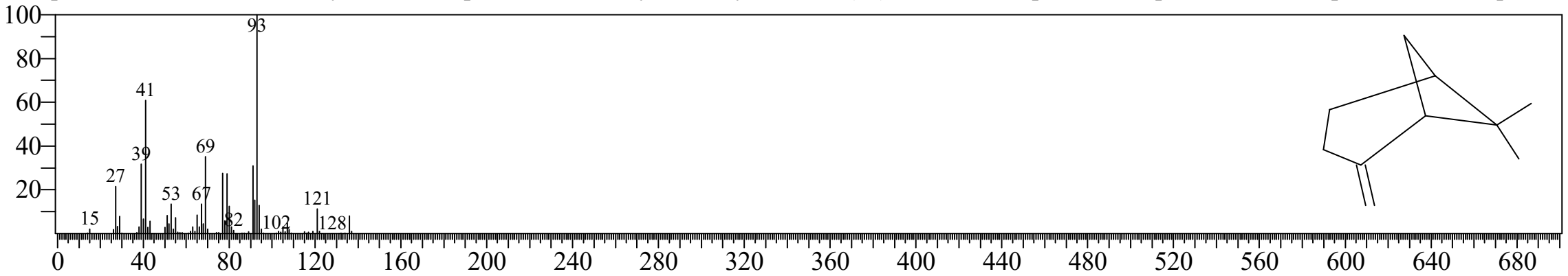

Hit#:2 Entry:28596 Library:WILEY8.LIB
SI:96 Formula:C10H16 CAS:0-00-0 MolWeight:136 RetIndex:0
CompName:(+)-BETA-PINEN $$ 6,6-DIMETHYL-2-METHYLENEBICYCLO[3.1.1]HEPTANE

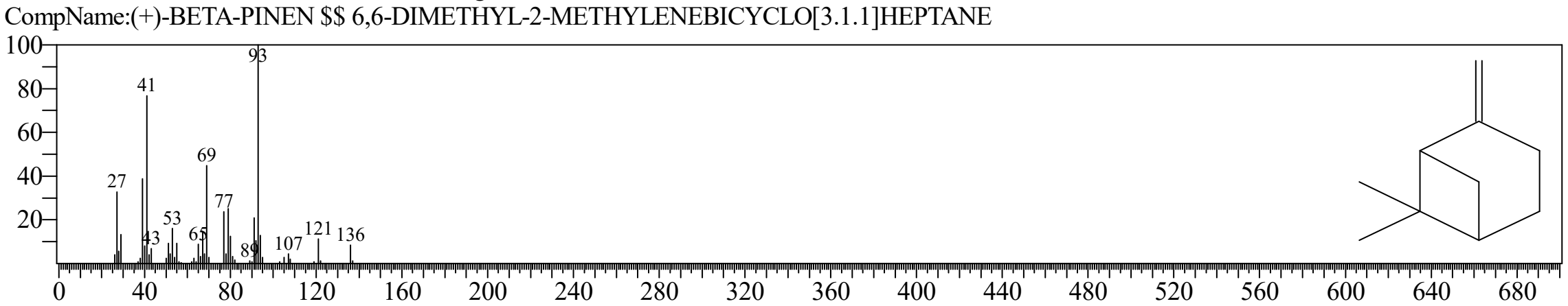

Hit#:3 Entry:28328 Library:WILEY8.LIB
SI:96 Formula:C10H16 CAS:127-91-3 MolWeight:136 RetIndex:0
CompName:BICYCLO[3.1.1]HEPTANE, 6,6-DIMETHYL-2-METHYLENE- $$ (+)-BETA-PINEN $$ (1)-6,6-DIMETHYL-2-METHYLENEBICYCLO(3

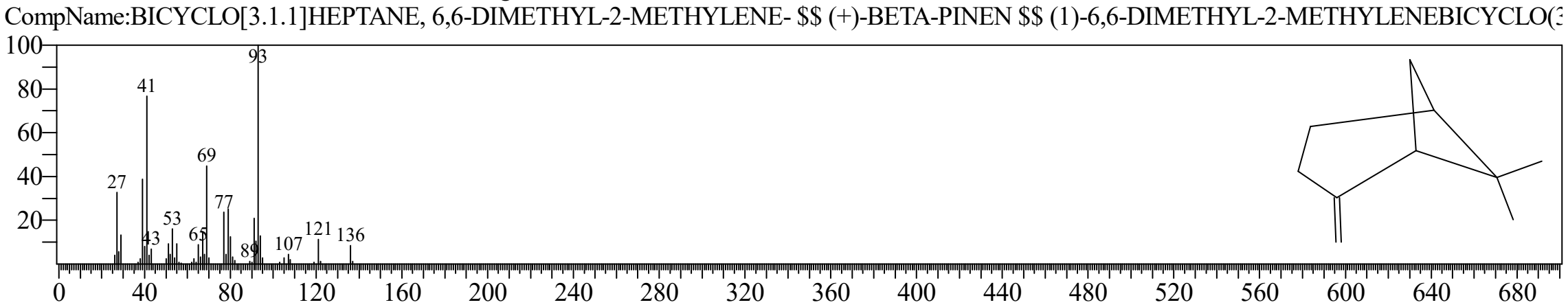

<< Target >>
Line#:14   R.Time:5.755(Scan#:452) MassPeaks:34
RawMode:Averaged 5.750-5.760(451-453) BasePeak:41.05(28349)
BG Mode:Calc. from Peak Group 1 - Event 1 Scan

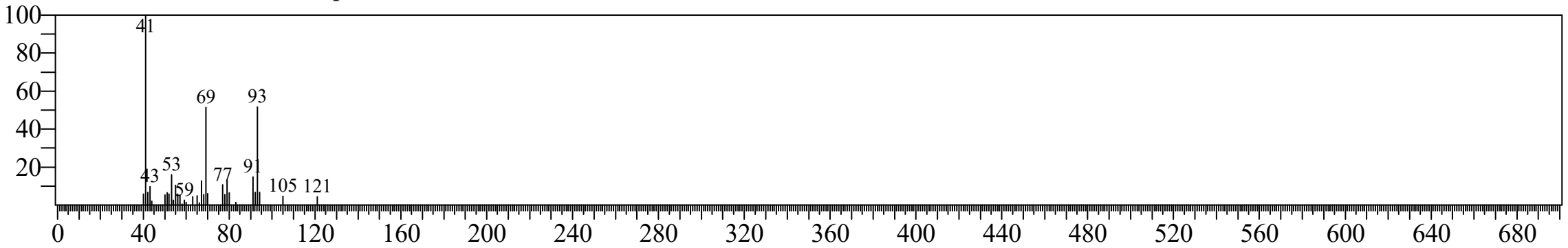

Hit#:1 Entry:28027 Library:WILEY8.LIB
SI:91 Formula:C10H16 CAS:123-35-3 MolWeight:136 RetIndex:0
CompName:1,6-OCTADIENE, 7-METHYL-3-METHYLENE- $$ 7-METHYL-3-METHYLENE-OCTA-1,6-DIENE $$ .BETA.-MYRCENE $$ 1, 6-OCTA

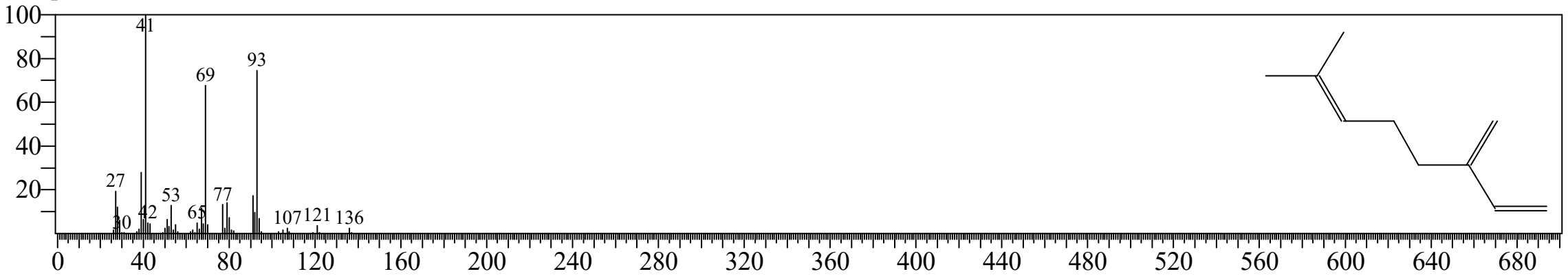

Hit#:2 Entry:28026 Library:WILEY8.LIB
SI:91 Formula:C10H16 CAS:123-35-3 MolWeight:136 RetIndex:0
CompName:1,6-OCTADIENE, 7-METHYL-3-METHYLENE- $$ 7-METHYL-3-METHYLENE-OCTA-1,6-DIENE $$ .BETA.-MYRCENE $$ 1, 6-OCTA

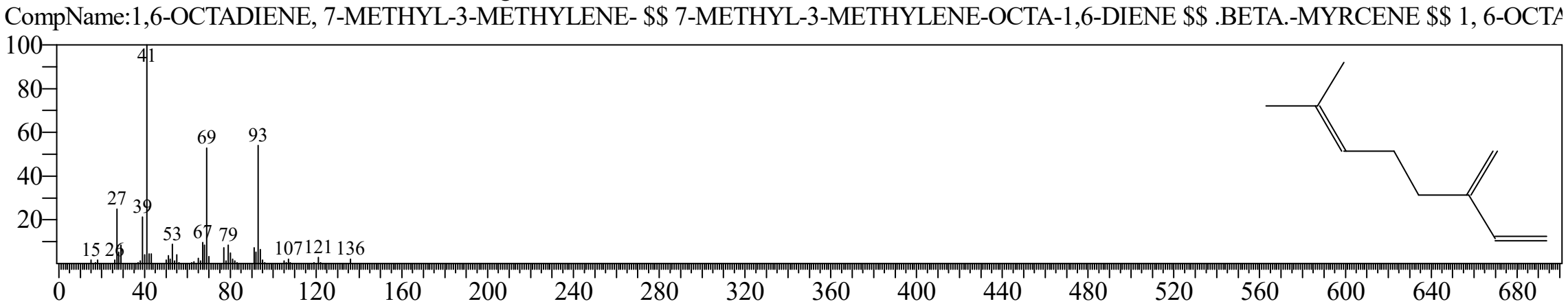

Hit#:3 Entry:46542 Library:WILEY8.LIB
SI:91 Formula:C10H18O CAS:106-24-1 MolWeight:154 RetIndex:0
CompName:2,6-OCTADIEN-1-OL, 3,7-DIMETHYL-, (E)- $$ 3,7-DIMETHYLOCTA-2,6-DIEN-1-OL $$ (2E)-3,7-DIMETHYL-2,6-OCTADIEN-1-OL #

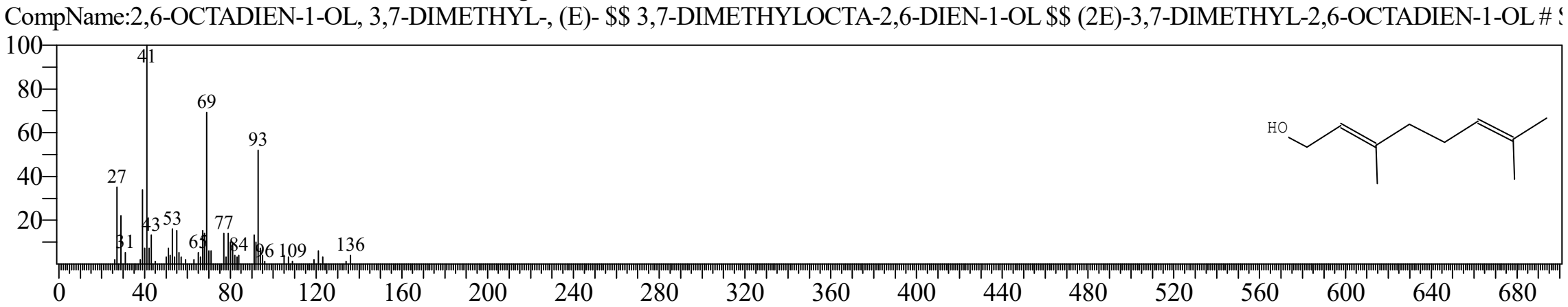

<< Target >>
Line#:15 R.Time:5.865(Scan#:474) MassPeaks:21
RawMode:Averaged 5.860-5.870(473-475) BasePeak:43.00(7819)
BG Mode:Calc. from Peak Group 1 - Event 1 Scan

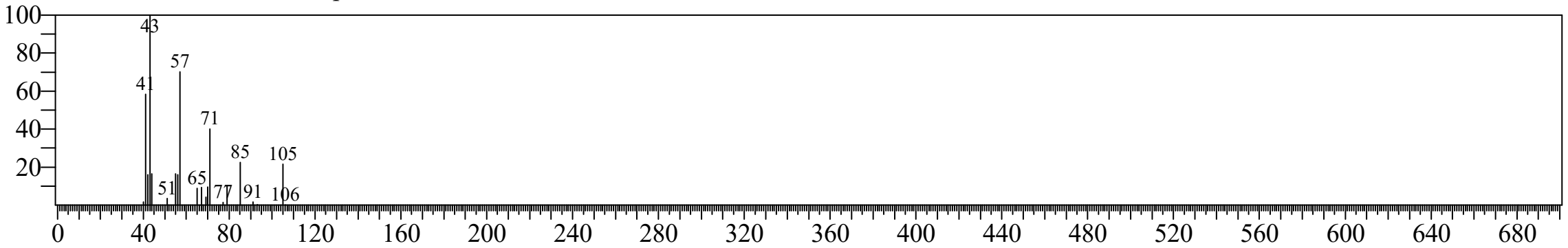

Hit#:1 Entry:22362 Library:WILEY8.LIB
SI:88 Formula:C9H20 CAS:0-00-0 MolWeight:128 RetIndex:0
CompName:NONANE $$ NONAN

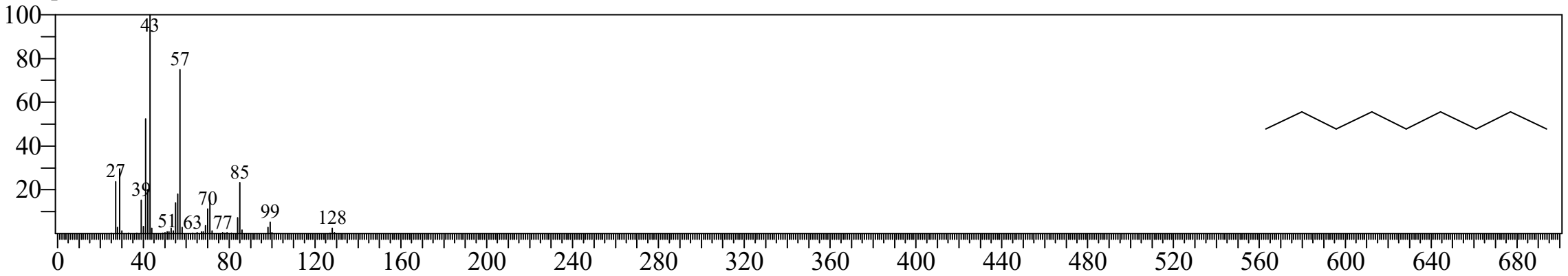

Hit#:2 Entry:49769 Library:WILEY8.LIB
SI:88 Formula:C11H24 CAS:6975-98-0 MolWeight:156 RetIndex:0
CompName:DECANE, 2-METHYL- $$ 2-METHYLDECANE $$ 2-METHYL-DECANE $$ DECANE, 2-METHYL- (8CI)(9CI) $$ EINECS 230-236-1 $

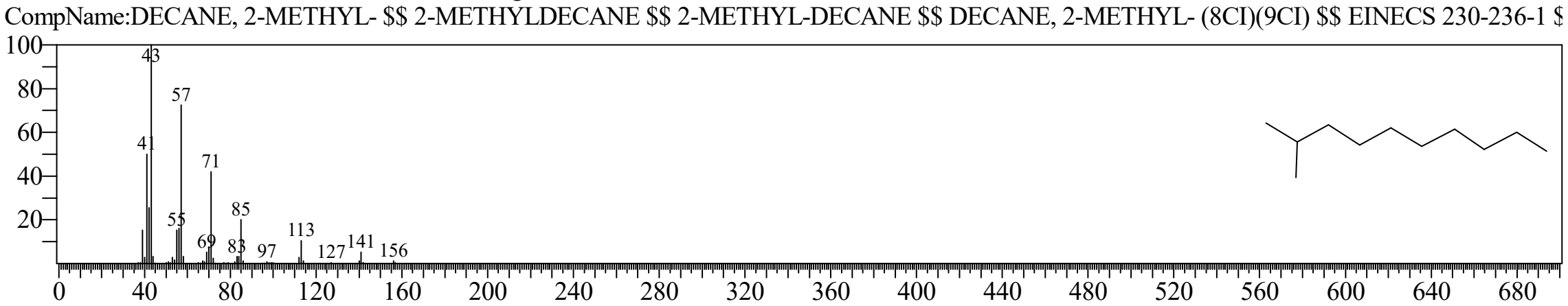

Hit#:3 Entry:101937 Library:NIST14.lib
SI:87 Formula:C10H21I CAS:0-00-0 MolWeight:268 RetIndex:1365
CompName:1-Iodo-2-methylnonane

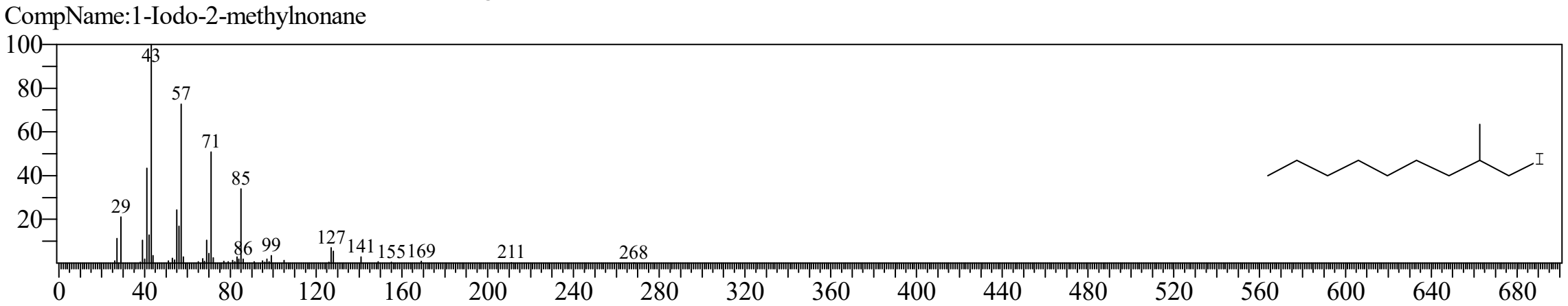

<< Target >>
Line#:16 R.Time:5.990(Scan#:499) MassPeaks:37
RawMode:Averaged 5.985-5.995(498-500) BasePeak:93.10(47823)
BG Mode:Calc. from Peak Group 1 - Event 1 Scan

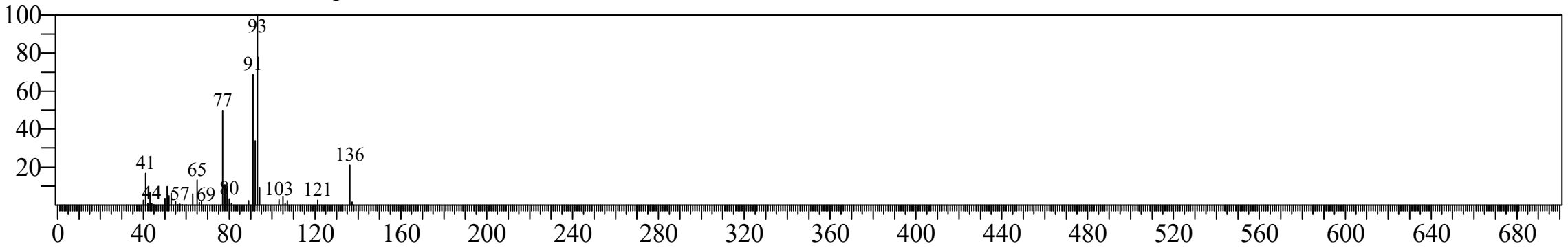

Hit#:1 Entry:28088 Library:WILEY8.LIB
SI:95 Formula:C10H16 CAS:99-83-2 MolWeight:136 RetIndex:0
CompName:1,3-CYCLOHEXADIENE, 2-METHYL-5-(1-METHYLETHYL)- $$ (-)-2-METHYL-5-(1-METHYLETHYL)-1,3-CYCLOHEXADIENE $$ (

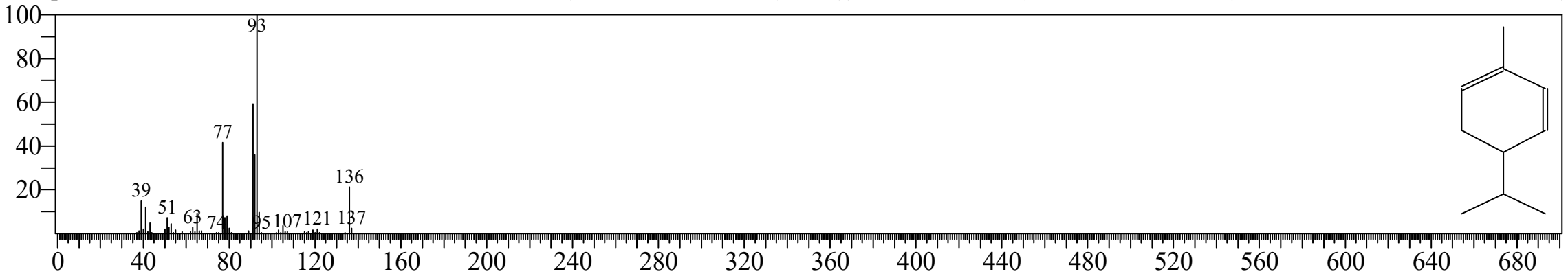

Hit#:2 Entry:28440 Library:WILEY8.LIB
SI:95 Formula:C10H16 CAS:1529-99-3 MolWeight:136 RetIndex:0
CompName:.ALPHA. PHELLANDRENE

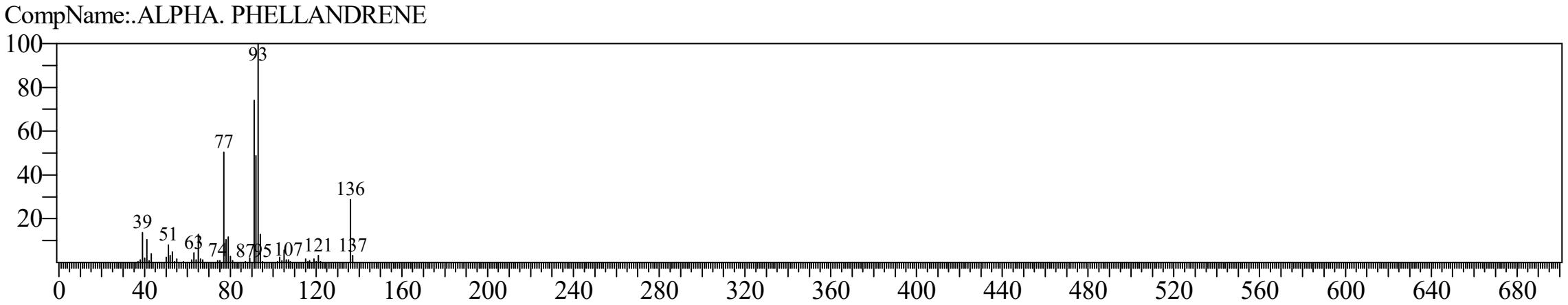

Hit#:3 Entry:6840 Library:NIST14s.lib
SI:95 Formula:C10H16 CAS:99-83-2 MolWeight:136 RetIndex:969
CompName:.alpha.-Phellandrene $$ 1,3-Cyclohexadiene, 2-methyl-5-(1-methylethyl)- $$ .alpha.-Fellandrene $$ p-Mentha-1,5-diene $$ 5-Isopropyl-2-methy

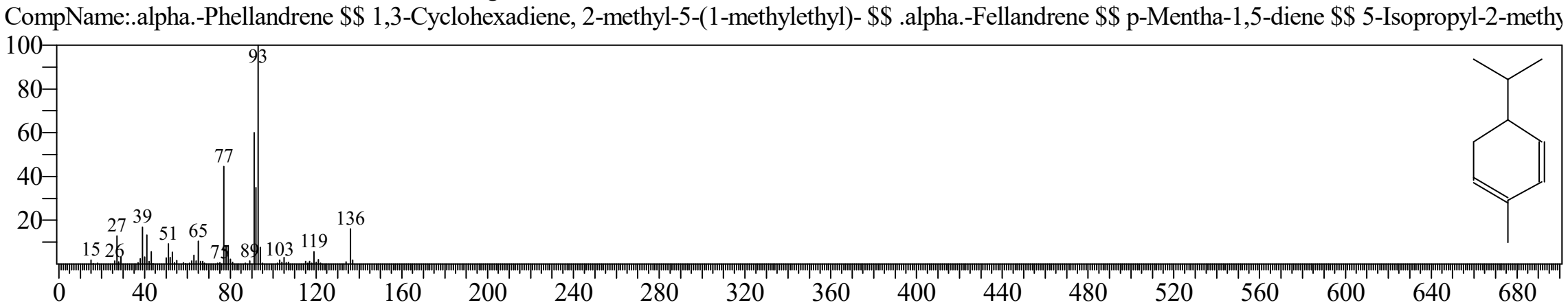

<< Target >>
Line#:17 R.Time:6.085(Scan#:518) MassPeaks:56
RawMode:Averaged 6.080-6.090(517-519) BasePeak:93.10(79422)
BG Mode:Calc. from Peak Group 1 - Event 1 Scan

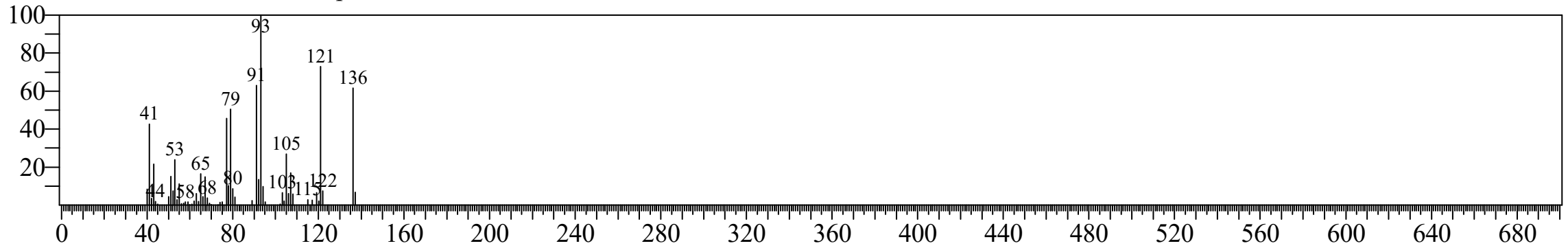

Hit#:1 Entry:28189 Library:WILEY8.LIB
SI:96 Formula:C10H16 CAS:586-62-9 MolWeight:136 RetIndex:0
CompName:CYCLOHEXENE, 1-METHYL-4-(1-METHYLETHYLIDENE)- $$ 1-METHYL-4-(1-METHYLETHYLIDENE)-1-CYCLOHEXENE # $$ .A

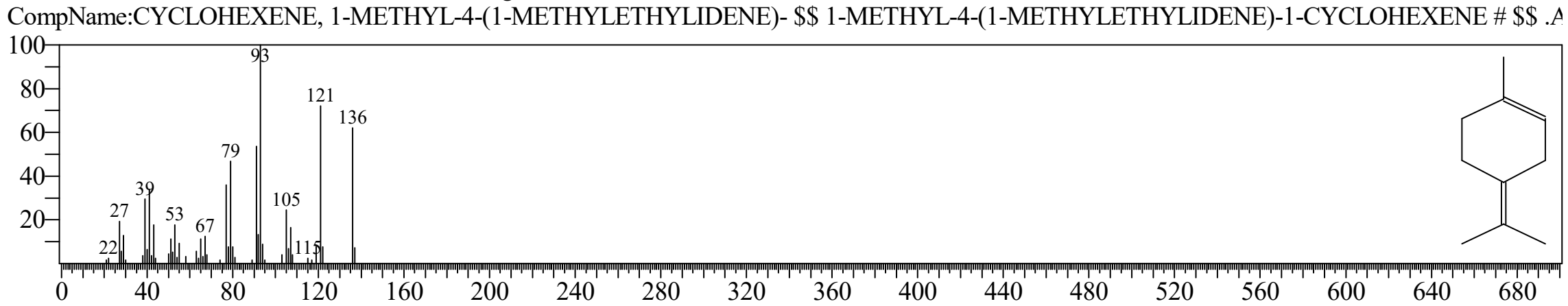

Hit#:2 Entry:28571 Library:WILEY8.LIB
SI:96 Formula:C10H16 CAS:586-62-9 MolWeight:136 RetIndex:0
CompName:1-METHYL-4-(1-METHYLETHYLIDENE)-1-CYCLOHEXENE $$ ALPHA-TERPINOLEN

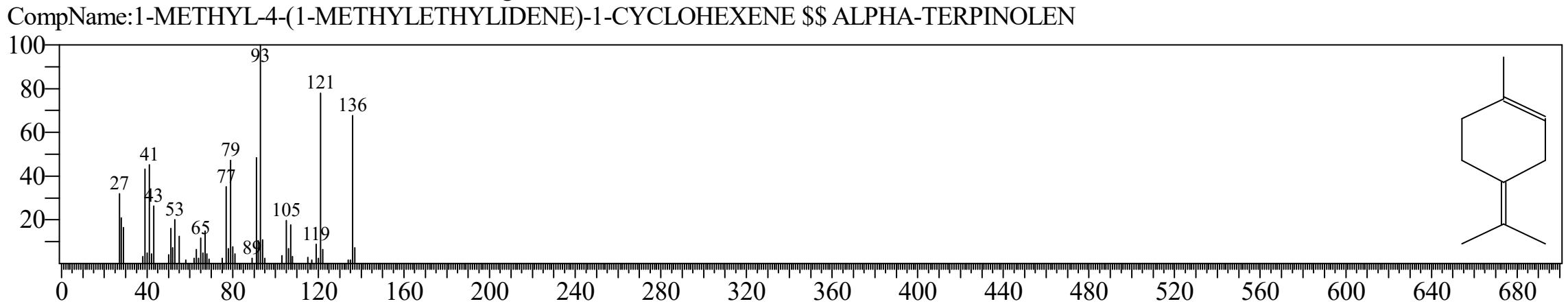

Hit#:3 Entry:28184 Library:WILEY8.LIB
SI:96 Formula:C10H16 CAS:586-62-9 MolWeight:136 RetIndex:0
CompName:CYCLOHEXENE, 1-METHYL-4-(1-METHYLETHYLIDENE)- $$ 1-METHYL-4-(1-METHYLETHYLIDENE)-1-CYCLOHEXENE # $$ .A

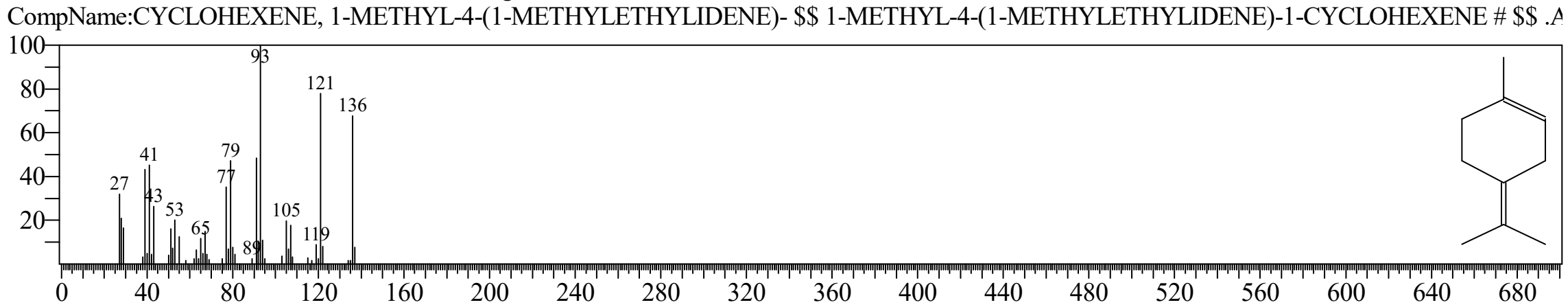

<< Target >>
Line#:18   R.Time:6.175(Scan#:536) MassPeaks:43
RawMode:Averaged 6.170-6.180(535-537) BasePeak:93.10(23148)
BG Mode:Calc. from Peak Group 1 - Event 1 Scan

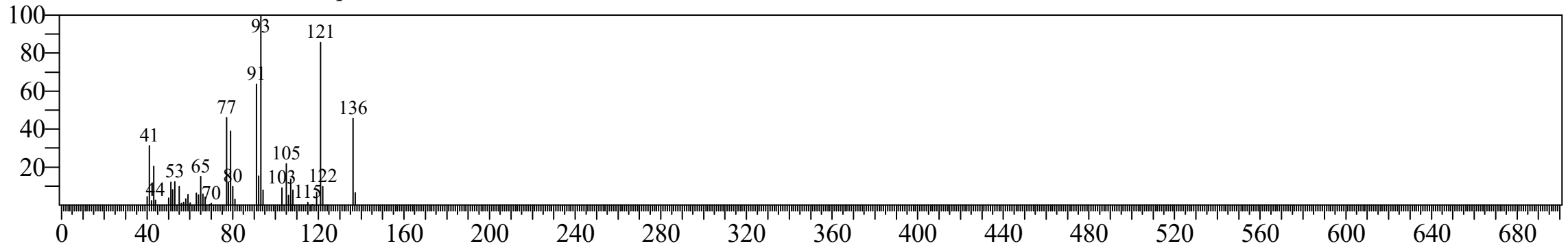

Hit#:1 Entry:28072 Library:WILEY8.LIB
SI:96 Formula:C10H16 CAS:99-86-5 MolWeight:136 RetIndex:0
CompName:1,3-CYCLOHEXADIENE, 1-METHYL-4-(1-METHYLETHYL)- $$ .ALPHA. TERPINENE $$ .ALPHA.-TERPINE $$ .ALPHA.-TERPINEN

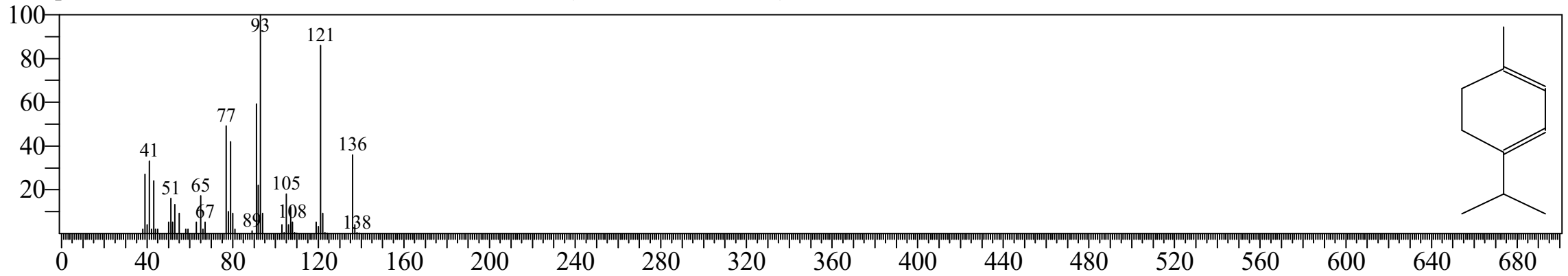

Hit#:2 Entry:28548 Library:WILEY8.LIB
SI:95 Formula:C10H16 CAS:99-86-5 MolWeight:136 RetIndex:0
CompName:1,3-CYCLOHEXADIENE, 1-METHYL-4-(1-METHYLETHYL)- $$ .ALPHA. TERPINENE $$ .ALPHA.-TERPINE $$ .ALPHA.-TERPINEN

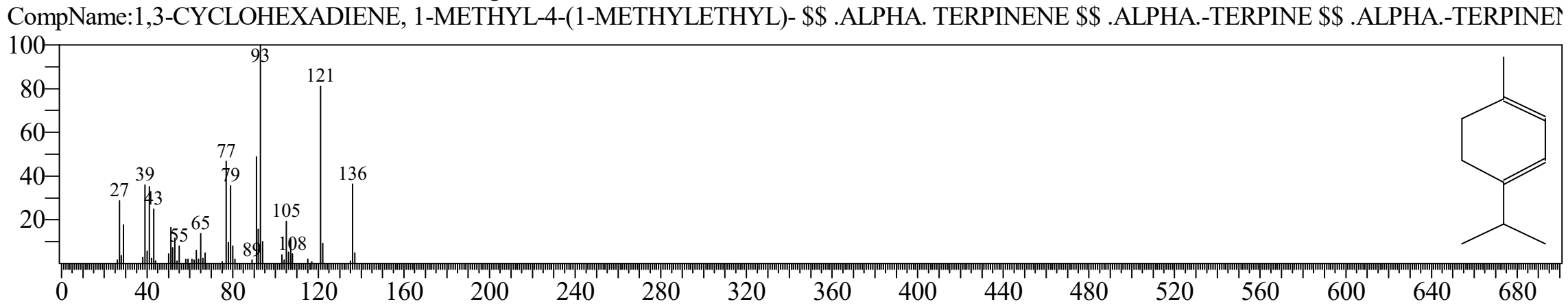

Hit#:3 Entry:28071 Library:WILEY8.LIB
SI:94 Formula:C10H16 CAS:99-86-5 MolWeight:136 RetIndex:0
CompName:1,3-CYCLOHEXADIENE, 1-METHYL-4-(1-METHYLETHYL)- $$ .ALPHA. TERPINENE $$ .ALPHA.-TERPINE $$ .ALPHA.-TERPINEN

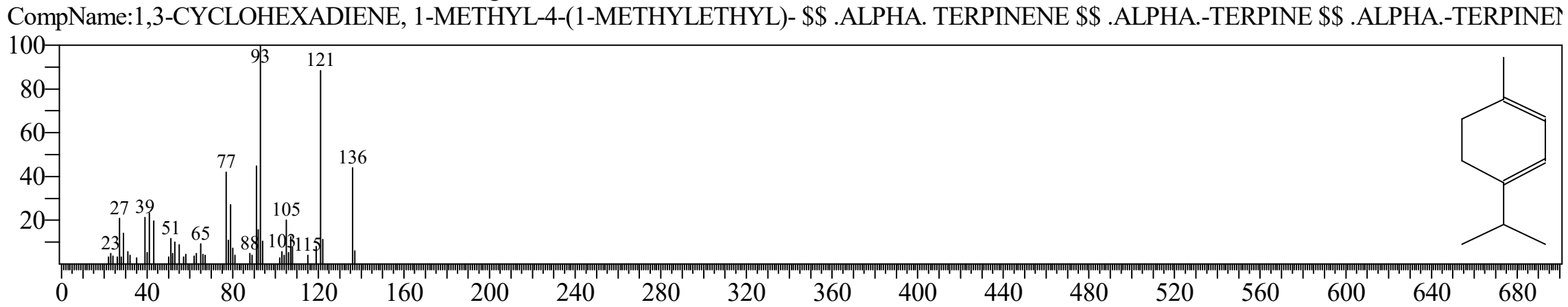

<< Target >>
Line#:19   R.Time:6.300(Scan#:561) MassPeaks:63
RawMode:Averaged 6.295-6.305(560-562) BasePeak:119.10(493558)
BG Mode:Calc. from Peak Group 1 - Event 1 Scan

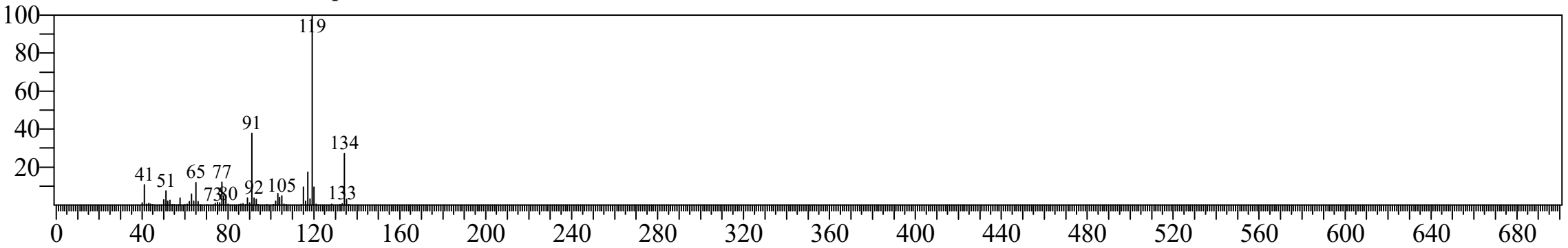

Hit#:1 Entry:6363 Library:NIST14s.lib
SI:97 Formula:C10H14 CAS:535-77-3 MolWeight:134 RetIndex:1042
CompName:Benzene, 1-methyl-3-(1-methylethyl)- $$ m-Cymene $$ .beta.-Cymene $$ m-Cymol $$ m-Isopropyltoluene $$ m-Methylisopropylbenzene $$ 1-

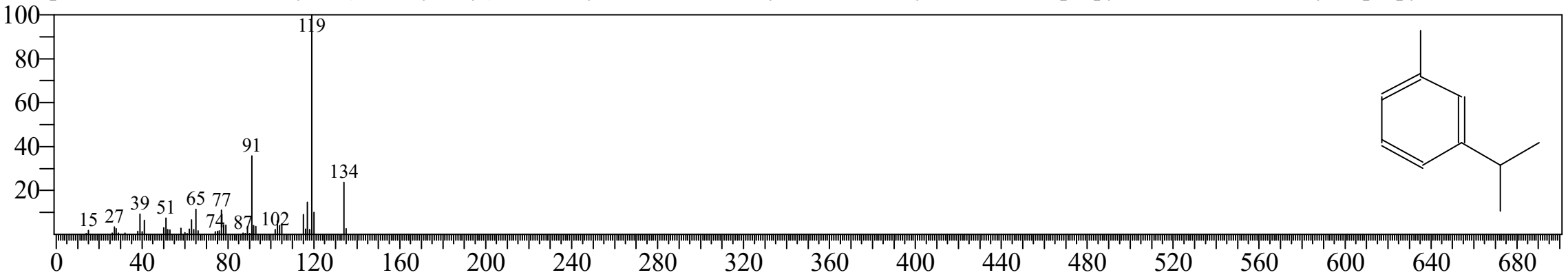

Hit#:2 Entry:6359 Library:NIST14s.lib
SI:97 Formula:C10H14 CAS:99-87-6 MolWeight:134 RetIndex:1042
CompName:p-Cymene $$ Benzene, 1-methyl-4-(1-methylethyl)- $$ p-Cimene $$ p-Cymol $$ p-Isopropyltoluene $$ p-Methylisopropylbenzene $$ Campho

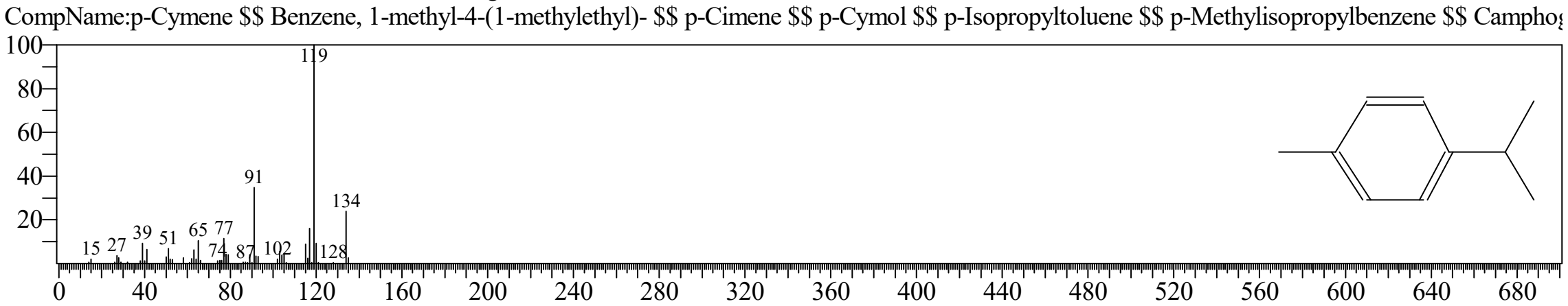

Hit#:3 Entry:9309 Library:NIST14.lib
SI:97 Formula:C10H14 CAS:527-84-4 MolWeight:134 RetIndex:1042
CompName:o-Cymene $$ Benzene, 1-methyl-2-(1-methylethyl)- $$ o-Cymol $$ o-Isopropyltoluene $$ 1-Isopropyl-2-methylbenzene $$ 1-Methyl-2-isoprop

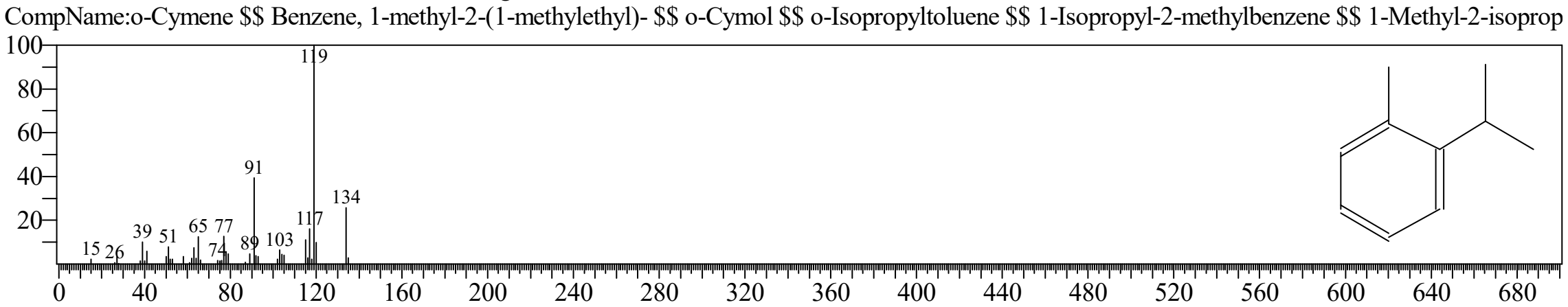

<< Target >>
Line#:20 R.Time:6.375(Scan#:576) MassPeaks:56
RawMode:Averaged 6.370-6.380(575-577) BasePeak:93.10(40237)
BG Mode:Calc. from Peak Group 1 - Event 1 Scan

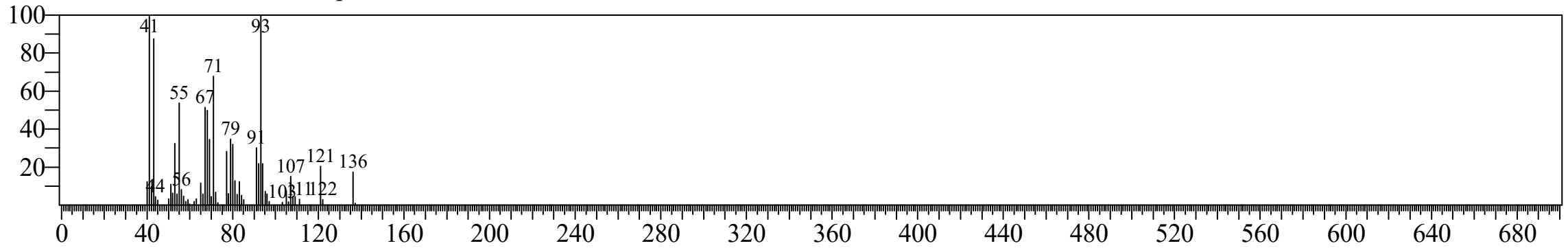

Hit#:1 Entry:46564 Library:WILEY8.LIB
SI:90 Formula:C10H18O CAS:78-70-6 MolWeight:154 RetIndex:0
CompName:1,6-OCTADIEN-3-OL, 3,7-DIMETHYL- $$ 3,7-DIMETHYLOCTA-1,6-DIEN-3-OL $$ (+-)-LINALOOL $$ (1)-3,7-DIMETHYL-1,6-OCTAD

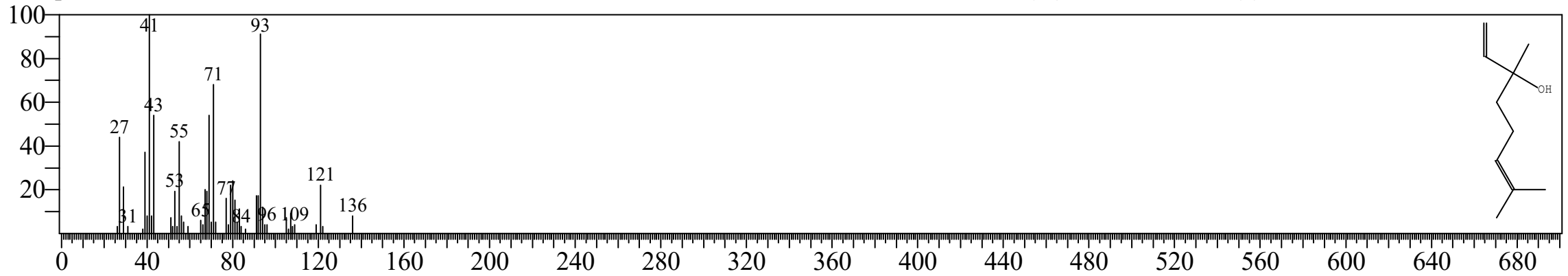

Hit#:2 Entry:47065 Library:WILEY8.LIB
SI:90 Formula:C10H18O CAS:78-70-6 MolWeight:154 RetIndex:0
CompName:1,6-OCTADIEN-3-OL, 3,7-DIMETHYL- $$ 3,7-DIMETHYLOCTA-1,6-DIEN-3-OL $$ (+-)-LINALOOL $$ (1)-3,7-DIMETHYL-1,6-OCTAD

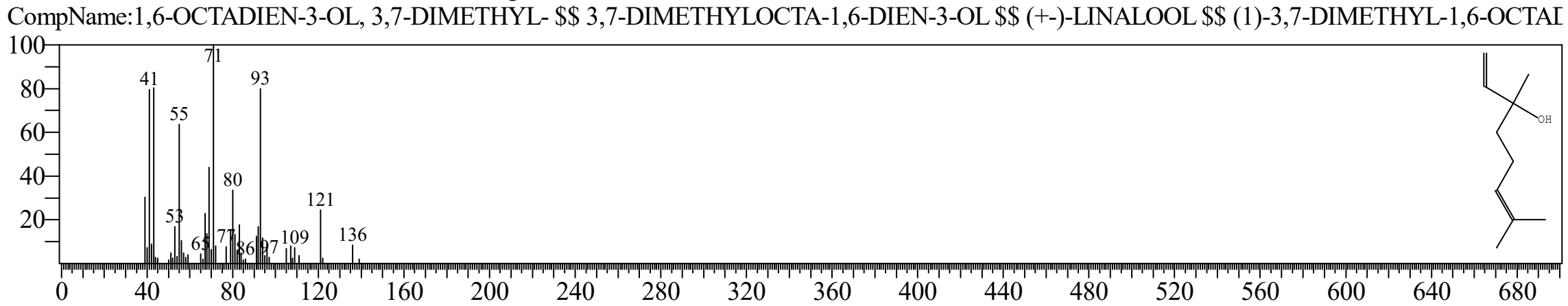

Hit#:3 Entry:47346 Library:WILEY8.LIB
SI:90 Formula:C10H18O CAS:0-00-0 MolWeight:154 RetIndex:0
CompName:3,7-DIMETHYL-1,6-OCTADIEN-3-OL $$ BETA-LINALOOL

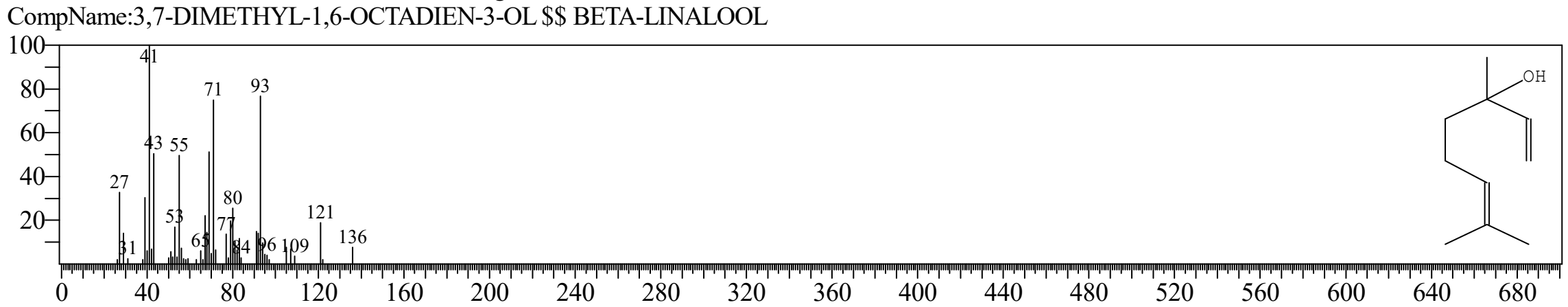

<< Target >>
Line#:21   R.Time:6.470(Scan#:595) MassPeaks:37
RawMode:Averaged 6.465-6.475(594-596) BasePeak:93.10(14389)
BG Mode:Calc. from Peak Group 1 - Event 1 Scan

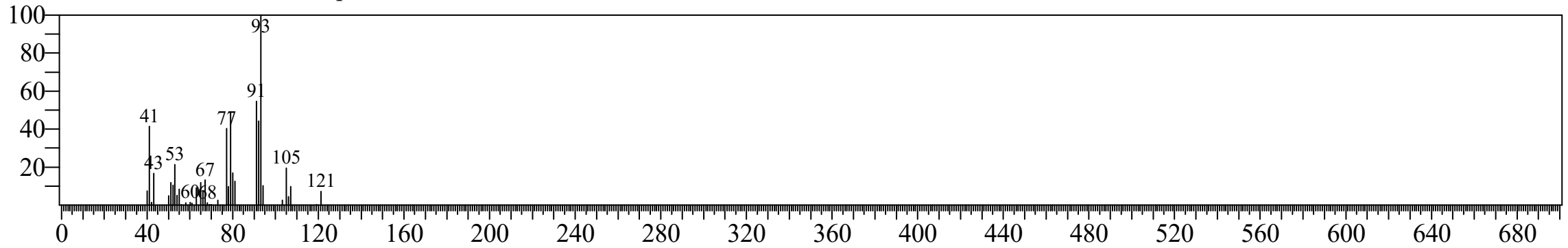

Hit#:1 Entry:6803 Library:NIST14s.lib
SI:94 Formula:C10H16 CAS:7785-26-4 MolWeight:136 RetIndex:948
CompName:(1S)-2,6,6-Trimethylbicyclo[3.1.1]hept-2-ene $$ 1S-.alpha.-Pinene $$ (-)-.alpha.-Pinene $$ L-.alpha.-Pinene $$ Bicyclo[3.1.1]hept-2-ene, 2,6,6-

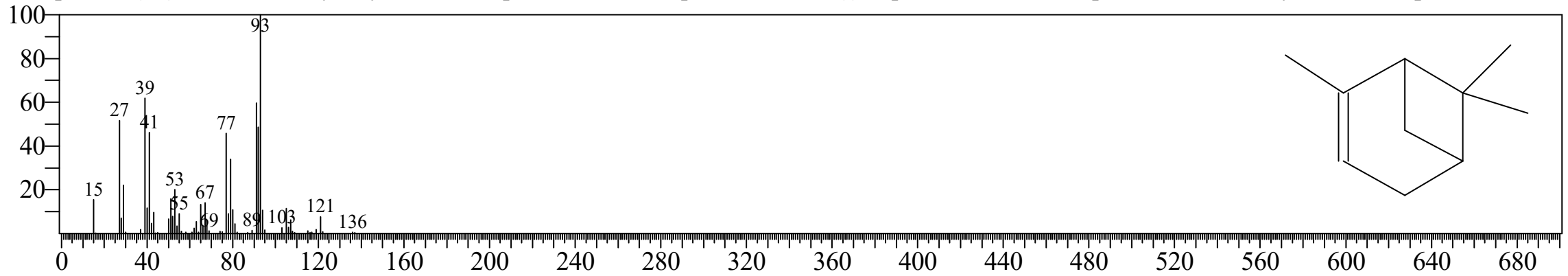

Hit#:2 Entry:27980 Library:WILEY8.LIB
SI:94 Formula:C10H16 CAS:3338-55-4 MolWeight:136 RetIndex:0
CompName:1,3,6-OCTATRIENE, 3,7-DIMETHYL-, (Z)- $$ (3Z)-3,7-DIMETHYL-1,3,6-OCTATRIENE # $$ (3Z)-3,7-DIMETHYL-1,3,6-OCTATRIENE

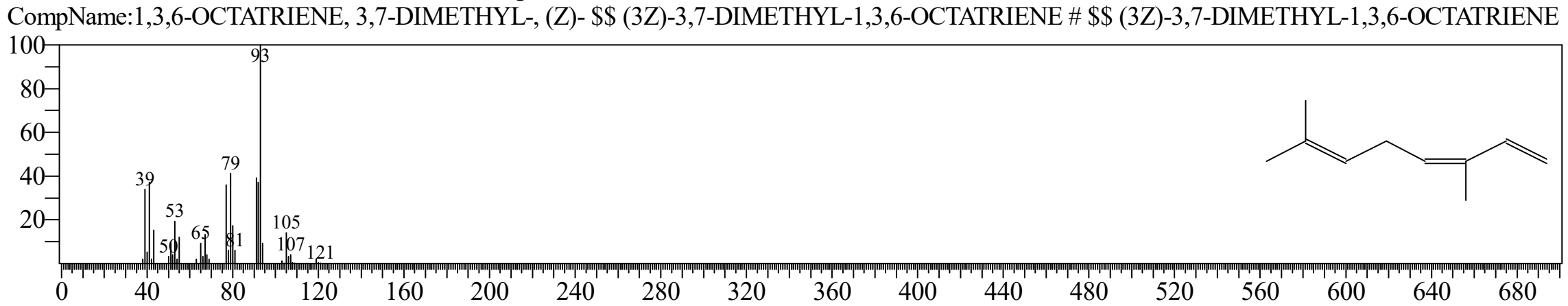

Hit#:3 Entry:27983 Library:WILEY8.LIB
SI:93 Formula:C10H16 CAS:3779-61-1 MolWeight:136 RetIndex:0
CompName:1,3,6-OCTATRIENE, 3,7-DIMETHYL-, (E)- $$ (3E)-3,7-DIMETHYL-1,3,6-OCTATRIENE # $$ (3E)-3,7-DIMETHYL-1,3,6-OCTATRIENE

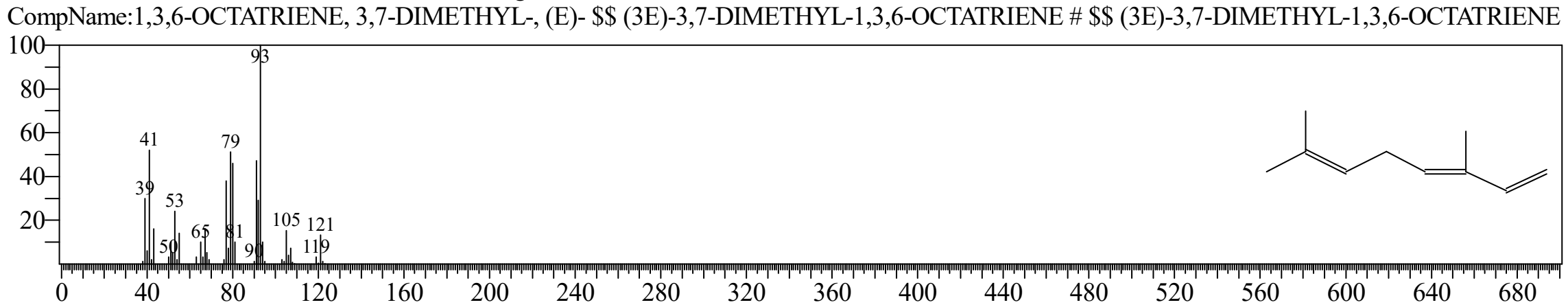

<< Target >>
Line#:22 R.Time:6.695(Scan#:640) MassPeaks:42
RawMode:Averaged 6.690-6.700(639-641) BasePeak:43.00(17646)
BG Mode:Calc. from Peak Group 1 - Event 1 Scan

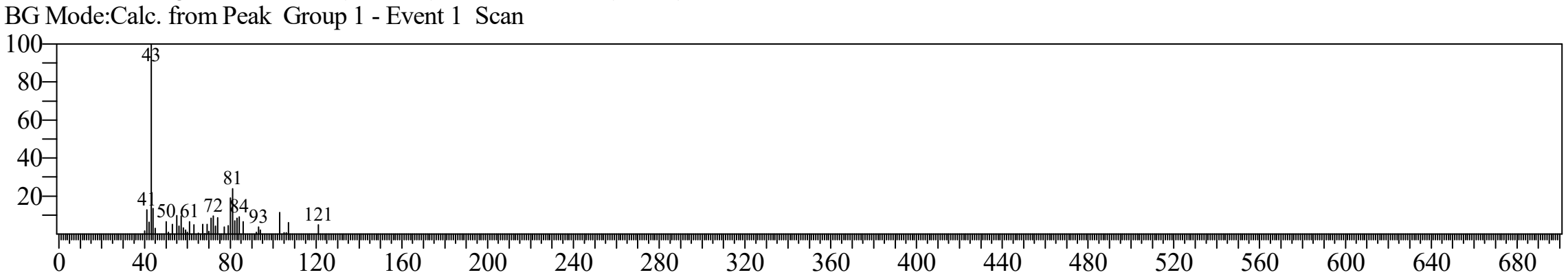

Hit#:1 Entry:4043 Library:NIST14.lib
SI:79 Formula:C6H10O2 CAS:0-00-0 MolWeight:114 RetIndex:793
CompName:2,3-Dioxabicyclo[2.2.1]heptane, 1-methyl- $$ 1-Methyl-2,3-dioxabicyclo[2.2.1]heptane # $$

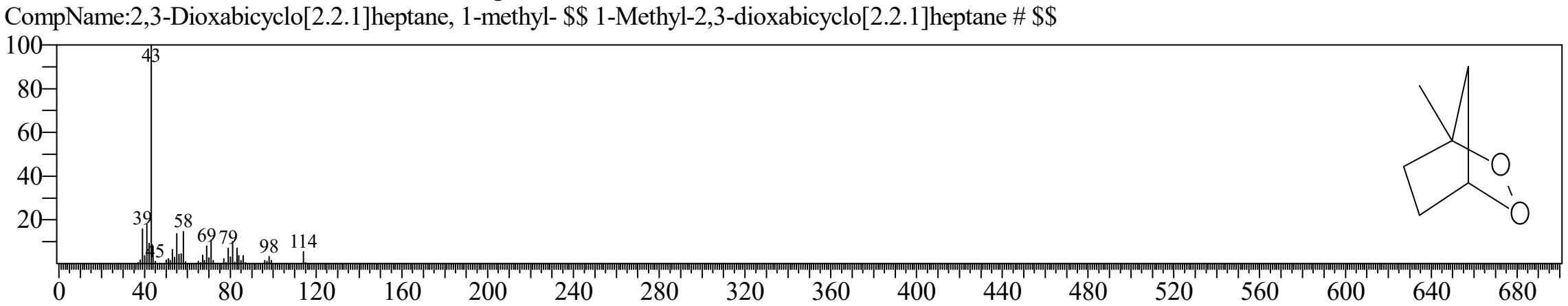

Hit#:2 Entry:17958 Library:NIST14.lib
SI:79 Formula:C10H18O CAS:62911-76-6 MolWeight:154 RetIndex:1041
CompName:3,7-Octadien-2-ol, 2,6-dimethyl- $$ (3E)-2,6-Dimethyl-3,7-octadien-2-ol # $$

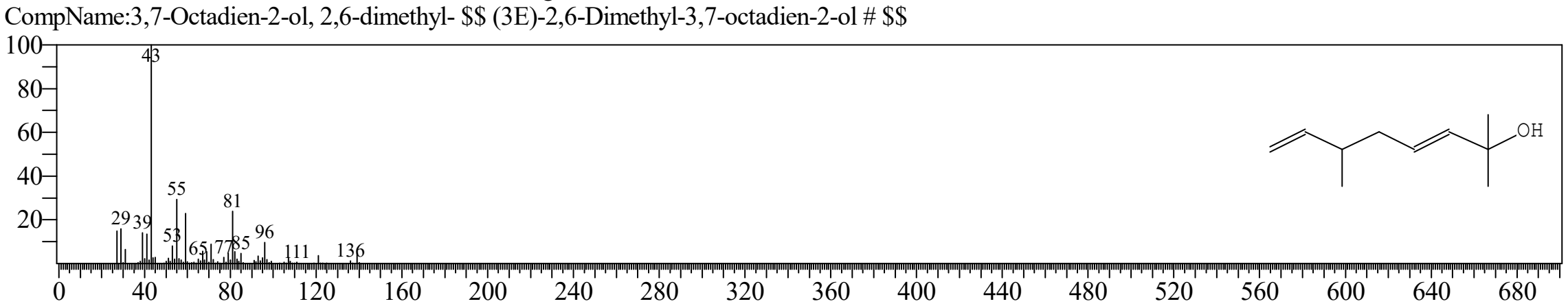

Hit#:3 Entry:47355 Library:WILEY8.LIB
SI:79 Formula:C10H18O CAS:62911-76-6 MolWeight:154 RetIndex:0
CompName:(3E)-2,6-DIMETHYL-3,7-OCTADIEN-2-OL # $$ (3E)-2,6-DIMETHYL-3,7-OCTADIEN-2-OL $$ 2,6-DIMETHYL-3,7-OCTADIEN-2-OL $

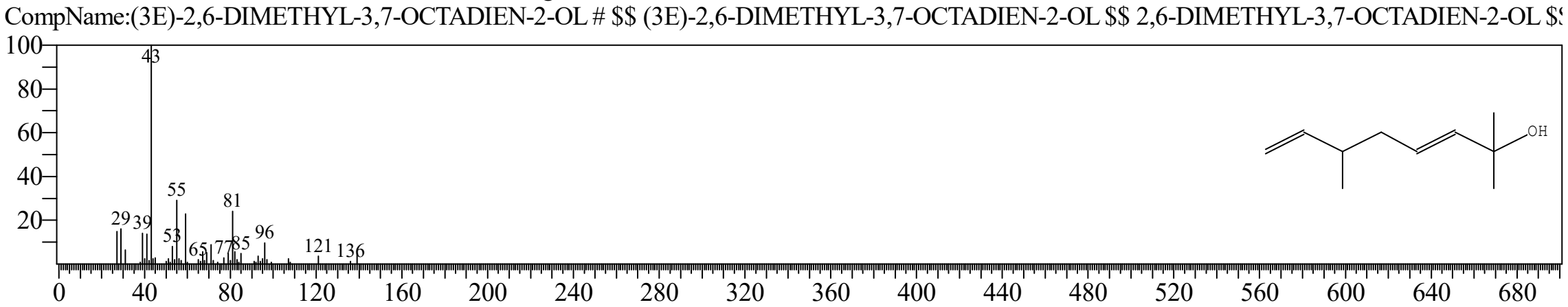

<< Target >>
Line#:23   R.Time:6.825(Scan#:666) MassPeaks:80
RawMode:Averaged 6.820-6.830(665-667) BasePeak:93.10(1628596)
BG Mode:Calc. from Peak Group 1 - Event 1 Scan

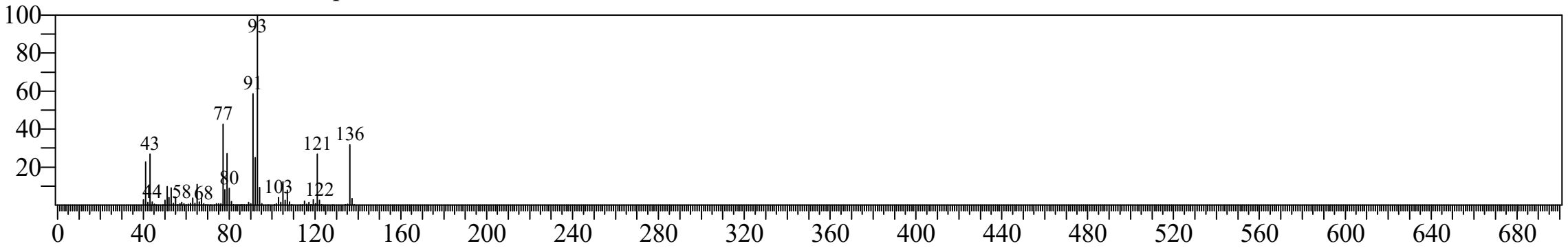

Hit#:1 Entry:28552 Library:WILEY8.LIB
SI:97 Formula:C10H16 CAS:99-85-4 MolWeight:136 RetIndex:0
CompName:1,4-CYCLOHEXADIENE, 1-METHYL-4-(1-METHYLETHYL)- $$ 1-ISOPROPYL-4-METHYL-1,4-CYCLOHEXADIENE # $$ .GAMMA.

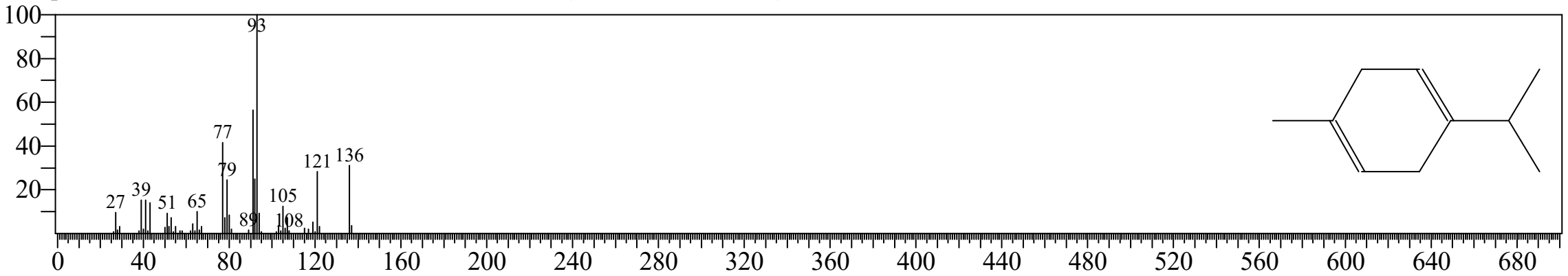

Hit#:2 Entry:6836 Library:NIST14s.lib
SI:97 Formula:C10H16 CAS:99-85-4 MolWeight:136 RetIndex:998
CompName:.gamma.-Terpinene $$ 1,4-Cyclohexadiene, 1-methyl-4-(1-methylethyl)- $$ .gamma.-Terpinen $$ p-Mentha-1,4-diene $$ Crithmene $$ Moslene

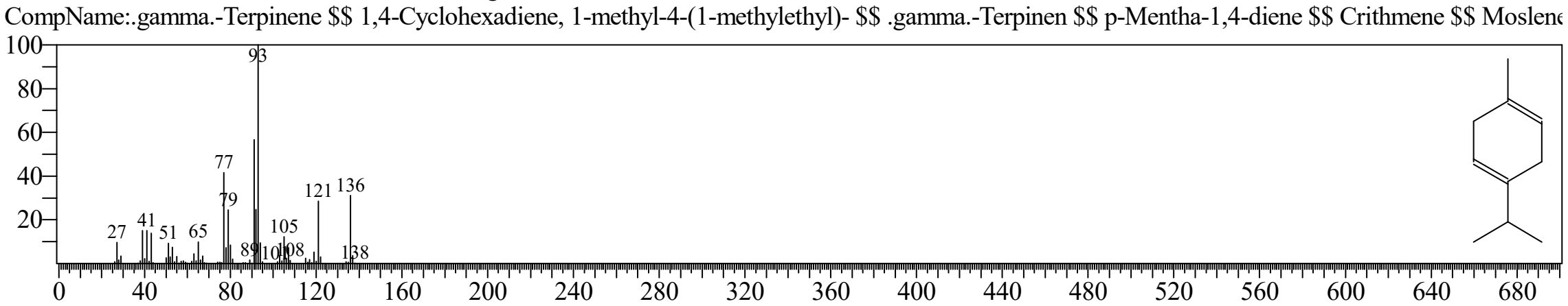

Hit#:3 Entry:28119 Library:WILEY8.LIB
SI:96 Formula:C10H16 CAS:99-85-4 MolWeight:136 RetIndex:0
CompName:1,4-CYCLOHEXADIENE, 1-METHYL-4-(1-METHYLETHYL)- $$ 1-ISOPROPYL-4-METHYL-1,4-CYCLOHEXADIENE # $$ .GAMMA.

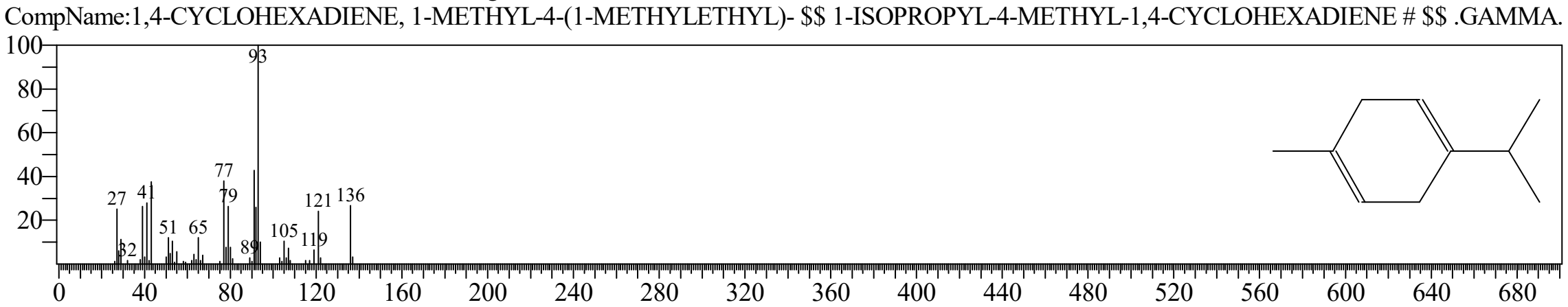

<< Target >>
Line#:24   R.Time:6.970(Scan#:695) MassPeaks:37
RawMode:Averaged 6.965-6.975(694-696) BasePeak:43.00(3247)
BG Mode:Calc. from Peak Group 1 - Event 1 Scan

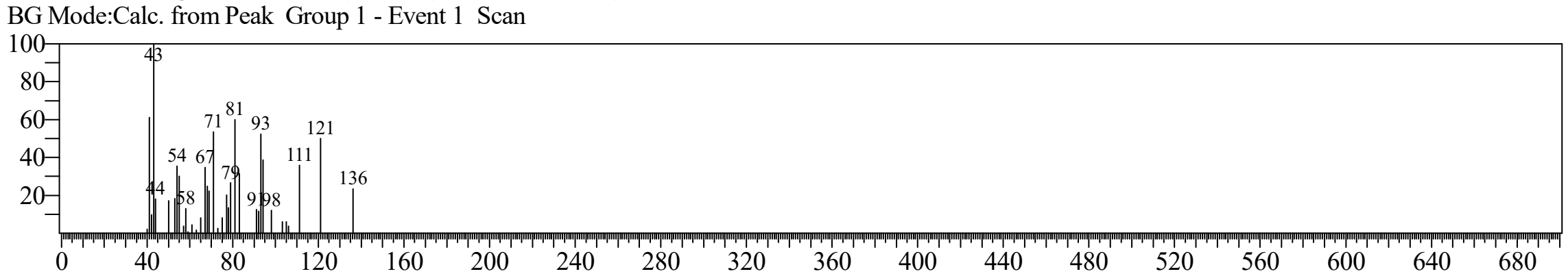

Hit#:1 Entry:17979 Library:NIST14.lib
SI:81 Formula:C10H18O CAS:15537-55-0 MolWeight:154 RetIndex:1041
CompName:Bicyclo[3.1.0]hexan-2-ol, 2-methyl-5-(1-methylethyl)-, (1.alpha.,2.beta.,5.alpha.)- $$ 5-Isopropyl-2-methylbicyclo[3.1.0]hexan-2-ol-, (1.alpha.,2

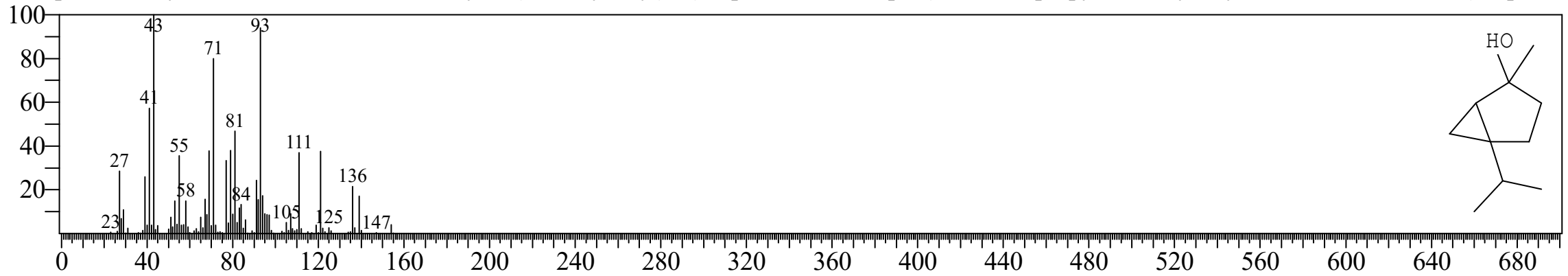

Hit#:2 Entry:46833 Library:WILEY8.LIB
SI:81 Formula:C10H18O CAS:546-79-2 MolWeight:154 RetIndex:0
CompName:5-ISOPROPYL-2-METHYLBICYCLO[3.1.0]HEXAN-2-OL # $$ 2-METHYL-5-(1-METHYLETHYL)BICYCLO(3.1.0)HEXAN-2-OL $$ 4-

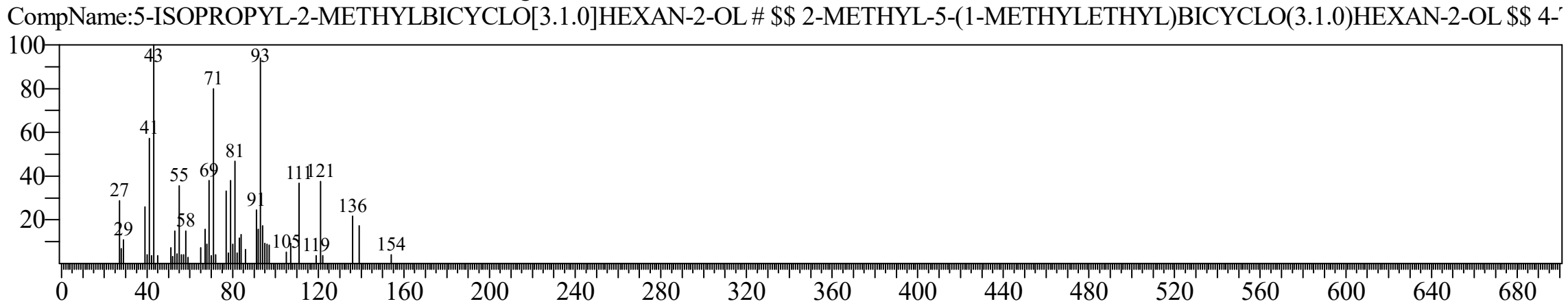

Hit#:3 Entry:47053 Library:WILEY8.LIB
SI:79 Formula:C10H18O CAS:15826-82-1 MolWeight:154 RetIndex:0
CompName:4-THUJANOL, CIS-(.+-.)- $$ BICYCLO[3.1.0]HEXAN-2-OL, 2-METHYL-5-(1-METHYLETHYL)-, (1.ALPHA.,2.BETA.,5.ALPHA.)-(.+-.)-

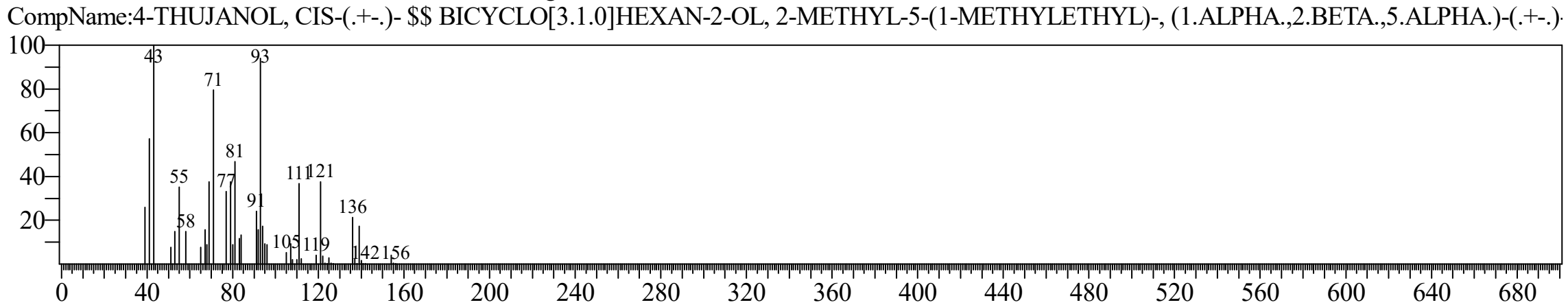

<< Target >>
Line#:25 R.Time:7.110(Scan#:723) MassPeaks:40
RawMode:Averaged 7.105-7.115(722-724) BasePeak:43.00(4956)
BG Mode:Calc. from Peak Group 1 - Event 1 Scan

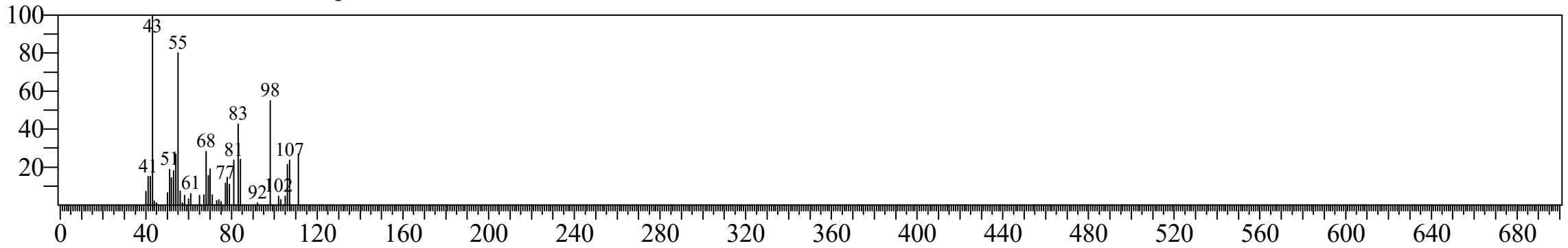

Hit#:1 Entry:45063 Library:WILEY8.LIB
SI:76 Formula:C9H15NO CAS:56336-06-2 MolWeight:153 RetIndex:0
CompName:2-CYCLOHEXEN-1-ONE, 3,5-DIMETHYL-, O-METHYLOXIME $$ (1Z)-3,5-DIMETHYL-2-CYCLOHEXEN-1-ONE O-METHYLOXIMI

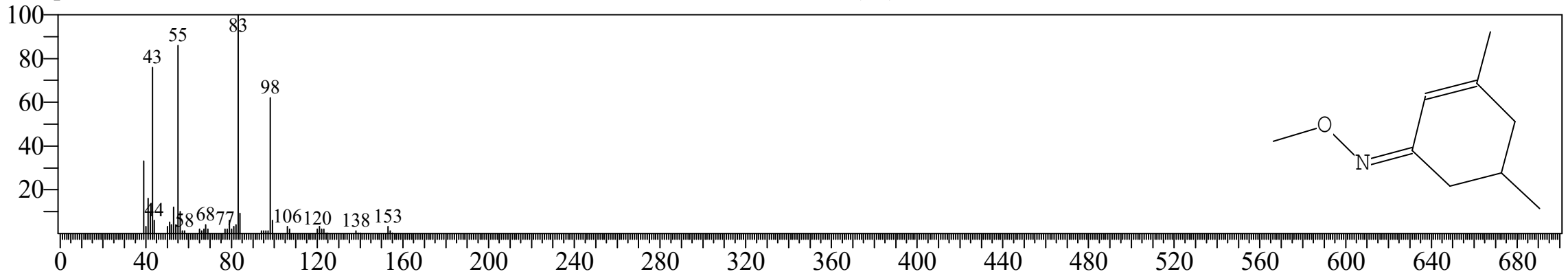

Hit#:2 Entry:17305 Library:NIST14.lib
SI:76 Formula:C9H15NO CAS:56336-06-2 MolWeight:153 RetIndex:1221
CompName:2-Cyclohexen-1-one, 3,5-dimethyl-, O-methyloxime $$ (1Z)-3,5-Dimethyl-2-cyclohexen-1-one o-methyloxime # $$

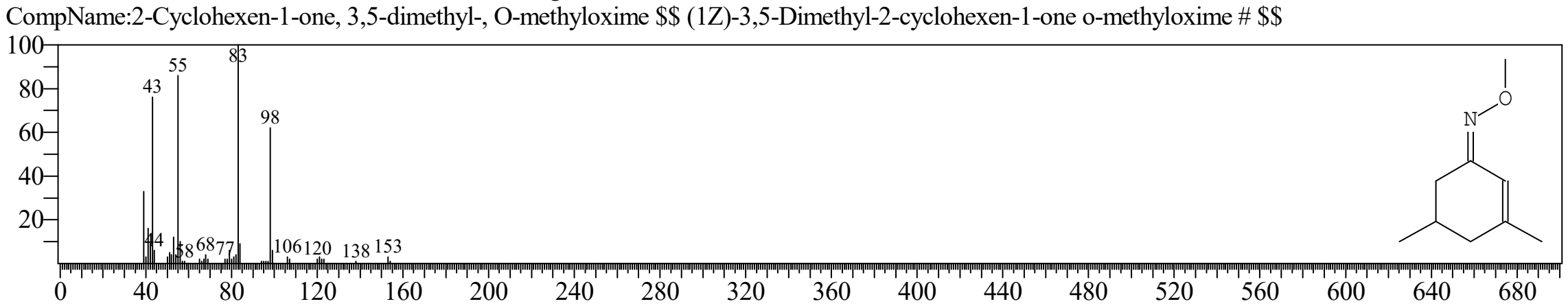

Hit#:3 Entry:31781 Library:WILEY8.LIB
SI:76 Formula:C9H16O CAS:18829-56-6 MolWeight:140 RetIndex:0
CompName:2-NONENAL $$ 2-NONENAL, (E)- $$ (2E)-2-NONENAL # $$ (2E)-2-NONENAL $$ (2E)-2-NONENAL (COMPUTER-GENERATED NAI

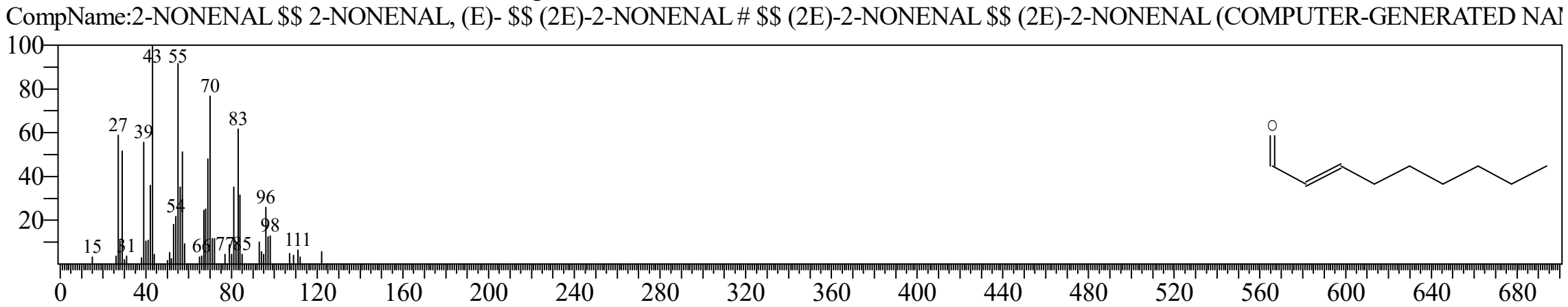

<< Target >>
Line#:26   R.Time:7.280(Scan#:757) MassPeaks:67
RawMode:Averaged 7.275-7.285(756-758) BasePeak:93.10(96070)
BG Mode:Calc. from Peak Group 1 - Event 1 Scan

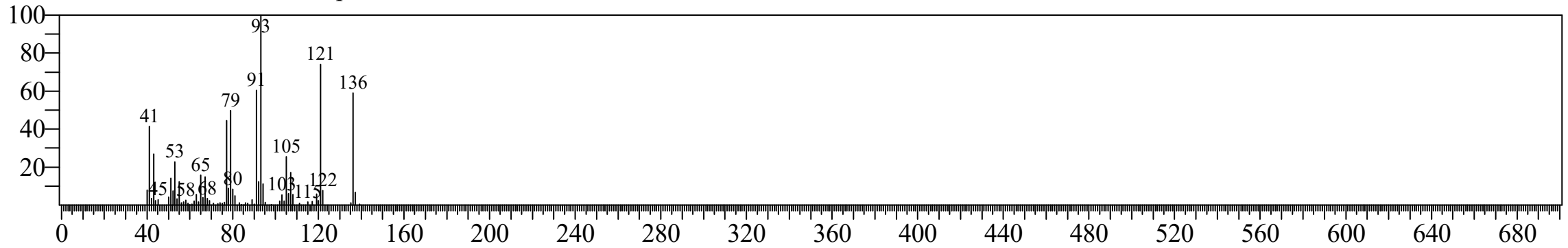

Hit#:1 Entry:28571 Library:WILEY8.LIB
SI:96 Formula:C10H16 CAS:586-62-9 MolWeight:136 RetIndex:0
CompName:1-METHYL-4-(1-METHYLETHYLIDENE)-1-CYCLOHEXENE $$ ALPHA-TERPINOLEN

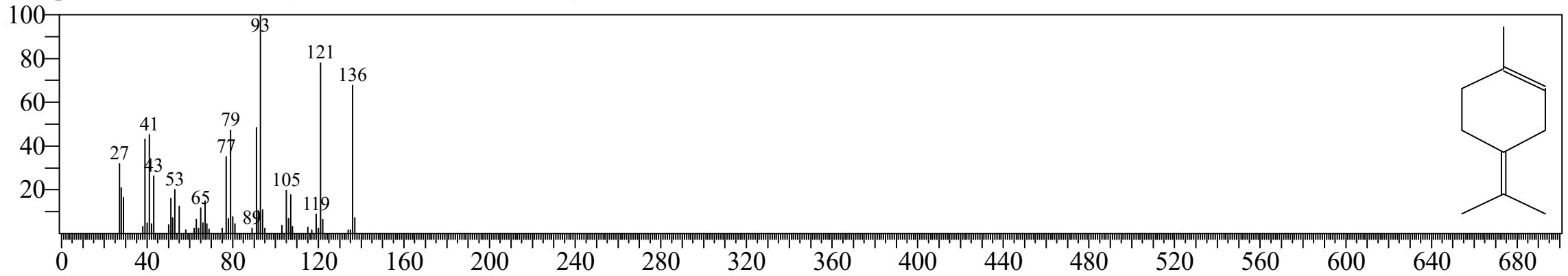

Hit#:2 Entry:28184 Library:WILEY8.LIB
SI:96 Formula:C10H16 CAS:586-62-9 MolWeight:136 RetIndex:0
CompName:CYCLOHEXENE, 1-METHYL-4-(1-METHYLETHYLIDENE)- $$ 1-METHYL-4-(1-METHYLETHYLIDENE)-1-CYCLOHEXENE # $$ .A

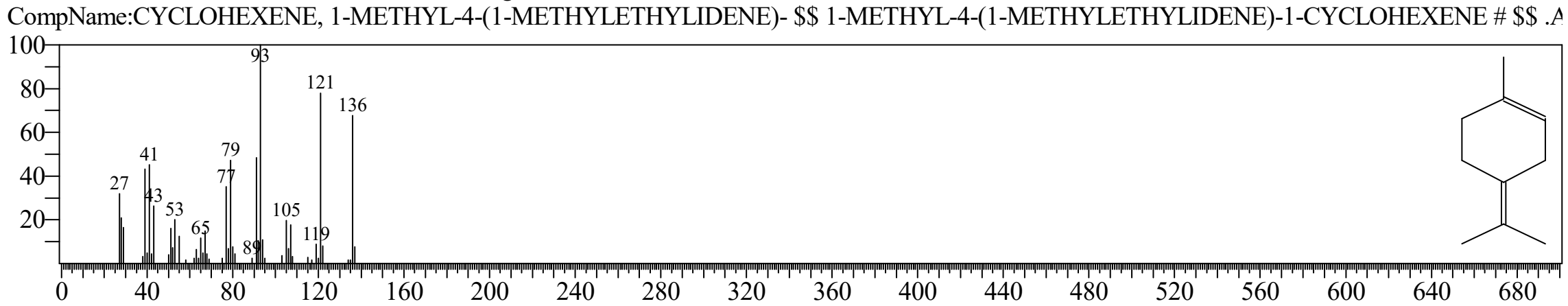

Hit#:3 Entry:28189 Library:WILEY8.LIB
SI:96 Formula:C10H16 CAS:586-62-9 MolWeight:136 RetIndex:0
CompName:CYCLOHEXENE, 1-METHYL-4-(1-METHYLETHYLIDENE)- $$ 1-METHYL-4-(1-METHYLETHYLIDENE)-1-CYCLOHEXENE # $$ .A

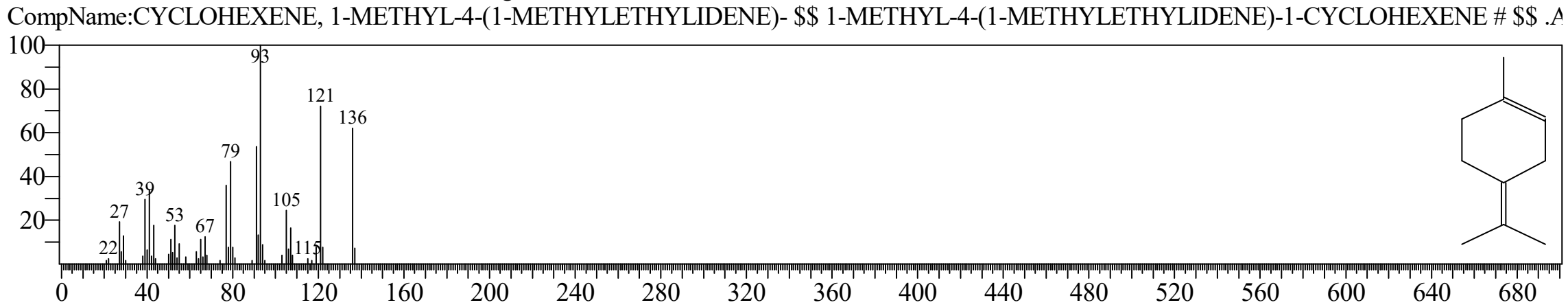

<< Target >>
Line#:27   R.Time:7.375(Scan#:776) MassPeaks:57
RawMode:Averaged 7.370-7.380(775-777) BasePeak:41.05(8777)
BG Mode:Calc. from Peak Group 1 - Event 1 Scan

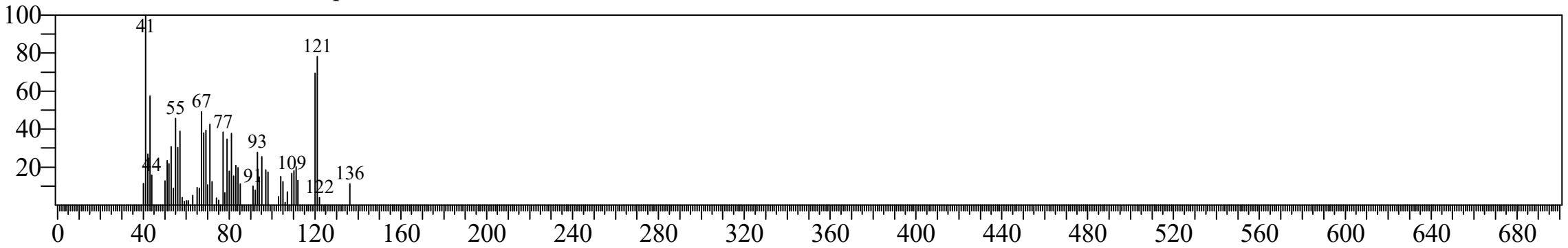

Hit#:1 Entry:46772 Library:WILEY8.LIB
SI:86 Formula:C10H18O CAS:89-79-2 MolWeight:154 RetIndex:0
CompName:CYCLOHEXANOL, 5-METHYL-2-(1-METHYLETHENYL)-, [1R-(1.ALPHA.,2.BETA.,5.ALPHA.)]- $$ 2-ISOPROPENYL-5-METHYLCY

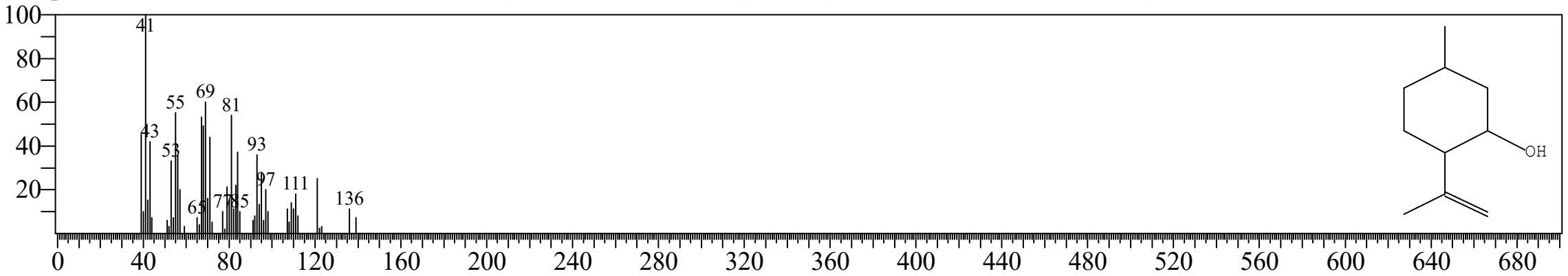

Hit#:2 Entry:47083 Library:WILEY8.LIB
SI:85 Formula:C10H18O CAS:21290-09-5 MolWeight:154 RetIndex:0
CompName:NEOISO(ISO)PULEGOL

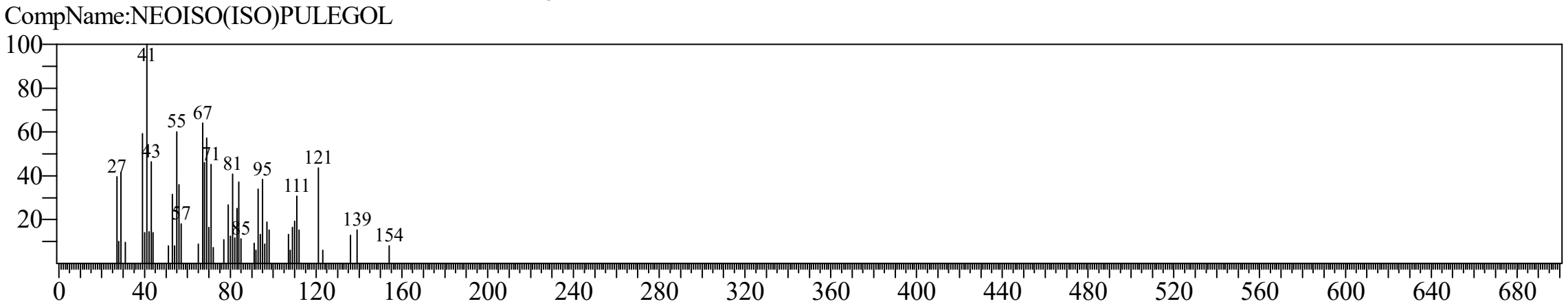

Hit#:3 Entry:46773 Library:WILEY8.LIB
SI:83 Formula:C10H18O CAS:89-79-2 MolWeight:154 RetIndex:0
CompName:CYCLOHEXANOL, 5-METHYL-2-(1-METHYLETHENYL)-, [1R-(1.ALPHA.,2.BETA.,5.ALPHA.)]- $$ 2-ISOPROPENYL-5-METHYLCY

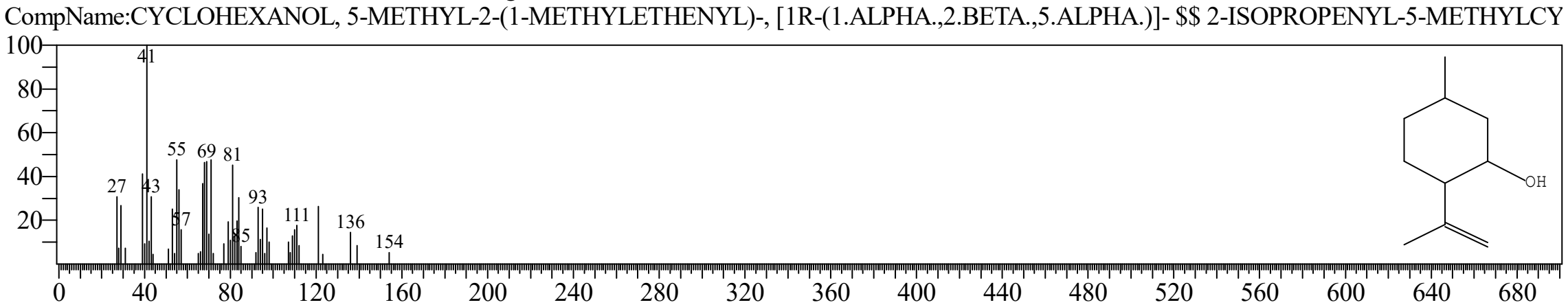

<< Target >>
Line#:28   R.Time:7.435(Scan#:788) MassPeaks:59
RawMode:Averaged 7.430-7.440(787-789) BasePeak:43.00(46365)
BG Mode:Calc. from Peak Group 1 - Event 1 Scan

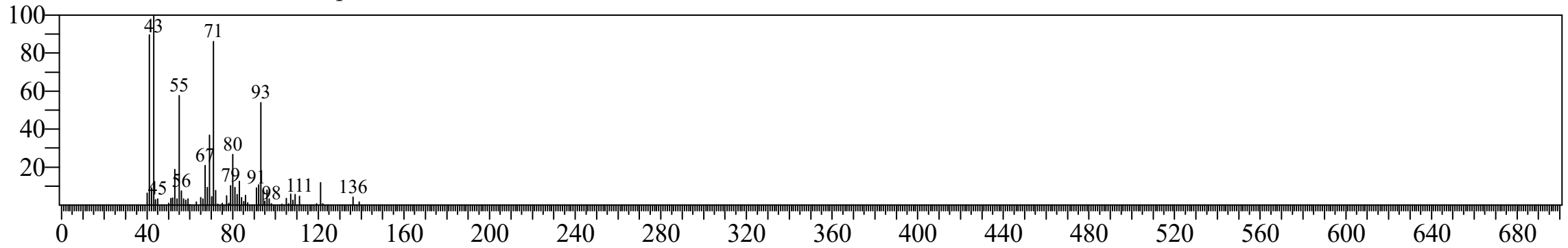

Hit#:1 Entry:28190 Library:WILEY8.LIB
SI:95 Formula:C10H16 CAS:586-62-9 MolWeight:136 RetIndex:0
CompName:CYCLOHEXENE, 1-METHYL-4-(1-METHYLETHYLIDENE)- $$ 1-METHYL-4-(1-METHYLETHYLIDENE)-1-CYCLOHEXENE # $$ .A

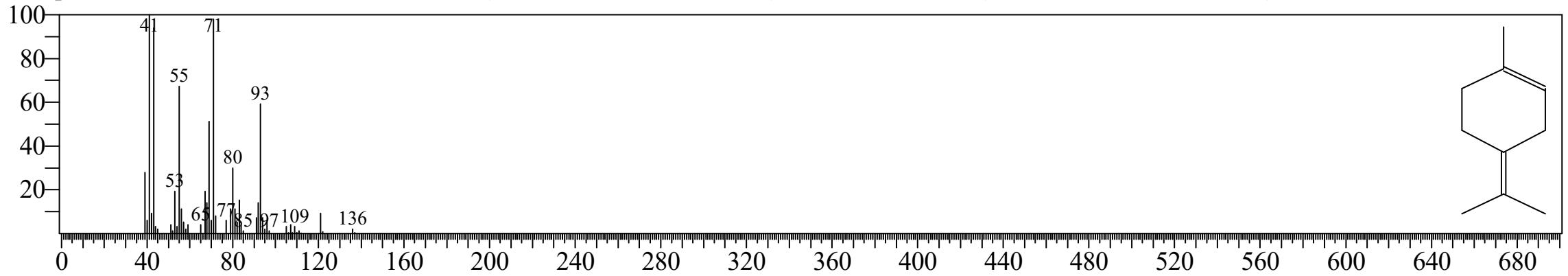

Hit#:2 Entry:46565 Library:WILEY8.LIB
SI:95 Formula:C10H18O CAS:78-70-6 MolWeight:154 RetIndex:0
CompName:1,6-OCTADIEN-3-OL, 3,7-DIMETHYL- $$ 3,7-DIMETHYLOCTA-1,6-DIEN-3-OL $$ (+-)-LINALOOL $$ (1)-3,7-DIMETHYL-1,6-OCTAD

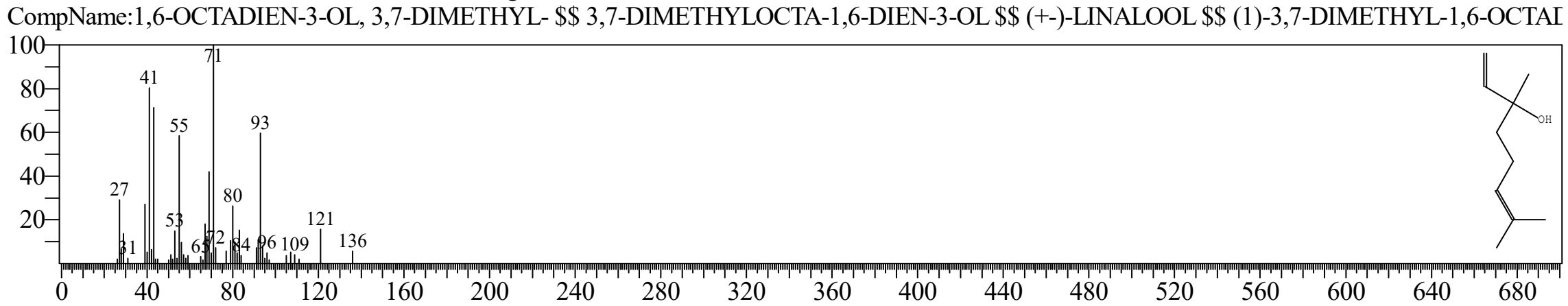

Hit#:3 Entry:47039 Library:WILEY8.LIB
SI:95 Formula:C10H18O CAS:78-70-6 MolWeight:154 RetIndex:0
CompName:1,6-OCTADIEN-3-OL, 3,7-DIMETHYL- $$ 3,7-DIMETHYLOCTA-1,6-DIEN-3-OL $$ (+-)-LINALOOL $$ (1)-3,7-DIMETHYL-1,6-OCTAD

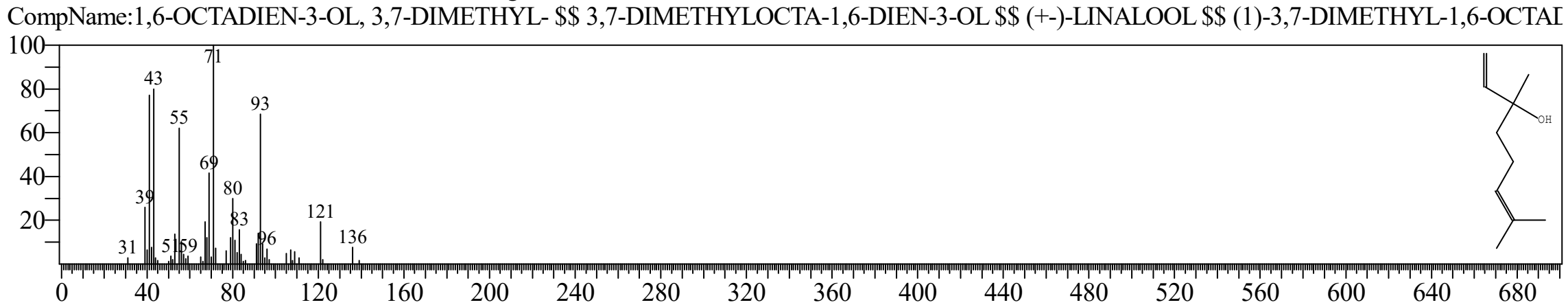

<< Target >>
Line#:29   R.Time:7.595(Scan#:820) MassPeaks:45
RawMode:Averaged 7.590-7.600(819-821) BasePeak:43.00(156220)
BG Mode:Calc. from Peak Group 1 - Event 1 Scan

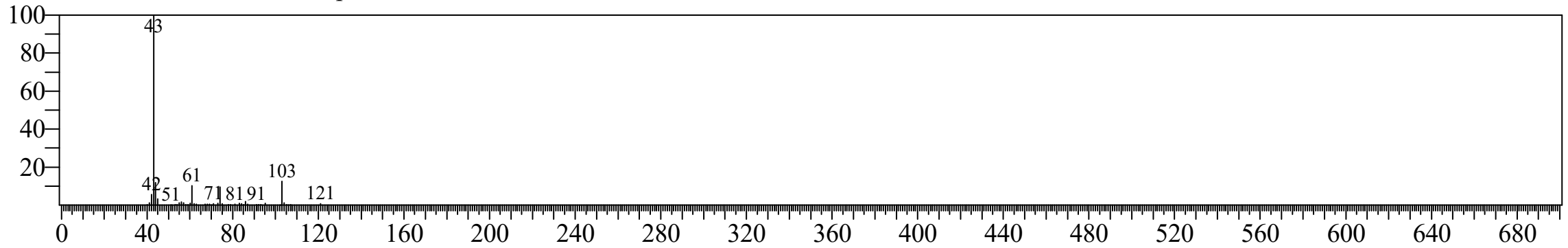

Hit#:1 Entry:6207 Library:NIST14s.lib
SI:95 Formula:C5H10O4 CAS:106-61-6 MolWeight:134 RetIndex:1091
CompName:1,2,3-Propanetriol, 1-acetate $$ Acetin, 1-mono- $$ .alpha.-Monoacetin $$ Glycerol .alpha.-monoacetate $$ 1-Monoacetin $$ 2,3-Dihydroxypro

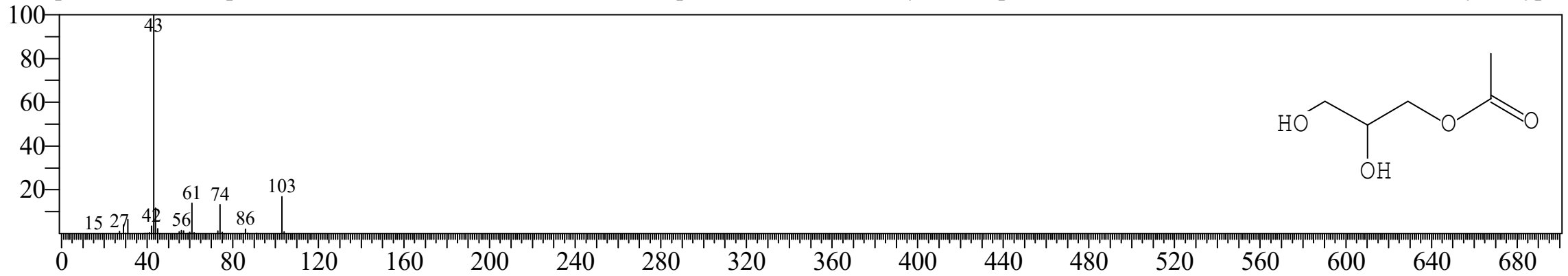

Hit#:2 Entry:6206 Library:NIST14s.lib
SI:94 Formula:C5H10O4 CAS:106-61-6 MolWeight:134 RetIndex:1091
CompName:1,2,3-Propanetriol, 1-acetate $$ Acetin, 1-mono- $$ .alpha.-Monoacetin $$ Glycerol .alpha.-monoacetate $$ 1-Monoacetin $$ 2,3-Dihydroxypro

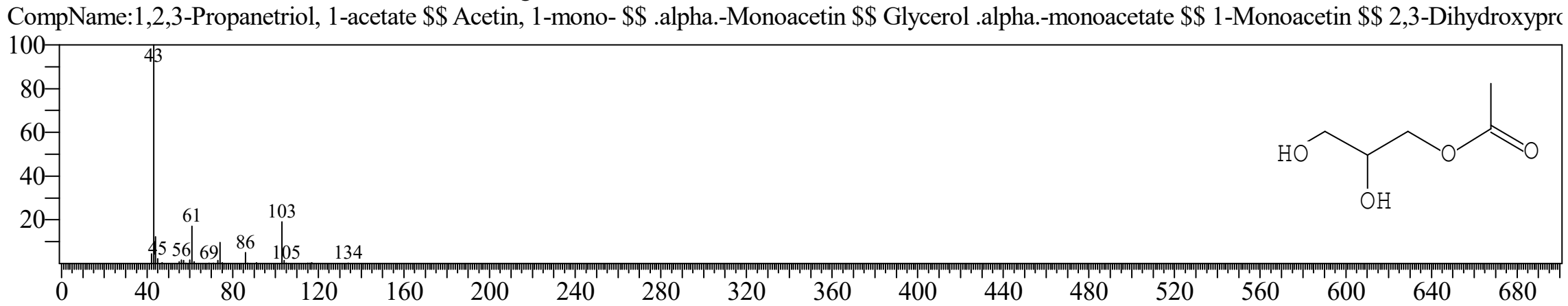

Hit#:3 Entry:25607 Library:WILEY8.LIB
SI:92 Formula:C5H10O4 CAS:26446-35-5 MolWeight:134 RetIndex:0
CompName:2,3-DIHYDROXYPROPYL ACETATE $$ 2,3-DIHYDROXYPROPYL ACETATE # $$ 1,2,3-PROPANETRIOL, MONOACETATE $$ 1-MO

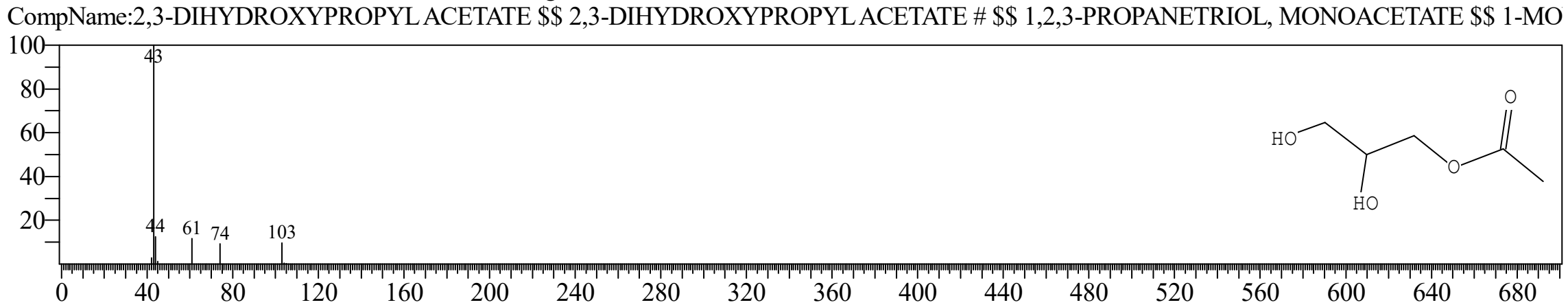

<< Target >>
Line#:30   R.Time:7.695(Scan#:840) MassPeaks:42
RawMode:Averaged 7.690-7.700(839-841) BasePeak:81.05(3839)
BG Mode:Calc. from Peak Group 1 - Event 1 Scan

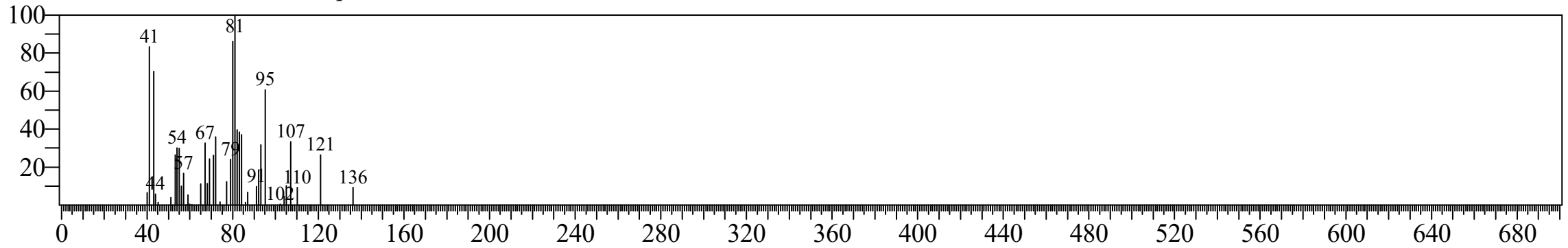

Hit#:1 Entry:18081 Library:NIST14.lib
SI:85 Formula:C10H18O CAS:2217-02-9 MolWeight:154 RetIndex:1138
CompName:Bicyclo[2.2.1]heptan-2-ol, 1,3,3-trimethyl-, (1R-endo)- $$ (1R)-endo-(+)-Fenchyl alcohol $$ 1,3,3-Trimethylbicyclo[2.2.1]heptan-2-ol # $$

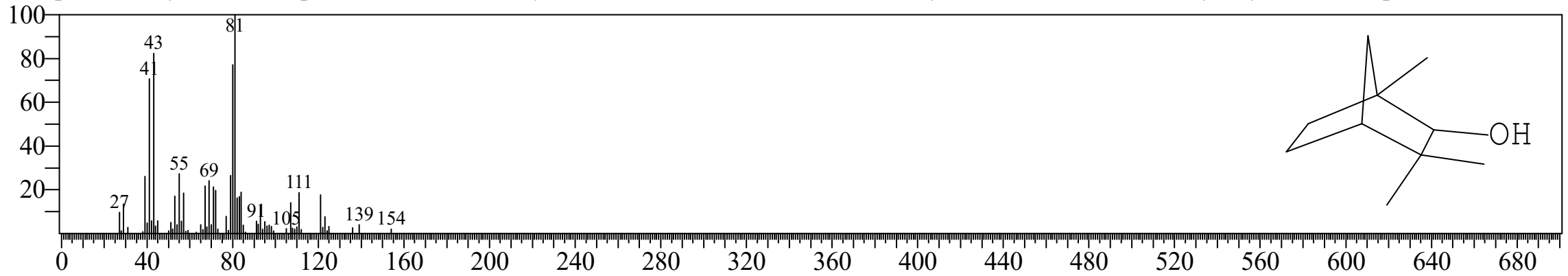

Hit#:2 Entry:18082 Library:NIST14.lib
SI:85 Formula:C10H18O CAS:22627-95-8 MolWeight:154 RetIndex:1138
CompName:Fenchol, exo- $$ exo-Fenchol $$ 1,3,3-Trimethylbicyclo[2.2.1]heptan-2-ol, exo $$ .beta.-Fenchol $$ .beta.-Fenchyl alcohol $$

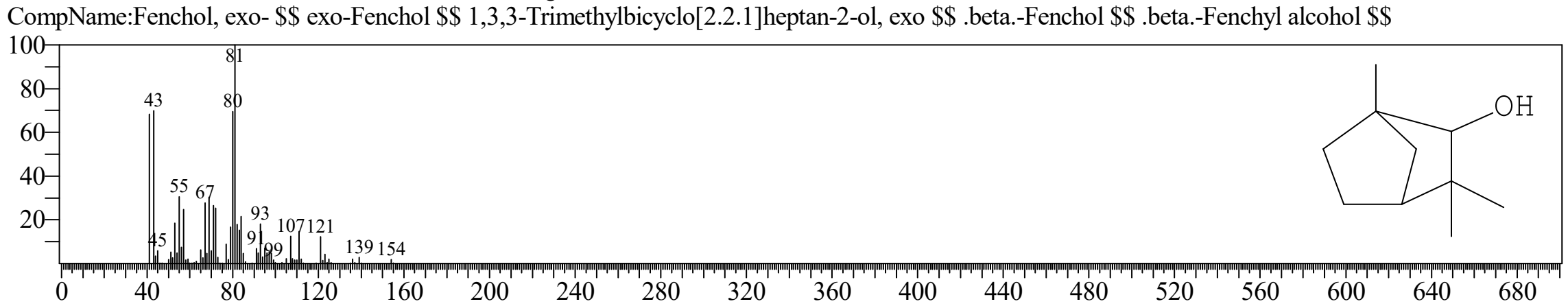

Hit#:3 Entry:47074 Library:WILEY8.LIB
SI:83 Formula:C10H18O CAS:3588-18-9 MolWeight:154 RetIndex:0
CompName:(5E)-2,6-DIMETHYL-5,7-OCTADIEN-4-ONE # $$ (5E)-2,6-DIMETHYL-5,7-OCTADIEN-4-ONE $$ (5E)-2,6-DIMETHYL-5,7-OCTADIEN

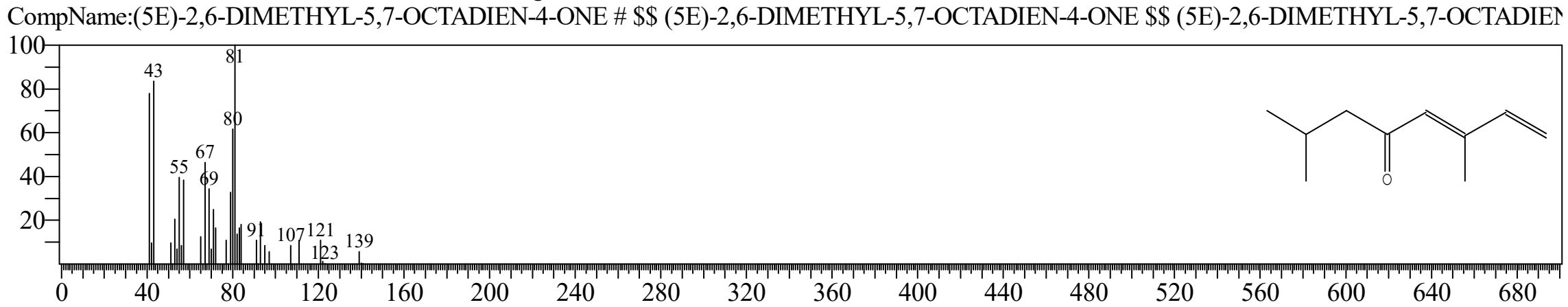

<< Target >>
Line#:31   R.Time:7.800(Scan#:861) MassPeaks:54
RawMode:Averaged 7.795-7.805(860-862) BasePeak:95.10(19610)
BG Mode:Calc. from Peak Group 1 - Event 1 Scan

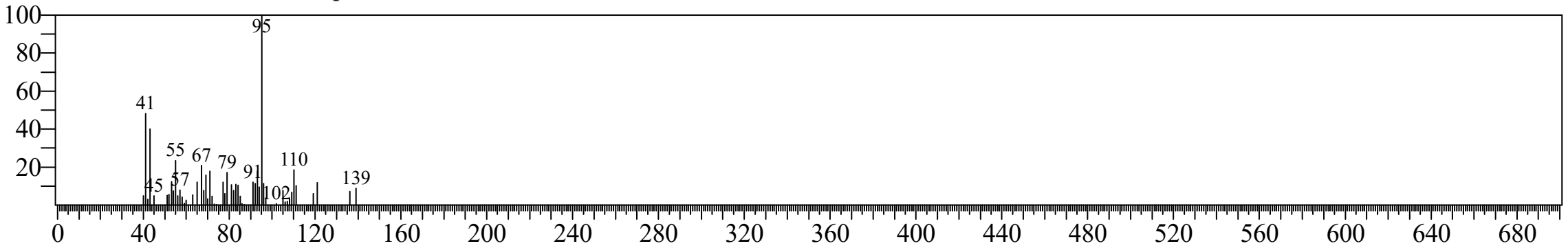

Hit#:1 Entry:46892 Library:WILEY8.LIB
SI:89 Formula:C10H18O CAS:464-45-9 MolWeight:154 RetIndex:0
CompName:BICYCLO[2.2.1]HEPTAN-2-OL, 1,7,7-TRIMETHYL-, (1S-ENDO)- $$ 1,7,7-TRIMETHYLBICYCLO[2.2.1]HEPTAN-2-OL # $$ ((1S)-ENI

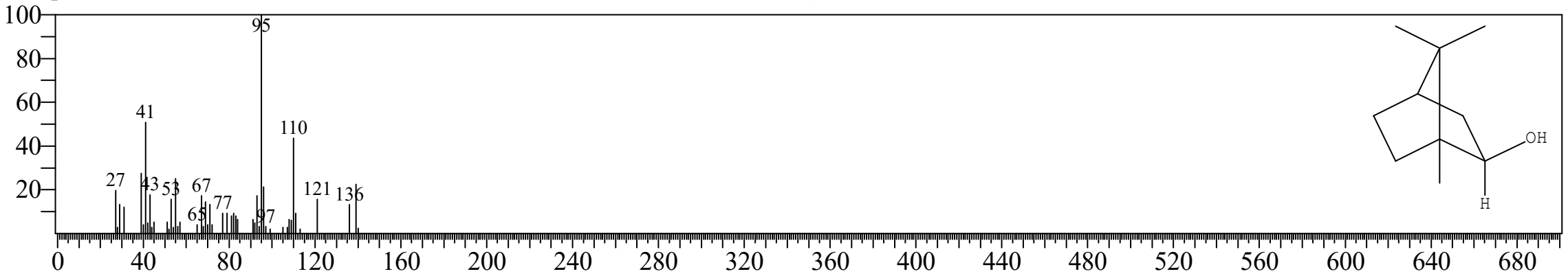

Hit#:2 Entry:46908 Library:WILEY8.LIB
SI:87 Formula:C10H18O CAS:124-76-5 MolWeight:154 RetIndex:0
CompName:BICYCLO[2.2.1]HEPTAN-2-OL, 1,7,7-TRIMETHYL-, EXO- $$ 1,7,7-TRIMETHYLBICYCLO[2.2.1]HEPTAN-2-OL # $$ 1,7,7-TRIMETH

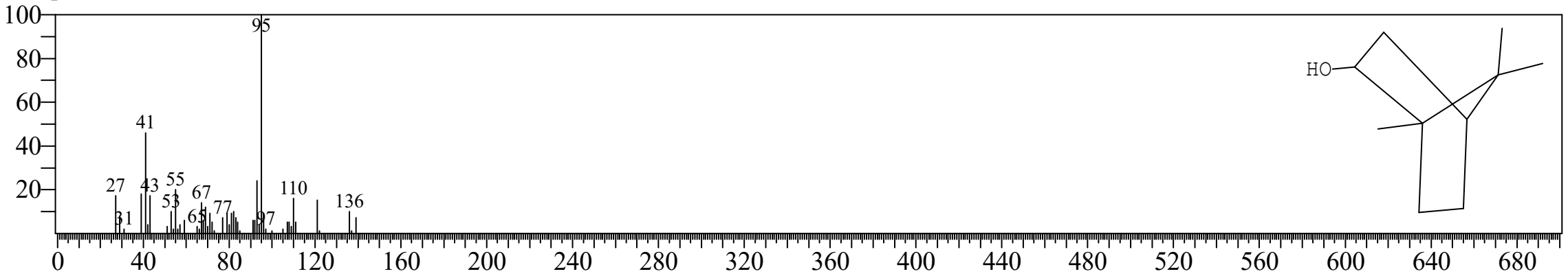

Hit#:3 Entry:10397 Library:NIST14s.lib
SI:87 Formula:C10H18O CAS:124-76-5 MolWeight:154 RetIndex:1138
CompName:Isoborneol $$ Bicyclo[2.2.1]heptan-2-ol, 1,7,7-trimethyl-, exo- $$ exo-2-Hydroxy-1,7,7-trimethylnorbornane $$ Isobornyl alcohol $$ exo-1,7,7

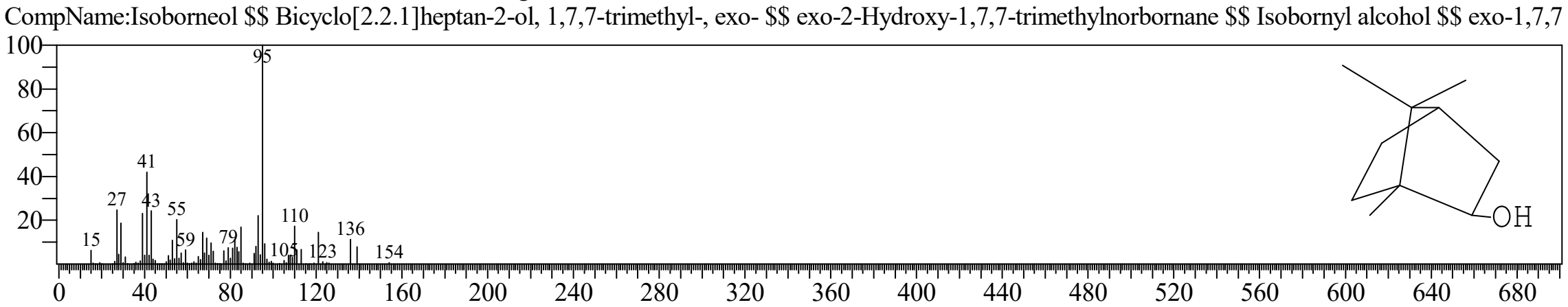

<< Target >>
Line#:32   R.Time:8.000(Scan#:901) MassPeaks:80
RawMode:Averaged 7.995-8.005(900-902) BasePeak:71.05(277725)
BG Mode:Calc. from Peak Group 1 - Event 1 Scan

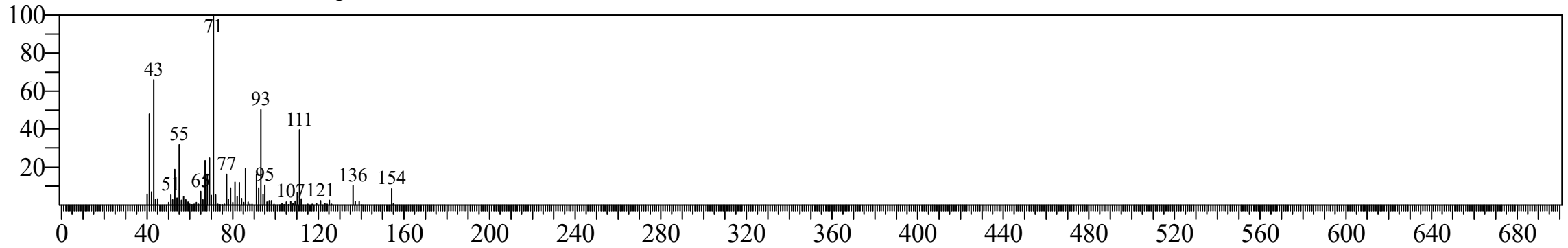

Hit#:1 Entry:46671 Library:WILEY8.LIB
SI:97 Formula:C10H18O CAS:562-74-3 MolWeight:154 RetIndex:0
CompName:3-CYCLOHEXEN-1-OL, 4-METHYL-1-(1-METHYLETHYL)- $$ 1-ISOPROPYL-4-METHYL-3-CYCLOHEXEN-1-OL # $$ (+-)-P-MENTI

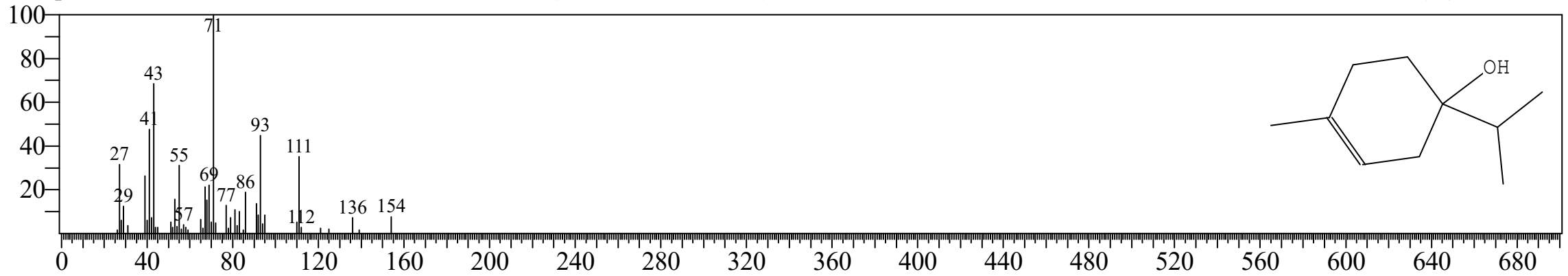

Hit#:2 Entry:47344 Library:WILEY8.LIB
SI:95 Formula:C10H18O CAS:0-00-0 MolWeight:154 RetIndex:0
CompName:1-ISOPROPYL-4-METHYL-3-CYCLOHEXEN-1-OL $$ 4-TERPINEOL

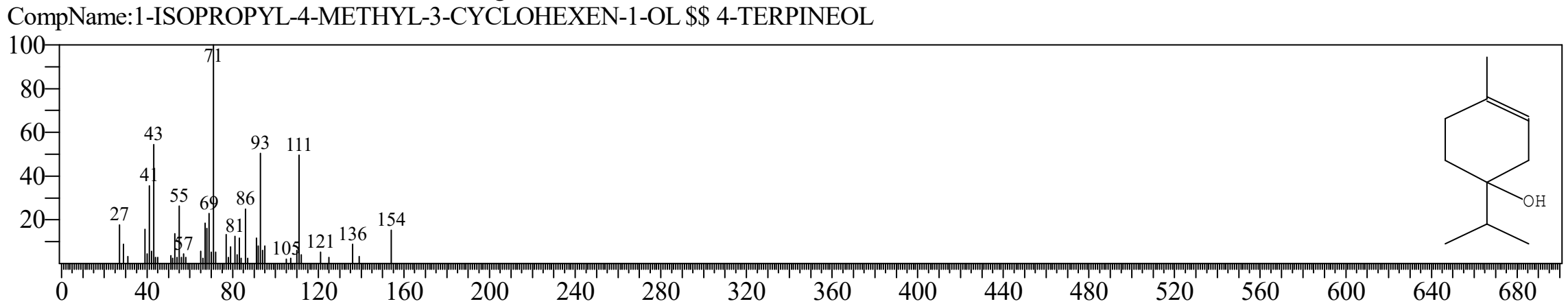

Hit#:3 Entry:10355 Library:NIST14s.lib
SI:95 Formula:C10H18O CAS:562-74-3 MolWeight:154 RetIndex:1137
CompName:Terpinen-4-ol $$ 3-Cyclohexen-1-ol, 4-methyl-1-(1-methylethyl)- $$ p-Menth-1-en-4-ol $$ 1-Terpinen-4-ol $$ 4-Carvomenthenol $$ 4-Terpinec

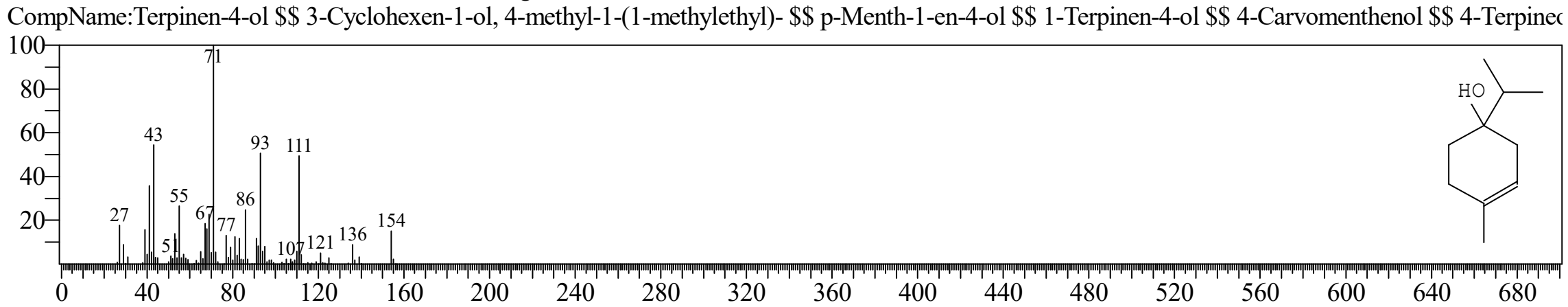

<< Target >>
Line#:33 R.Time:8.080(Scan#:917) MassPeaks:46
RawMode:Averaged 8.075-8.085(916-918) BasePeak:43.00(6381)
BG Mode:Calc. from Peak Group 1 - Event 1 Scan

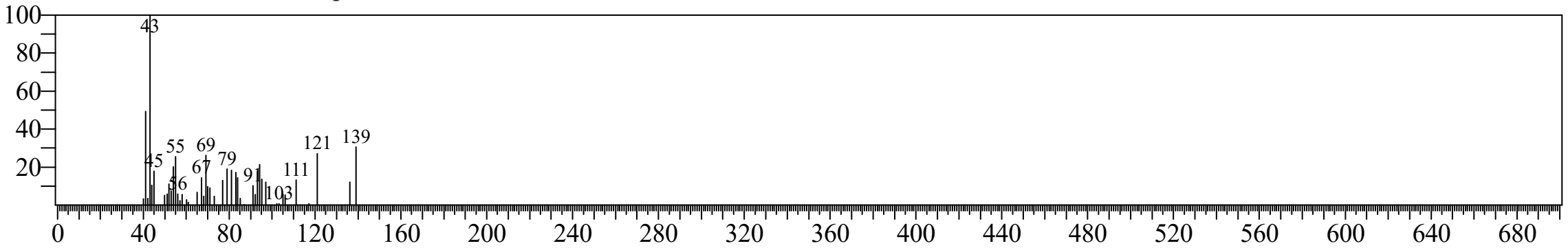

Hit#:1 Entry:17972 Library:NIST14.lib
SI:85 Formula:C10H18O CAS:619-62-5 MolWeight:154 RetIndex:0
CompName:4-Isopropyl-1-methylcyclohex-2-enol $$ p-Menth-2-en-1-ol $$ 2-p-Menthen-1-ol $$

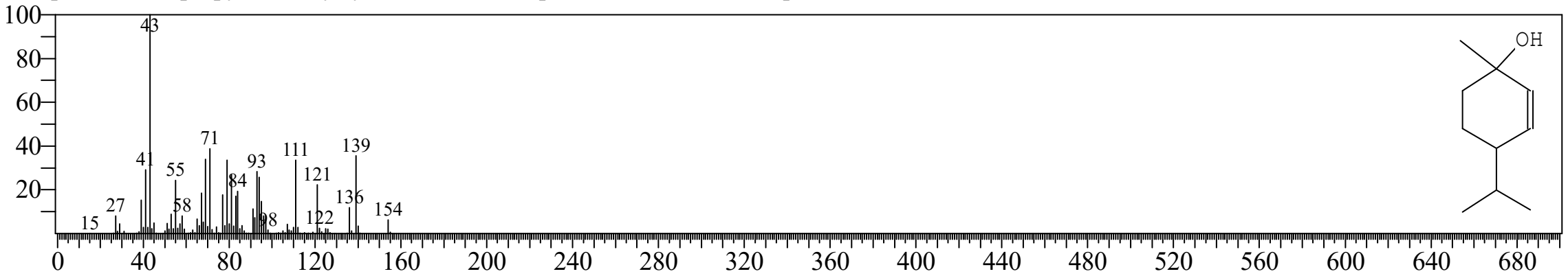

Hit#:2 Entry:47050 Library:WILEY8.LIB
SI:83 Formula:C10H18O CAS:35408-04-9 MolWeight:154 RetIndex:0
CompName:TRANS-PINENE HYDRATE

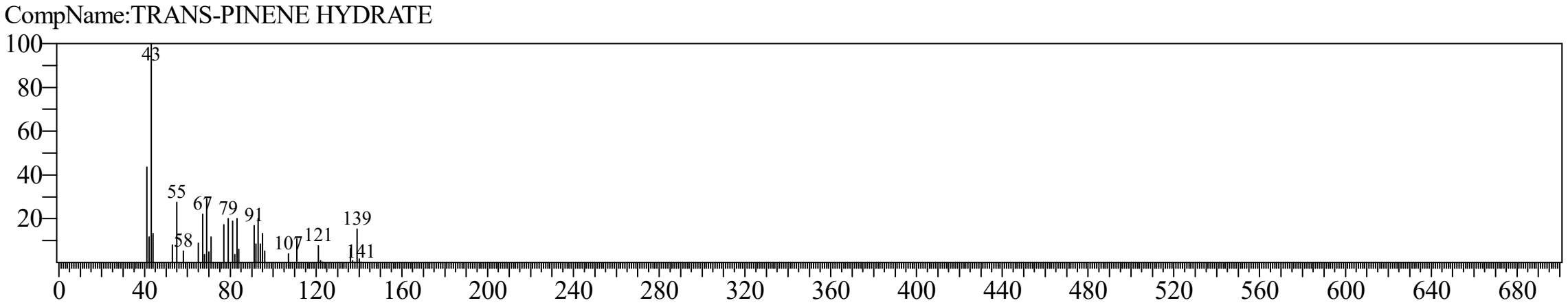

Hit#:3 Entry:47051 Library:WILEY8.LIB
SI:83 Formula:C10H18O CAS:35519-42-7 MolWeight:154 RetIndex:0
CompName:CIS-PINENE HYDRATE

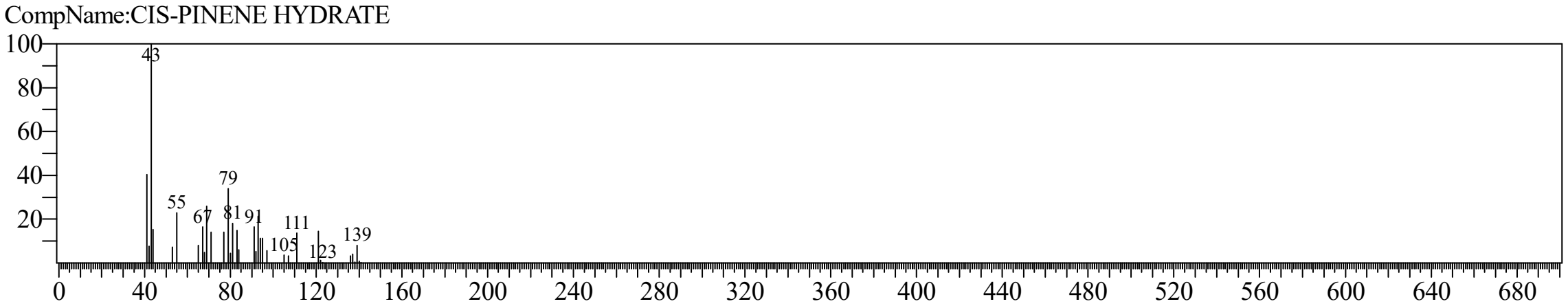

<< Target >>
Line#:34 R.Time:8.190(Scan#:939) MassPeaks:73
RawMode:Averaged 8.185-8.195(938-940) BasePeak:43.05(84419)
BG Mode:Calc. from Peak Group 1 - Event 1 Scan

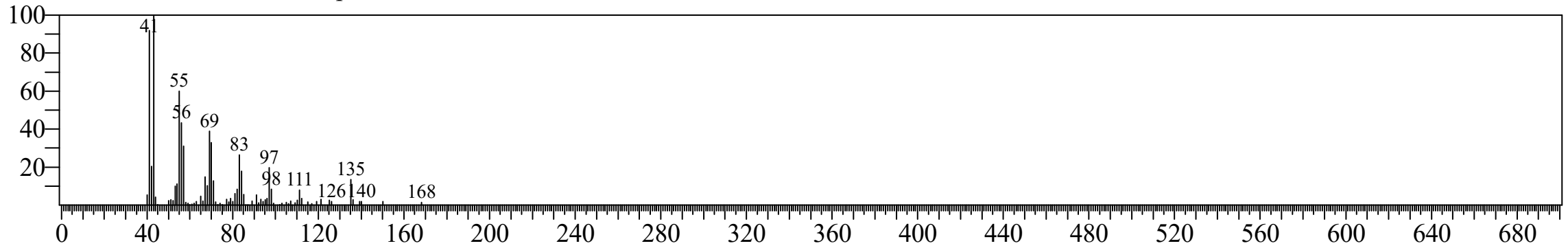

Hit#:1 Entry:12980 Library:NIST14s.lib
SI:92 Formula:C12H24 CAS:112-41-4 MolWeight:168 RetIndex:1204
CompName:1-Dodecene $$ .alpha.-Dodecene $$ n-Dodec-1-ene $$ Adacene 12 $$ Dodec-1-ene $$ .alpha.-Dodecylene $$ Dodecylene .alpha.- $$ Dodecen

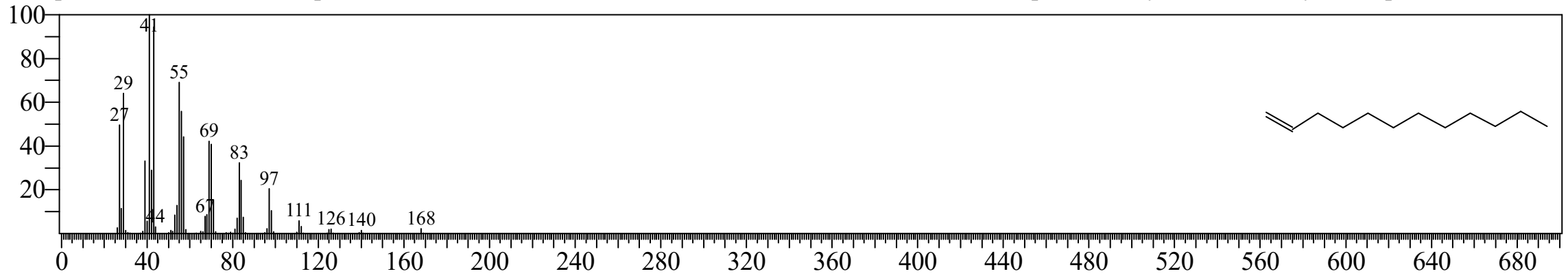

Hit#:2 Entry:63399 Library:WILEY8.LIB
SI:92 Formula:C12H24 CAS:112-41-4 MolWeight:168 RetIndex:0
CompName:1-DODECENE $$ DODEC-1-ENE $$ .ALPHA.-DODECENE $$ .ALPHA.-DODECYLENE $$ ADACENE 12 $$ ALPHA-DODECENE $$ A

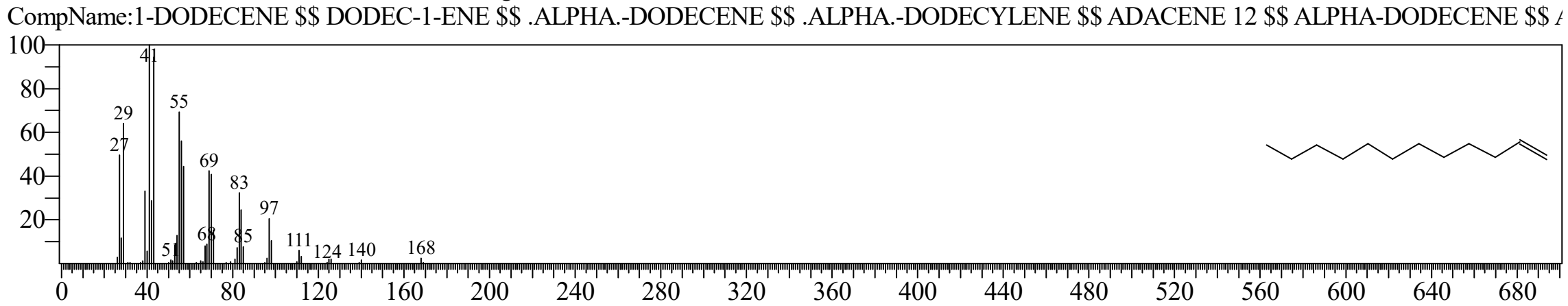

Hit#:3 Entry:34797 Library:NIST14.lib
SI:91 Formula:C13H26 CAS:41446-57-5 MolWeight:182 RetIndex:1321
CompName:3-Tridecene, (E)- $$ (3E)-3-Tridecene $$ 3-Tridecene, (E) $$ (E)-3-Tridecene $$ trans-3-Tridecene $$

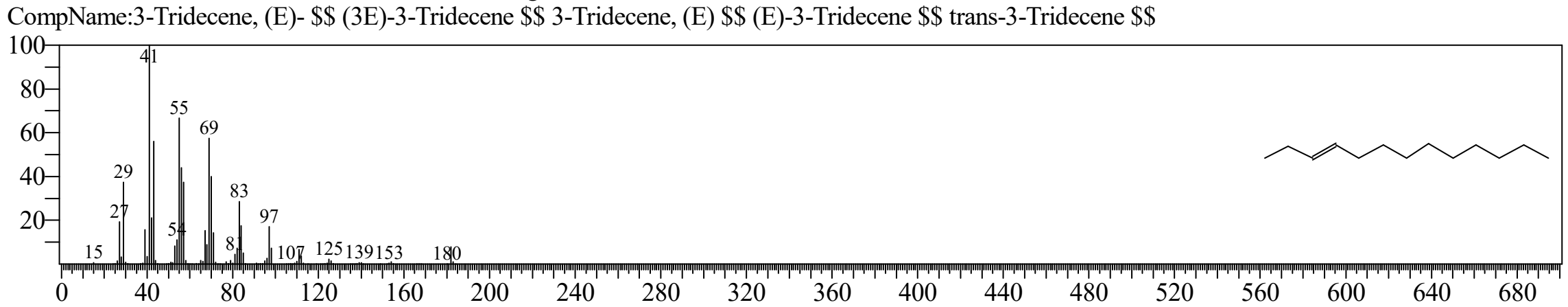

<< Target >>
Line#:35 R.Time:8.270(Scan#:955) MassPeaks:55
RawMode:Averaged 8.265-8.275(954-956) BasePeak:59.05(65877)
BG Mode:Calc. from Peak Group 1 - Event 1 Scan

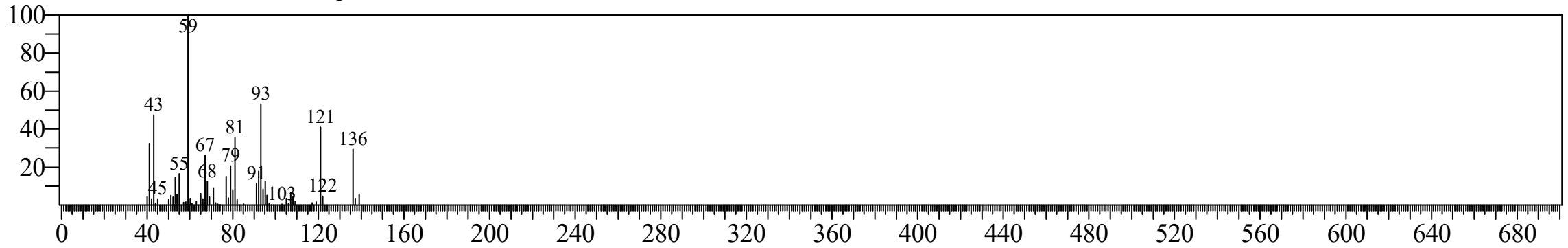

Hit#:1 Entry:18020 Library:NIST14.lib
SI:97 Formula:C10H18O CAS:0-00-0 MolWeight:154 RetIndex:0
CompName:Terpineol

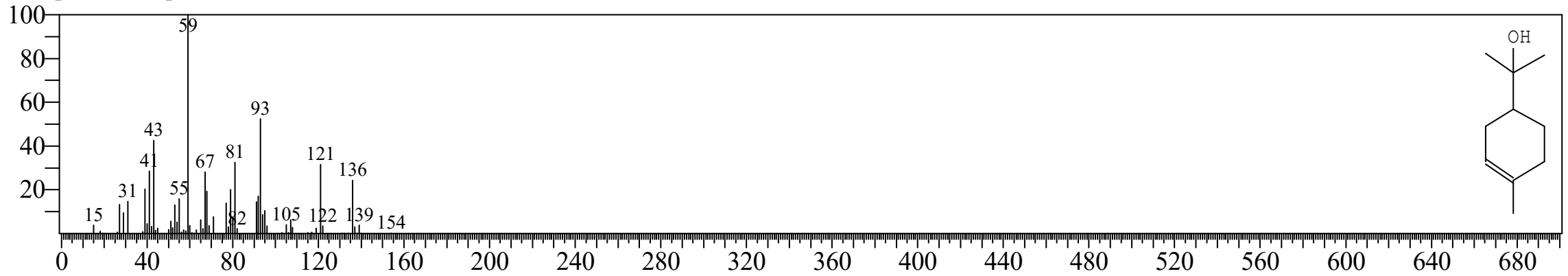

Hit#:2 Entry:47343 Library:WILEY8.LIB
SI:96 Formula:C10H18O CAS:0-00-0 MolWeight:154 RetIndex:0
CompName:(+)-ALPHA-TERPINEOL (P-MENTH-1-EN-8-OL) $$ 2-(4-METHYL-3-CYCLOHEXEN-1-YL)-2-PROPANOL

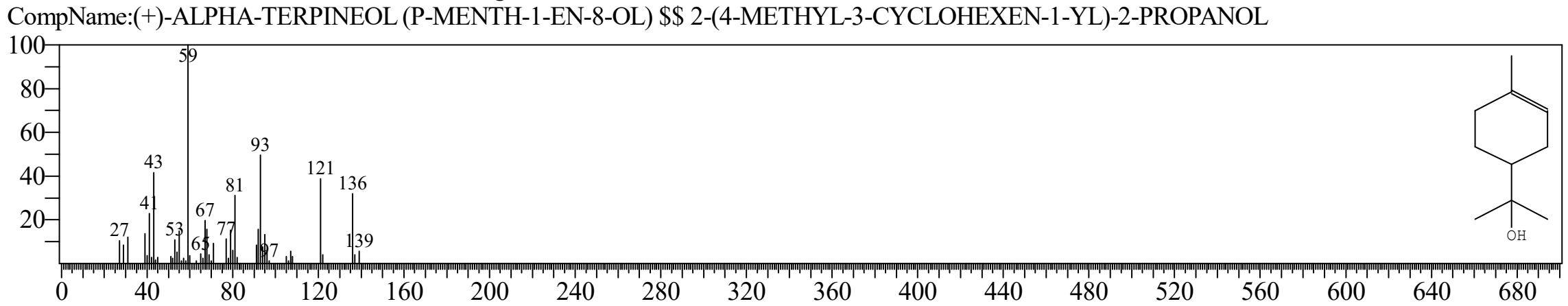

Hit#:3 Entry:46690 Library:WILEY8.LIB
SI:96 Formula:C10H18O CAS:98-55-5 MolWeight:154 RetIndex:0
CompName:3-CYCLOHEXENE-1-METHANOL, .ALPHA.,.ALPHA.,4-TRIMETHYL- $$ 3-CYCLOHEXENE-1-METHANOL, .ALPHA.,.ALPHA.,4-TR

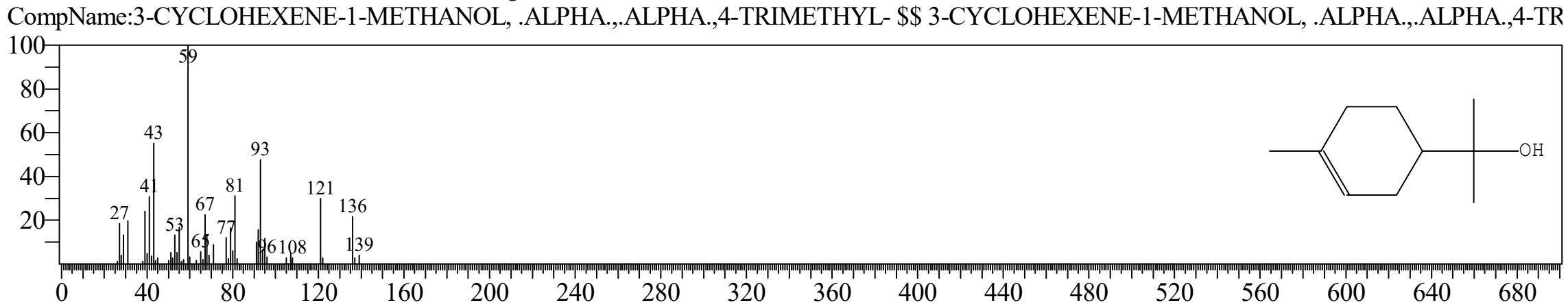

<< Target >>
Line#:36   R.Time:8.340(Scan#:969) MassPeaks:40
RawMode:Averaged 8.335-8.345(968-970) BasePeak:43.00(25118)
BG Mode:Calc. from Peak Group 1 - Event 1 Scan

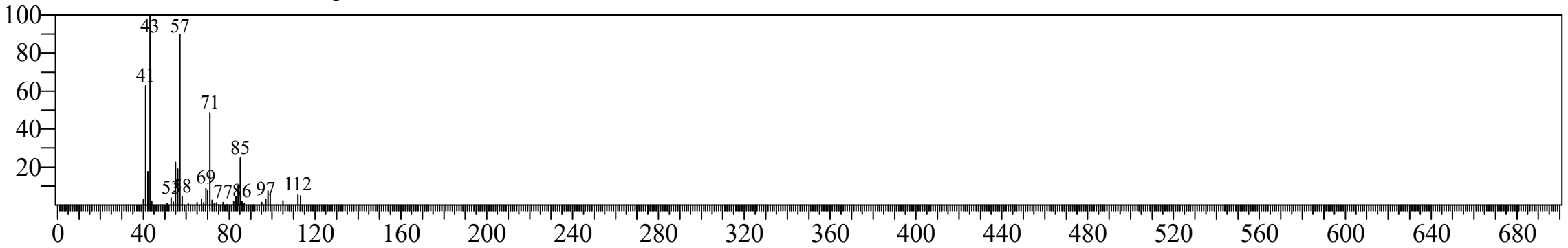

Hit#:1 Entry:102055 Library:WILEY8.LIB
SI:95 Formula:C14H30 CAS:629-59-4 MolWeight:198 RetIndex:0
CompName:TETRADECANE $$ AI3-04240 $$ BRN 1733859 $$ CCRIS 715 $$ EINECS 211-096-0 $$ HSDB 5728 $$ ISOTETRADECANE $$ N-TET

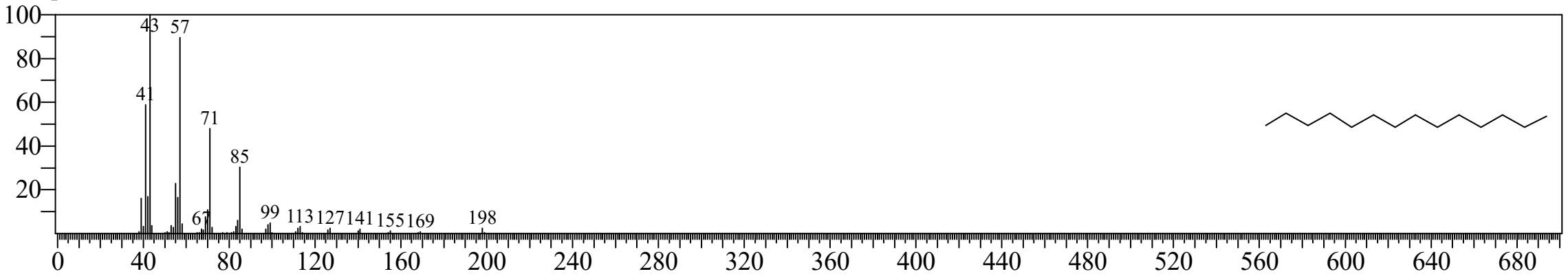

Hit#:2 Entry:83387 Library:WILEY8.LIB
SI:95 Formula:C13H28 CAS:629-50-5 MolWeight:184 RetIndex:0
CompName:TRIDECANE $$ EINECS 211-093-4 $$ HSDB 5727 $$ N-TRIDECANE $$ NSC 66205 $$ TRIDECAN $$ TRIDECANE, N-

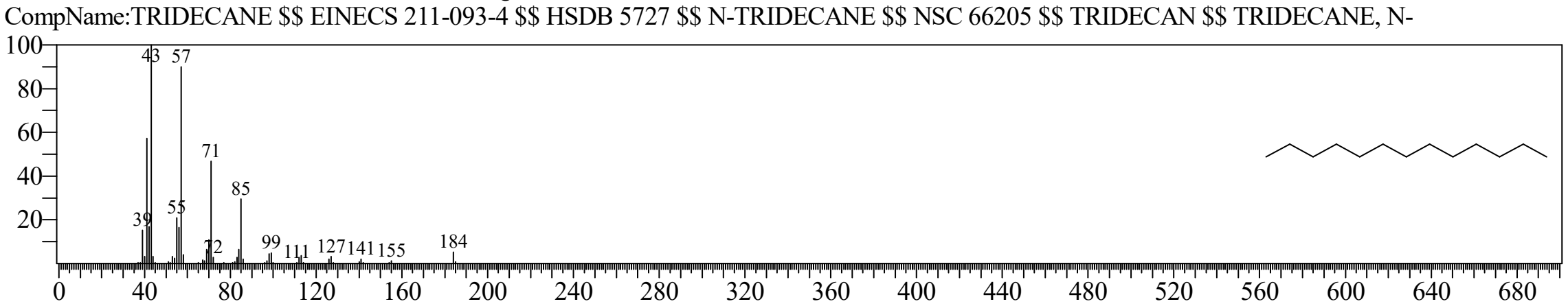

Hit#:3 Entry:142363 Library:WILEY8.LIB
SI:95 Formula:C16H34 CAS:544-76-3 MolWeight:226 RetIndex:0
CompName:HEXADECANE $$ AI3-06522 $$ BRN 1736592 $$ CCRIS 5833 $$ CETANE $$ EINECS 208-878-9 $$ HEXADECAN $$ HSDB 6854 $$

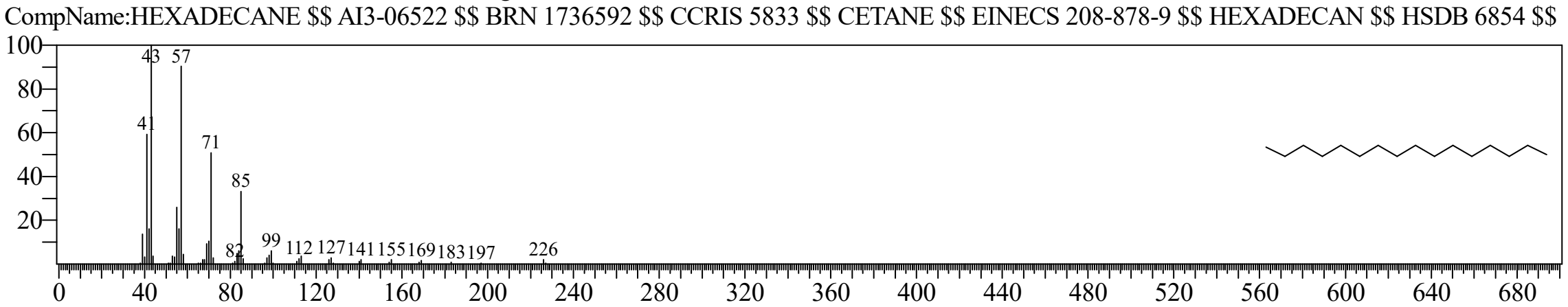

<< Target >>
Line#:37   R.Time:8.505(Scan#:1002) MassPeaks:50
RawMode:Averaged 8.500-8.510(1001-1003) BasePeak:95.10(20610)
BG Mode:Calc. from Peak Group 1 - Event 1 Scan

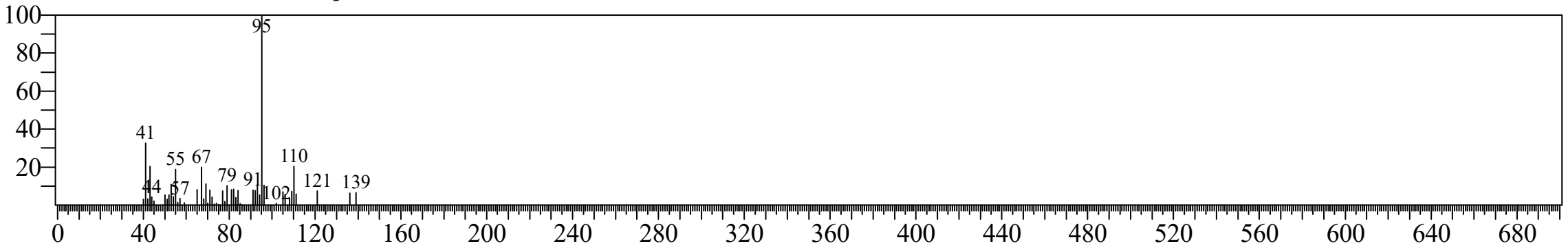

Hit#:1 Entry:10400 Library:NIST14s.lib
SI:92 Formula:C10H18O CAS:124-76-5 MolWeight:154 RetIndex:1138
CompName:Isoborneol $$ Bicyclo[2.2.1]heptan-2-ol, 1,7,7-trimethyl-, exo- $$ exo-2-Hydroxy-1,7,7-trimethylnorbornane $$ Isobornyl alcohol $$ exo-1,7,7

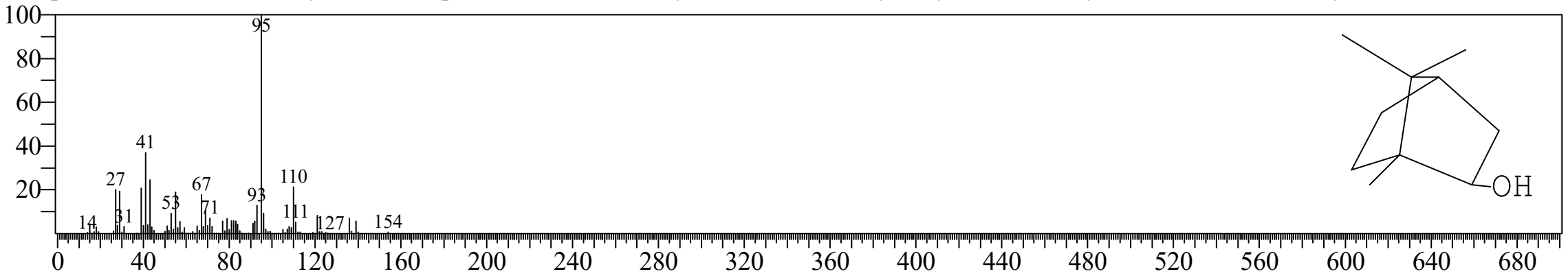

Hit#:2 Entry:46890 Library:WILEY8.LIB
SI:91 Formula:C10H18O CAS:464-45-9 MolWeight:154 RetIndex:0
CompName:BICYCLO[2.2.1]HEPTAN-2-OL, 1,7,7-TRIMETHYL-, (1S-ENDO)- $$ 1,7,7-TRIMETHYLBICYCLO[2.2.1]HEPTAN-2-OL # $$ ((1S)-ENI

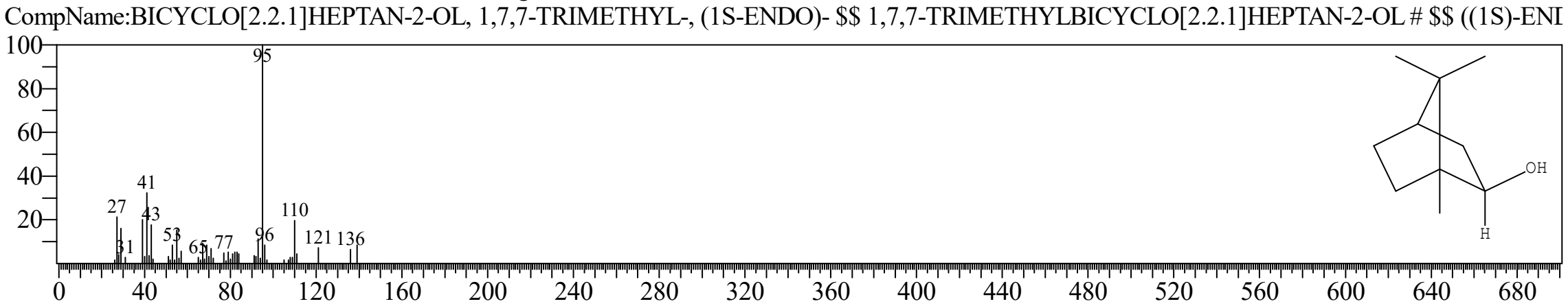

Hit#:3 Entry:10396 Library:NIST14s.lib
SI:91 Formula:C10H18O CAS:464-45-9 MolWeight:154 RetIndex:1138
CompName:Bicyclo[2.2.1]heptan-2-ol, 1,7,7-trimethyl-, (1S-endo)- $$ Borneol, (1S,2R,4S)-(-)- $$ (-)-Borneol $$ L-Borneol $$ Linderol $$ Ngai camphor

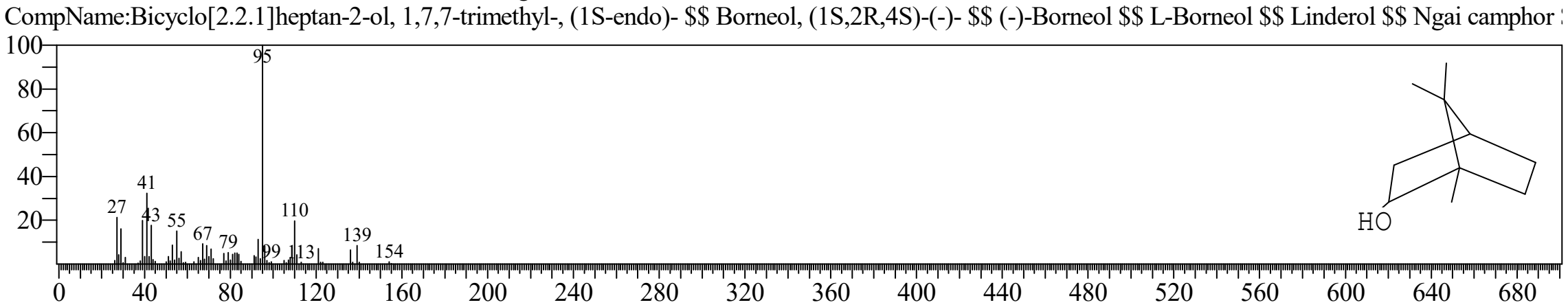

<< Target >>
Line#:38 R.Time:8.655(Scan#:1032) MassPeaks:77
RawMode:Averaged 8.650-8.660(1031-1033) BasePeak:71.05(245112)
BG Mode:Calc. from Peak Group 1 - Event 1 Scan

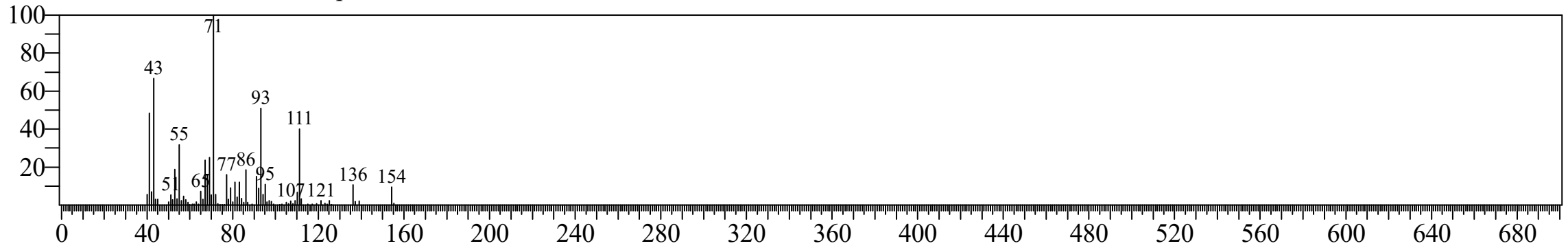

Hit#:1 Entry:46671 Library:WILEY8.LIB
SI:96 Formula:C10H18O CAS:562-74-3 MolWeight:154 RetIndex:0
CompName:3-CYCLOHEXEN-1-OL, 4-METHYL-1-(1-METHYLETHYL)- $$ 1-ISOPROPYL-4-METHYL-3-CYCLOHEXEN-1-OL # $$ (+-)-P-MENTI

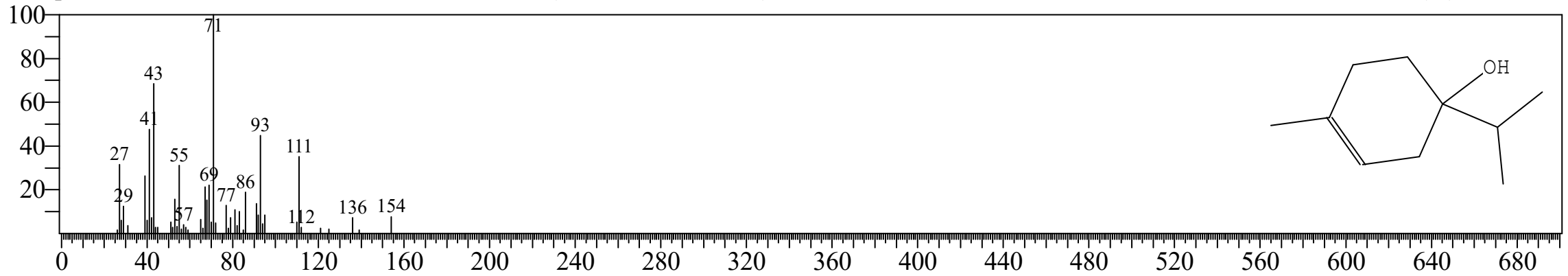

Hit#:2 Entry:47344 Library:WILEY8.LIB
SI:95 Formula:C10H18O CAS:0-00-0 MolWeight:154 RetIndex:0
CompName:1-ISOPROPYL-4-METHYL-3-CYCLOHEXEN-1-OL $$ 4-TERPINEOL

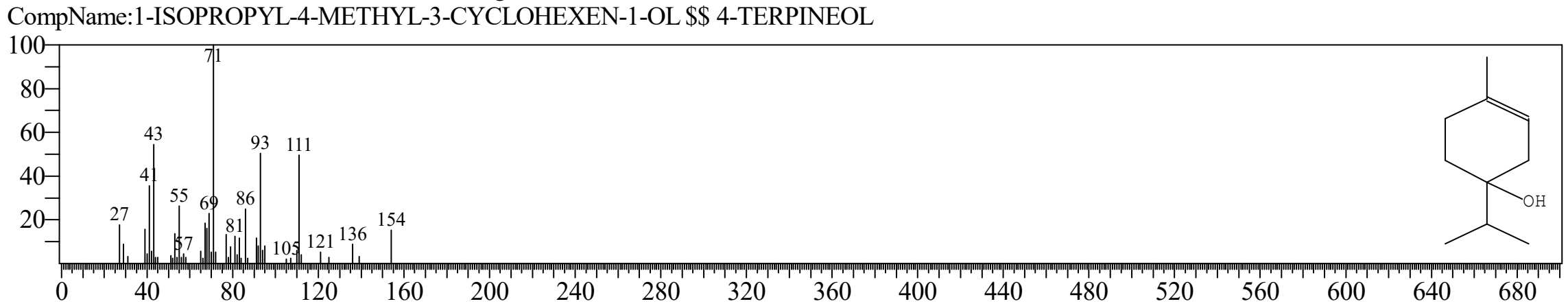

Hit#:3 Entry:10355 Library:NIST14s.lib
SI:95 Formula:C10H18O CAS:562-74-3 MolWeight:154 RetIndex:1137
CompName:Terpinen-4-ol $$ 3-Cyclohexen-1-ol, 4-methyl-1-(1-methylethyl)- $$ p-Menth-1-en-4-ol $$ 1-Terpinen-4-ol $$ 4-Carvomenthenol $$ 4-Terpinec

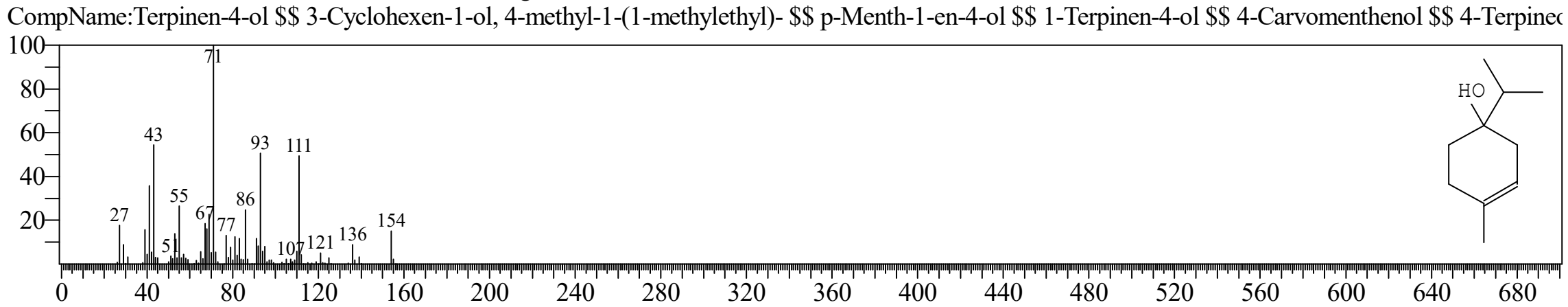

<< Target >>
Line#:39   R.Time:8.750(Scan#:1051) MassPeaks:55
RawMode:Averaged 8.745-8.755(1050-1052) BasePeak:41.05(64586)
BG Mode:Calc. from Peak Group 1 - Event 1 Scan

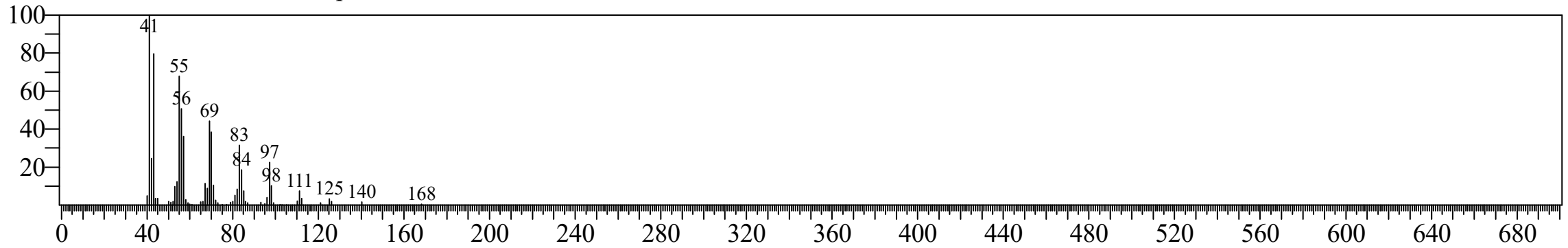

Hit#:1 Entry:12980 Library:NIST14s.lib
SI:96 Formula:C12H24 CAS:112-41-4 MolWeight:168 RetIndex:1204
CompName:1-Dodecene $$ .alpha.-Dodecene $$ n-Dodec-1-ene $$ Adacene 12 $$ Dodec-1-ene $$ .alpha.-Dodecylene $$ Dodecylene .alpha.- $$ Dodecen

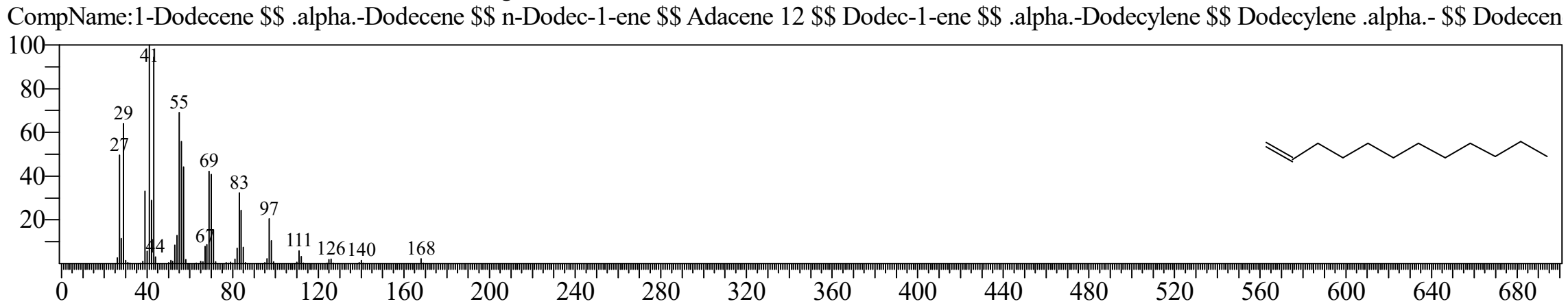

Hit#:2 Entry:63399 Library:WILEY8.LIB
SI:95 Formula:C12H24 CAS:112-41-4 MolWeight:168 RetIndex:0
CompName:1-DODECENE $$ DODEC-1-ENE $$ .ALPHA.-DODECENE $$ .ALPHA.-DODECYLENE $$ ADACENE 12 $$ ALPHA-DODECENE $$ A

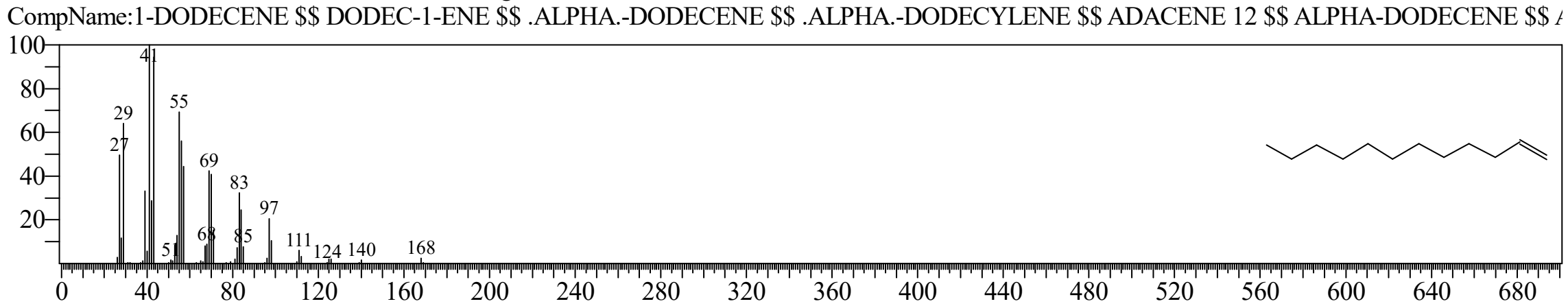

Hit#:3 Entry:99250 Library:WILEY8.LIB
SI:95 Formula:C14H28 CAS:1120-36-1 MolWeight:196 RetIndex:0
CompName:1-TETRADECENE $$ TETRADECANE $$ TETRADEC-1-ENE $$ .ALPHA.-TETRADECENE $$ 1-BUTADECENE $$ 1-TETRADECYL

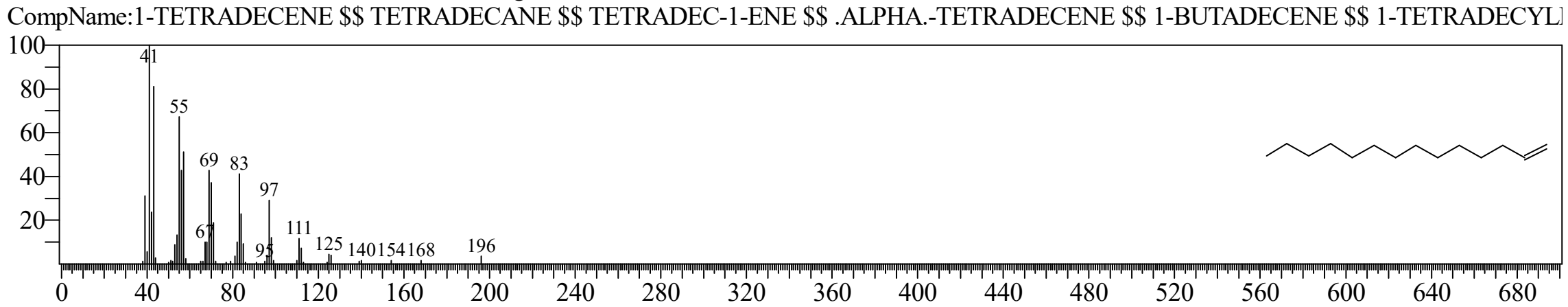

<< Target >>
Line#:40 R.Time:8.860(Scan#:1073) MassPeaks:68
RawMode:Averaged 8.855-8.865(1072-1074) BasePeak:43.00(61859)
BG Mode:Calc. from Peak Group 1 - Event 1 Scan

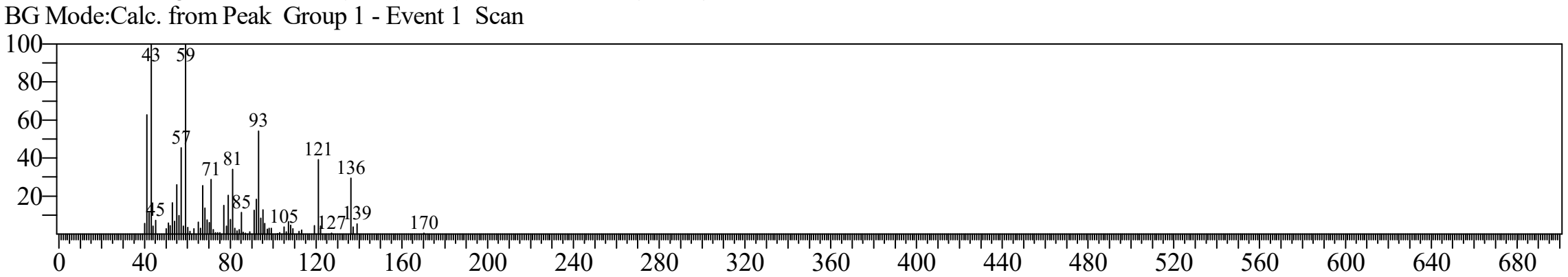

Hit#:1 Entry:46688 Library:WILEY8.LIB
SI:90 Formula:C10H18O CAS:98-55-5 MolWeight:154 RetIndex:0
CompName:3-CYCLOHEXENE-1-METHANOL, .ALPHA.,.ALPHA.,4-TRIMETHYL- $$ 3-CYCLOHEXENE-1-METHANOL, .ALPHA.,.ALPHA.,4-TR

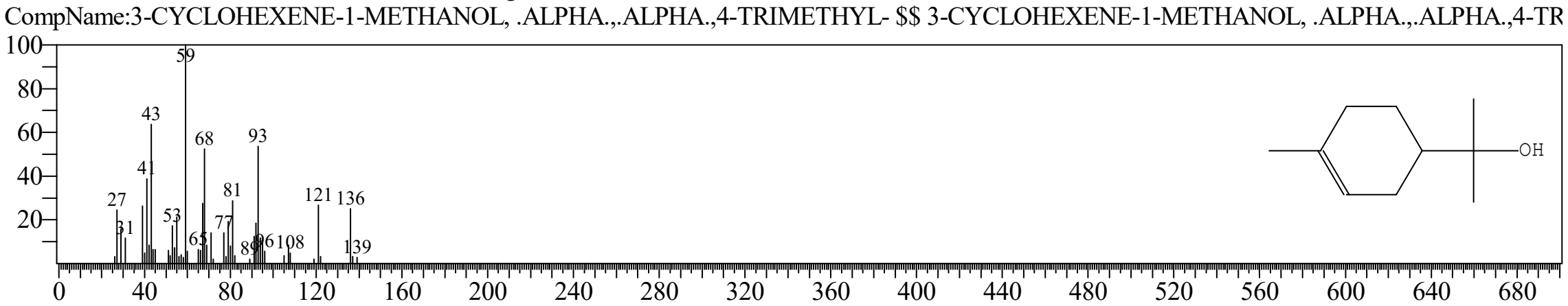

Hit#:2 Entry:46700 Library:WILEY8.LIB
SI:90 Formula:C10H18O CAS:10482-56-1 MolWeight:154 RetIndex:0
CompName:3-CYCLOHEXENE-1-METHANOL, .ALPHA.,.ALPHA.,4-TRIMETHYL-, (S)- $$ 2-(4-METHYL-3-CYCLOHEXEN-1-YL)-2-PROPANOL

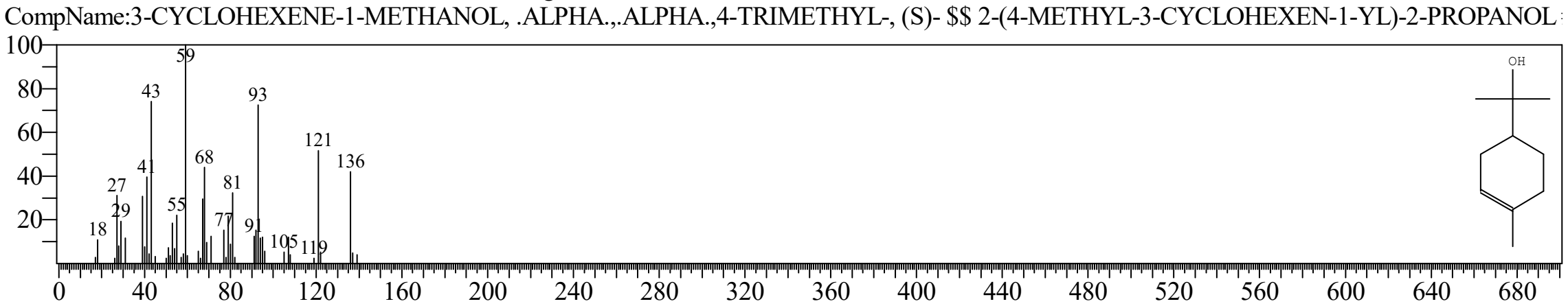

Hit#:3 Entry:18020 Library:NIST14.lib
SI:89 Formula:C10H18O CAS:0-00-0 MolWeight:154 RetIndex:0
CompName:Terpineol

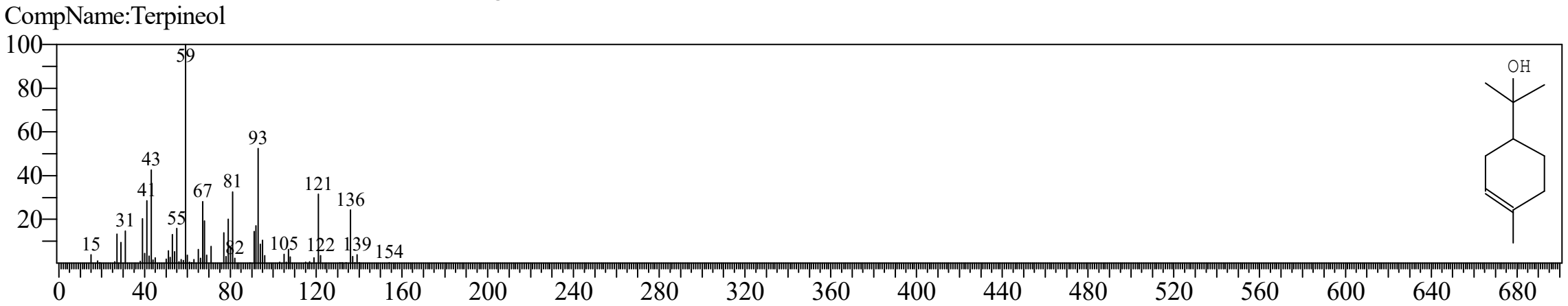

<< Target >>
Line#:41   R.Time:8.950(Scan#:1091) MassPeaks:39
RawMode:Averaged 8.945-8.955(1090-1092) BasePeak:43.05(7256)
BG Mode:Calc. from Peak Group 1 - Event 1 Scan

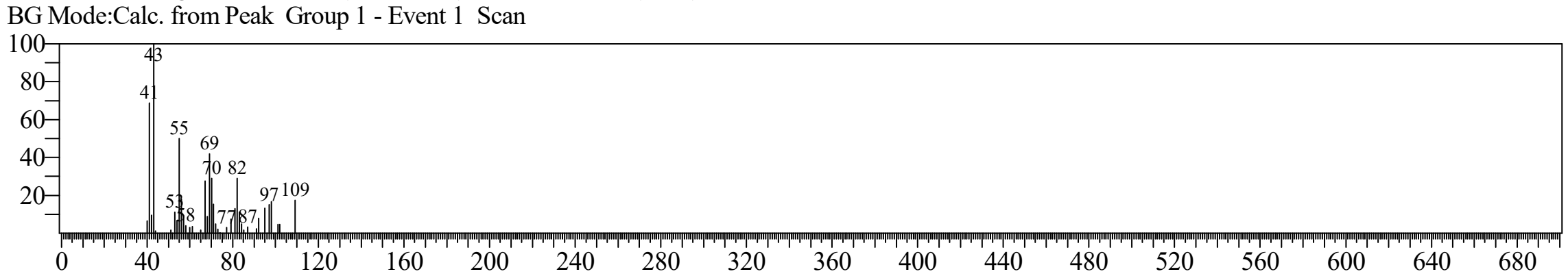

Hit#:1 Entry:6769 Library:NIST14.lib
SI:84 Formula:C9H18 CAS:124-11-8 MolWeight:126 RetIndex:906
CompName:1-Nonene $$ n-Non-1-ene $$ 1-C9H18 $$ NSC 73961 $$ Nonene-(1) $$ Nonylene $$ Non-1-ene $$

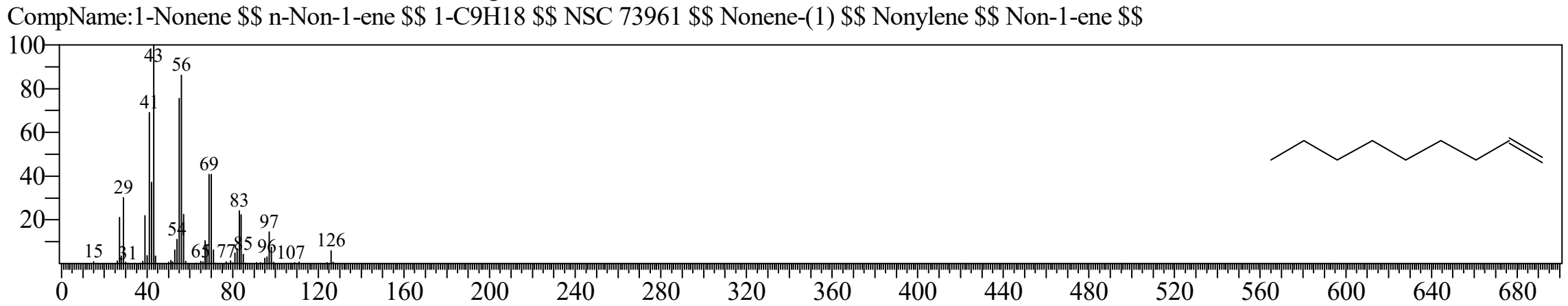

Hit#:2 Entry:297053 Library:WILEY8.LIB
SI:83 Formula:C23H44O2 CAS:56630-69-4 MolWeight:352 RetIndex:0
CompName:13-DOCOSENOIC ACID, METHYL ESTER $$ METHYL (13E)-13-DOCOSENOATE # $$ METHYL (13E)-13-DOCOSENOATE $$ METH

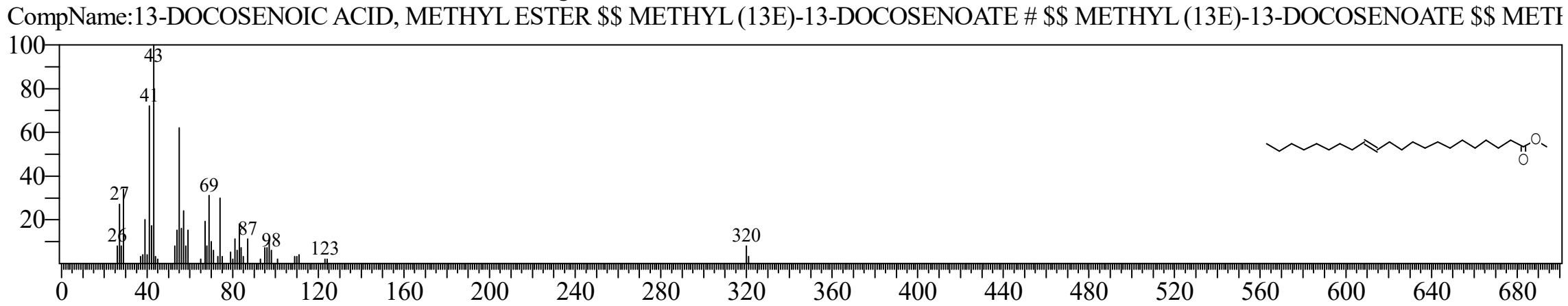

Hit#:3 Entry:176446 Library:NIST14.lib
SI:83 Formula:C23H44O2 CAS:56630-69-4 MolWeight:352 RetIndex:2483
CompName:13-Docosenoic acid, methyl ester $$ Methyl 13-docosenoate $$

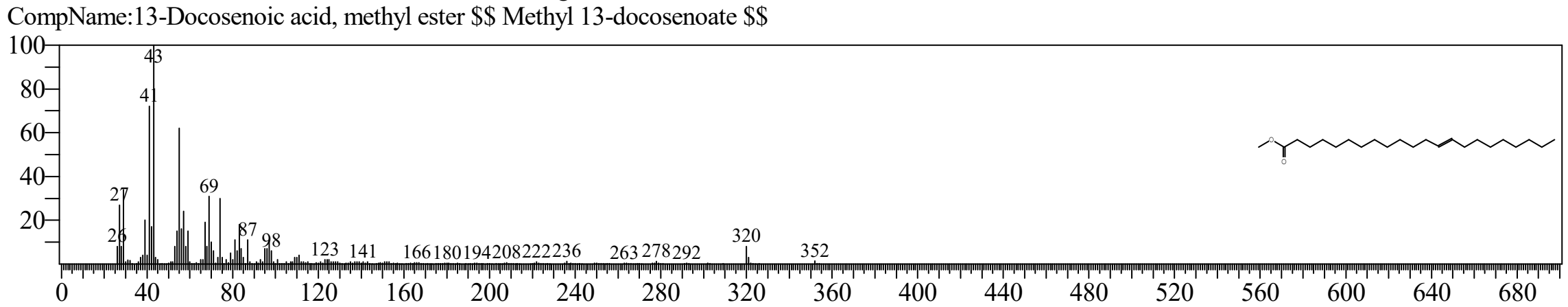

<< Target >>
Line#:42   R.Time:9.045(Scan#:1110) MassPeaks:35
RawMode:Averaged 9.040-9.050(1109-1111) BasePeak:91.05(1358)
BG Mode:Calc. from Peak Group 1 - Event 1 Scan

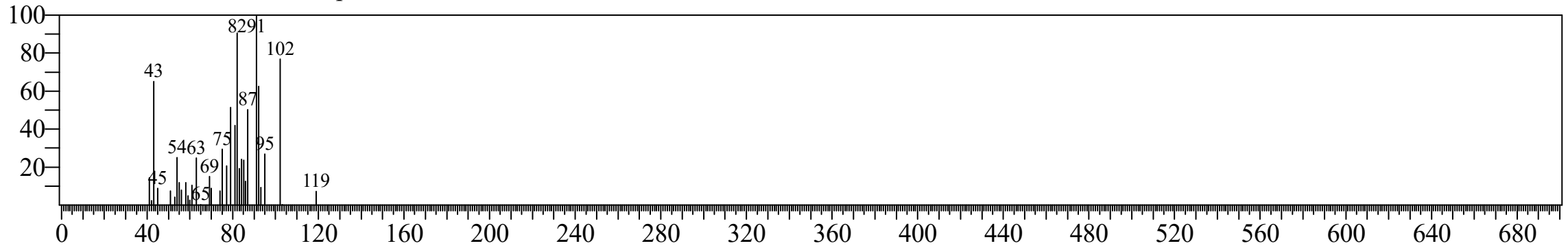

Hit#:1 Entry:43880 Library:WILEY8.LIB
SI:64 Formula:C10H16O CAS:3310-02-9 MolWeight:152 RetIndex:0
CompName:BICYCLO[3.1.0]HEXAN-3-OL, 4-METHYLENE-1-(1-METHYLETHYL)-, (1.ALPHA.,3.ALPHA.,5.ALPHA.)- $$ 1-ISOPROPYL-4-METH

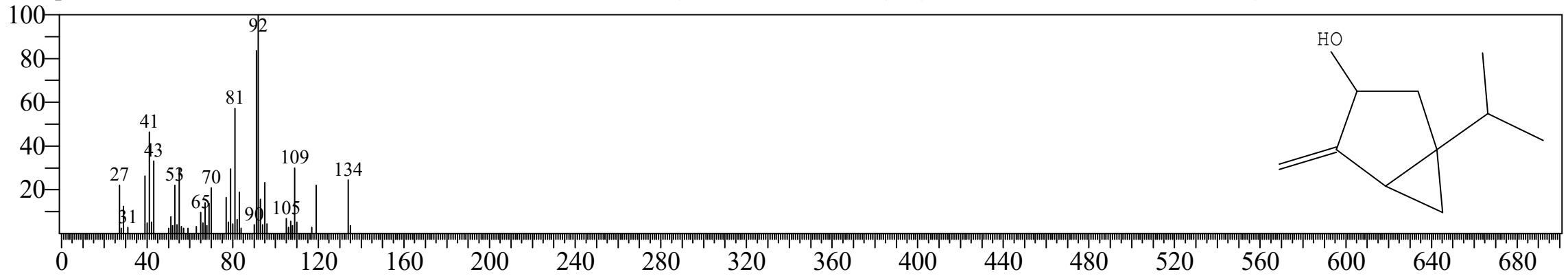

Hit#:2 Entry:16879 Library:NIST14.lib
SI:64 Formula:C10H16O CAS:3310-02-9 MolWeight:152 RetIndex:1085
CompName:Bicyclo[3.1.0]hexan-3-ol, 4-methylene-1-(1-methylethyl)-, (1.alpha.,3.alpha.,5.alpha.)- $$ (1R,3R,5R)-1-Isopropyl-4-methylenebicyclo[3.1.0]he

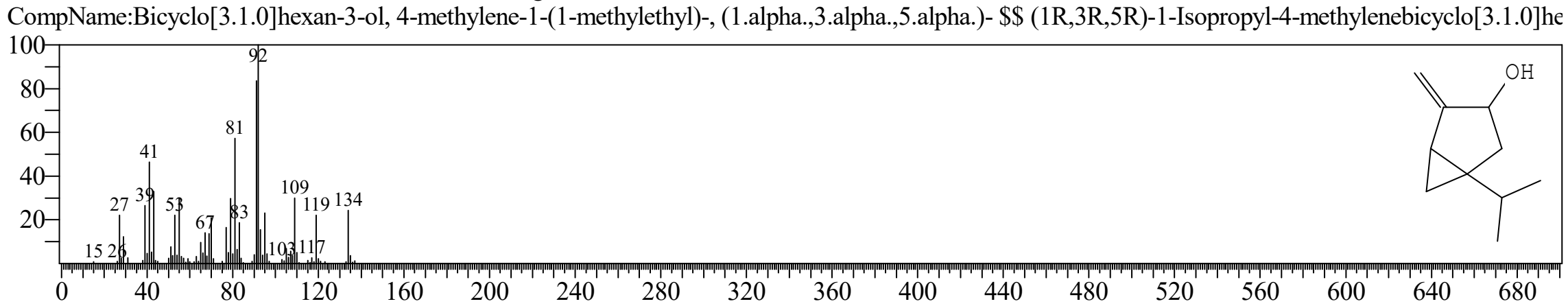

Hit#:3 Entry:122385 Library:NIST14.lib
SI:63 Formula:C19H30O2 CAS:56846-98-1 MolWeight:290 RetIndex:2112
CompName:13,16-Octadecadiynoic acid, methyl ester $$ Methyl 13,16-octadecadiynoate # $$

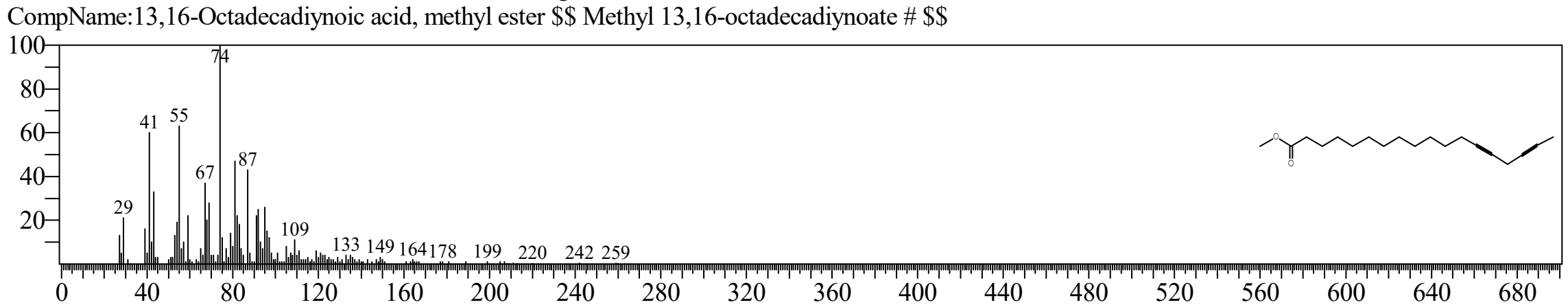

<< Target >>
Line#:43   R.Time:9.140(Scan#:1129) MassPeaks:50
RawMode:Averaged 9.135-9.145(1128-1130) BasePeak:121.10(12793)
BG Mode:Calc. from Peak Group 1 - Event 1 Scan

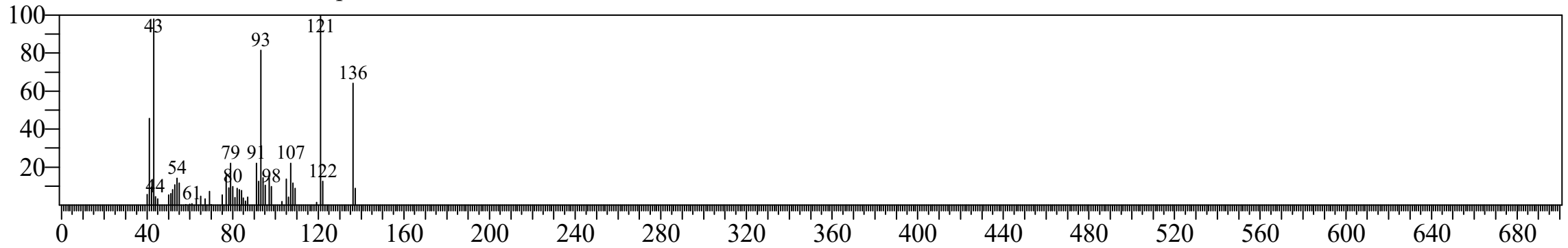

Hit#:1 Entry:112876 Library:WILEY8.LIB
SI:86 Formula:C14H22O CAS:79-69-6 MolWeight:206 RetIndex:0
CompName:.ALPHA.-IONONE, METHYL- $$ 3-BUTEN-2-ONE, 4-(2,5,6,6-TETRAMETHYL-2-CYCLOHEXEN-1-YL)- $$ (3E)-4-(2,5,6,6-TETRAME

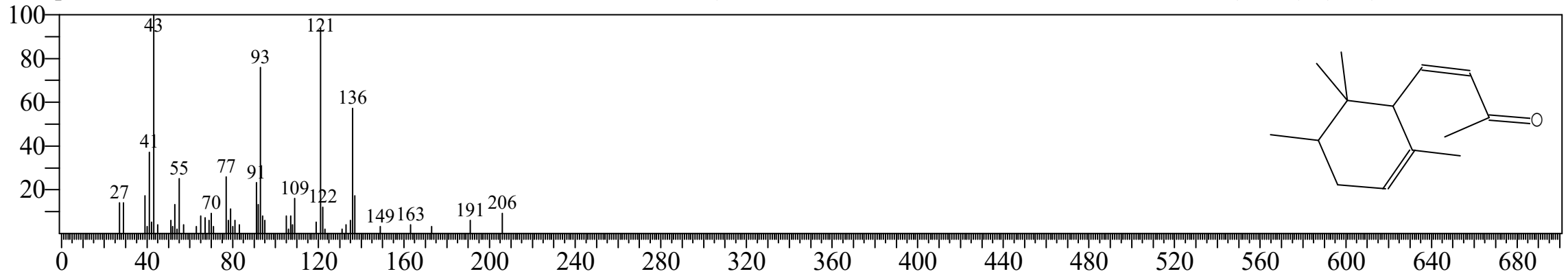

Hit#:2 Entry:19469 Library:NIST14s.lib
SI:86 Formula:C14H22O CAS:79-69-6 MolWeight:206 RetIndex:1490
CompName:.alpha.-Irone $$ 3-Buten-2-one, 4-(2,5,6,6-tetramethyl-2-cyclohexen-1-yl)- $$ .alpha.-Ionone, 6-methyl- $$ Methyl-.alpha.-ionone $$ 6-Methyl-.

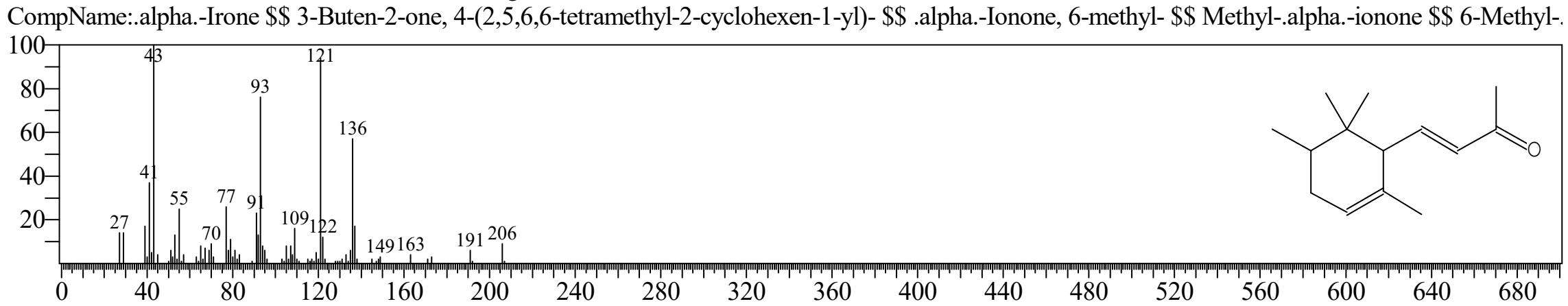

Hit#:3 Entry:98489 Library:WILEY8.LIB
SI:85 Formula:C12H20O2 CAS:80-26-2 MolWeight:196 RetIndex:0
CompName:3-CYCLOHEXENE-1-METHANOL, .ALPHA.,.ALPHA.,4-TRIMETHYL-, ACETATE $$ TERPINYL ACETATE $$ 1-METHYL-1-(4-METH

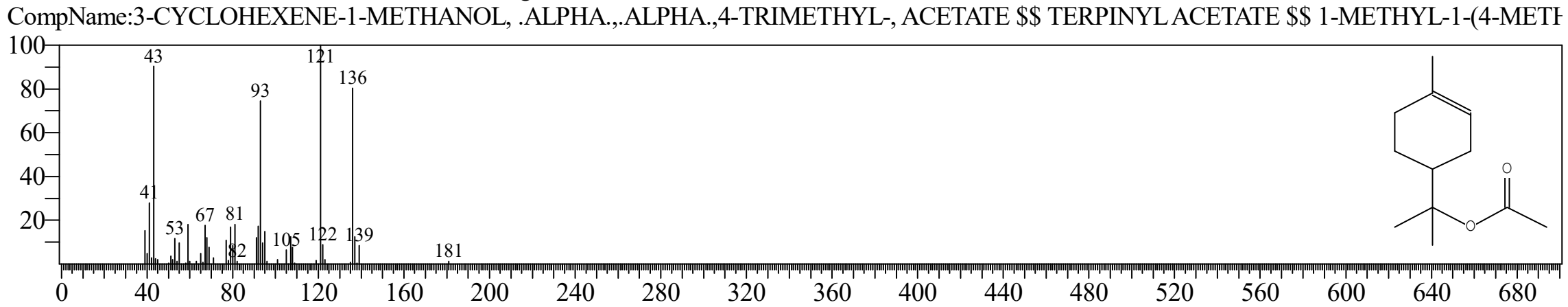

<< Target >>
Line#:44   R.Time:9.275(Scan#:1156) MassPeaks:30
RawMode:Averaged 9.270-9.280(1155-1157) BasePeak:43.00(261223)
BG Mode:Calc. from Peak Group 1 - Event 1 Scan

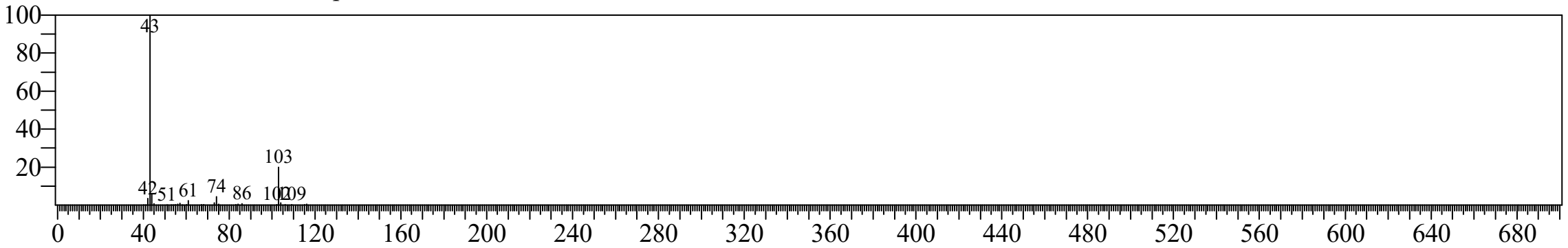

Hit#:1 Entry:71678 Library:WILEY8.LIB
SI:98 Formula:C7H12O5 CAS:25395-31-7 MolWeight:176 RetIndex:0
CompName:1,2,3-PROPANETRIOL, DIACETATE $$ 2-(ACETYLOXY)-1-(HYDROXYMETHYL)ETHYL ACETATE # $$ 2-(ACETYLOXY)-1-(HYDR

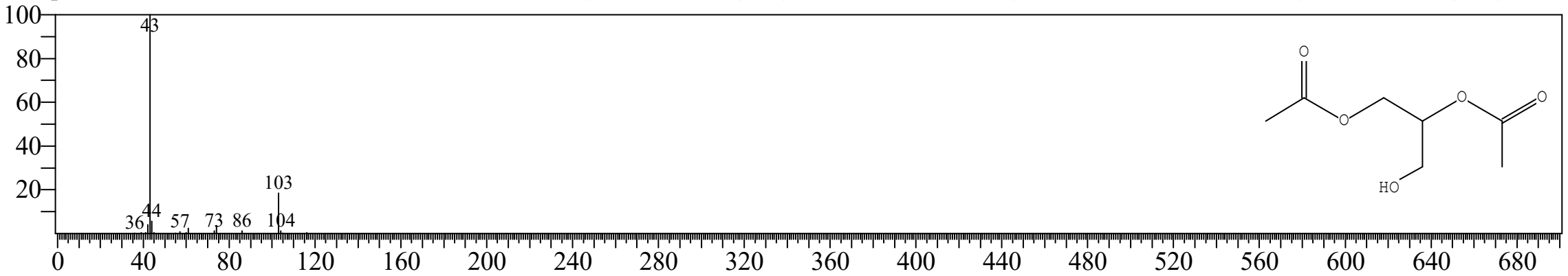

Hit#:2 Entry:9022 Library:NIST14.lib
SI:97 Formula:C5H10O4 CAS:106-61-6 MolWeight:134 RetIndex:1091
CompName:1,2,3-Propanetriol, 1-acetate $$ Acetin, 1-mono- $$ .alpha.-Monoacetin $$ Glycerol .alpha.-monoacetate $$ 1-Monoacetin $$ 2,3-Dihydroxypro

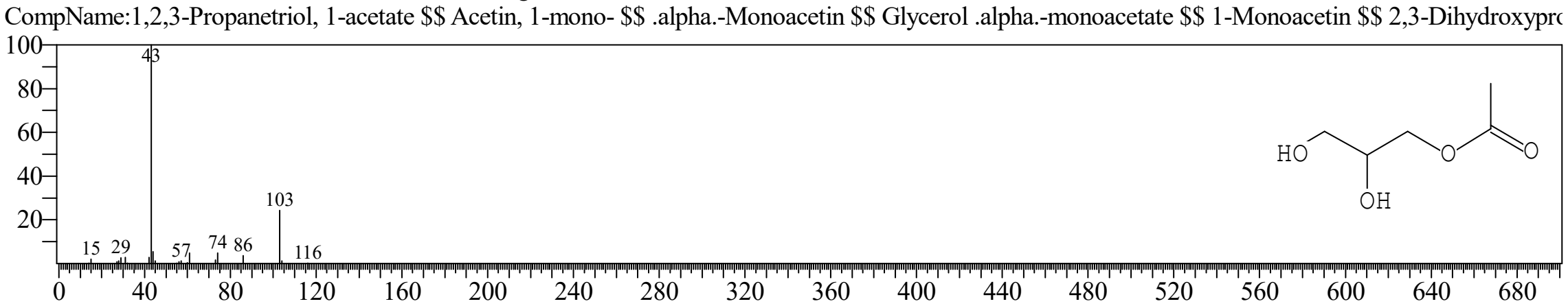

Hit#:3 Entry:25606 Library:WILEY8.LIB
SI:97 Formula:C5H10O4 CAS:26446-35-5 MolWeight:134 RetIndex:0
CompName:2,3-DIHYDROXYPROPYL ACETATE $$ 2,3-DIHYDROXYPROPYL ACETATE # $$ 1,2,3-PROPANETRIOL, MONOACETATE $$ 1-MO

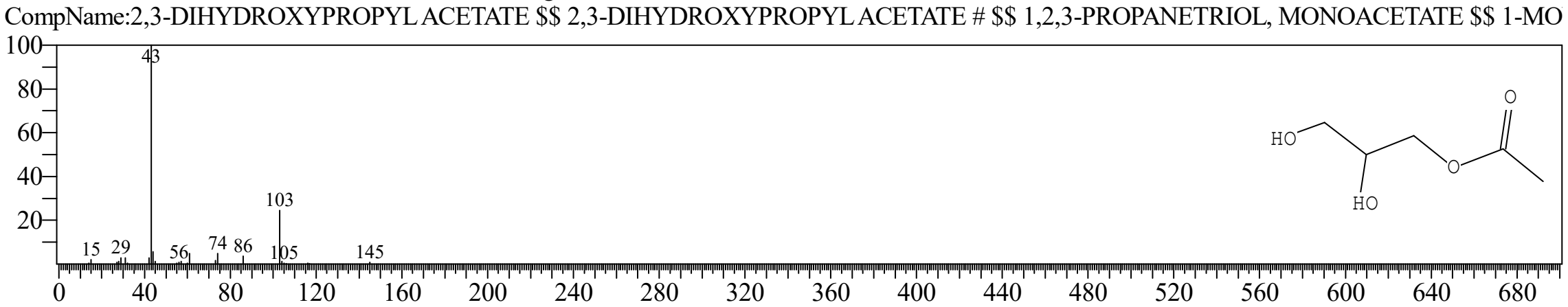

<< Target >>
Line#:45   R.Time:9.345(Scan#:1170) MassPeaks:40
RawMode:Averaged 9.340-9.350(1169-1171) BasePeak:43.00(58446)
BG Mode:Calc. from Peak Group 1 - Event 1 Scan

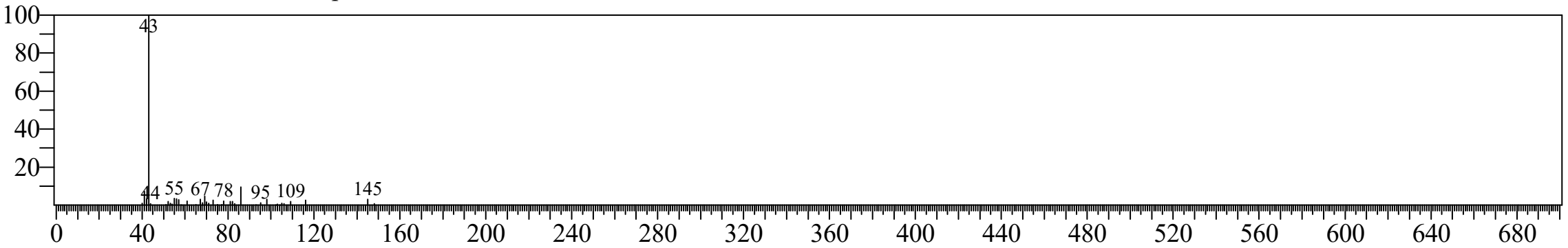

Hit#:1 Entry:3608 Library:WILEY8.LIB
SI:86 Formula:C5H10O CAS:563-80-4 MolWeight:86 RetIndex:0
CompName:2-BUTANONE, 3-METHYL- $$ 3-METHYLBUTAN-2-ONE $$ 2-ACETYL PROPANE $$ 2-ACETYLPROPANE $$ 2-BUTANONE, 3-ME

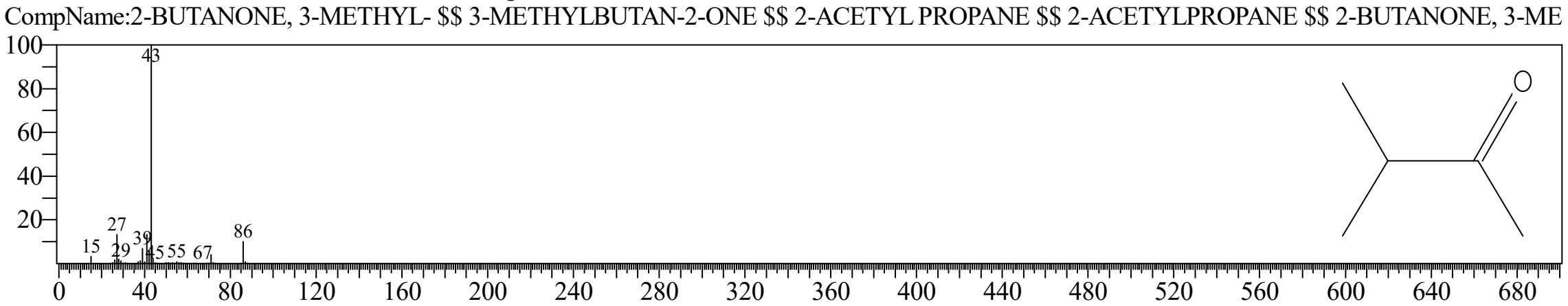

Hit#:2 Entry:3614 Library:WILEY8.LIB
SI:86 Formula:C5H10O CAS:563-80-4 MolWeight:86 RetIndex:0
CompName:2-BUTANONE, 3-METHYL- $$ 3-METHYLBUTAN-2-ONE $$ 2-ACETYL PROPANE $$ 2-ACETYLPROPANE $$ 2-BUTANONE, 3-ME

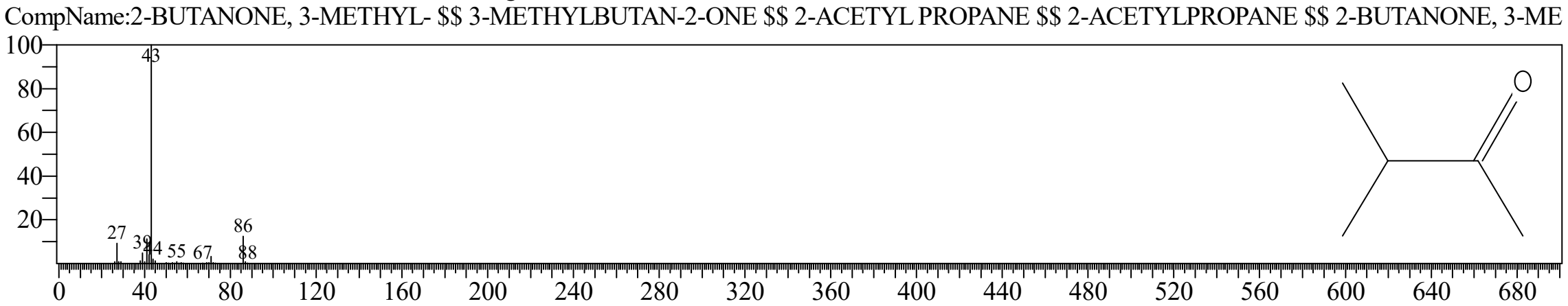

Hit#:3 Entry:20839 Library:WILEY8.LIB
SI:85 Formula:C6H9NO2 CAS:0-00-0 MolWeight:127 RetIndex:0
CompName:2-CYANOPROPYL ACETATE $$ PROPAN, 1-ACETOXY-2-CYANO-

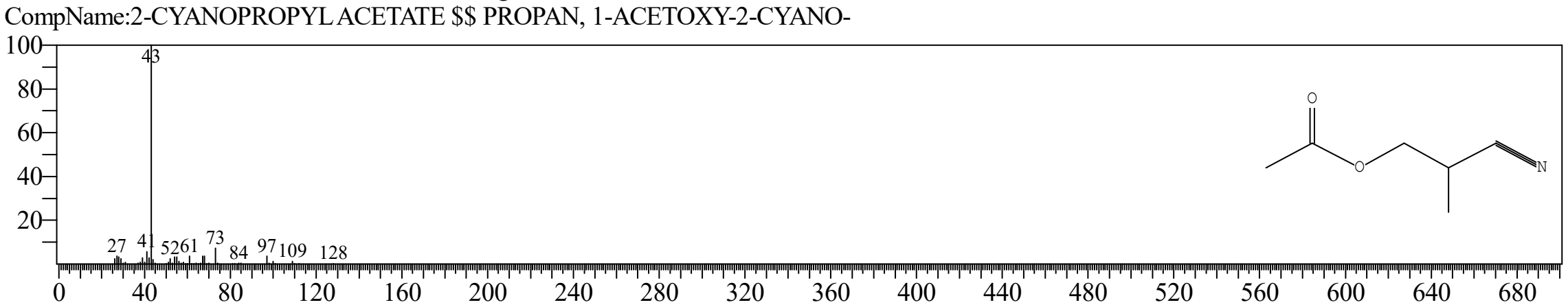

<< Target >>
Line#:46 R.Time:9.615(Scan#:1224) MassPeaks:41
RawMode:Averaged 9.610-9.620(1223-1225) BasePeak:43.00(554535)
BG Mode:Calc. from Peak Group 1 - Event 1 Scan

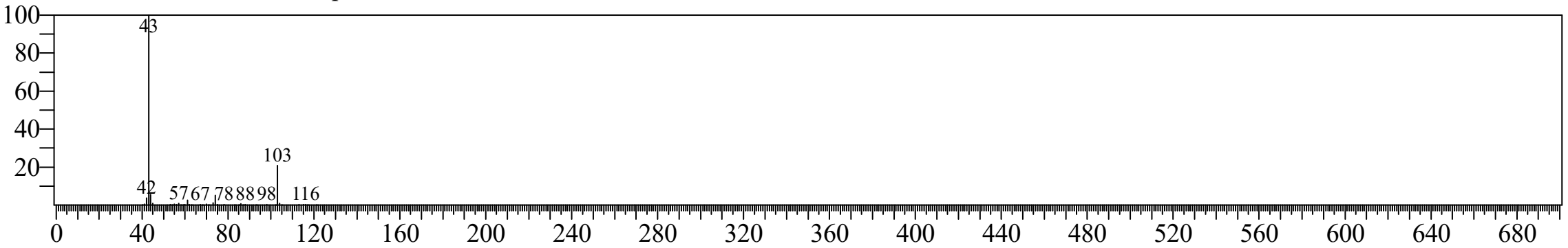

Hit#:1 Entry:71678 Library:WILEY8.LIB
SI:98 Formula:C7H12O5 CAS:25395-31-7 MolWeight:176 RetIndex:0
CompName:1,2,3-PROPANETRIOL, DIACETATE $$ 2-(ACETYLOXY)-1-(HYDROXYMETHYL)ETHYL ACETATE # $$ 2-(ACETYLOXY)-1-(HYDF

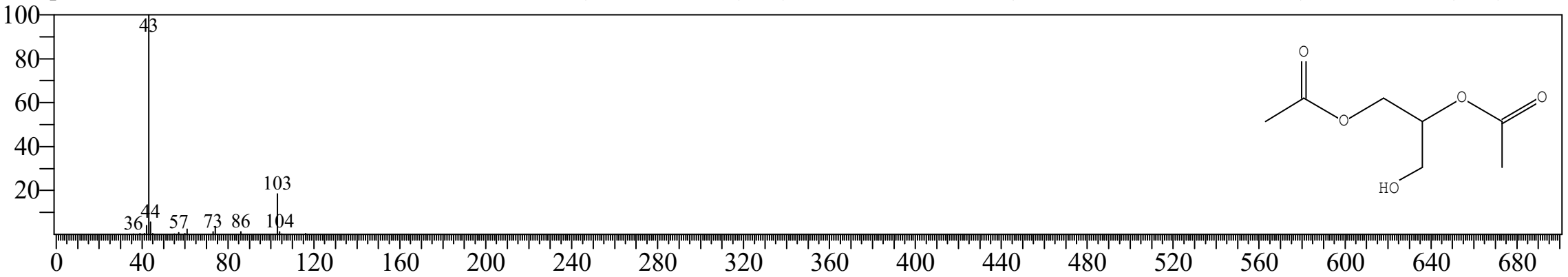

Hit#:2 Entry:9022 Library:NIST14.lib
SI:97 Formula:C5H10O4 CAS:106-61-6 MolWeight:134 RetIndex:1091
CompName:1,2,3-Propanetriol, 1-acetate $$ Acetin, 1-mono- $$ .alpha.-Monoacetin $$ Glycerol .alpha.-monoacetate $$ 1-Monoacetin $$ 2,3-Dihydroxypro

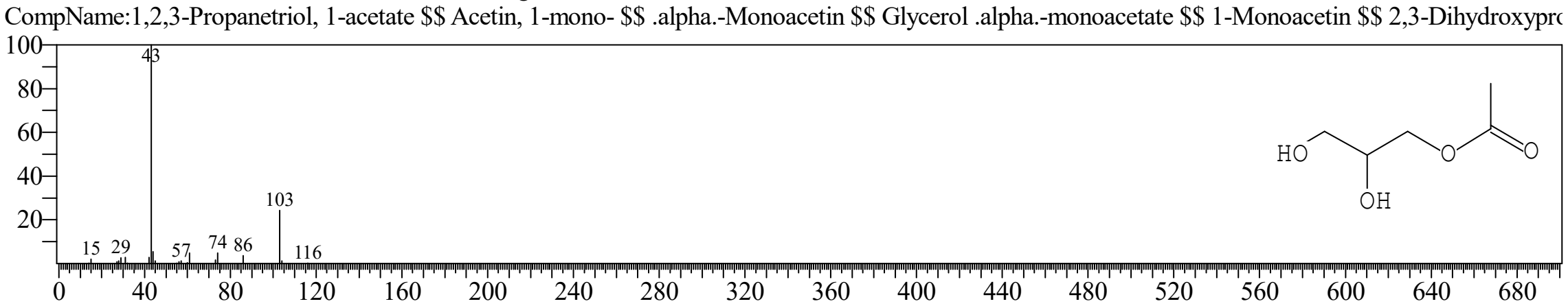

Hit#:3 Entry:25606 Library:WILEY8.LIB
SI:97 Formula:C5H10O4 CAS:26446-35-5 MolWeight:134 RetIndex:0
CompName:2,3-DIHYDROXYPROPYL ACETATE $$ 2,3-DIHYDROXYPROPYL ACETATE # $$ 1,2,3-PROPANETRIOL, MONOACETATE $$ 1-MO

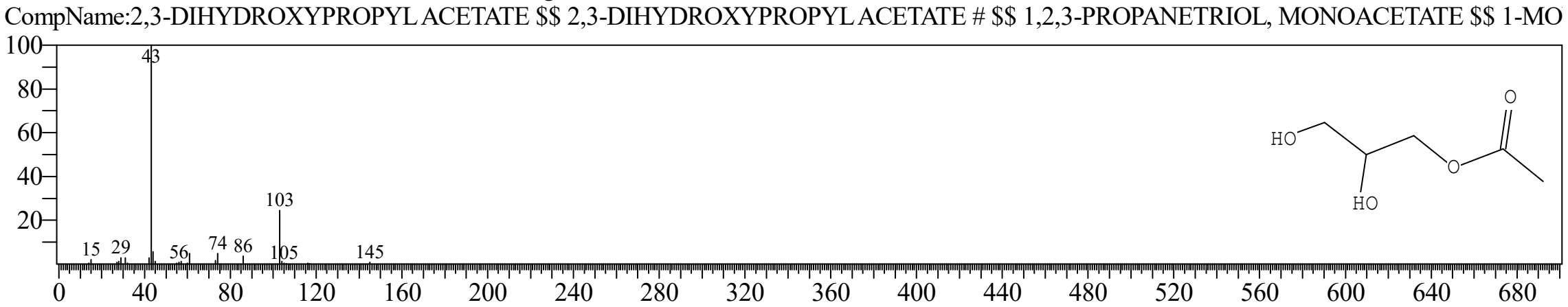

<< Target >>
Line#:47 R.Time:9.675(Scan#:1236) MassPeaks:53
RawMode:Averaged 9.670-9.680(1235-1237) BasePeak:43.00(57926)
BG Mode:Calc. from Peak Group 1 - Event 1 Scan

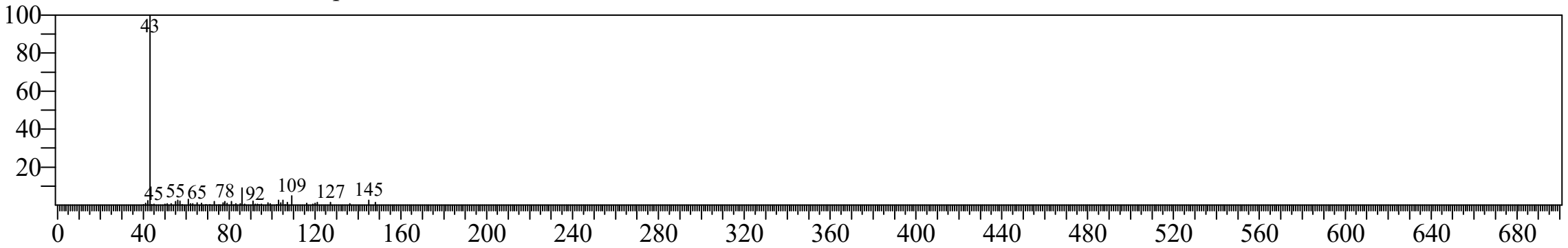

Hit#:1 Entry:71703 Library:WILEY8.LIB
SI:83 Formula:C7H12O5 CAS:0-00-0 MolWeight:176 RetIndex:0
CompName:1,2,3-PROPANETRIOL, 1,2-DIACETATE $$ 2-(ACETYLOXY)-1-(HYDROXYMETHYL)ETHYL ACETATE

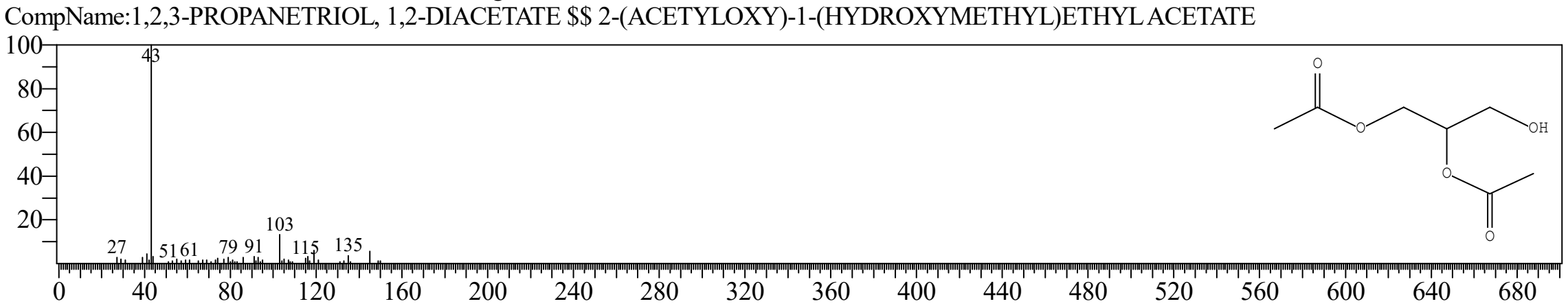

Hit#:2 Entry:71680 Library:WILEY8.LIB
SI:83 Formula:C7H12O5 CAS:102-62-5 MolWeight:176 RetIndex:0
CompName:2-(ACETYLOXY)-1-(HYDROXYMETHYL)ETHYL ACETATE # $$ (HYDROXYMETHYL)ETHYLENE ACETATE $$ 1,2,3-PROPANETI

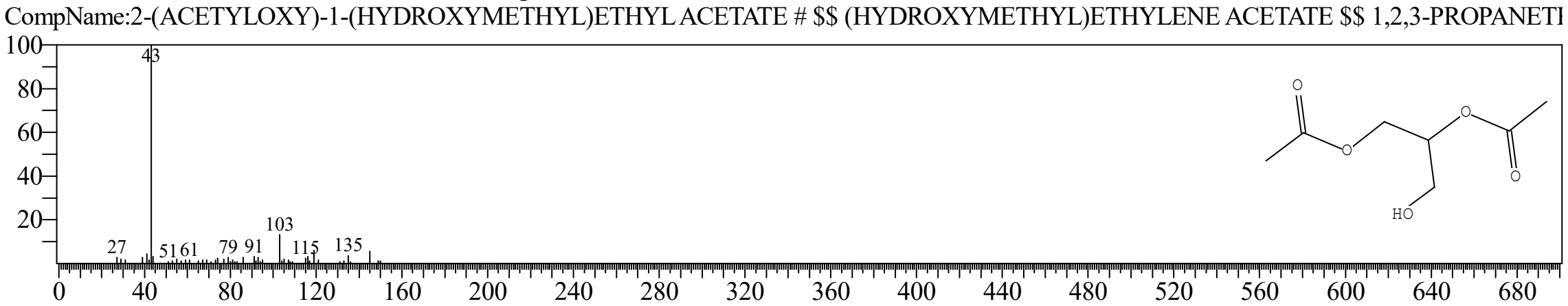

Hit#:3 Entry:304151 Library:WILEY8.LIB
SI:83 Formula:C22H32O4 CAS:51424-66-9 MolWeight:360 RetIndex:0
CompName:(-)-3-BETA-ACETOXY-5-ETIENIC ACID $$ 3-(ACETYLOXY)ANDROST-5-ENE-17-CARBOXYLIC ACID $$ ANDROST-5-ENE-17-CAI

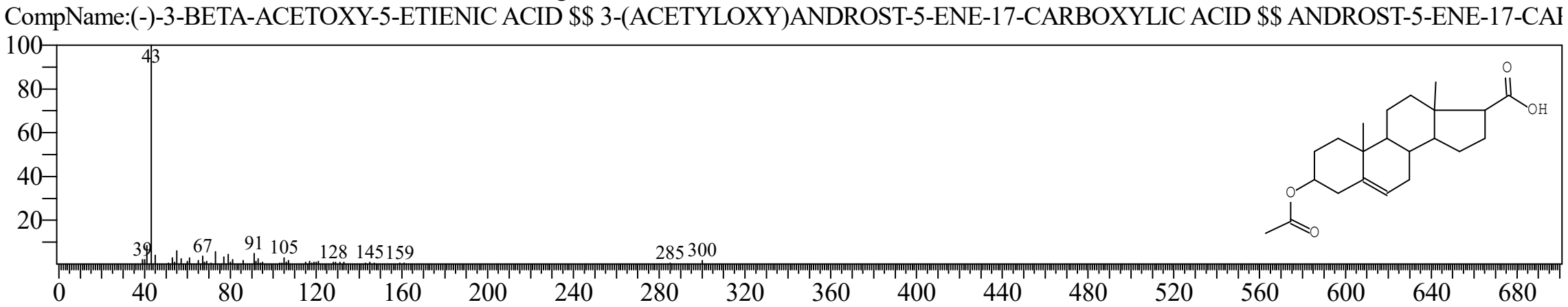

<< Target >>
Line#:48   R.Time:9.850(Scan#:1271) MassPeaks:48
RawMode:Averaged 9.845-9.855(1270-1272) BasePeak:55.05(9093)
BG Mode:Calc. from Peak Group 1 - Event 1 Scan

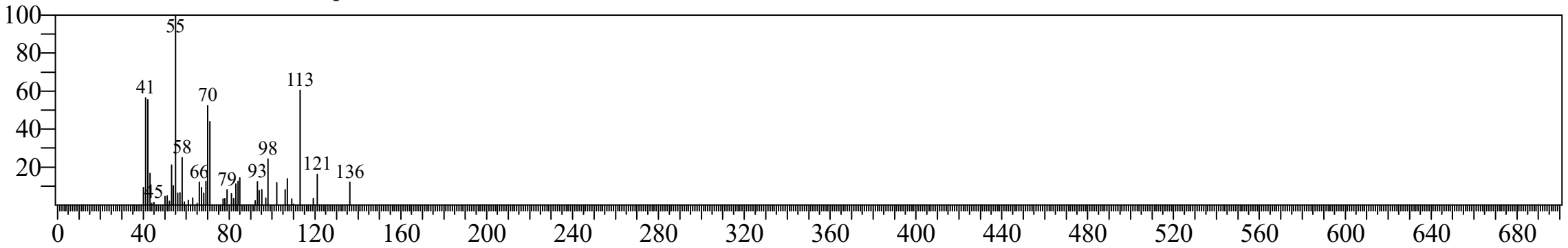

Hit#:1 Entry:20323 Library:WILEY8.LIB
SI:76 Formula:C8H14O CAS:2363-89-5 MolWeight:126 RetIndex:0
CompName:2 OCTENAL $$ 2-OCTEN-1-AL $$ 2-OCTENAL $$ 2-OCTENEL $$ AI3-36269 $$ CCRIS 3418 $$ EINECS 219-115-4 $$ FEMA NO. 321

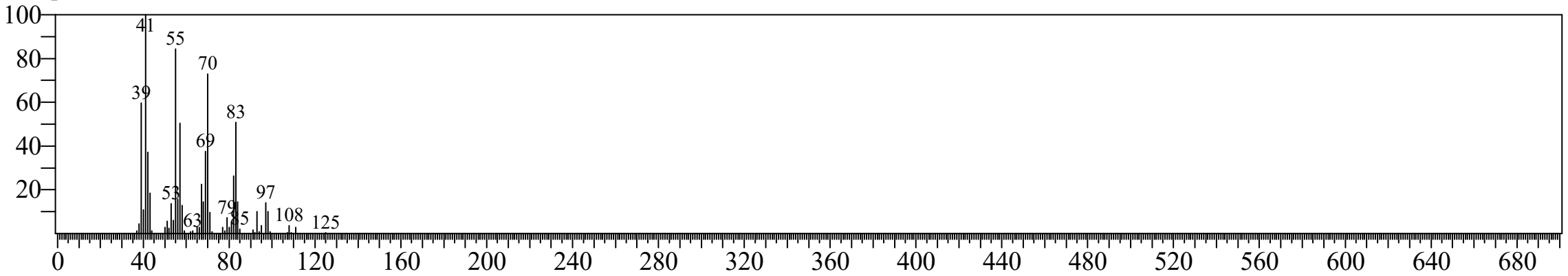

Hit#:2 Entry:20282 Library:WILEY8.LIB
SI:76 Formula:C8H14O CAS:13609-59-1 MolWeight:126 RetIndex:0
CompName:CYCLOHEPTANONE, 4-METHYL-, (R)- $$ 4-METHYLCYCLOHEPTANONE # $$ 4-METHYLCYCLOHEPTANONE $$ CYCLOHEPTA

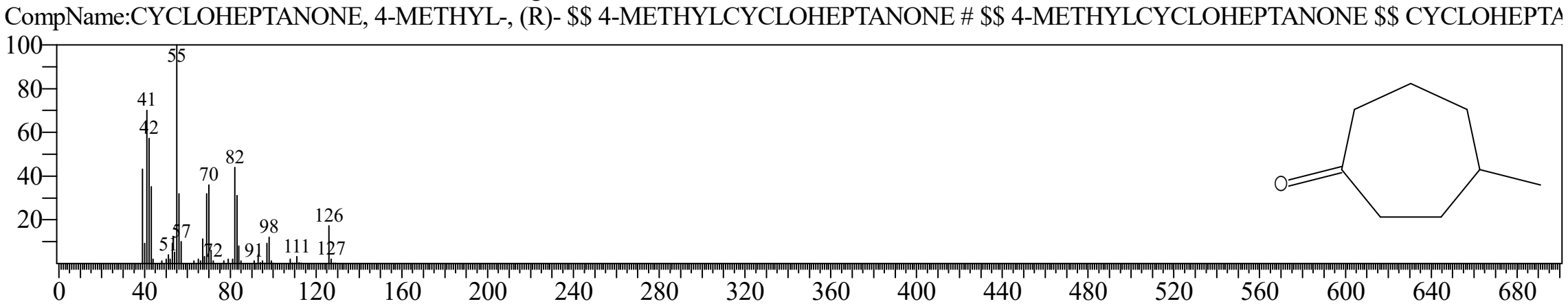

Hit#:3 Entry:12739 Library:WILEY8.LIB
SI:75 Formula:C6H11NO CAS:0-00-0 MolWeight:113 RetIndex:0
CompName:AZEPAN-2-ONE $$ 2-AZEPANONE

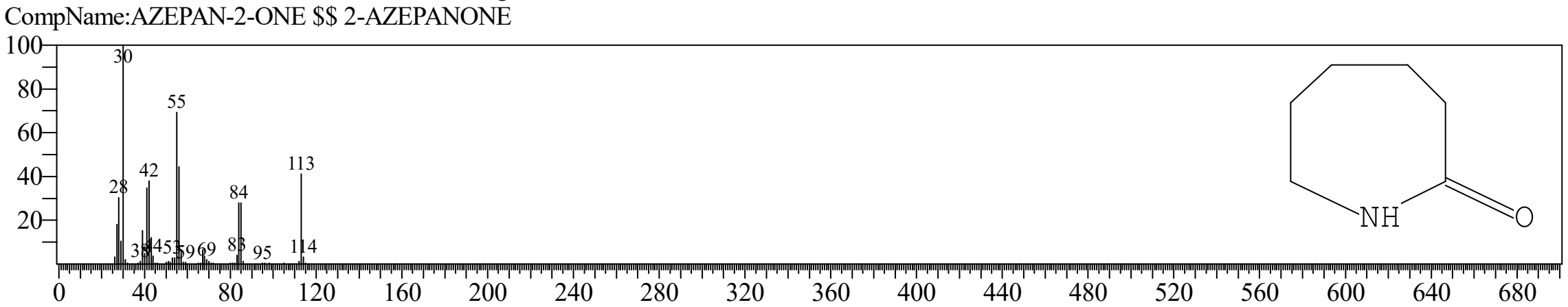

<< Target >>
Line#:49   R.Time:10.010(Scan#:1303) MassPeaks:63
RawMode:Averaged 10.005-10.015(1302-1304) BasePeak:43.00(21235)
BG Mode:Calc. from Peak Group 1 - Event 1 Scan

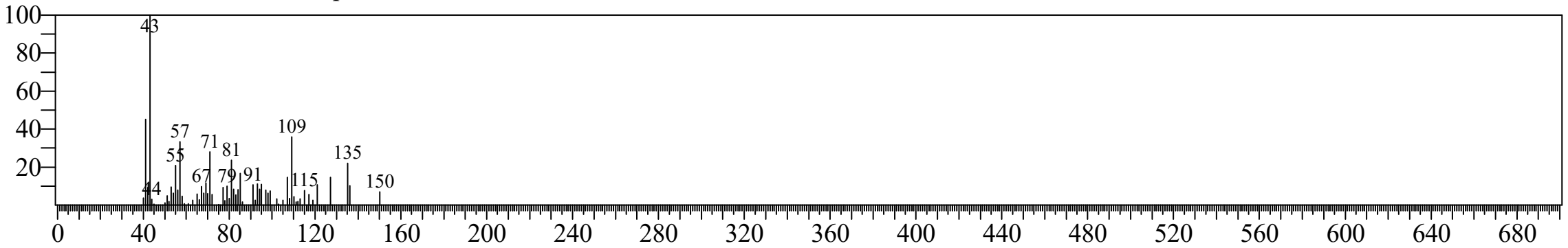

Hit#:1 Entry:62575 Library:WILEY8.LIB
SI:81 Formula:C10H16O2 CAS:512-85-6 MolWeight:168 RetIndex:0
CompName:2,3-DIOXABICYCLO[2.2.2]OCT-5-ENE, 1-METHYL-4-(1-METHYLETHYL)- $$ 1-ISOPROPYL-4-METHYL-2,3-DIOXABICYCLO[2.2.

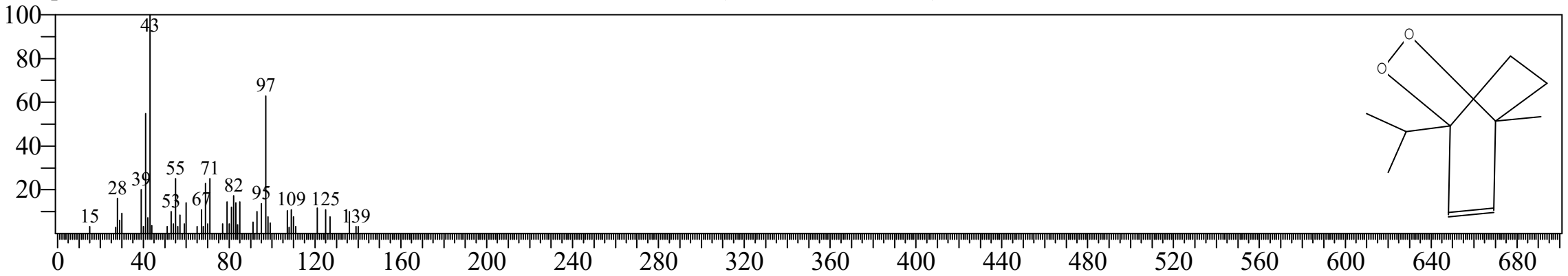

Hit#:2 Entry:159034 Library:WILEY8.LIB
SI:81 Formula:C16H30O CAS:0-00-0 MolWeight:238 RetIndex:0
CompName:1-(2-METHYLENE-3-PENTYLCYCLOPROPYL)-1-HEPTANOL $$ CYCLOPROPAN, 1-(1-HYDROXY-1-HEPTYL)-2-METHYLEN-3-PE

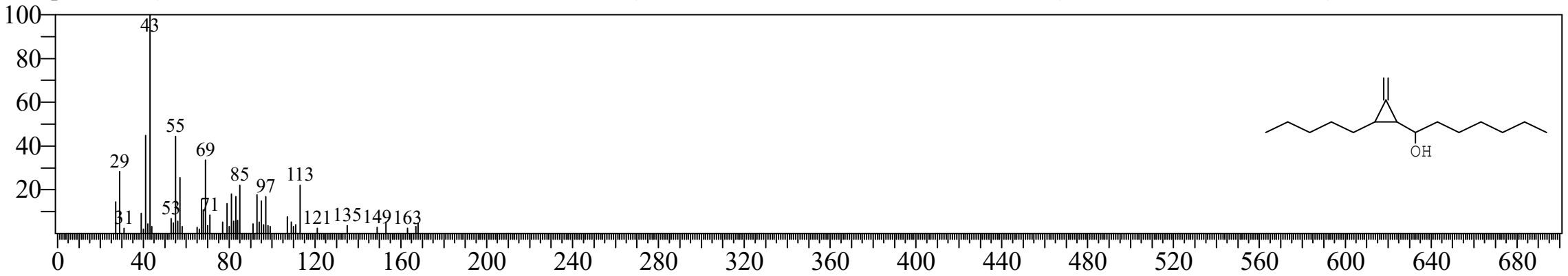

Hit#:3 Entry:77343 Library:NIST14.lib
SI:81 Formula:C16H30O CAS:0-00-0 MolWeight:238 RetIndex:1705
CompName:Cyclopropane, 1-(1-hydroxy-1-heptyl)-2-methylene-3-pentyl- $$ 1-(2-Methylene-3-pentylcyclopropyl)-1-heptanol # $$

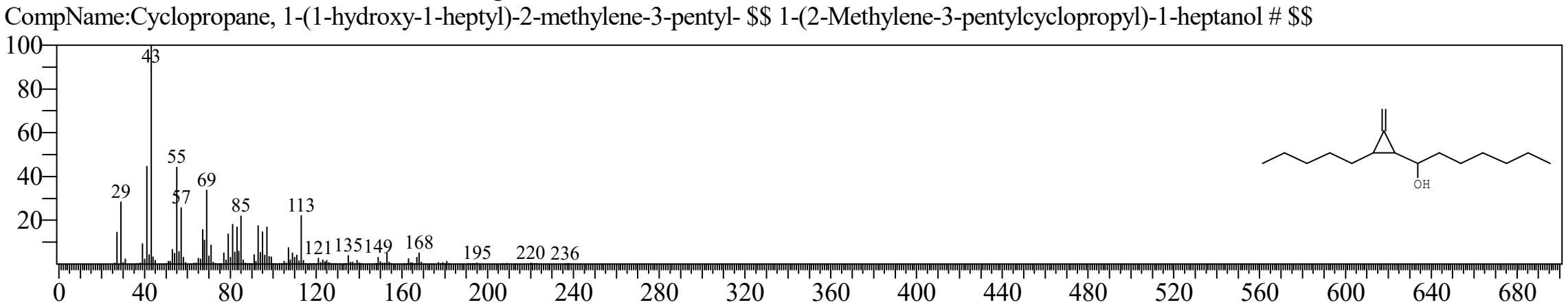

<< Target >>
Line#:50   R.Time:10.120(Scan#:1325) MassPeaks:43
RawMode:Averaged 10.115-10.125(1324-1326) BasePeak:43.00(8811)
BG Mode:Calc. from Peak Group 1 - Event 1 Scan

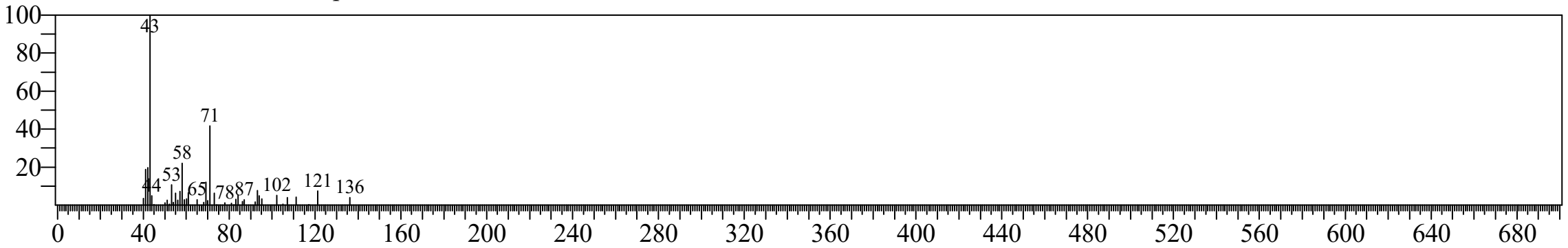

Hit#:1 Entry:5589 Library:NIST14s.lib
SI:86 Formula:C6H10O3 CAS:674-26-0 MolWeight:130 RetIndex:1156
CompName:dl-Mevalonic acid lactone $$ 2H-Pyran-2-one, tetrahydro-4-hydroxy-4-methyl-, (.+/-.)- $$ 4-Hydroxy-4-methyltetrahydro-2H-pyran-2-one $$ 2H

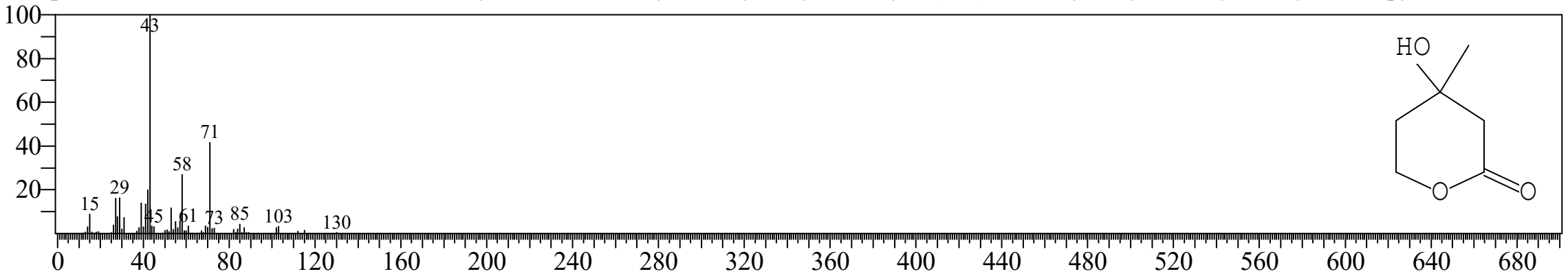

Hit#:2 Entry:23067 Library:WILEY8.LIB
SI:84 Formula:C6H10O3 CAS:503-48-0 MolWeight:130 RetIndex:0
CompName:2H-PYRAN-2-ONE, TETRAHYDRO-4-HYDROXY-4-METHYL- $$ VALERIC ACID, 3,5-DIHYDROXY-3-METHYL-, .DELTA.-LACTON

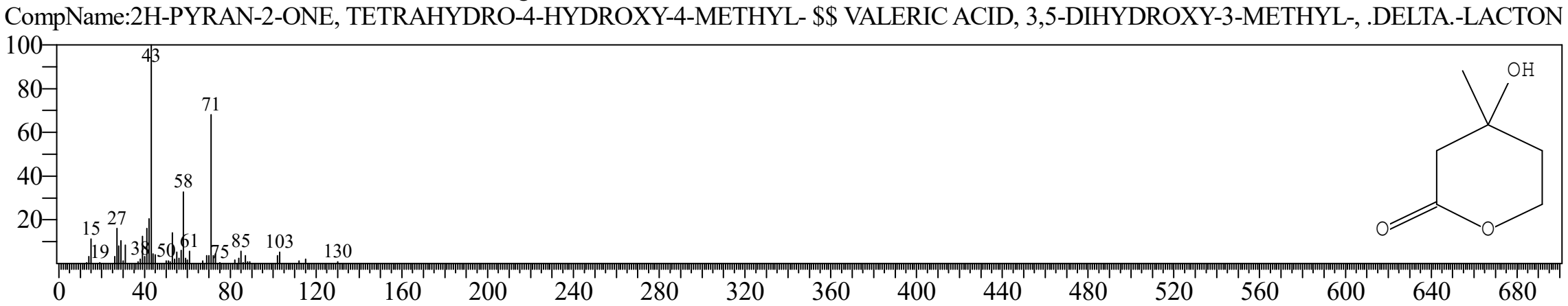

Hit#:3 Entry:5588 Library:NIST14s.lib
SI:84 Formula:C6H10O3 CAS:674-26-0 MolWeight:130 RetIndex:1156
CompName:dl-Mevalonic acid lactone $$ 2H-Pyran-2-one, tetrahydro-4-hydroxy-4-methyl-, (.+/-.)- $$ 4-Hydroxy-4-methyltetrahydro-2H-pyran-2-one $$ 2H

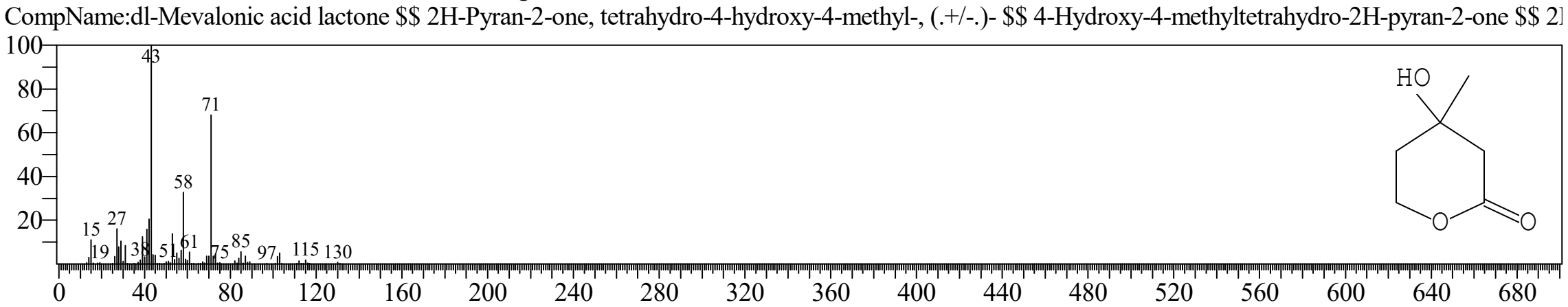

<< Target >>
Line#:51   R.Time:10.245(Scan#:1350) MassPeaks:68
RawMode:Averaged 10.240-10.250(1349-1351) BasePeak:135.10(14013)
BG Mode:Calc. from Peak Group 1 - Event 1 Scan

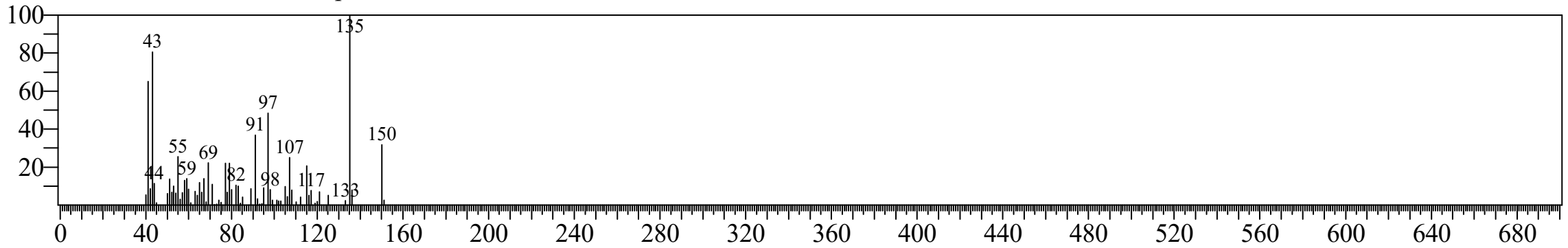

Hit#:1 Entry:35704 Library:NIST14.lib
SI:76 Formula:C10H16O3 CAS:135760-25-7 MolWeight:184 RetIndex:1009
CompName:Ascaridole epoxide

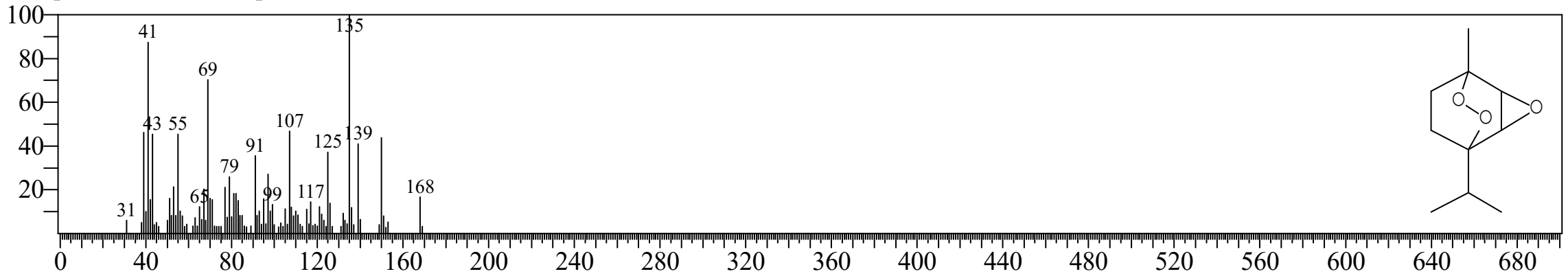

Hit#:2 Entry:15857 Library:NIST14.lib
SI:73 Formula:C10H14O CAS:33746-71-3 MolWeight:150 RetIndex:0
CompName:(Z)-2,6-Dimethylocta-2,5,7-trien-4-one $$ (Z)-Tagetenone $$ cis-Tagetenone $$ cis-Ocimenone $$

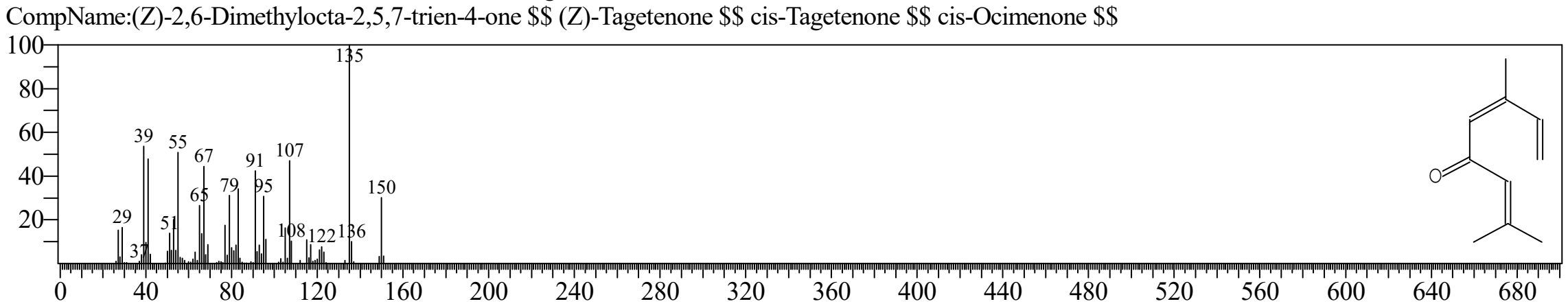

Hit#:3 Entry:41440 Library:WILEY8.LIB
SI:73 Formula:C10H14O CAS:15186-51-3 MolWeight:150 RetIndex:0
CompName:.ALPHA.-NAGINATENE $$ .GAMMA.-CLAUSENANE $$ 3-METHYL-2-(2-METHYL-2-BUTENYL)-FURAN $$ FURAN, 3-METHYL-2-

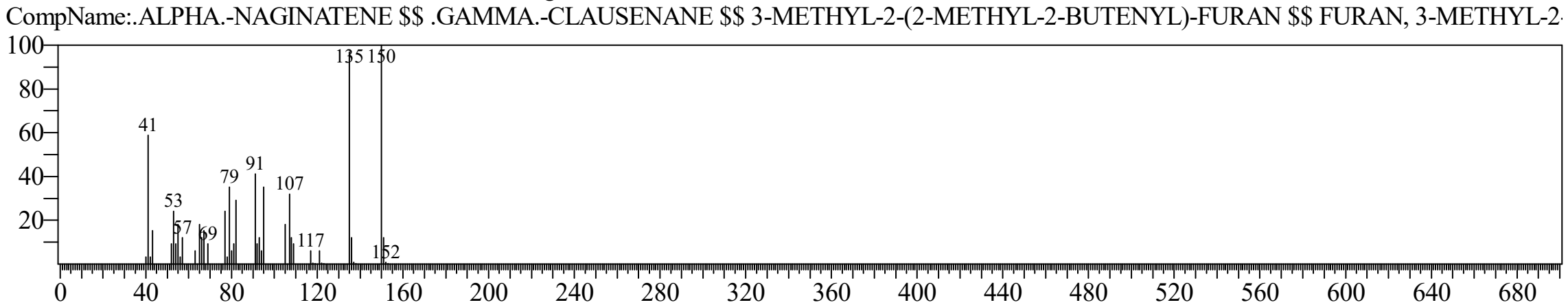

<< Target >>
Line#:52 R.Time:10.340(Scan#:1369) MassPeaks:59
RawMode:Averaged 10.335-10.345(1368-1370) BasePeak:93.10(13508)
BG Mode:Calc. from Peak Group 1 - Event 1 Scan

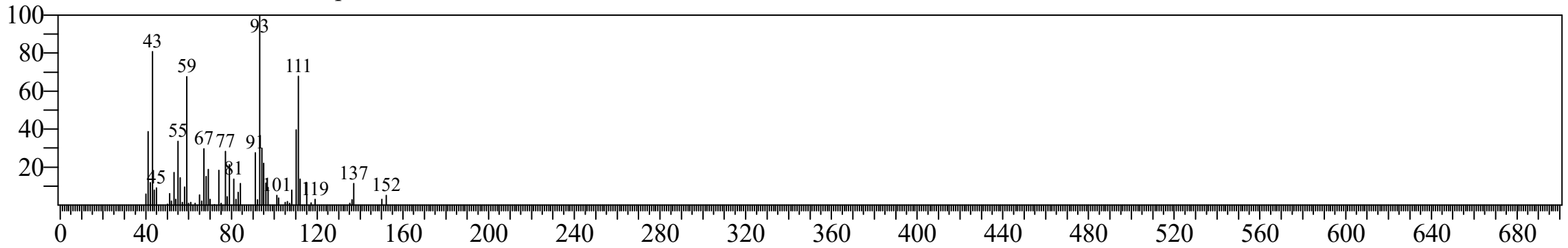

Hit#:1 Entry:65663 Library:WILEY8.LIB
SI:79 Formula:C10H18O2 CAS:5989-33-3 MolWeight:170 RetIndex:0
CompName:2-FURANMETHANOL, 5-ETHENYLTETRAHYDRO-.ALPHA.,.ALPHA.,5-TRIMETHYL-, CIS- $$ FURFURYL ALCOHOL, TETRAHYD

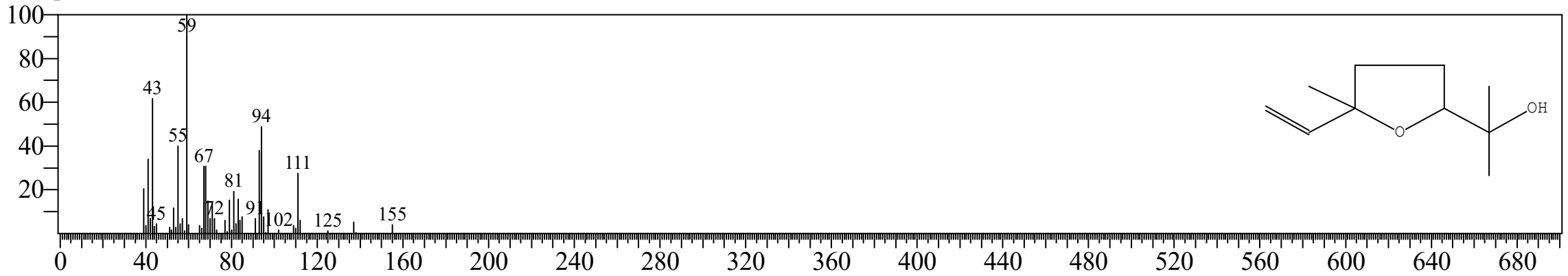

Hit#:2 Entry:65664 Library:WILEY8.LIB
SI:79 Formula:C10H18O2 CAS:34995-77-2 MolWeight:170 RetIndex:0
CompName:2-FURANMETHANOL, 5-ETHENYLTETRAHYDRO-.ALPHA.,.ALPHA.,5-TRIMETHYL-, TRANS- $$ FURFURYL ALCOHOL, TETRAH

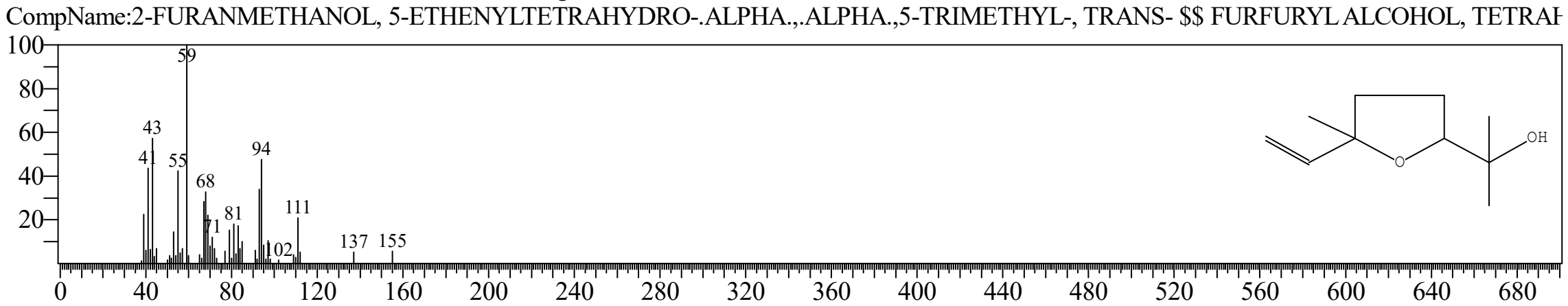

Hit#:3 Entry:12424 Library:NIST14.lib
SI:78 Formula:C8H14O2 CAS:109532-58-3 MolWeight:142 RetIndex:1208
CompName:Spiro[2.4]heptane-5-methanol, 5-hydroxy- $$ 5-(Hydroxymethyl)spiro[2.4]heptan-5-ol # $$

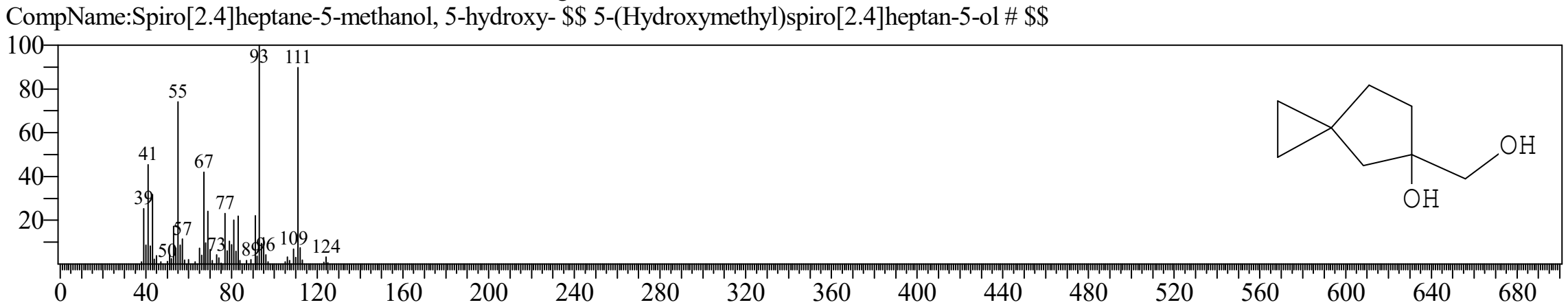

<< Target >>
Line#:53   R.Time:10.470(Scan#:1395) MassPeaks:55
RawMode:Averaged 10.465-10.475(1394-1396) BasePeak:135.10(9694)
BG Mode:Calc. from Peak Group 1 - Event 1 Scan

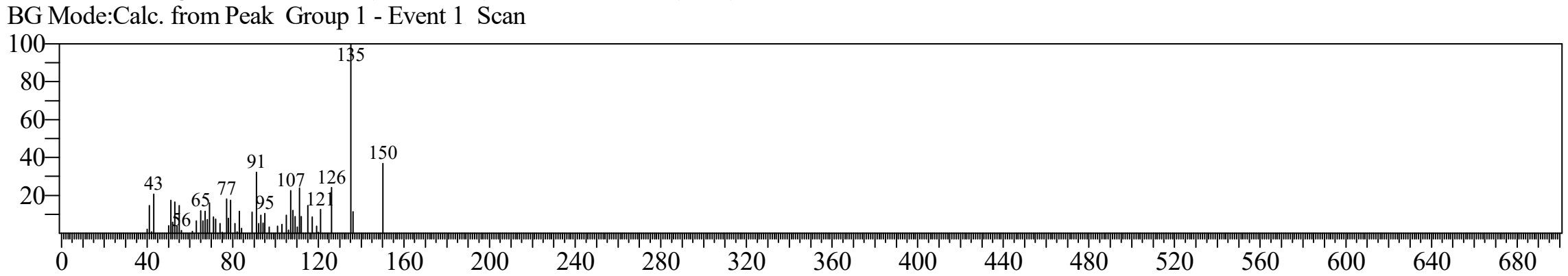

Hit#:1 Entry:41878 Library:WILEY8.LIB
SI:79 Formula:C11H18 CAS:13943-77-6 MolWeight:150 RetIndex:0
CompName:NAPHTHALENE, 1,2,3,4,4A,5,6,7-OCTAHYDRO-4A-METHYL- $$ 4A-METHYL-1,2,3,4,4A,5,6,7-OCTAHYDRONAPHTHALENE # $$ 4

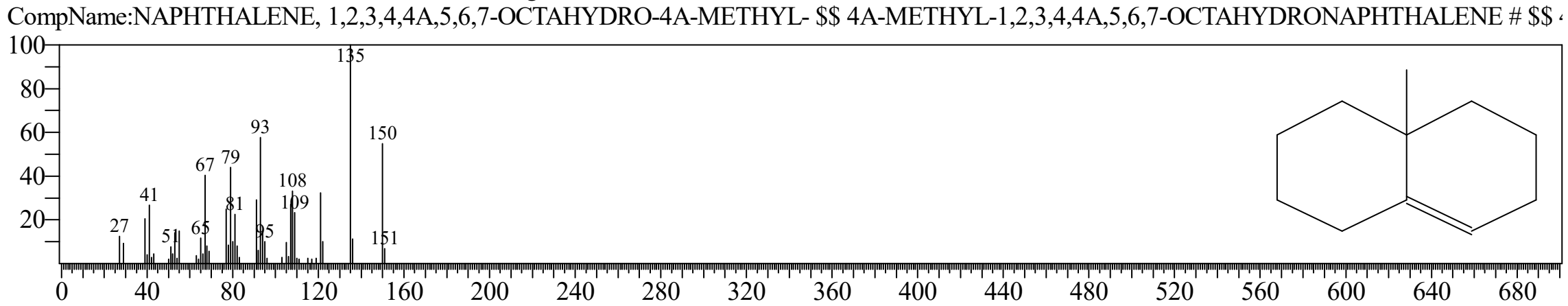

Hit#:2 Entry:15959 Library:NIST14.lib
SI:79 Formula:C11H18 CAS:13943-77-6 MolWeight:150 RetIndex:1185
CompName:Naphthalene, 1,2,3,4,4a,5,6,7-octahydro-4a-methyl- $$ 4a-Methyl-1,2,3,4,4a,5,6,7-octahydronaphthalene # $$

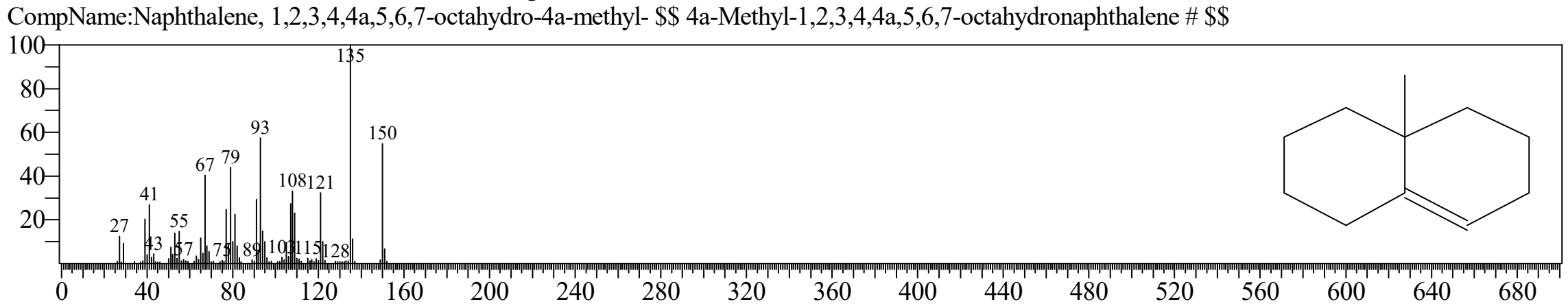

Hit#:3 Entry:15872 Library:NIST14.lib
SI:78 Formula:C10H14O CAS:15186-51-3 MolWeight:150 RetIndex:0
CompName:3-Methyl-2-(2-methyl-2-butenyl)-furan $$ Furan, 3-methyl-2-(3-methyl-2-butenyl)- $$ Rosefuran $$ .alpha.-Naginatene $$ Furan, 3-methyl-2-(3

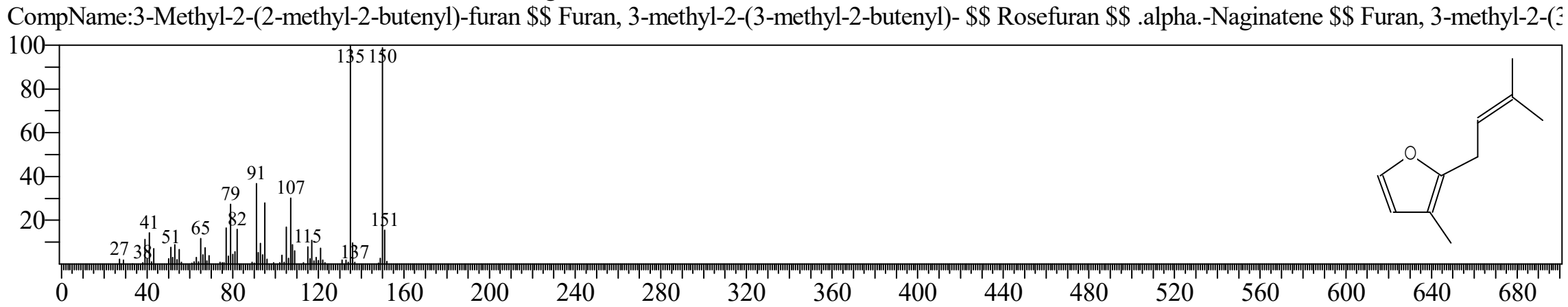

<< Target >>
Line#:54   R.Time:10.505(Scan#:1402) MassPeaks:44
RawMode:Averaged 10.500-10.510(1401-1403) BasePeak:43.00(7853)
BG Mode:Calc. from Peak Group 1 - Event 1 Scan

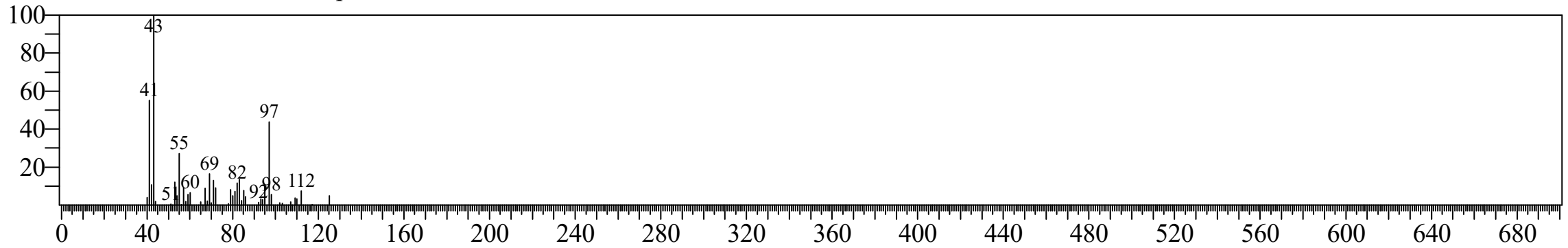

Hit#:1 Entry:25508 Library:NIST14.lib
SI:90 Formula:C10H16O2 CAS:1619-26-7 MolWeight:168 RetIndex:0
CompName:(1S,2R,4R,7R)-4-Isopropyl-7-methyl-3,8-dioxatricyclo[5.1.0.02,4]octane $$ 3,8-Dioxatricyclo[5.1.0.02,4]octane, 4-methyl-7-(1-methylethyl)-,

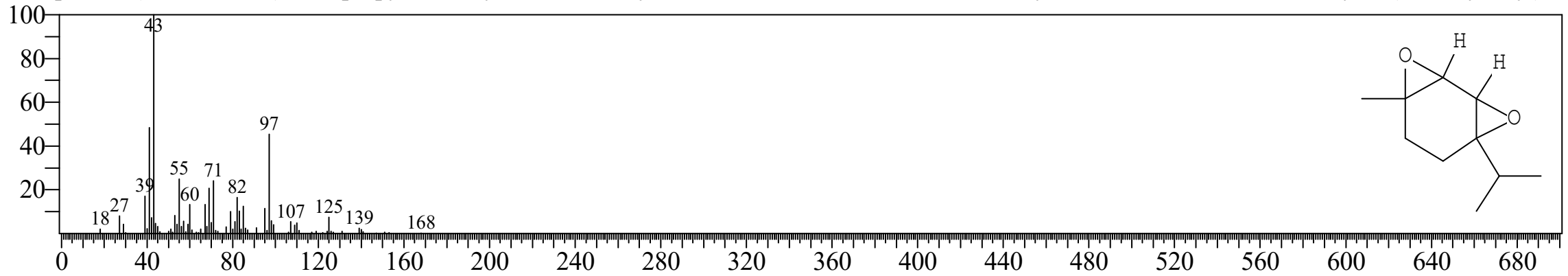

Hit#:2 Entry:62575 Library:WILEY8.LIB
SI:85 Formula:C10H16O2 CAS:512-85-6 MolWeight:168 RetIndex:0
CompName:2,3-DIOXABICYCLO[2.2.2]OCT-5-ENE, 1-METHYL-4-(1-METHYLETHYL)- $$ 1-ISOPROPYL-4-METHYL-2,3-DIOXABICYCLO[2.2.2

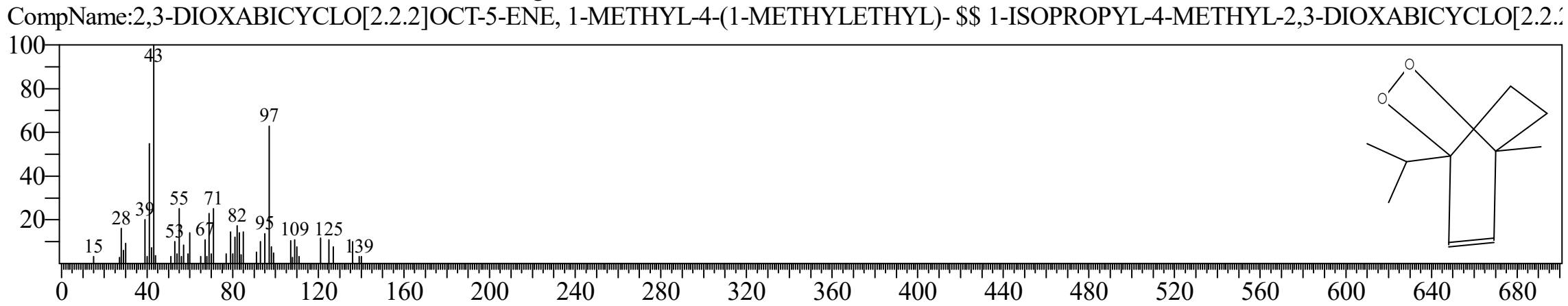

Hit#:3 Entry:12082 Library:WILEY8.LIB
SI:83 Formula:C7H12O CAS:1713-33-3 MolWeight:112 RetIndex:0
CompName:7-OXABICYCLO[4.1.0]HEPTANE, 1-METHYL- $$ 1-METHYL-7-OXABICYCLO[4.1.0]HEPTANE $$ 1-METHYL-7-OXABICYCLO[4.1

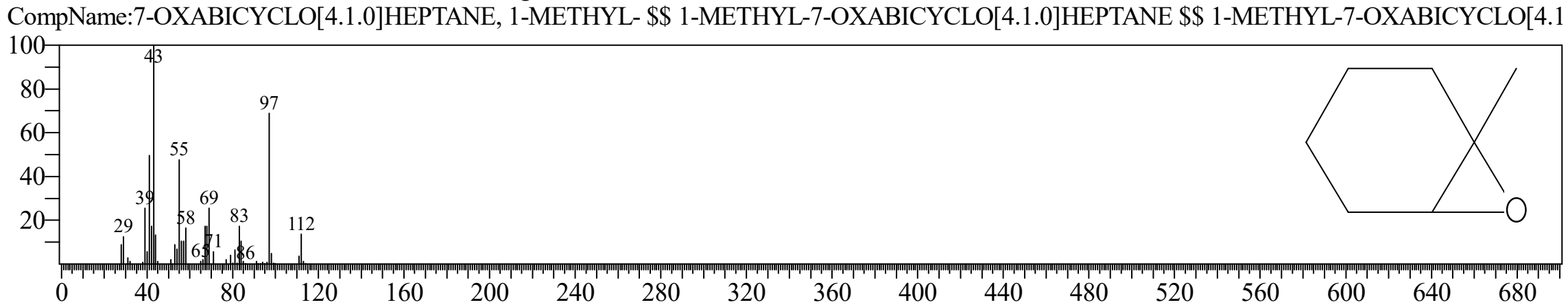

<< Target >>
Line#:55   R.Time:10.600(Scan#:1421) MassPeaks:56
RawMode:Averaged 10.595-10.605(1420-1422) BasePeak:43.00(13884)
BG Mode:Calc. from Peak Group 1 - Event 1 Scan

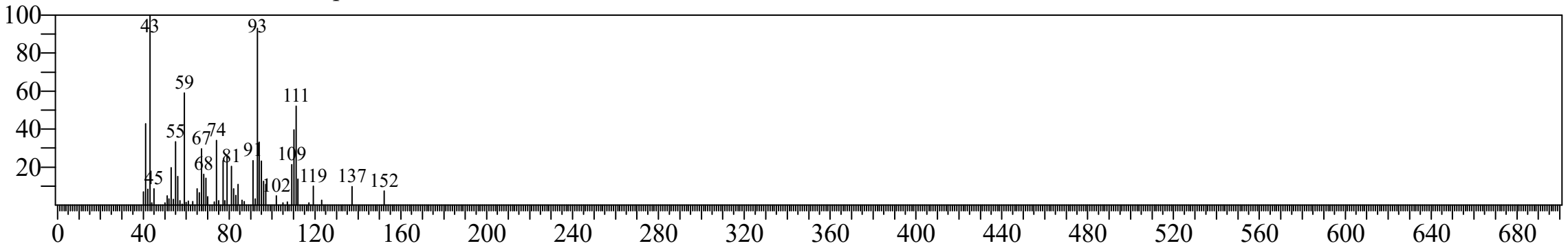

Hit#:1 Entry:65663 Library:WILEY8.LIB
SI:79 Formula:C10H18O2 CAS:5989-33-3 MolWeight:170 RetIndex:0
CompName:2-FURANMETHANOL, 5-ETHENYLTETRAHYDRO-.ALPHA.,.ALPHA.,5-TRIMETHYL-, CIS- $$ FURFURYL ALCOHOL, TETRAHYD

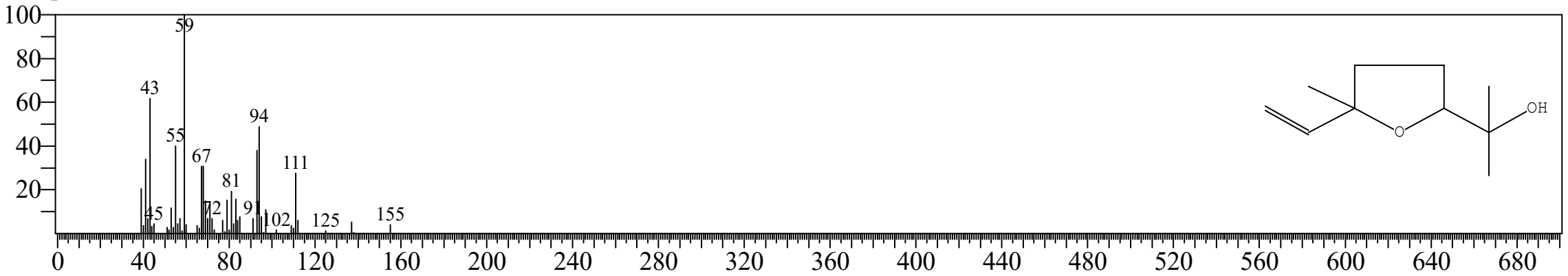

Hit#:2 Entry:65664 Library:WILEY8.LIB
SI:79 Formula:C10H18O2 CAS:34995-77-2 MolWeight:170 RetIndex:0
CompName:2-FURANMETHANOL, 5-ETHENYLTETRAHYDRO-.ALPHA.,.ALPHA.,5-TRIMETHYL-, TRANS- $$ FURFURYL ALCOHOL, TETRAH

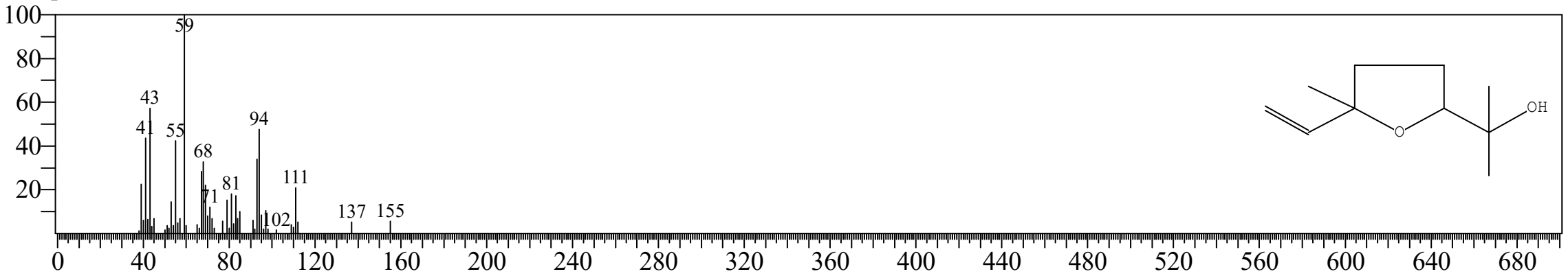

Hit#:3 Entry:12424 Library:NIST14.lib
SI:78 Formula:C8H14O2 CAS:109532-58-3 MolWeight:142 RetIndex:1208
CompName:Spiro[2.4]heptane-5-methanol, 5-hydroxy- $$ 5-(Hydroxymethyl)spiro[2.4]heptan-5-ol # $$

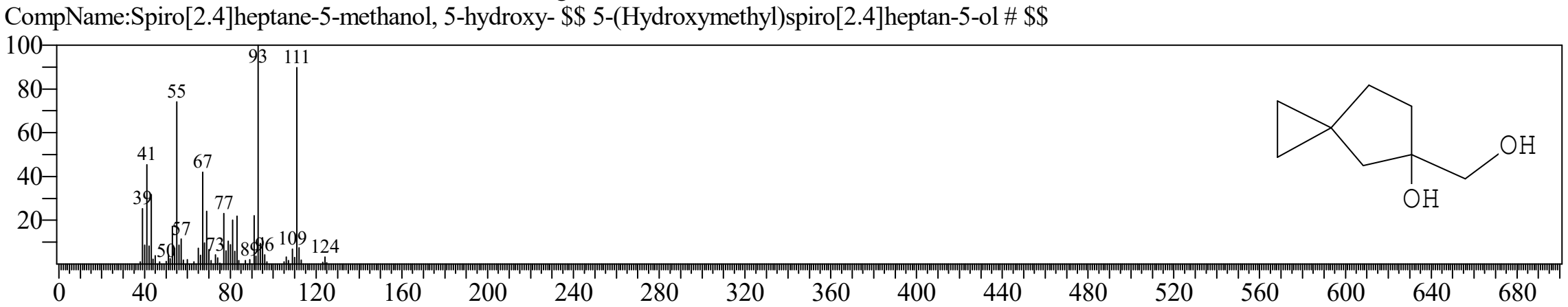

<< Target >>
Line#:56   R.Time:10.765(Scan#:1454) MassPeaks:45
RawMode:Averaged 10.760-10.770(1453-1455) BasePeak:43.00(3556)
BG Mode:Calc. from Peak Group 1 - Event 1 Scan

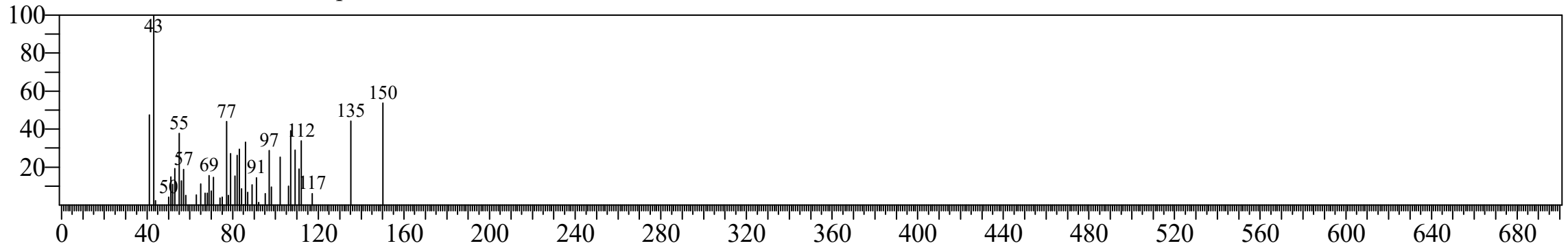

Hit#:1 Entry:62658 Library:WILEY8.LIB
SI:71 Formula:C10H16O2 CAS:0-00-0 MolWeight:168 RetIndex:0
CompName:(-)-NEPETALACTOL

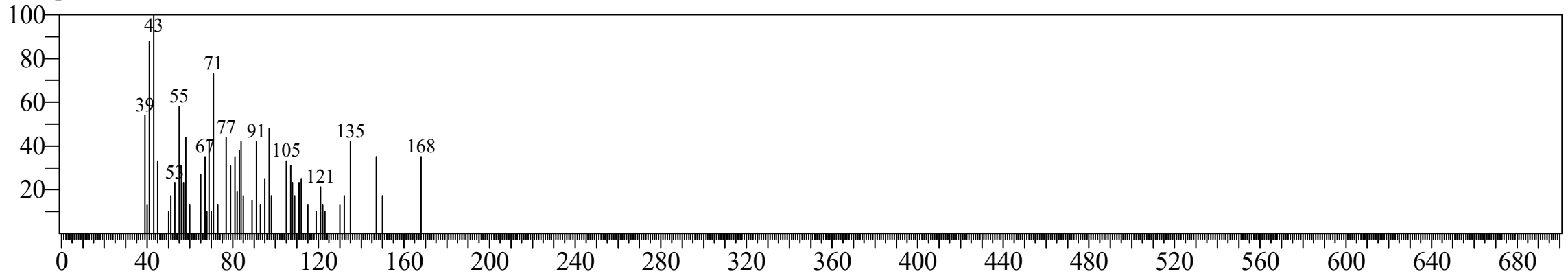

Hit#:2 Entry:63346 Library:WILEY8.LIB
SI:70 Formula:C11H20O CAS:0-00-0 MolWeight:168 RetIndex:0
CompName:5-ISOPROPYL-6-METHYL-HEPTA-3,5-DIEN-2-OL $$ 5-ISOPROPYL-6-METHYL-3,5-HEPTADIEN-2-OL

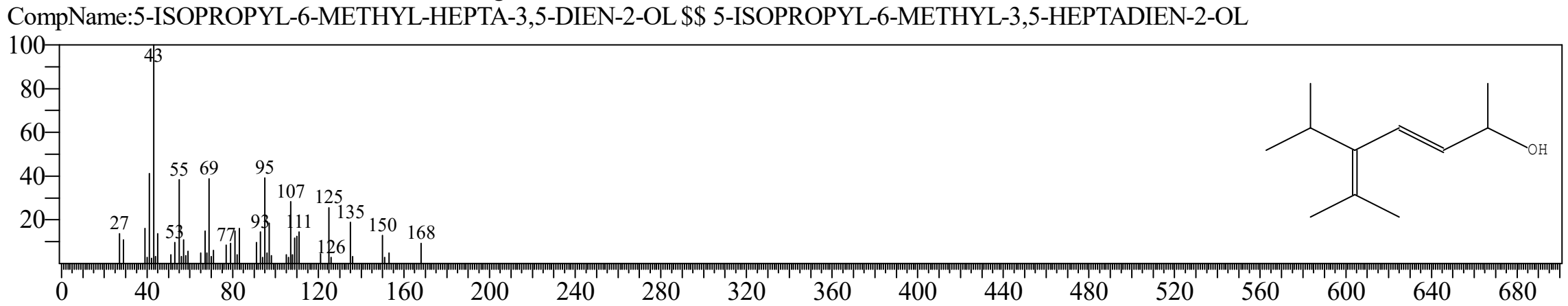

Hit#:3 Entry:25797 Library:NIST14.lib
SI:70 Formula:C11H20O CAS:0-00-0 MolWeight:168 RetIndex:1183
CompName:5-Isopropyl-6-methyl-hepta-3,5-dien-2-ol $$ (3E)-5-Isopropyl-6-methyl-3,5-heptadien-2-ol # $$

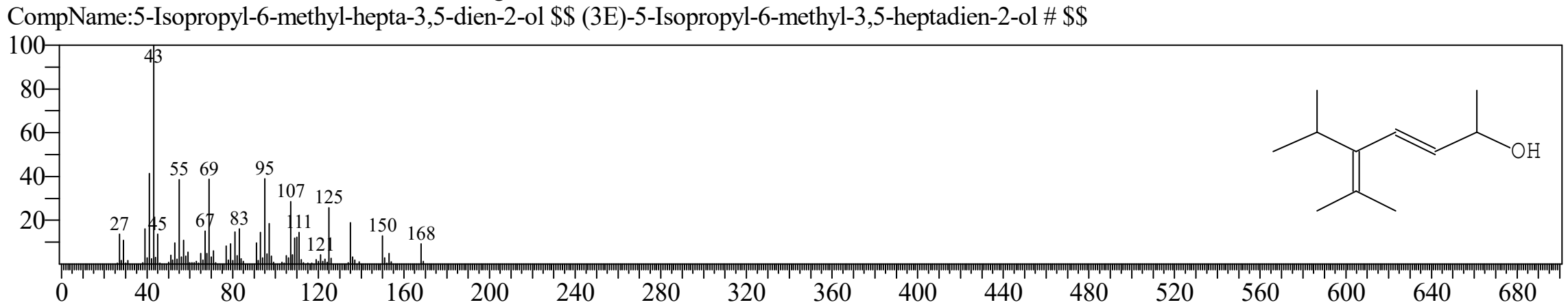

<< Target >>
Line#:57 R.Time:10.865(Scan#:1474) MassPeaks:72
RawMode:Averaged 10.860-10.870(1473-1475) BasePeak:43.00(34633)
BG Mode:Calc. from Peak Group 1 - Event 1 Scan

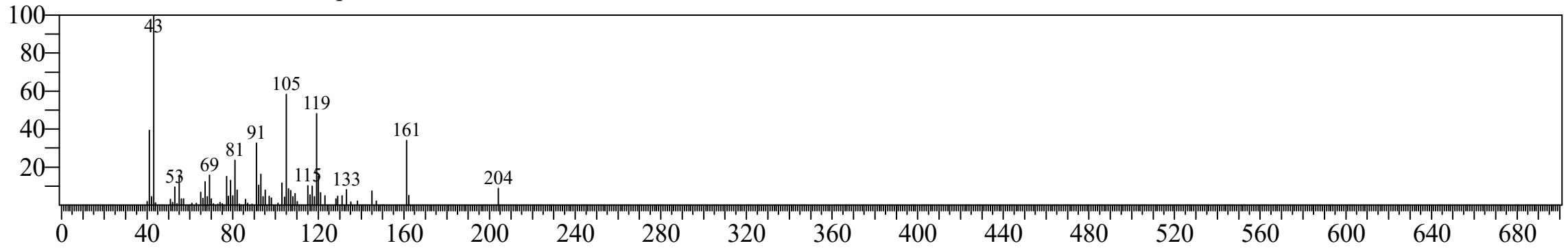

Hit#:1 Entry:19121 Library:NIST14s.lib
SI:83 Formula:C15H24 CAS:17699-14-8 MolWeight:204 RetIndex:1344
CompName:.alpha.-Cubebene $$ 1H-Cyclopenta[1,3]cyclopropa[1,2]benzene, 3a,3b,4,5,6,7-hexahydro-3,7-dimethyl-4-(1-methylethyl)-, [3aS-(3a.alpha.,3b.

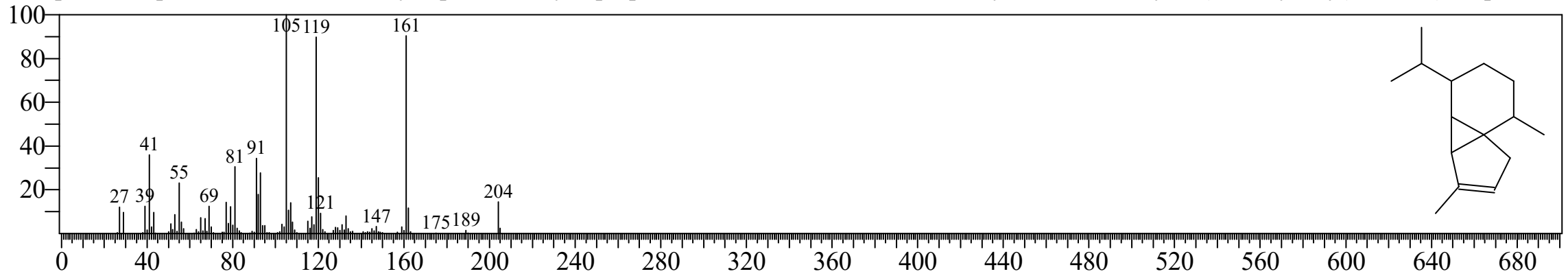

Hit#:2 Entry:110375 Library:WILEY8.LIB
SI:83 Formula:C15H24 CAS:0-00-0 MolWeight:204 RetIndex:0
CompName:4-ISOPROPYL-3,7-DIMETHYL-3A,3B,4,5,6,7-HEXAHYDRO-1H-CYCLOPENTA[2,3]CYCLOPROPA[1,2-A]BENZENE $$ ALPHA-CUE

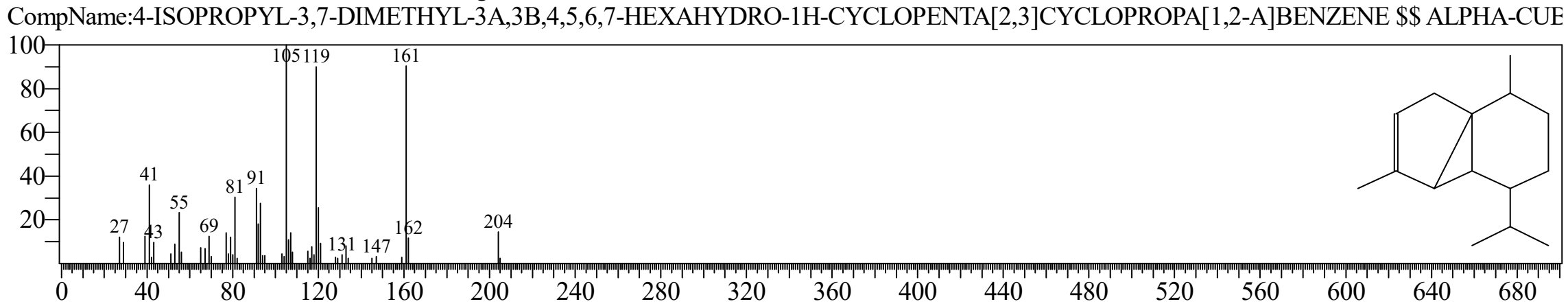

Hit#:3 Entry:19178 Library:NIST14s.lib
SI:83 Formula:C15H24 CAS:13744-15-5 MolWeight:204 RetIndex:1339
CompName:1H-Cyclopenta[1,3]cyclopropa[1,2]benzene, octahydro-7-methyl-3-methylene-4-(1-methylethyl)-, [3aS-(3a.alpha.,3b.beta.,4.beta.,7.alpha.,7aS*

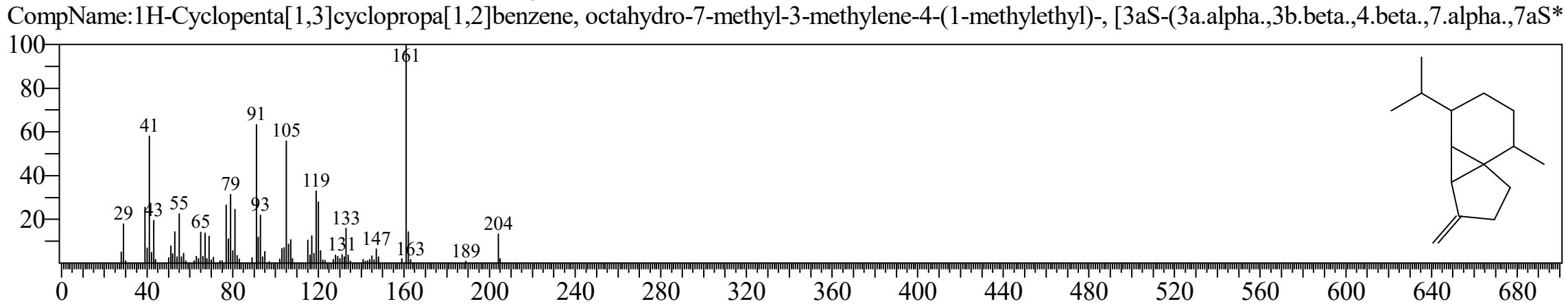

<< Target >>
Line#:58   R.Time:10.990(Scan#:1499) MassPeaks:39
RawMode:Averaged 10.985-10.995(1498-1500) BasePeak:43.00(40300)
BG Mode:Calc. from Peak Group 1 - Event 1 Scan

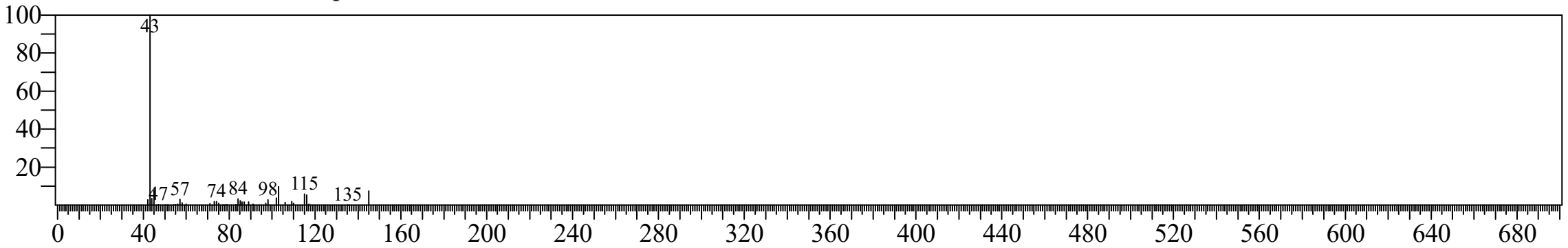

Hit#:1 Entry:128308 Library:WILEY8.LIB
SI:90 Formula:C9H14O6 CAS:102-76-1 MolWeight:218 RetIndex:0
CompName:1,2,3-PROPANETRIOL, TRIACETATE $$ 2-(ACETYLOXY)-1-[(ACETYLOXY)METHYL]ETHYL ACETATE # $$ 1,2,3-PROPANETRIOI

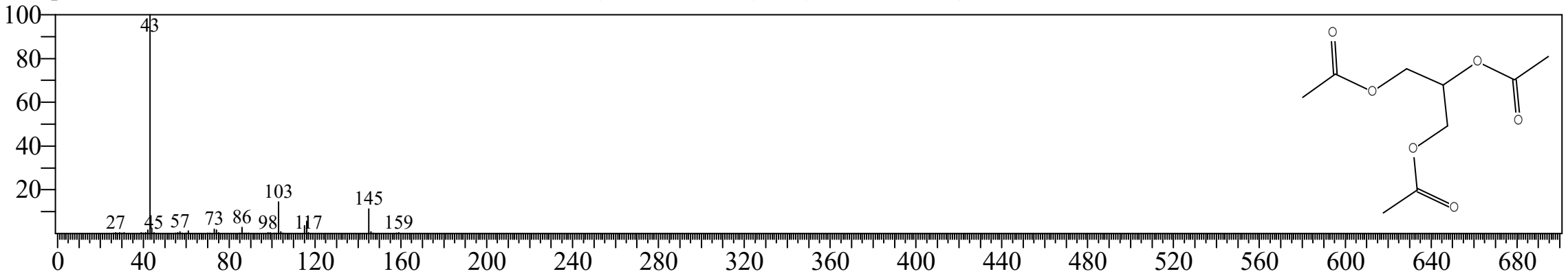

Hit#:2 Entry:20989 Library:NIST14s.lib
SI:90 Formula:C9H14O6 CAS:102-76-1 MolWeight:218 RetIndex:1354
CompName:Triacetin $$ 1,2,3-Propanetriol, triacetate $$ Acetin, tri- $$ Enzactin $$ Fungacetin $$ Glycerin triacetate $$ Glycerol triacetate $$ Glyceryl tria

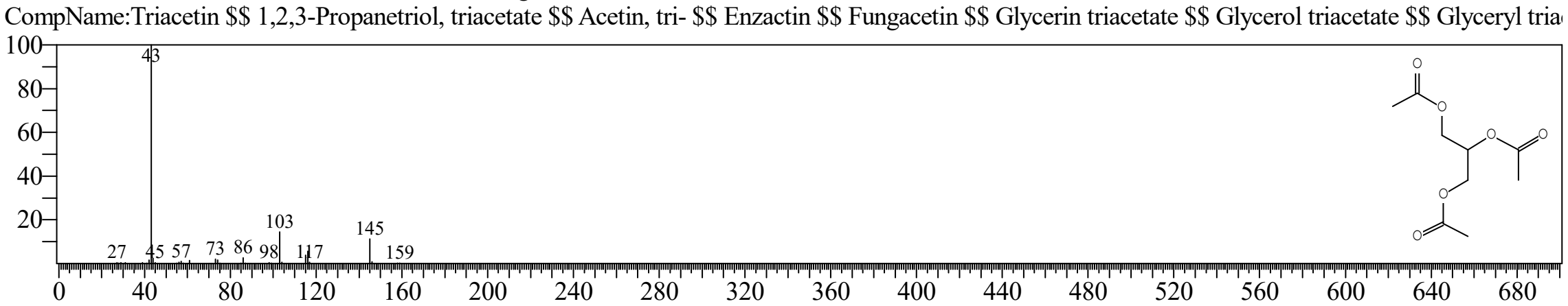

Hit#:3 Entry:60065 Library:NIST14.lib
SI:89 Formula:C9H14O6 CAS:102-76-1 MolWeight:218 RetIndex:1354
CompName:Triacetin $$ 1,2,3-Propanetriol, triacetate $$ Acetin, tri- $$ Enzactin $$ Fungacetin $$ Glycerin triacetate $$ Glycerol triacetate $$ Glyceryl tria

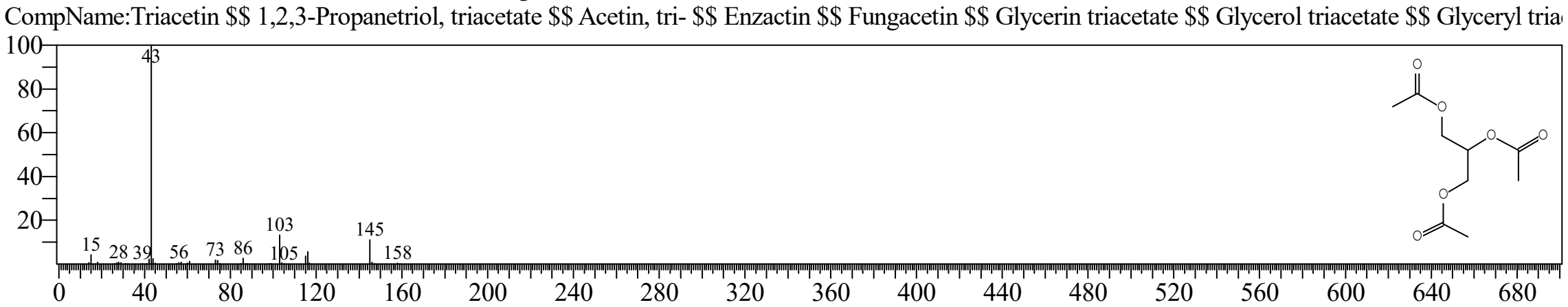

<< Target >>
Line#:59 R.Time:11.070(Scan#:1515) MassPeaks:68
RawMode:Averaged 11.065-11.075(1514-1516) BasePeak:105.05(18292)
BG Mode:Calc. from Peak Group 1 - Event 1 Scan

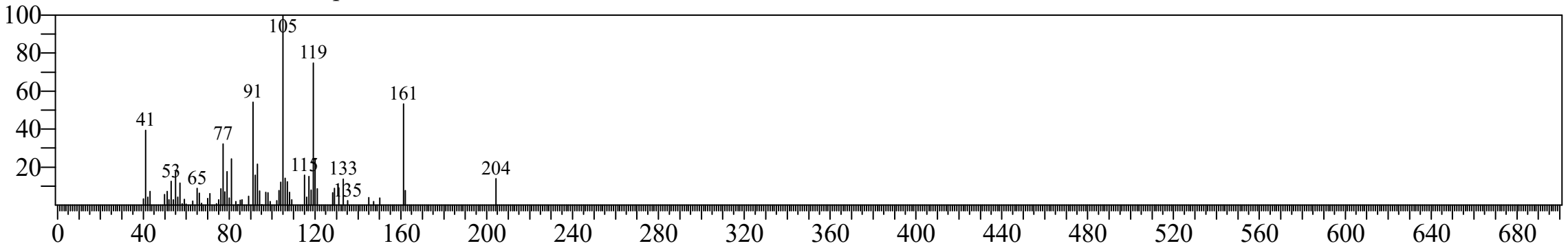

Hit#:1 Entry:110129 Library:WILEY8.LIB
SI:88 Formula:C15H24 CAS:17699-14-8 MolWeight:204 RetIndex:0
CompName:1H-CYCLOPENTA[1,3]CYCLOPROPA[1,2]BENZENE, 3A,3B,4,5,6,7-HEXAHYDRO-3,7-DIMETHYL-4-(1-METHYLETHYL)-, [3AS-(3A

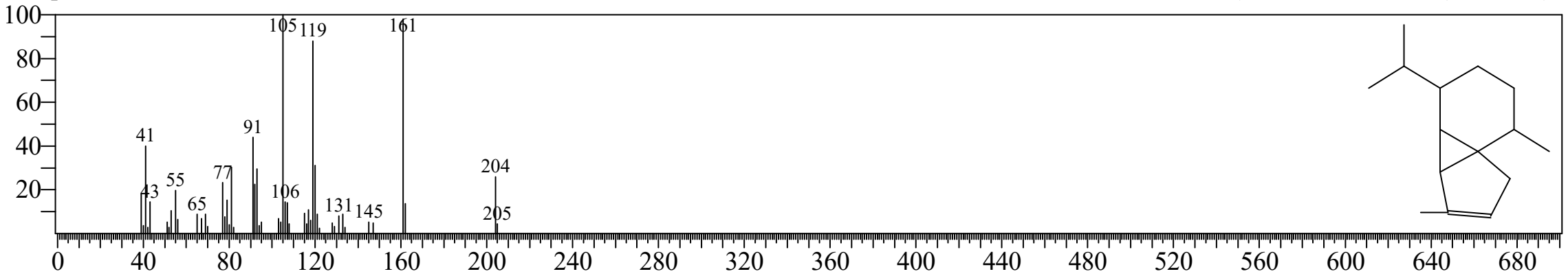

Hit#:2 Entry:110123 Library:WILEY8.LIB
SI:87 Formula:C15H24 CAS:3856-25-5 MolWeight:204 RetIndex:0
CompName:TRICYCLO[4.4.0.0(2,7)]DEC-3-ENE, 1,3-DIMETHYL-8-(1-METHYLETHYL)-, ST $$ TRICYCLO[4.4.0.0(2,7)]DEC-3-ENE, 1,3-DIMETI

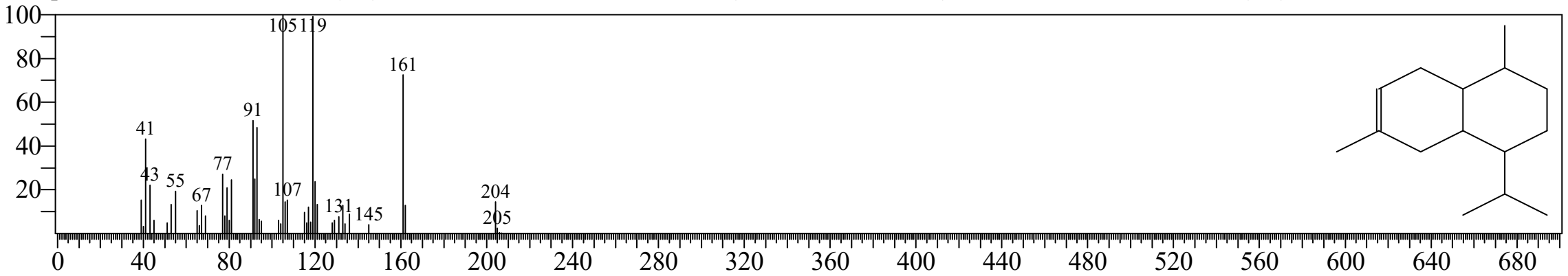

Hit#:3 Entry:110121 Library:WILEY8.LIB
SI:87 Formula:C15H24 CAS:3856-25-5 MolWeight:204 RetIndex:0
CompName:TRICYCLO[4.4.0.0(2,7)]DEC-3-ENE, 1,3-DIMETHYL-8-(1-METHYLETHYL)-, ST $$ TRICYCLO[4.4.0.0(2,7)]DEC-3-ENE, 1,3-DIMETI

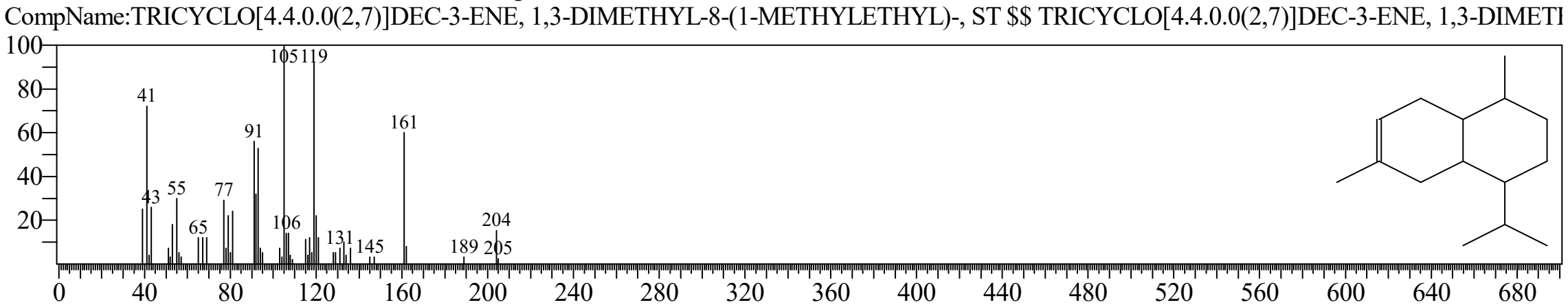

<< Target >>
Line#:60   R.Time:11.215(Scan#:1544) MassPeaks:66
RawMode:Averaged 11.210-11.220(1543-1545) BasePeak:91.05(5902)
BG Mode:Calc. from Peak Group 1 - Event 1 Scan

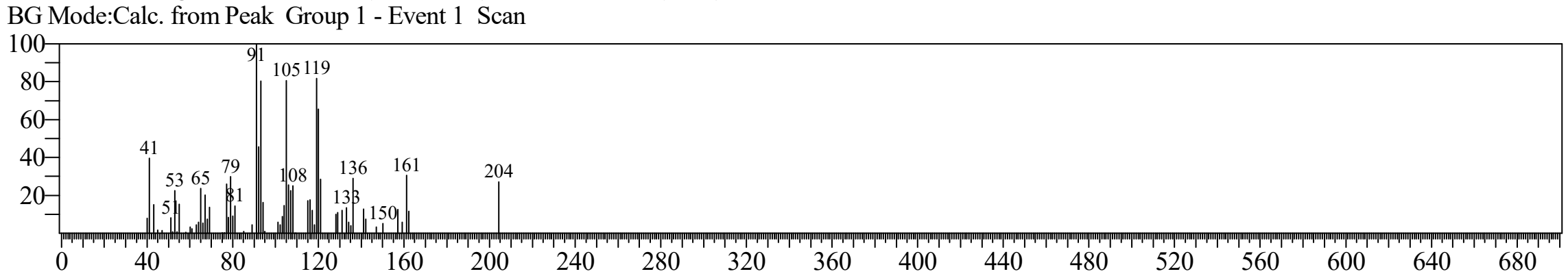

Hit#:1 Entry:49912 Library:NIST14.lib
SI:86 Formula:C15H24 CAS:0-00-0 MolWeight:204 RetIndex:1221
CompName:.alpha.-ylangene

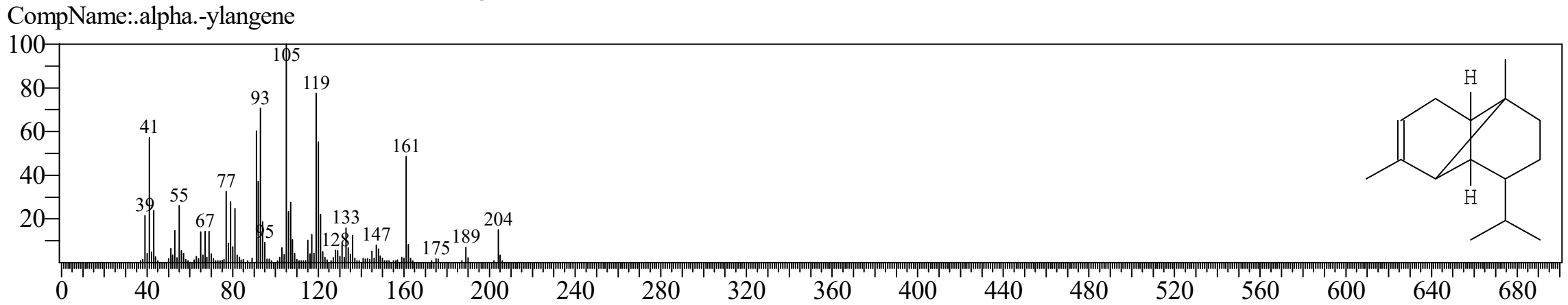

Hit#:2 Entry:49913 Library:NIST14.lib
SI:85 Formula:C15H24 CAS:14912-44-8 MolWeight:204 RetIndex:1221
CompName:Ylangene $$ Tricyclo[4.4.0.0(2,7)]dec-3-ene, 8-isopropyl-1,3-dimethyl-, (1S,2R,6R,7R,8S)-(+)- $$ (1S,2R,6R,7R,8S)-8-Isopropyl-1,3-dimethy

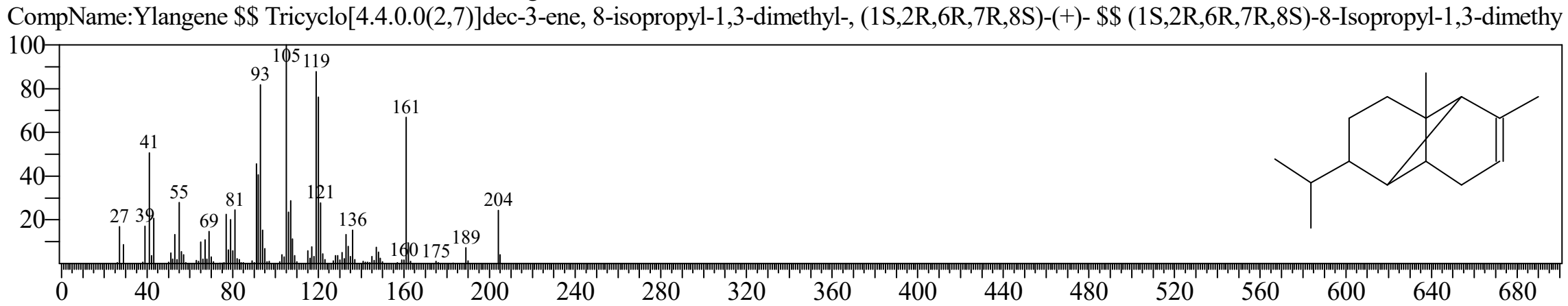

Hit#:3 Entry:110112 Library:WILEY8.LIB
SI:85 Formula:C15H24 CAS:14912-44-8 MolWeight:204 RetIndex:0
CompName:TRICYCLO[4.4.0.0(2,7)]DEC-3-ENE, 1,3-DIMETHYL-8-(1-METHYLETHYL)-, STEREOISOMER $$ TRICYCLO[4.4.0.02,7]DEC-3-ENE

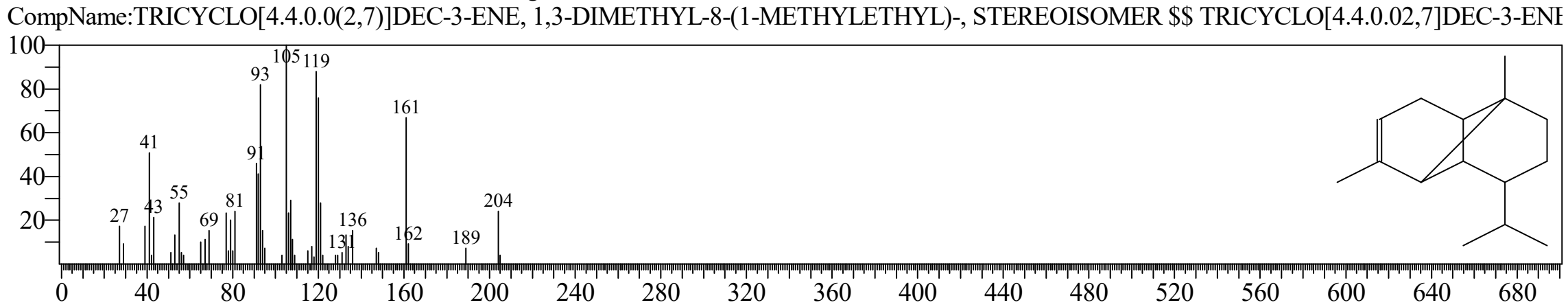

<< Target >>
Line#:61   R.Time:11.280(Scan#:1557) MassPeaks:107
RawMode:Averaged 11.275-11.285(1556-1558) BasePeak:105.10(166296)
BG Mode:Calc. from Peak Group 1 - Event 1 Scan

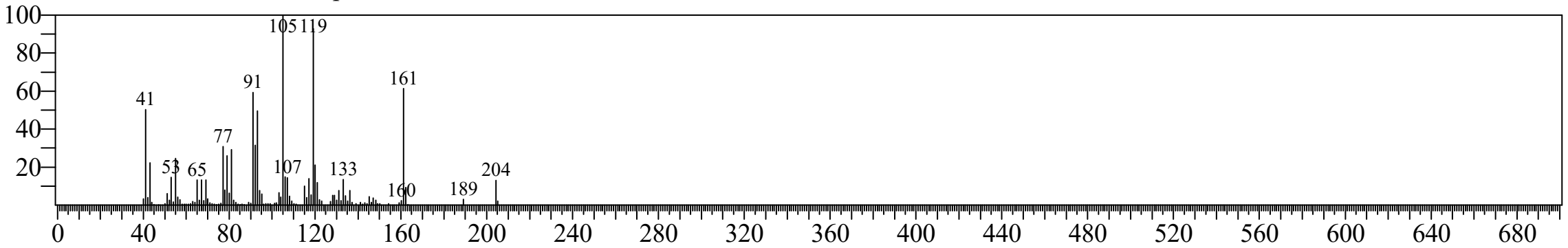

Hit#:1 Entry:110121 Library:WILEY8.LIB
SI:97 Formula:C15H24 CAS:3856-25-5 MolWeight:204 RetIndex:0
CompName:TRICYCLO[4.4.0.0(2,7)]DEC-3-ENE, 1,3-DIMETHYL-8-(1-METHYLETHYL)-, ST $$ TRICYCLO[4.4.0.0(2,7)]DEC-3-ENE, 1,3-DIMETI

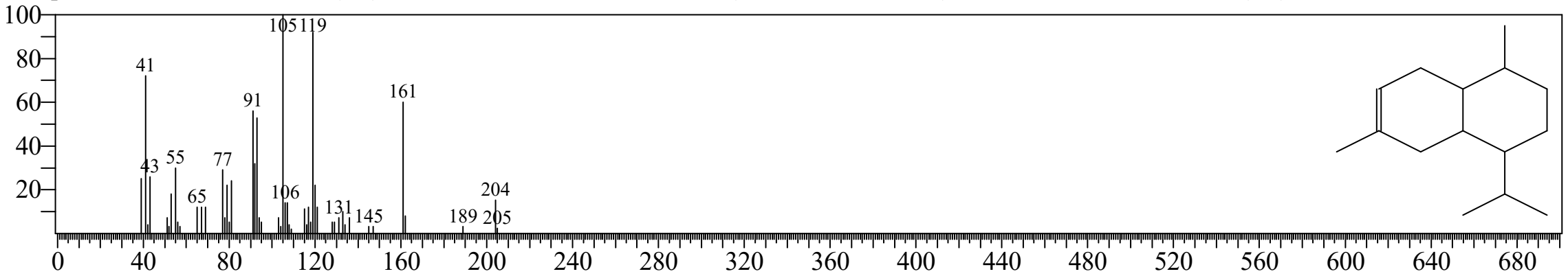

Hit#:2 Entry:110123 Library:WILEY8.LIB
SI:95 Formula:C15H24 CAS:3856-25-5 MolWeight:204 RetIndex:0
CompName:TRICYCLO[4.4.0.0(2,7)]DEC-3-ENE, 1,3-DIMETHYL-8-(1-METHYLETHYL)-, ST $$ TRICYCLO[4.4.0.0(2,7)]DEC-3-ENE, 1,3-DIMETI

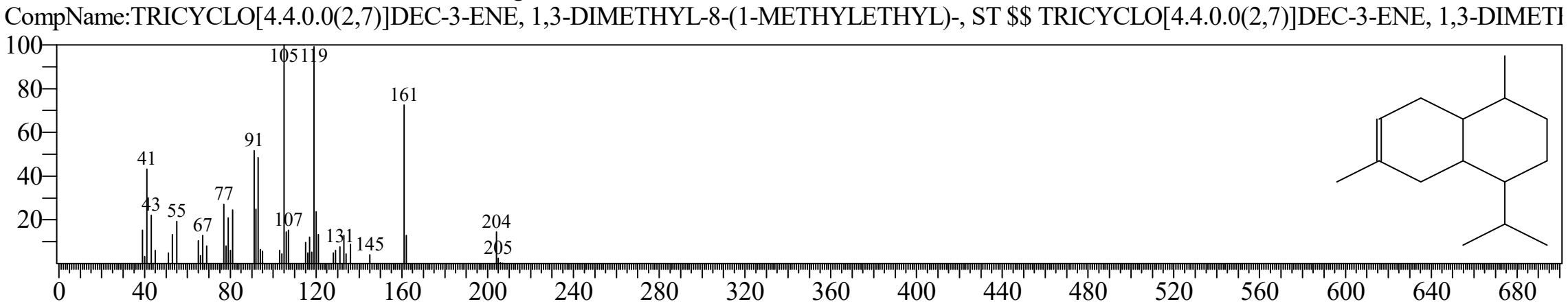

Hit#:3 Entry:19149 Library:NIST14s.lib
SI:95 Formula:C15H24 CAS:3856-25-5 MolWeight:204 RetIndex:1221
CompName:Copaene $$ Tricyclo[4.4.0.02,7]dec-3-ene, 1,3-dimethyl-8-(1-methylethyl)-, stereoisomer $$ Tricyclo[4.4.0.02,7]dec-3-ene, 8-isopropyl-1,3-din

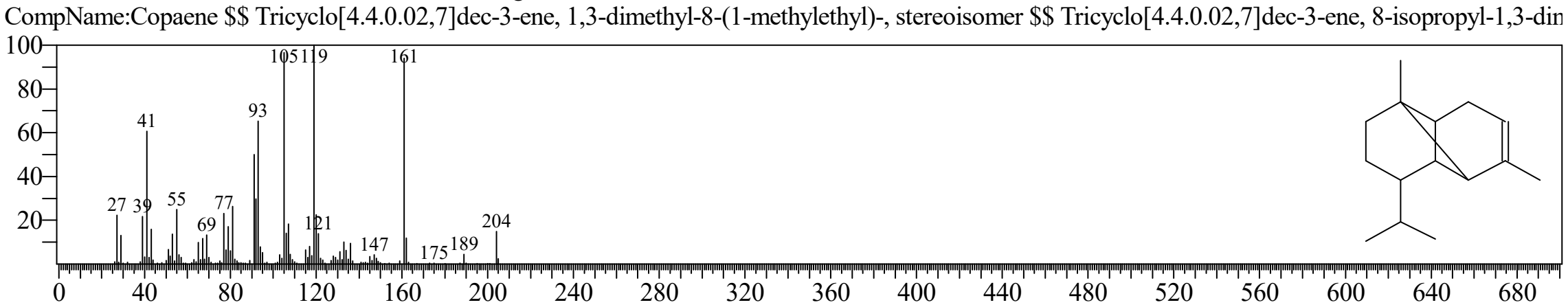

<< Target >>
Line#:62   R.Time:11.395(Scan#:1580) MassPeaks:94
RawMode:Averaged 11.390-11.400(1579-1581) BasePeak:41.05(151765)
BG Mode:Calc. from Peak Group 1 - Event 1 Scan

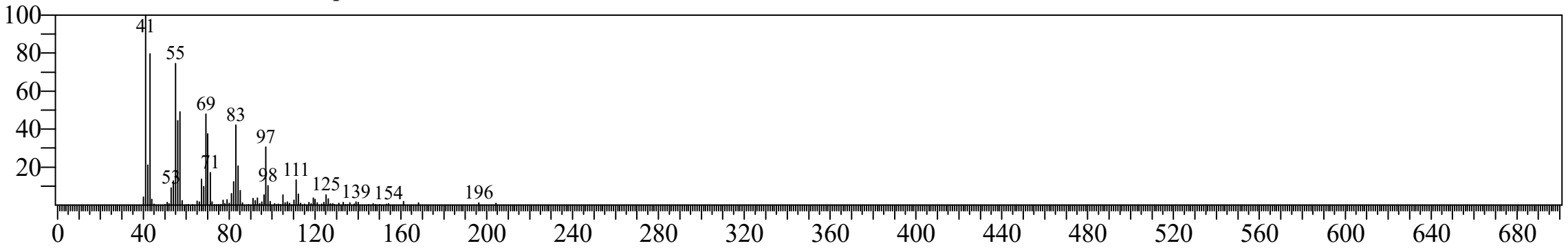

Hit#:1 Entry:99250 Library:WILEY8.LIB
SI:96 Formula:C14H28 CAS:1120-36-1 MolWeight:196 RetIndex:0
CompName:1-TETRADECENE $$ TETRADECANE $$ TETRADEC-1-ENE $$ .ALPHA.-TETRADECENE $$ 1-BUTADECENE $$ 1-TETRADECYL

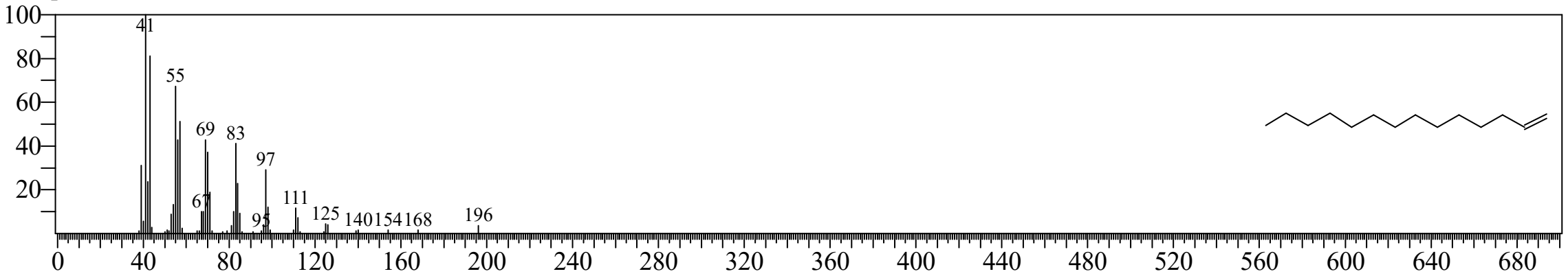

Hit#:2 Entry:80752 Library:WILEY8.LIB
SI:94 Formula:C13H26 CAS:2437-56-1 MolWeight:182 RetIndex:0
CompName:1-TRIDECENE $$ .ALPHA.-TRIDECENE $$ 1-C13H26 $$ 2-CYCLOHEXYL-N1,N1,N4,N4-TETRAISOBUTYL-3-METHYL-SUCCINAM

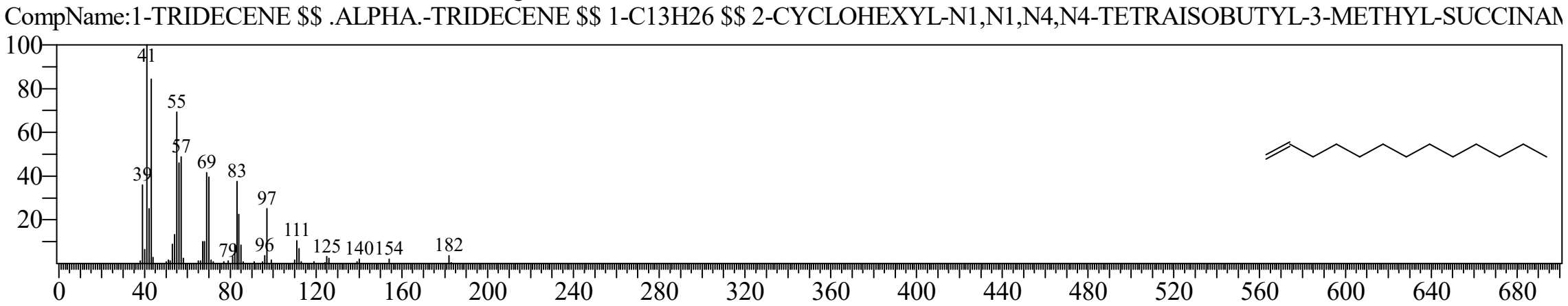

Hit#:3 Entry:17865 Library:NIST14s.lib
SI:94 Formula:C14H28 CAS:41446-68-8 MolWeight:196 RetIndex:1421
CompName:3-Tetradecene, (E)- $$ (3E)-3-Tetradecene $$ (E)-3-Tetradecene $$ trans-3-Tetradecene $$

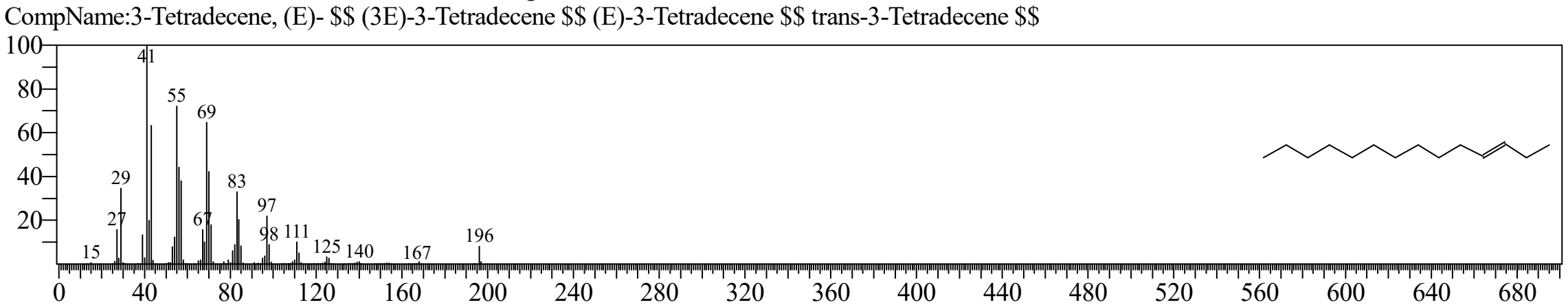

<< Target >>
Line#:63 R.Time:11.455(Scan#:1592) MassPeaks:98
RawMode:Averaged 11.450-11.460(1591-1593) BasePeak:105.10(128172)
BG Mode:Calc. from Peak Group 1 - Event 1 Scan

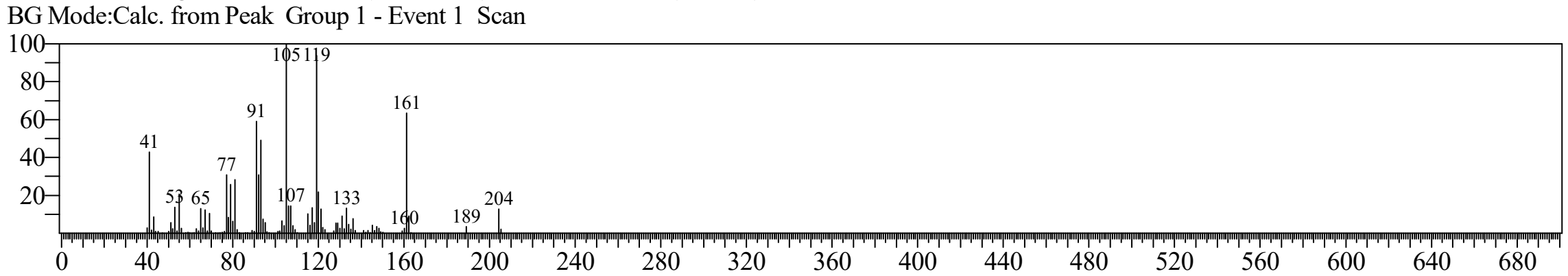

Hit#:1 Entry:110123 Library:WILEY8.LIB
SI:96 Formula:C15H24 CAS:3856-25-5 MolWeight:204 RetIndex:0
CompName:TRICYCLO[4.4.0.0(2,7)]DEC-3-ENE, 1,3-DIMETHYL-8-(1-METHYLETHYL)-, ST $$ TRICYCLO[4.4.0.0(2,7)]DEC-3-ENE, 1,3-DIMETI

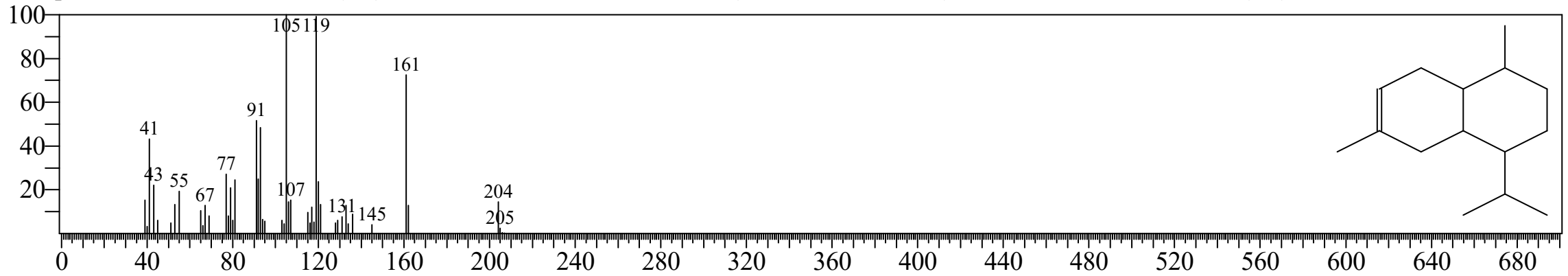

Hit#:2 Entry:110121 Library:WILEY8.LIB
SI:95 Formula:C15H24 CAS:3856-25-5 MolWeight:204 RetIndex:0
CompName:TRICYCLO[4.4.0.0(2,7)]DEC-3-ENE, 1,3-DIMETHYL-8-(1-METHYLETHYL)-, ST $$ TRICYCLO[4.4.0.0(2,7)]DEC-3-ENE, 1,3-DIMETI

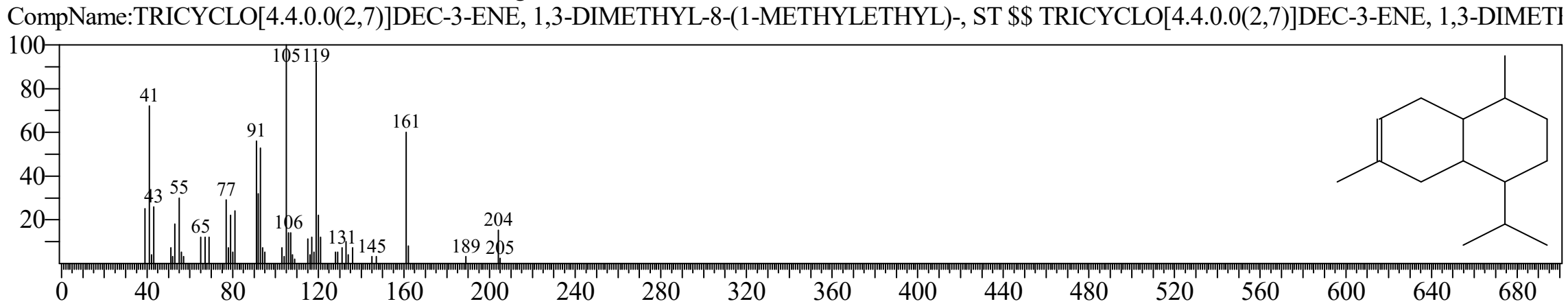

Hit#:3 Entry:110119 Library:WILEY8.LIB
SI:94 Formula:C15H24 CAS:3856-25-5 MolWeight:204 RetIndex:0
CompName:TRICYCLO[4.4.0.0(2,7)]DEC-3-ENE, 1,3-DIMETHYL-8-(1-METHYLETHYL)-, ST $$ TRICYCLO[4.4.0.0(2,7)]DEC-3-ENE, 1,3-DIMETI

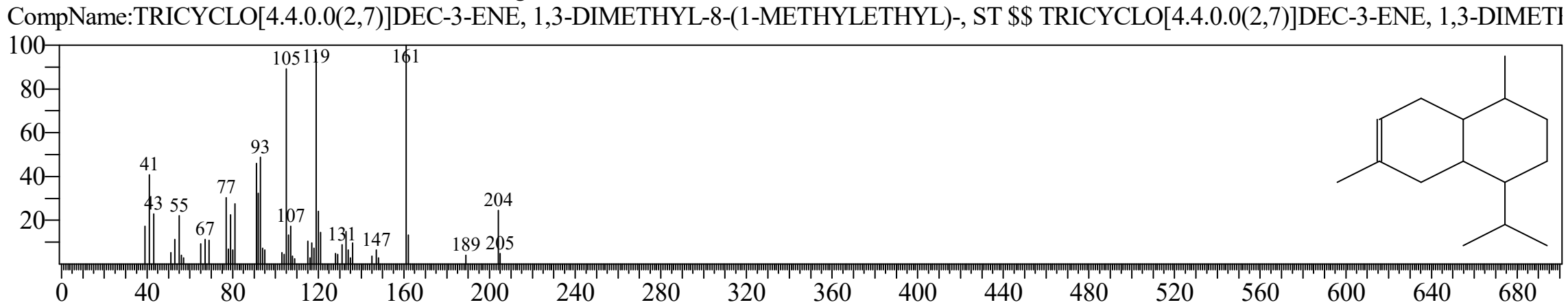

<< Target >>
Line#:64 R.Time:11.520(Scan#:1605) MassPeaks:58
RawMode:Averaged 11.515-11.525(1604-1606) BasePeak:41.05(113962)
BG Mode:Calc. from Peak Group 1 - Event 1 Scan

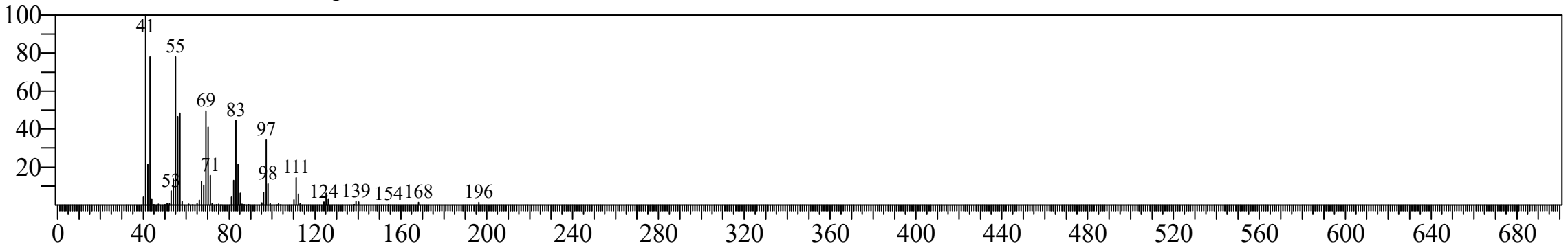

Hit#:1 Entry:99250 Library:WILEY8.LIB
SI:97 Formula:C14H28 CAS:1120-36-1 MolWeight:196 RetIndex:0
CompName:1-TETRADECENE $$ TETRADECANE $$ TETRADEC-1-ENE $$ .ALPHA.-TETRADECENE $$ 1-BUTADECENE $$ 1-TETRADECYL

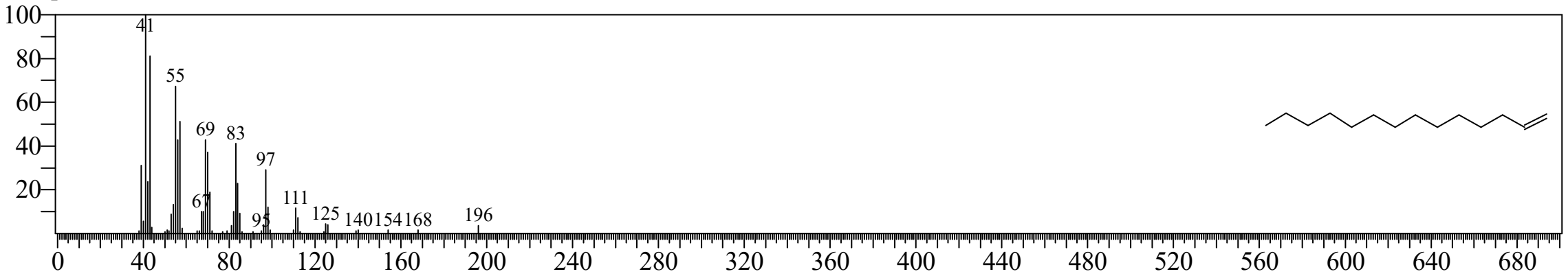

Hit#:2 Entry:119241 Library:WILEY8.LIB
SI:96 Formula:C15H30 CAS:13360-61-7 MolWeight:210 RetIndex:0
CompName:1-PENTADECENE $$ PENTADEC-1-ENE $$ CCRIS 5721 $$ EINECS 236-414-5 $$ NSC 77125 $$ PENTADECENE,1-

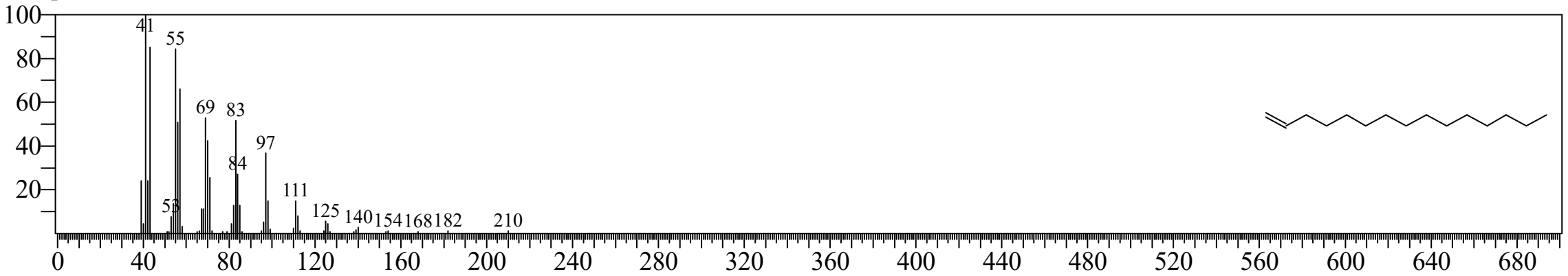

Hit#:3 Entry:139512 Library:WILEY8.LIB
SI:95 Formula:C16H32 CAS:629-73-2 MolWeight:224 RetIndex:0
CompName:1-HEXADECENE $$ HEXADEC-1-ENE $$ .ALPHA.-HEXADECENE $$ 1-CETENE $$ 1-N-HEXADECENE $$ AI3-06556 $$ ALPHA-H

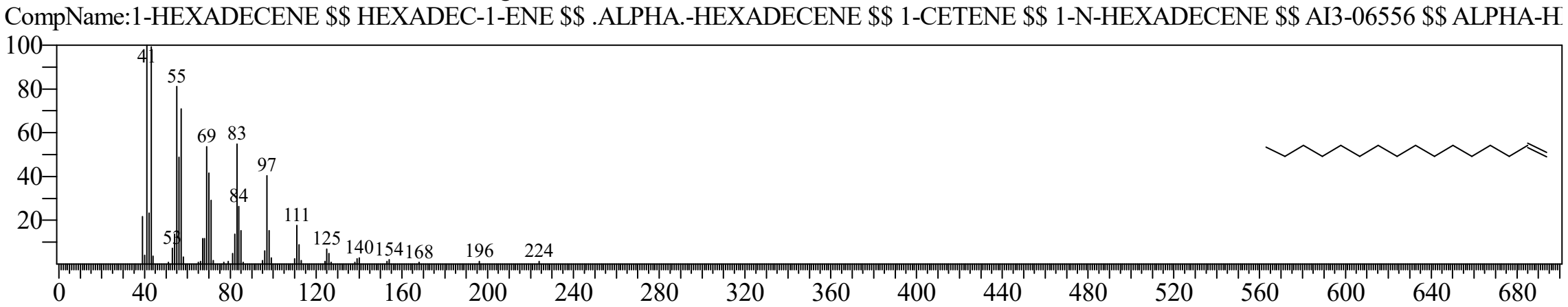

<< Target >>
Line#:65 R.Time:11.625(Scan#:1626) MassPeaks:51
RawMode:Averaged 11.620-11.630(1625-1627) BasePeak:43.05(40825)
BG Mode:Calc. from Peak Group 1 - Event 1 Scan

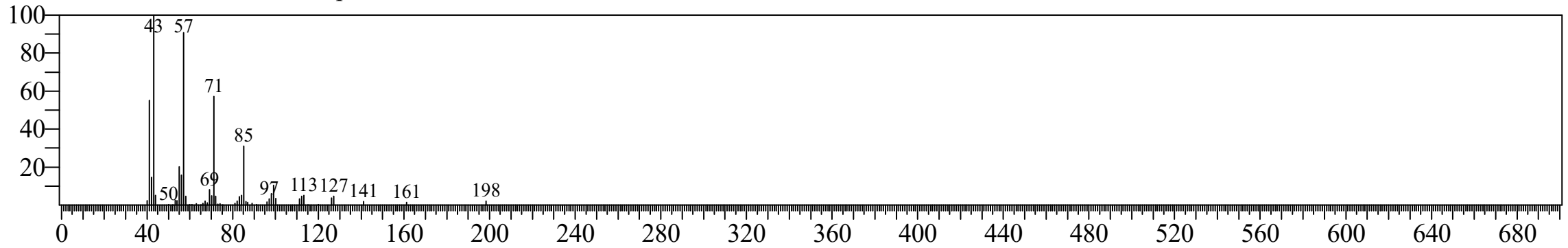

Hit#:1 Entry:102055 Library:WILEY8.LIB
SI:95 Formula:C14H30 CAS:629-59-4 MolWeight:198 RetIndex:0
CompName:TETRADECANE $$ AI3-04240 $$ BRN 1733859 $$ CCRIS 715 $$ EINECS 211-096-0 $$ HSDB 5728 $$ ISOTETRADECANE $$ N-TET

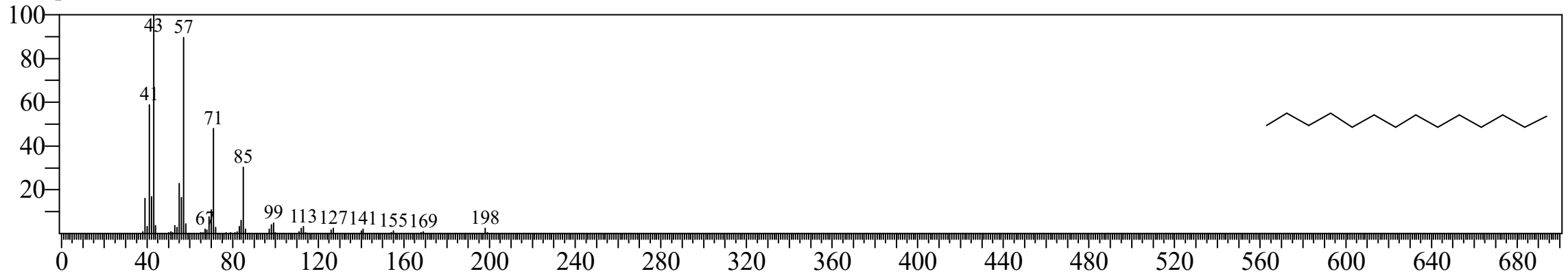

Hit#:2 Entry:102051 Library:WILEY8.LIB
SI:95 Formula:C14H30 CAS:629-59-4 MolWeight:198 RetIndex:0
CompName:TETRADECANE $$ AI3-04240 $$ BRN 1733859 $$ CCRIS 715 $$ EINECS 211-096-0 $$ HSDB 5728 $$ ISOTETRADECANE $$ N-TET

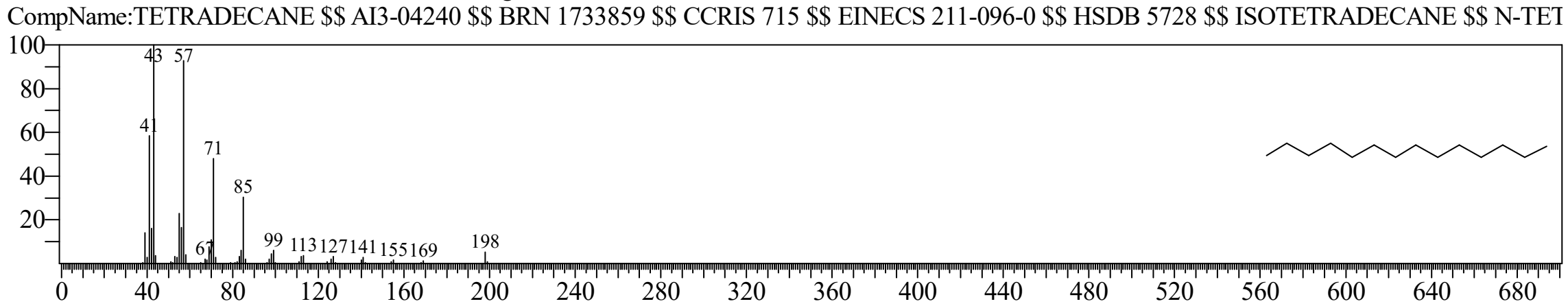

Hit#:3 Entry:102060 Library:WILEY8.LIB
SI:95 Formula:C14H30 CAS:629-59-4 MolWeight:198 RetIndex:0
CompName:TETRADECANE $$ AI3-04240 $$ BRN 1733859 $$ CCRIS 715 $$ EINECS 211-096-0 $$ HSDB 5728 $$ ISOTETRADECANE $$ N-TET

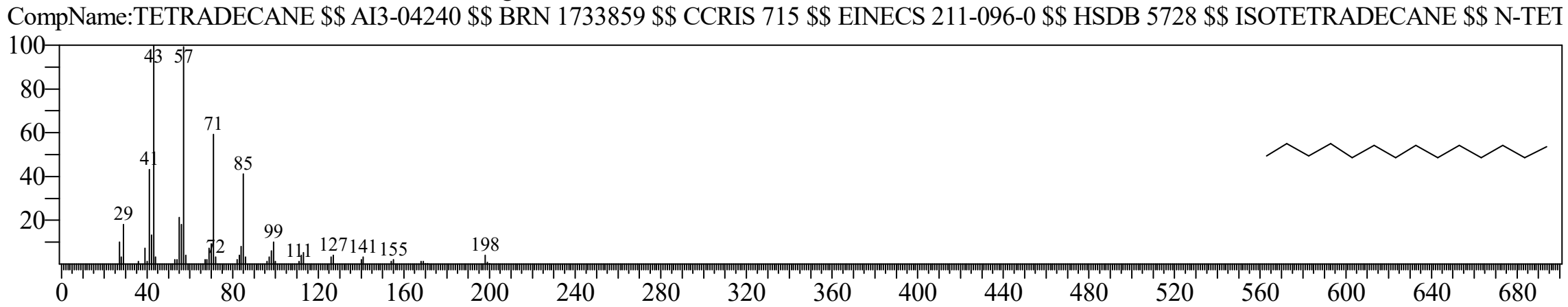

<< Target >>
Line#:66   R.Time:11.775(Scan#:1656) MassPeaks:98
RawMode:Averaged 11.770-11.780(1655-1657) BasePeak:105.10(46996)
BG Mode:Calc. from Peak Group 1 - Event 1 Scan

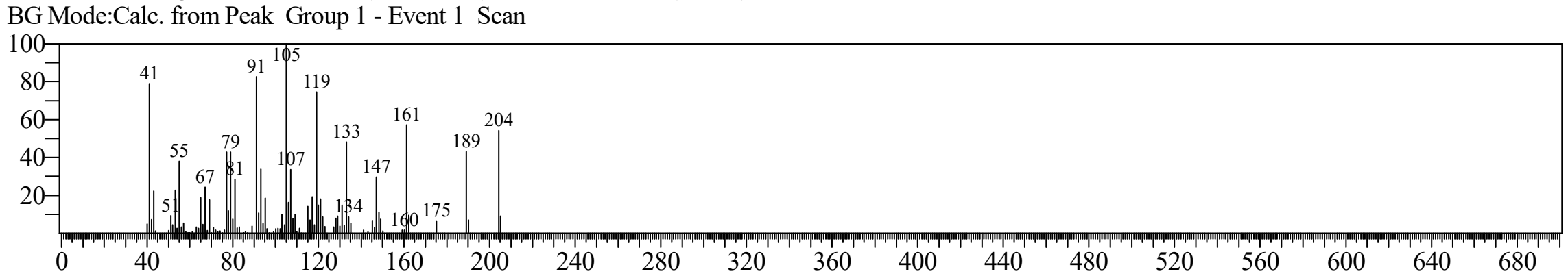

Hit#:1 Entry:19049 Library:NIST14s.lib
SI:94 Formula:C15H24 CAS:489-40-7 MolWeight:204 RetIndex:1419
CompName:1H-Cycloprop[e]azulene, 1a,2,3,4,4a,5,6,7b-octahydro-1,1,4,7-tetramethyl-, [1aR-(1a.alpha.,4.alpha.,4a.beta.,7b.alpha.)]- $$ 1H-Cycloprop[e]a:

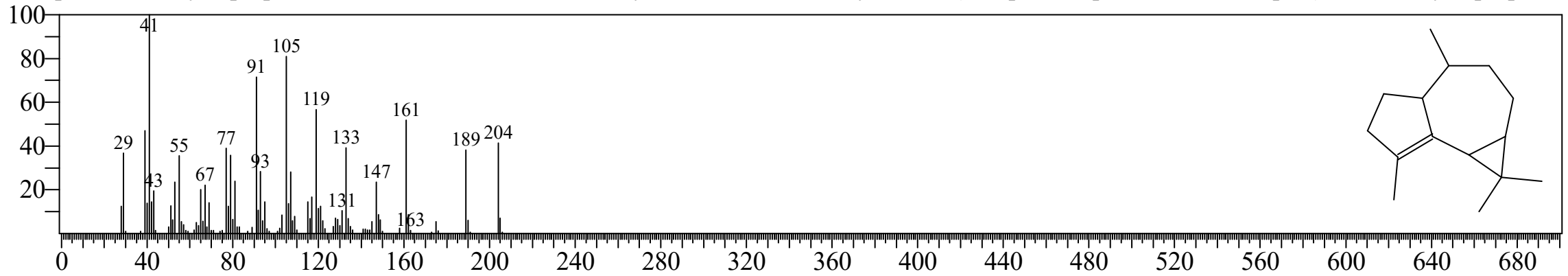

Hit#:2 Entry:110056 Library:WILEY8.LIB
SI:94 Formula:C15H24 CAS:489-40-7 MolWeight:204 RetIndex:0
CompName:1H-CYCLOPROP[E]AZULENE, 1A,2,3,4,4A,5,6,7B-OCTAHYDRO-1,1,4,7-TETRAMETHYL-, [1AR-(1A.ALPHA.,4.ALPHA.,4A.BETA.,7

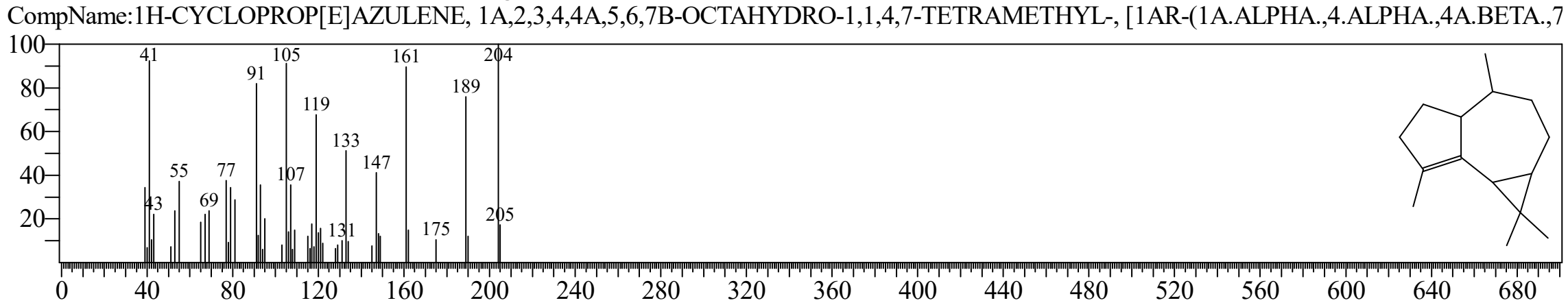

Hit#:3 Entry:110055 Library:WILEY8.LIB
SI:93 Formula:C15H24 CAS:489-40-7 MolWeight:204 RetIndex:0
CompName:1H-CYCLOPROP[E]AZULENE, 1A,2,3,4,4A,5,6,7B-OCTAHYDRO-1,1,4,7-TETRAMETHYL-, [1AR-(1A.ALPHA.,4.ALPHA.,4A.BETA.,7

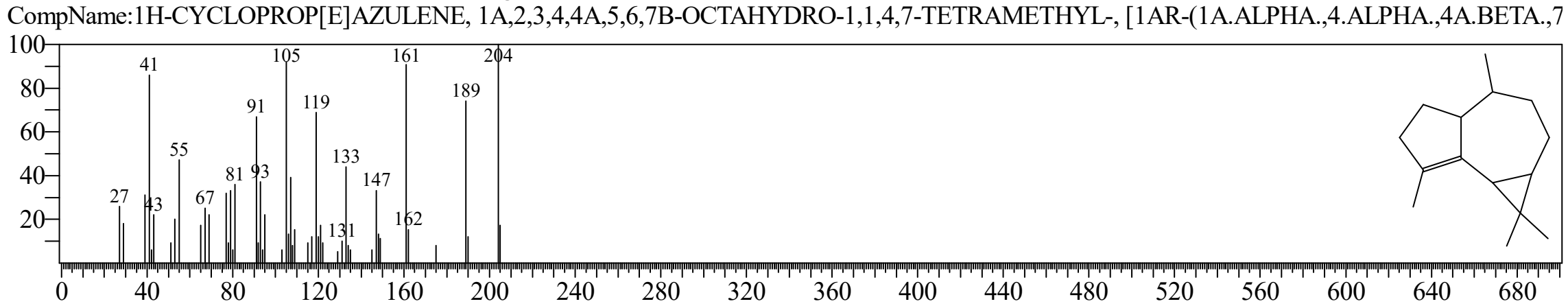

<< Target >>
Line#:67   R.Time:11.945(Scan#:1690) MassPeaks:140
RawMode:Averaged 11.940-11.950(1689-1691) BasePeak:41.05(2842862)
BG Mode:Calc. from Peak Group 1 - Event 1 Scan

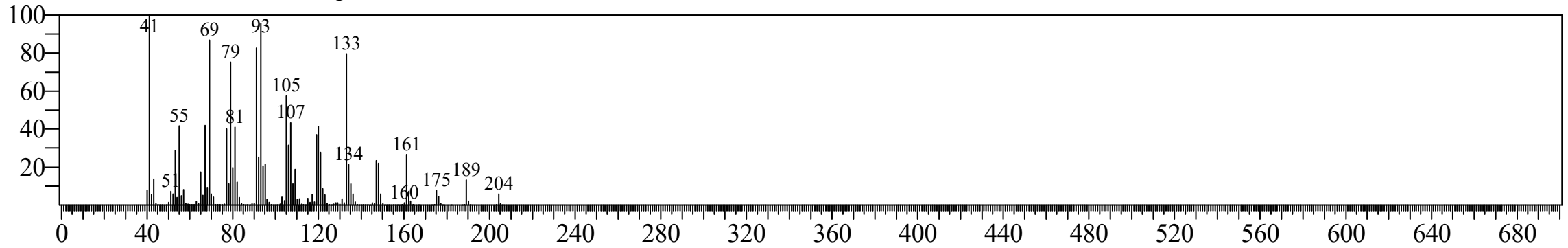

Hit#:1 Entry:49890 Library:NIST14.lib
SI:96 Formula:C15H24 CAS:87-44-5 MolWeight:204 RetIndex:1494
CompName:Caryophyllene $$ Bicyclo[7.2.0]undec-4-ene, 4,11,11-trimethyl-8-methylene-, [1R-(1R*,4E,9S*)]- $$ Bicyclo[7.2.0]undec-4-ene, 4,11,11-trime

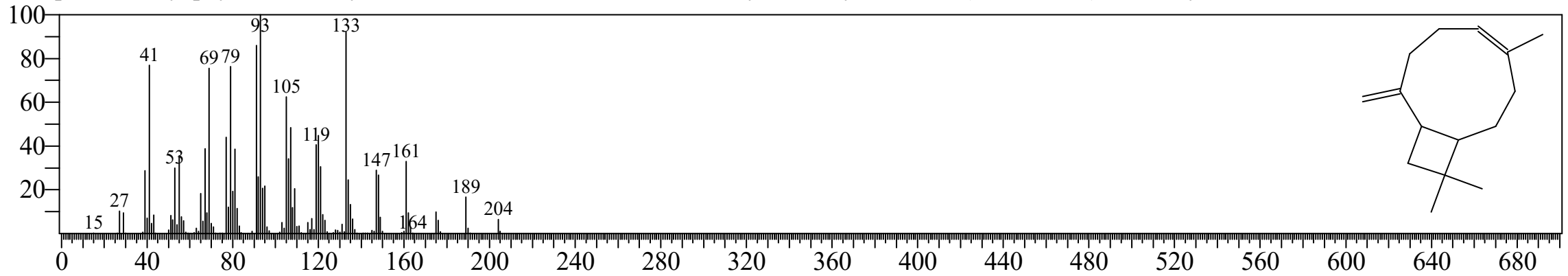

Hit#:2 Entry:109821 Library:WILEY8.LIB
SI:96 Formula:C15H24 CAS:87-44-5 MolWeight:204 RetIndex:0
CompName:BICYCLO[7.2.0]UNDEC-4-ENE, 4,11,11-TRIMETHYL-8-METHYLENE-, [1R-(1R*,4E,9S*)]- $$ 2,6,10,10-TETRAMETHYLBICYCLO[7

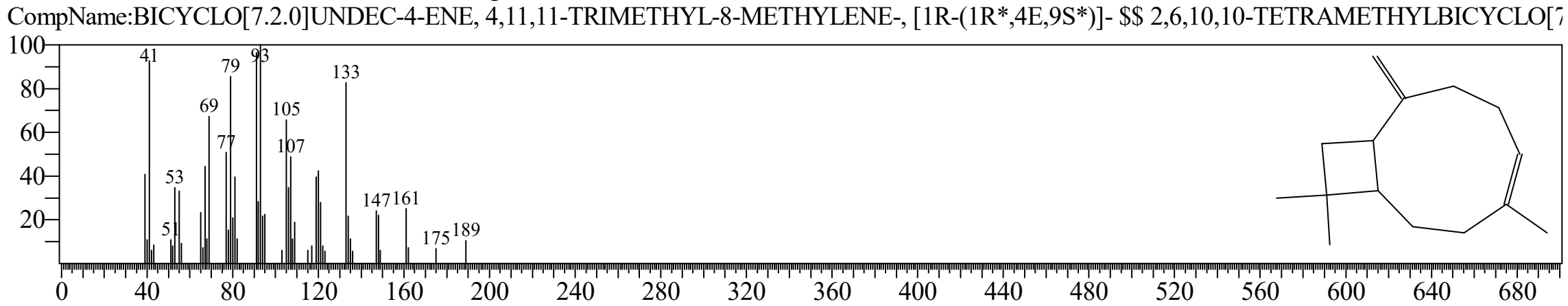

Hit#:3 Entry:110300 Library:WILEY8.LIB
SI:96 Formula:C15H24 CAS:0-00-0 MolWeight:204 RetIndex:0
CompName:TRANS(.BETA.)-CARYOPHYLLENE

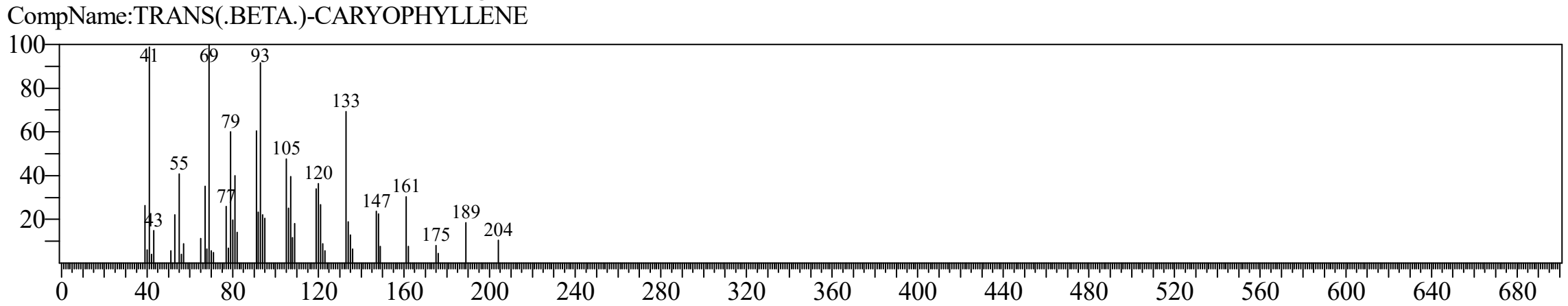

<< Target >>
Line#:68   R.Time:12.080(Scan#:1717) MassPeaks:143
RawMode:Averaged 12.075-12.085(1716-1718) BasePeak:41.05(2657869)
BG Mode:Calc. from Peak Group 1 - Event 1 Scan

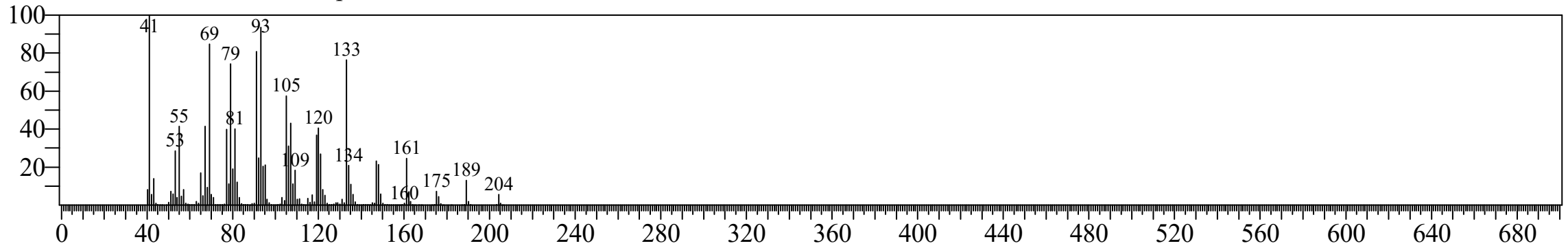

Hit#:1 Entry:49890 Library:NIST14.lib
SI:96 Formula:C15H24 CAS:87-44-5 MolWeight:204 RetIndex:1494
CompName:Caryophyllene $$ Bicyclo[7.2.0]undec-4-ene, 4,11,11-trimethyl-8-methylene-, [1R-(1R*,4E,9S*)]- $$ Bicyclo[7.2.0]undec-4-ene, 4,11,11-trime

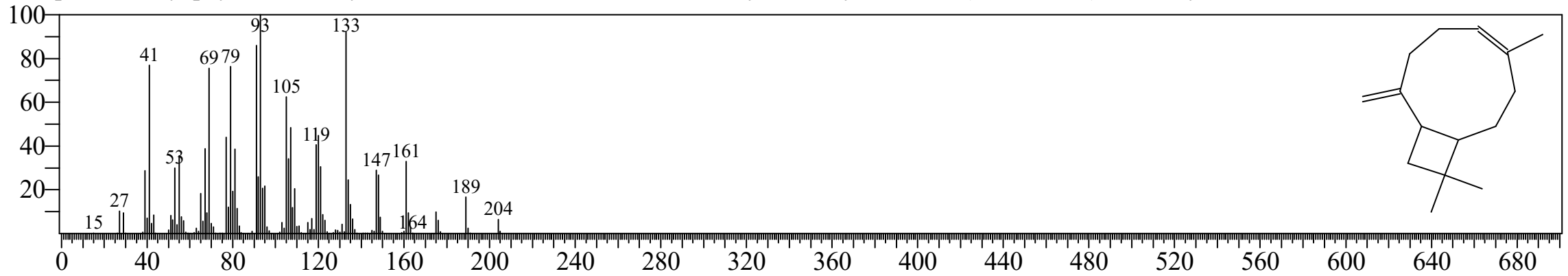

Hit#:2 Entry:110300 Library:WILEY8.LIB
SI:96 Formula:C15H24 CAS:0-00-0 MolWeight:204 RetIndex:0
CompName:TRANS(.BETA.)-CARYOPHYLLENE

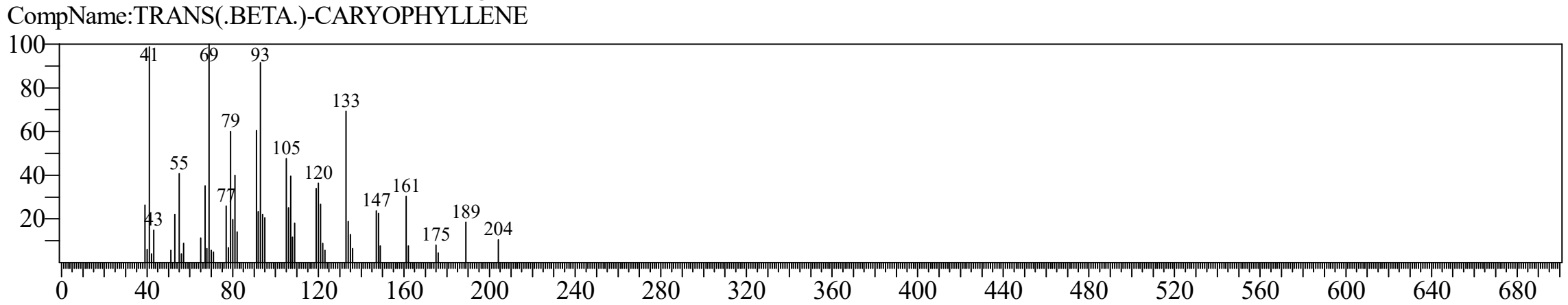

Hit#:3 Entry:109821 Library:WILEY8.LIB
SI:96 Formula:C15H24 CAS:87-44-5 MolWeight:204 RetIndex:0
CompName:BICYCLO[7.2.0]UNDEC-4-ENE, 4,11,11-TRIMETHYL-8-METHYLENE-, [1R-(1R*,4E,9S*)]- $$ 2,6,10,10-TETRAMETHYLBICYCLO[7

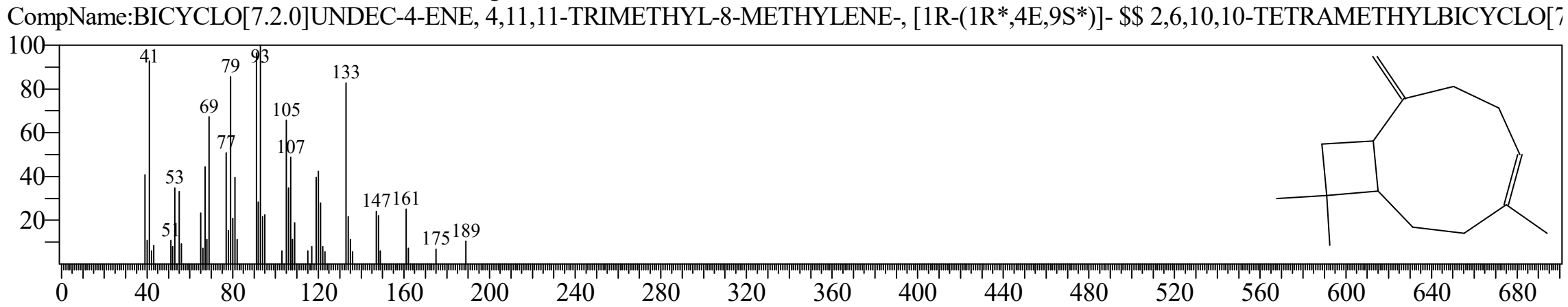

<< Target >>
Line#:69   R.Time:12.190(Scan#:1739) MassPeaks:94
RawMode:Averaged 12.185-12.195(1738-1740) BasePeak:41.05(38446)
BG Mode:Calc. from Peak Group 1 - Event 1 Scan

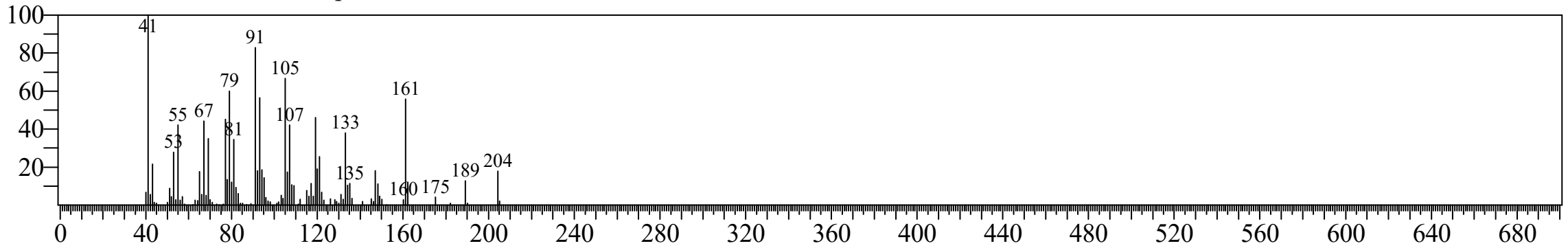

Hit#:1 Entry:110069 Library:WILEY8.LIB
SI:94 Formula:C15H24 CAS:25246-27-9 MolWeight:204 RetIndex:0
CompName:1H-CYCLOPROP[E]AZULENE, DECAHYDRO-1,1,7-TRIMETHYL-4-METHYLENE-, [1AR-(1A.ALPHA.,4A.BETA.,7.ALPHA.,7A.BETA

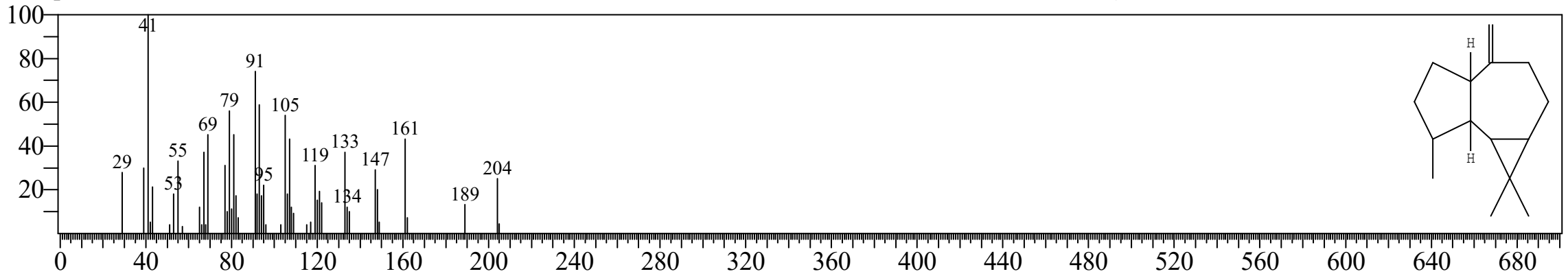

Hit#:2 Entry:19038 Library:NIST14s.lib
SI:93 Formula:C15H24 CAS:489-39-4 MolWeight:204 RetIndex:1386
CompName:Aromandendrene $$ 1H-Cycloprop[e]azulene, decahydro-1,1,7-trimethyl-4-methylene-, [1aR-(1a.alpha.,4a.alpha.,7.alpha.,7a.beta.,7b.alpha.)]- S

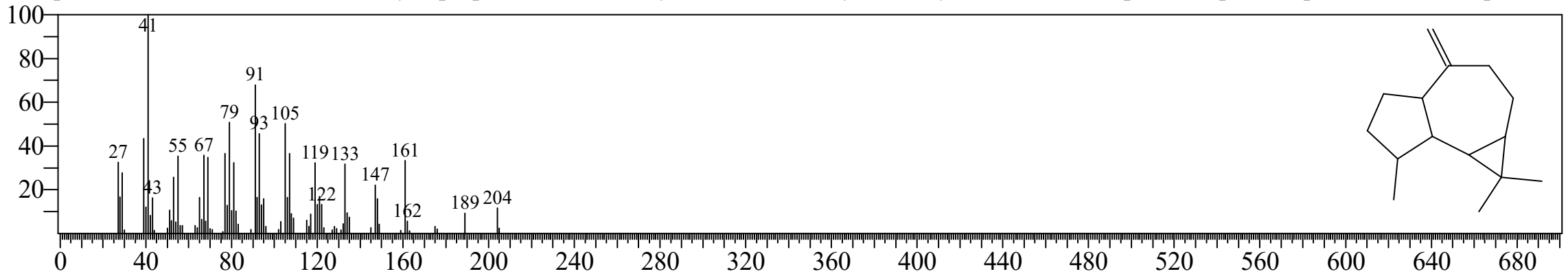

Hit#:3 Entry:19039 Library:NIST14s.lib
SI:93 Formula:C15H24 CAS:25246-27-9 MolWeight:204 RetIndex:1386
CompName:Alloaromadendrene $$ 1H-Cycloprop[e]azulene, decahydro-1,1,7-trimethyl-4-methylene-, [1aR-(1a.alpha.,4a.beta.,7.alpha.,7a.beta.,7b.alpha.)]-

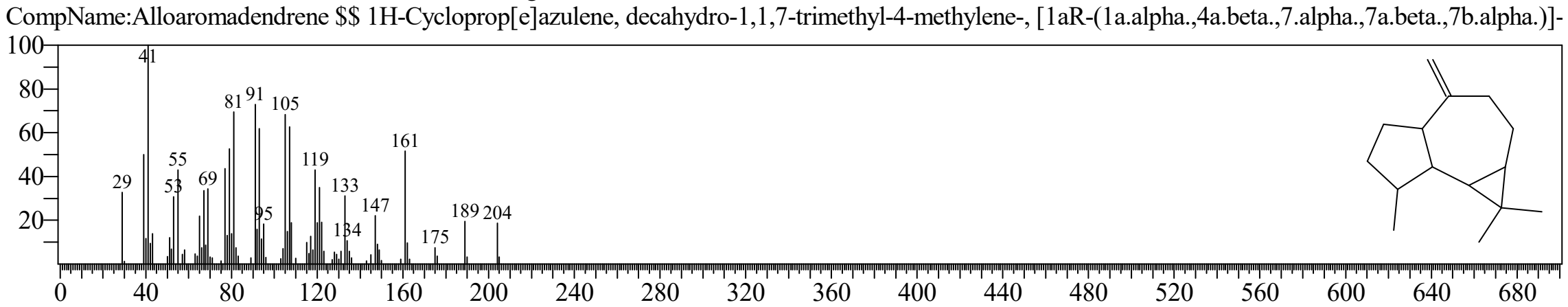

<< Target >>
Line#:70 R.Time:12.320(Scan#:1765) MassPeaks:101
RawMode:Averaged 12.315-12.325(1764-1766) BasePeak:41.05(36577)
BG Mode:Calc. from Peak Group 1 - Event 1 Scan

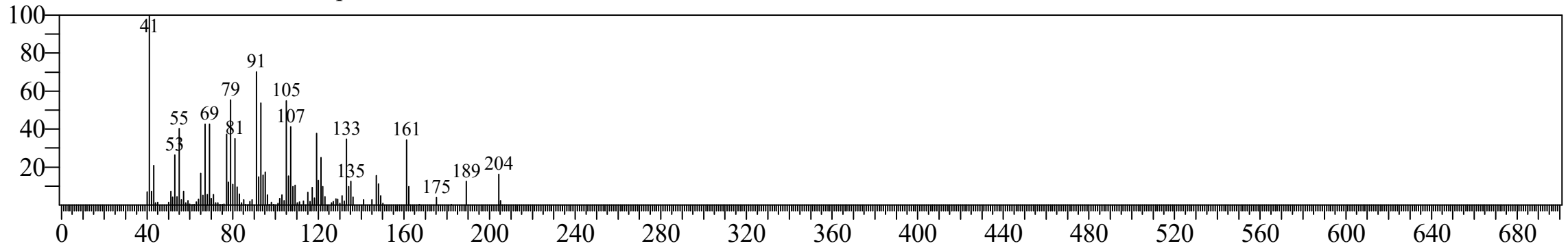

Hit#:1 Entry:110069 Library:WILEY8.LIB
SI:95 Formula:C15H24 CAS:25246-27-9 MolWeight:204 RetIndex:0
CompName:1H-CYCLOPROP[E]AZULENE, DECAHYDRO-1,1,7-TRIMETHYL-4-METHYLENE-, [1AR-(1A.ALPHA.,4A.BETA.,7.ALPHA.,7A.BET

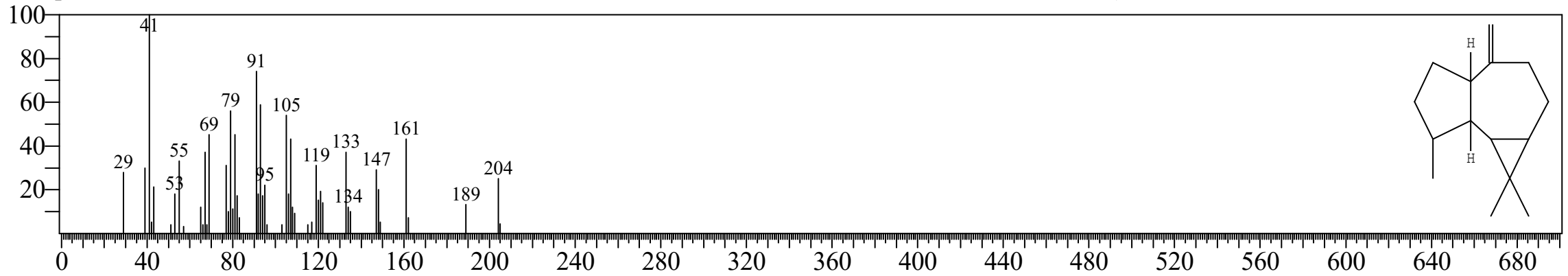

Hit#:2 Entry:19038 Library:NIST14s.lib
SI:95 Formula:C15H24 CAS:489-39-4 MolWeight:204 RetIndex:1386
CompName:Aromandendrene $$ 1H-Cycloprop[e]azulene, decahydro-1,1,7-trimethyl-4-methylene-, [1aR-(1a.alpha.,4a.alpha.,7.alpha.,7a.beta.,7b.alpha.)]- S

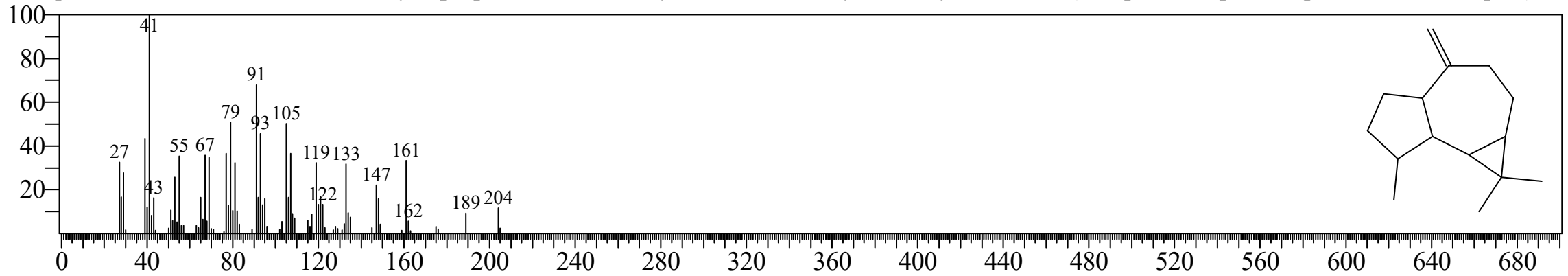

Hit#:3 Entry:110165 Library:WILEY8.LIB
SI:94 Formula:C15H24 CAS:489-39-4 MolWeight:204 RetIndex:0
CompName:1H-CYCLOPROP[E]AZULENE, DECAHYDRO-1,1,7-TRIMETHYL-4-METHYLENE-, [1AR-(1A.ALPHA.,4A.ALPHA.,7.ALPHA.,7A.BET

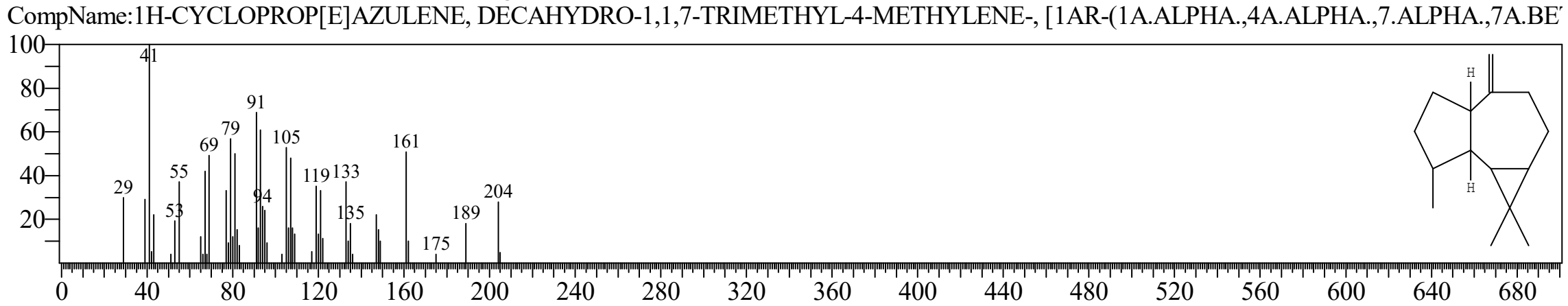

<< Target >>
Line#:71 R.Time:12.395(Scan#:1780) MassPeaks:116
RawMode:Averaged 12.390-12.400(1779-1781) BasePeak:93.10(812726)
BG Mode:Calc. from Peak Group 1 - Event 1 Scan

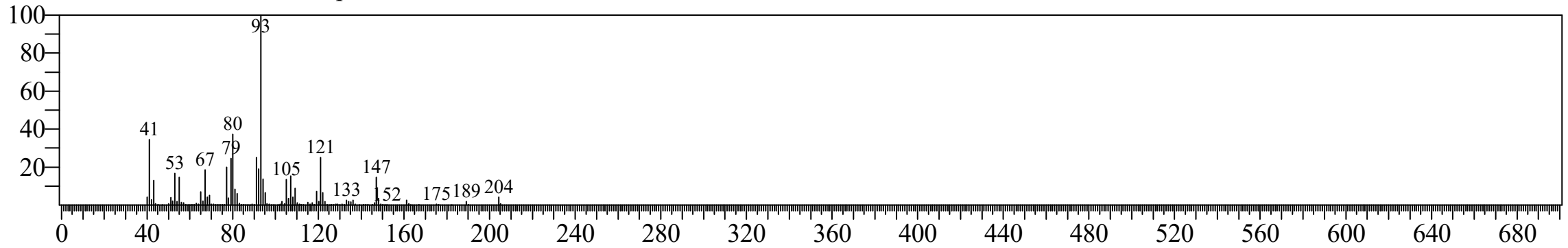

Hit#:1 Entry:19082 Library:NIST14s.lib
SI:97 Formula:C15H24 CAS:6753-98-6 MolWeight:204 RetIndex:1579
CompName:Humulene $$ .alpha.-Caryophyllene $$ 1,4,8-Cycloundecatriene, 2,6,6,9-tetramethyl-, (E,E,E)- $$ .alpha.-Humulene $$ Cycloundeca-1,4,8-trier

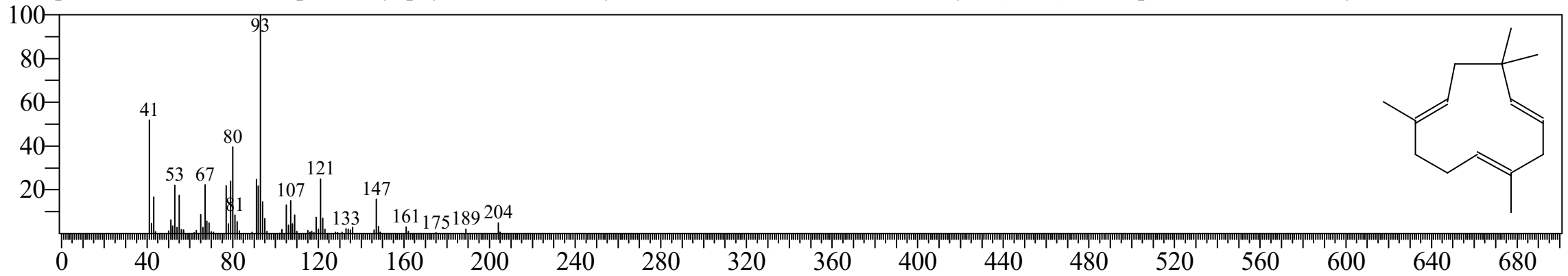

Hit#:2 Entry:109772 Library:WILEY8.LIB
SI:95 Formula:C15H24 CAS:6753-98-6 MolWeight:204 RetIndex:0
CompName:1,4,8-CYCLOUNDECATRIENE, 2,6,6,9-TETRAMETHYL-, (E,E,E)- $$ 2,6,6,9-TETRAMETHYL-1,4,8-CYCLOUNDECATRIENE # $$ .A

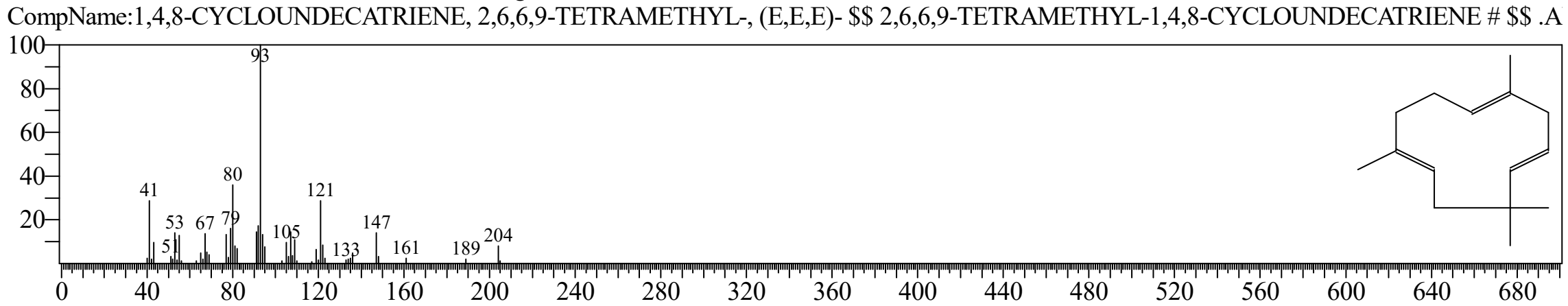

Hit#:3 Entry:109780 Library:WILEY8.LIB
SI:95 Formula:C15H24 CAS:6753-98-6 MolWeight:204 RetIndex:0
CompName:1,4,8-CYCLOUNDECATRIENE, 2,6,6,9-TETRAMETHYL-, (E,E,E)- $$ 2,6,6,9-TETRAMETHYL-1,4,8-CYCLOUNDECATRIENE # $$ .A

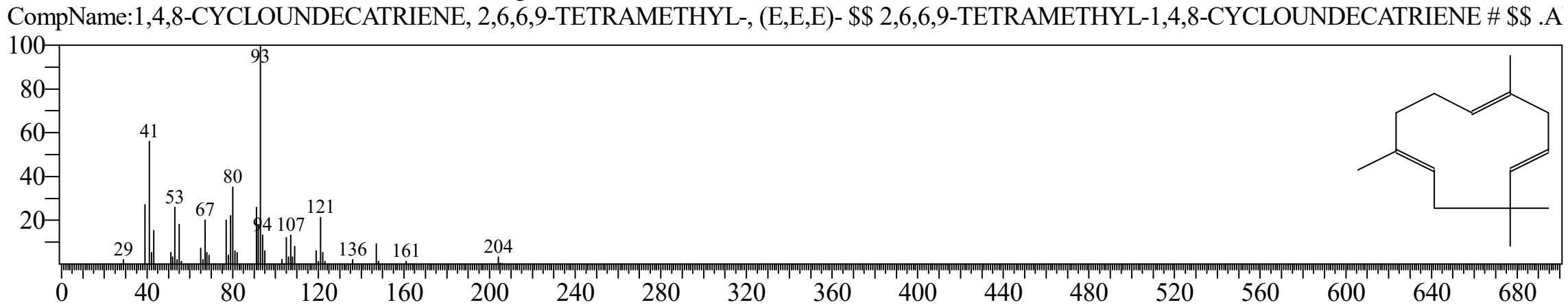

<< Target >>
Line#:72 R.Time:12.500(Scan#:1801) MassPeaks:117
RawMode:Averaged 12.495-12.505(1800-1802) BasePeak:93.05(648905)
BG Mode:Calc. from Peak Group 1 - Event 1 Scan

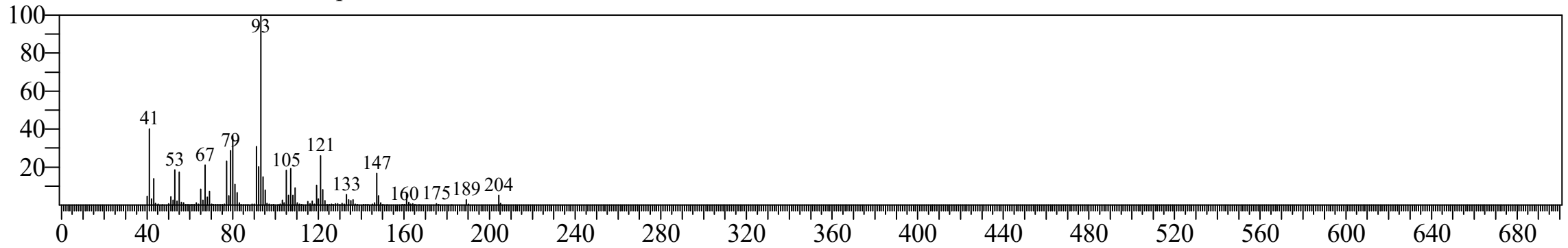

Hit#:1 Entry:19082 Library:NIST14s.lib
SI:96 Formula:C15H24 CAS:6753-98-6 MolWeight:204 RetIndex:1579
CompName:Humulene $$ .alpha.-Caryophyllene $$ 1,4,8-Cycloundecatriene, 2,6,6,9-tetramethyl-, (E,E,E)- $$ .alpha.-Humulene $$ Cycloundeca-1,4,8-trier

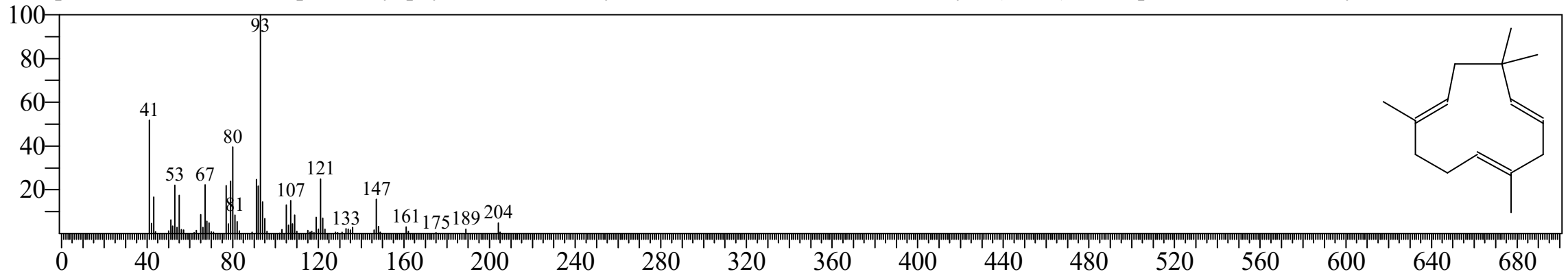

Hit#:2 Entry:109780 Library:WILEY8.LIB
SI:93 Formula:C15H24 CAS:6753-98-6 MolWeight:204 RetIndex:0
CompName:1,4,8-CYCLOUNDECATRIENE, 2,6,6,9-TETRAMETHYL-, (E,E,E)- $$ 2,6,6,9-TETRAMETHYL-1,4,8-CYCLOUNDECATRIENE # $$ .A

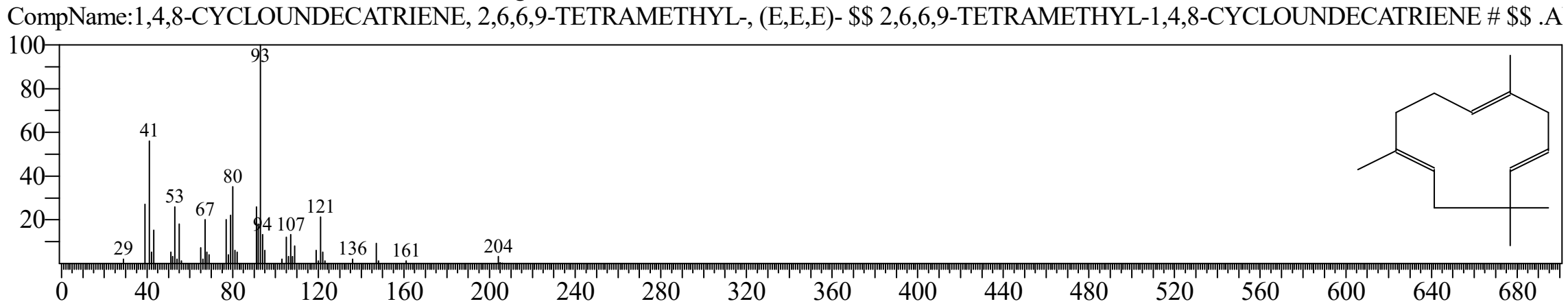

Hit#:3 Entry:109772 Library:WILEY8.LIB
SI:93 Formula:C15H24 CAS:6753-98-6 MolWeight:204 RetIndex:0
CompName:1,4,8-CYCLOUNDECATRIENE, 2,6,6,9-TETRAMETHYL-, (E,E,E)- $$ 2,6,6,9-TETRAMETHYL-1,4,8-CYCLOUNDECATRIENE # $$ .A

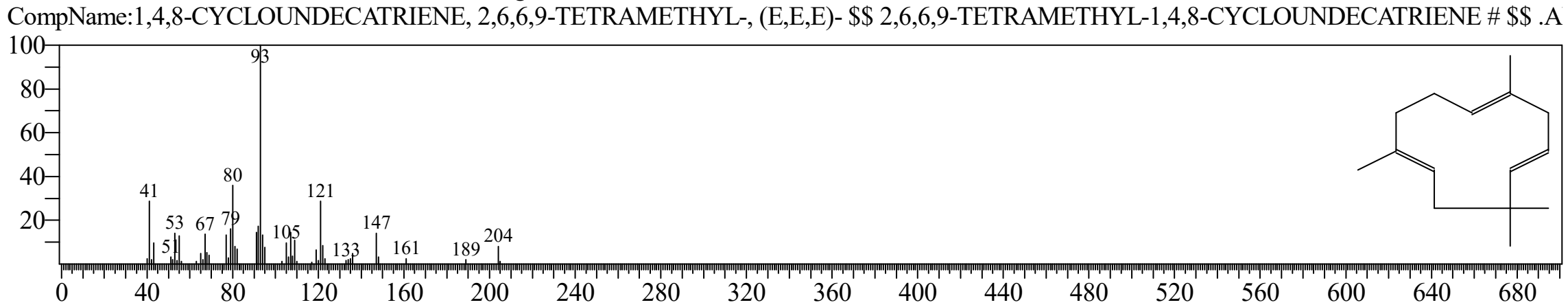

<< Target >>
Line#:73 R.Time:12.610(Scan#:1823) MassPeaks:59
RawMode:Averaged 12.605-12.615(1822-1824) BasePeak:41.05(15940)
BG Mode:Calc. from Peak Group 1 - Event 1 Scan

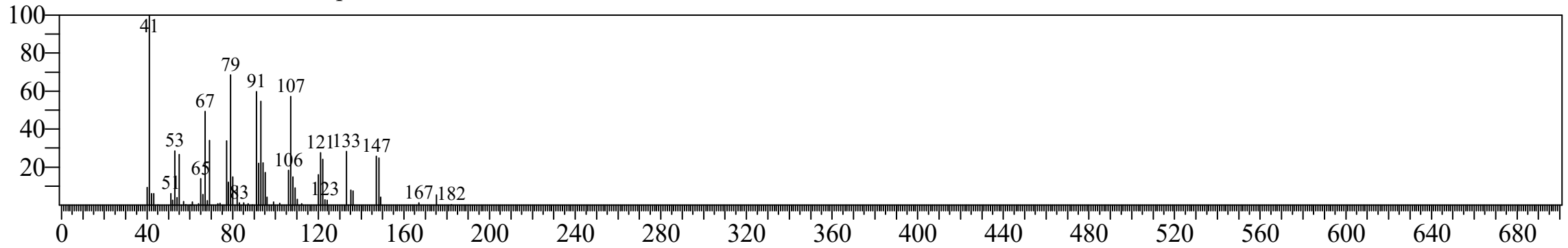

Hit#:1 Entry:109829 Library:WILEY8.LIB
SI:84 Formula:C15H24 CAS:87-44-5 MolWeight:204 RetIndex:0
CompName:BICYCLO[7.2.0]UNDEC-4-ENE, 4,11,11-TRIMETHYL-8-METHYLENE-, [1R-(1R*,4E,9S*)]- $$ 2,6,10,10-TETRAMETHYLBICYCLO[7

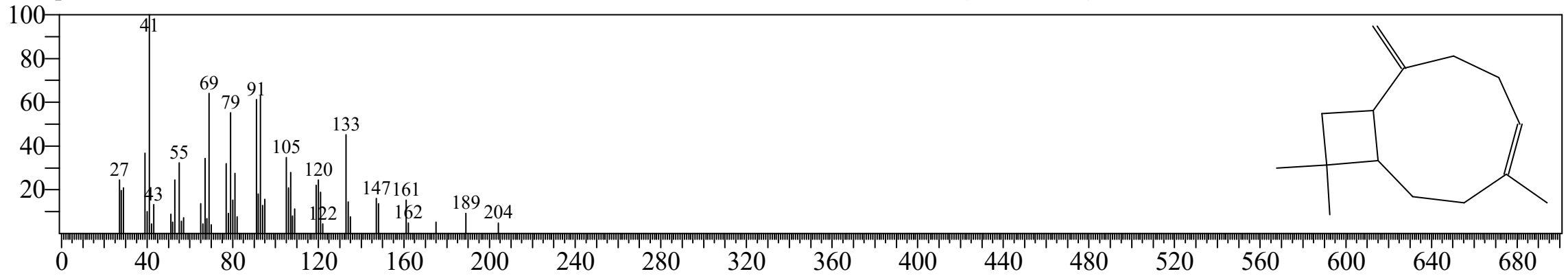

Hit#:2 Entry:109830 Library:WILEY8.LIB
SI:84 Formula:C15H24 CAS:87-44-5 MolWeight:204 RetIndex:0
CompName:BICYCLO[7.2.0]UNDEC-4-ENE, 4,11,11-TRIMETHYL-8-METHYLENE-, [1R-(1R*,4E,9S*)]- $$ 2,6,10,10-TETRAMETHYLBICYCLO[7

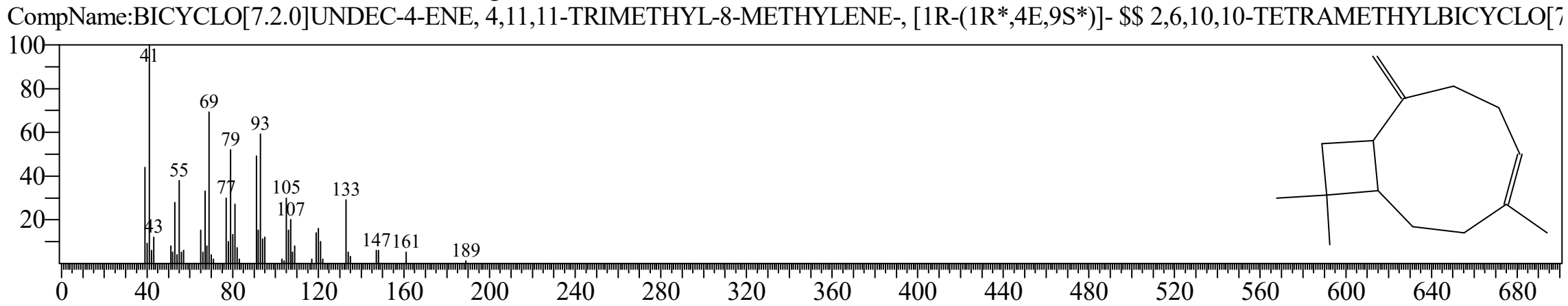

Hit#:3 Entry:90653 Library:WILEY8.LIB
SI:84 Formula:C14H22 CAS:62338-54-9 MolWeight:190 RetIndex:0
CompName:1,5-CYCLOUNDECADIENE, 8,8-DIMETHYL-9-METHYLENE- $$ 8,8-DIMETHYL-9-METHYLENE-1,5-CYCLOUNDECADIENE # $$

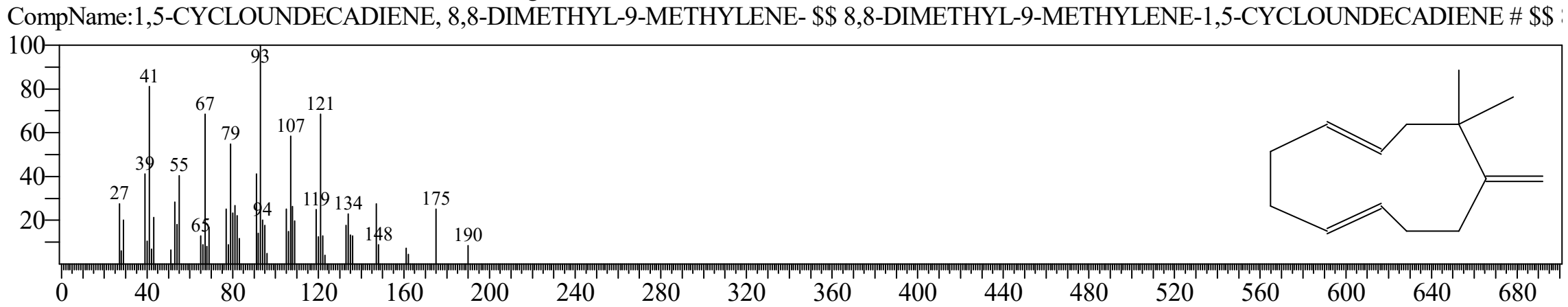

<< Target >>
Line#:74 R.Time:12.660(Scan#:1833) MassPeaks:105
RawMode:Averaged 12.655-12.665(1832-1834) BasePeak:91.05(70590)
BG Mode:Calc. from Peak Group 1 - Event 1 Scan

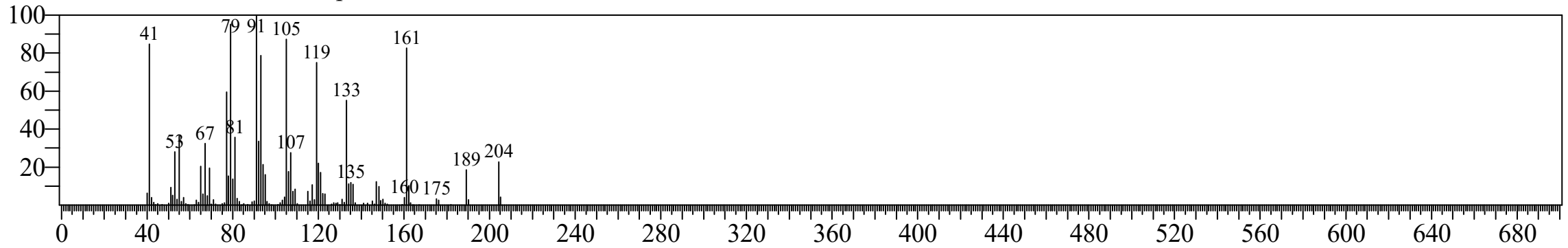

Hit#:1 Entry:19053 Library:NIST14s.lib
SI:91 Formula:C15H24 CAS:4630-07-3 MolWeight:204 RetIndex:1474
CompName:Naphthalene, 1,2,3,5,6,7,8,8a-octahydro-1,8a-dimethyl-7-(1-methylethenyl)-, [1R-(1.alpha.,7.beta.,8a.alpha.)]- $$ 4.beta.H,5.alpha.-Eremophila-

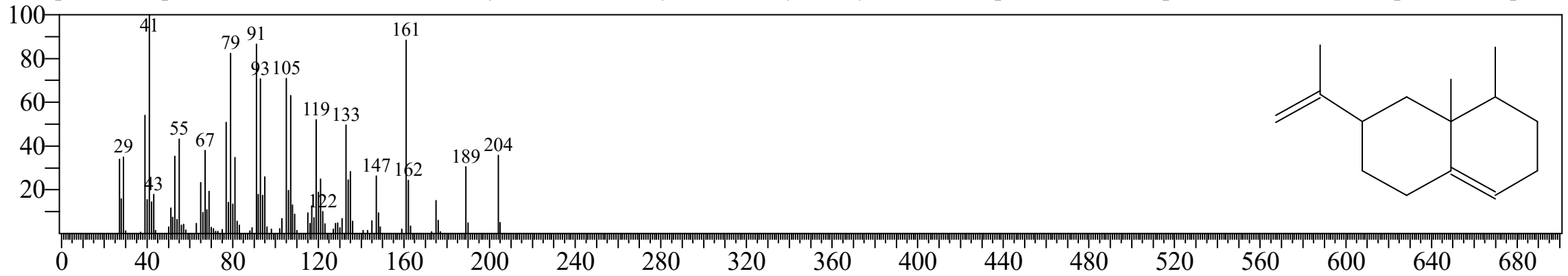

Hit#:2 Entry:109977 Library:WILEY8.LIB
SI:91 Formula:C15H24 CAS:4630-07-3 MolWeight:204 RetIndex:0
CompName:NAPHTHALENE, 1,2,3,5,6,7,8,8A-OCTAHYDRO-1,8A-DIMETHYL-7-(1-METHYLETHENYL)-, [1R-(1.ALPHA.,7.BETA.,8A.ALPHA.)]-

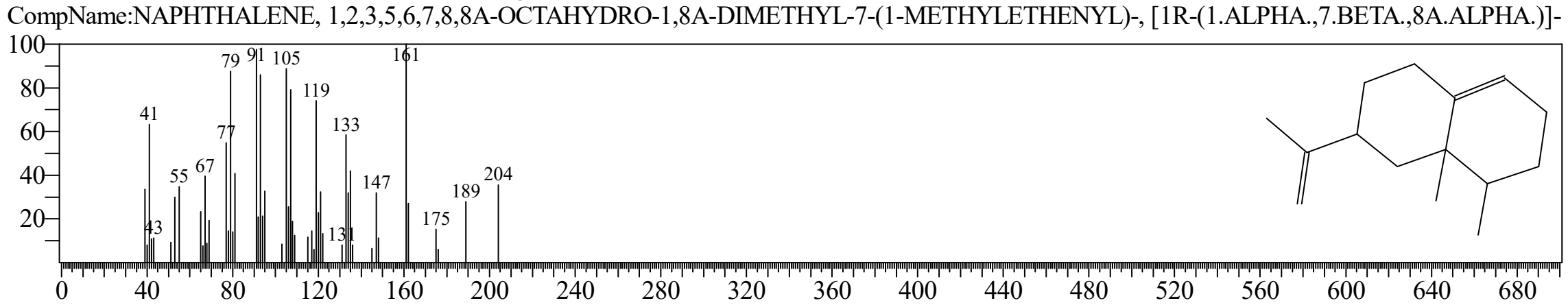

Hit#:3 Entry:50017 Library:NIST14.lib
SI:91 Formula:C15H24 CAS:317819-80-0 MolWeight:204 RetIndex:0
CompName:(S,1Z,6Z)-8-Isopropyl-1-methyl-5-methylenecyclodeca-1,6-diene $$ 1,6-Cyclodecadiene, 1-methyl-5-methylene-8-(1-methylethyl)-, (1Z,6Z,8S)

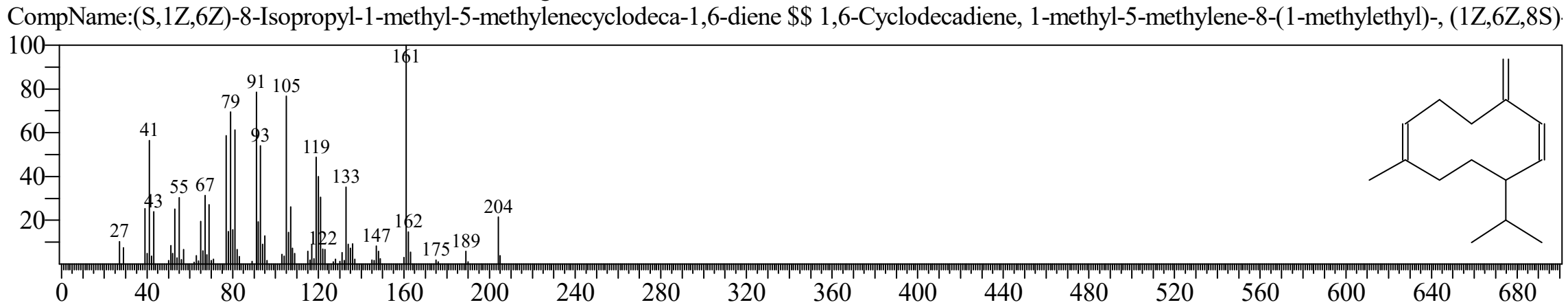

<< Target >>
Line#:75 R.Time:12.750(Scan#:1851) MassPeaks:106
RawMode:Averaged 12.745-12.755(1850-1852) BasePeak:161.20(68573)
BG Mode:Calc. from Peak Group 1 - Event 1 Scan

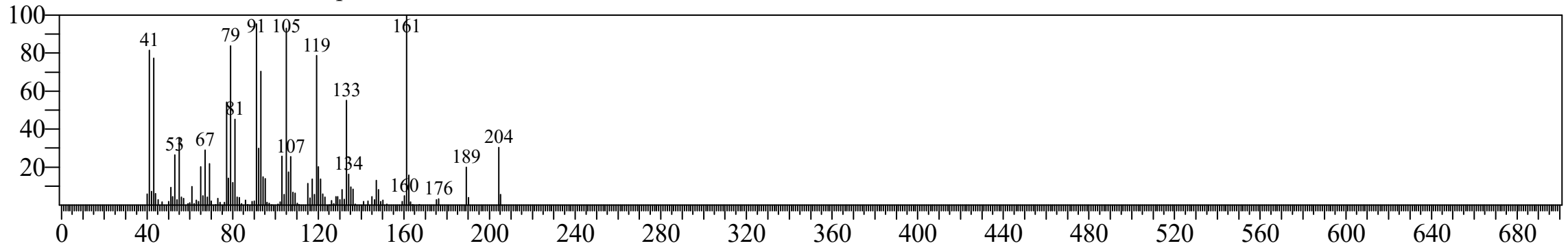

Hit#:1 Entry:109977 Library:WILEY8.LIB
SI:91 Formula:C15H24 CAS:4630-07-3 MolWeight:204 RetIndex:0
CompName:NAPHTHALENE, 1,2,3,5,6,7,8,8A-OCTAHYDRO-1,8A-DIMETHYL-7-(1-METHYLETHENYL)-, [1R-(1.ALPHA.,7.BETA.,8A.ALPHA.)]-

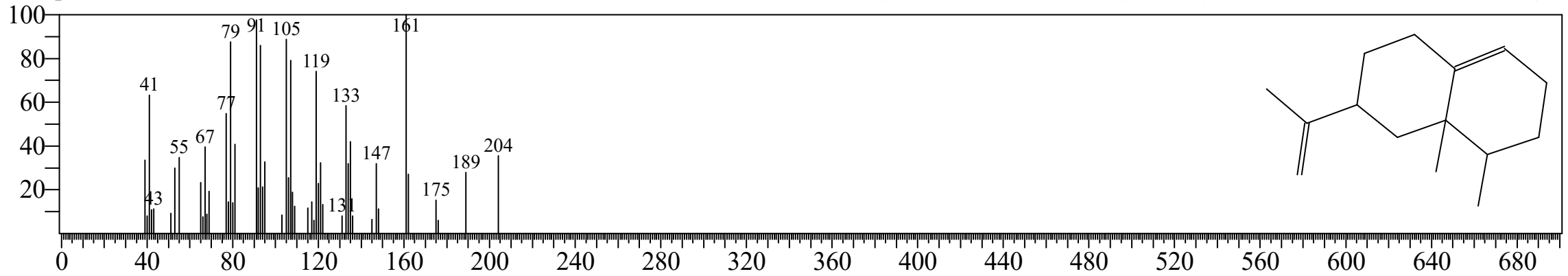

Hit#:2 Entry:19053 Library:NIST14s.lib
SI:90 Formula:C15H24 CAS:4630-07-3 MolWeight:204 RetIndex:1474
CompName:Naphthalene, 1,2,3,5,6,7,8,8a-octahydro-1,8a-dimethyl-7-(1-methylethenyl)-, [1R-(1.alpha.,7.beta.,8a.alpha.)]- $$ 4.beta.H,5.alpha.-Eremophila-

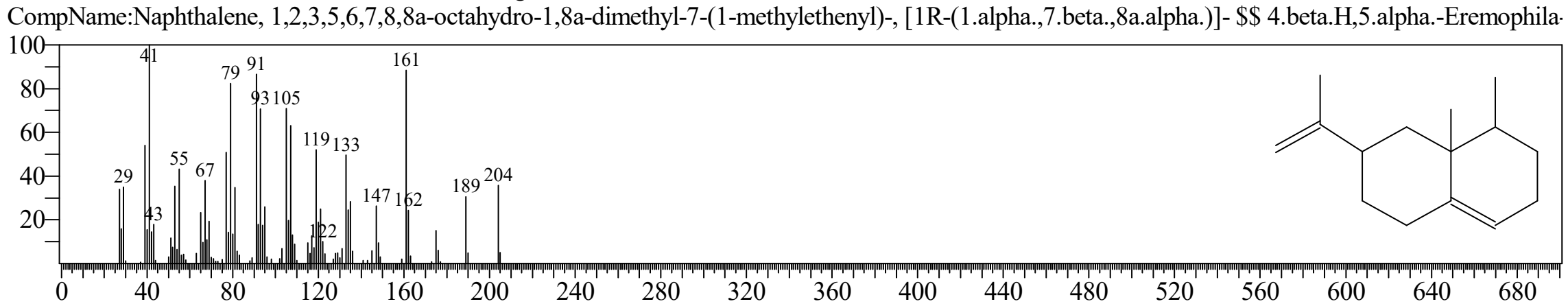

Hit#:3 Entry:109996 Library:WILEY8.LIB
SI:90 Formula:C15H24 CAS:30021-74-0 MolWeight:204 RetIndex:0
CompName:NAPHTHALENE, 1,2,3,4,4A,5,6,8A-OCTAHYDRO-7-METHYL-4-METHYLENE-1-(1-METHYLETHYL)-, (1.ALPHA.,4A.ALPHA.,8A.Al

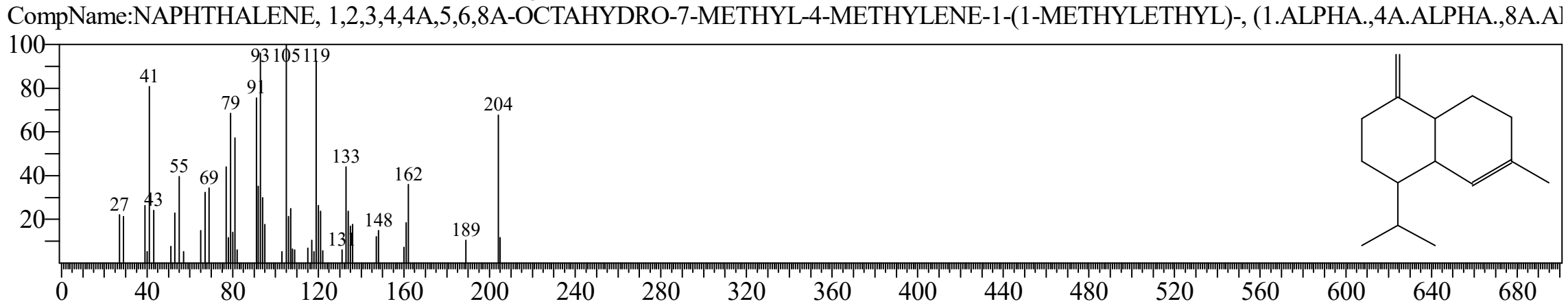

<< Target >>
Line#:76 R.Time:12.830(Scan#:1867) MassPeaks:111
RawMode:Averaged 12.825-12.835(1866-1868) BasePeak:41.05(74685)
BG Mode:Calc. from Peak Group 1 - Event 1 Scan

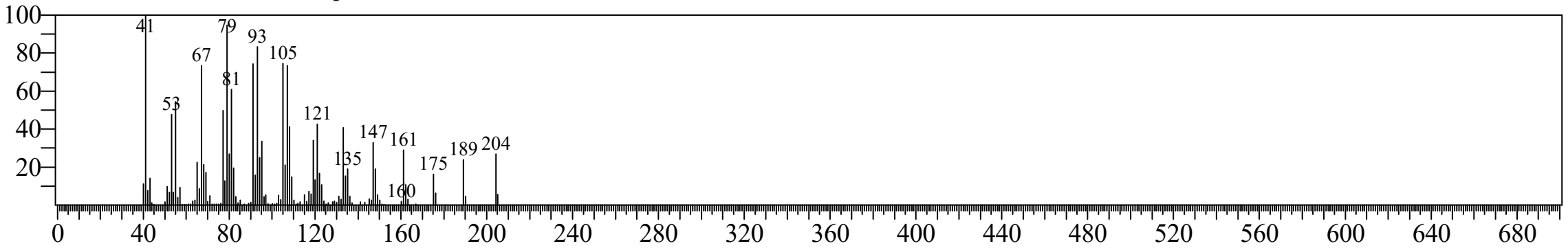

Hit#:1 Entry:19037 Library:NIST14s.lib
SI:92 Formula:C15H24 CAS:473-13-2 MolWeight:204 RetIndex:1474
CompName:Naphthalene, 1,2,3,4,4a,5,6,8a-octahydro-4a,8-dimethyl-2-(1-methylethenyl)-, [2R-(2.alpha.,4a.alpha.,8a.beta.)]- $$ .alpha.-Selinene $$ 2-Isopro

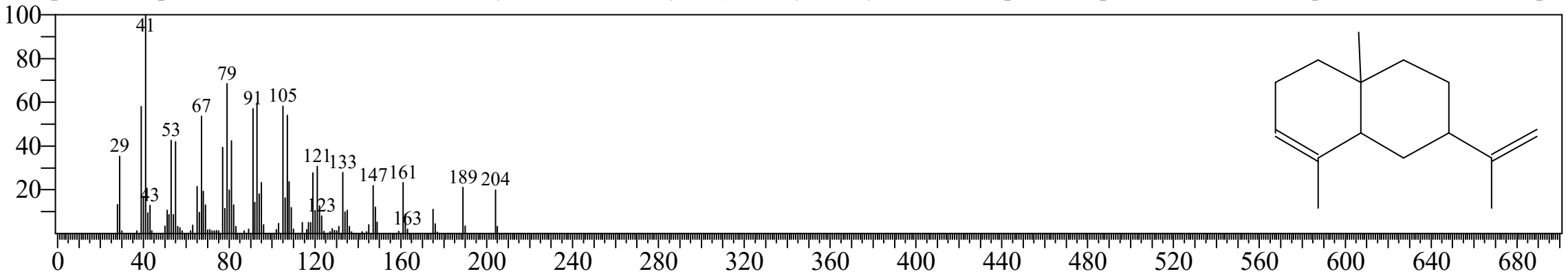

Hit#:2 Entry:110390 Library:WILEY8.LIB
SI:92 Formula:C15H24 CAS:0-00-0 MolWeight:204 RetIndex:0
CompName:7-ISOPROPENYL-4A-METHYL-1-METHYLENEDECAHYDRONAPHTHALENE $$ EUDESMA-4(14),11-DIEN

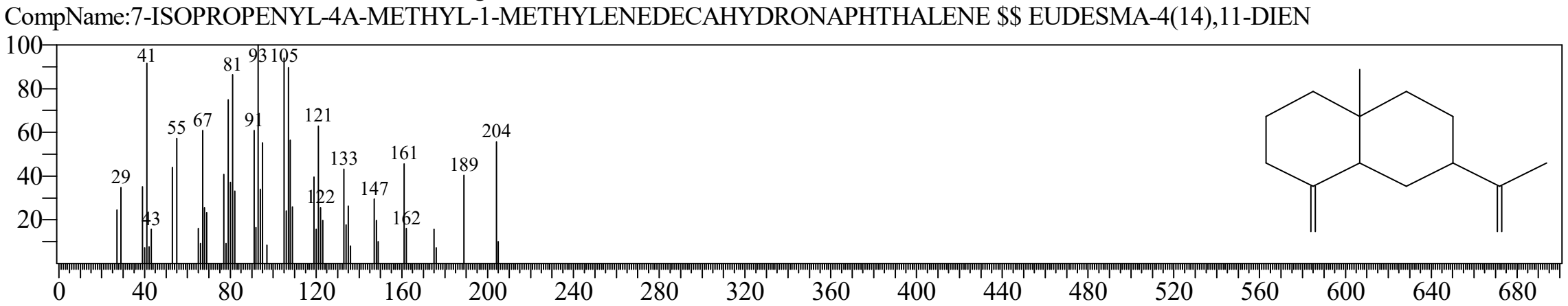

Hit#:3 Entry:19113 Library:NIST14s.lib
SI:92 Formula:C15H24 CAS:17066-67-0 MolWeight:204 RetIndex:1469
CompName:Naphthalene, decahydro-4a-methyl-1-methylene-7-(1-methylethenyl)-, [4aR-(4a.alpha.,7.alpha.,8a.beta.)]- $$ Eudesma-4(14),11-diene $$ .beta.-

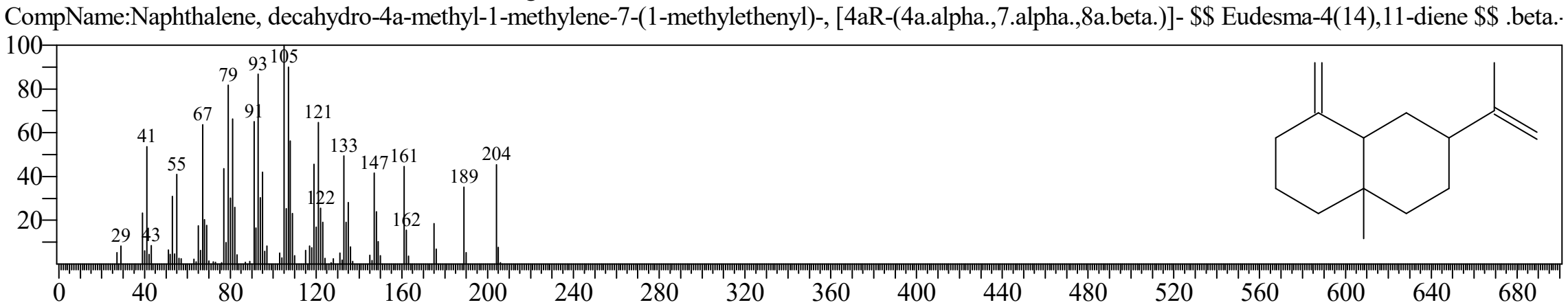

<< Target >>
Line#:77   R.Time:12.940(Scan#:1889) MassPeaks:132
RawMode:Averaged 12.935-12.945(1888-1890) BasePeak:81.05(223525)
BG Mode:Calc. from Peak Group 1 - Event 1 Scan

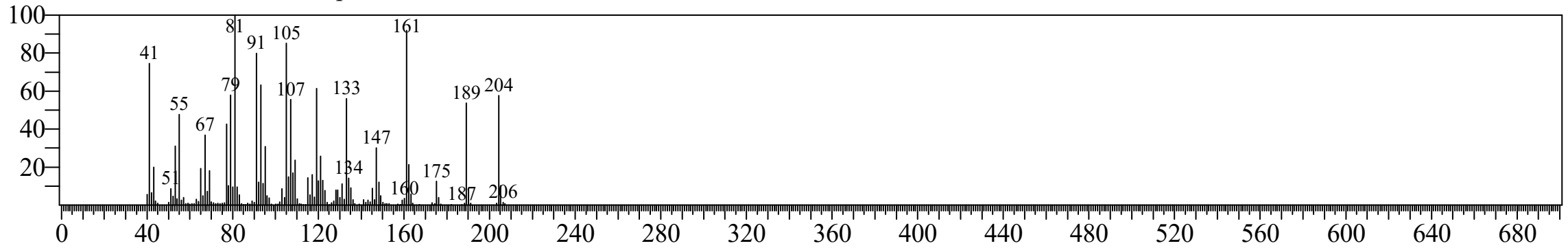

Hit#:1 Entry:49881 Library:NIST14.lib
SI:92 Formula:C15H24 CAS:0-00-0 MolWeight:204 RetIndex:1490
CompName:Guaia-1(10),11-diene

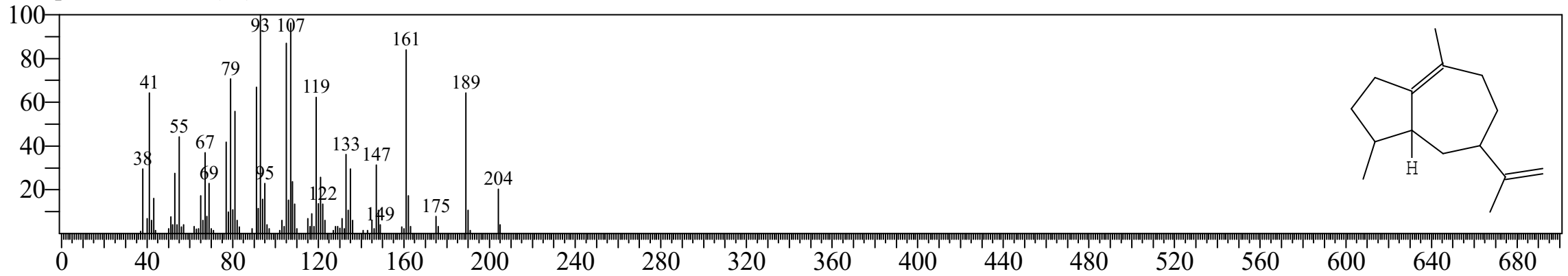

Hit#:2 Entry:50089 Library:NIST14.lib
SI:92 Formula:C15H24 CAS:489-40-7 MolWeight:204 RetIndex:1419
CompName:1H-Cycloprop[e]azulene, 1a,2,3,4,4a,5,6,7b-octahydro-1,1,4,7-tetramethyl-, [1aR-(1a.alpha.,4.alpha.,4a.beta.,7b.alpha.)]- $$ 1H-Cycloprop[e]a:

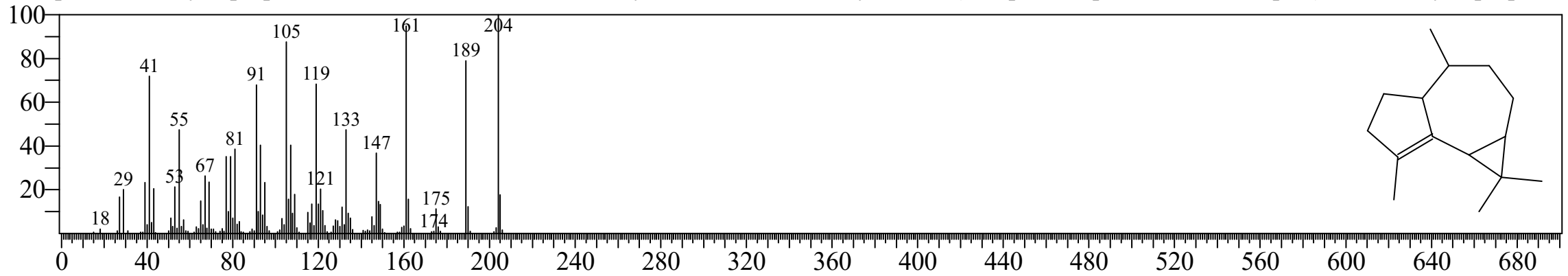

Hit#:3 Entry:110052 Library:WILEY8.LIB
SI:92 Formula:C15H24 CAS:489-40-7 MolWeight:204 RetIndex:0
CompName:1H-CYCLOPROP[E]AZULENE, 1A,2,3,4,4A,5,6,7B-OCTAHYDRO-1,1,4,7-TETRAMETHYL-, [1AR-(1A.ALPHA.,4.ALPHA.,4A.BETA.,7

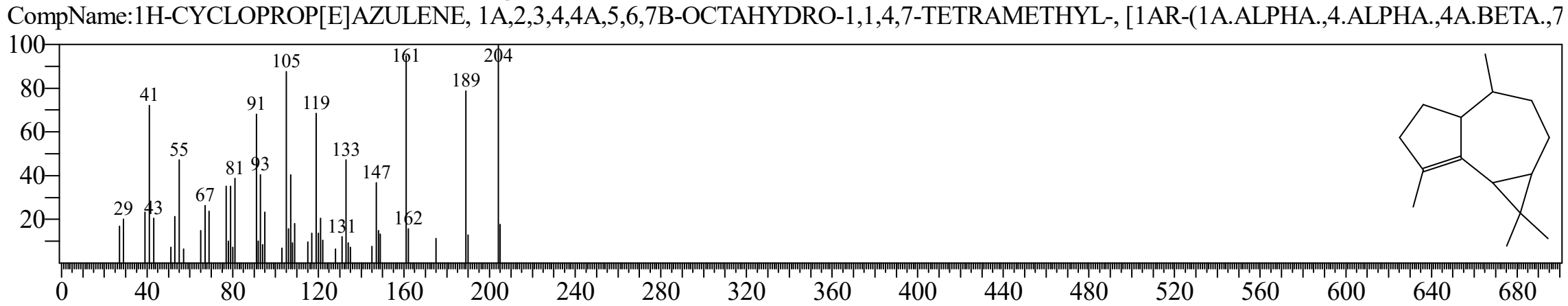

<< Target >>
Line#:78   R.Time:13.025(Scan#:1906) MassPeaks:128
RawMode:Averaged 13.020-13.030(1905-1907) BasePeak:41.05(171148)
BG Mode:Calc. from Peak Group 1 - Event 1 Scan

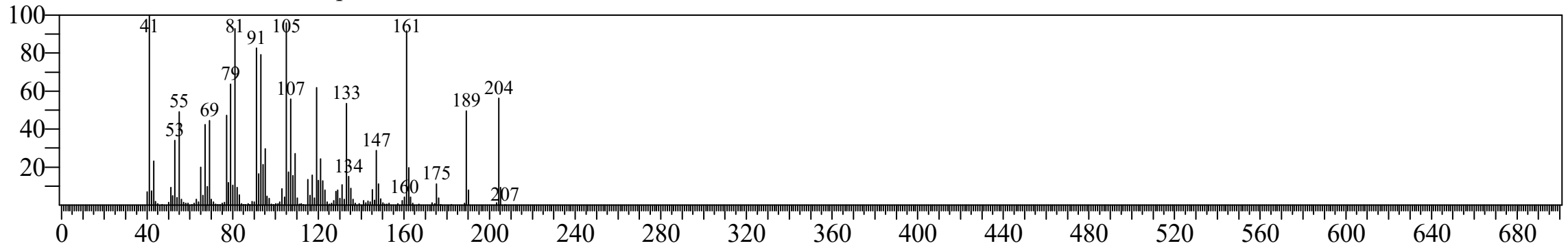

Hit#:1 Entry:110071 Library:WILEY8.LIB
SI:92 Formula:C15H24 CAS:25246-27-9 MolWeight:204 RetIndex:0
CompName:1H-CYCLOPROP[E]AZULENE, DECAHYDRO-1,1,7-TRIMETHYL-4-METHYLENE-, [1AR-(1A.ALPHA.,4A.BETA.,7.ALPHA.,7A.BET

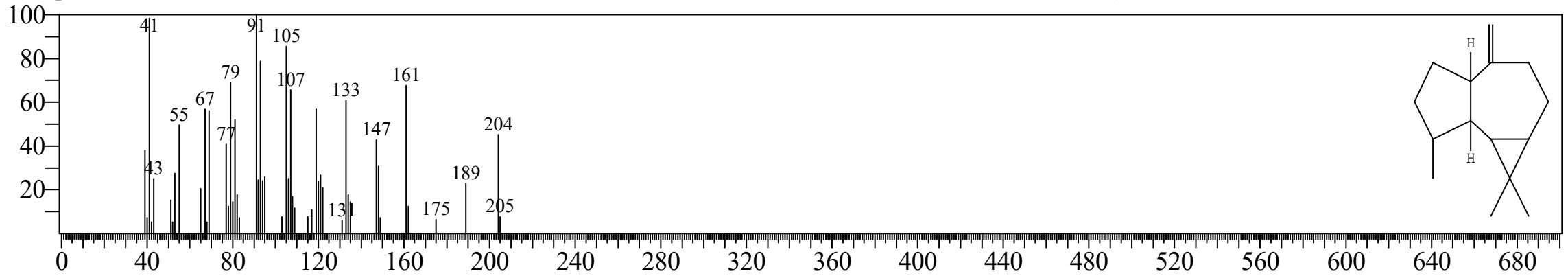

Hit#:2 Entry:49881 Library:NIST14.lib
SI:92 Formula:C15H24 CAS:0-00-0 MolWeight:204 RetIndex:1490
CompName:Guaia-1(10),11-diene

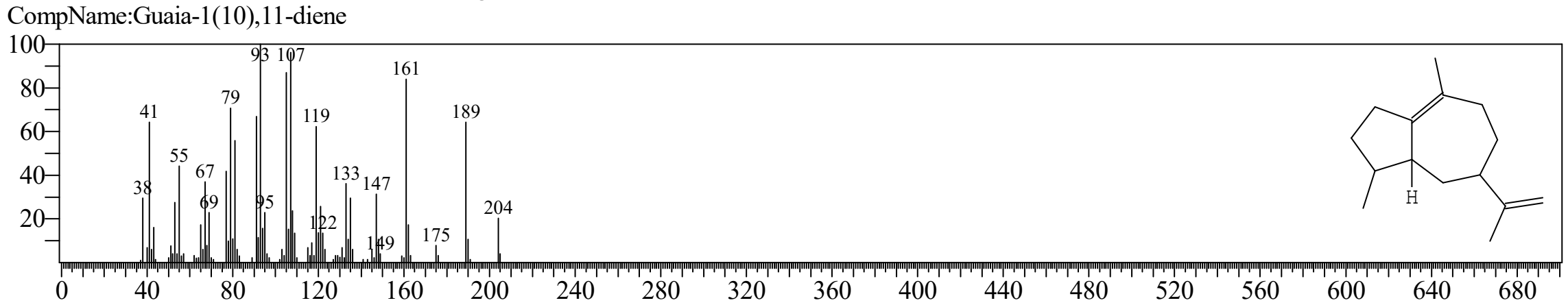

Hit#:3 Entry:110063 Library:WILEY8.LIB
SI:92 Formula:C15H24 CAS:489-39-4 MolWeight:204 RetIndex:0
CompName:1H-CYCLOPROP[E]AZULENE, DECAHYDRO-1,1,7-TRIMETHYL-4-METHYLENE-, [1AR-(1A.ALPHA.,4A.ALPHA.,7.ALPHA.,7A.BE

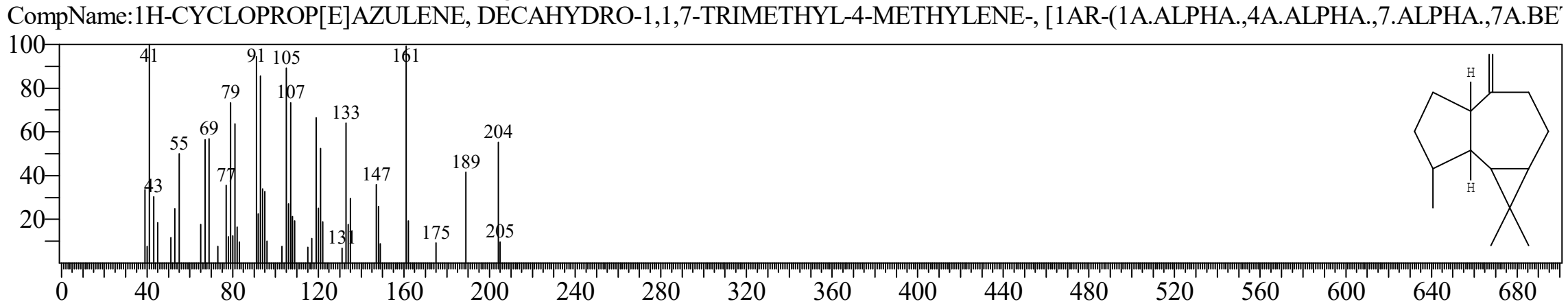

<< Target >>
Line#:79   R.Time:13.165(Scan#:1934) MassPeaks:130
RawMode:Averaged 13.160-13.170(1933-1935) BasePeak:161.15(599854)
BG Mode:Calc. from Peak Group 1 - Event 1 Scan

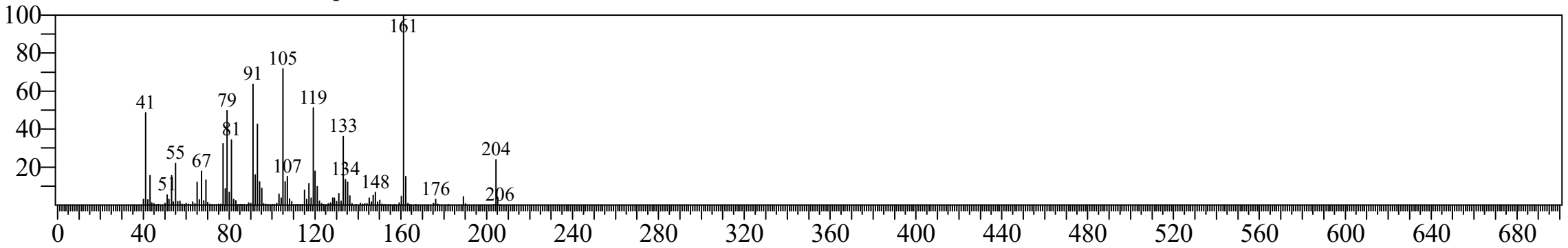

Hit#:1 Entry:50032 Library:NIST14.lib
SI:94 Formula:C15H24 CAS:6980-46-7 MolWeight:204 RetIndex:0
CompName:(1S,4aR,8aS)-1-Isopropyl-7-methyl-4-methylene-1,2,3,4,4a,5,6,8a-octahydronaphthalene $$ .gamma.-Amorphene $$

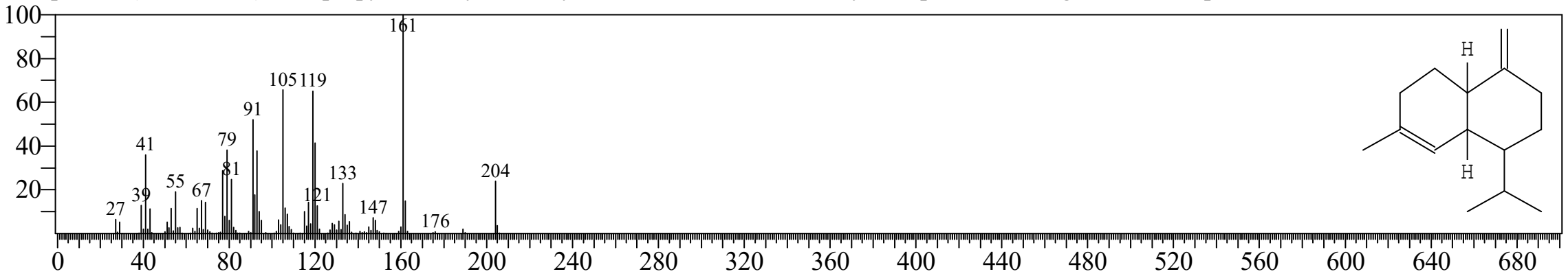

Hit#:2 Entry:19197 Library:NIST14s.lib
SI:94 Formula:C15H24 CAS:30021-74-0 MolWeight:204 RetIndex:1435
CompName:.gamma.-Muurolene $$ Naphthalene, 1,2,3,4,4a,5,6,8a-octahydro-7-methyl-4-methylene-1-(1-methylethyl)-, (1.alpha.,4a.alpha.,8a.alpha.)- $$ 1-

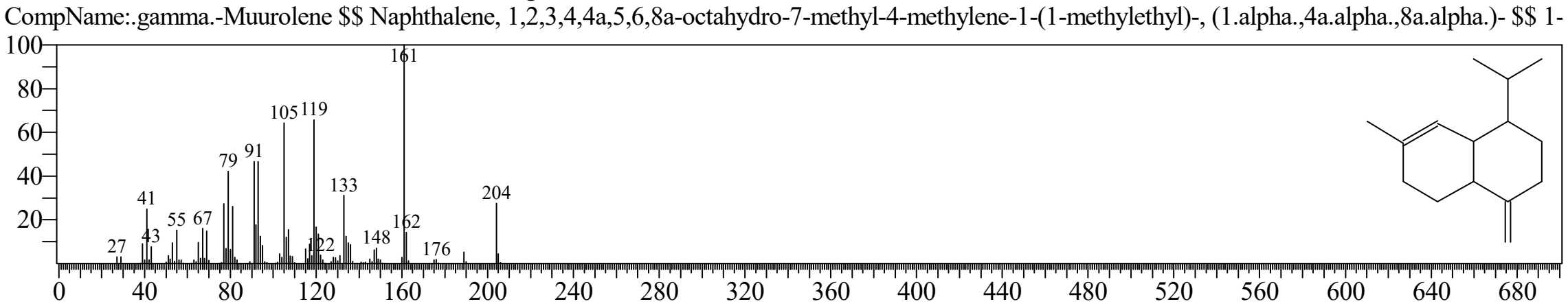

Hit#:3 Entry:19174 Library:NIST14s.lib
SI:94 Formula:C15H24 CAS:30021-74-0 MolWeight:204 RetIndex:1435
CompName:.gamma.-Muurolene $$ Naphthalene, 1,2,3,4,4a,5,6,8a-octahydro-7-methyl-4-methylene-1-(1-methylethyl)-, (1.alpha.,4a.alpha.,8a.alpha.)- $$ 1-

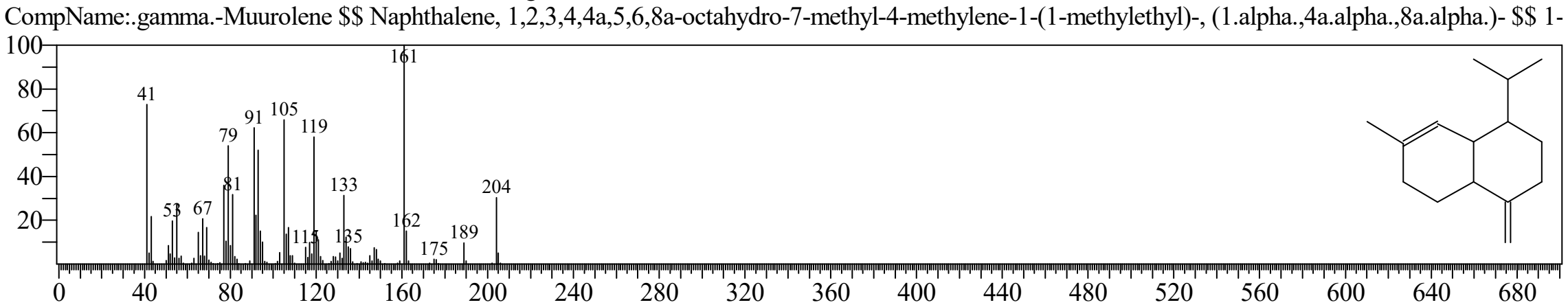

<< Target >>
Line#:80 R.Time:13.260(Scan#:1953) MassPeaks:143
RawMode:Averaged 13.255-13.265(1952-1954) BasePeak:161.15(1233674)
BG Mode:Calc. from Peak Group 1 - Event 1 Scan

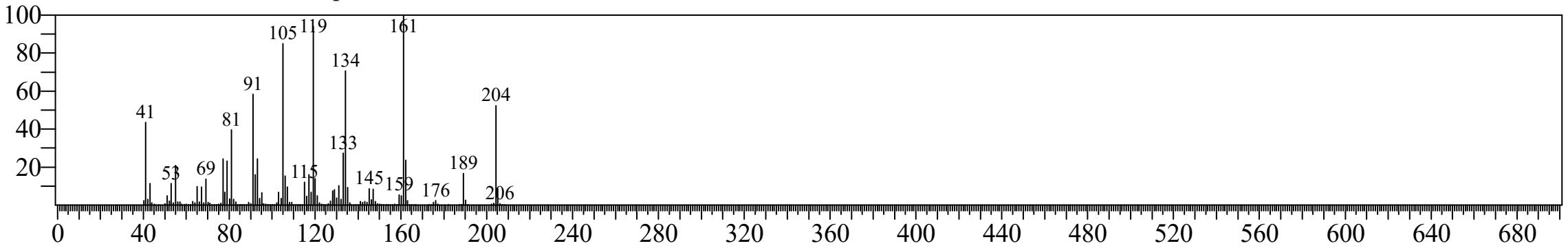

Hit#:1 Entry:109934 Library:WILEY8.LIB
SI:94 Formula:C15H24 CAS:483-76-1 MolWeight:204 RetIndex:0
CompName:NAPHTHALENE, 1,2,3,5,6,8A-HEXAHYDRO-4,7-DIMETHYL-1-(1-METHYLETHYL)-, (1S-CIS)- $$ 1-ISOPROPYL-4,7-DIMETHYL-1,

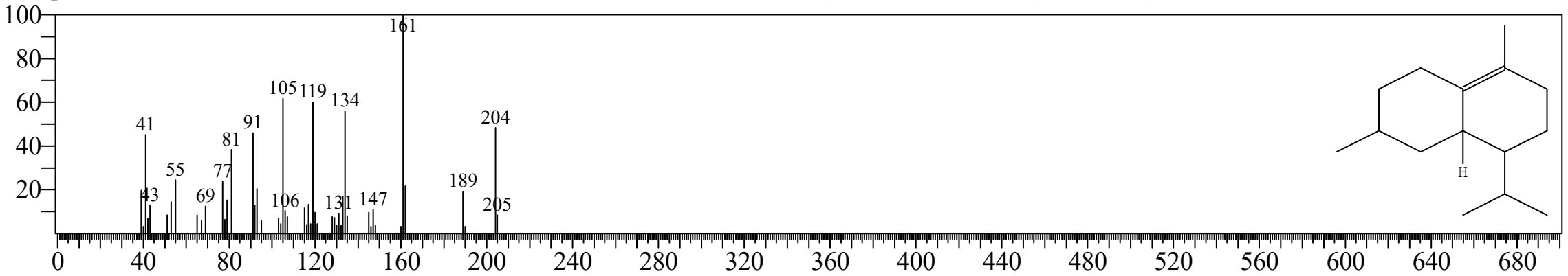

Hit#:2 Entry:109936 Library:WILEY8.LIB
SI:94 Formula:C15H24 CAS:483-76-1 MolWeight:204 RetIndex:0
CompName:NAPHTHALENE, 1,2,3,5,6,8A-HEXAHYDRO-4,7-DIMETHYL-1-(1-METHYLETHYL)-, (1S-CIS)- $$ 1-ISOPROPYL-4,7-DIMETHYL-1,

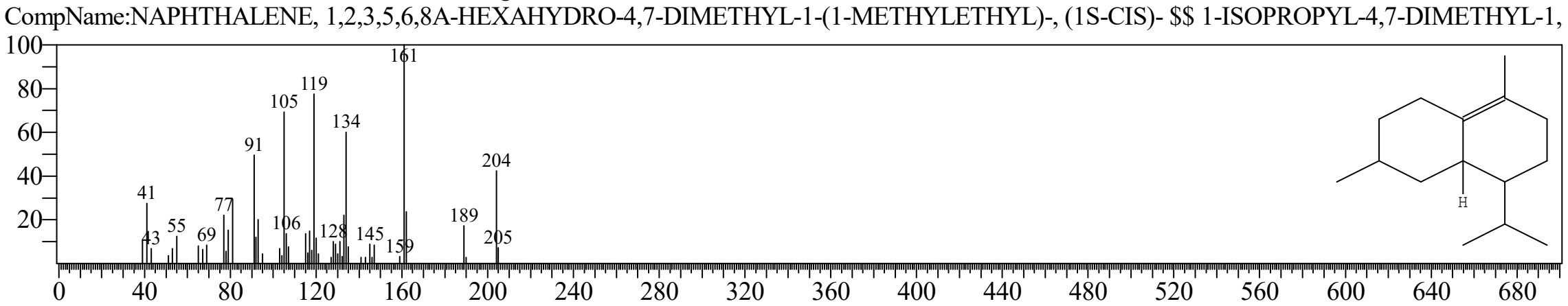

Hit#:3 Entry:19198 Library:NIST14s.lib
SI:93 Formula:C15H24 CAS:483-76-1 MolWeight:204 RetIndex:1469
CompName:Naphthalene, 1,2,3,5,6,8a-hexahydro-4,7-dimethyl-1-(1-methylethyl)-, (1S-cis)- $$ Cadina-1(10),4-diene $$ .delta.-Cadinene, (+)- $$ (+)-.delta.

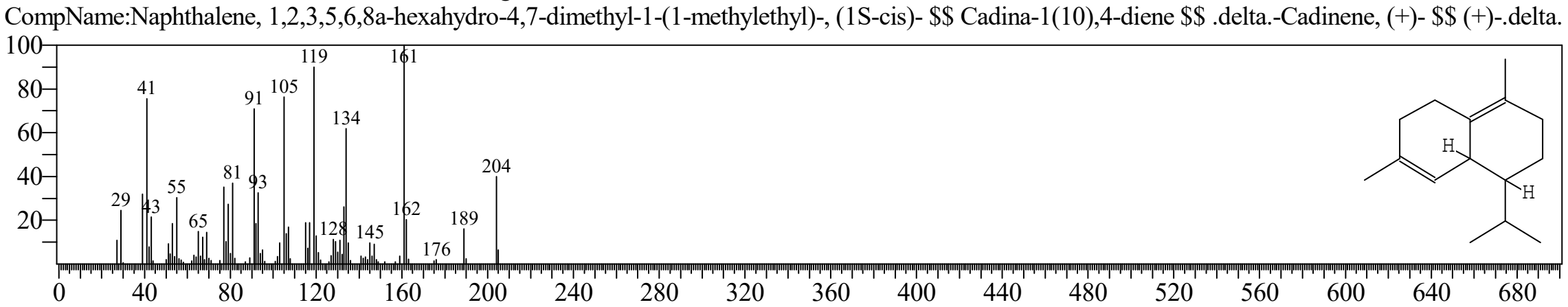

<< Target >>
Line#:81 R.Time:13.325(Scan#:1966) MassPeaks:141
RawMode:Averaged 13.320-13.330(1965-1967) BasePeak:161.15(963202)
BG Mode:Calc. from Peak Group 1 - Event 1 Scan

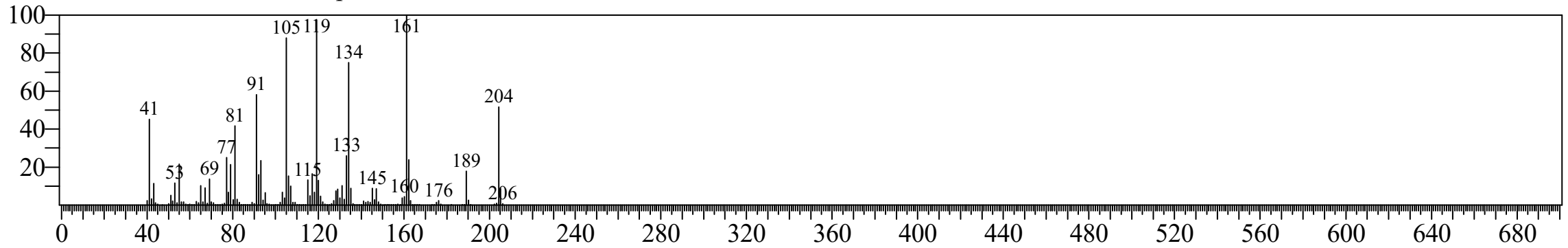

Hit#:1 Entry:109934 Library:WILEY8.LIB
SI:95 Formula:C15H24 CAS:483-76-1 MolWeight:204 RetIndex:0
CompName:NAPHTHALENE, 1,2,3,5,6,8A-HEXAHYDRO-4,7-DIMETHYL-1-(1-METHYLETHYL)-, (1S-CIS)- $$ 1-ISOPROPYL-4,7-DIMETHYL-1,

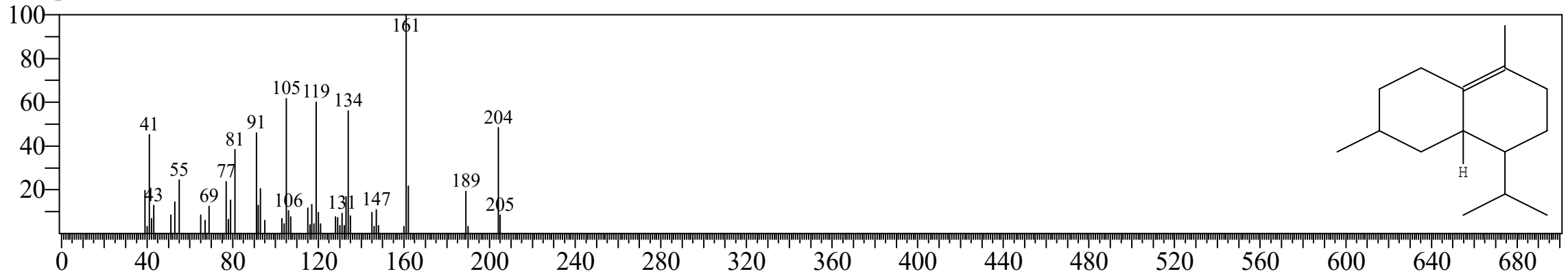

Hit#:2 Entry:109936 Library:WILEY8.LIB
SI:94 Formula:C15H24 CAS:483-76-1 MolWeight:204 RetIndex:0
CompName:NAPHTHALENE, 1,2,3,5,6,8A-HEXAHYDRO-4,7-DIMETHYL-1-(1-METHYLETHYL)-, (1S-CIS)- $$ 1-ISOPROPYL-4,7-DIMETHYL-1,

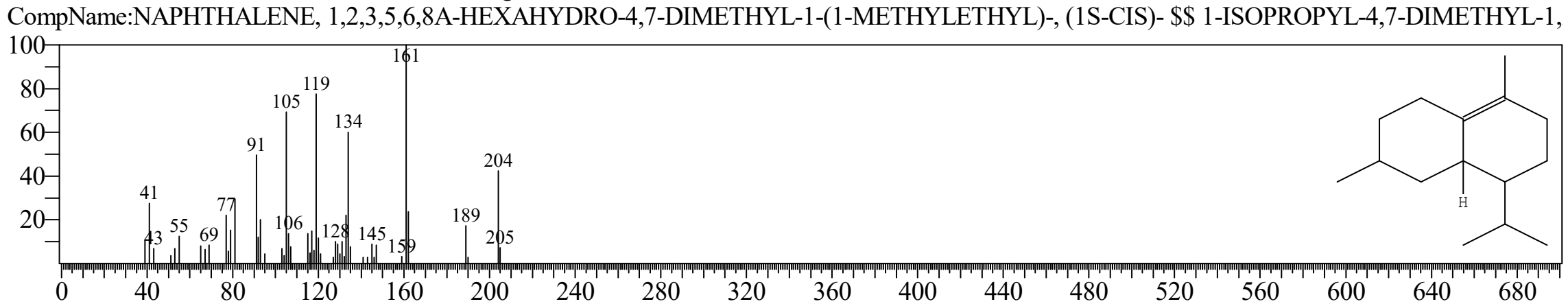

Hit#:3 Entry:19198 Library:NIST14s.lib
SI:93 Formula:C15H24 CAS:483-76-1 MolWeight:204 RetIndex:1469
CompName:Naphthalene, 1,2,3,5,6,8a-hexahydro-4,7-dimethyl-1-(1-methylethyl)-, (1S-cis)- $$ Cadina-1(10),4-diene $$ .delta.-Cadinene, (+)- $$ (+)-.delta.

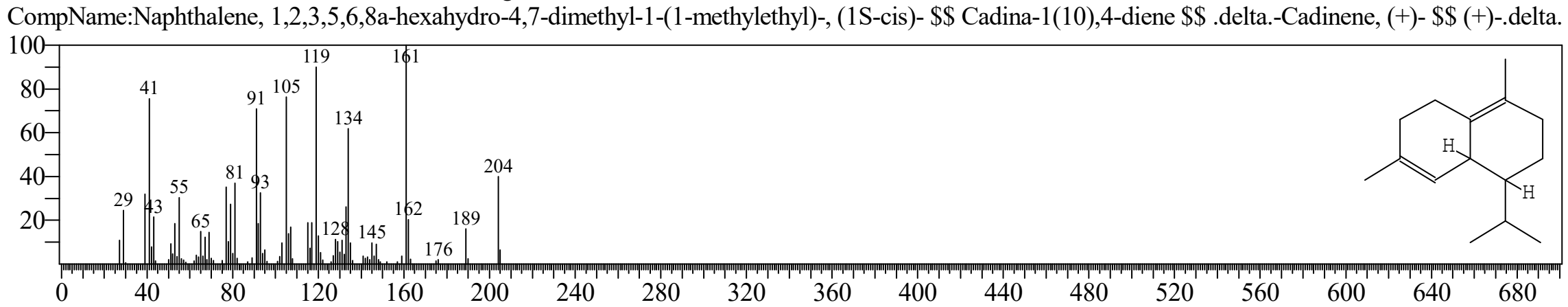

<< Target >>
Line#:82   R.Time:13.455(Scan#:1992) MassPeaks:129
RawMode:Averaged 13.450-13.460(1991-1993) BasePeak:105.10(177855)
BG Mode:Calc. from Peak Group 1 - Event 1 Scan

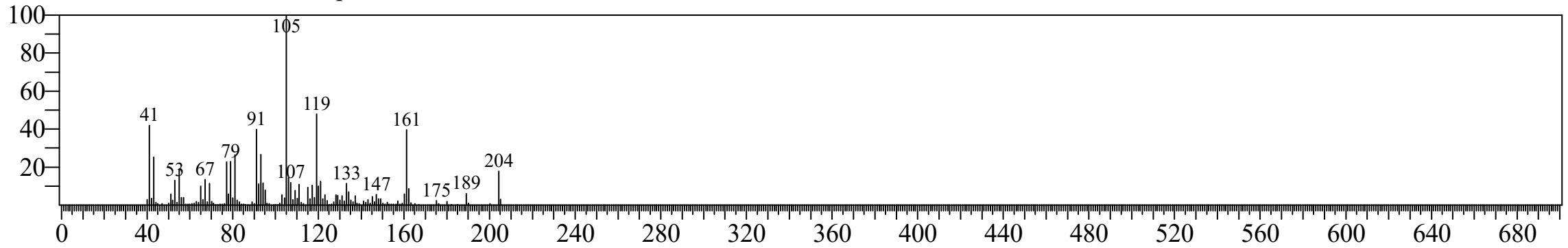

Hit#:1 Entry:19117 Library:NIST14s.lib
SI:92 Formula:C15H24 CAS:31983-22-9 MolWeight:204 RetIndex:1440
CompName:.alpha.-Muurolene $$ [1.alpha.,4a.alpha.,8a.alpha.]-1,2,4a,5,6,8a-hexahydro-4-7-dimethyl-1-[1-methylethyl]naphthalene $$ Naphthalene, 1,2,4a

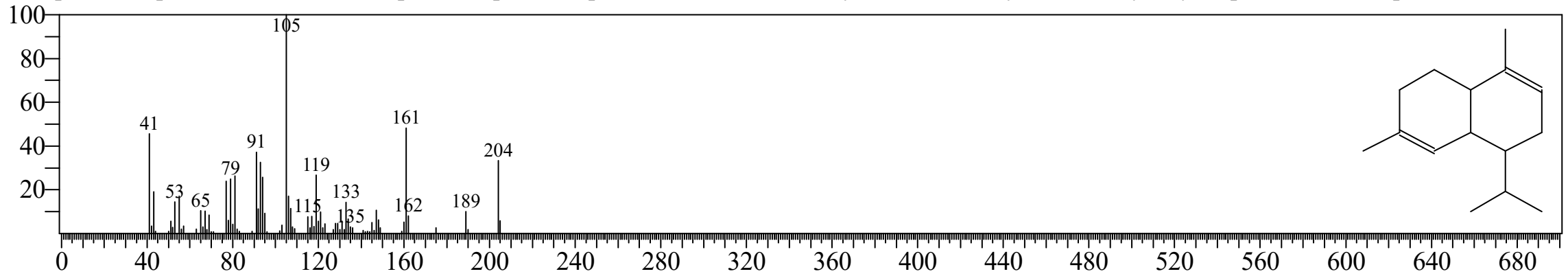

Hit#:2 Entry:110008 Library:WILEY8.LIB
SI:90 Formula:C15H24 CAS:31983-22-9 MolWeight:204 RetIndex:0
CompName:NAPHTHALENE, 1,2,4A,5,6,8A-HEXAHYDRO-4,7-DIMETHYL-1-(1-METHYLETHYL)-, (1.ALPHA.,4A.ALPHA.,8A.ALPHA.)- $$ NAP

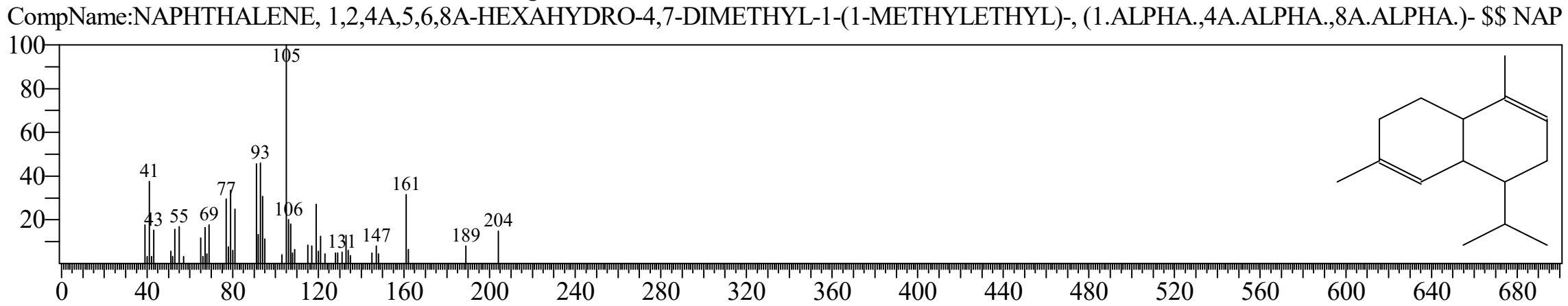

Hit#:3 Entry:110121 Library:WILEY8.LIB
SI:89 Formula:C15H24 CAS:3856-25-5 MolWeight:204 RetIndex:0
CompName:TRICYCLO[4.4.0.0(2,7)]DEC-3-ENE, 1,3-DIMETHYL-8-(1-METHYLETHYL)-, ST $$ TRICYCLO[4.4.0.0(2,7)]DEC-3-ENE, 1,3-DIMETI

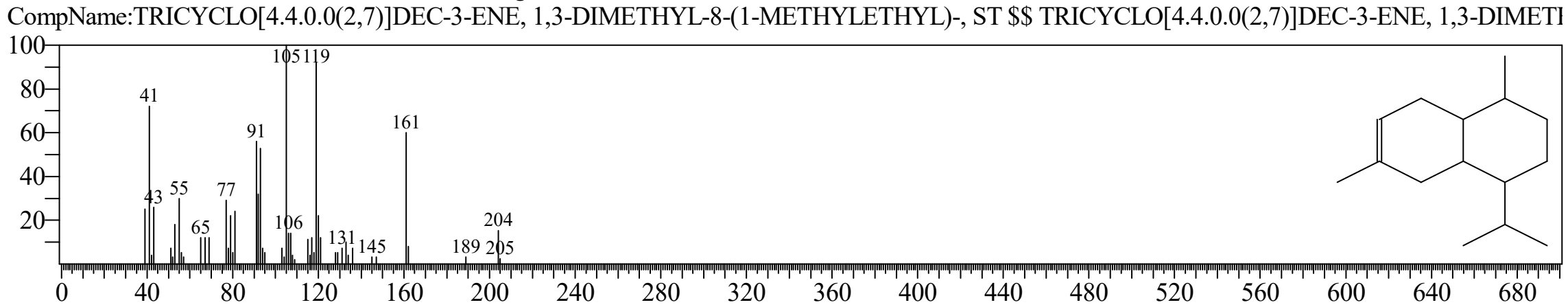

<< Target >>
Line#:83 R.Time:13.520(Scan#:2005) MassPeaks:130
RawMode:Averaged 13.515-13.525(2004-2006) BasePeak:105.10(115929)
BG Mode:Calc. from Peak Group 1 - Event 1 Scan

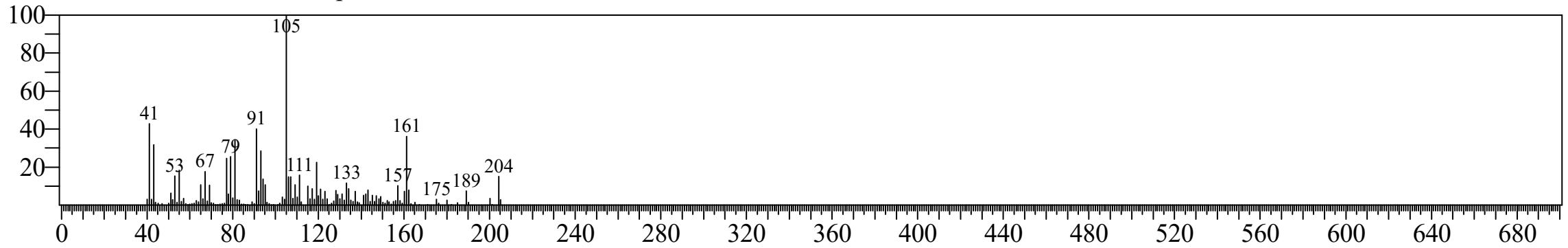

Hit#:1 Entry:110008 Library:WILEY8.LIB
SI:89 Formula:C15H24 CAS:31983-22-9 MolWeight:204 RetIndex:0
CompName:NAPHTHALENE, 1,2,4A,5,6,8A-HEXAHYDRO-4,7-DIMETHYL-1-(1-METHYLETHYL)-, (1.ALPHA.,4A.ALPHA.,8A.ALPHA.)- $$ NAP

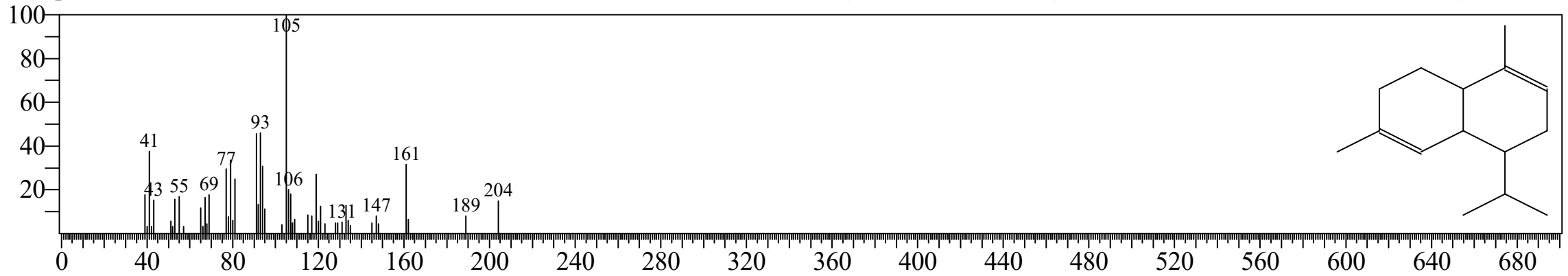

Hit#:2 Entry:19117 Library:NIST14s.lib
SI:89 Formula:C15H24 CAS:31983-22-9 MolWeight:204 RetIndex:1440
CompName:.alpha.-Muurolene $$ [1.alpha.,4a.alpha.,8a.alpha.]-1,2,4a,5,6,8a-hexahydro-4-7-dimethyl-1-[1-methylethyl]naphthalene $$ Naphthalene, 1,2,4a

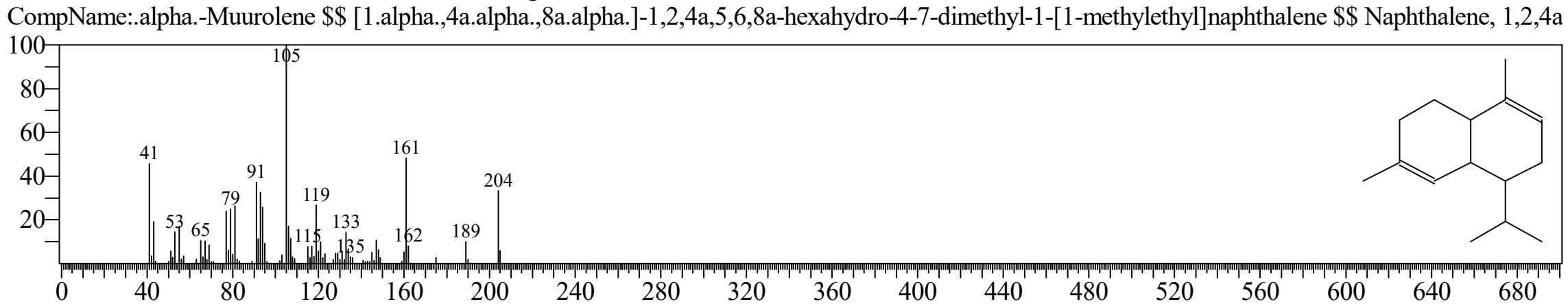

Hit#:3 Entry:110009 Library:WILEY8.LIB
SI:85 Formula:C15H24 CAS:31983-22-9 MolWeight:204 RetIndex:0
CompName:NAPHTHALENE, 1,2,4A,5,6,8A-HEXAHYDRO-4,7-DIMETHYL-1-(1-METHYLETHYL)-, (1.ALPHA.,4A.ALPHA.,8A.ALPHA.)- $$ NAP

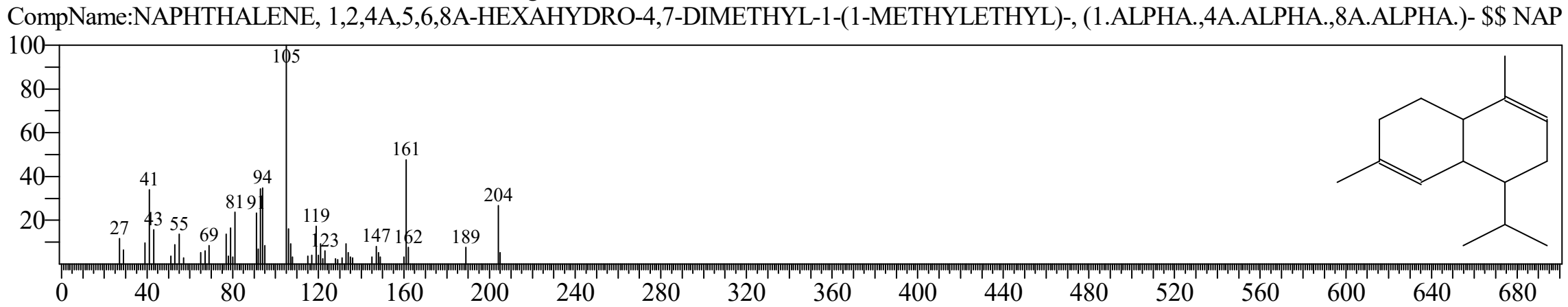

<< Target >>
Line#:84 R.Time:13.595(Scan#:2020) MassPeaks:83
RawMode:Averaged 13.590-13.600(2019-2021) BasePeak:157.15(18981)
BG Mode:Calc. from Peak Group 1 - Event 1 Scan

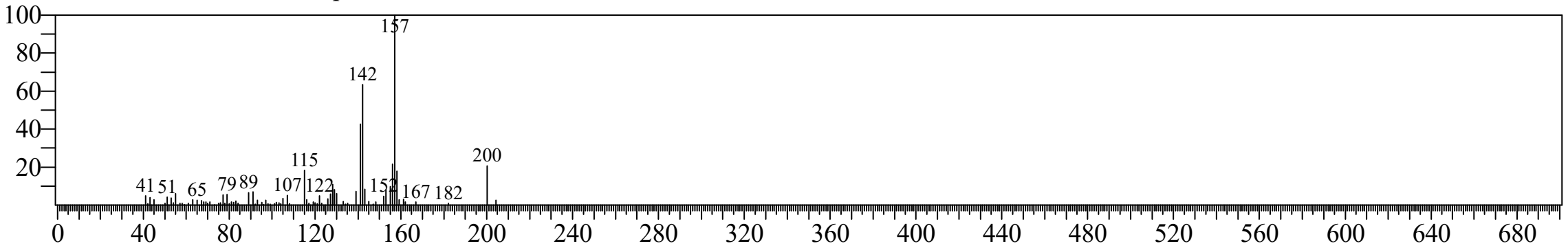

Hit#:1 Entry:104682 Library:WILEY8.LIB
SI:84 Formula:C15H20 CAS:21391-99-1 MolWeight:200 RetIndex:0
CompName:1-ISOPROPYL-4,7-DIMETHYL-1,2-DIHYDRONAPHTHALENE # $$ .ALPHA.-CALACORENE $$ 1-ISOPROPYL-4,7-DIMETHYL-1,2-I

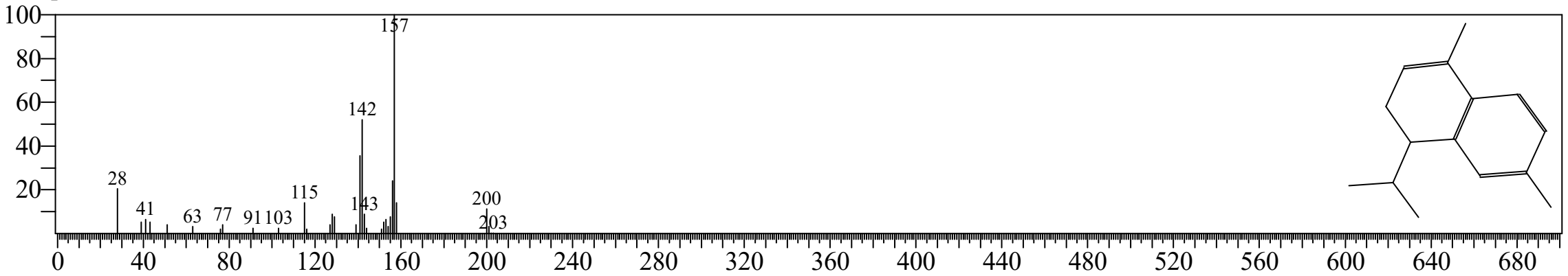

Hit#:2 Entry:47137 Library:NIST14.lib
SI:81 Formula:C15H20 CAS:21391-99-1 MolWeight:200 RetIndex:1547
CompName:.alpha.-Calacorene $$ 1-Isopropyl-4,7-dimethyl-1,2-dihydronaphthalene, (S)- $$ .alpha.-Calacorene, (+)- $$ Cadina-1,3,5,9-tetraene $$

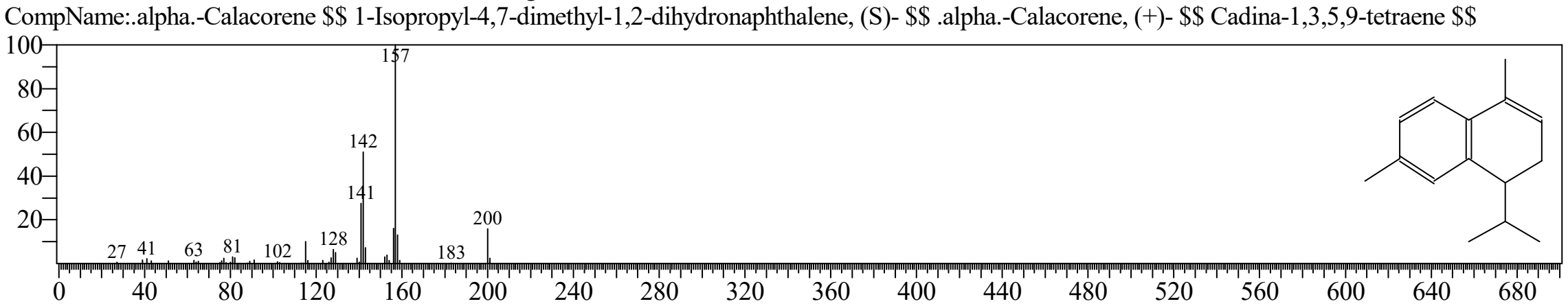

Hit#:3 Entry:18539 Library:NIST14s.lib
SI:81 Formula:C15H20 CAS:21391-99-1 MolWeight:200 RetIndex:1547
CompName:.alpha.-Calacorene $$ 1-Isopropyl-4,7-dimethyl-1,2-dihydronaphthalene, (S)- $$ .alpha.-Calacorene, (+)- $$ Cadina-1,3,5,9-tetraene $$

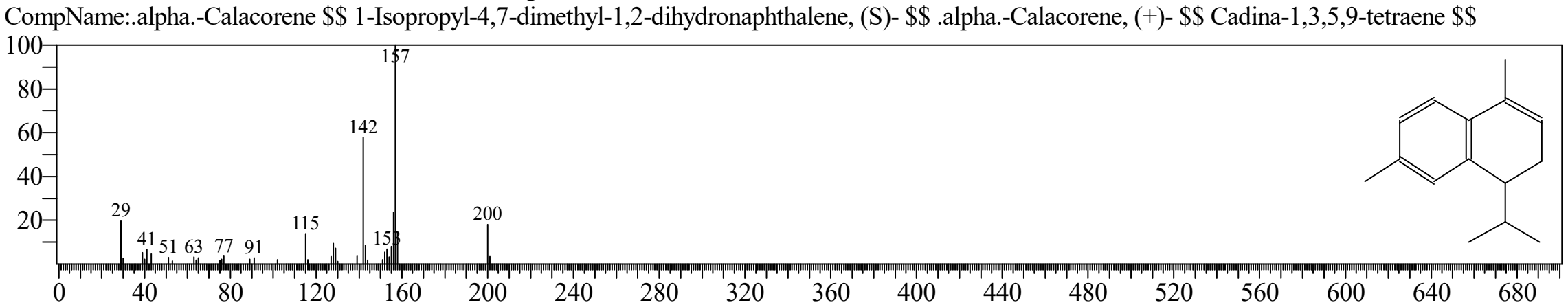

<< Target >>
Line#:85   R.Time:13.740(Scan#:2049) MassPeaks:99
RawMode:Averaged 13.735-13.745(2048-2050) BasePeak:41.05(25799)
BG Mode:Calc. from Peak Group 1 - Event 1 Scan

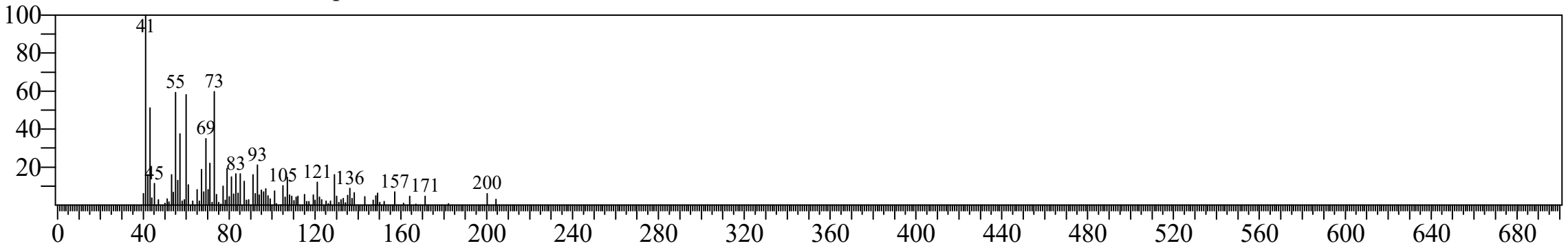

Hit#:1 Entry:104075 Library:WILEY8.LIB
SI:80 Formula:C12H24O2 CAS:143-07-7 MolWeight:200 RetIndex:0
CompName:DODECANOIC ACID $$ AMMONIUM LAURATE $$ DODECANOATE $$ LAURIC ACID $$ LITHIUM LAURATE $$ POTASSIUM DO

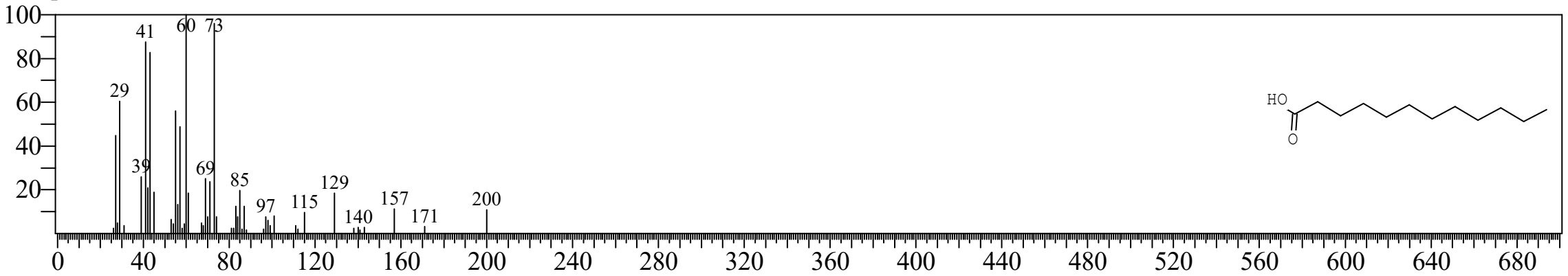

Hit#:2 Entry:286184 Library:WILEY8.LIB
SI:79 Formula:C21H29N2O2 CAS:55322-92-4 MolWeight:341 RetIndex:0
CompName:10-METHOXY-NB-.ALPHA.-METHYLCORYNANTHEOL $$ 10-METHOXY-NB-ALPHA-METHYLCORYNANTHEOL

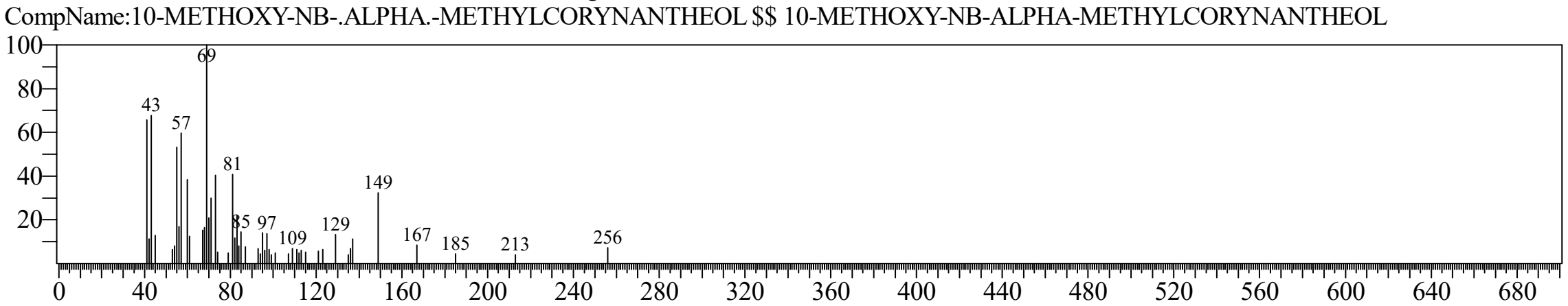

Hit#:3 Entry:16100 Library:NIST14s.lib
SI:79 Formula:C11H22O2 CAS:112-37-8 MolWeight:186 RetIndex:1471
CompName:Undecanoic acid $$ n-Undecanoic acid $$ n-Undecoic acid $$ n-Undecylic acid $$ Hendecanoic acid $$ Undecylic acid $$ 1-Decanecarboxylic

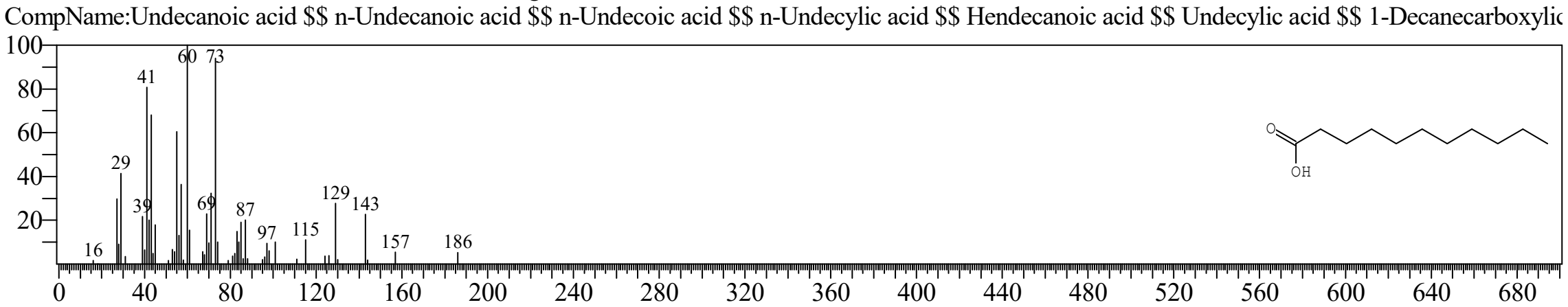

<< Target >>
Line#:86   R.Time:13.840(Scan#:2069) MassPeaks:102
RawMode:Averaged 13.835-13.845(2068-2070) BasePeak:43.00(9411)
BG Mode:Calc. from Peak Group 1 - Event 1 Scan

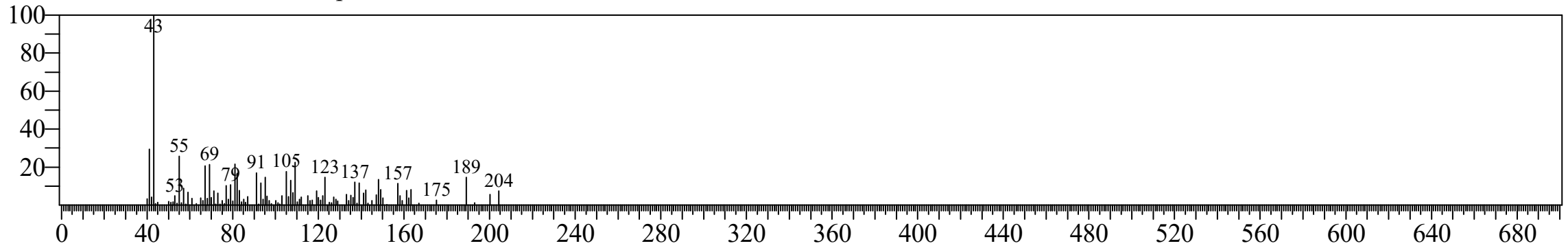

Hit#:1 Entry:21635 Library:NIST14s.lib
SI:78 Formula:C15H26O CAS:552-02-3 MolWeight:222 RetIndex:1530
CompName:1H-Cycloprop[e]azulen-4-ol, decahydro-1,1,4,7-tetramethyl-, [1aR-(1a.alpha.,4.beta.,4a.beta.,7.alpha.,7a.beta.,7b.alpha.)]- $$ Viridiflorol $$ 1,1

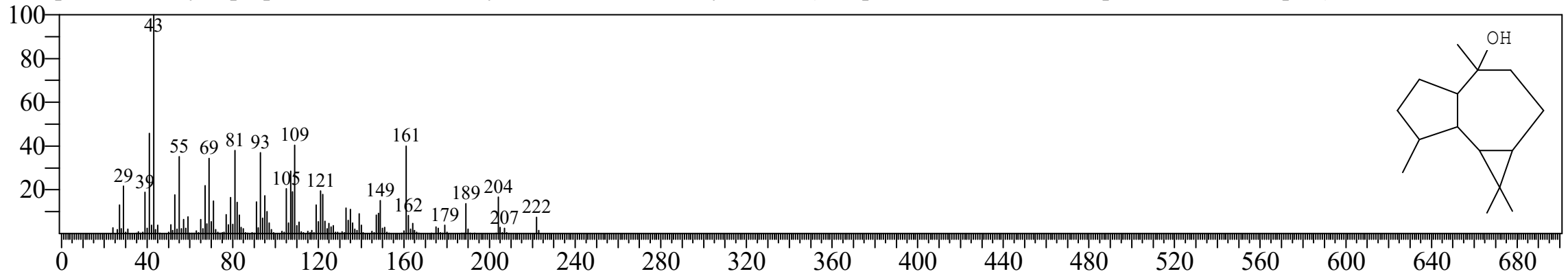

Hit#:2 Entry:136424 Library:WILEY8.LIB
SI:78 Formula:C15H26O CAS:0-00-0 MolWeight:222 RetIndex:0
CompName:1,1,4,7-TETRAMETHYLDECAHYDRO-1H-CYCLOPROPA[E]AZULEN-4-OL $$ VIRIDIFLOROL

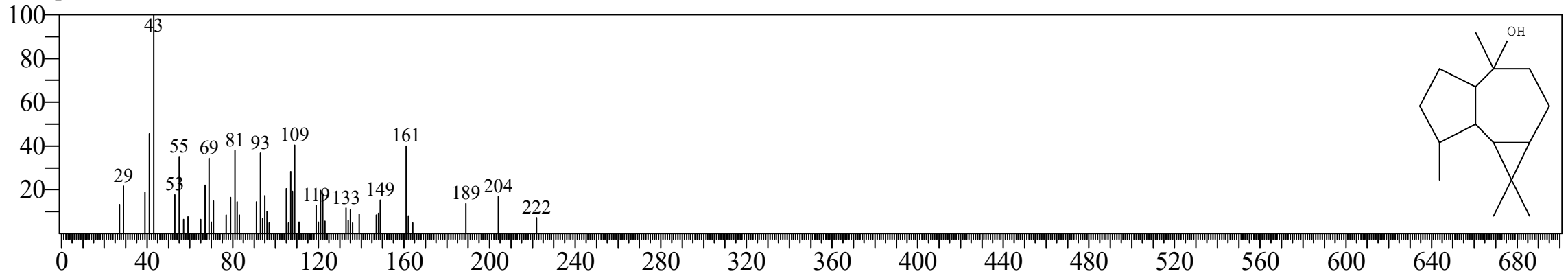

Hit#:3 Entry:136160 Library:WILEY8.LIB
SI:78 Formula:C15H26O CAS:552-02-3 MolWeight:222 RetIndex:0
CompName:1,1,4,7-TETRAMETHYLDECAHYDRO-1H-CYCLOPROPA[E]AZULEN-4-OL # $$ (1AR-(1AALPHA,4BETA,4ABETA,7ALPHA,7ABETA

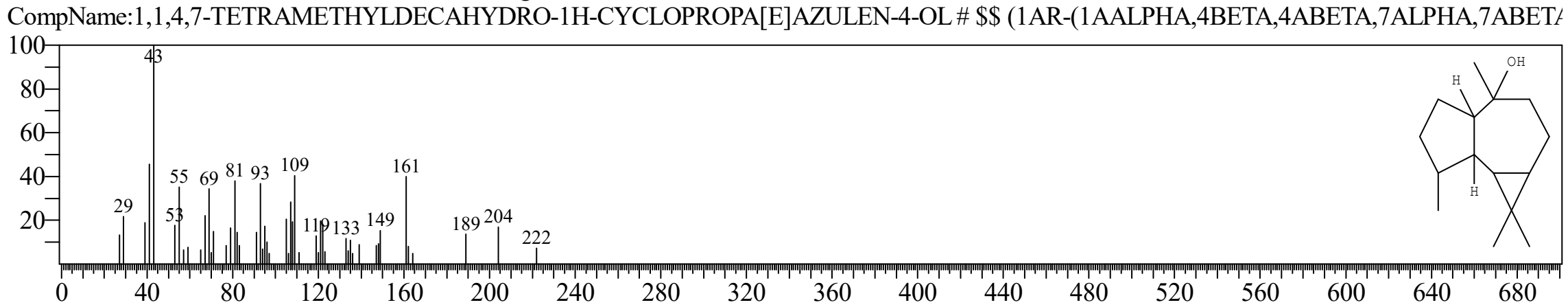

<< Target >>
Line#:87 R.Time:13.970(Scan#:2095) MassPeaks:115
RawMode:Averaged 13.965-13.975(2094-2096) BasePeak:41.05(186667)
BG Mode:Calc. from Peak Group 1 - Event 1 Scan

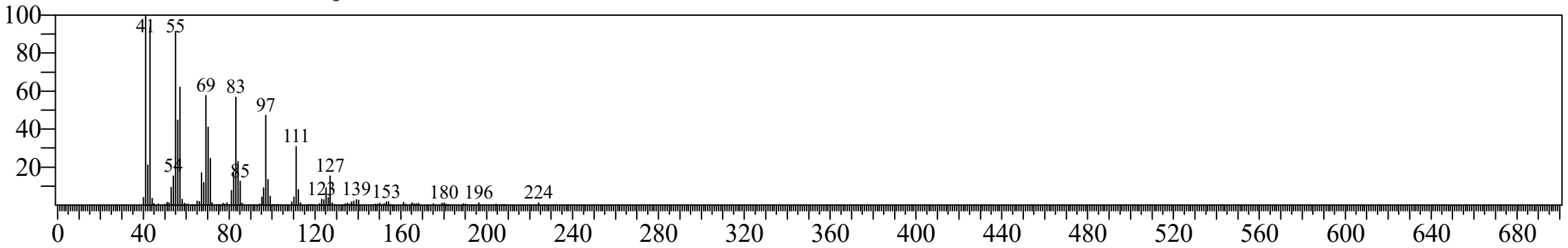

Hit#:1 Entry:21950 Library:NIST14s.lib
SI:97 Formula:C16H32 CAS:629-73-2 MolWeight:224 RetIndex:1602
CompName:Cetene $$ 1-Hexadecene $$ .alpha.-Hexadecene $$ n-Hexadec-1-ene $$ 1-Cetene $$ Hexadecylene-1 $$ Hexadec-1-ene $$ Hexadecene-1 $$ N

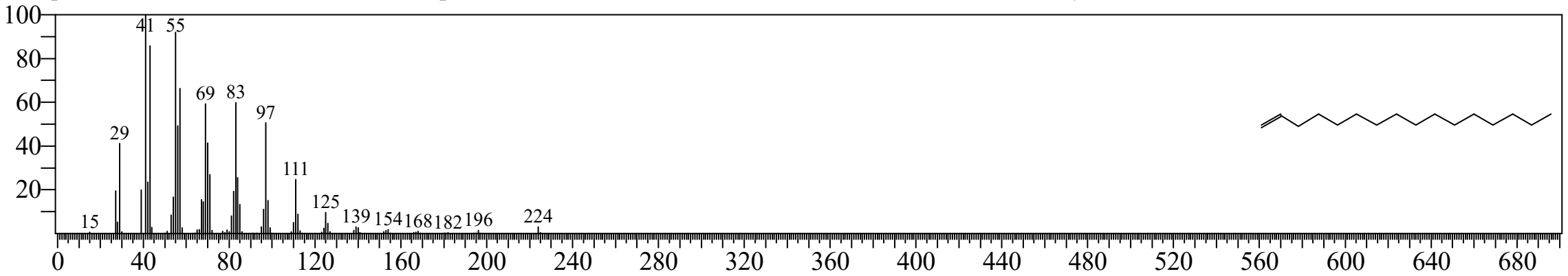

Hit#:2 Entry:139512 Library:WILEY8.LIB
SI:96 Formula:C16H32 CAS:629-73-2 MolWeight:224 RetIndex:0
CompName:1-HEXADECENE $$ HEXADEC-1-ENE $$ .ALPHA.-HEXADECENE $$ 1-CETENE $$ 1-N-HEXADECENE $$ AI3-06556 $$ ALPHA-H

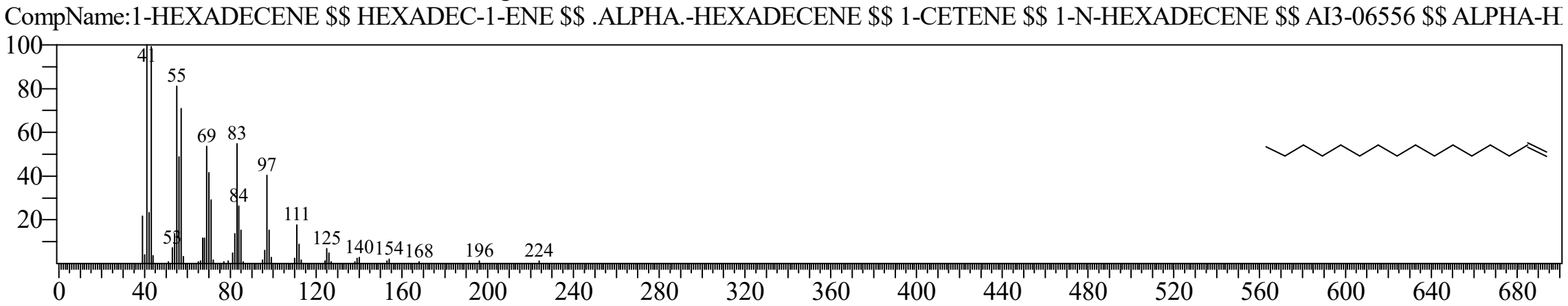

Hit#:3 Entry:79083 Library:NIST14.lib
SI:96 Formula:C16H32O CAS:64437-47-4 MolWeight:240 RetIndex:1862
CompName:Hexadecen-1-ol, trans-9- $$ (9E)-9-Hexadecen-1-ol $$ 9-Hexadecenol, E $$ (E)9-Hexadecen-1-ol $$

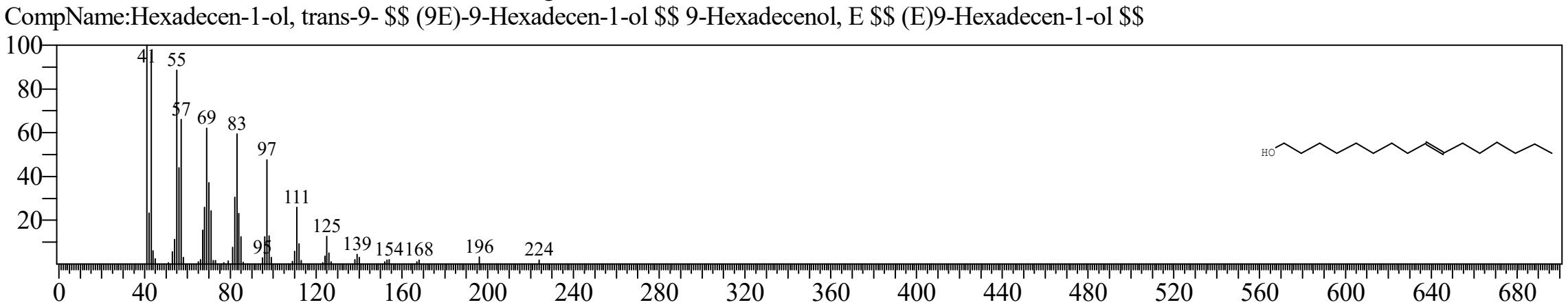

<< Target >>
Line#:88 R.Time:14.080(Scan#:2117) MassPeaks:138
RawMode:Averaged 14.075-14.085(2116-2118) BasePeak:43.05(260353)
BG Mode:Calc. from Peak Group 1 - Event 1 Scan

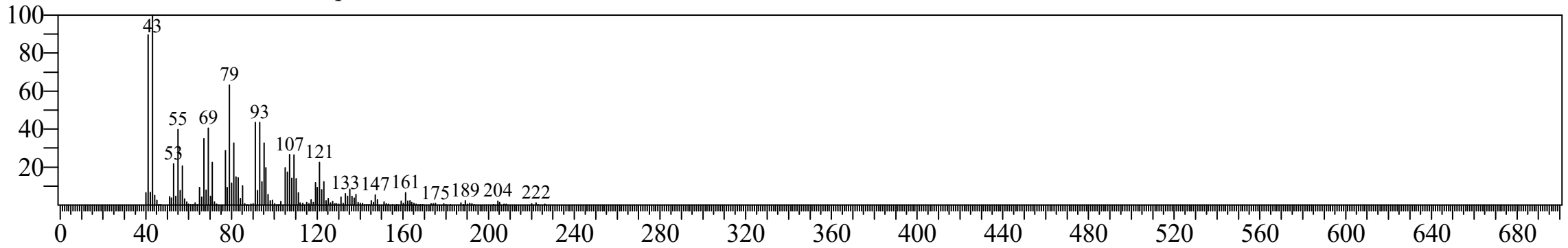

Hit#:1 Entry:132802 Library:WILEY8.LIB
SI:93 Formula:C15H24O CAS:1139-30-6 MolWeight:220 RetIndex:0
CompName:(-)-5-OXATRICYCLO[8.2.0.0(4,6)]DODECANE,,12-TRIMETHYL-9-METHYLENE-, [1R-(1R*,4R*,6R*,10S*)]- $$ 5-OXATRICYCLO[8.

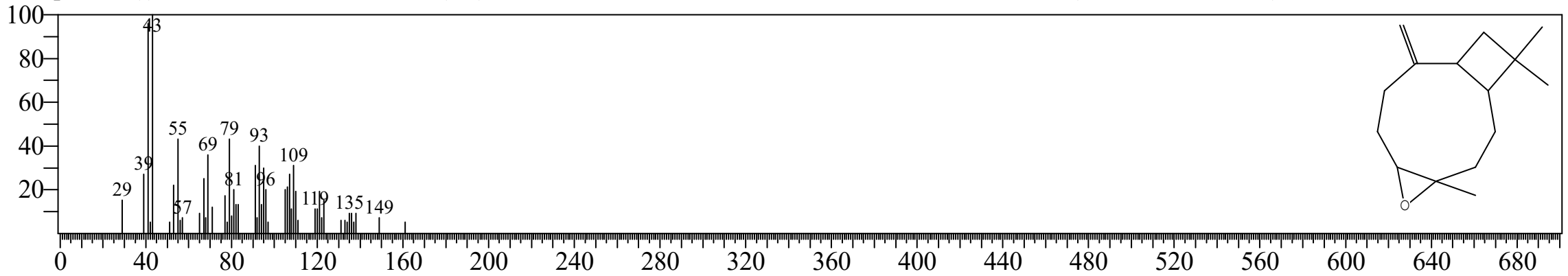

Hit#:2 Entry:62421 Library:NIST14.lib
SI:91 Formula:C15H24O CAS:1139-30-6 MolWeight:220 RetIndex:1507
CompName:Caryophyllene oxide $$ 5-Oxatricyclo[8.2.0.0(4,6)-]dodecane, 4,12,12-trimethyl-9-methylene-, [1R-(1R*,4R*,6R*,10S*)]- $$ 5-Oxatricyclo(8.

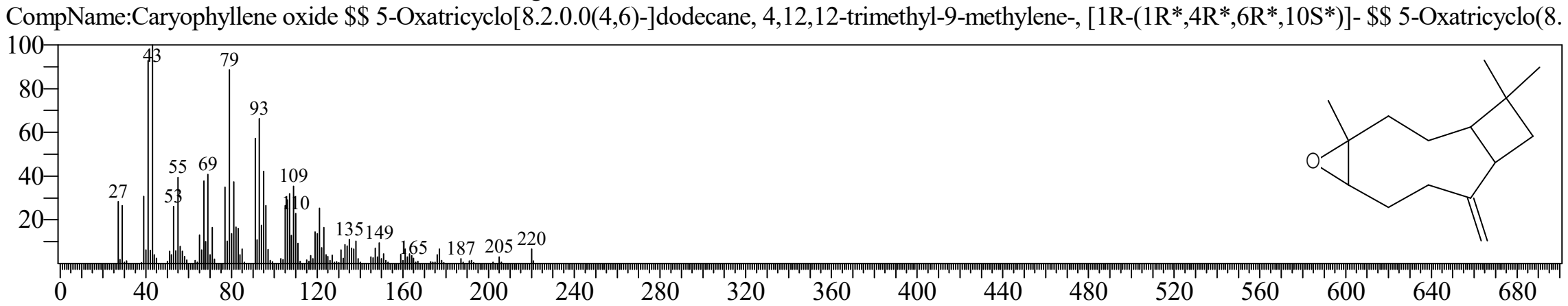

Hit#:3 Entry:133096 Library:WILEY8.LIB
SI:91 Formula:C15H24O CAS:0-00-0 MolWeight:220 RetIndex:0
CompName:4,12,12-TRIMETHYL-9-METHYLENE-5-OXATRICYCLO[8.2.0.0~4,6~]DODECANE $$ CARYOPHYLLENOXID

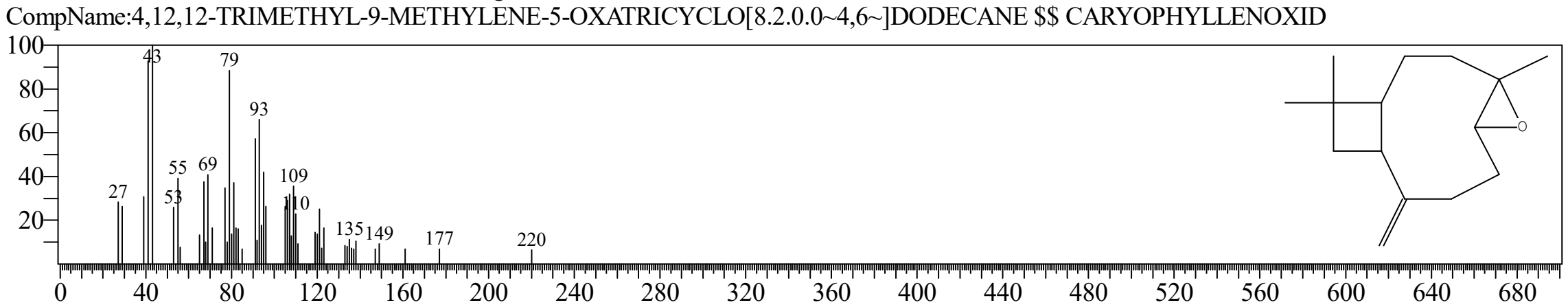

<< Target >>
Line#:89   R.Time:14.140(Scan#:2129) MassPeaks:148
RawMode:Averaged 14.135-14.145(2128-2130) BasePeak:149.10(1129441)
BG Mode:Calc. from Peak Group 1 - Event 1 Scan

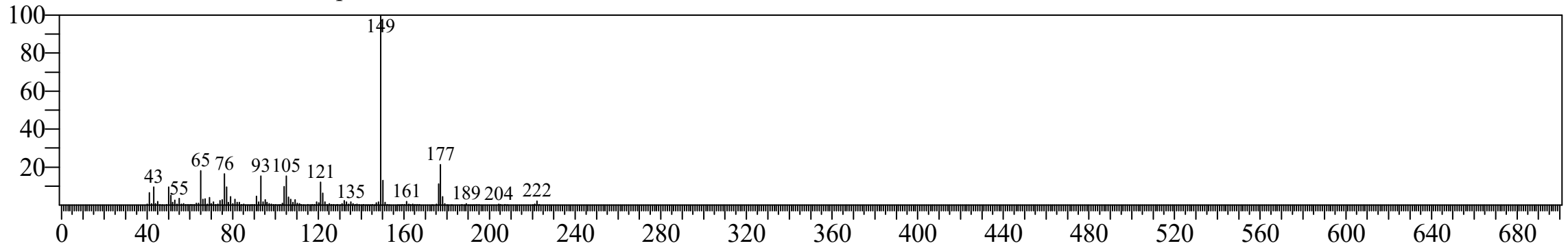

Hit#:1 Entry:134974 Library:WILEY8.LIB
SI:88 Formula:C12H14O4 CAS:84-66-2 MolWeight:222 RetIndex:0
CompName:1,2-BENZENEDICARBOXYLIC ACID, DIETHYL ESTER $$ PHTHALIC ACID $$ -BENZENEDICARBOXYLIC ACID DIETHYL ESTE

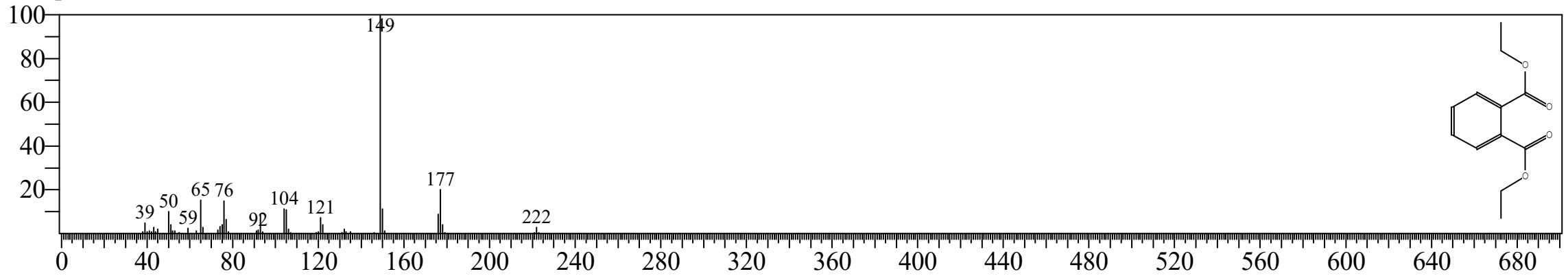

Hit#:2 Entry:21541 Library:NIST14s.lib
SI:88 Formula:C12H14O4 CAS:84-66-2 MolWeight:222 RetIndex:1639
CompName:Diethyl Phthalate $$ 1,2-Benzenedicarboxylic acid, diethyl ester $$ Phthalic acid, diethyl ester $$ o-Benzenedicarboxylic acid, diethyl ester $$

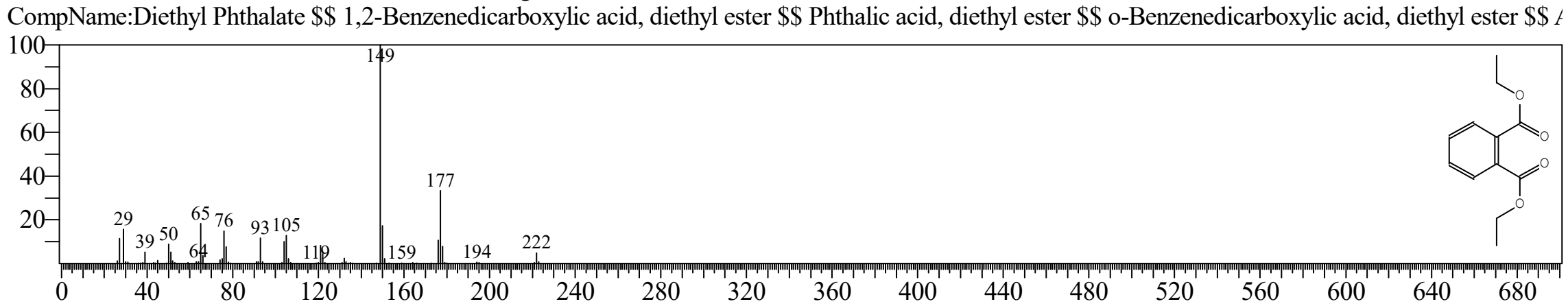

Hit#:3 Entry:134826 Library:WILEY8.LIB
SI:88 Formula:C12H14O4 CAS:84-66-2 MolWeight:222 RetIndex:0
CompName:1,2-BENZENEDICARBOXYLIC ACID, DIETHYL ESTER $$ PHTHALIC ACID $$ -BENZENEDICARBOXYLIC ACID DIETHYL ESTE

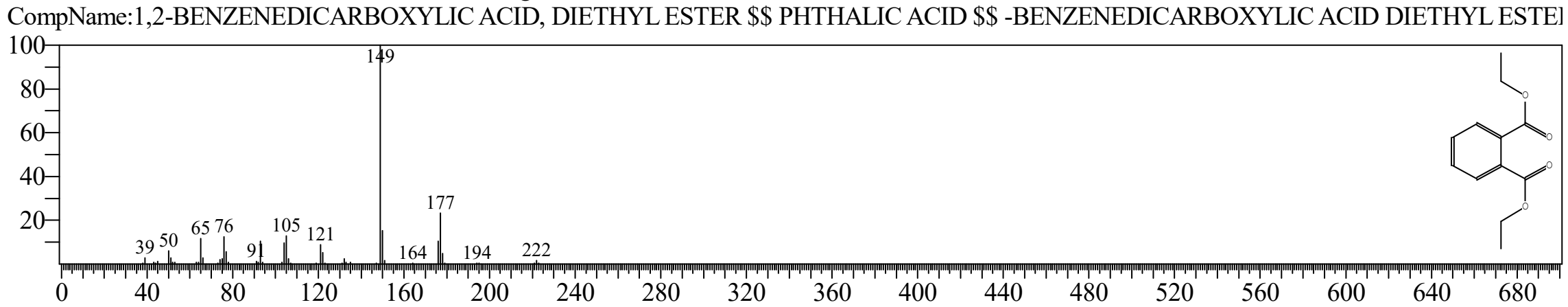

<< Target >>
Line#:90 R.Time:14.325(Scan#:2166) MassPeaks:110
RawMode:Averaged 14.320-14.330(2165-2167) BasePeak:43.00(25234)
BG Mode:Calc. from Peak Group 1 - Event 1 Scan

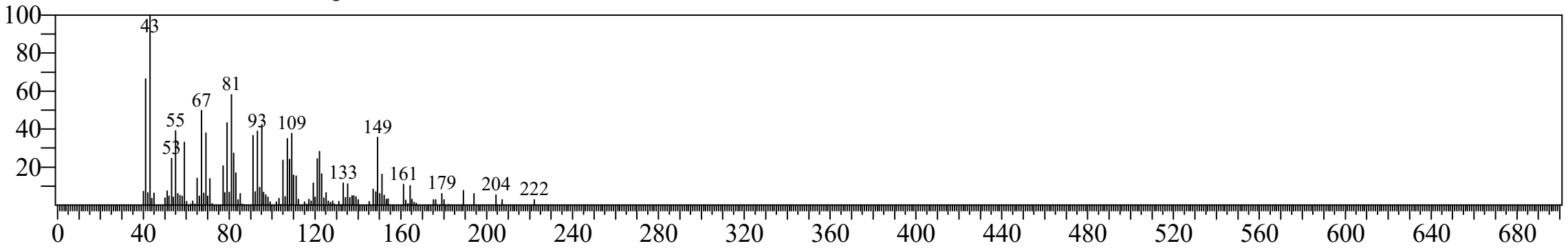

Hit#:1 Entry:136421 Library:WILEY8.LIB
SI:86 Formula:C15H26O CAS:577-27-5 MolWeight:222 RetIndex:0
CompName:1H-CYCLOPROP[E]AZULEN-4-OL, DECAHYDRO-1,1,4,7-TETRAMETHYL-, [1AR-(1A.ALPHA.,4.ALPHA.,4A.BETA.,7.ALPHA.,7A.B

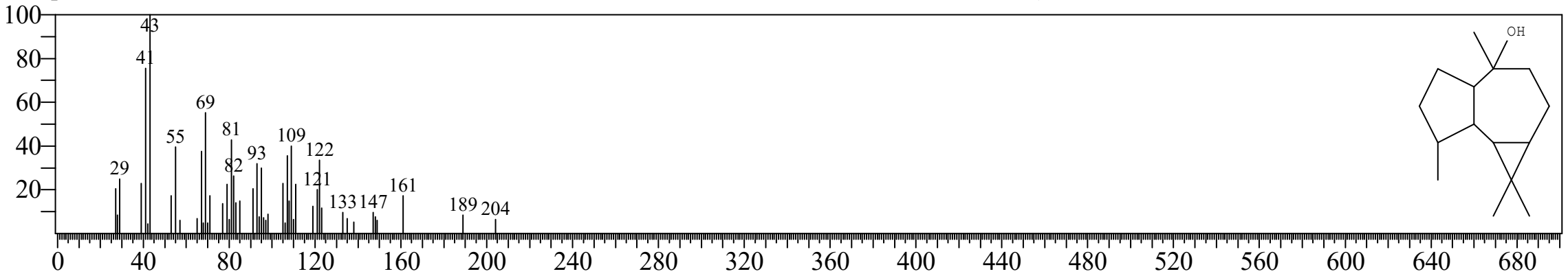

Hit#:2 Entry:21632 Library:NIST14s.lib
SI:86 Formula:C15H26O CAS:577-27-5 MolWeight:222 RetIndex:1530
CompName:Ledol $$ 1H-Cycloprop[e]azulen-4-ol, decahydro-1,1,4,7-tetramethyl-, [1ar-(1a.alpha.,4.alpha.,4a.beta.,7.alpha.,7a.beta.,7b.alpha.)]- $$ 1H-Cyc

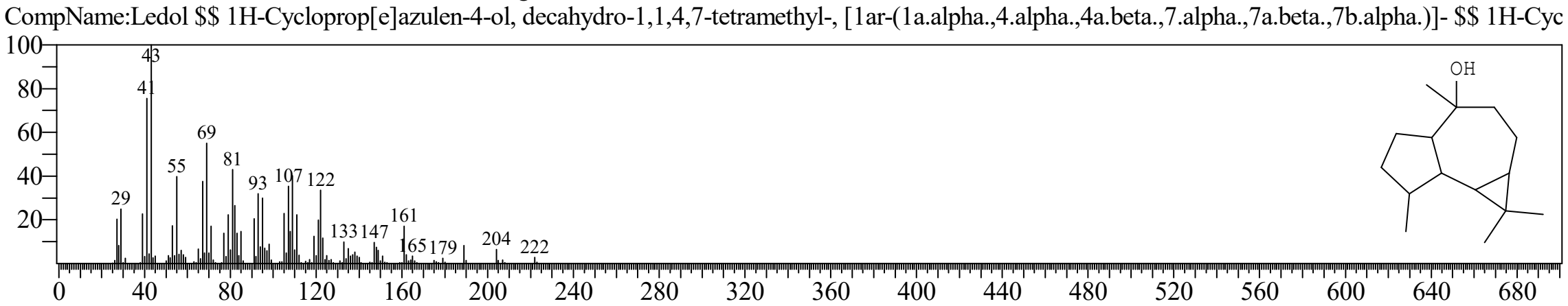

Hit#:3 Entry:136140 Library:WILEY8.LIB
SI:86 Formula:C15H26O CAS:577-27-5 MolWeight:222 RetIndex:0
CompName:1H-CYCLOPROP[E]AZULEN-4-OL, DECAHYDRO-1,1,4,7-TETRAMETHYL-, [1AR-(1A.ALPHA.,4.ALPHA.,4A.BETA.,7.ALPHA.,7A.B

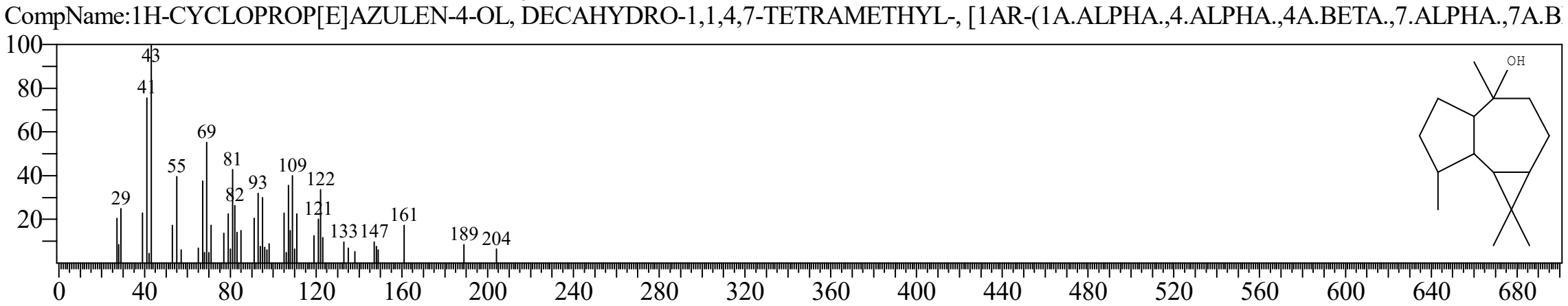

<< Target >>
Line#:91 R.Time:14.430(Scan#:2187) MassPeaks:121
RawMode:Averaged 14.425-14.435(2186-2188) BasePeak:41.05(58265)
BG Mode:Calc. from Peak Group 1 - Event 1 Scan

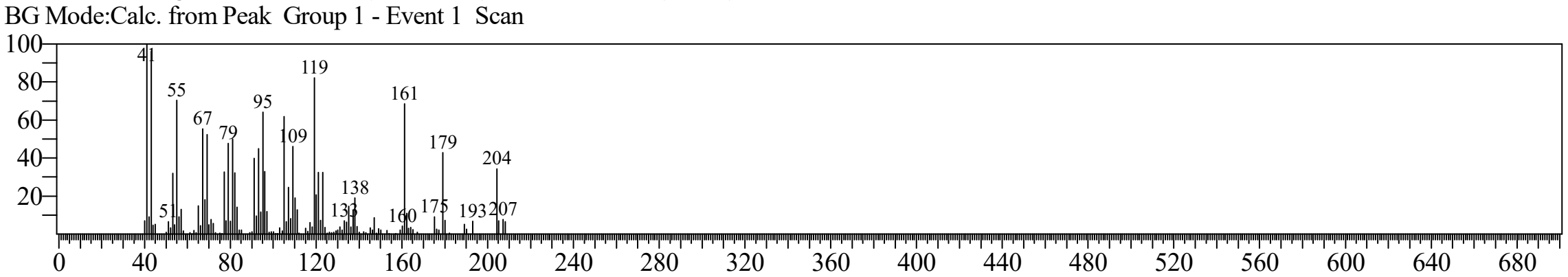

Hit#:1 Entry:64391 Library:NIST14.lib
SI:86 Formula:C15H26O CAS:21284-22-0 MolWeight:222 RetIndex:1580
CompName:Cubenol $$ 1-Isopropyl-4,7-dimethyl-1,3,4,5,6,8a-hexahydro-4a(2H)-naphthalenol-, [1S-(1.alpha.,4.beta.,4a.beta.,8a.alpha.)]- $$ (-)-Cubenol $$

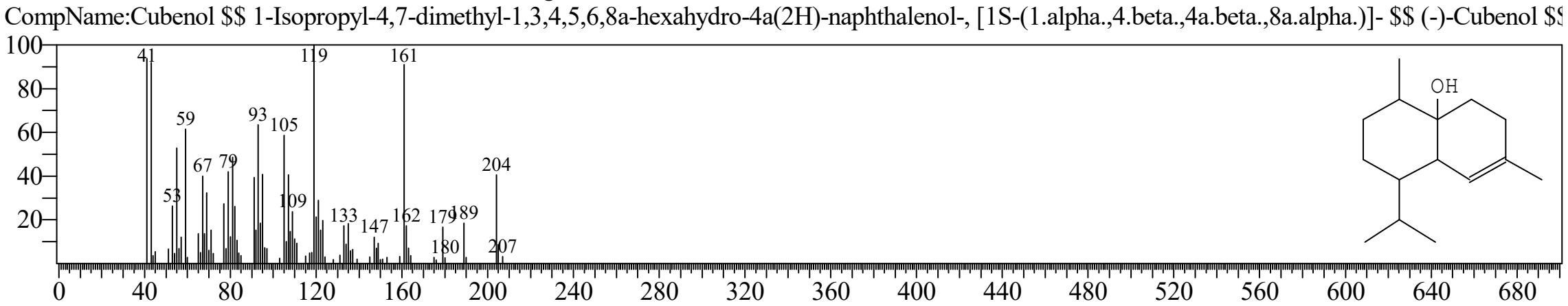

Hit#:2 Entry:64397 Library:NIST14.lib
SI:84 Formula:C15H26O CAS:19912-67-5 MolWeight:222 RetIndex:0
CompName:Epicubenol $$ (1S,4R,4aS,8aR)-1-Isopropyl-4,7-dimethyl-1,2,3,4,4a,5,6,8a-octahydronaphthalen-4a-ol $$ epi-Cubenol $$ 4a(2H)-Naphthaleno

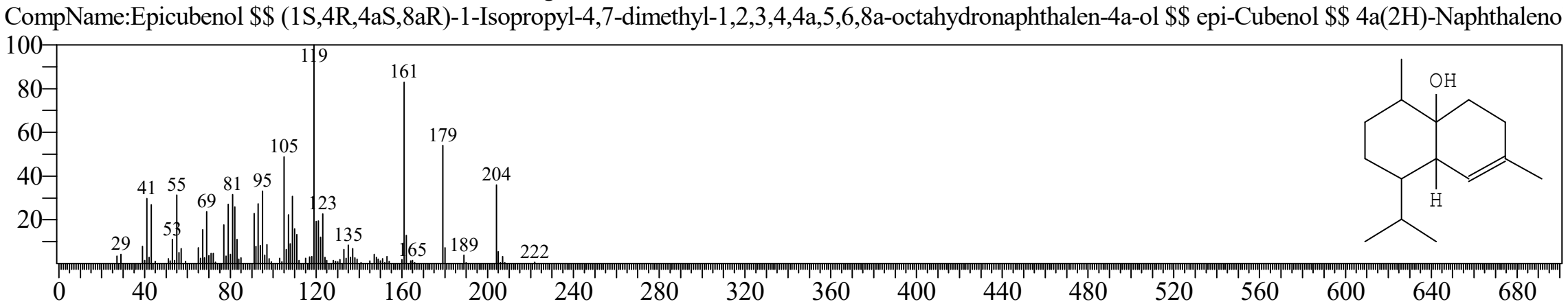

Hit#:3 Entry:64345 Library:NIST14.lib
SI:83 Formula:C15H26O CAS:0-00-0 MolWeight:222 RetIndex:1523
CompName:7-epi-cis-sesquisabinene hydrate

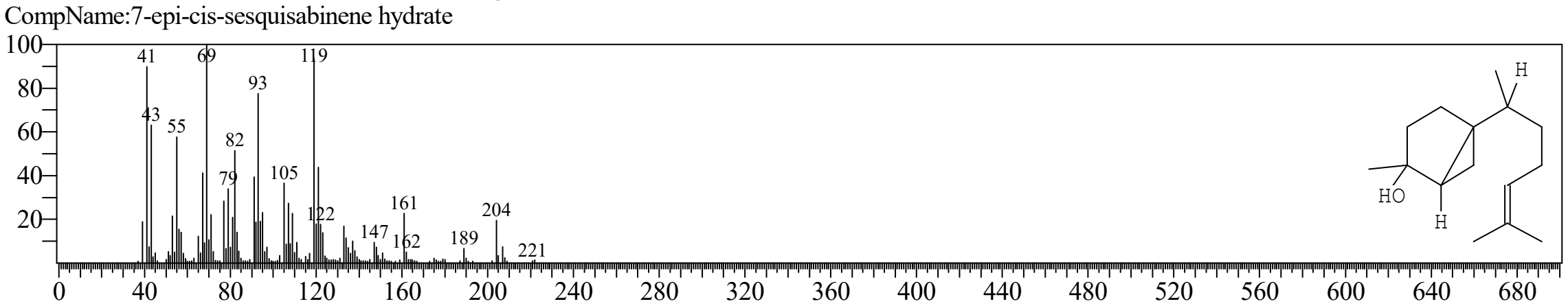

<< Target >>
Line#:92   R.Time:14.580(Scan#:2217) MassPeaks:135
RawMode:Averaged 14.575-14.585(2216-2218) BasePeak:119.10(114934)
BG Mode:Calc. from Peak Group 1 - Event 1 Scan

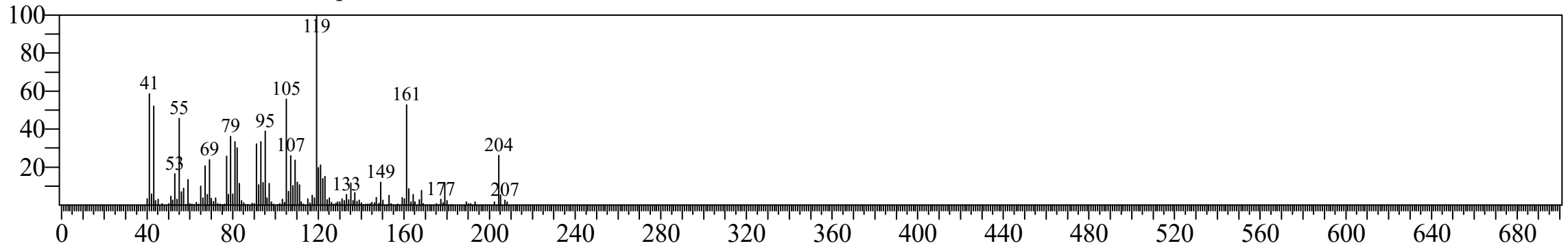

Hit#:1 Entry:64397 Library:NIST14.lib
SI:88 Formula:C15H26O CAS:19912-67-5 MolWeight:222 RetIndex:0
CompName:Epicubenol $$ (1S,4R,4aS,8aR)-1-Isopropyl-4,7-dimethyl-1,2,3,4,4a,5,6,8a-octahydronaphthalen-4a-ol $$ epi-Cubenol $$ 4a(2H)-Naphthaleno

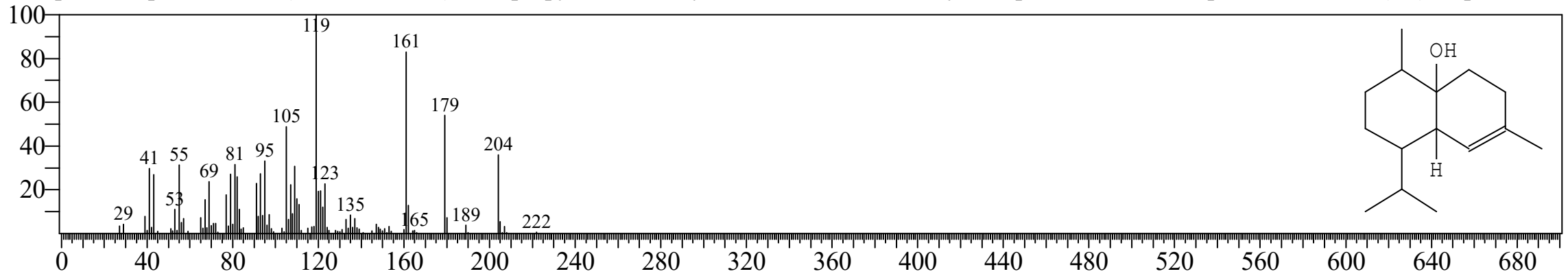

Hit#:2 Entry:64391 Library:NIST14.lib
SI:87 Formula:C15H26O CAS:21284-22-0 MolWeight:222 RetIndex:1580
CompName:Cubenol $$ 1-Isopropyl-4,7-dimethyl-1,3,4,5,6,8a-hexahydro-4a(2H)-naphthalenol-, [1S-(1.alpha.,4.beta.,4a.beta.,8a.alpha.)]- $$ (-)-Cubenol $$

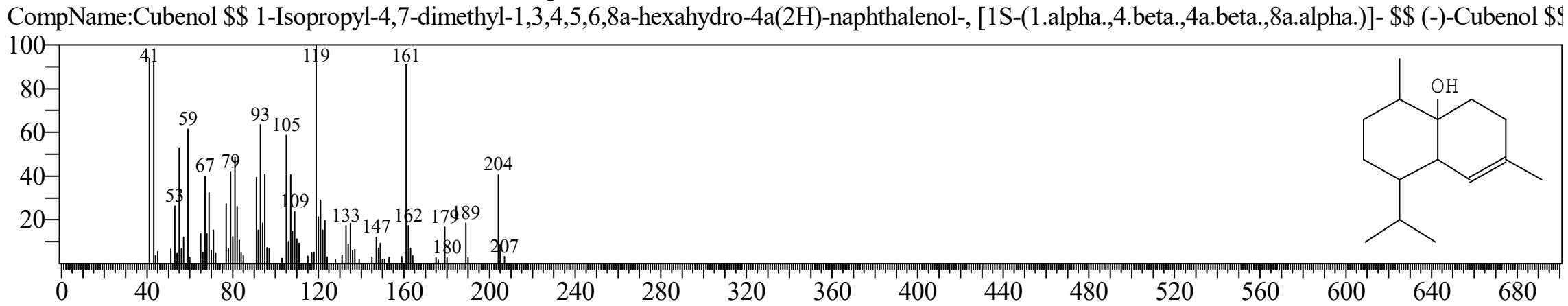

Hit#:3 Entry:21675 Library:NIST14s.lib
SI:85 Formula:C15H26O CAS:145512-84-1 MolWeight:222 RetIndex:0
CompName:trans-Sesquisabinene hydrate $$ (1R,2R,5S)-2-Methyl-5-(6-methylhept-5-en-2-yl)bicyclo[3.1.0]hexan-2-ol, (relative configuration) $$ Bicyclo[

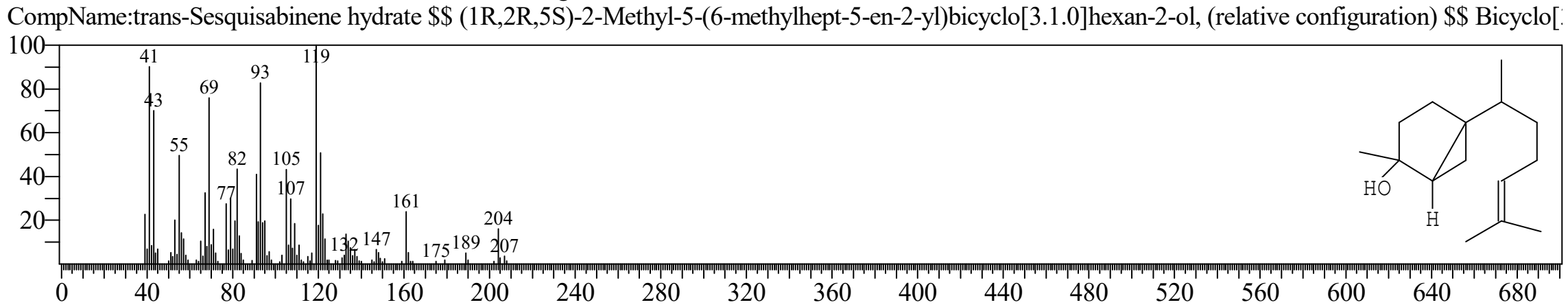

<< Target >>
Line#:93   R.Time:14.745(Scan#:2250) MassPeaks:149
RawMode:Averaged 14.740-14.750(2249-2251) BasePeak:161.15(894910)
BG Mode:Calc. from Peak Group 1 - Event 1 Scan

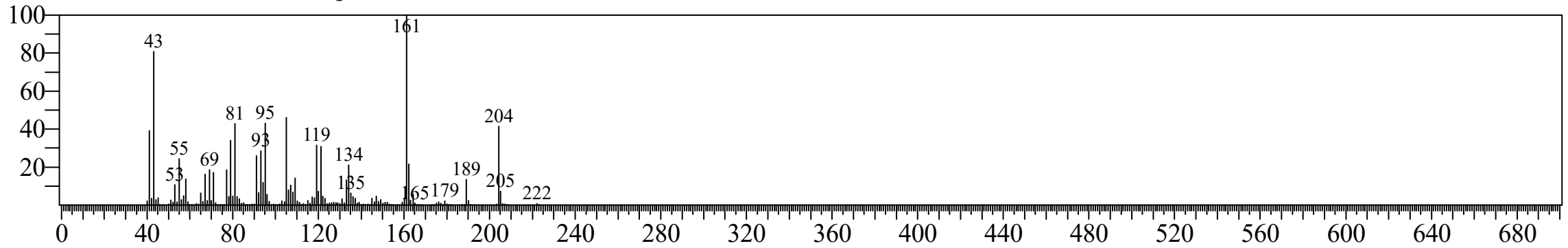

Hit#:1 Entry:64417 Library:NIST14.lib
SI:96 Formula:C15H26O CAS:5937-11-1 MolWeight:222 RetIndex:1580
CompName:.tau.-Cadinol $$ 4-Isopropyl-1,6-dimethyl-1,2,3,4,4a,7,8,8a-octahydro-1-naphthalenol-, (1S-(1alpha,4alpha,4aalpha,8abeta))- $$ (1S,4S,4aR,8al

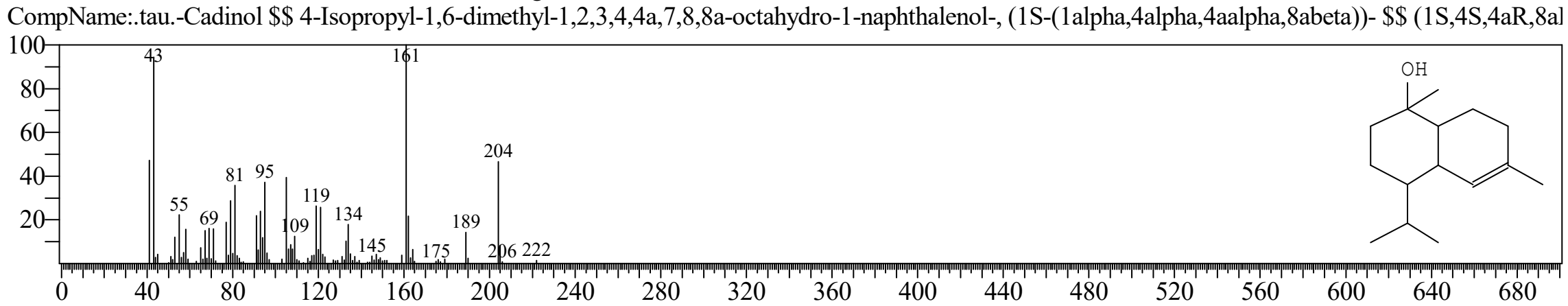

Hit#:2 Entry:136081 Library:WILEY8.LIB
SI:93 Formula:C15H26O CAS:19435-97-3 MolWeight:222 RetIndex:0
CompName:1-NAPHTHALENOL, 1,2,3,4,4A,7,8,8A-OCTAHYDRO-1,6-DIMETHYL-4-(1-METHYLETHYL)-, [1R-(1.ALPHA.,4.BETA.,4A.BETA.,8A.

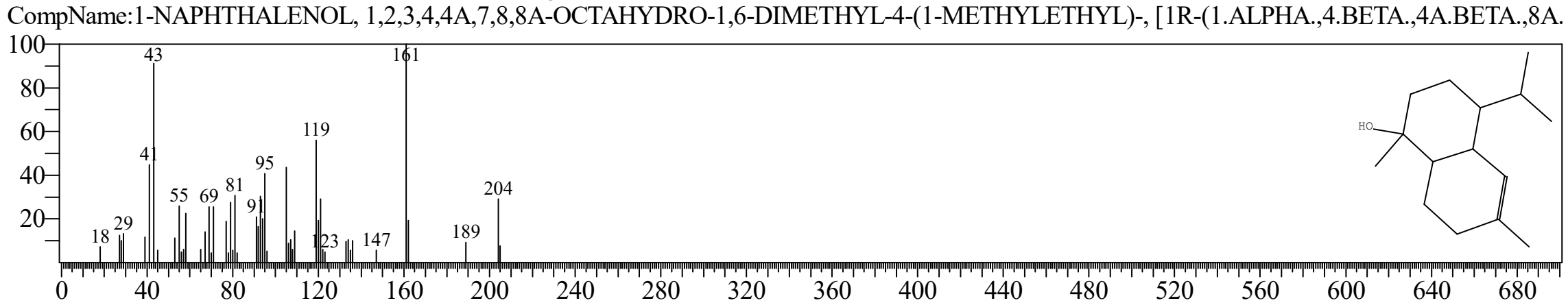

Hit#:3 Entry:64418 Library:NIST14.lib
SI:93 Formula:C15H26O CAS:19435-97-3 MolWeight:222 RetIndex:1580
CompName:1-Naphthalenol, 1,2,3,4,4a,7,8,8a-octahydro-1,6-dimethyl-4-(1-methylethyl)-, [1R-(1.alpha.,4.beta.,4a.beta.,8a.beta.)]- $$ (1R,4S,4aR,8aS)-4-Is

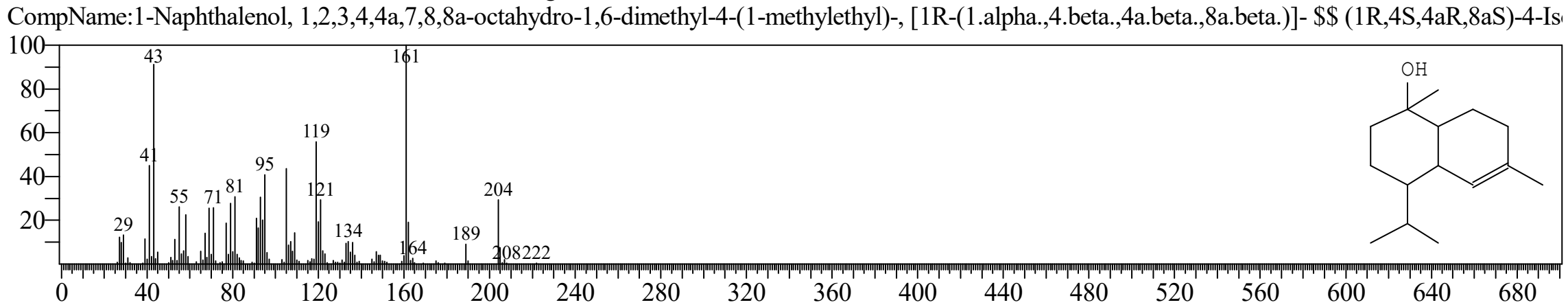

<< Target >>
Line#:94 R.Time:14.910(Scan#:2283) MassPeaks:146
RawMode:Averaged 14.905-14.915(2282-2284) BasePeak:43.00(472852)
BG Mode:Calc. from Peak Group 1 - Event 1 Scan

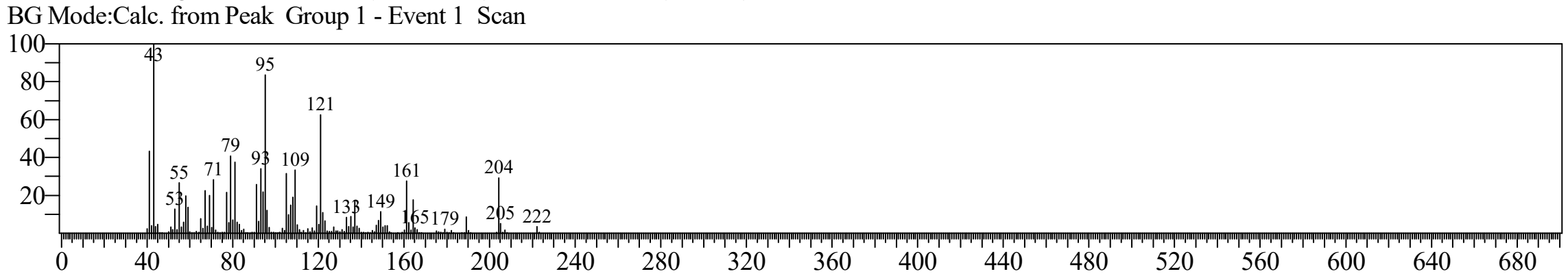

Hit#:1 Entry:64366 Library:NIST14.lib
SI:94 Formula:C15H26O CAS:481-34-5 MolWeight:222 RetIndex:1580
CompName:.alpha.-Cadinol $$ 4-Isopropyl-1,6-dimethyl-1,2,3,4,4a,7,8,8a-octahydro-1-naphthalenol # $$ (1R,4S,4aR,8aR)-4-Isopropyl-1,6-dimethyl-1,2,3,

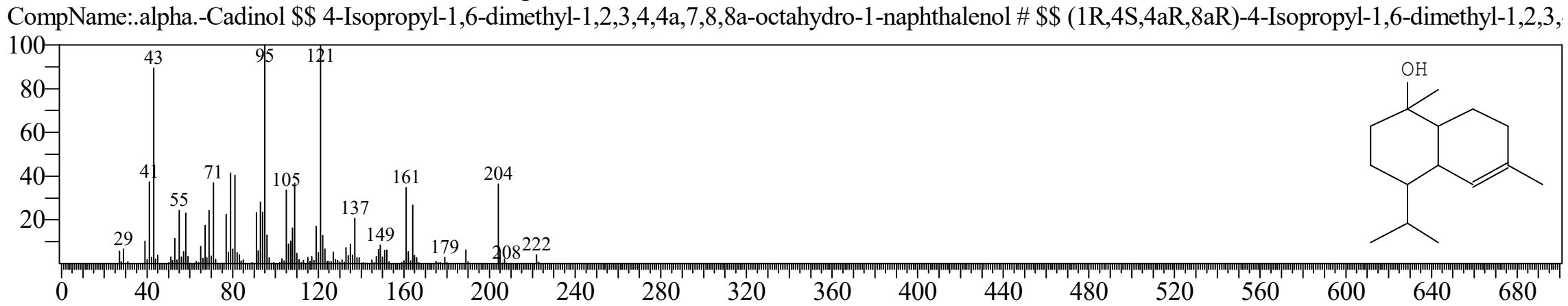

Hit#:2 Entry:21636 Library:NIST14s.lib
SI:91 Formula:C15H26O CAS:481-34-5 MolWeight:222 RetIndex:1580
CompName:.alpha.-Cadinol $$ 4-Isopropyl-1,6-dimethyl-1,2,3,4,4a,7,8,8a-octahydro-1-naphthalenol # $$ (1R,4S,4aR,8aR)-4-Isopropyl-1,6-dimethyl-1,2,3,

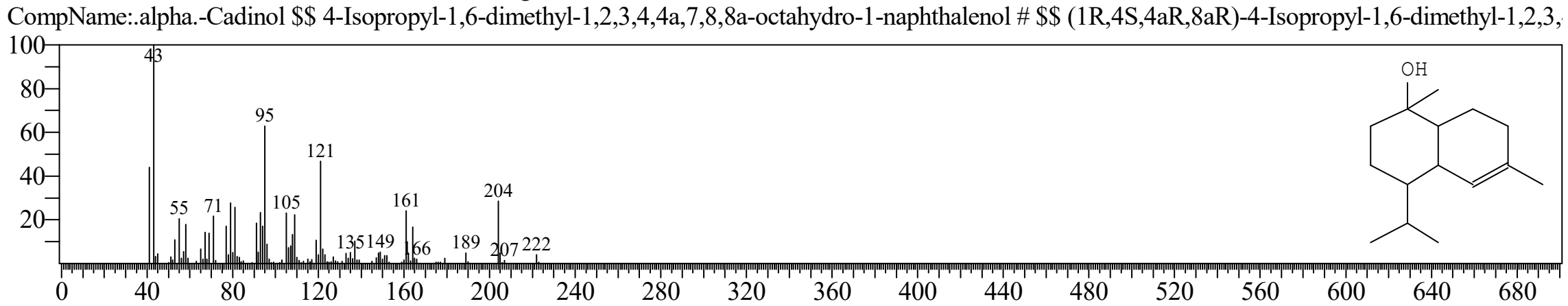

Hit#:3 Entry:64367 Library:NIST14.lib
SI:91 Formula:C15H26O CAS:19912-62-0 MolWeight:222 RetIndex:1580
CompName:.tau.-Muurolol $$ 4-Isopropyl-1,6-dimethyl-1,2,3,4,4a,7,8,8a-octahydro-1-naphthalenol-, [1S-(1.alpha.,4.alpha.,4a.alpha.,8a.alpha.)]- $$ .alpha.-

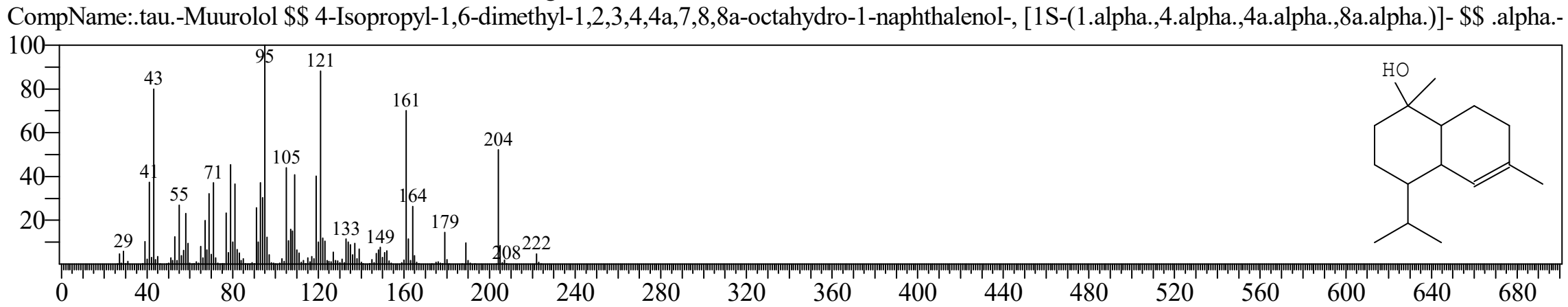

<< Target >>
Line#:95   R.Time:15.110(Scan#:2323) MassPeaks:89
RawMode:Averaged 15.105-15.115(2322-2324) BasePeak:93.10(2105)
BG Mode:Calc. from Peak Group 1 - Event 1 Scan

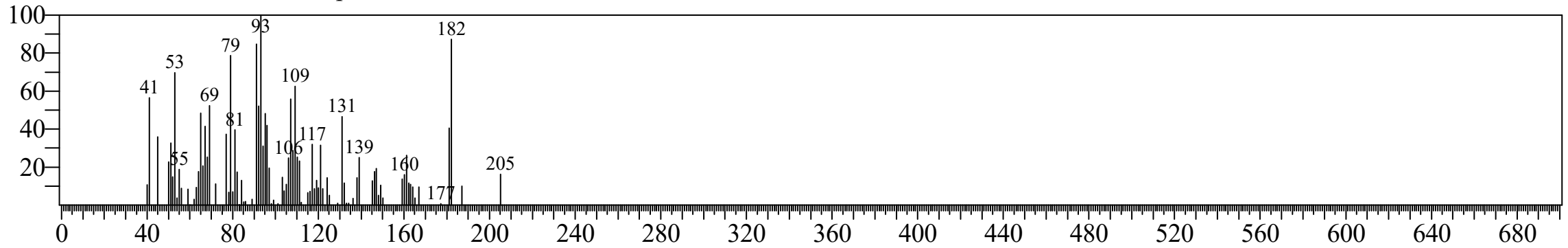

Hit#:1 Entry:147730 Library:NIST14.lib
SI:67 Formula:C21H34O2 CAS:57344-99-7 MolWeight:318 RetIndex:2251
CompName:Androstan-17-one, 3-ethyl-3-hydroxy-, (5.alpha.)- $$ 3-Ethyl-3-hydroxyandrostan-17-one # $$

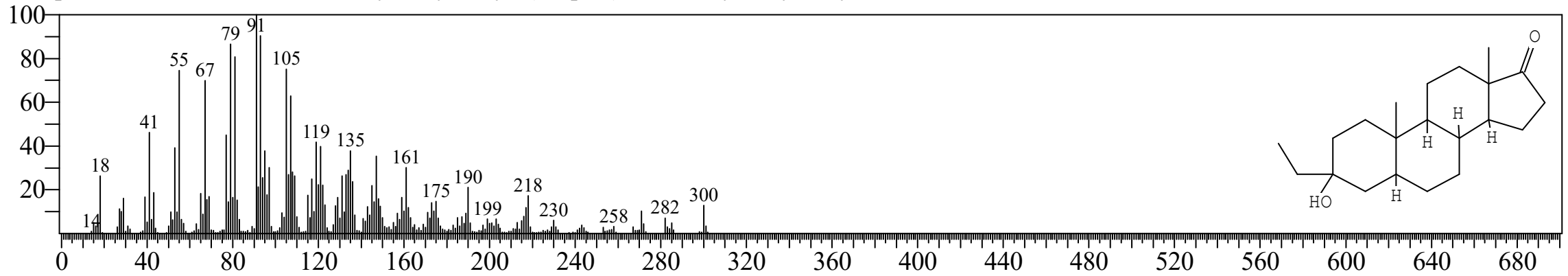

Hit#:2 Entry:88258 Library:WILEY8.LIB
SI:67 Formula:C14H20 CAS:79772-15-9 MolWeight:188 RetIndex:0
CompName:PENTACYCLO[7.5.0.0(2,8).0(5,14).0(7,11)]TETRADECANE

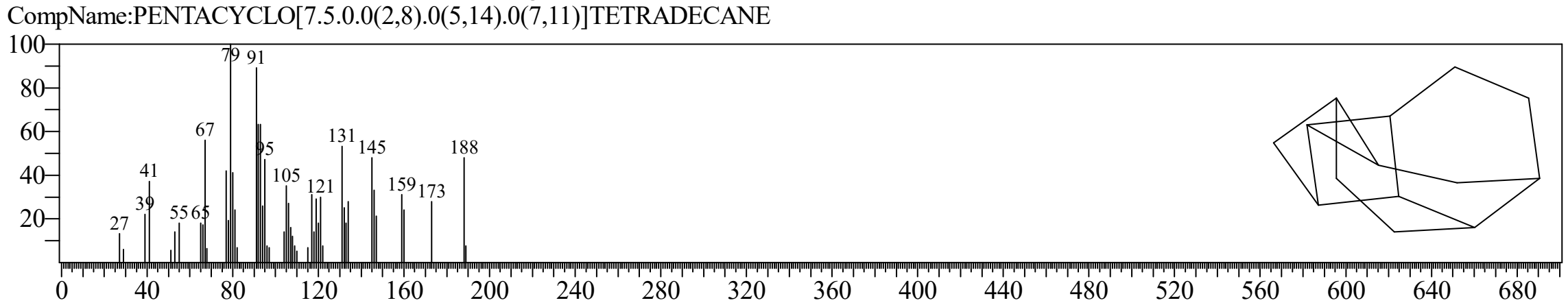

Hit#:3 Entry:38534 Library:NIST14.lib
SI:67 Formula:C14H20 CAS:79772-15-9 MolWeight:188 RetIndex:1164
CompName:Pentacyclo[7.5.0.0(2,8).0(5,14).0(7,11)]tetradecane

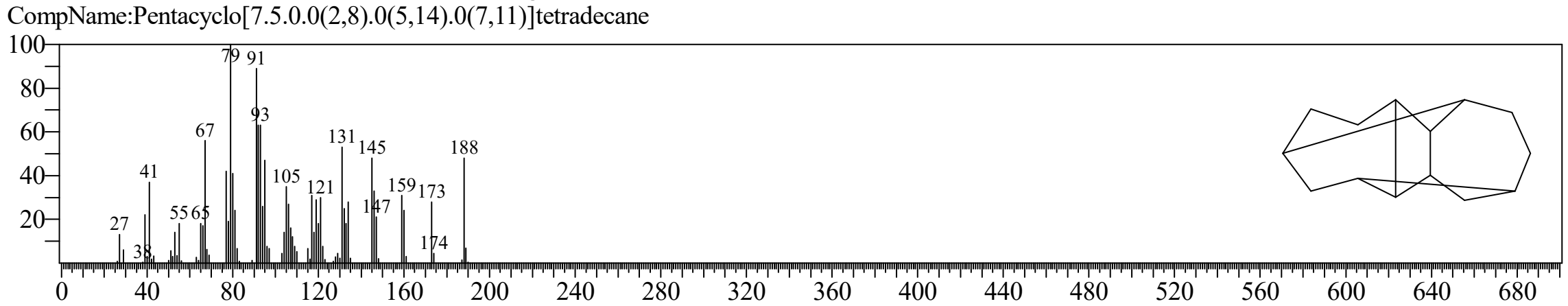

<< Target >>
Line#:96   R.Time:15.155(Scan#:2332) MassPeaks:72
RawMode:Averaged 15.150-15.160(2331-2333) BasePeak:55.00(45701)
BG Mode:Calc. from Peak Group 1 - Event 1 Scan

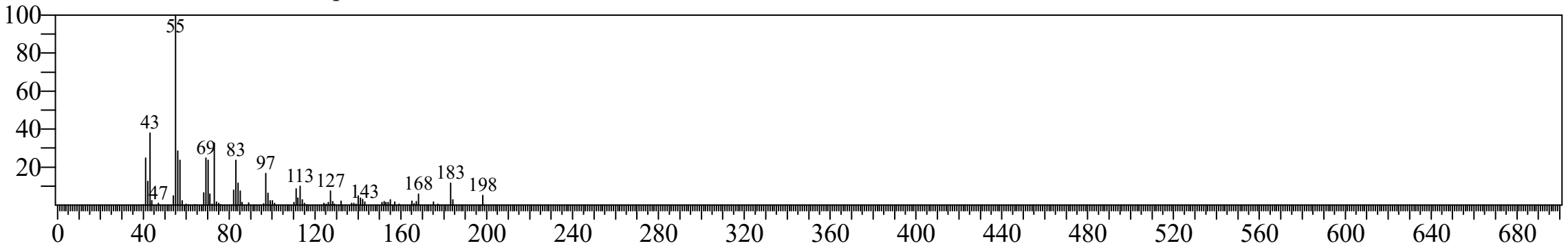

Hit#:1 Entry:23631 Library:NIST14s.lib
SI:88 Formula:C15H28O2 CAS:2156-97-0 MolWeight:240 RetIndex:1670
CompName:Dodecyl acrylate $$ n-Lauryl acrylate $$ 2-Propenoic acid, dodecyl ester $$ n-Dodecyl acrylate $$ Acrylic acid, dodecyl ester $$ Lauryl acrylat

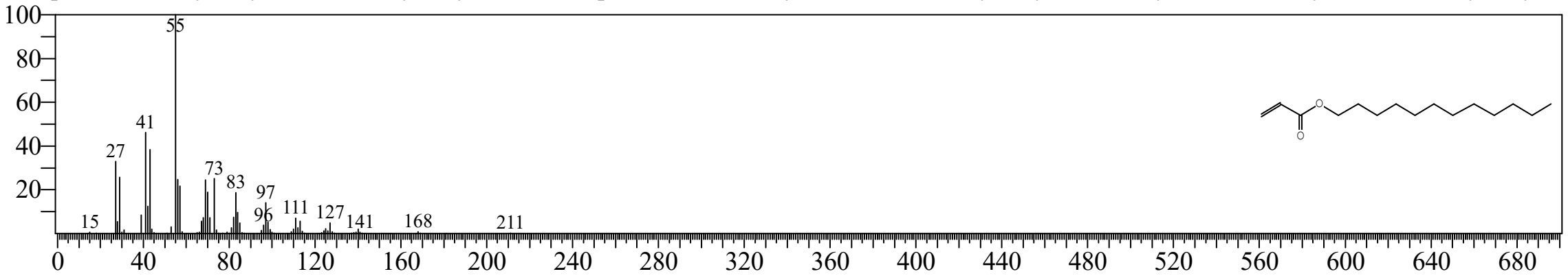

Hit#:2 Entry:161503 Library:WILEY8.LIB
SI:88 Formula:C15H28O2 CAS:2156-97-0 MolWeight:240 RetIndex:0
CompName:2-PROPENOIC ACID, DODECYL ESTER $$ ACRYLIC ACID, DODECYL ESTER $$ 2-PROPENOIC ACID, N-DODECYL ESTER $$ AC

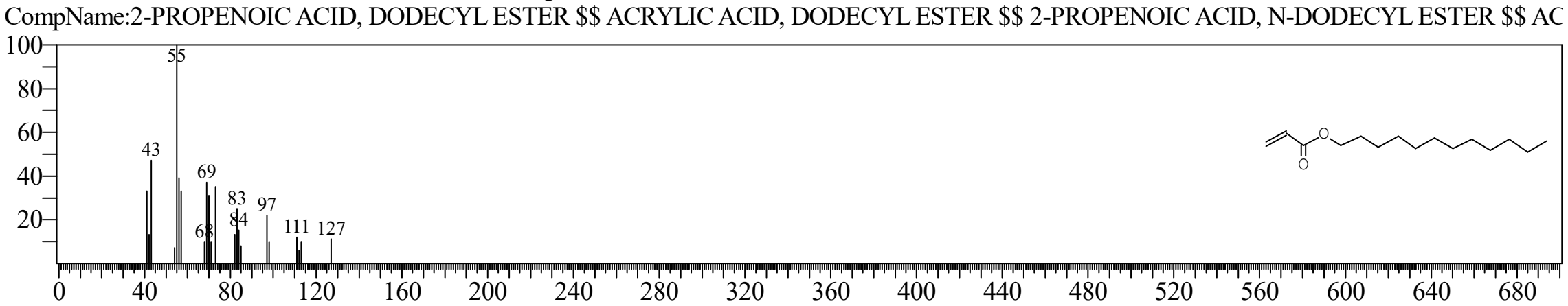

Hit#:3 Entry:23630 Library:NIST14s.lib
SI:87 Formula:C15H28O2 CAS:2156-97-0 MolWeight:240 RetIndex:1670
CompName:Dodecyl acrylate $$ n-Lauryl acrylate $$ 2-Propenoic acid, dodecyl ester $$ n-Dodecyl acrylate $$ Acrylic acid, dodecyl ester $$ Lauryl acrylat

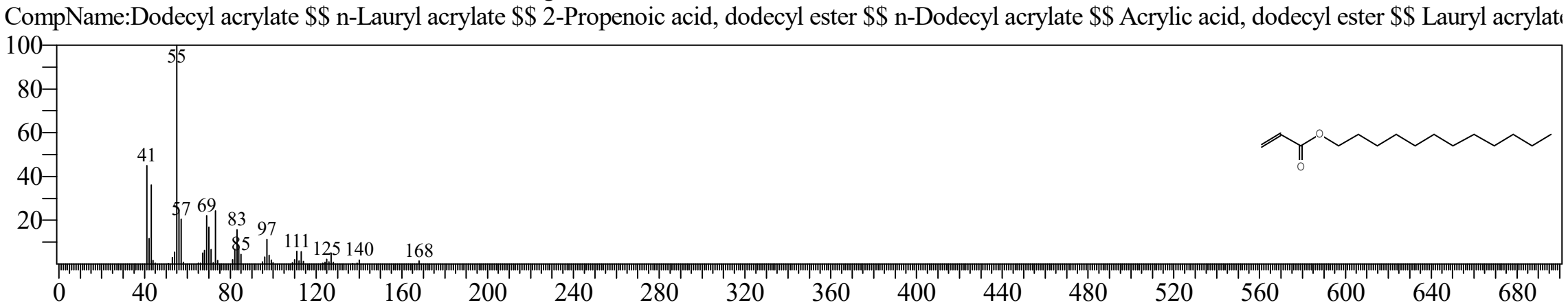

<< Target >>
Line#:97   R.Time:15.260(Scan#:2353) MassPeaks:98
RawMode:Averaged 15.255-15.265(2352-2354) BasePeak:43.00(46884)
BG Mode:Calc. from Peak Group 1 - Event 1 Scan

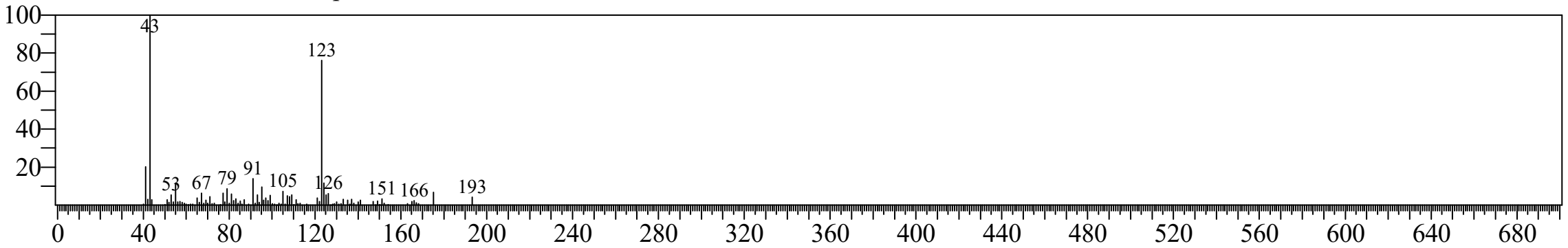

Hit#:1 Entry:96113 Library:WILEY8.LIB
SI:86 Formula:C13H22O CAS:22029-76-1 MolWeight:194 RetIndex:0
CompName:3-BUTEN-2-OL, 4-(2,6,6-TRIMETHYL-1-CYCLOHEXEN-1-YL)- $$ (3E)-4-(2,6,6-TRIMETHYL-1-CYCLOHEXEN-1-YL)-3-BUTEN-2-O

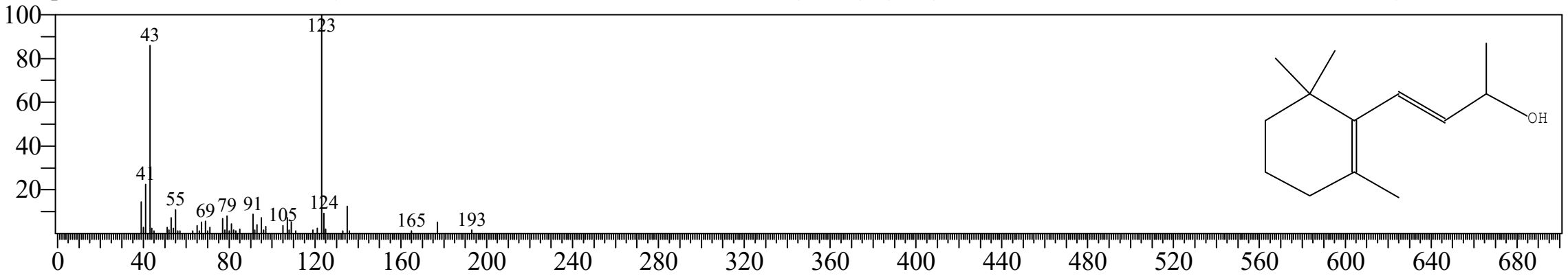

Hit#:2 Entry:75315 Library:NIST14.lib
SI:84 Formula:C14H20O3 CAS:0-00-0 MolWeight:236 RetIndex:1633
CompName:Spirio-10-(2,11-dioxabicyclo[4.4.1]undeca-3,5-diene)-2'-(oxirane), 1,3,7,7-tetramethyl-

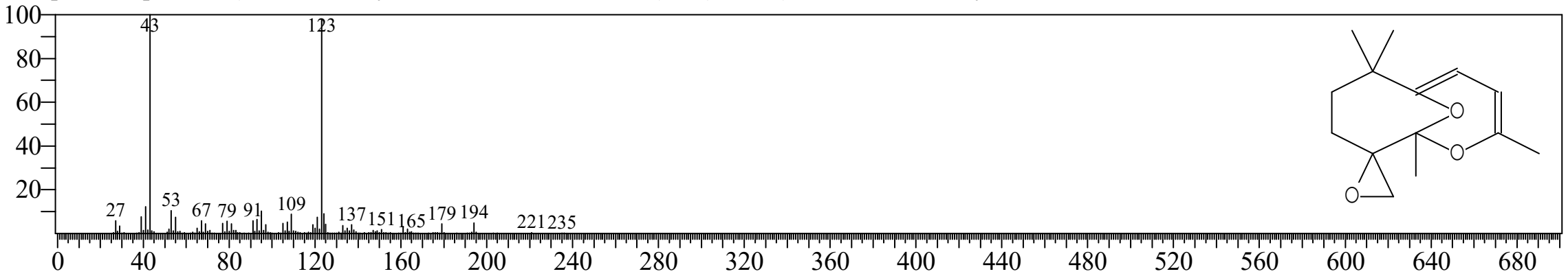

Hit#:3 Entry:155295 Library:WILEY8.LIB
SI:84 Formula:C14H20O3 CAS:0-00-0 MolWeight:236 RetIndex:0
CompName:<NO NAME>

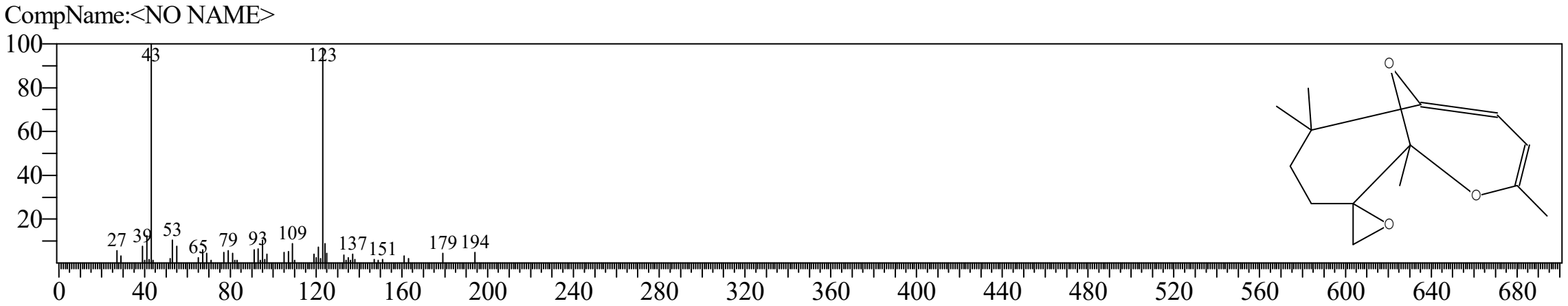

<< Target >>
Line#:98 R.Time:15.430(Scan#:2387) MassPeaks:104
RawMode:Averaged 15.425-15.435(2386-2388) BasePeak:194.10(19650)
BG Mode:Calc. from Peak Group 1 - Event 1 Scan

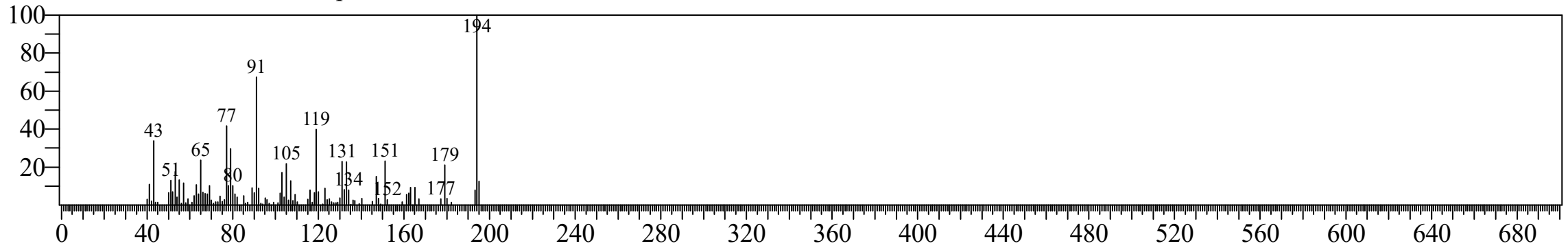

Hit#:1 Entry:95026 Library:WILEY8.LIB
SI:83 Formula:C11H14O3 CAS:6627-88-9 MolWeight:194 RetIndex:0
CompName:PHENOL, 2,6-DIMETHOXY-4-(2-PROPENYL)- $$ 2,6-DIMETHOXY-4-(2-PROPENYL)PHENOL $$ 2,6-DIMETHOXY-4-ALLYLPHENC

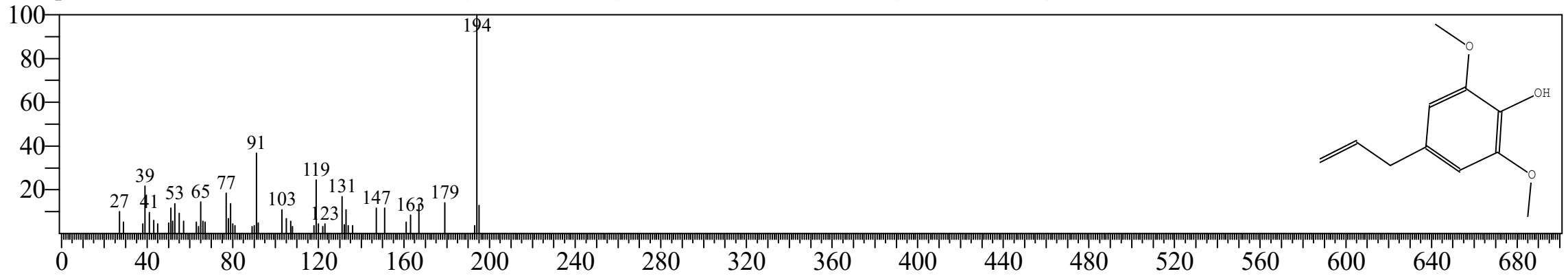

Hit#:2 Entry:42516 Library:NIST14.lib
SI:82 Formula:C11H14O3 CAS:6627-88-9 MolWeight:194 RetIndex:1581
CompName:Phenol, 2,6-dimethoxy-4-(2-propenyl)- $$ Phenol, 4-allyl-2,6-dimethoxy- $$ Methoxyeugenol $$ 2,6-Dimethoxy-4-allylphenol $$ 4-Allyl-2,6-d

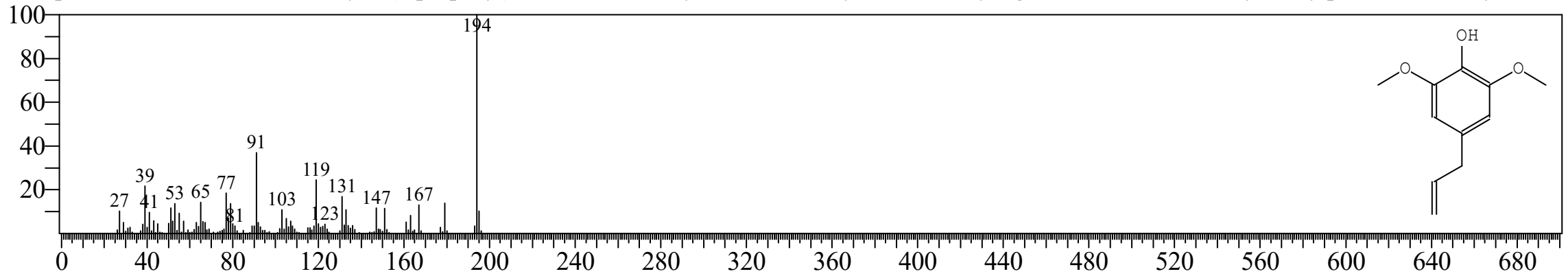

Hit#:3 Entry:95027 Library:WILEY8.LIB
SI:82 Formula:C11H14O3 CAS:6627-88-9 MolWeight:194 RetIndex:0
CompName:PHENOL, 2,6-DIMETHOXY-4-(2-PROPENYL)- $$ 2,6-DIMETHOXY-4-(2-PROPENYL)PHENOL $$ 2,6-DIMETHOXY-4-ALLYLPHENC

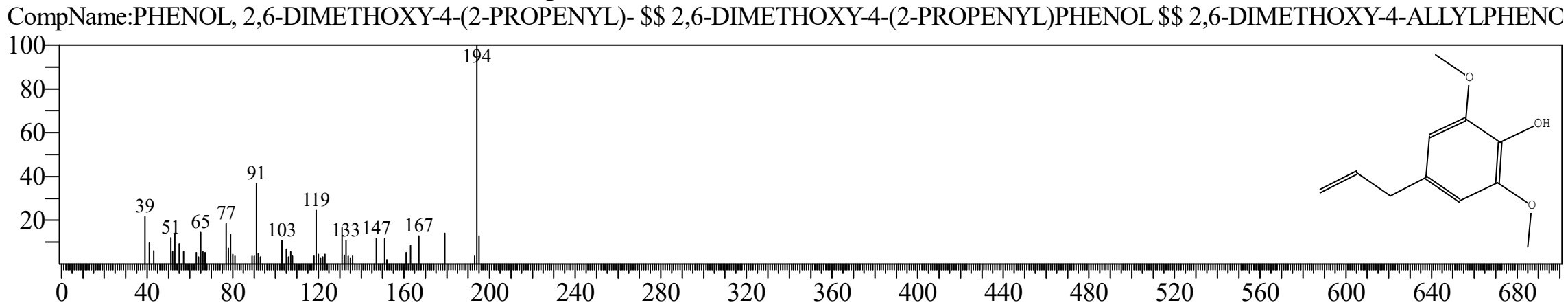

<< Target >>
Line#:99   R.Time:15.485(Scan#:2398) MassPeaks:85
RawMode:Averaged 15.480-15.490(2397-2399) BasePeak:41.05(4384)
BG Mode:Calc. from Peak Group 1 - Event 1 Scan

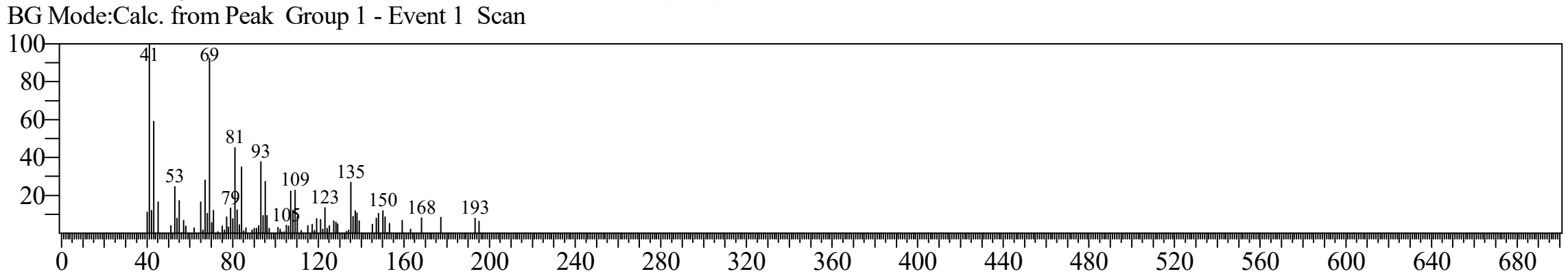

Hit#:1 Entry:277408 Library:WILEY8.LIB
SI:79 Formula:C22H36O2 CAS:0-00-0 MolWeight:332 RetIndex:0
CompName:3,7,11,15-TETRAMETHYL-2,6,10,14-HEXADECATETRAENYL ACETATE $$ ACETIC ACID 3,7,11,15-TETRAMETHYL-HEXADECA-2

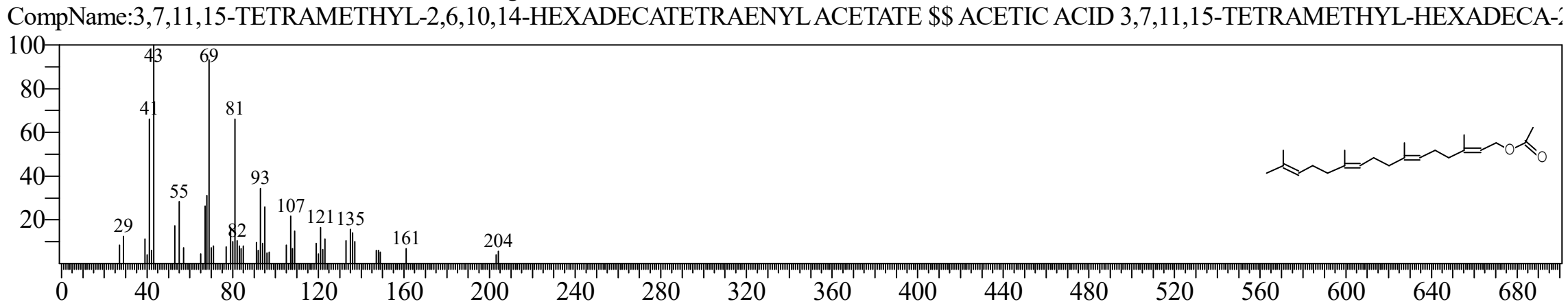

Hit#:2 Entry:30240 Library:NIST14s.lib
SI:79 Formula:C22H36O2 CAS:61691-98-3 MolWeight:332 RetIndex:2316
CompName:2,6,10,14-Hexadecatetraen-1-ol, 3,7,11,15-tetramethyl-, acetate, (E,E,E)- $$ (2E,6E,10E)-3,7,11,15-Tetramethyl-2,6,10,14-hexadecatetraenyl ac

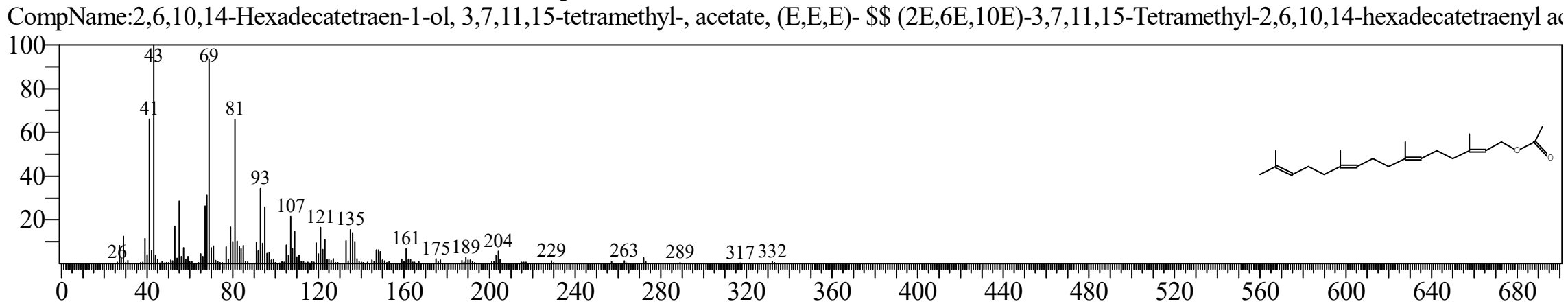

Hit#:3 Entry:135959 Library:WILEY8.LIB
SI:79 Formula:C15H26O CAS:4602-84-0 MolWeight:222 RetIndex:0
CompName:2,6,10-DODECATRIEN-1-OL, 3,7,11-TRIMETHYL- $$ (2E,6E)-3,7,11-TRIMETHYL-2,6,10-DODECATRIEN-1-OL # $$ (2E,6E)-3,7,11-TI

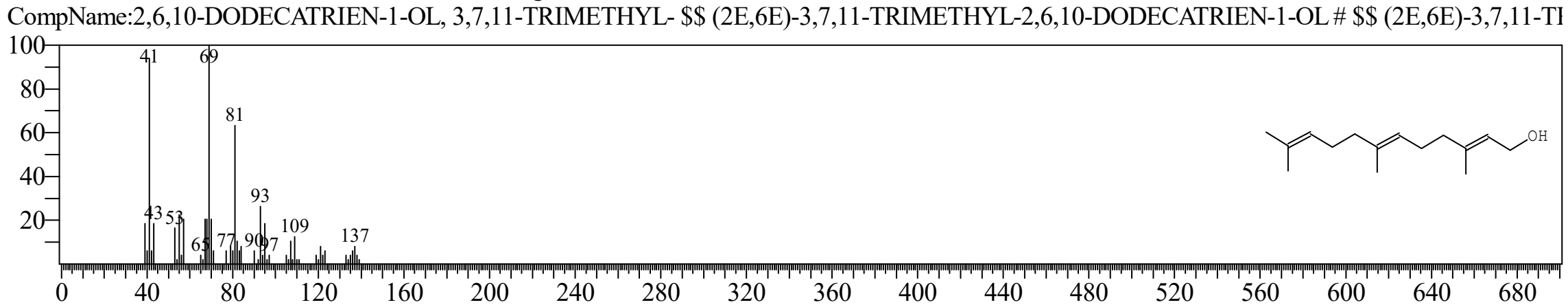

<< Target >>
Line#:100 R.Time:15.550(Scan#:2411) MassPeaks:110
RawMode:Averaged 15.545-15.555(2410-2412) BasePeak:69.05(118674)
BG Mode:Calc. from Peak Group 1 - Event 1 Scan

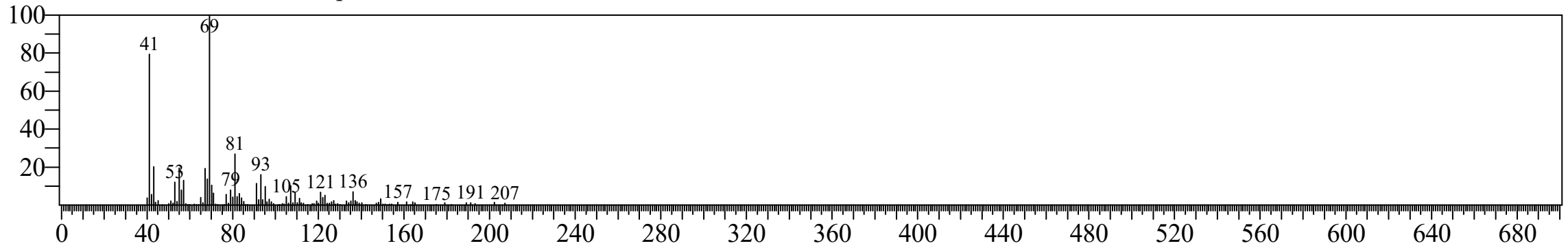

Hit#:1 Entry:64298 Library:NIST14.lib
SI:93 Formula:C15H26O CAS:106-28-5 MolWeight:222 RetIndex:1710
CompName:trans-Farnesol $$ 2,6,10-Dodecatrien-1-ol, 3,7,11-trimethyl-, (E,E)- $$ (E,E)-Farnesol $$ (2E,6E)-Farnesol $$ all-trans-Farnesol $$ trans,trans-

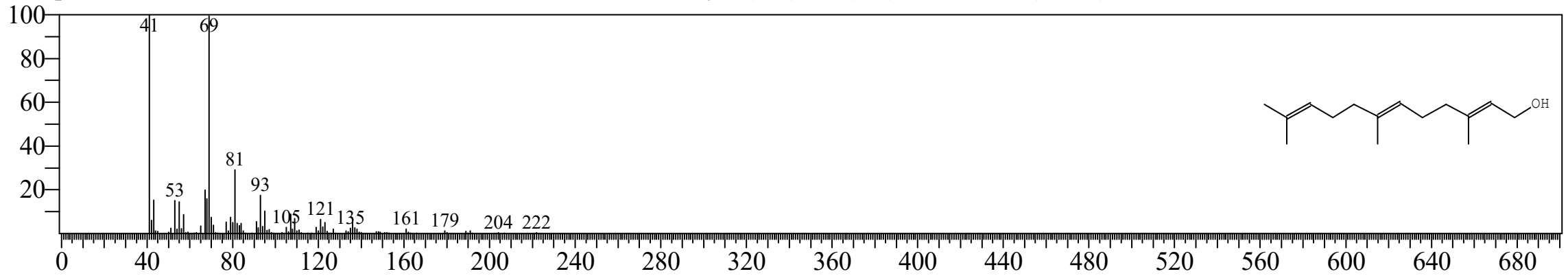

Hit#:2 Entry:135958 Library:WILEY8.LIB
SI:93 Formula:C15H26O CAS:4602-84-0 MolWeight:222 RetIndex:0
CompName:2,6,10-DODECATRIEN-1-OL, 3,7,11-TRIMETHYL- $$ (2E,6E)-3,7,11-TRIMETHYL-2,6,10-DODECATRIEN-1-OL # $$ (2E,6E)-3,7,11-TI

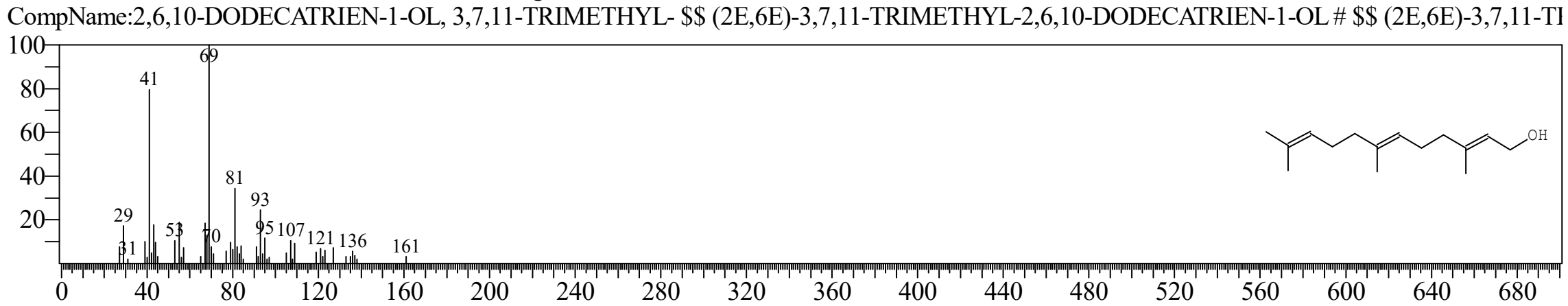

Hit#:3 Entry:21655 Library:NIST14s.lib
SI:93 Formula:C15H26O CAS:4602-84-0 MolWeight:222 RetIndex:1710
CompName:2,6,10-Dodecatrien-1-ol, 3,7,11-trimethyl- $$ Farnesol $$ Farnesyl alcohol $$ 3,7,11-Trimethyl-2,6,10-dodecatrien-1-ol $$ 3,7,11-Trimethyl-2,

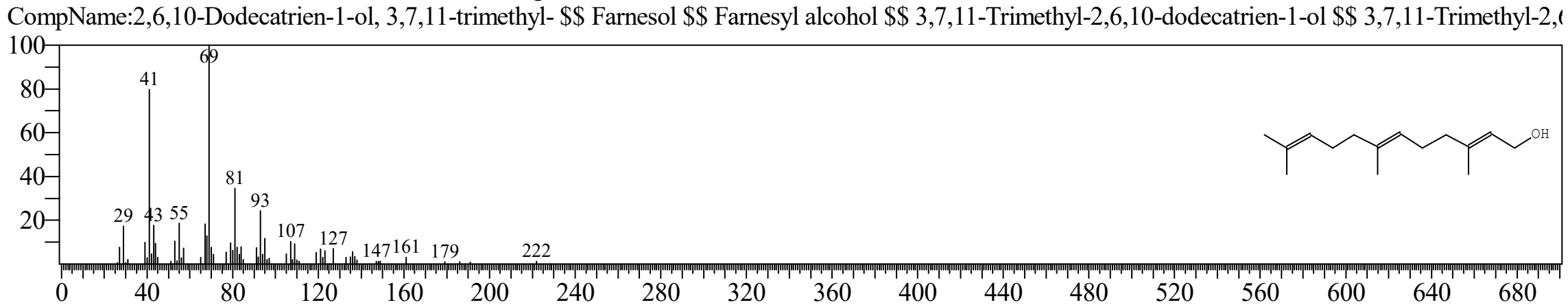

<< Target >>
Line#:101   R.Time:15.675(Scan#:2436) MassPeaks:111
RawMode:Averaged 15.670-15.680(2435-2437) BasePeak:43.00(22931)
BG Mode:Calc. from Peak Group 1 - Event 1 Scan

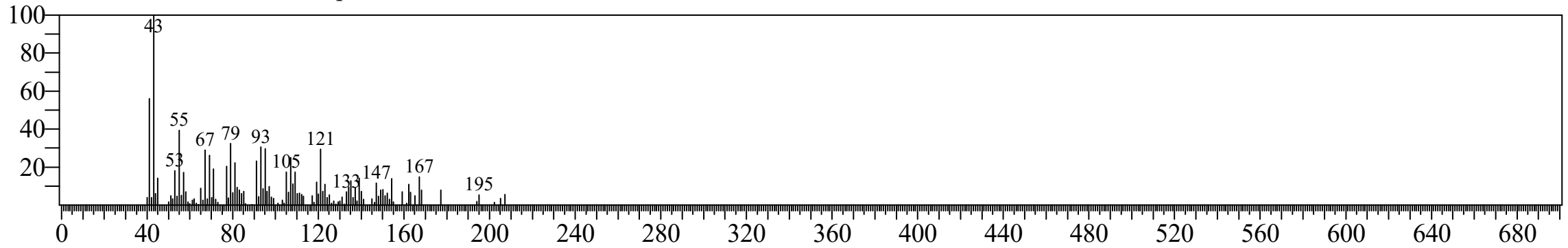

Hit#:1 Entry:21634 Library:NIST14s.lib
SI:82 Formula:C15H26O CAS:552-02-3 MolWeight:222 RetIndex:1530
CompName:1H-Cycloprop[e]azulen-4-ol, decahydro-1,1,4,7-tetramethyl-, [1aR-(1a.alpha.,4.beta.,4a.beta.,7.alpha.,7a.beta.,7b.alpha.)]- $$ Viridiflorol $$ 1,1

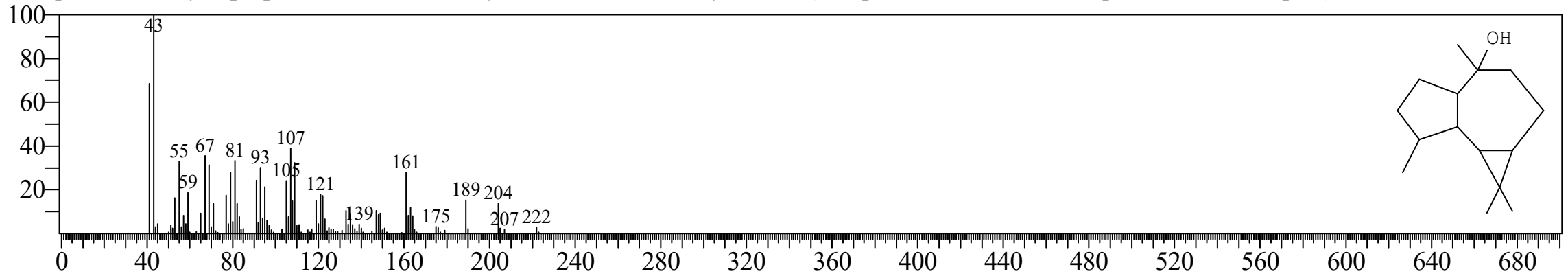

Hit#:2 Entry:122493 Library:NIST14.lib
SI:82 Formula:C20H34O CAS:25269-17-4 MolWeight:290 RetIndex:2211
CompName:Thunbergol $$ (1R,2E,4S,7E,11E)-4-Isopropyl-1,7,11-trimethylcyclotetradeca-2,7,11-trienol $$ Isocembrol $$ 4-Isopropyl-1,7,11-trimethyl-2,7

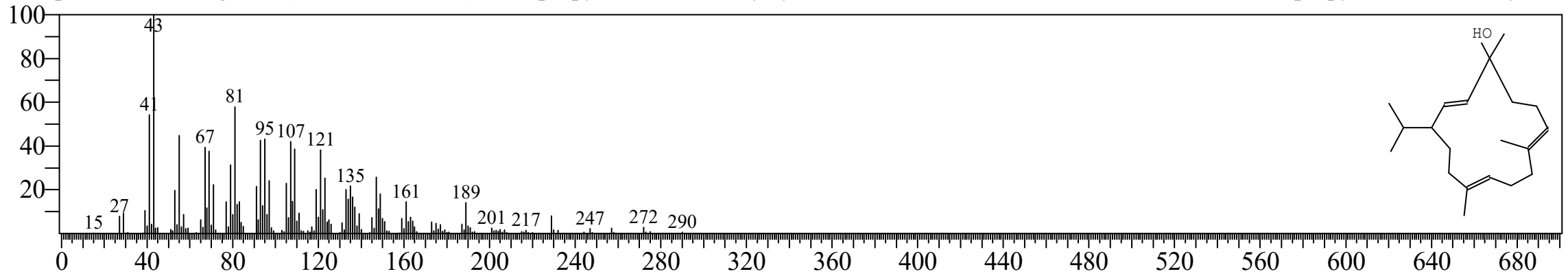

Hit#:3 Entry:62425 Library:NIST14.lib
SI:81 Formula:C15H24O CAS:0-00-0 MolWeight:220 RetIndex:1531
CompName:cis-Z-.alpha.-Bisabolene epoxide $$ 4-[(1Z)-1,5-Dimethyl-1,4-hexadienyl]-1-methyl-7-oxabicyclo[4.1.0]heptane # $$

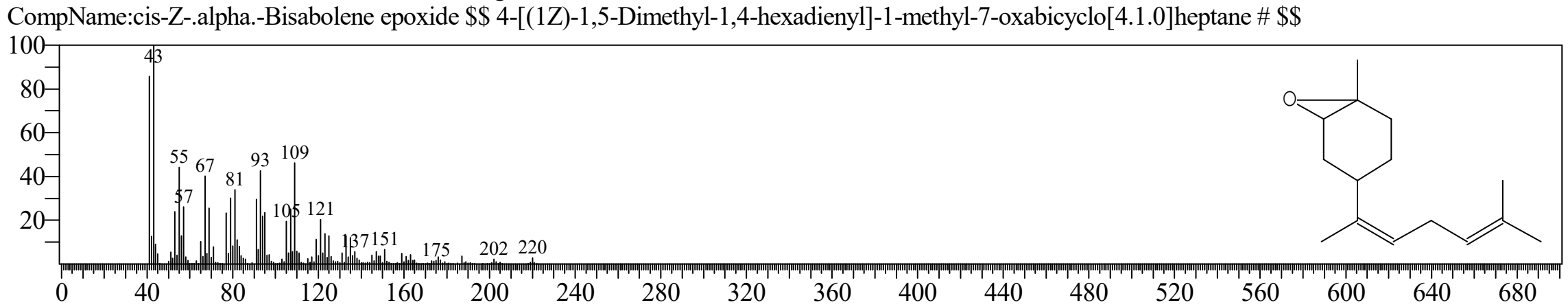

<< Target >>
Line#:102 R.Time:15.790(Scan#:2459) MassPeaks:76
RawMode:Averaged 15.785-15.795(2458-2460) BasePeak:69.05(19855)
BG Mode:Calc. from Peak Group 1 - Event 1 Scan

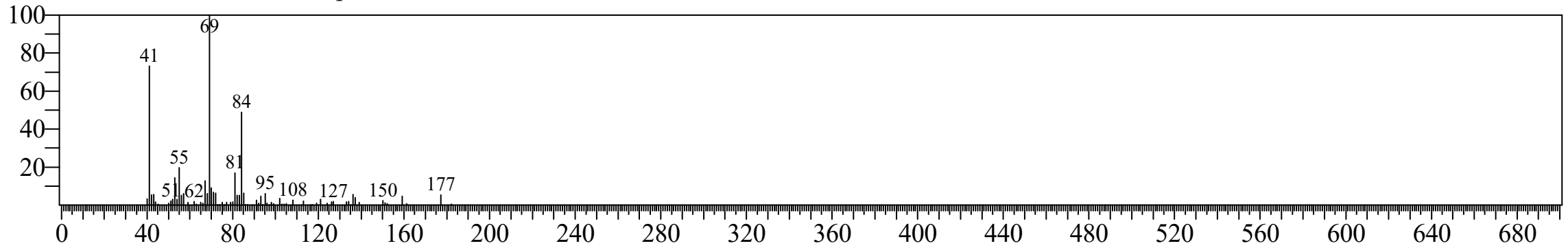

Hit#:1 Entry:21340 Library:NIST14s.lib
SI:88 Formula:C15H24O CAS:502-67-0 MolWeight:220 RetIndex:1656
CompName:2,6,10-Dodecatrienal, 3,7,11-trimethyl-, (E,E)- $$ (E,E)-3,7,11-Trimethyl-2,6,10-dodecatrienal $$ trans,trans-Farnesal $$ trans,trans-2,6-Farnes

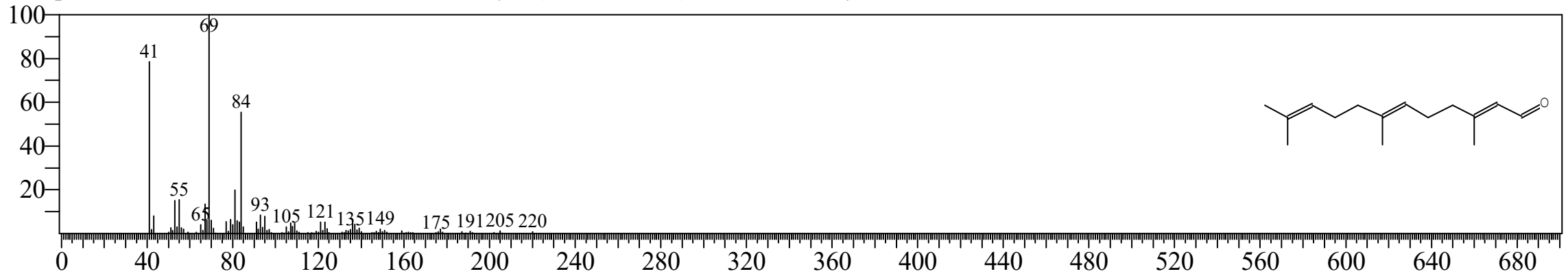

Hit#:2 Entry:62435 Library:NIST14.lib
SI:86 Formula:C15H24O CAS:502-67-0 MolWeight:220 RetIndex:1656
CompName:2,6,10-Dodecatrienal, 3,7,11-trimethyl-, (E,E)- $$ (E,E)-3,7,11-Trimethyl-2,6,10-dodecatrienal $$ trans,trans-Farnesal $$ trans,trans-2,6-Farnes

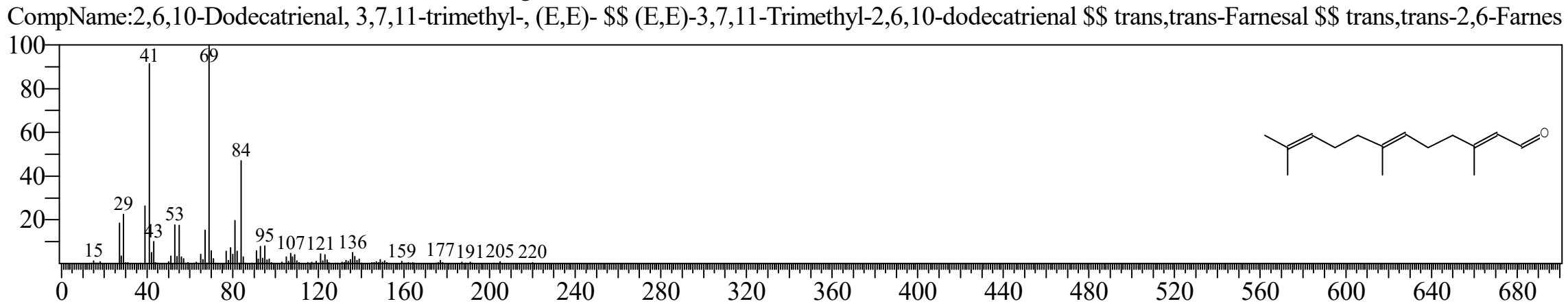

Hit#:3 Entry:133047 Library:WILEY8.LIB
SI:85 Formula:C15H24O CAS:19317-11-4 MolWeight:220 RetIndex:0
CompName:2,6,10-DODECATRIENAL, 3,7,11-TRIMETHYL- $$ FARNESAL $$ (2E,6E)-3,7,11-TRIMETHYL-2,6,10-DODECATRIENAL # $$ (2E,6E)

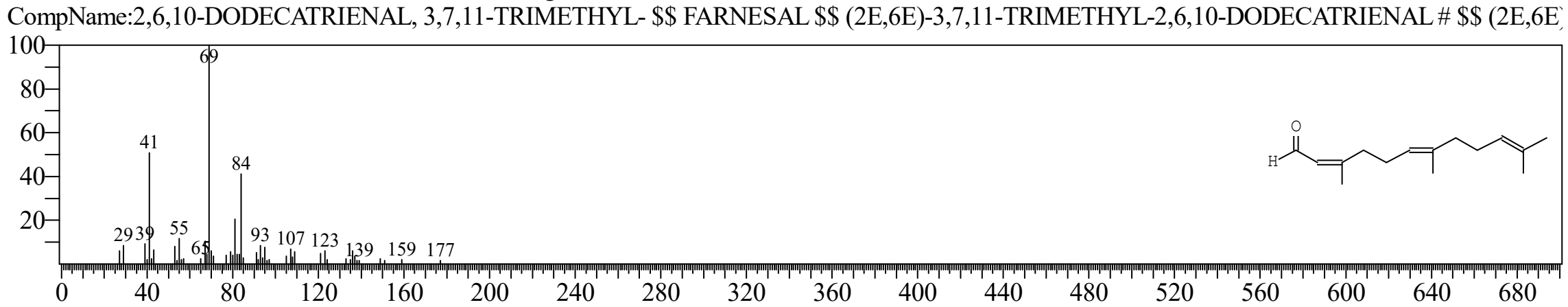

<< Target >>
Line#:103 R.Time:15.840(Scan#:2469) MassPeaks:82
RawMode:Averaged 15.835-15.845(2468-2470) BasePeak:43.05(4739)
BG Mode:Calc. from Peak Group 1 - Event 1 Scan

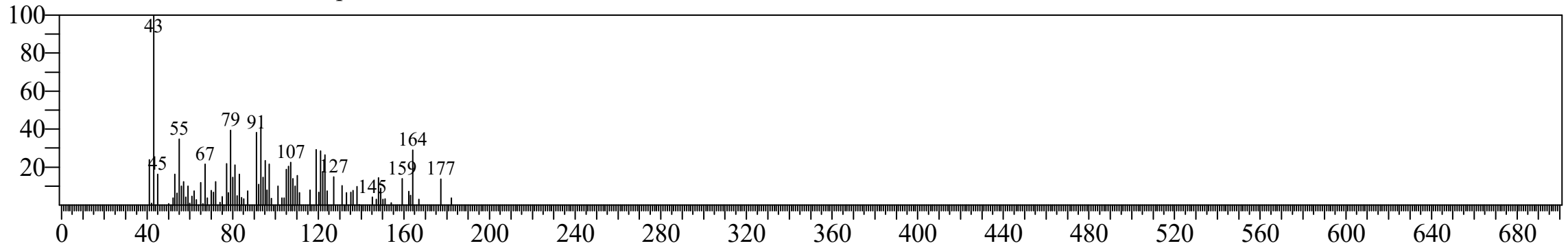

Hit#:1 Entry:132802 Library:WILEY8.LIB
SI:77 Formula:C15H24O CAS:1139-30-6 MolWeight:220 RetIndex:0
CompName:(-)-5-OXATRICYCLO[8.2.0.0(4,6)]DODECANE,,12-TRIMETHYL-9-METHYLENE-, [1R-(1R*,4R*,6R*,10S*)]- $$ 5-OXATRICYCLO[8.2

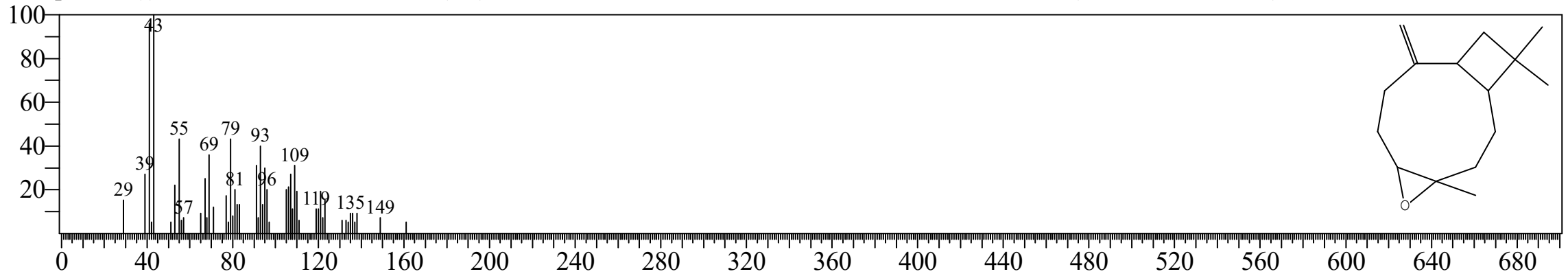

Hit#:2 Entry:132803 Library:WILEY8.LIB
SI:74 Formula:C15H24O CAS:1139-30-6 MolWeight:220 RetIndex:0
CompName:(-)-5-OXATRICYCLO[8.2.0.0(4,6)]DODECANE,,12-TRIMETHYL-9-METHYLENE-, [1R-(1R*,4R*,6R*,10S*)]- $$ 5-OXATRICYCLO[8.2

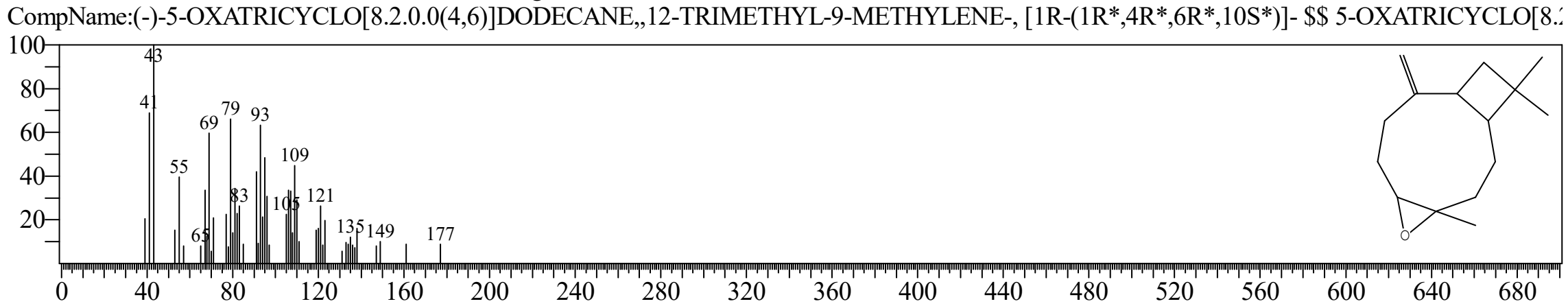

Hit#:3 Entry:21335 Library:NIST14s.lib
SI:74 Formula:C15H24O CAS:1139-30-6 MolWeight:220 RetIndex:1507
CompName:Caryophyllene oxide $$ 5-Oxatricyclo[8.2.0.0(4,6)-]dodecane, 4,12,12-trimethyl-9-methylene-, [1R-(1R*,4R*,6R*,10S*)]- $$ 5-Oxatricyclo(8.

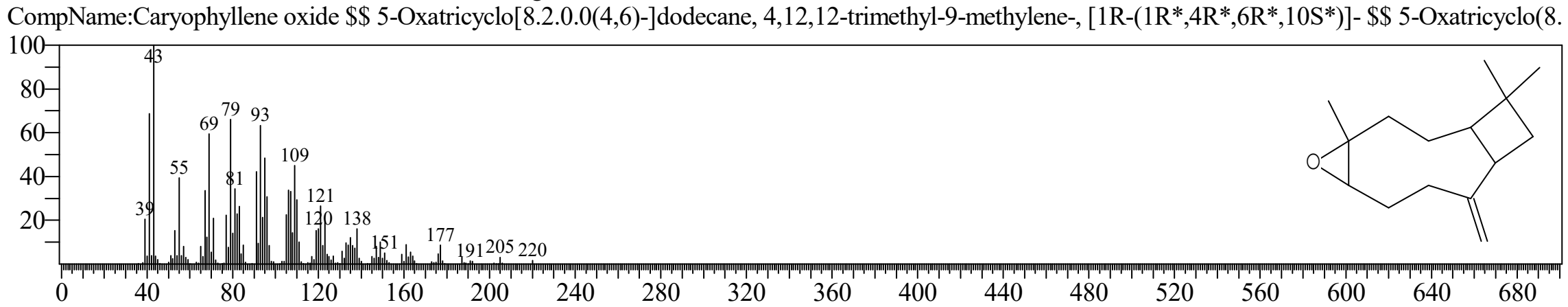

<< Target >>
Line#:104 R.Time:15.900(Scan#:2481) MassPeaks:98
RawMode:Averaged 15.895-15.905(2480-2482) BasePeak:43.00(17633)
BG Mode:Calc. from Peak Group 1 - Event 1 Scan

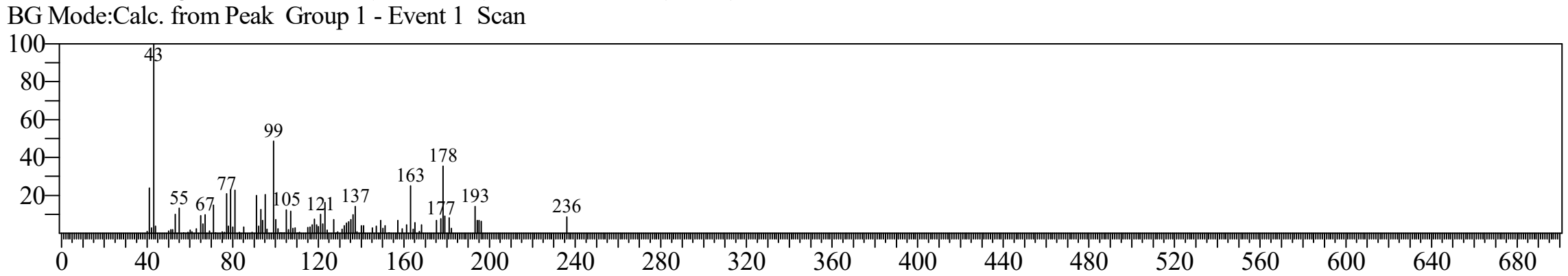

Hit#:1 Entry:75192 Library:WILEY8.LIB
SI:70 Formula:C12H18O CAS:65133-79-1 MolWeight:178 RetIndex:0
CompName:3-BUTEN-2-ONE, 4-(6,6-DIMETHYL-1-CYCLOHEXEN-1-YL)- $$ (3E)-4-(6,6-DIMETHYL-1-CYCLOHEXEN-1-YL)-3-BUTEN-2-ONE #

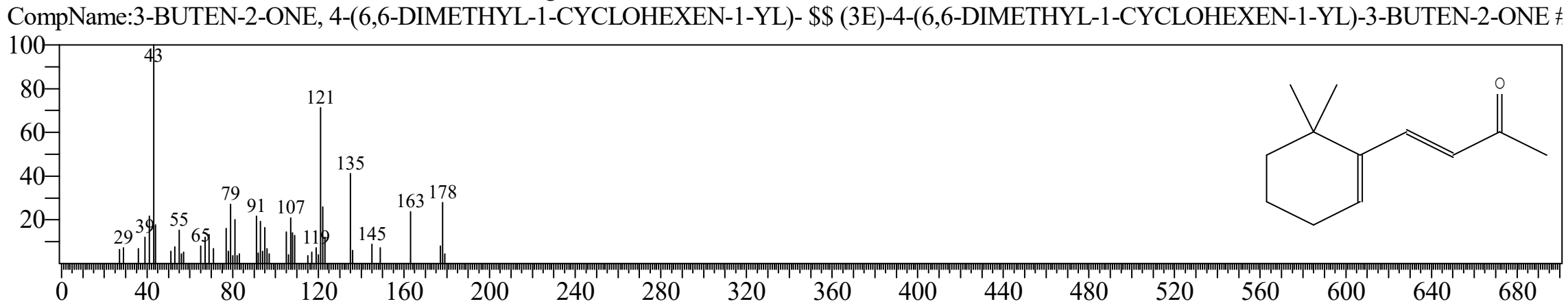

Hit#:2 Entry:31925 Library:NIST14.lib
SI:70 Formula:C12H18O CAS:65133-79-1 MolWeight:178 RetIndex:1368
CompName:3-Buten-2-one, 4-(6,6-dimethyl-1-cyclohexen-1-yl)- $$ (3E)-4-(6,6-Dimethyl-1-cyclohexen-1-yl)-3-buten-2-one # $$

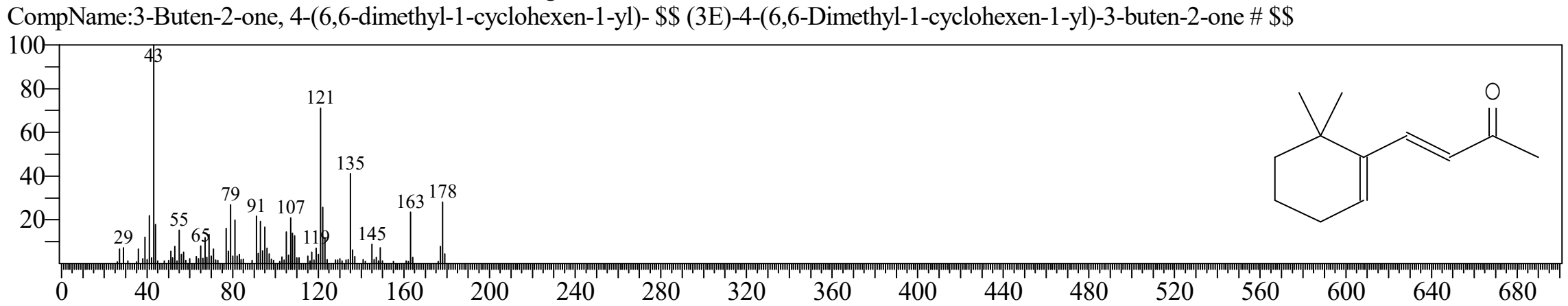

Hit#:3 Entry:180994 Library:NIST14.lib
SI:68 Formula:C23H34O3 CAS:0-00-0 MolWeight:358 RetIndex:2570
CompName:Pregn-4-ene-1,20-dione, 12-hydroxy-16,17-dimethyl- $$ 12-Hydroxy-16,17-dimethylpregn-4-ene-1,20-dione # $$

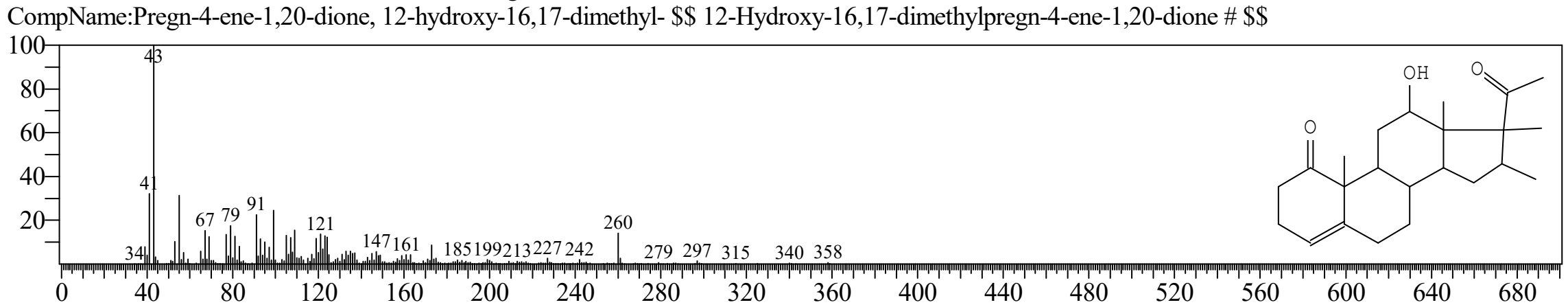

<< Target >>
Line#:105   R.Time:15.965(Scan#:2494) MassPeaks:131
RawMode:Averaged 15.960-15.970(2493-2495) BasePeak:43.05(54362)
BG Mode:Calc. from Peak Group 1 - Event 1 Scan

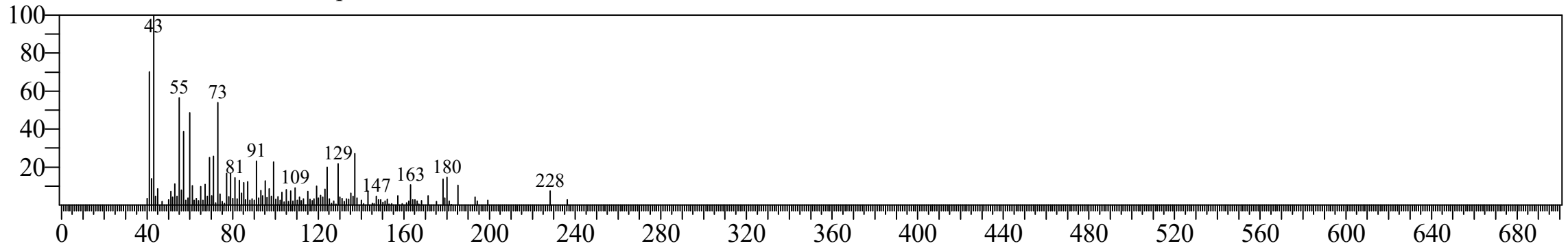

Hit#:1 Entry:220217 Library:WILEY8.LIB
SI:75 Formula:C18H36O2 CAS:57-11-4 MolWeight:284 RetIndex:0
CompName:OCTADECANOIC ACID $$ STEARATE $$ STEARIC ACID $$ 1-HEPTADECANECARBOXYLIC ACID $$ 1-HEPTADECANECARBOX

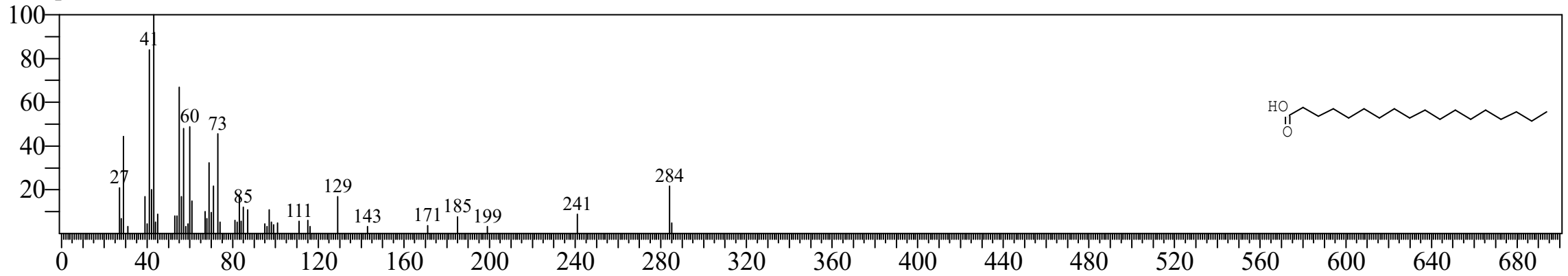

Hit#:2 Entry:144609 Library:WILEY8.LIB
SI:75 Formula:C14H28O2 CAS:544-63-8 MolWeight:228 RetIndex:0
CompName:TETRADECANOIC ACID $$ METHYL TRIDECANOATE $$ MYRISTIC ACID $$ TETRADECANOATE $$ 1-TRIDECANECARBOXYI

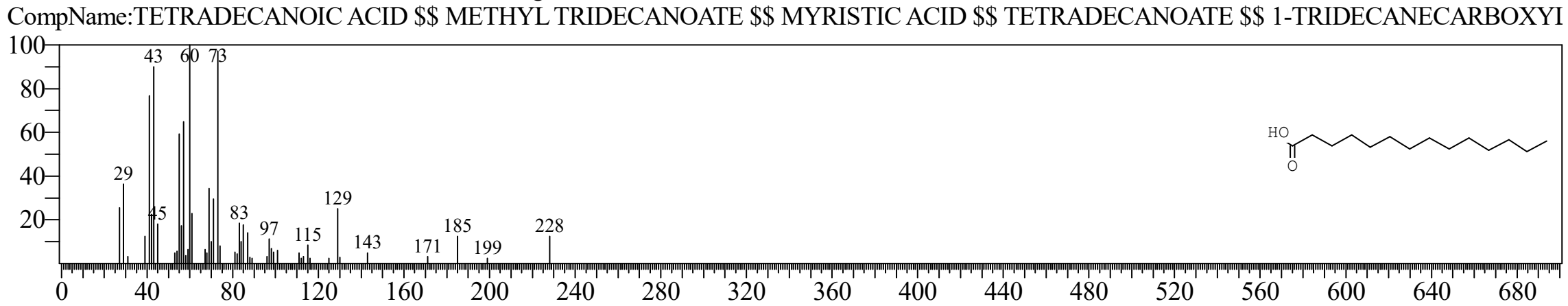

Hit#:3 Entry:144703 Library:WILEY8.LIB
SI:75 Formula:C14H28O2 CAS:544-63-8 MolWeight:228 RetIndex:0
CompName:TETRADECANOIC ACID $$ METHYL TRIDECANOATE $$ MYRISTIC ACID $$ TETRADECANOATE $$ 1-TRIDECANECARBOXYI

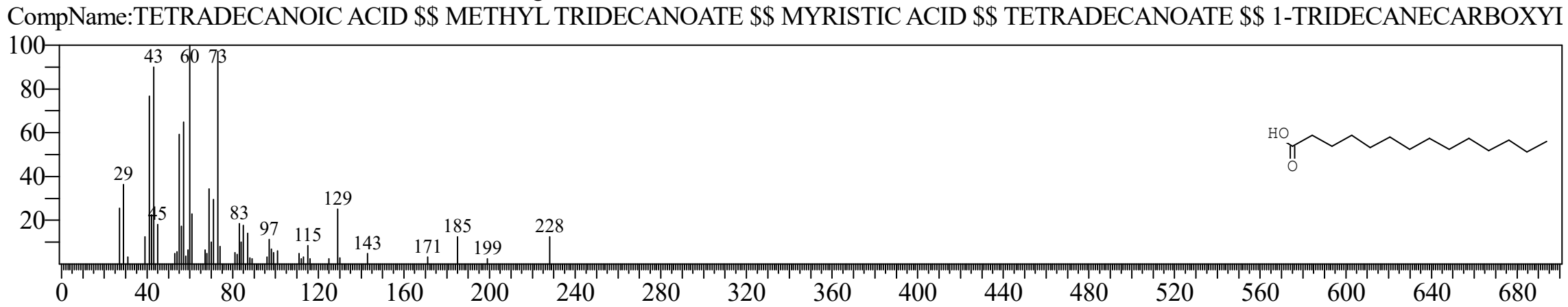

<< Target >>
Line#:106   R.Time:16.095(Scan#:2520) MassPeaks:112
RawMode:Averaged 16.090-16.100(2519-2521) BasePeak:43.05(41368)
BG Mode:Calc. from Peak Group 1 - Event 1 Scan

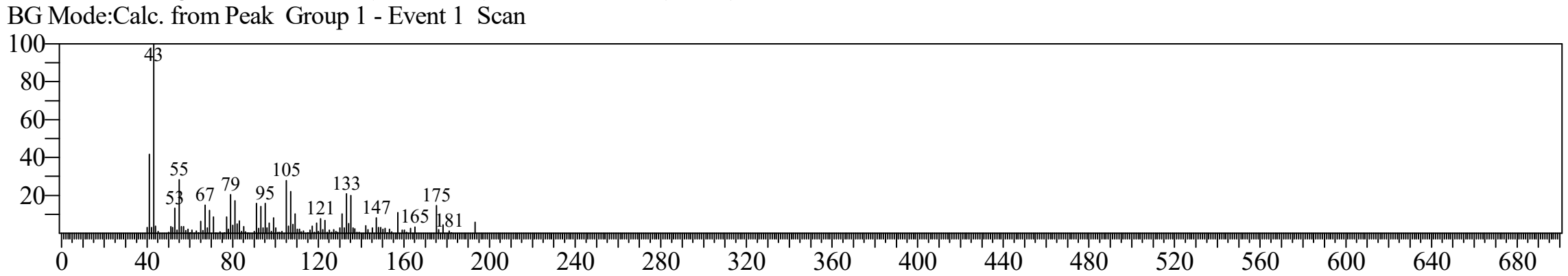

Hit#:1 Entry:168888 Library:NIST14.lib
SI:79 Formula:C23H34O2 CAS:0-00-0 MolWeight:342 RetIndex:2382
CompName:Pregn-4-ene-1,20-dione, 16,17-dimethyl- $$ 16,17-Dimethylpregn-4-ene-1,20-dione # $$

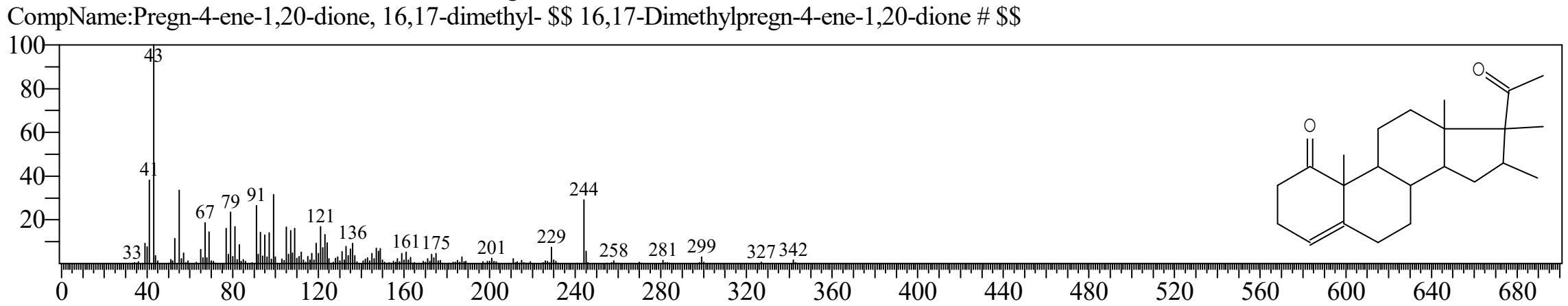

Hit#:2 Entry:136158 Library:WILEY8.LIB
SI:79 Formula:C15H26O CAS:489-41-8 MolWeight:222 RetIndex:0
CompName:1,1,4,7-TETRAMETHYLDECAHYDRO-1H-CYCLOPROPA[E]AZULEN-4-OL # $$ (-)-GLOBULOL $$ (1AR-(1AALPHA,4ALPHA,4AAL

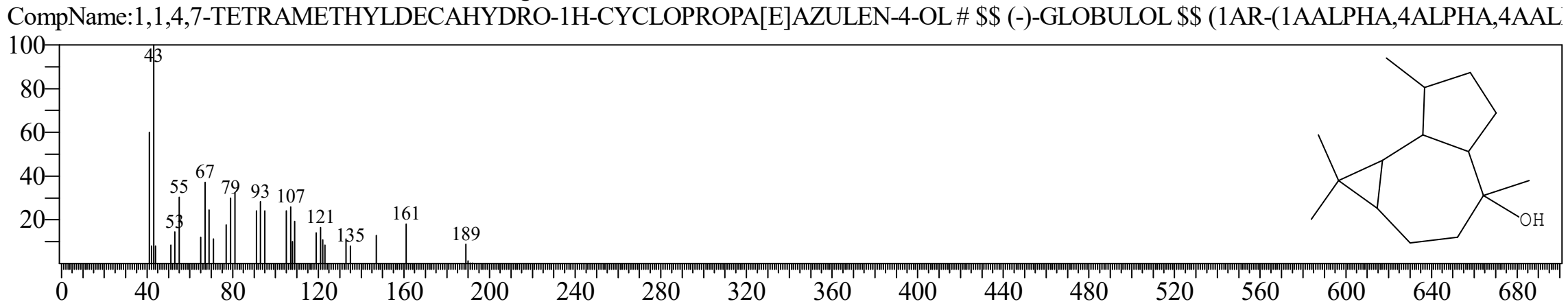

Hit#:3 Entry:287456 Library:WILEY8.LIB
SI:79 Formula:C23H34O2 CAS:0-00-0 MolWeight:342 RetIndex:0
CompName:16,17-DIMETHYLPREGN-4-ENE-1,20-DIONE $$ PREGN-4-ENE-1,20-DIONE, 16,17-DIMETHYL-

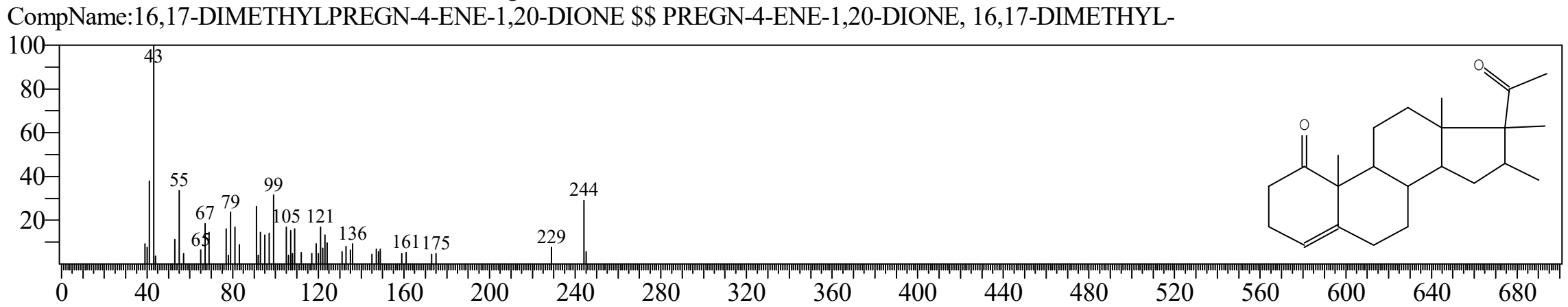

<< Target >>
Line#:107   R.Time:16.215(Scan#:2544) MassPeaks:94
RawMode:Averaged 16.210-16.220(2543-2545) BasePeak:55.05(258865)
BG Mode:Calc. from Peak Group 1 - Event 1 Scan

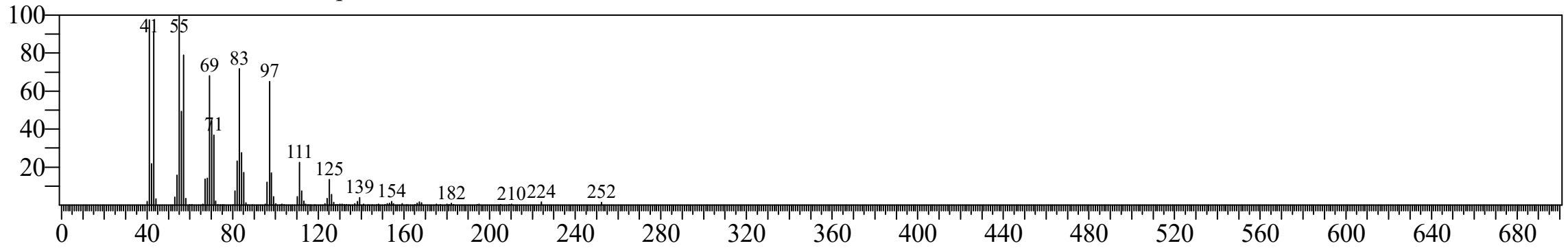

Hit#:1 Entry:89019 Library:NIST14.lib
SI:97 Formula:C17H32O CAS:0-00-0 MolWeight:252 RetIndex:1907
CompName:E-15-Heptadecenal $$ (15E)-15-Heptadecenal # $$

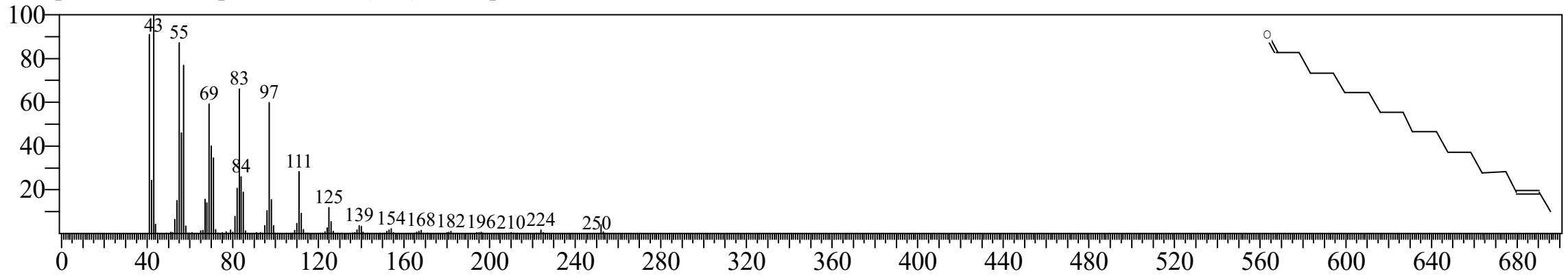

Hit#:2 Entry:77344 Library:NIST14.lib
SI:97 Formula:C16H30O CAS:330207-53-9 MolWeight:238 RetIndex:1808
CompName:E-14-Hexadecenal $$ (14E)-14-Hexadecenal $$ 14-Hexadecenal, (E)- $$

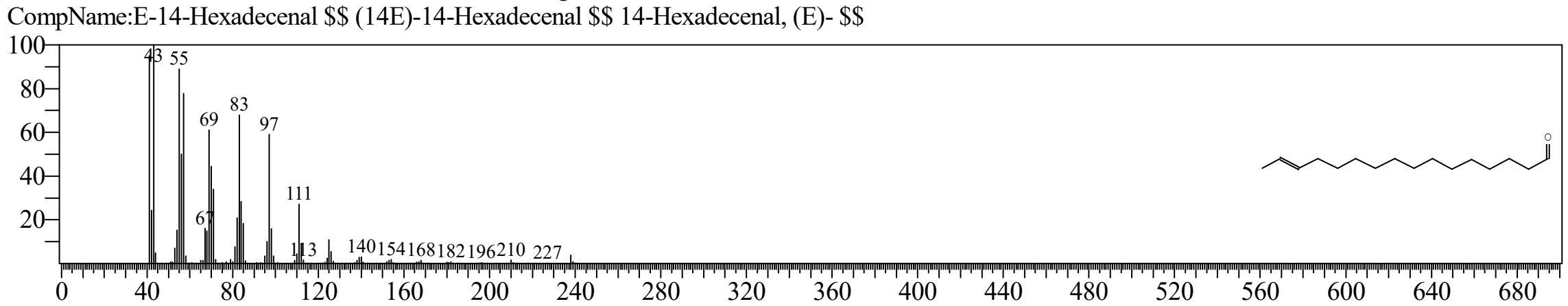

Hit#:3 Entry:25913 Library:NIST14s.lib
SI:96 Formula:C19H38 CAS:18435-45-5 MolWeight:266 RetIndex:1900
CompName:1-Nonadecene $$ Nonadec-1-ene $$

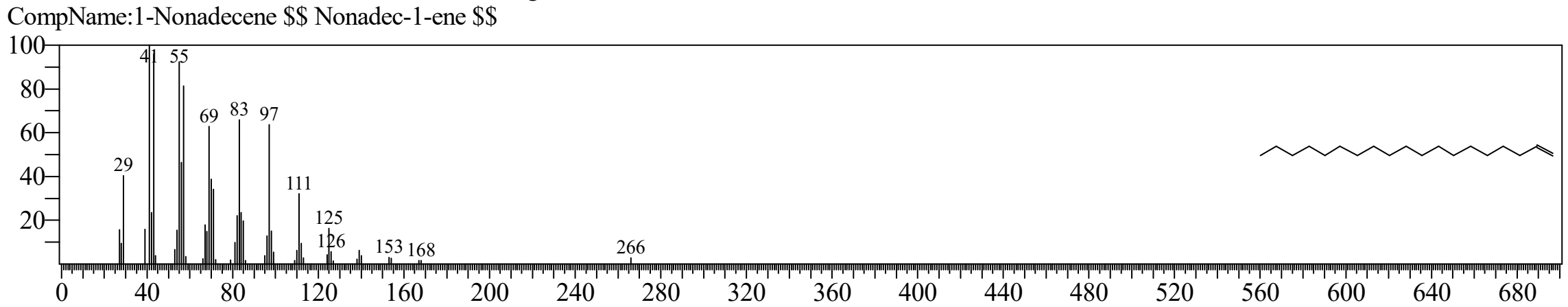

<< Target >>
Line#:108   R.Time:16.270(Scan#:2555) MassPeaks:108
RawMode:Averaged 16.265-16.275(2554-2556) BasePeak:43.05(51797)
BG Mode:Calc. from Peak Group 1 - Event 1 Scan

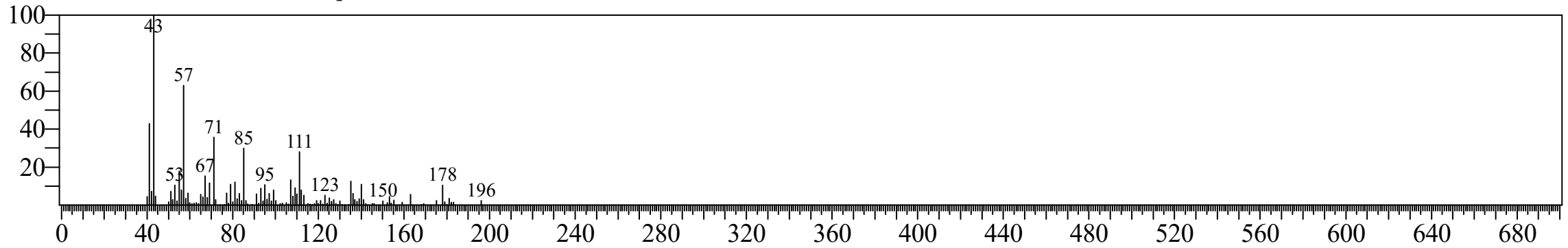

Hit#:1 Entry:98128 Library:WILEY8.LIB
SI:81 Formula:C11H16O3 CAS:5989-02-6 MolWeight:196 RetIndex:0
CompName:2(4H)-BENZOFURANONE, 5,6,7,7A-TETRAHYDRO-6-HYDROXY-4,4,7A-TRIMETHYL-, (6S-CIS)- $$ (-)-LOLIOLIDE $$ (-)LOLIOLID

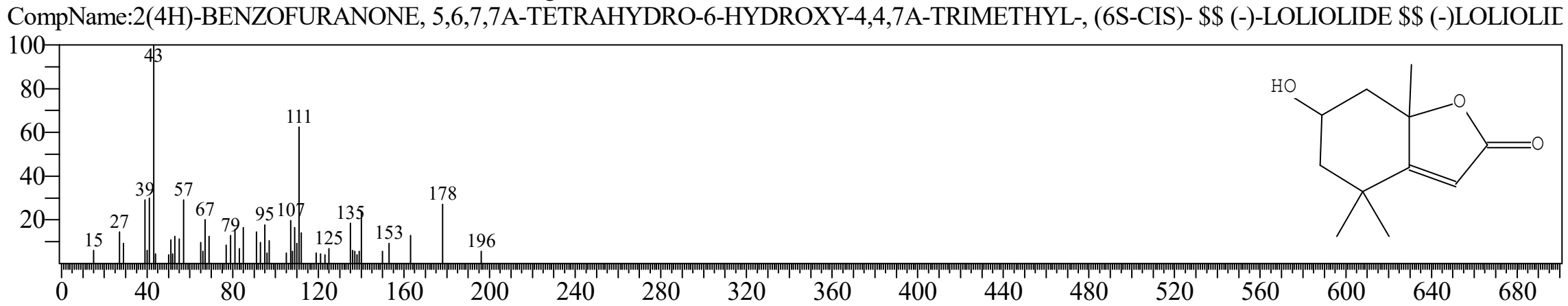

Hit#:2 Entry:159034 Library:WILEY8.LIB
SI:78 Formula:C16H30O CAS:0-00-0 MolWeight:238 RetIndex:0
CompName:1-(2-METHYLENE-3-PENTYLCYCLOPROPYL)-1-HEPTANOL $$ CYCLOPROPAN, 1-(1-HYDROXY-1-HEPTYL)-2-METHYLEN-3-PE

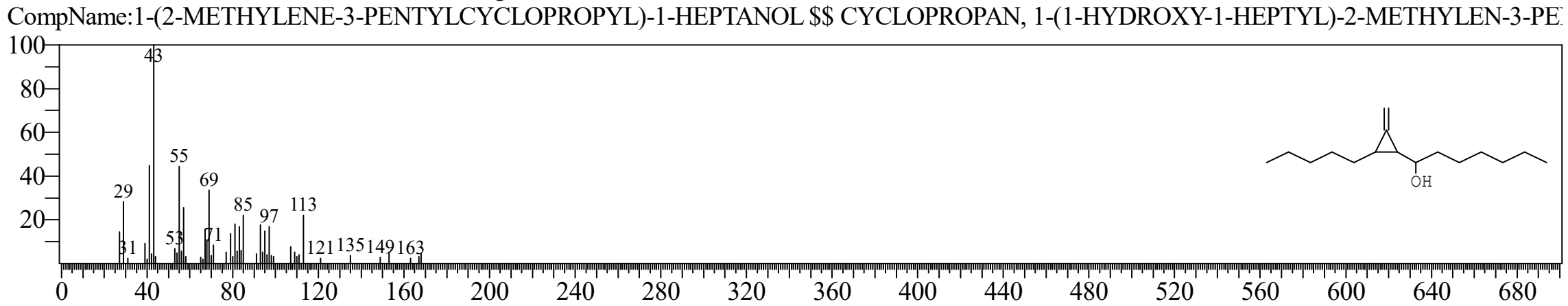

Hit#:3 Entry:77343 Library:NIST14.lib
SI:78 Formula:C16H30O CAS:0-00-0 MolWeight:238 RetIndex:1705
CompName:Cyclopropane, 1-(1-hydroxy-1-heptyl)-2-methylene-3-pentyl- $$ 1-(2-Methylene-3-pentylcyclopropyl)-1-heptanol # $$

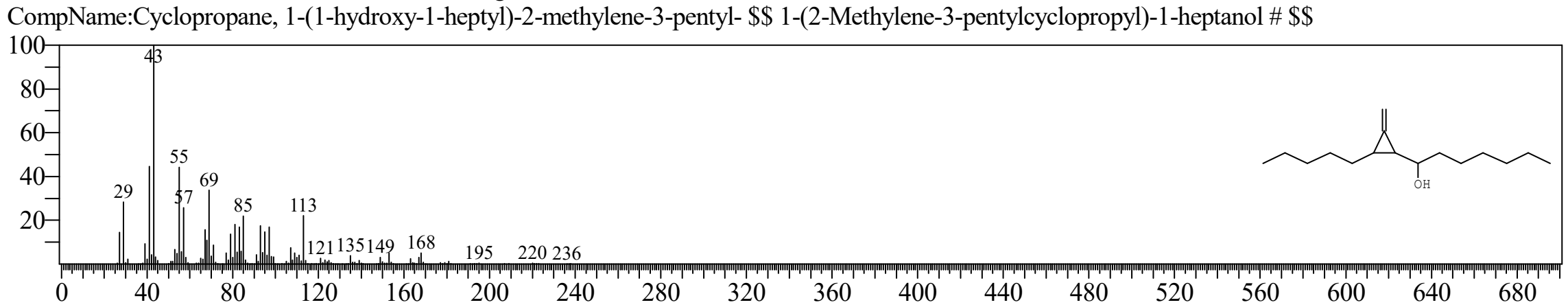

<< Target >>
Line#:109 R.Time:16.440(Scan#:2589) MassPeaks:123
RawMode:Averaged 16.435-16.445(2588-2590) BasePeak:124.10(185185)
BG Mode:Calc. from Peak Group 1 - Event 1 Scan

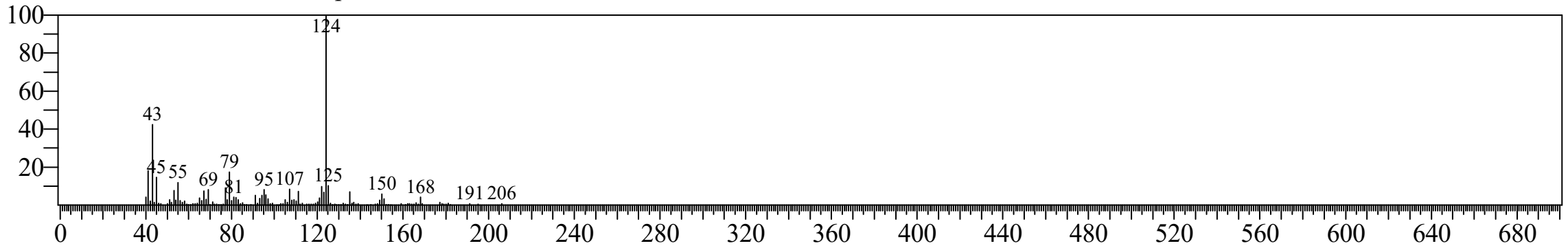

Hit#:1 Entry:63975 Library:NIST14.lib
SI:81 Formula:C13H18O3 CAS:39763-33-2 MolWeight:222 RetIndex:0
CompName:(S,E)-4-Hydroxy-3,5,5-trimethyl-4-(3-oxobut-1-en-1-yl)cyclohex-2-enone $$ 2-Cyclohexen-1-one, 4-hydroxy-3,5,5-trimethyl-4-[(1E)-3-oxo-1-l

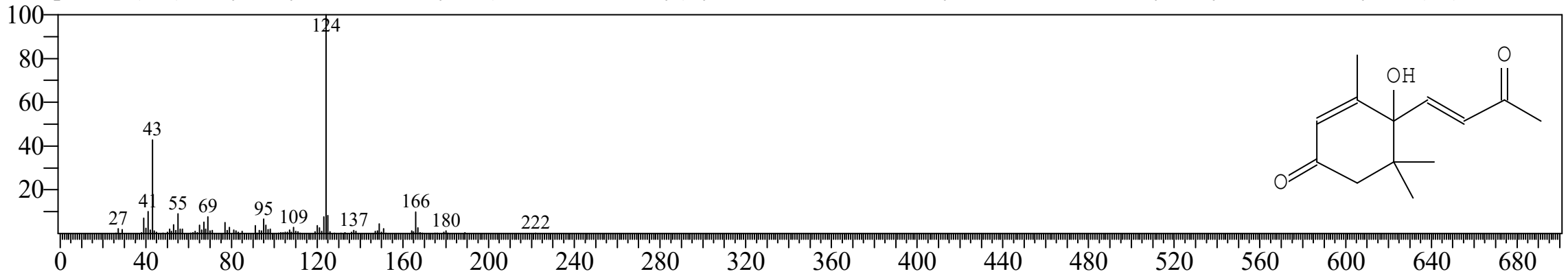

Hit#:2 Entry:135381 Library:WILEY8.LIB
SI:80 Formula:C13H18O3 CAS:7070-24-8 MolWeight:222 RetIndex:0
CompName:4-HYDROXY-3,5,5-TRIMETHYL-4-[(1E)-3-OXO-1-BUTENYL]-2-CYCLOHEXEN-1-ONE # $$ 2-CYCLOHEXEN-1-ONE, 4-HYDROXY

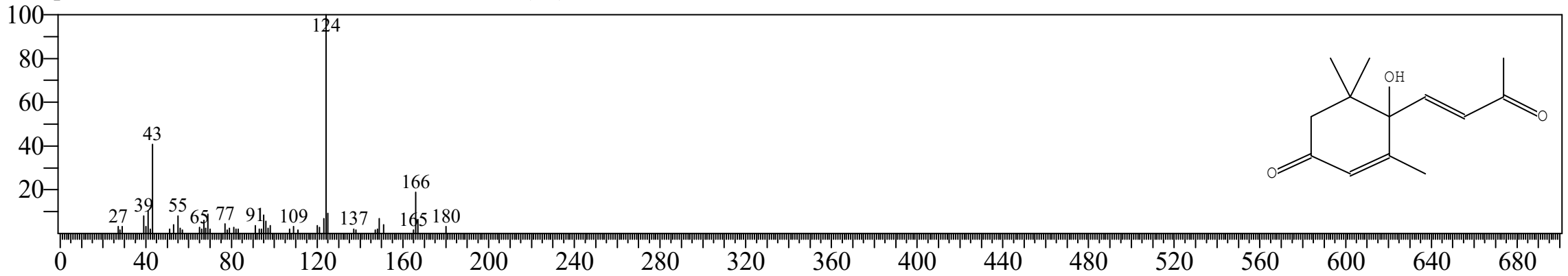

Hit#:3 Entry:21564 Library:NIST14s.lib
SI:80 Formula:C13H18O3 CAS:7070-24-8 MolWeight:222 RetIndex:1751
CompName:2-Cyclohexen-1-one, 4-hydroxy-3,5,5-trimethyl-4-(3-oxo-1-butenyl)- $$ 4-Hydroxy-3,5,5-trimethyl-4-[3-oxo-1-butenyl]-2-cyclohexen-1-one $$

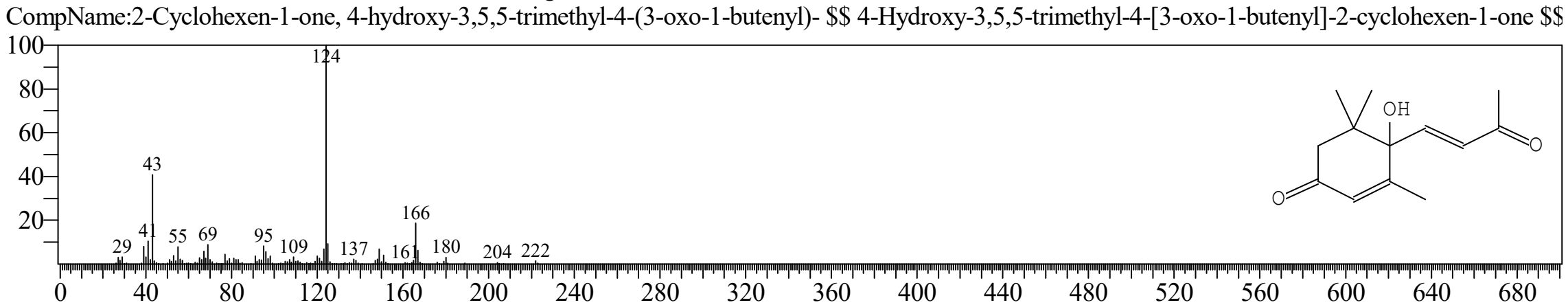

<< Target >>
Line#:110 R.Time:16.725(Scan#:2646) MassPeaks:168
RawMode:Averaged 16.720-16.730(2645-2647) BasePeak:68.05(2307529)
BG Mode:Calc. from Peak Group 1 - Event 1 Scan

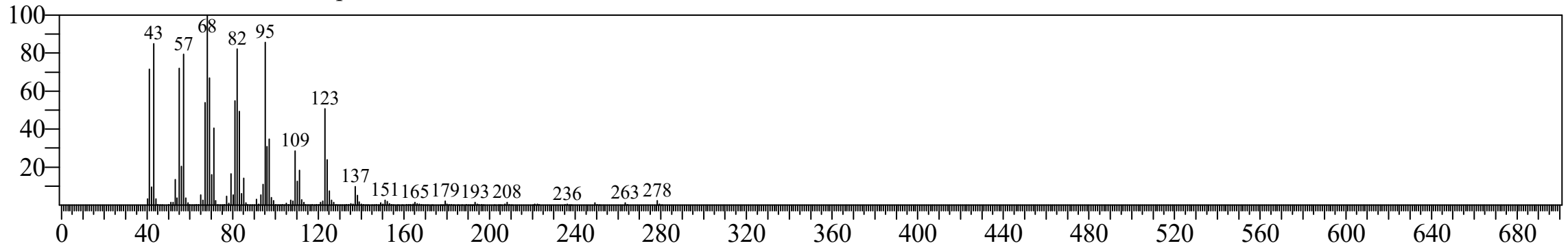

Hit#:1 Entry:111732 Library:NIST14.lib
SI:96 Formula:C20H38 CAS:504-96-1 MolWeight:278 RetIndex:0
CompName:Neophytadiene $$ 7,11,15-Trimethyl-3-methylenehexadec-1-ene $$ 1-Hexadecene, 7,11,15-trimethyl-3-methylene- $$ 1,3-Butadiene, 2-(4,8,12-

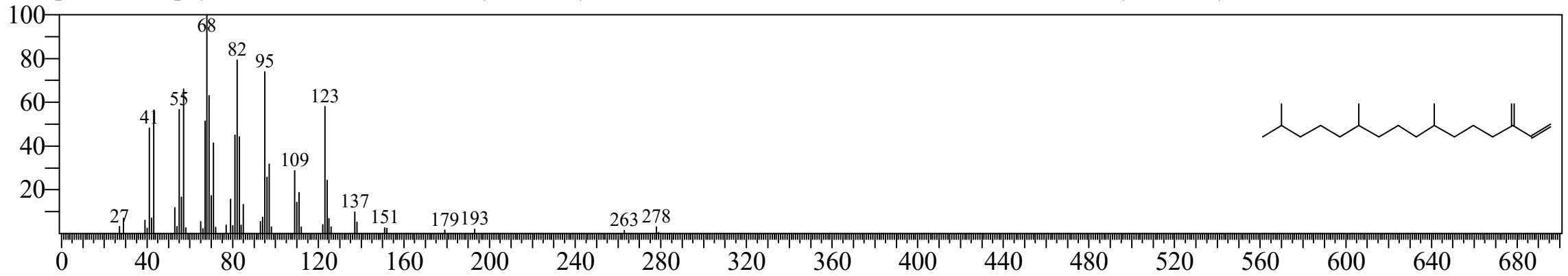

Hit#:2 Entry:235588 Library:WILEY8.LIB
SI:95 Formula:C20H40O CAS:150-86-7 MolWeight:296 RetIndex:0
CompName:2-HEXADECEN-1-OL, 3,7,11,15-TETRAMETHYL-, [R-[R*,R*-(E)]]- $$ 3,7,11,15-TETRAMETHYLHEXADEC-2-EN-1-OL $$ (2E)-3,7,1

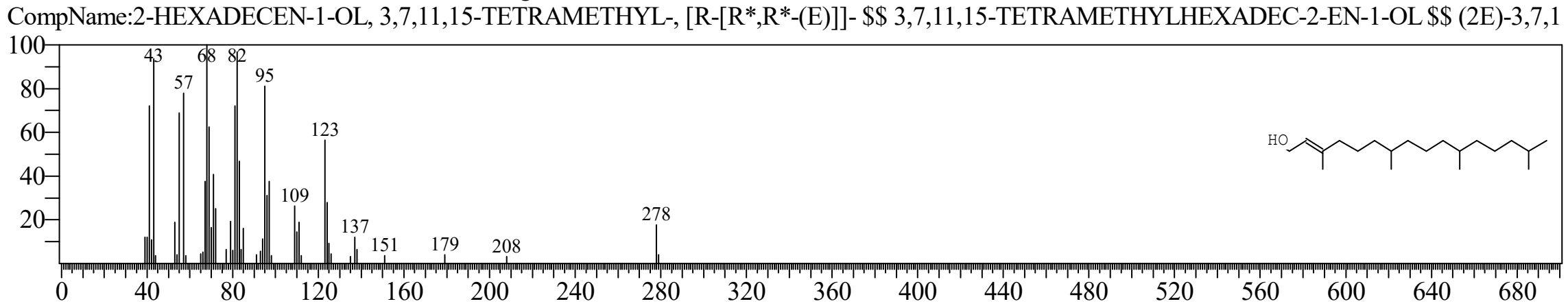

Hit#:3 Entry:212507 Library:WILEY8.LIB
SI:94 Formula:C20H38 CAS:504-96-1 MolWeight:278 RetIndex:0
CompName:2,6,10-TRIMETHYL,14-ETHYLENE-14-PENTADECNE $$ NEOPHYTADIENE

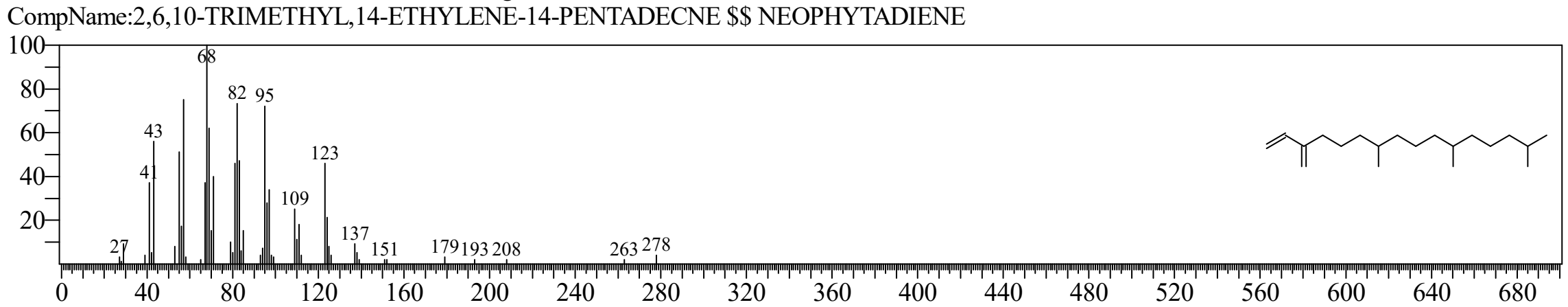

<< Target >>
Line#:111   R.Time:16.975(Scan#:2696) MassPeaks:131
RawMode:Averaged 16.970-16.980(2695-2697) BasePeak:43.05(254513)
BG Mode:Calc. from Peak Group 1 - Event 1 Scan

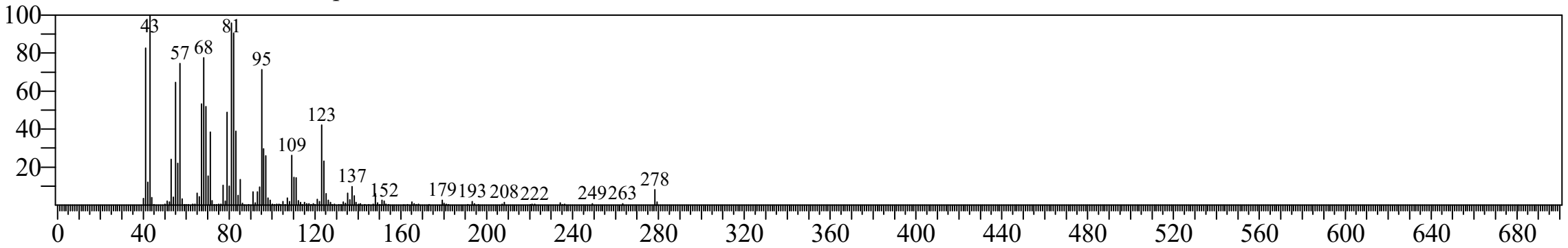

Hit#:1 Entry:127774 Library:NIST14.lib
SI:94 Formula:C20H40O CAS:102608-53-7 MolWeight:296 RetIndex:2045
CompName:3,7,11,15-Tetramethyl-2-hexadecen-1-ol $$ 2-Hexadecen-1-ol, 3,7,11,15-tetramethyl $$

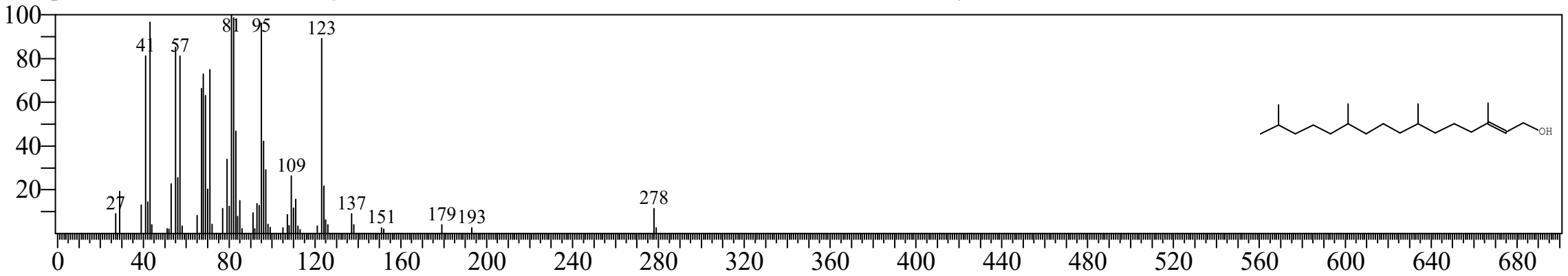

Hit#:2 Entry:235588 Library:WILEY8.LIB
SI:92 Formula:C20H40O CAS:150-86-7 MolWeight:296 RetIndex:0
CompName:2-HEXADECEN-1-OL, 3,7,11,15-TETRAMETHYL-, [R-[R*,R*-(E)]]- $$ 3,7,11,15-TETRAMETHYLHEXADEC-2-EN-1-OL $$ (2E)-3,7,1

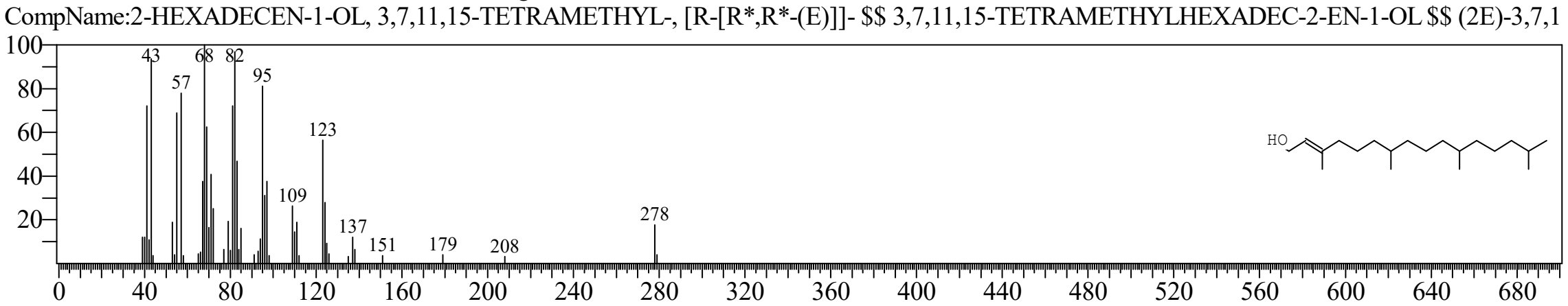

Hit#:3 Entry:111732 Library:NIST14.lib
SI:91 Formula:C20H38 CAS:504-96-1 MolWeight:278 RetIndex:0
CompName:Neophytadiene $$ 7,11,15-Trimethyl-3-methylenehexadec-1-ene $$ 1-Hexadecene, 7,11,15-trimethyl-3-methylene- $$ 1,3-Butadiene, 2-(4,8,12-

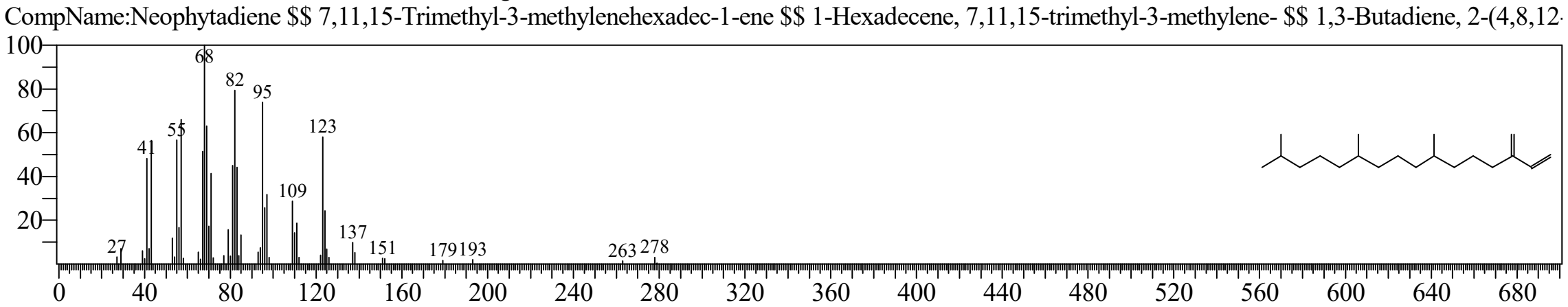

<< Target >>
Line#:112 R.Time:17.160(Scan#:2733) MassPeaks:136
RawMode:Averaged 17.155-17.165(2732-2734) BasePeak:43.05(480545)
BG Mode:Calc. from Peak Group 1 - Event 1 Scan

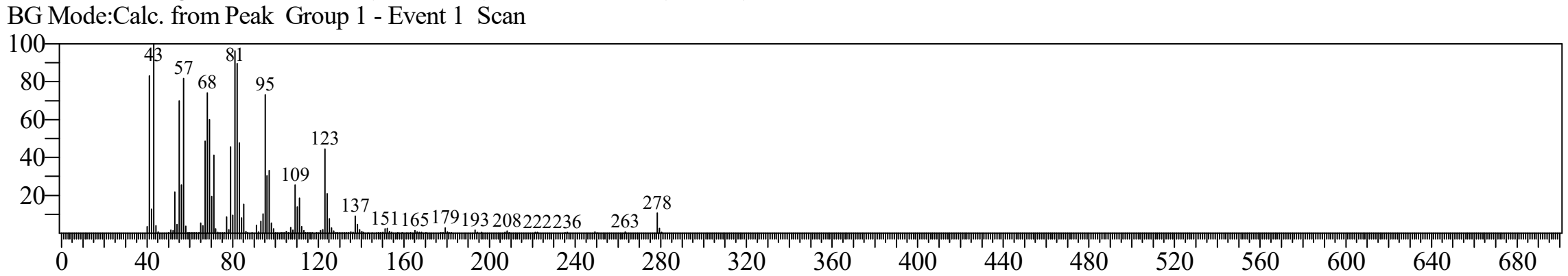

Hit#:1 Entry:127774 Library:NIST14.lib
SI:95 Formula:C20H40O CAS:102608-53-7 MolWeight:296 RetIndex:2045
CompName:3,7,11,15-Tetramethyl-2-hexadecen-1-ol $$ 2-Hexadecen-1-ol, 3,7,11,15-tetramethyl $$

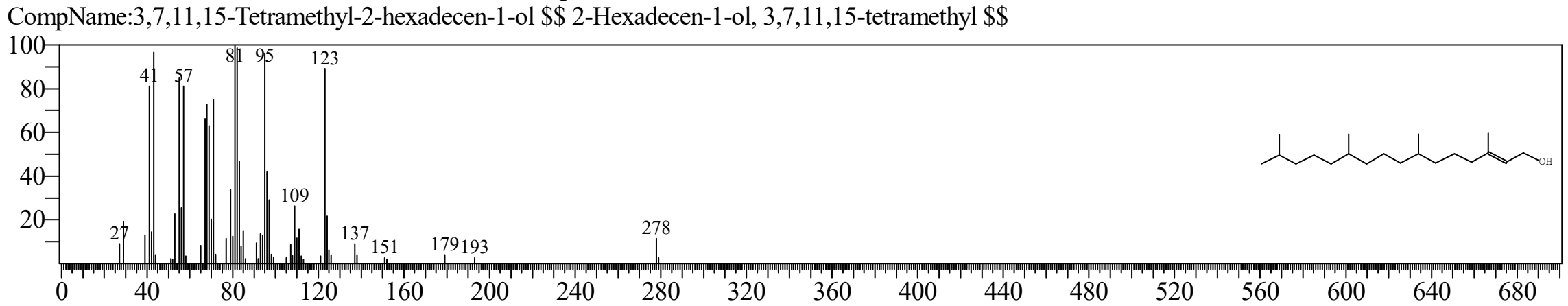

Hit#:2 Entry:235588 Library:WILEY8.LIB
SI:94 Formula:C20H40O CAS:150-86-7 MolWeight:296 RetIndex:0
CompName:2-HEXADECEN-1-OL, 3,7,11,15-TETRAMETHYL-, [R-[R*,R*-(E)]]- $$ 3,7,11,15-TETRAMETHYLHEXADEC-2-EN-1-OL $$ (2E)-3,7,1

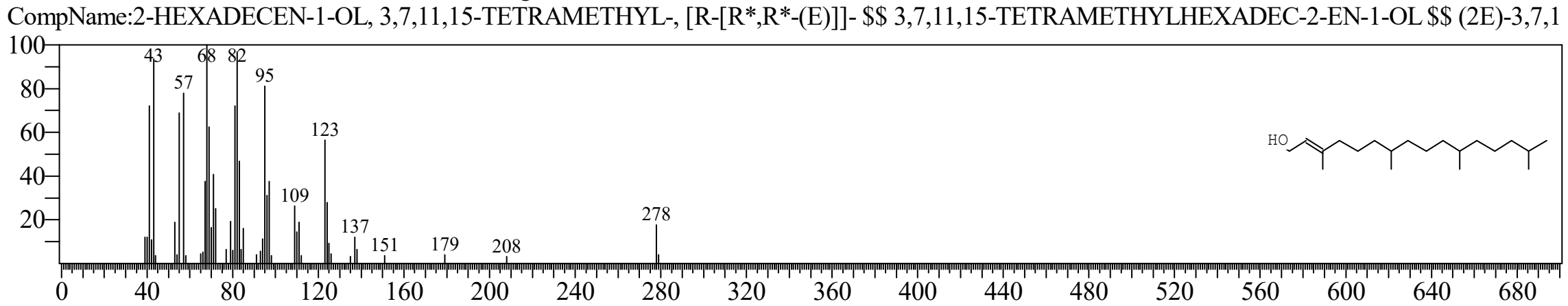

Hit#:3 Entry:111732 Library:NIST14.lib
SI:92 Formula:C20H38 CAS:504-96-1 MolWeight:278 RetIndex:0
CompName:Neophytadiene $$ 7,11,15-Trimethyl-3-methylenehexadec-1-ene $$ 1-Hexadecene, 7,11,15-trimethyl-3-methylene- $$ 1,3-Butadiene, 2-(4,8,12-

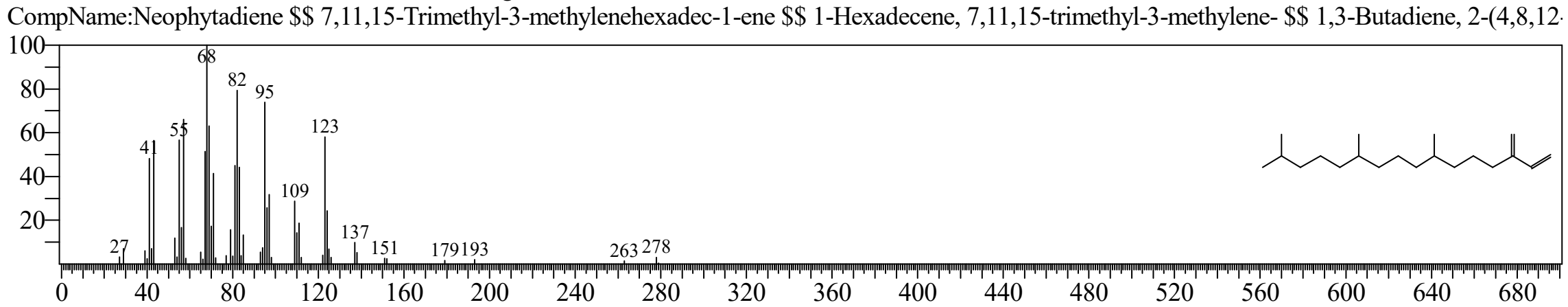

<< Target >>
Line#:113 R.Time:17.385(Scan#:2778) MassPeaks:147
RawMode:Averaged 17.380-17.390(2777-2779) BasePeak:41.05(79351)
BG Mode:Calc. from Peak Group 1 - Event 1 Scan

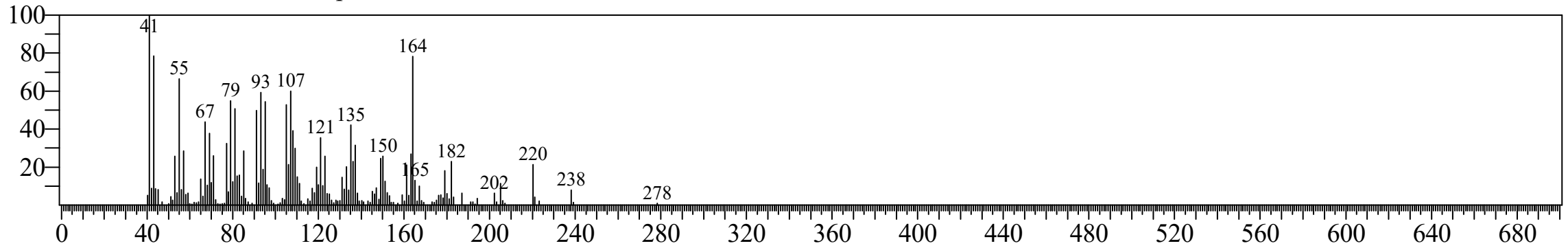

Hit#:1 Entry:77247 Library:NIST14.lib
SI:86 Formula:C15H26O2 CAS:0-00-0 MolWeight:238 RetIndex:1840
CompName:4,4,8-Trimethyltricyclo[6.3.1.0(1,5)]dodecane-2,9-diol

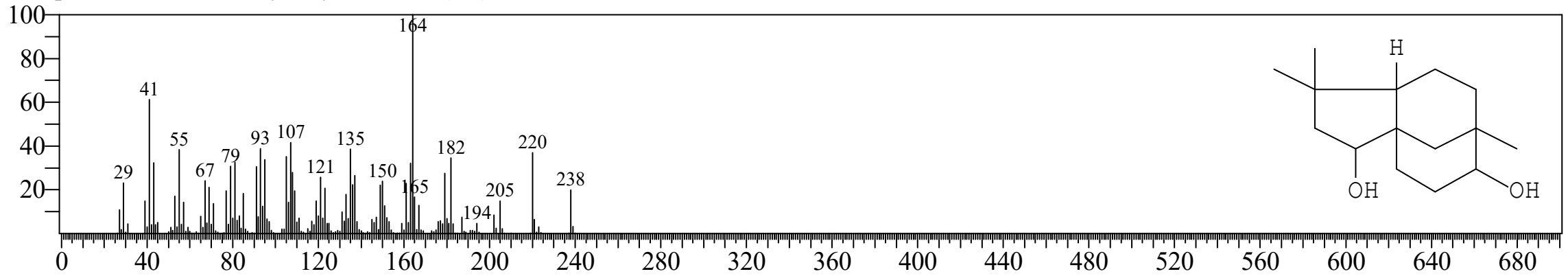

Hit#:2 Entry:158767 Library:WILEY8.LIB
SI:86 Formula:C15H26O2 CAS:0-00-0 MolWeight:238 RetIndex:0
CompName:4,4,8-TRIMETHYL-TRICYCLO[6.3.1.0 1,5]DODECANE-2,9-DIOL

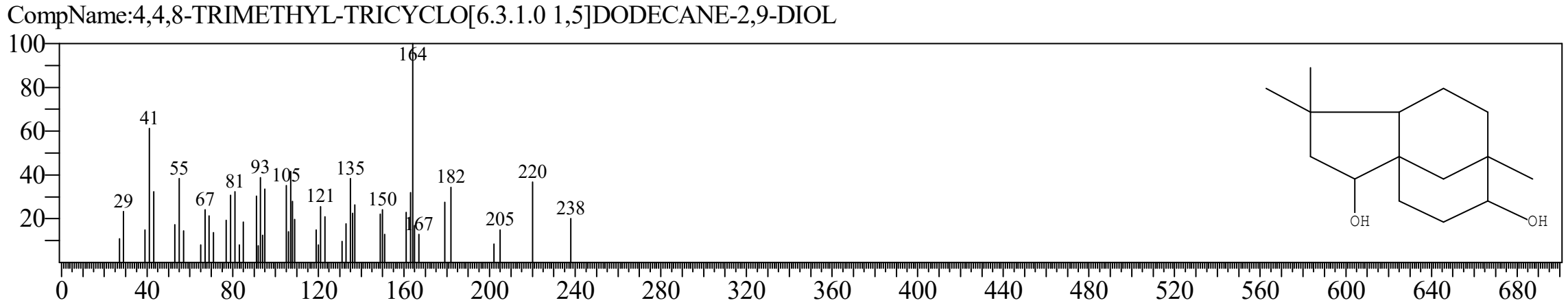

Hit#:3 Entry:77248 Library:NIST14.lib
SI:85 Formula:C15H26O2 CAS:2649-64-1 MolWeight:238 RetIndex:0
CompName:(3S,3aS,6R,7R,9aS)-1,1,7-Trimethyldecahydro-3a,7-methanocyclopenta[8]annulene-3,6-diol $$ 3a,7-Methano-3aH-cyclopentacyclooctene-3,6-

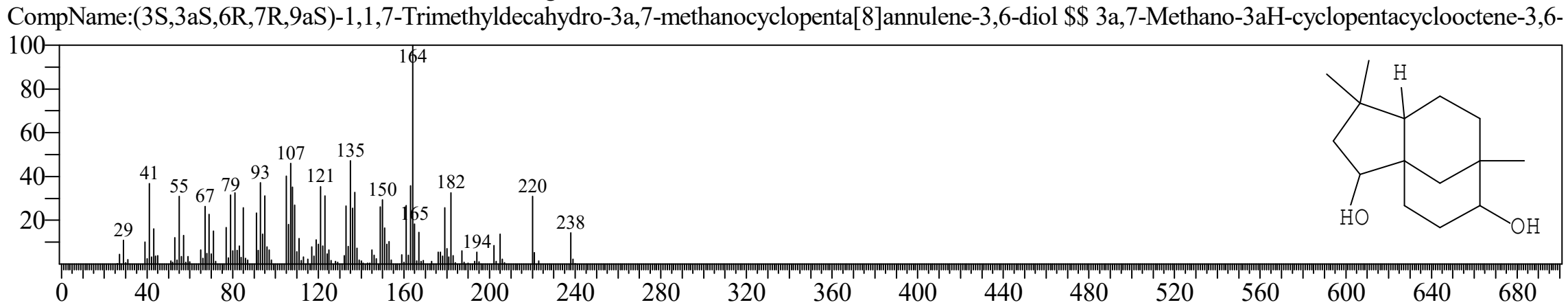

<< Target >>
Line#:114 R.Time:17.510(Scan#:2803) MassPeaks:103
RawMode:Averaged 17.505-17.515(2802-2804) BasePeak:168.10(13219)
BG Mode:Calc. from Peak Group 1 - Event 1 Scan

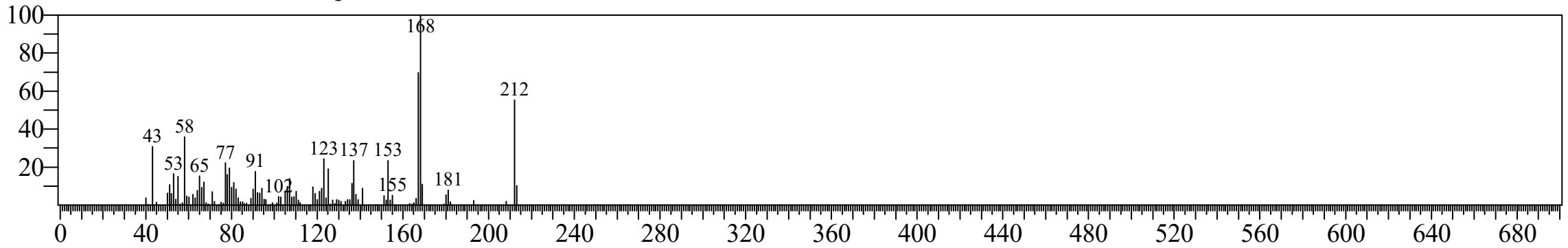

Hit#:1 Entry:55944 Library:NIST14.lib
SI:74 Formula:C11H16O4 CAS:63543-12-4 MolWeight:212 RetIndex:0
CompName:5-(3-Hydroxypropyl)-2,3-dimethoxyphenol $$ Benzenepropanol, 3-hydroxy-4,5-dimethoxy- $$

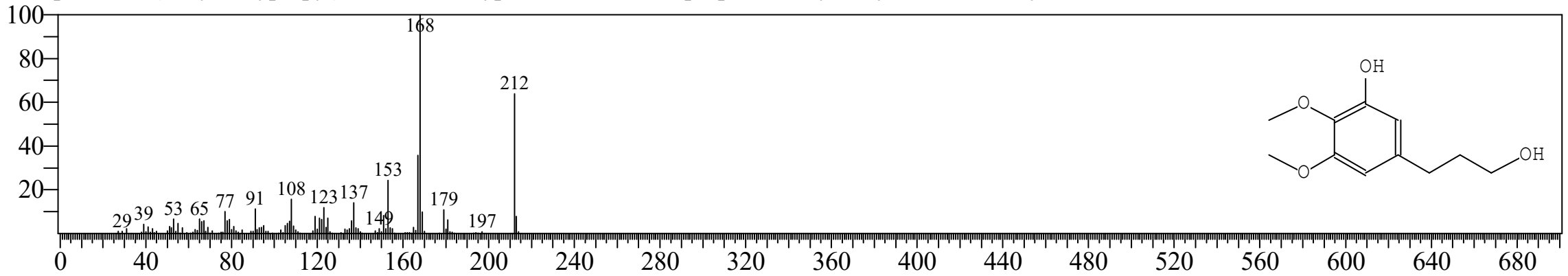

Hit#:2 Entry:44648 Library:NIST14.lib
SI:70 Formula:C10H15NO3 CAS:2413-00-5 MolWeight:197 RetIndex:1735
CompName:3,5-Dimethoxy-4-hydroxyphenethylamine $$ 4-(2-Aminoethyl)-2,6-dimethoxyphenol # $$

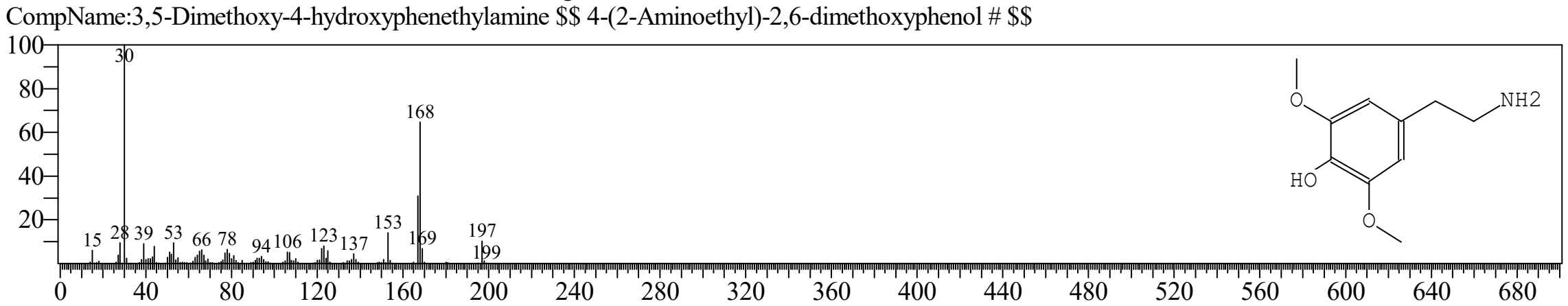

Hit#:3 Entry:25426 Library:NIST14.lib
SI:67 Formula:C9H12O3 CAS:5653-67-8 MolWeight:168 RetIndex:1415
CompName:2,3-Dimethoxybenzyl alcohol $$ Benzenemethanol, 2,3-dimethoxy- $$ o-Veratryl alcohol $$ (2,3-Dimethoxyphenyl)methanol # $$

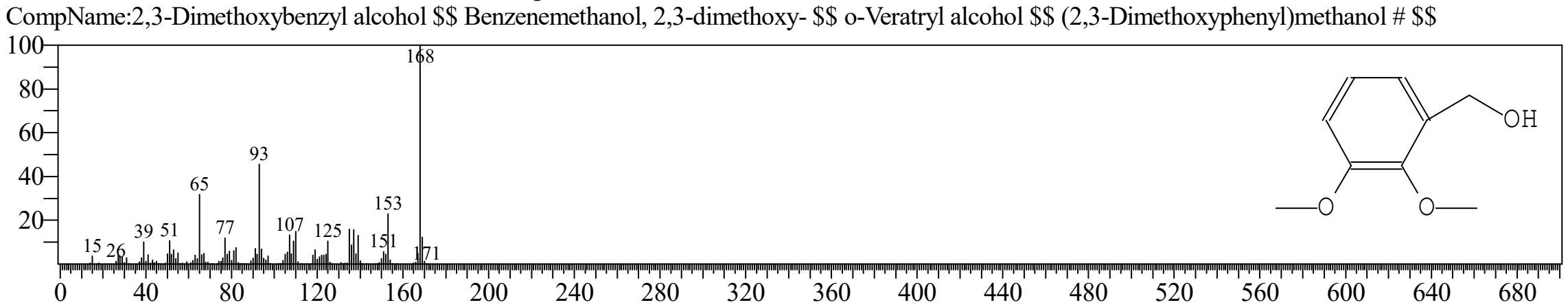

<< Target >>
Line#:115 R.Time:17.575(Scan#:2816) MassPeaks:85
RawMode:Averaged 17.570-17.580(2815-2817) BasePeak:43.05(14990)
BG Mode:Calc. from Peak Group 1 - Event 1 Scan

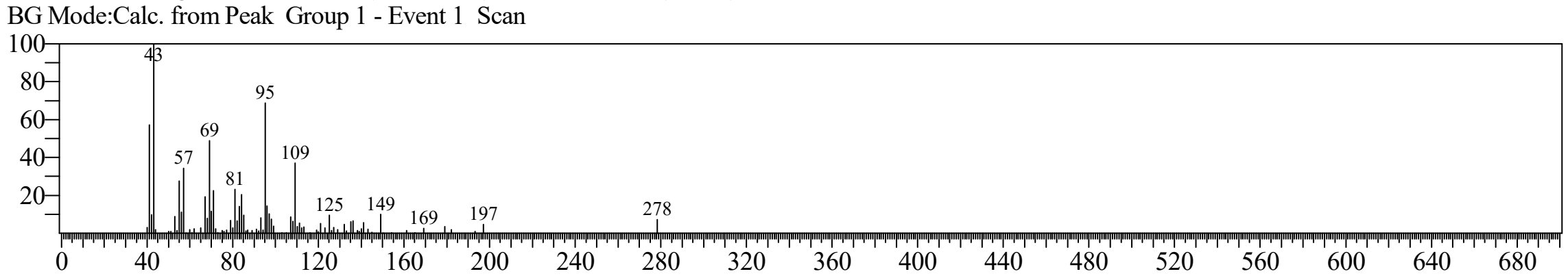

Hit#:1 Entry:72567 Library:NIST14.lib
SI:81 Formula:C9H16BrNO CAS:195194-80-0 MolWeight:233 RetIndex:1635
CompName:2-Piperidinone, N-[4-bromo-n-butyl]- $$ 1-(4-Bromobutyl)-2-piperidinone # $$

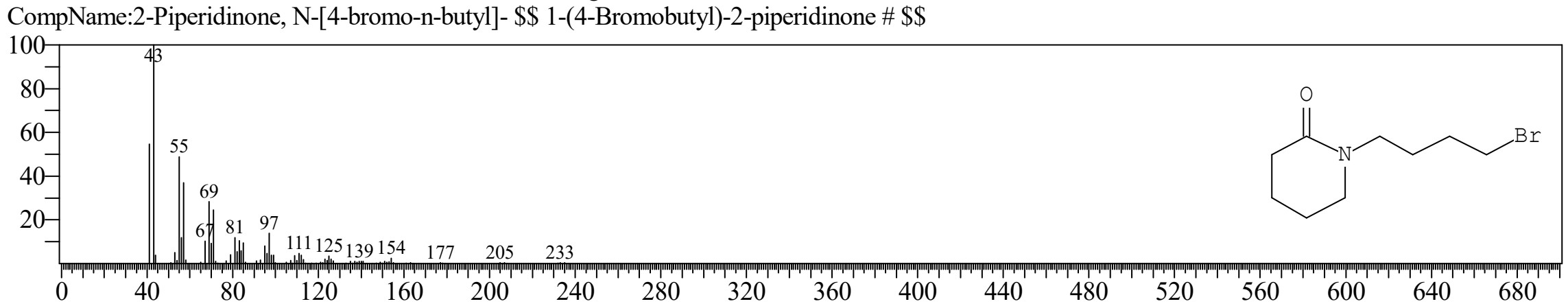

Hit#:2 Entry:102612 Library:NIST14.lib
SI:80 Formula:C16H28O3 CAS:0-00-0 MolWeight:268 RetIndex:1877
CompName:Z-(13,14-Epoxy)tetradec-11-en-1-ol acetate $$ (11Z)-12-(2-Oxiranyl)-11-dodecenyl acetate # $$

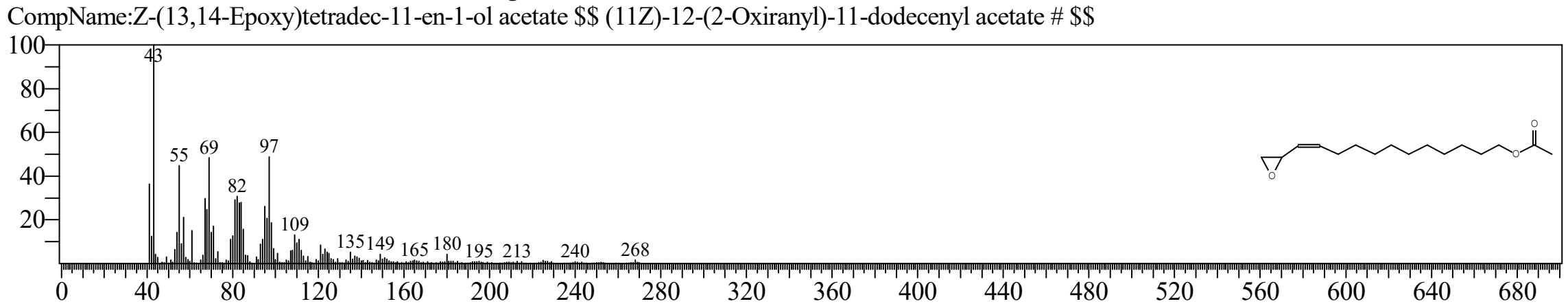

Hit#:3 Entry:44287 Library:NIST14.lib
SI:80 Formula:C13H24O CAS:0-00-0 MolWeight:196 RetIndex:1423
CompName:9-Undecenal, 2,10-dimethyl- $$ 2,10-Dimethyl-9-undecenal # $$

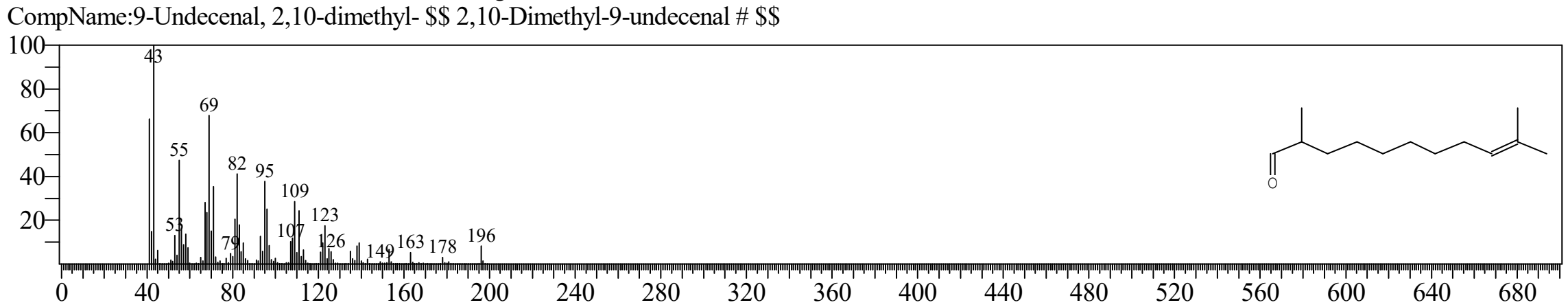

<< Target >>
Line#:116   R.Time:17.665(Scan#:2834) MassPeaks:132
RawMode:Averaged 17.660-17.670(2833-2835) BasePeak:43.05(171120)
BG Mode:Calc. from Peak Group 1 - Event 1 Scan

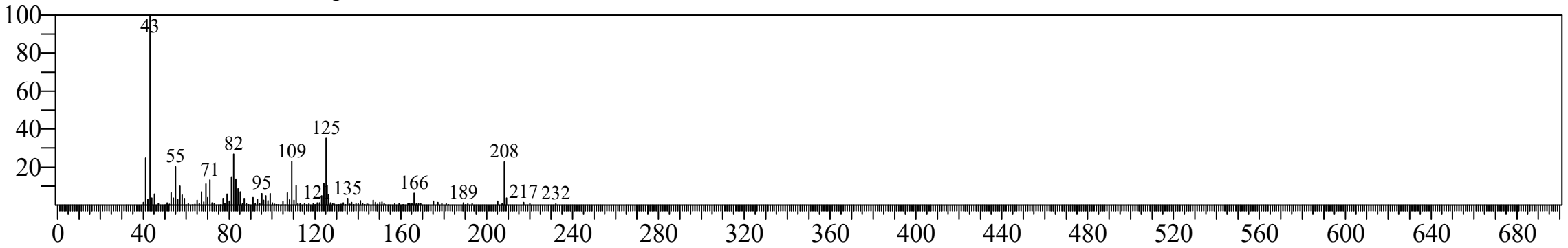

Hit#:1 Entry:67466 Library:NIST14.lib
SI:85 Formula:C13H22O3 CAS:72777-88-9 MolWeight:226 RetIndex:1669
CompName:7-Oxabicyclo[4.1.0]heptan-3-ol, 6-(3-hydroxy-1-butenyl)-1,5,5-trimethyl- $$ 6-[(1E)-3-Hydroxy-1-butenyl]-1,5,5-trimethyl-7-oxabicyclo[4.1.0]

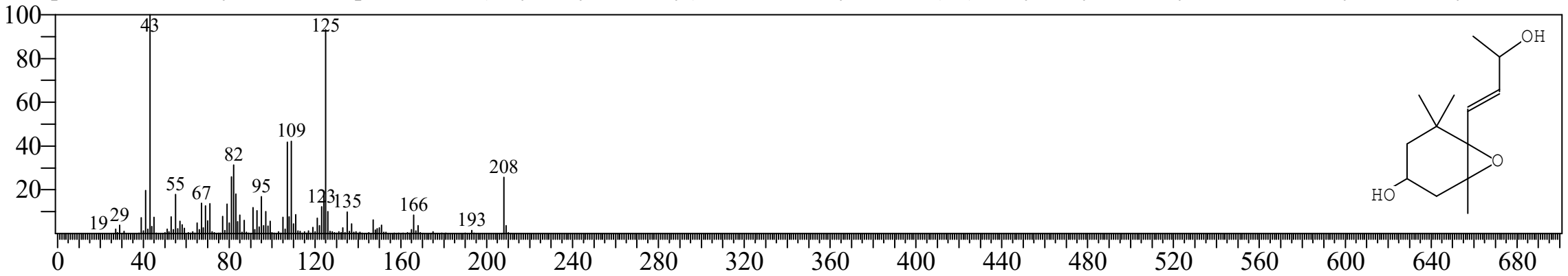

Hit#:2 Entry:54668 Library:NIST14.lib
SI:81 Formula:C13H22O2 CAS:55955-46-9 MolWeight:210 RetIndex:1569
CompName:3-Buten-2-one, 4-(2-hydroxy-2,6,6-trimethylcyclohexyl)- $$ (3E)-4-(2-Hydroxy-2,6,6-trimethylcyclohexyl)-3-buten-2-one # $$

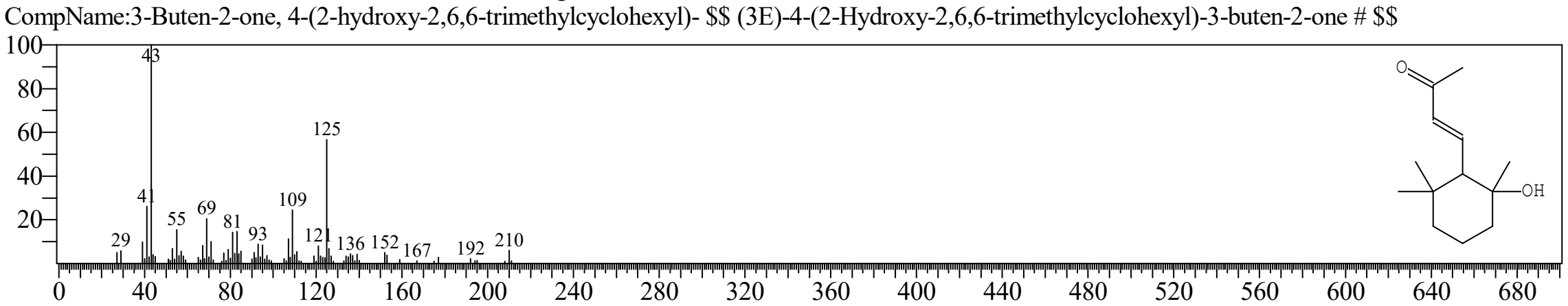

Hit#:3 Entry:118883 Library:WILEY8.LIB
SI:81 Formula:C13H22O2 CAS:0-00-0 MolWeight:210 RetIndex:0
CompName:4-(2-HYDROXY-2,6,6-TRIMETHYL-CYCLOHEXYL)-BUT-3-EN-2-ONE $$ 4-(2-HYDROXY-2,6,6-TRIMETHYLCYCLOHEXYL)-3-BUT

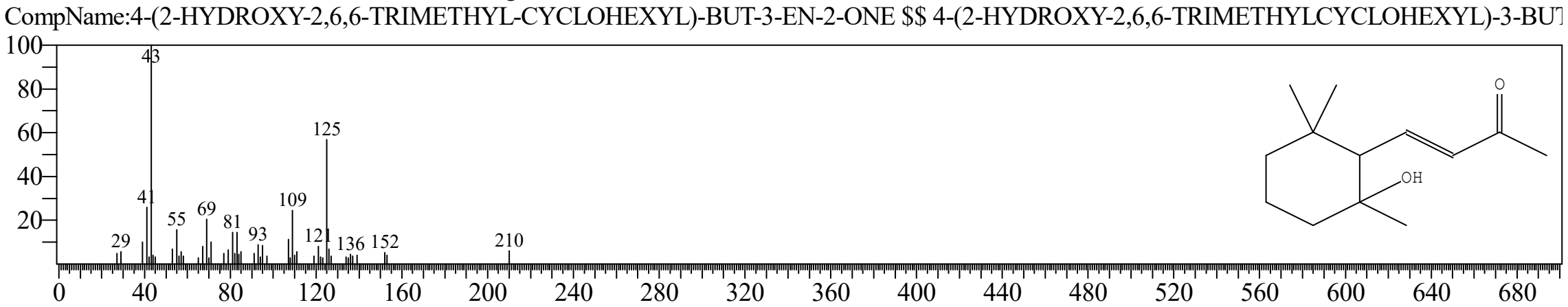

<< Target >>
Line#:117 R.Time:17.820(Scan#:2865) MassPeaks:84
RawMode:Averaged 17.815-17.825(2864-2866) BasePeak:71.05(22150)
BG Mode:Calc. from Peak Group 1 - Event 1 Scan

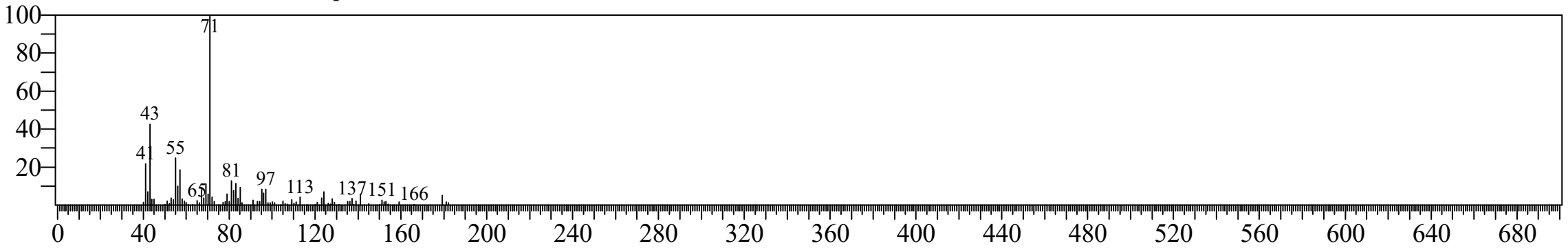

Hit#:1 Entry:127773 Library:NIST14.lib
SI:86 Formula:C20H40O CAS:505-32-8 MolWeight:296 RetIndex:1899
CompName:Isophytol $$ 1-Hexadecen-3-ol, 3,7,11,15-tetramethyl- $$ Hexadec-1-en-3-ol, 3,7,11,15-tetramethyl- $$ 3,7,11,15-Tetramethyl-1-hexadecen-3-c

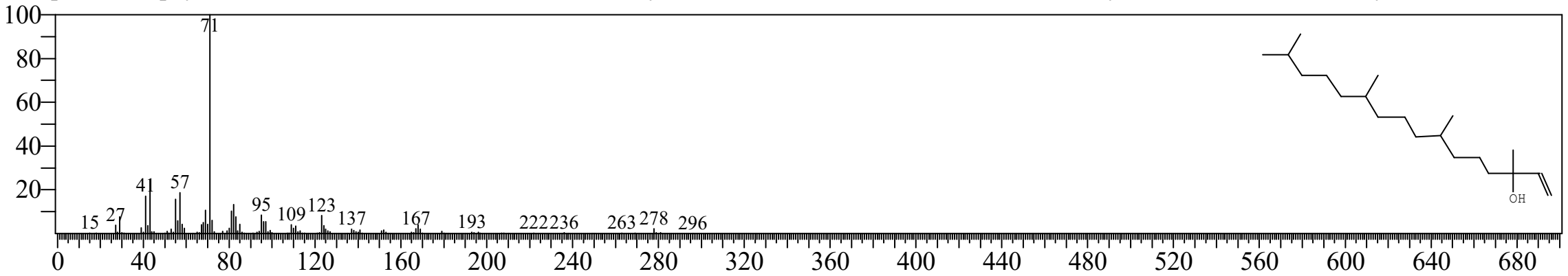

Hit#:2 Entry:28153 Library:NIST14s.lib
SI:85 Formula:C20H40O CAS:505-32-8 MolWeight:296 RetIndex:1899
CompName:Isophytol $$ 1-Hexadecen-3-ol, 3,7,11,15-tetramethyl- $$ Hexadec-1-en-3-ol, 3,7,11,15-tetramethyl- $$ 3,7,11,15-Tetramethyl-1-hexadecen-3-c

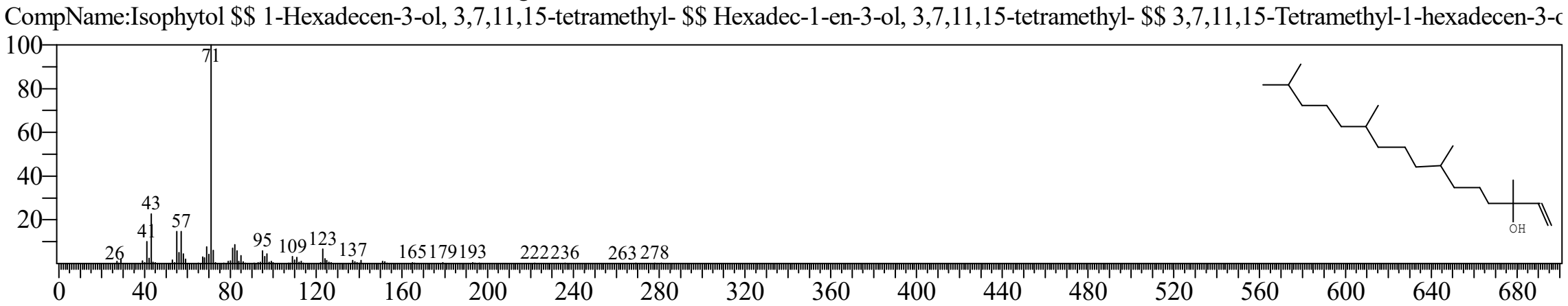

Hit#:3 Entry:235594 Library:WILEY8.LIB
SI:85 Formula:C20H40O CAS:150-86-7 MolWeight:296 RetIndex:0
CompName:2-HEXADECEN-1-OL, 3,7,11,15-TETRAMETHYL-, [R-[R*,R*-(E)]]- $$ 3,7,11,15-TETRAMETHYLHEXADEC-2-EN-1-OL $$ (2E)-3,7,1

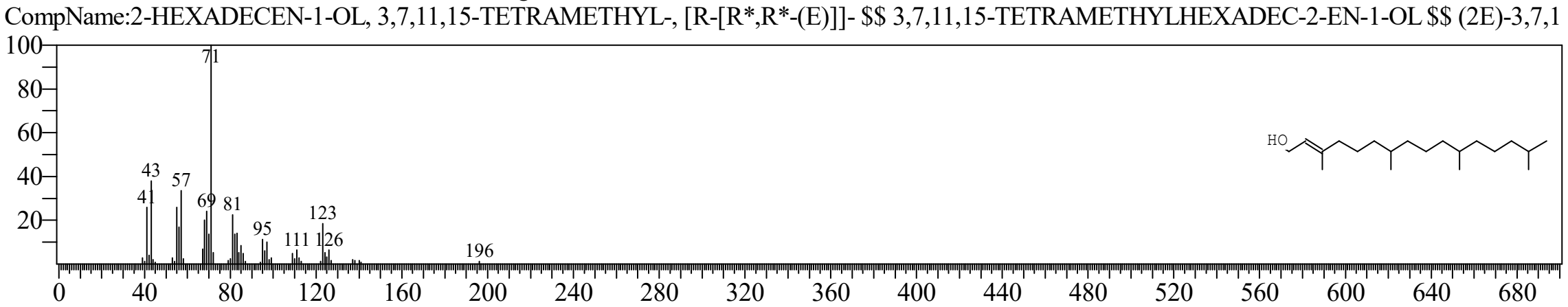

<< Target >>
Line#:118   R.Time:17.930(Scan#:2887) MassPeaks:145
RawMode:Averaged 17.925-17.935(2886-2888) BasePeak:138.05(555220)
BG Mode:Calc. from Peak Group 1 - Event 1 Scan

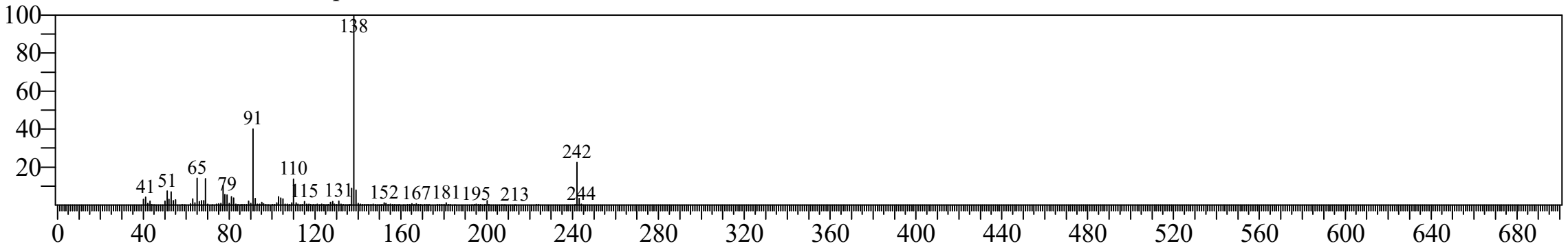

Hit#:1 Entry:95317 Library:WILEY8.LIB
SI:73 Formula:C12H15Cl CAS:0-00-0 MolWeight:194 RetIndex:0
CompName:1-PENTENE, 4-(M-CHLOROPHENYL)-2-METHYL-

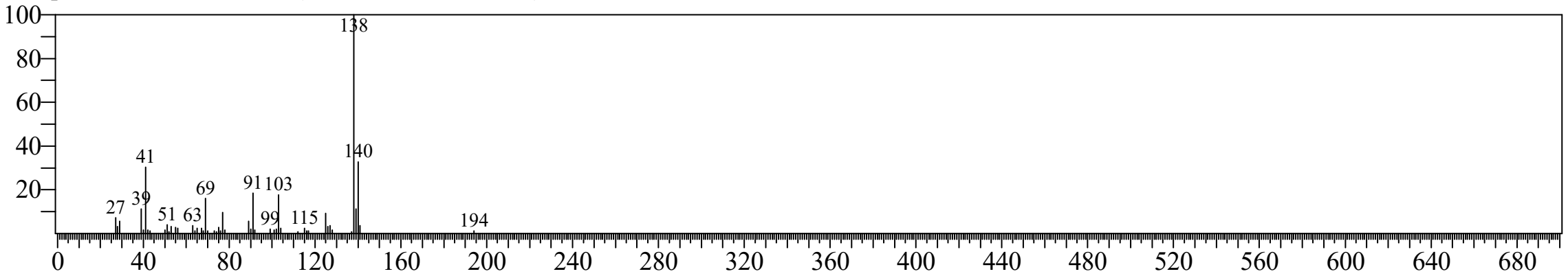

Hit#:2 Entry:95327 Library:WILEY8.LIB
SI:73 Formula:C12H15Cl CAS:74672-12-1 MolWeight:194 RetIndex:0
CompName:BENZENE, 1-CHLORO-3-(1,3-DIMETHYL-3-BUTENYL)- $$ 1-CHLORO-3-(1,3-DIMETHYL-3-BUTENYL)BENZENE

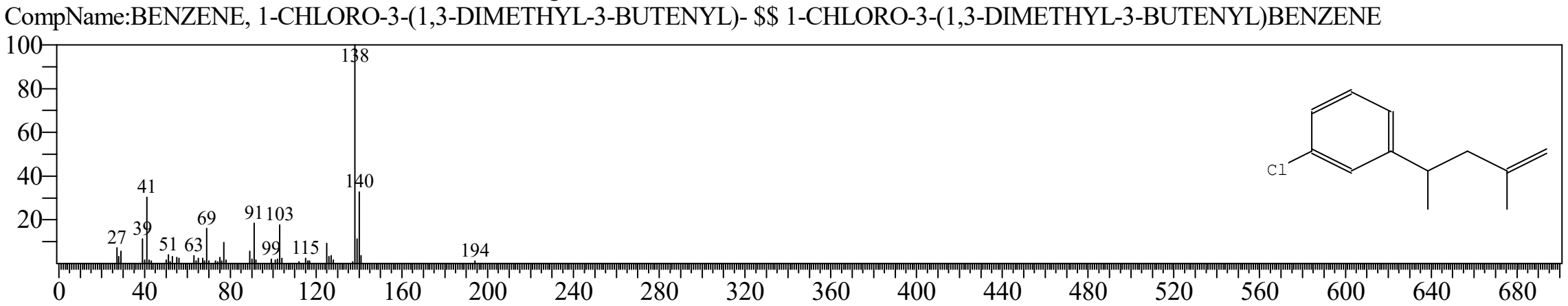

Hit#:3 Entry:305249 Library:WILEY8.LIB
SI:73 Formula:C16H14N2O4S2 CAS:0-00-0 MolWeight:362 RetIndex:0
CompName:4-METHYL-N-[5-(5-METHYL-FURAN-2-YLMETHYLENE)-4-OXO-THIAZOLIDIN-2-YLIDENE]-BENZENESULFONAMIDE $$ 4-ME

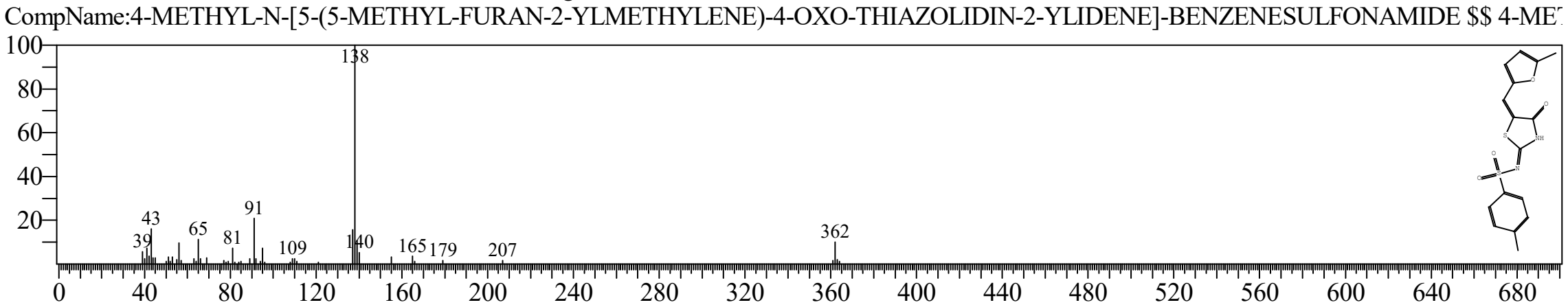

<< Target >>
Line#:119 R.Time:18.030(Scan#:2907) MassPeaks:119
RawMode:Averaged 18.025-18.035(2906-2908) BasePeak:43.05(141970)
BG Mode:Calc. from Peak Group 1 - Event 1 Scan

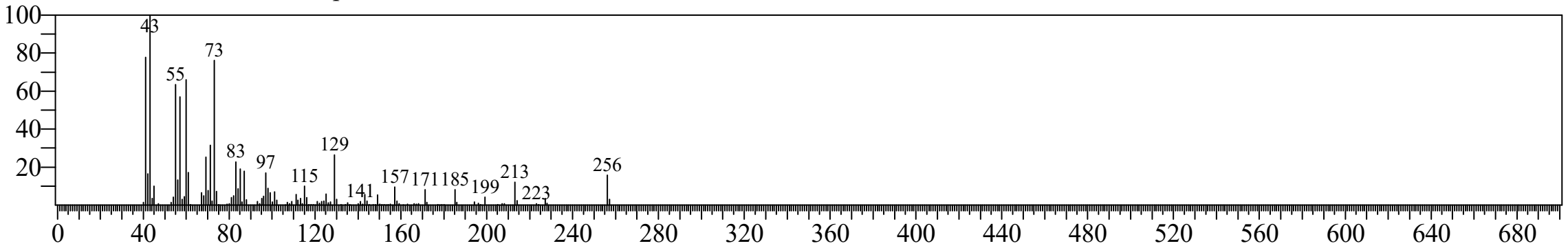

Hit#:1 Entry:92441 Library:NIST14.lib
SI:95 Formula:C16H32O2 CAS:57-10-3 MolWeight:256 RetIndex:1968
CompName:n-Hexadecanoic acid $$ Hexadecanoic acid $$ n-Hexadecoic acid $$ Palmitic acid $$ Pentadecanecarboxylic acid $$ 1-Pentadecanecarboxylic

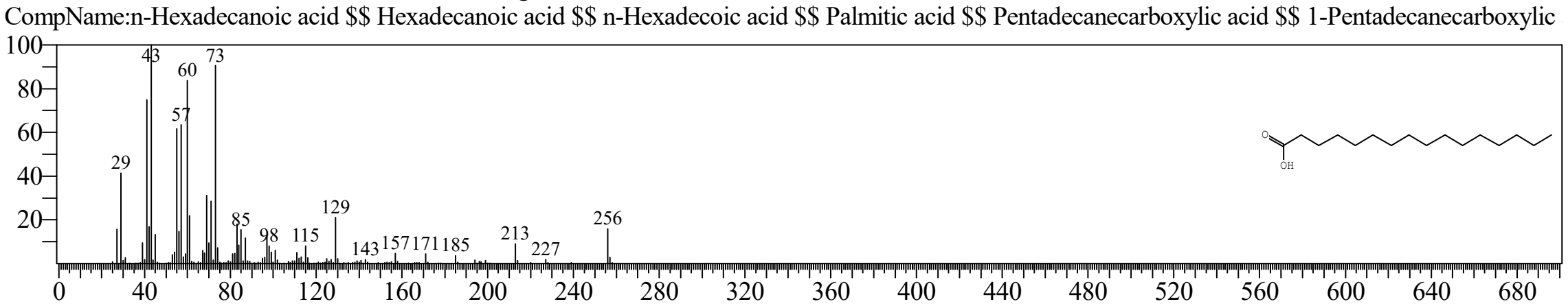

Hit#:2 Entry:183017 Library:WILEY8.LIB
SI:95 Formula:C16H32O2 CAS:57-10-3 MolWeight:256 RetIndex:0
CompName:HEXADECANOIC ACID $$ HEXADECANOATE $$ PALMITATE $$ PALMITIC ACID $$ 1-PENTADECANECARBOXYLIC ACID $$ A

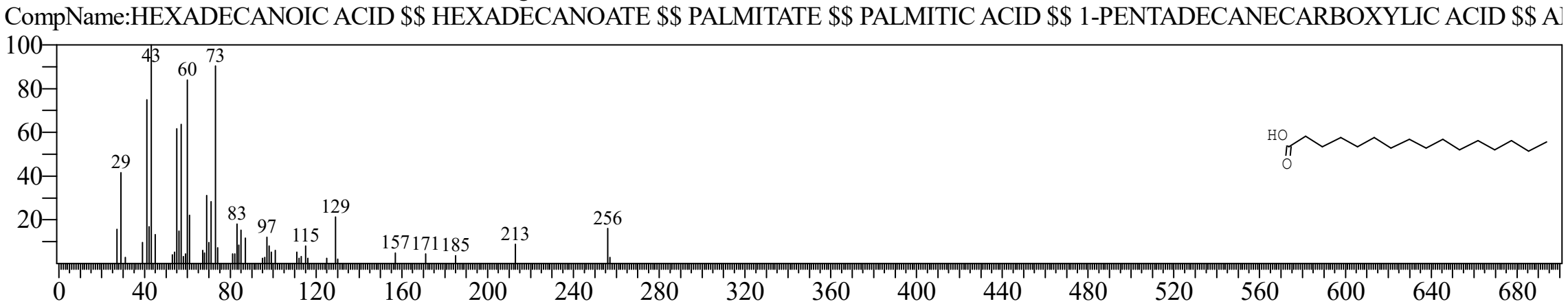

Hit#:3 Entry:183104 Library:WILEY8.LIB
SI:95 Formula:C16H32O2 CAS:57-10-3 MolWeight:256 RetIndex:0
CompName:HEXADECANOIC ACID $$ HEXADECANOATE $$ PALMITATE $$ PALMITIC ACID $$ 1-PENTADECANECARBOXYLIC ACID $$ A

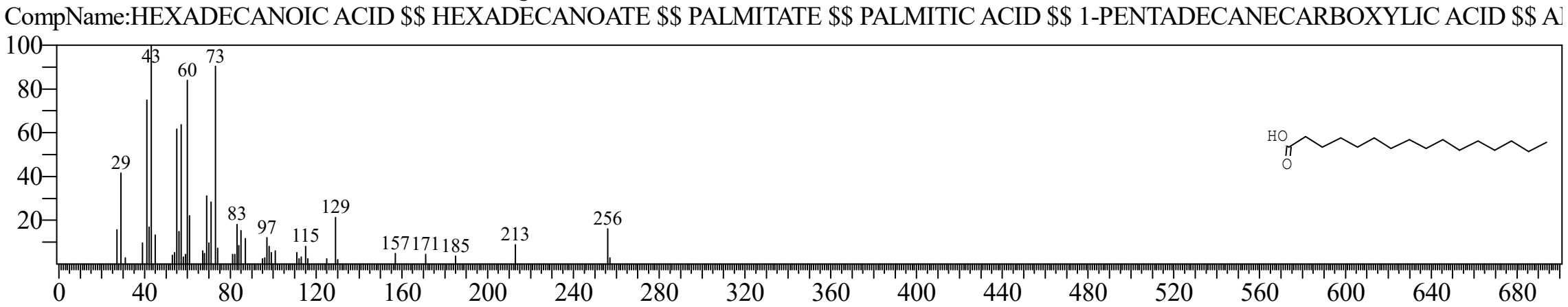

<< Target >>
Line#:120 R.Time:18.235(Scan#:2948) MassPeaks:137
RawMode:Averaged 18.230-18.240(2947-2949) BasePeak:43.05(206790)
BG Mode:Calc. from Peak Group 1 - Event 1 Scan

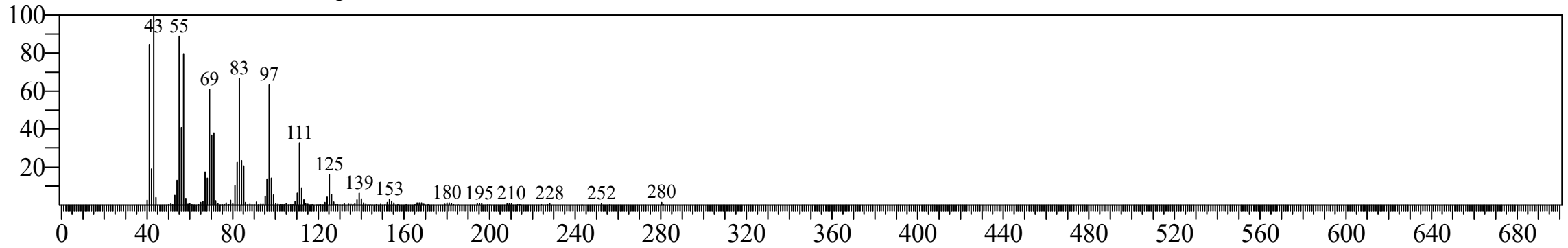

Hit#:1 Entry:25913 Library:NIST14s.lib
SI:98 Formula:C19H38 CAS:18435-45-5 MolWeight:266 RetIndex:1900
CompName:1-Nonadecene $$ Nonadec-1-ene $$

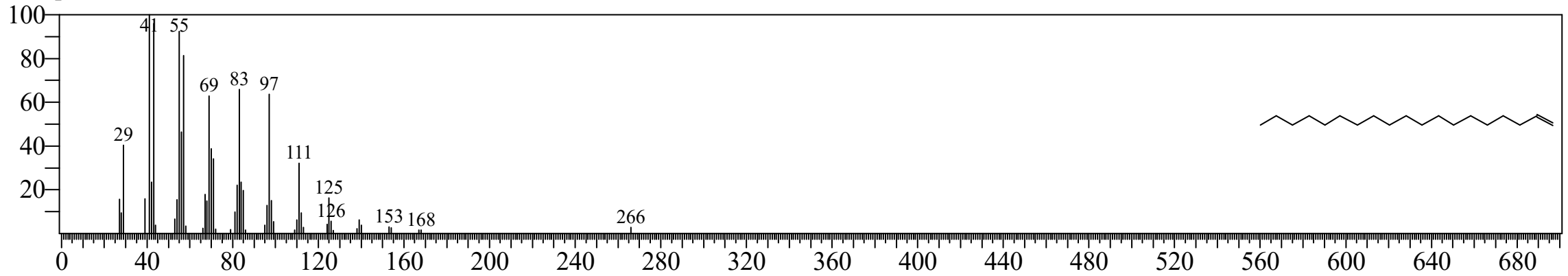

Hit#:2 Entry:28881 Library:NIST14s.lib
SI:97 Formula:C22H44 CAS:1599-67-3 MolWeight:308 RetIndex:2198
CompName:1-Docosene $$ Docos-1-ene $$

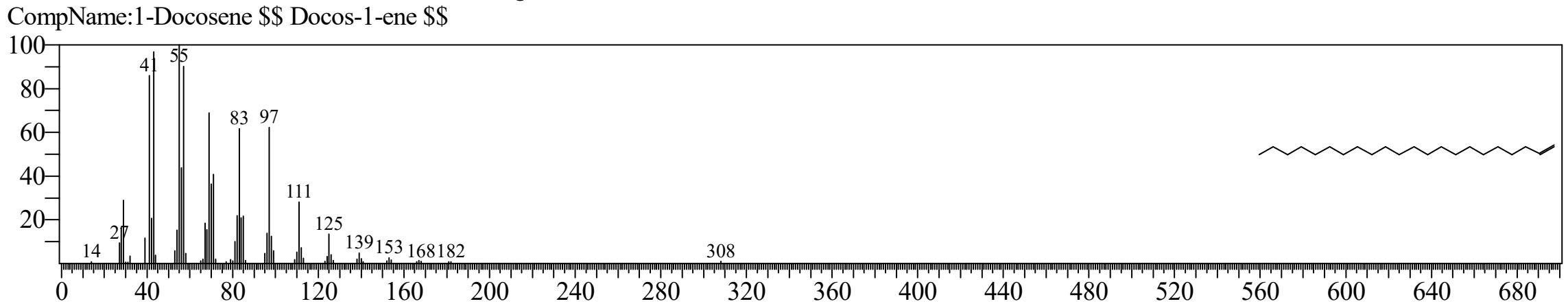

Hit#:3 Entry:89019 Library:NIST14.lib
SI:97 Formula:C17H32O CAS:0-00-0 MolWeight:252 RetIndex:1907
CompName:E-15-Heptadecenal $$ (15E)-15-Heptadecenal # $$

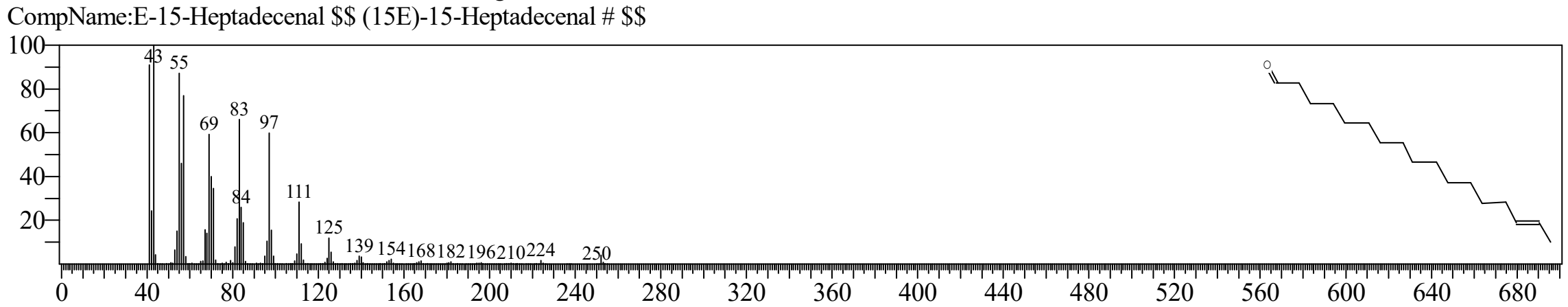

<< Target >>
Line#:121 R.Time:18.375(Scan#:2976) MassPeaks:73
RawMode:Averaged 18.370-18.380(2975-2977) BasePeak:70.05(3459)
BG Mode:Calc. from Peak Group 1 - Event 1 Scan

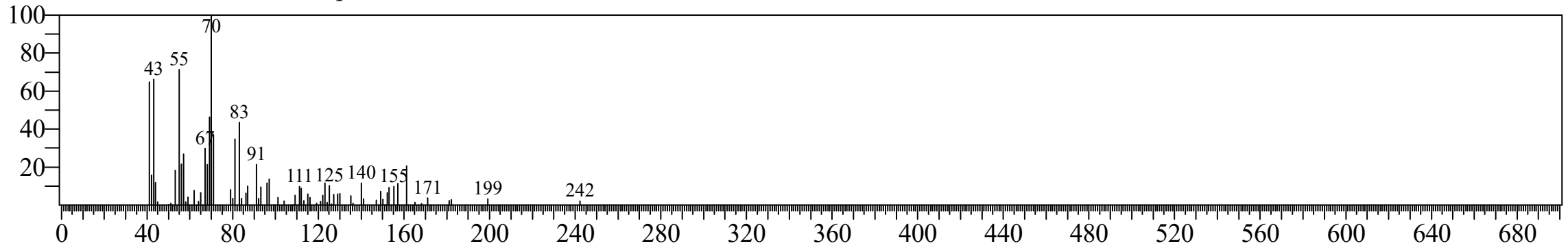

Hit#:1 Entry:99030 Library:WILEY8.LIB
SI:78 Formula:C13H24O CAS:71277-06-0 MolWeight:196 RetIndex:0
CompName:2-TRIDECENAL, (Z)- $$ (Z)-2-TRIDECENAL $$ E-2-TRIDECENAL $$ TRANS-2-TRIDECENAL

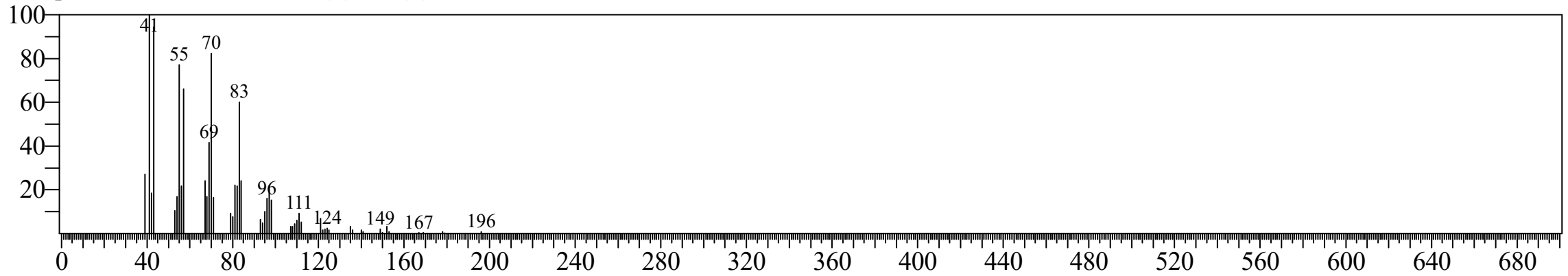

Hit#:2 Entry:99029 Library:WILEY8.LIB
SI:77 Formula:C13H24O CAS:71277-06-0 MolWeight:196 RetIndex:0
CompName:2-TRIDECENAL, (Z)- $$ (Z)-2-TRIDECENAL $$ E-2-TRIDECENAL $$ TRANS-2-TRIDECENAL

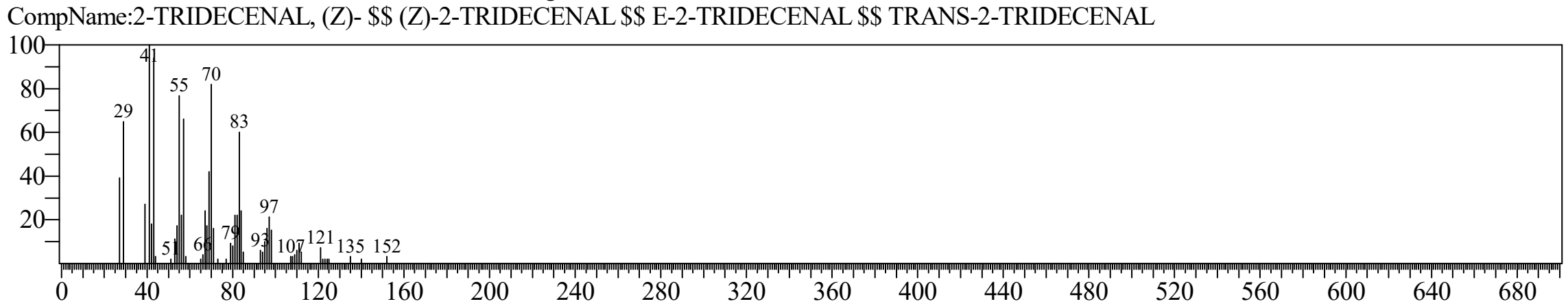

Hit#:3 Entry:31784 Library:WILEY8.LIB
SI:77 Formula:C9H16O CAS:18829-56-6 MolWeight:140 RetIndex:0
CompName:2-NONENAL $$ 2-NONENAL, (E)- $$ (2E)-2-NONENAL # $$ (2E)-2-NONENAL $$ (2E)-2-NONENAL (COMPUTER-GENERATED NAI

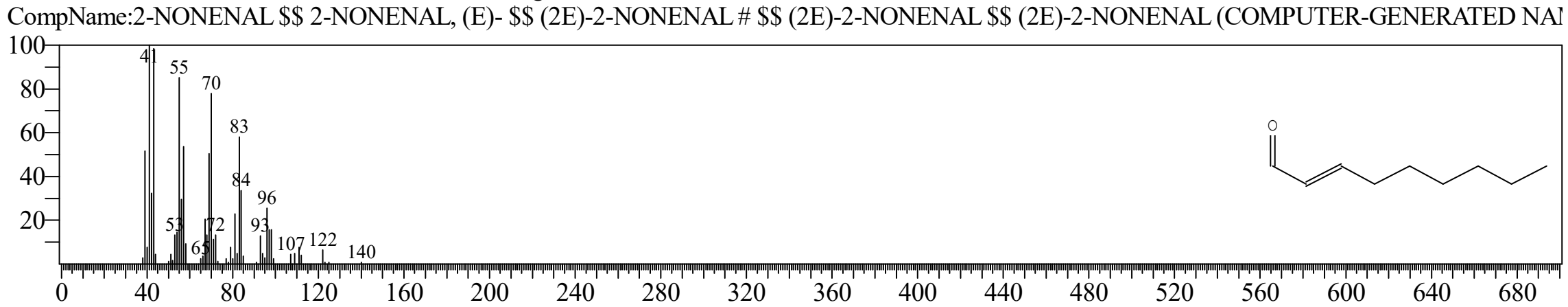

<< Target >>
Line#:122   R.Time:18.460(Scan#:2993) MassPeaks:77
RawMode:Averaged 18.455-18.465(2992-2994) BasePeak:138.05(1367)
BG Mode:Calc. from Peak Group 1 - Event 1 Scan

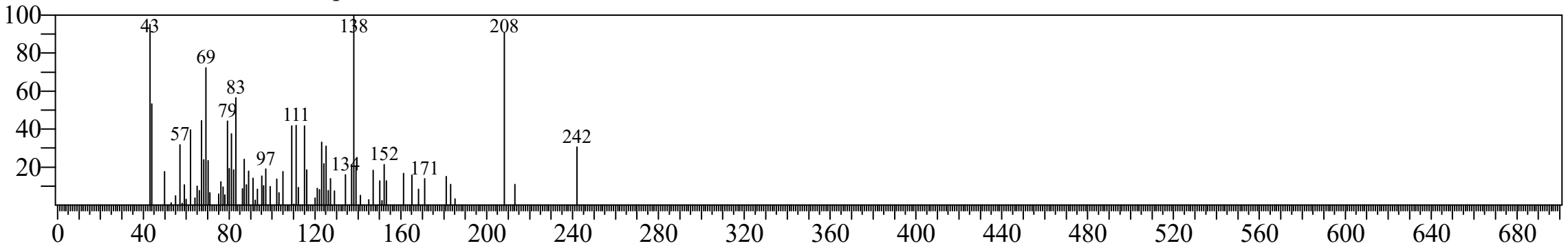

Hit#:1 Entry:116324 Library:WILEY8.LIB
SI:62 Formula:C15H28 CAS:30824-81-8 MolWeight:208 RetIndex:0
CompName:NAPHTHALENE, DECAHYDRO-1,4A-DIMETHYL-7-(1-METHYLETHYL)-, [1S-(1.ALPHA.,4A.ALPHA.,7.ALPHA.,8A.BETA.)]- $$ 7-IS

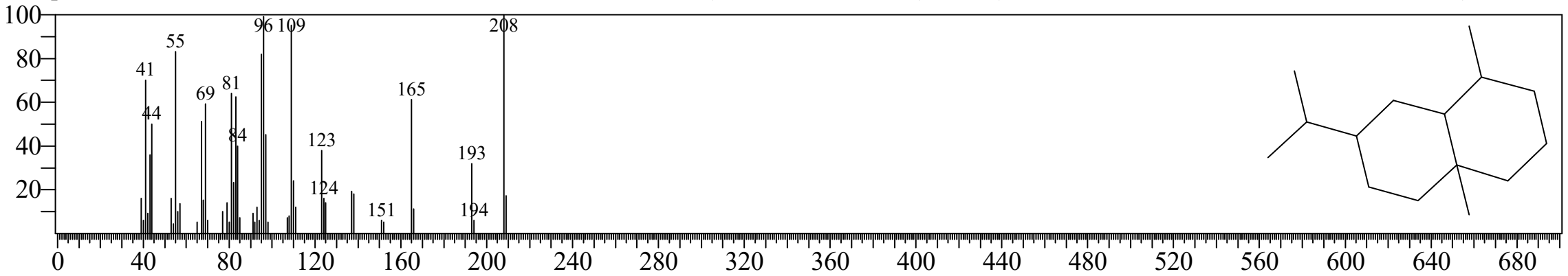

Hit#:2 Entry:78898 Library:NIST14.lib
SI:60 Formula:C15H28O2 CAS:68555-59-9 MolWeight:240 RetIndex:1576
CompName:Tetrahydroionyl acetate $$ Cyclohexanepropanol, .alpha.,2,2,6-tetramethyl-, acetate $$ 2-Butanol, 4-[2,2,6-trimethylcyclohexyl]-, acetate $$ 1-M

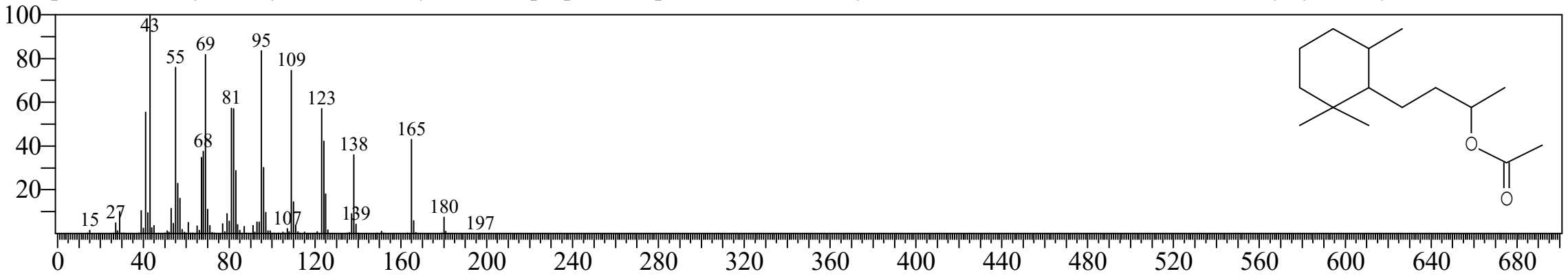

Hit#:3 Entry:102749 Library:NIST14.lib
SI:60 Formula:C17H32O2 CAS:0-00-0 MolWeight:268 RetIndex:1804
CompName:11-Methyl-13-tetradecen-1-ol acetate $$ 11-Methyl-13-tetradecenyl acetate # $$

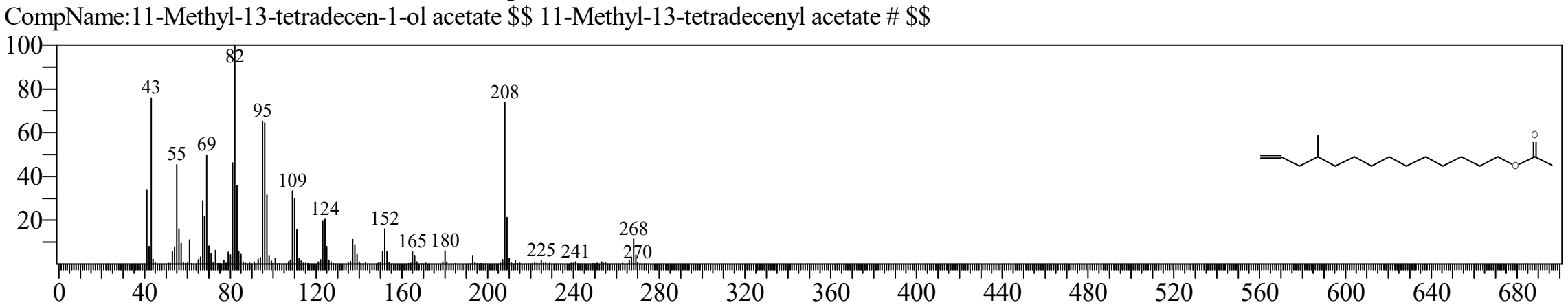

<< Target >>
Line#:123 R.Time:18.550(Scan#:3011) MassPeaks:123
RawMode:Averaged 18.545-18.555(3010-3012) BasePeak:91.05(4046)
BG Mode:Calc. from Peak Group 1 - Event 1 Scan

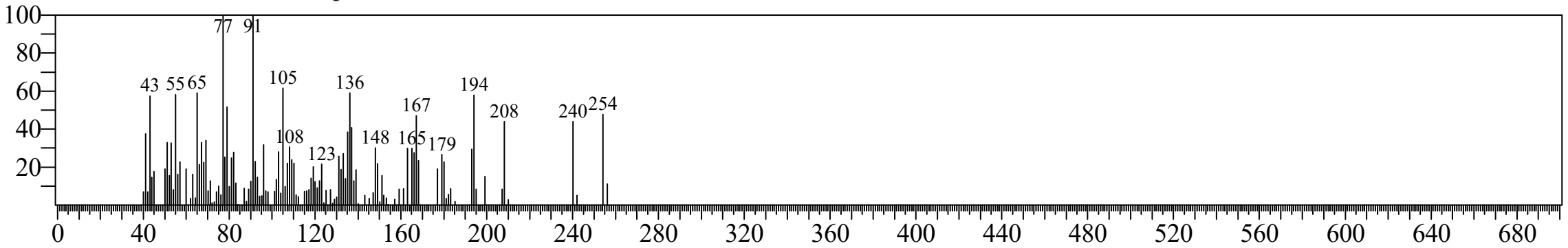

Hit#:1 Entry:160622 Library:WILEY8.LIB
SI:64 Formula:C12H16O5 CAS:57304-91-3 MolWeight:240 RetIndex:0
CompName:2-(2-CARBOXY-ETHYL)-6,6-DIMETHYL-3-OXO-CYCLOHEX-1-ENECARBOXYLIC ACID $$ 2-(2-CARBOXYETHYL)-6,6-DIMETHY

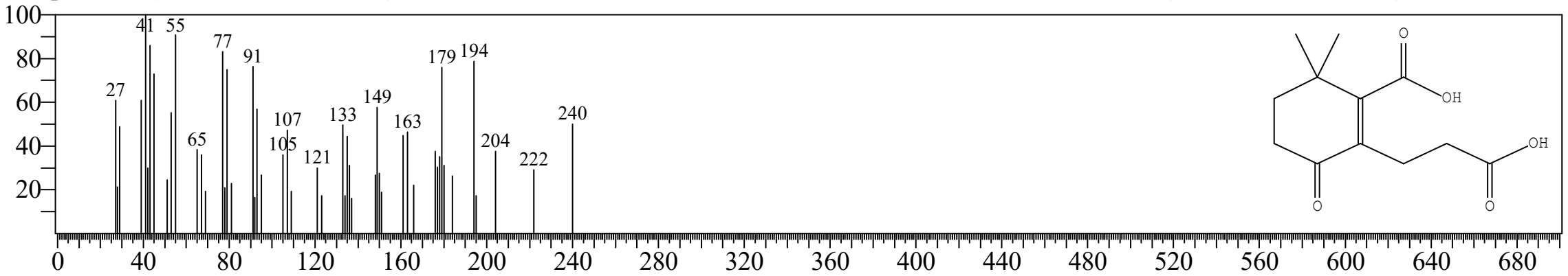

Hit#:2 Entry:78385 Library:NIST14.lib
SI:64 Formula:C12H16O5 CAS:57304-91-3 MolWeight:240 RetIndex:2098
CompName:2-(2-Carboxyethyl)-6,6-dimethyl-3-oxocyclohex-1-enecarboxylic acid $$ 2-(2-Carboxyethyl)-6,6-dimethyl-3-oxo-1-cyclohexene-1-carboxylic a

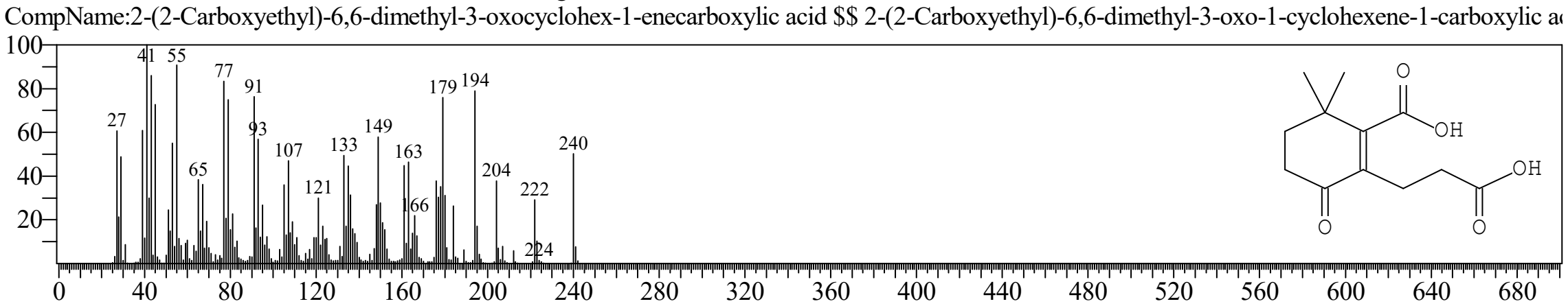

Hit#:3 Entry:116007 Library:WILEY8.LIB
SI:63 Formula:C14H24O CAS:25091-26-3 MolWeight:208 RetIndex:0
CompName:5-TETRADECEN-3-YN-1-OL, (Z)- $$ (Z)-5-TETRADECEN-3-YN-1-OL

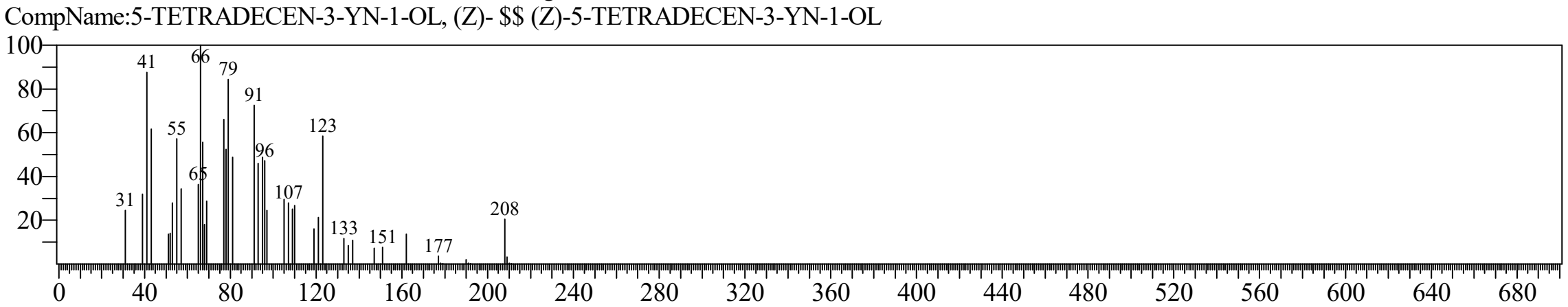

<< Target >>
Line#:124 R.Time:18.640(Scan#:3029) MassPeaks:104
RawMode:Averaged 18.635-18.645(3028-3030) BasePeak:91.05(16704)
BG Mode:Calc. from Peak Group 1 - Event 1 Scan

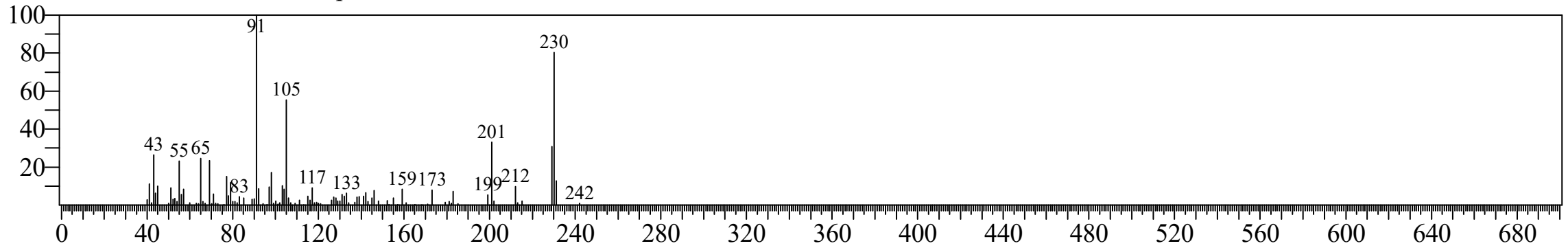

Hit#:1 Entry:147231 Library:WILEY8.LIB
SI:69 Formula:C14H18N2O CAS:0-00-0 MolWeight:230 RetIndex:0
CompName:3-PHENYL-PROPIONIC ACID CYCLOPENTYLIDENE-HYDRAZIDE $$ N'-CYCLOPENTYLIDENE-3-PHENYLPROPANOHYDRAZID

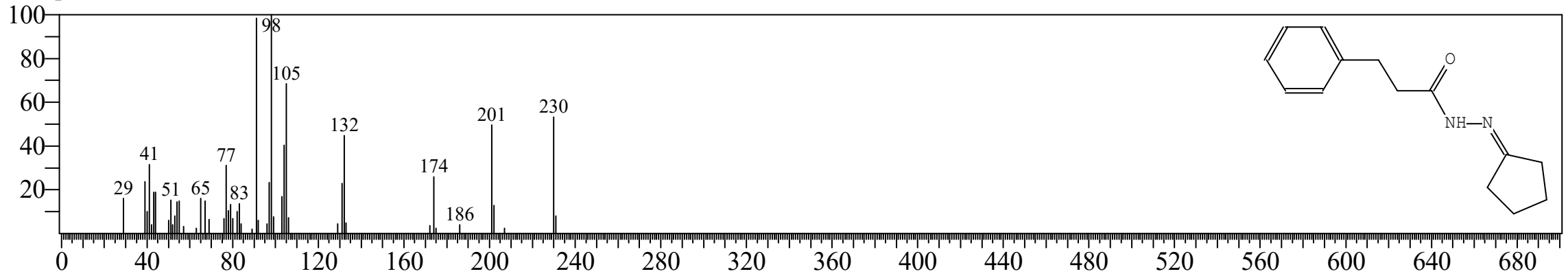

Hit#:2 Entry:314933 Library:WILEY8.LIB
SI:64 Formula:C13H12INO4 CAS:0-00-0 MolWeight:373 RetIndex:0
CompName:1-{[3-(2-IODOPHENYL)PROPANOYL]OXY}-2,5-PYRROLIDINEDIONE $$ 3-(2-IODO-PHENYL)-PROPIONIC ACID 2,5-DIOXO-PYRI

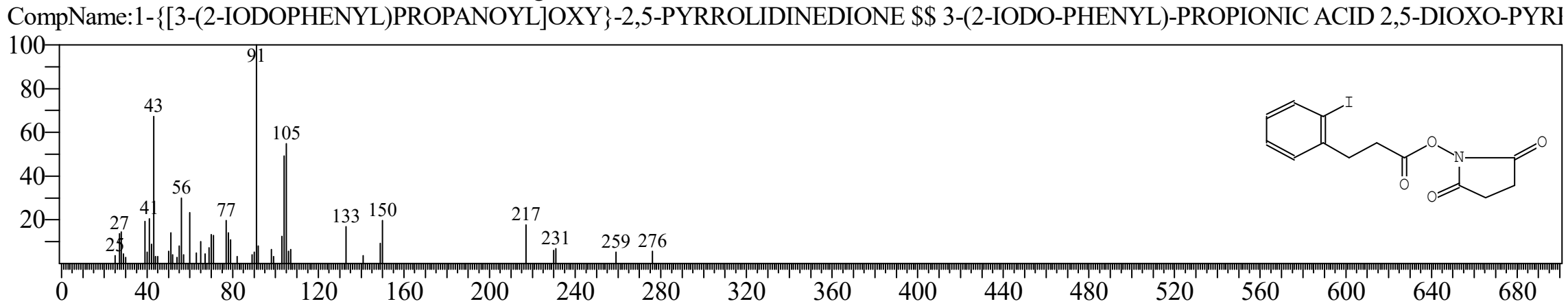

Hit#:3 Entry:190874 Library:NIST14.lib
SI:64 Formula:C13H12INO4 CAS:0-00-0 MolWeight:373 RetIndex:2535
CompName:3-(2-Iodo-phenyl)-propionic acid, 2,5-dioxo-pyrrolidin-1-yl ester $$ 1-([3-(2-Iodophenyl)propanoyl]oxy)-2,5-pyrrolidinedione # $$

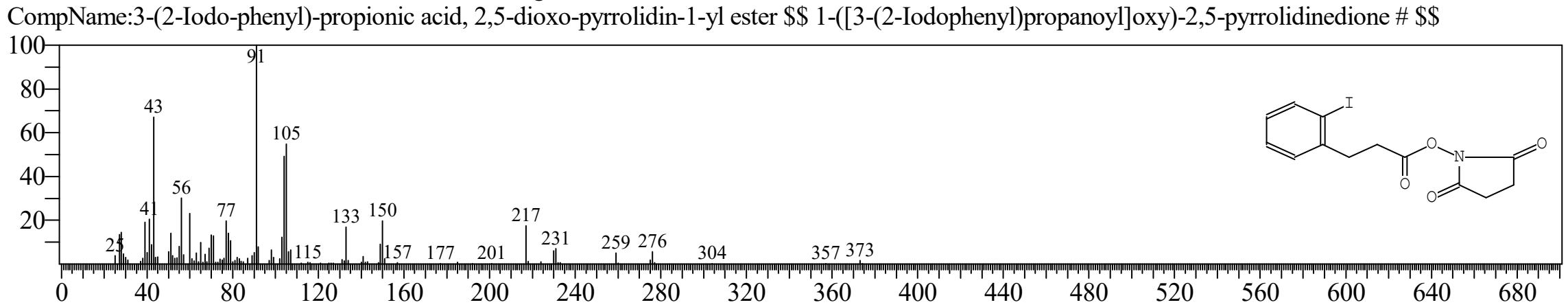

<< Target >>
Line#:125   R.Time:18.745(Scan#:3050) MassPeaks:90
RawMode:Averaged 18.740-18.750(3049-3051) BasePeak:91.05(7758)
BG Mode:Calc. from Peak Group 1 - Event 1 Scan

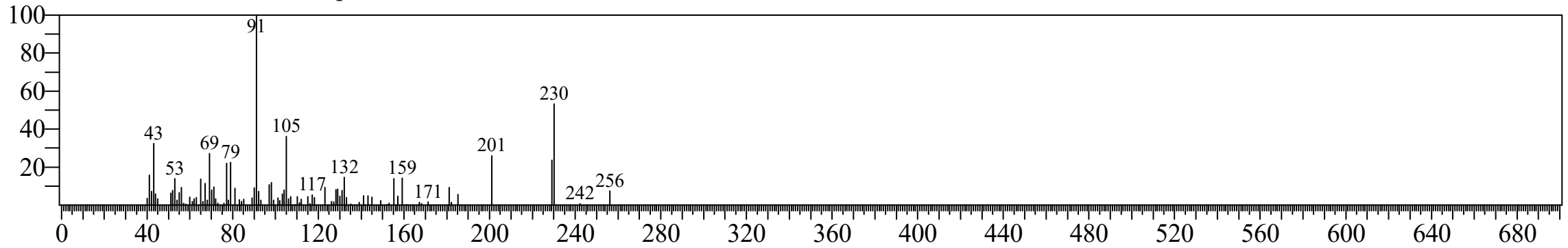

Hit#:1 Entry:147231 Library:WILEY8.LIB
SI:67 Formula:C14H18N2O CAS:0-00-0 MolWeight:230 RetIndex:0
CompName:3-PHENYL-PROPIONIC ACID CYCLOPENTYLIDENE-HYDRAZIDE $$ N'-CYCLOPENTYLIDENE-3-PHENYLPROPANOHYDRAZID

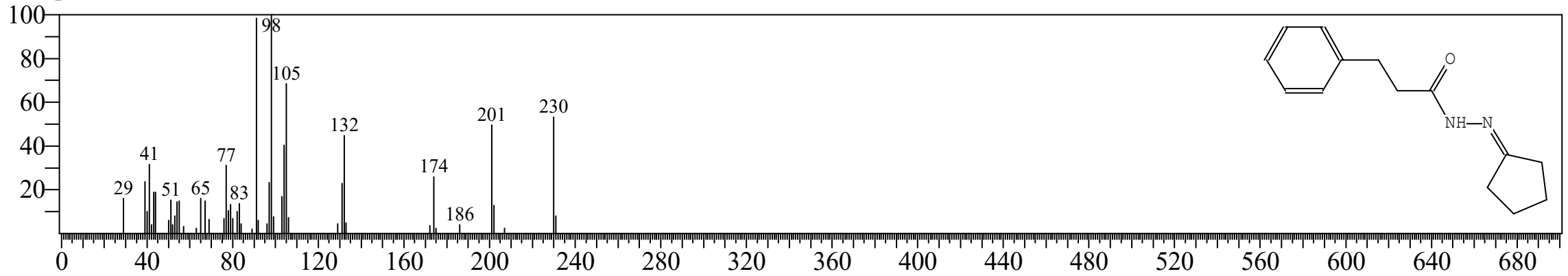

Hit#:2 Entry:185555 Library:WILEY8.LIB
SI:64 Formula:C16H22N2O CAS:339320-81-9 MolWeight:258 RetIndex:0
CompName:N'-[(1Z)-2-ETHYLCYCLOPENTYLIDENE]-3-PHENYLPROPANOHYDRAZIDE # $$ N'-[(1Z)-2-ETHYLCYCLOPENTYLIDENE]-3-PHE

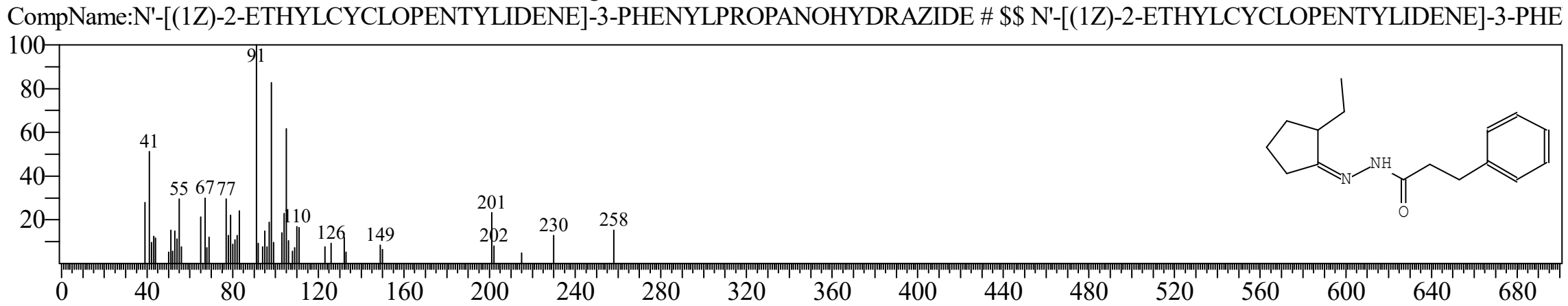

Hit#:3 Entry:94207 Library:NIST14.lib
SI:64 Formula:C16H22N2O CAS:339320-81-9 MolWeight:258 RetIndex:2324
CompName:Propanehydrazide, 3-phenyl-N2-(2-ethylcyclopentylideno)- $$ N'-[(1Z)-2-Ethylcyclopentylidene]-3-phenylpropanohydrazide # $$

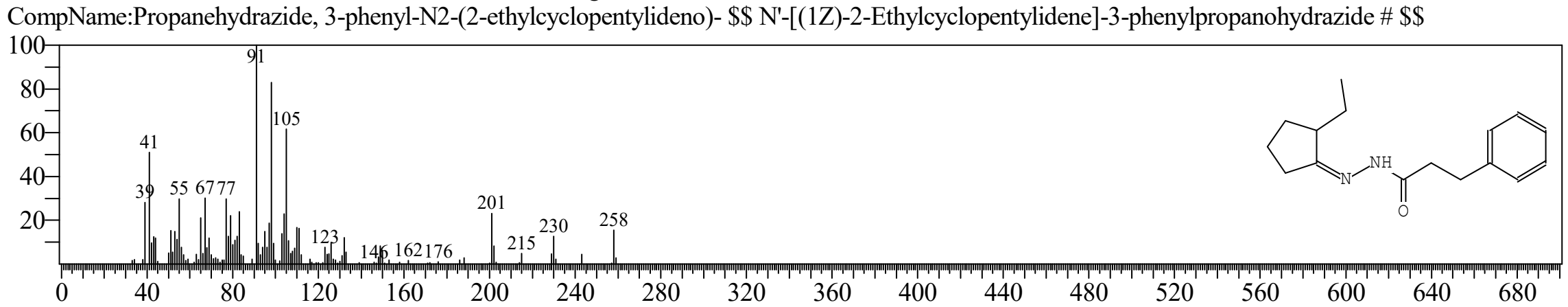

<< Target >>
Line#:126 R.Time:18.930(Scan#:3087) MassPeaks:71
RawMode:Averaged 18.925-18.935(3086-3088) BasePeak:55.05(13324)
BG Mode:Calc. from Peak Group 1 - Event 1 Scan

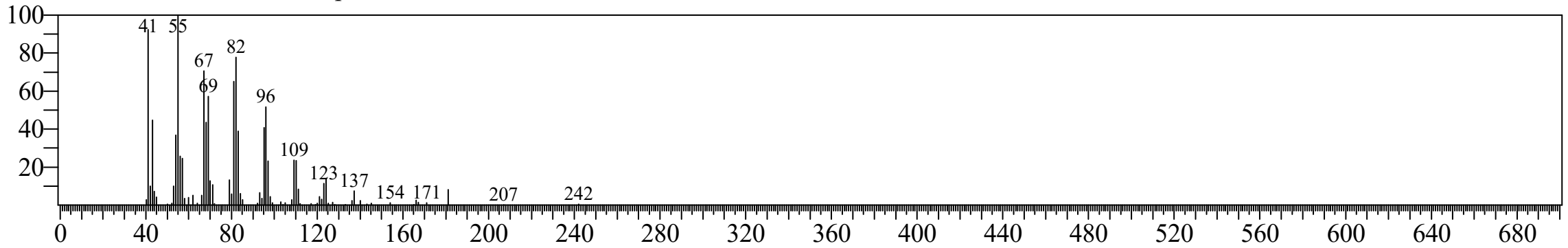

Hit#:1 Entry:199389 Library:WILEY8.LIB
SI:94 Formula:C18H36O CAS:143-28-2 MolWeight:268 RetIndex:0
CompName:9-OCTADECEN-1-OL, (Z)- $$ OCTADEC-9-EN-1-OL $$ (9Z)-9-OCTADECEN-1-OL $$ (Z)-9-OCTADECEN-1-OL $$ (Z)-9-OCTADECEN

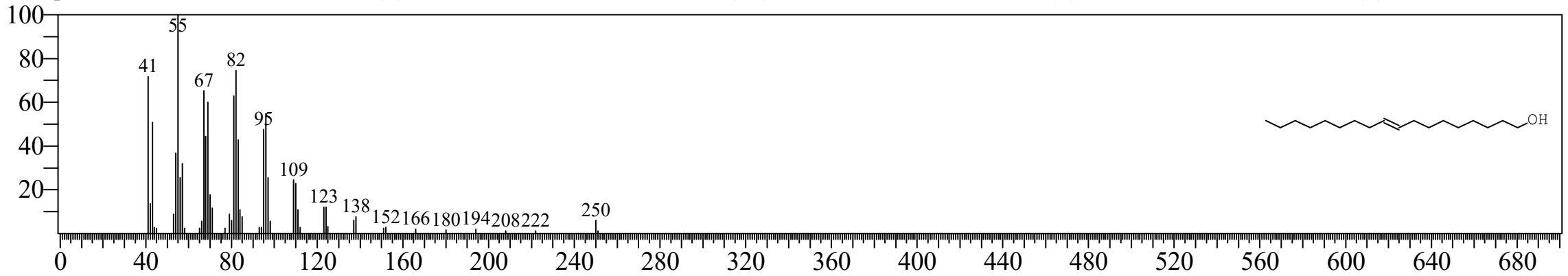

Hit#:2 Entry:17455 Library:NIST14s.lib
SI:93 Formula:C14H26 CAS:21964-49-8 MolWeight:194 RetIndex:1393
CompName:1,13-Tetradecadiene $$ Tetradeca-1,13-diene $$ Tetradecadiene-1,13 $$

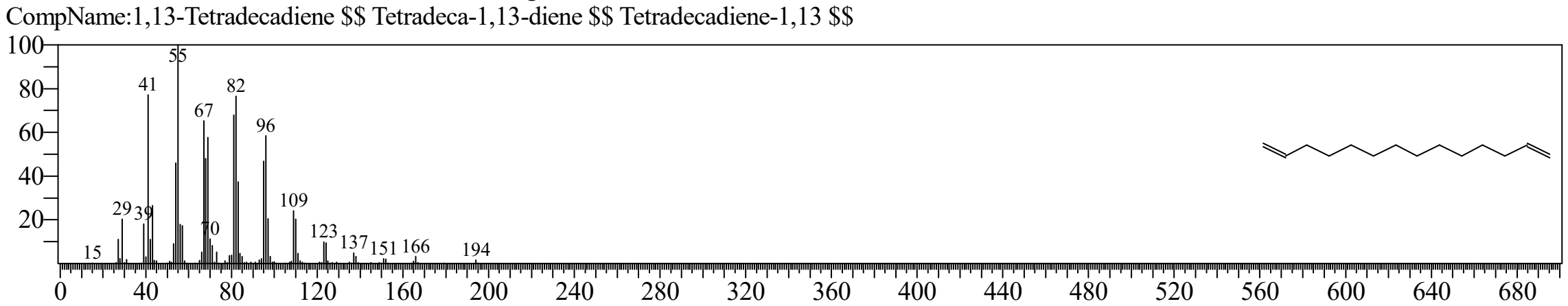

Hit#:3 Entry:127768 Library:NIST14.lib
SI:93 Formula:C20H40O CAS:112248-30-3 MolWeight:296 RetIndex:2260
CompName:Eicosen-1-ol, cis-9- $$ (9Z)-9-Icosen-1-ol # $$

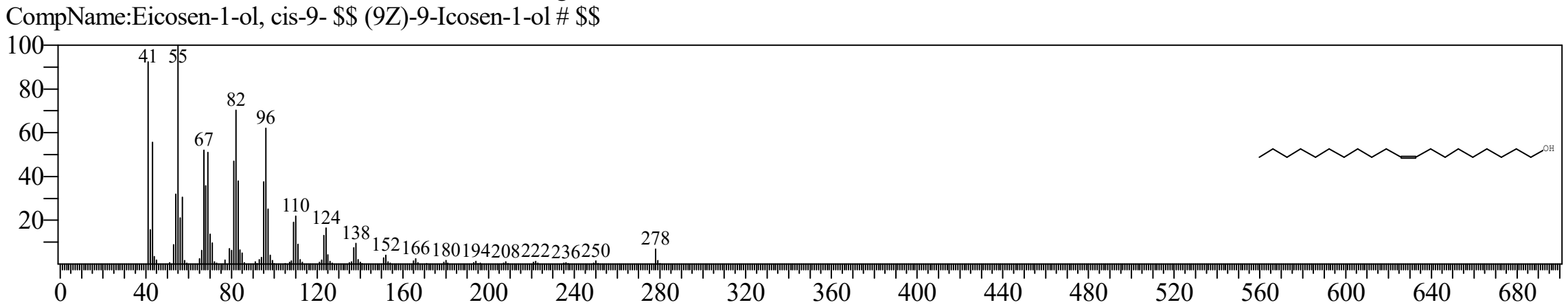

<< Target >>
Line#:127   R.Time:19.020(Scan#:3105) MassPeaks:82
RawMode:Averaged 19.015-19.025(3104-3106) BasePeak:57.05(24140)
BG Mode:Calc. from Peak Group 1 - Event 1 Scan

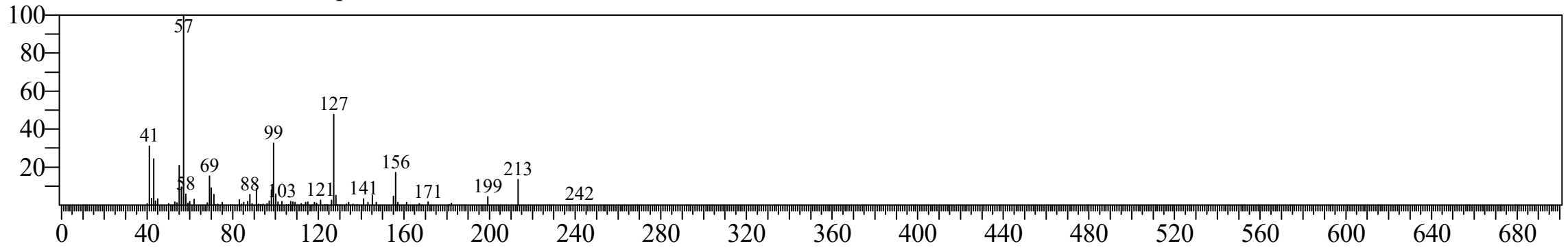

Hit#:1 Entry:164171 Library:WILEY8.LIB
SI:78 Formula:C15H30O2 CAS:0-00-0 MolWeight:242 RetIndex:0
CompName:3-HEPTYL 2-ETHYLHEXANOATE

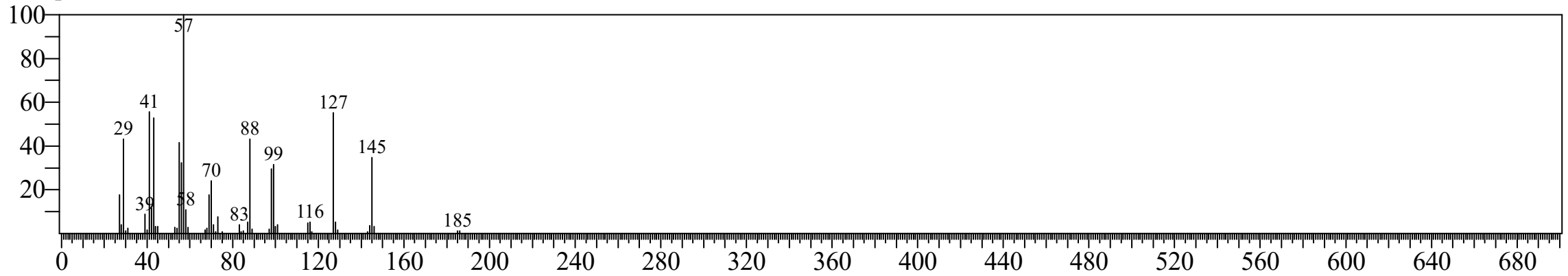

Hit#:2 Entry:67795 Library:NIST14.lib
SI:78 Formula:C15H30O CAS:0-00-0 MolWeight:226 RetIndex:1520
CompName:4,6-Dipropyl-nonan-5-one $$ 4,6-Dipropyl-5-nonanone # $$

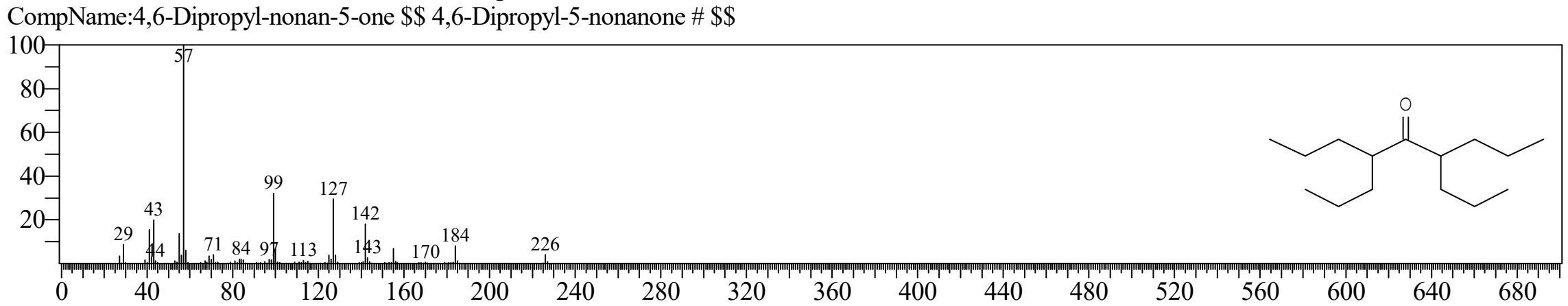

Hit#:3 Entry:142271 Library:WILEY8.LIB
SI:78 Formula:C15H30O CAS:0-00-0 MolWeight:226 RetIndex:0
CompName:4,6-DIPROPYL-5-NONANONE $$ 4,6-DIPROPYL-NONAN-5-ONE

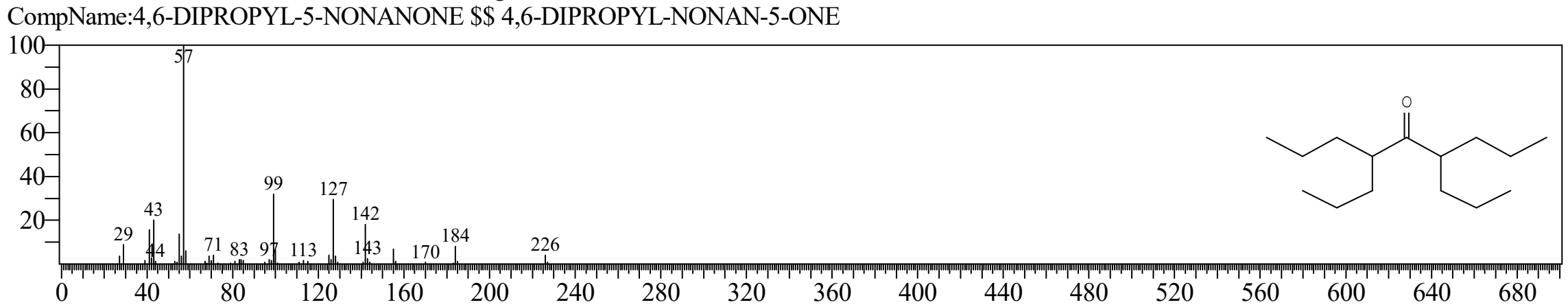

<< Target >>
Line#:128   R.Time:19.135(Scan#:3128) MassPeaks:105
RawMode:Averaged 19.130-19.140(3127-3129) BasePeak:43.05(39071)
BG Mode:Calc. from Peak Group 1 - Event 1 Scan

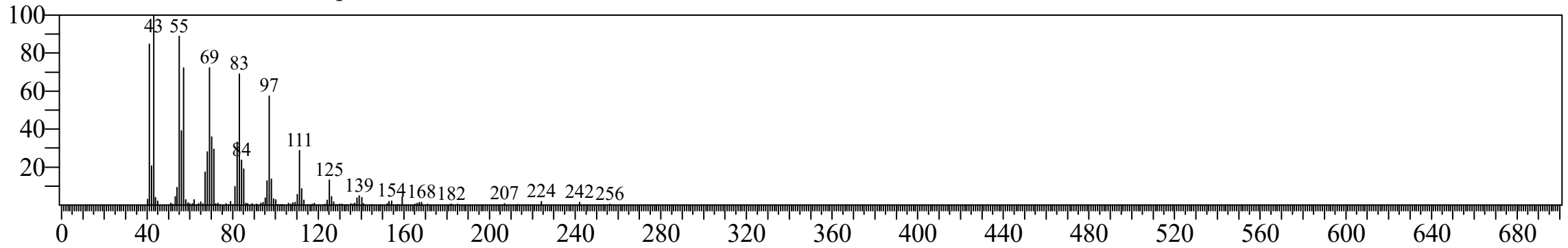

Hit#:1 Entry:28281 Library:NIST14s.lib
SI:96 Formula:C20H42O CAS:629-96-9 MolWeight:298 RetIndex:2252
CompName:1-Eicosanol $$ n-Eicosanol $$ n-1-Eicosanol $$ Arachic alcohol $$ Arachidic alcohol $$ Arachidyl alcohol $$ Eicosyl alcohol $$ 1-Icosanol $$

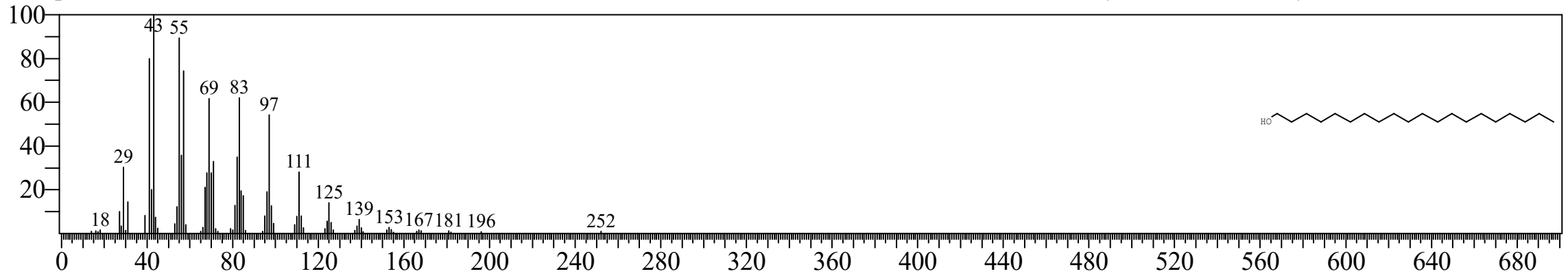

Hit#:2 Entry:79083 Library:NIST14.lib
SI:96 Formula:C16H32O CAS:64437-47-4 MolWeight:240 RetIndex:1862
CompName:Hexadecen-1-ol, trans-9- $$ (9E)-9-Hexadecen-1-ol $$ 9-Hexadecenol, E $$ (E)9-Hexadecen-1-ol $$

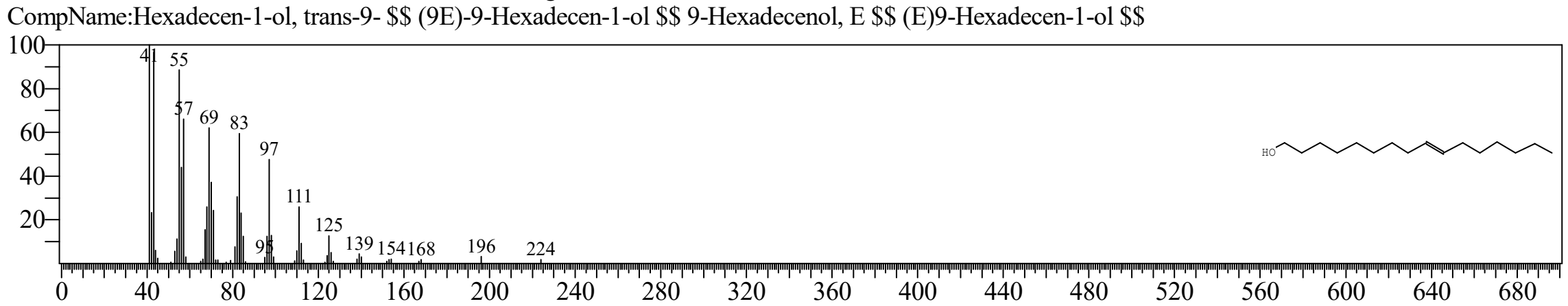

Hit#:3 Entry:89019 Library:NIST14.lib
SI:96 Formula:C17H32O CAS:0-00-0 MolWeight:252 RetIndex:1907
CompName:E-15-Heptadecenal $$ (15E)-15-Heptadecenal # $$

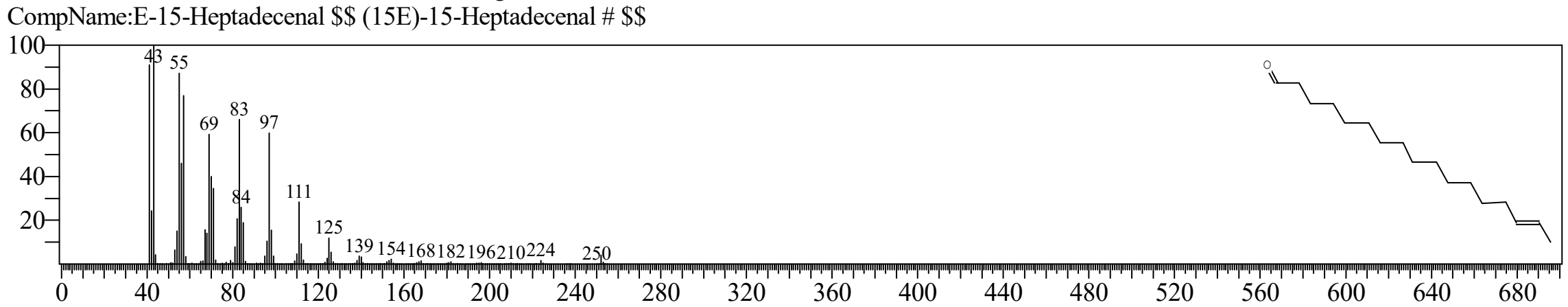

<< Target >>
Line#:129   R.Time:19.235(Scan#:3148) MassPeaks:88
RawMode:Averaged 19.230-19.240(3147-3149) BasePeak:41.05(3278)
BG Mode:Calc. from Peak Group 1 - Event 1 Scan

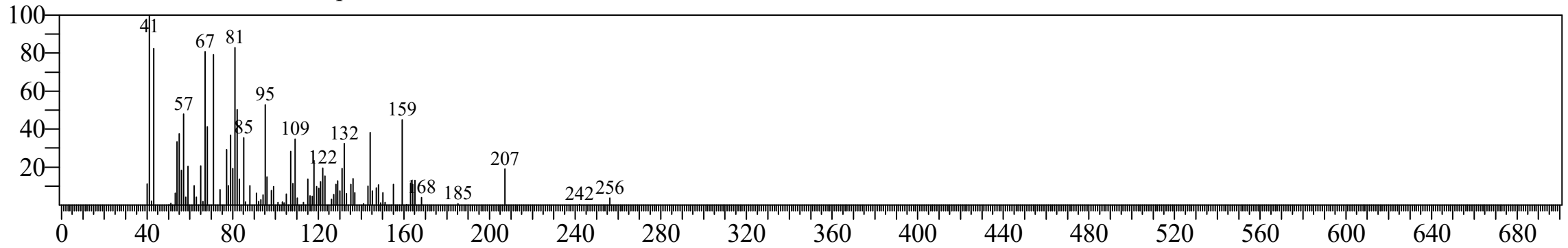

Hit#:1 Entry:232796 Library:WILEY8.LIB
SI:69 Formula:C19H34O2 CAS:1120-32-7 MolWeight:294 RetIndex:0
CompName:9-OCTADECYNOIC ACID, METHYL ESTER $$ METHYL OCTADEC-9-YNOATE $$ METHYL 9-OCTADECYNOATE # $$ METHYL 9

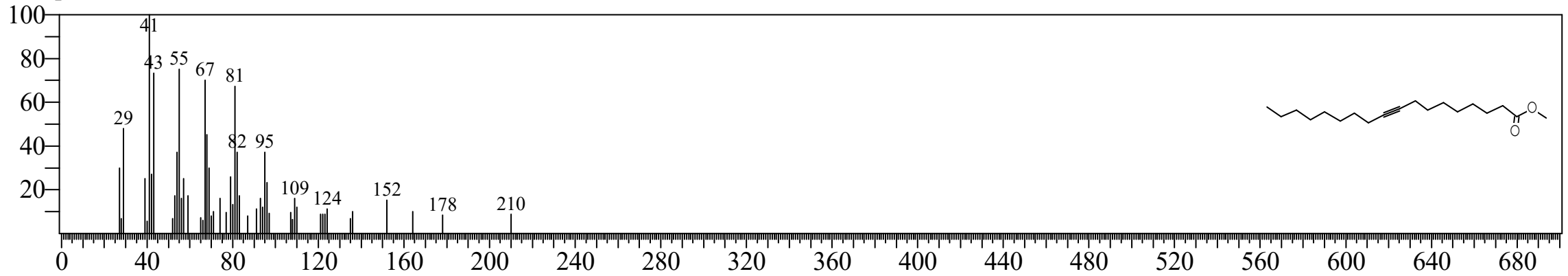

Hit#:2 Entry:27993 Library:NIST14s.lib
SI:69 Formula:C19H34O2 CAS:1120-32-7 MolWeight:294 RetIndex:2095
CompName:9-Octadecynoic acid, methyl ester $$ Methyl stearolate $$ Methyl 9-octadecynoate # $$

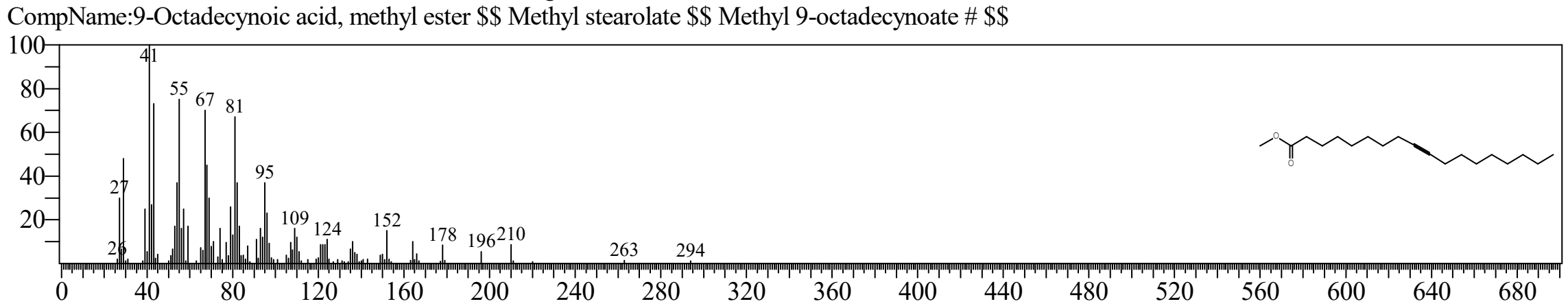

Hit#:3 Entry:279456 Library:WILEY8.LIB
SI:68 Formula:C22H38O2 CAS:10152-71-3 MolWeight:334 RetIndex:0
CompName:CYCLOPROPANEOCTANOIC ACID, 2-[[2-[(2-ETHYLCYCLOPROPYL)METHYL]CYCLOPROPYL]METHYL]-, METHYL ESTER $$ M

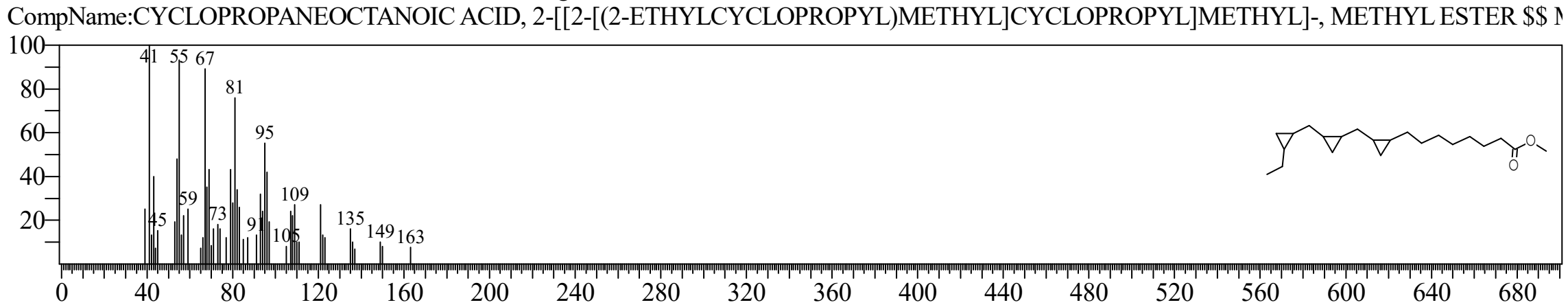

<< Target >>
Line#:130   R.Time:19.280(Scan#:3157) MassPeaks:95
RawMode:Averaged 19.275-19.285(3156-3158) BasePeak:55.05(3328)
BG Mode:Calc. from Peak Group 1 - Event 1 Scan

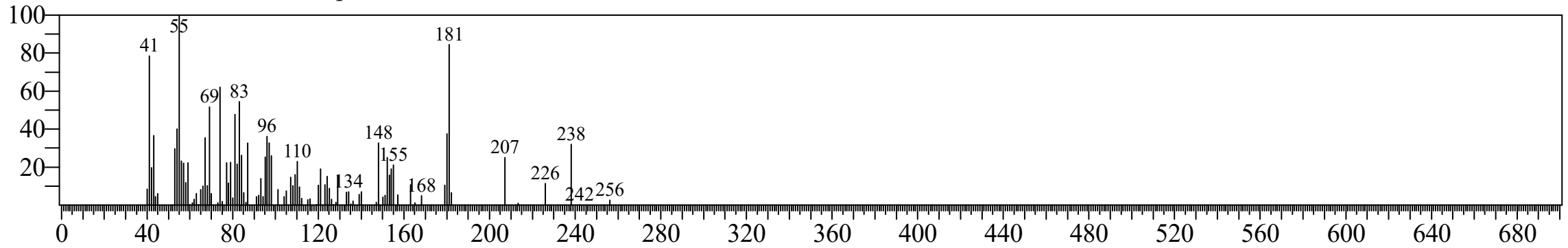

Hit#:1 Entry:28136 Library:NIST14s.lib
SI:74 Formula:C19H36O2 CAS:112-62-9 MolWeight:296 RetIndex:2085
CompName:9-Octadecenoic acid (Z)-, methyl ester $$ Oleic acid, methyl ester $$ Emery oleic acid ester 2301 $$ Methyl cis-9-octadecenoate $$ Methyl ole

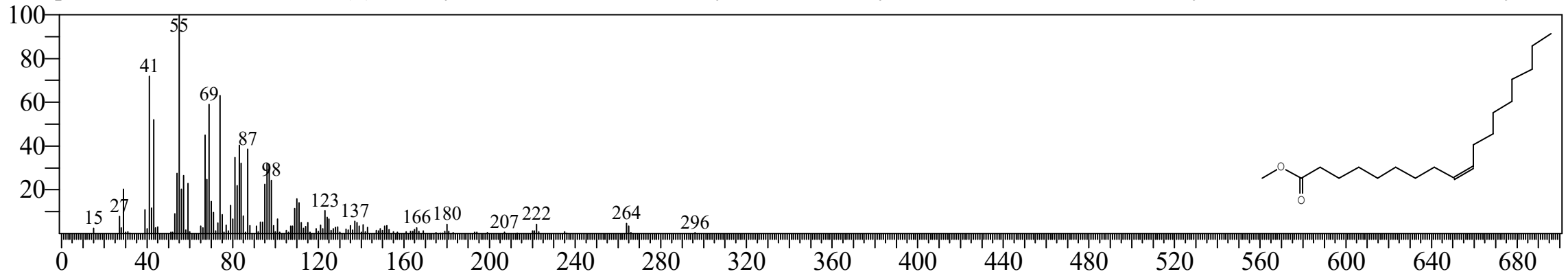

Hit#:2 Entry:235422 Library:WILEY8.LIB
SI:74 Formula:C19H36O2 CAS:112-62-9 MolWeight:296 RetIndex:0
CompName:9-OCTADECENOIC ACID (Z)-, METHYL ESTER $$ 9-OCTADECENOIC ACID, METHYL ESTER $$ METHYL (9Z)-9-OCTADECENO

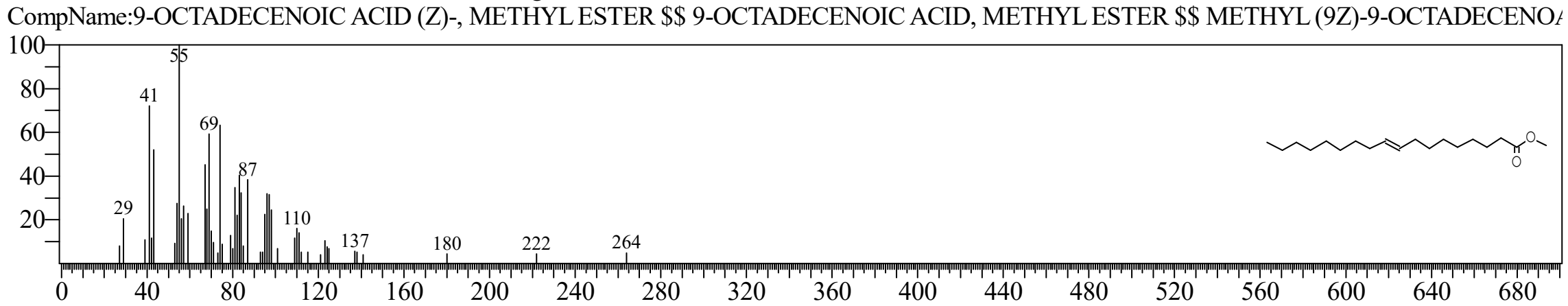

Hit#:3 Entry:199152 Library:WILEY8.LIB
SI:73 Formula:C17H32O2 CAS:1120-25-8 MolWeight:268 RetIndex:0
CompName:9-HEXADECENOIC ACID, METHYL ESTER, (Z)- $$ METHYL (9Z)-9-HEXADECENOATE # $$ AI3-36450 $$ EINECS 214-303-2 $$ M

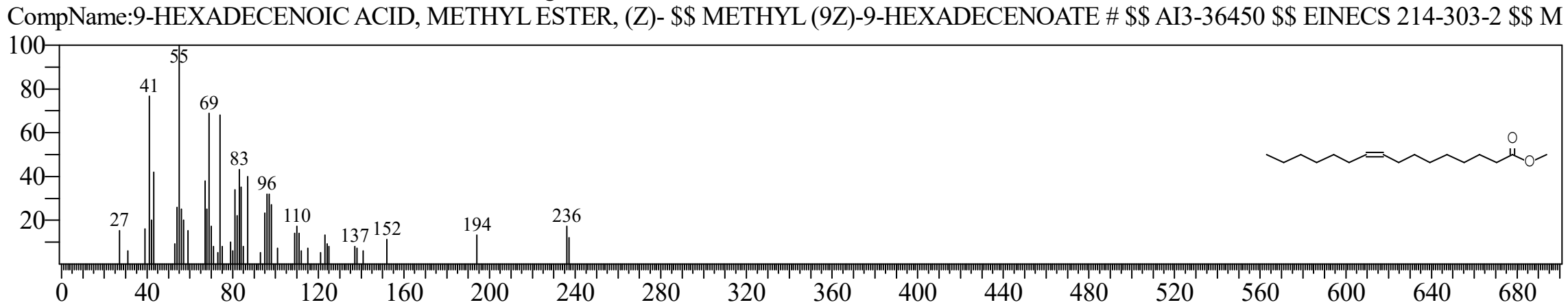

<< Target >>
Line#:131   R.Time:19.430(Scan#:3187) MassPeaks:193
RawMode:Averaged 19.425-19.435(3186-3188) BasePeak:71.05(5966870)
BG Mode:Calc. from Peak Group 1 - Event 1 Scan

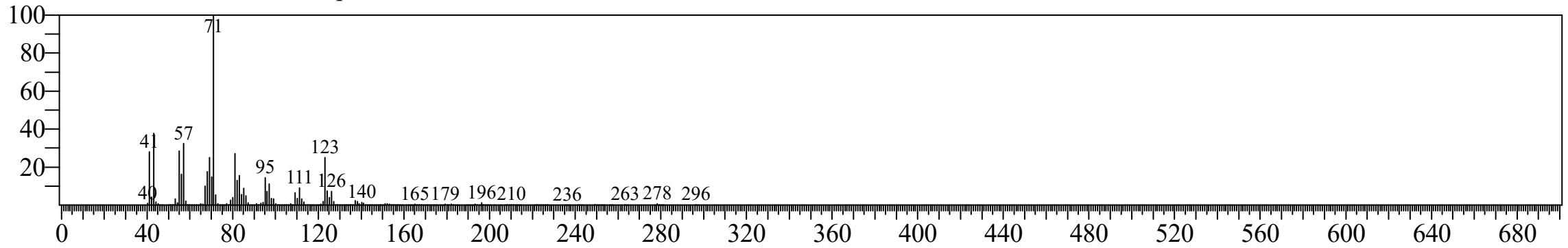

Hit#:1 Entry:127772 Library:NIST14.lib
SI:98 Formula:C20H40O CAS:150-86-7 MolWeight:296 RetIndex:2045
CompName:Phytol $$ 2-Hexadecen-1-ol, 3,7,11,15-tetramethyl-, [R-[R*,R*-(E)]]- $$ trans-Phytol $$ 3,7,11,15-Tetramethyl-2-hexadecen-1-ol-, (2E,7R,11R

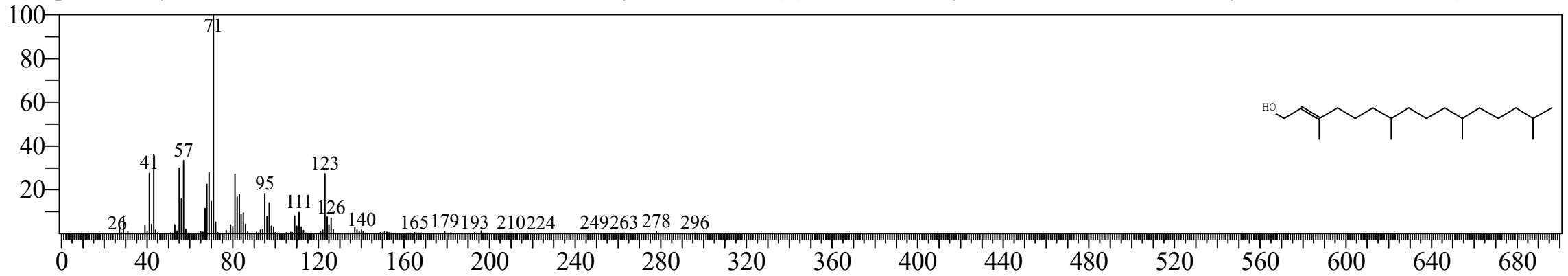

Hit#:2 Entry:28151 Library:NIST14s.lib
SI:97 Formula:C20H40O CAS:150-86-7 MolWeight:296 RetIndex:2045
CompName:Phytol $$ 2-Hexadecen-1-ol, 3,7,11,15-tetramethyl-, [R-[R*,R*-(E)]]- $$ trans-Phytol $$ 3,7,11,15-Tetramethyl-2-hexadecen-1-ol-, (2E,7R,11R

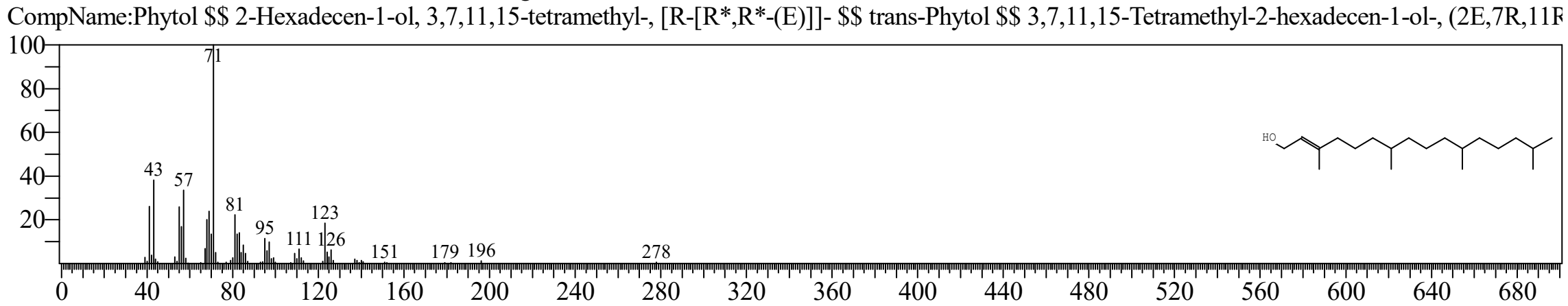

Hit#:3 Entry:235594 Library:WILEY8.LIB
SI:97 Formula:C20H40O CAS:150-86-7 MolWeight:296 RetIndex:0
CompName:2-HEXADECEN-1-OL, 3,7,11,15-TETRAMETHYL-, [R-[R*,R*-(E)]]- $$ 3,7,11,15-TETRAMETHYLHEXADEC-2-EN-1-OL $$ (2E)-3,7,1

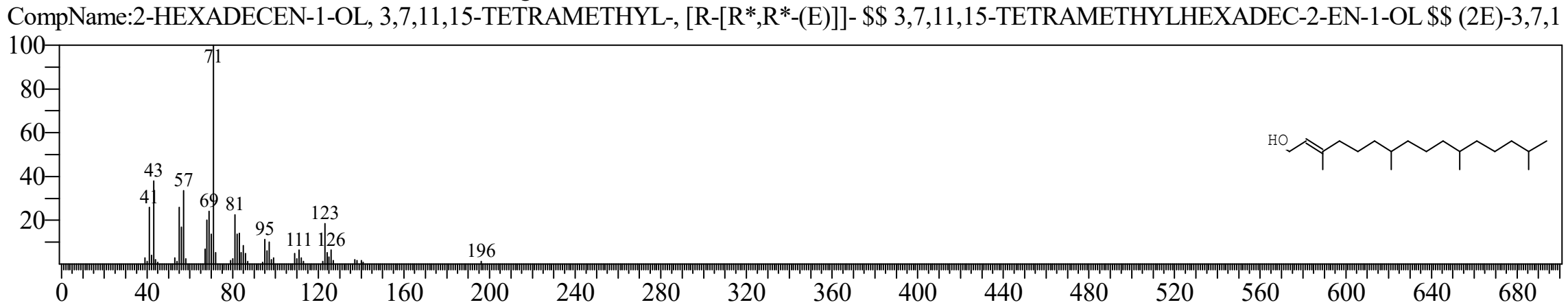

<< Target >>
Line#:132 R.Time:19.710(Scan#:3243) MassPeaks:111
RawMode:Averaged 19.705-19.715(3242-3244) BasePeak:84.05(14171)
BG Mode:Calc. from Peak Group 1 - Event 1 Scan

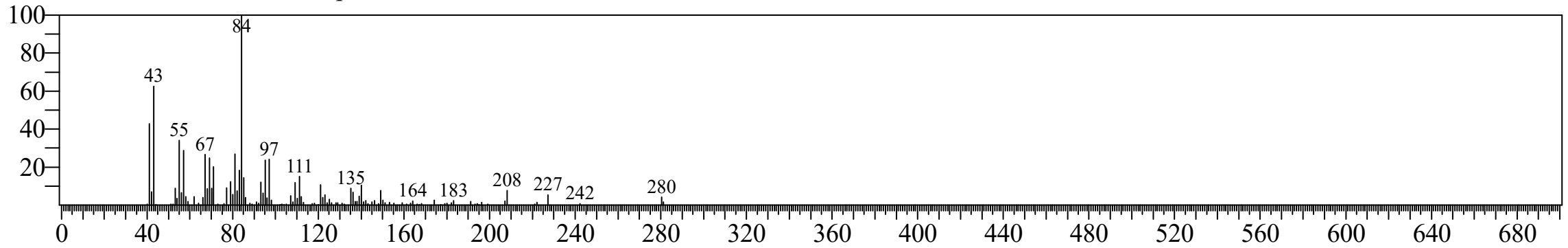

Hit#:1 Entry:283389 Library:WILEY8.LIB
SI:82 Formula:C22H42O2 CAS:18302-67-5 MolWeight:338 RetIndex:0
CompName:PALMITALDEHYDE, DIALLYL ACETAL $$ 1,1-DIPROP-2-ENOXY HEXADECANE $$ HEXADECANAL DIALLYL ACETAL

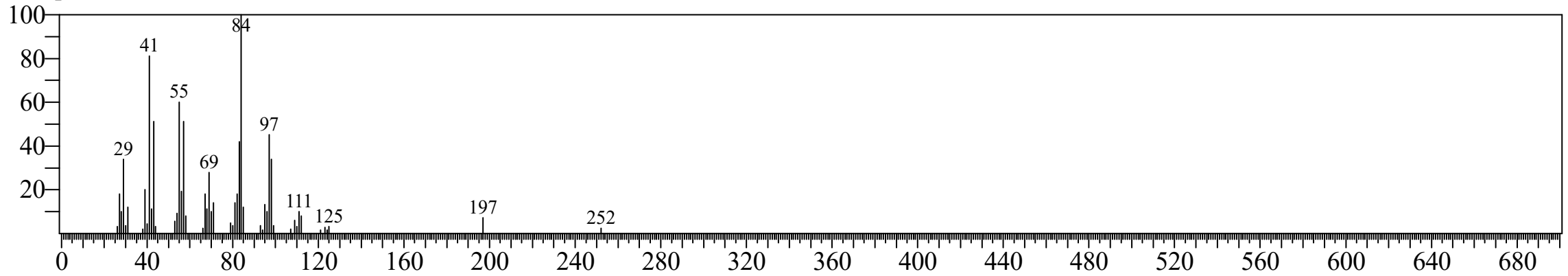

Hit#:2 Entry:46831 Library:WILEY8.LIB
SI:82 Formula:C10H18O CAS:4884-28-0 MolWeight:154 RetIndex:0
CompName:CYCLOPENTANOL, 1-CYCLOPENTYL- $$ [1,1'-BICYCLOPENTYL]-1-OL $$ 1-CYCLOPENTYLCYCLOPENTANOL $$ CYCLOPENT

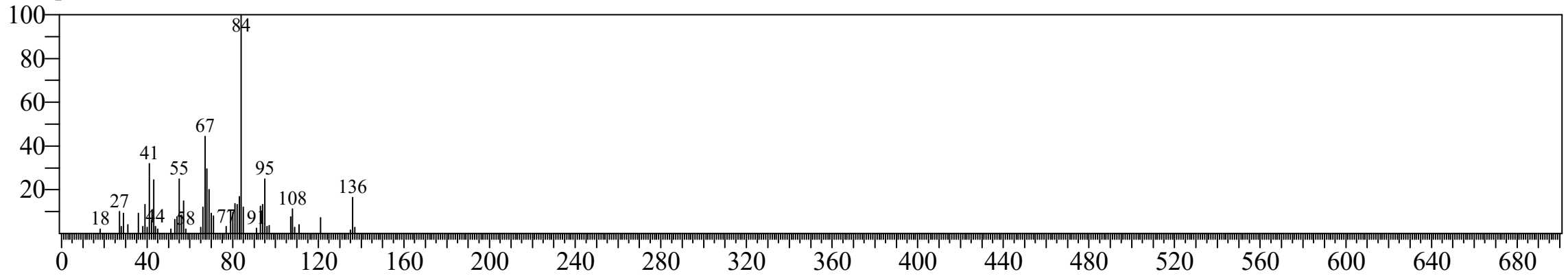

Hit#:3 Entry:102612 Library:NIST14.lib
SI:80 Formula:C16H28O3 CAS:0-00-0 MolWeight:268 RetIndex:1877
CompName:Z-(13,14-Epoxy)tetradec-11-en-1-ol acetate $$ (11Z)-12-(2-Oxiranyl)-11-dodecenyl acetate # $$

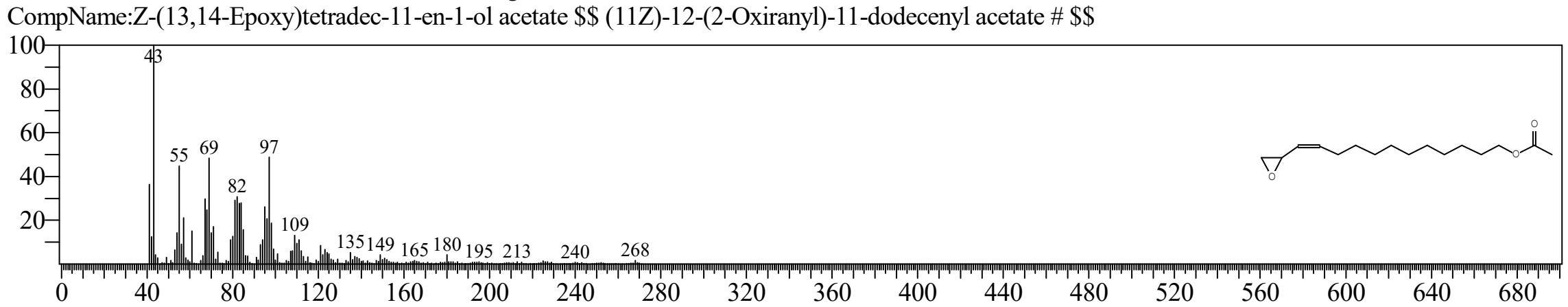

<< Target >>
Line#:133 R.Time:19.745(Scan#:3250) MassPeaks:127
RawMode:Averaged 19.740-19.750(3249-3251) BasePeak:55.05(16806)
BG Mode:Calc. from Peak Group 1 - Event 1 Scan

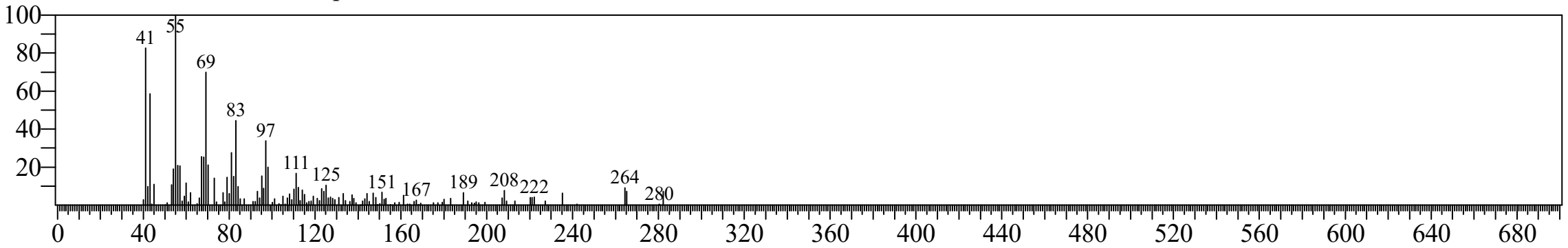

Hit#:1 Entry:217534 Library:WILEY8.LIB
SI:87 Formula:C18H34O2 CAS:112-80-1 MolWeight:282 RetIndex:0
CompName:9-OCTADECENOIC ACID (Z)- $$ OCTADEC-9-ENOIC ACID $$ (9E)-9-OCTADECENOIC ACID # $$ (9E)-9-OCTADECENOIC ACID (C

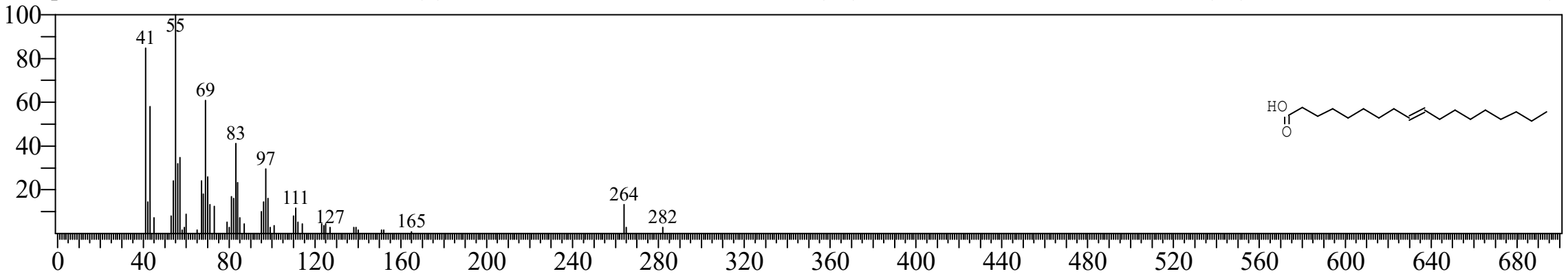

Hit#:2 Entry:115090 Library:NIST14.lib
SI:86 Formula:C18H34O2 CAS:506-17-2 MolWeight:282 RetIndex:2175
CompName:cis-Vaccenic acid $$ 11-Octadecenoic acid, (Z)- $$ (Z)-11-Octadecenoic acid $$

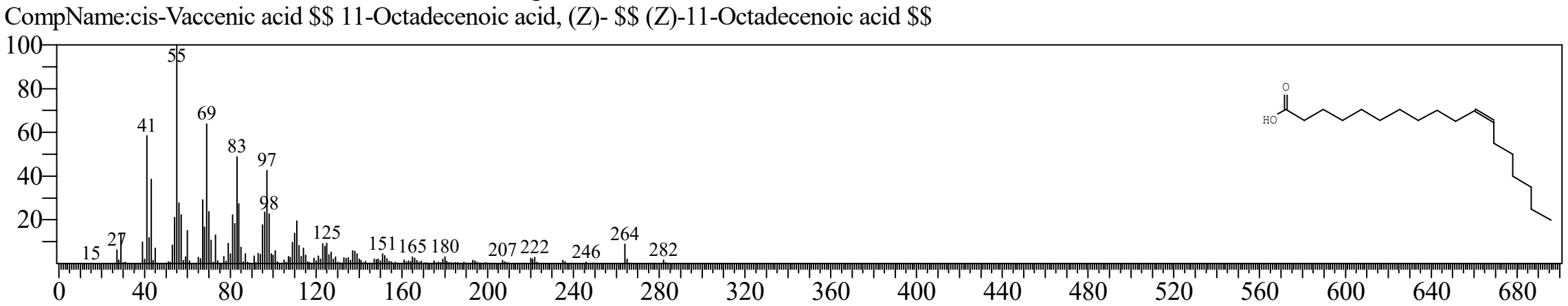

Hit#:3 Entry:217541 Library:WILEY8.LIB
SI:86 Formula:C18H34O2 CAS:112-80-1 MolWeight:282 RetIndex:0
CompName:9-OCTADECENOIC ACID (Z)- $$ OCTADEC-9-ENOIC ACID $$ (9E)-9-OCTADECENOIC ACID # $$ (9E)-9-OCTADECENOIC ACID (C

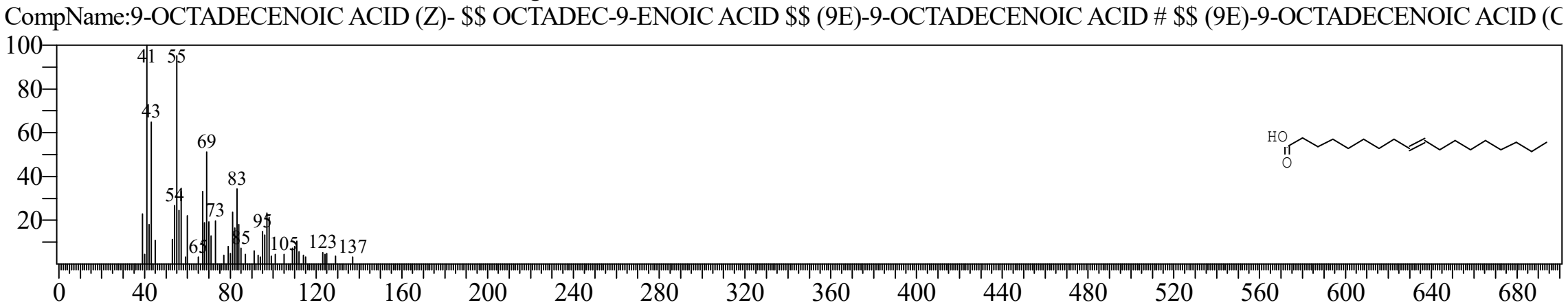

<< Target >>
Line#:134 R.Time:19.905(Scan#:3282) MassPeaks:100
RawMode:Averaged 19.900-19.910(3281-3283) BasePeak:55.05(7241)
BG Mode:Calc. from Peak Group 1 - Event 1 Scan

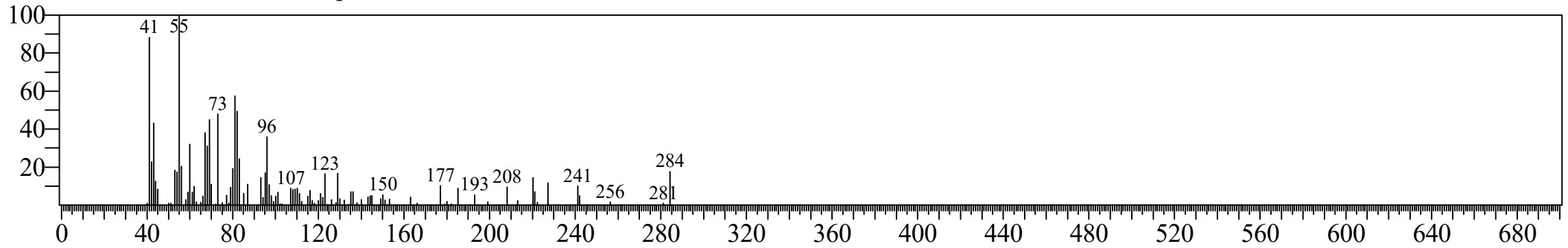

Hit#:1 Entry:26959 Library:NIST14s.lib
SI:79 Formula:C18H32O2 CAS:60-33-3 MolWeight:280 RetIndex:2183
CompName:9,12-Octadecadienoic acid (Z,Z)- $$ cis-9,cis-12-Octadecadienoic acid $$ cis,cis-Linoleic acid $$ Grape seed oil $$ Linoleic $$ Linoleic acid $

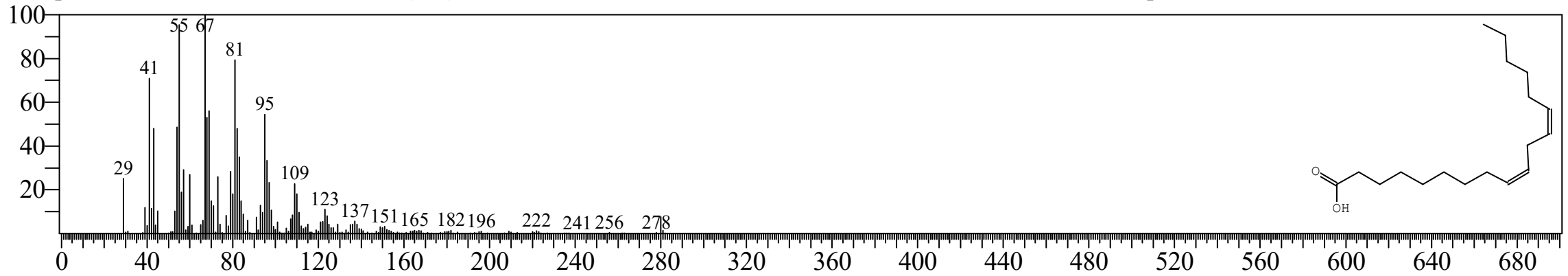

Hit#:2 Entry:113241 Library:NIST14.lib
SI:79 Formula:C18H32O2 CAS:34450-18-5 MolWeight:280 RetIndex:2165
CompName:17-Octadecynoic acid

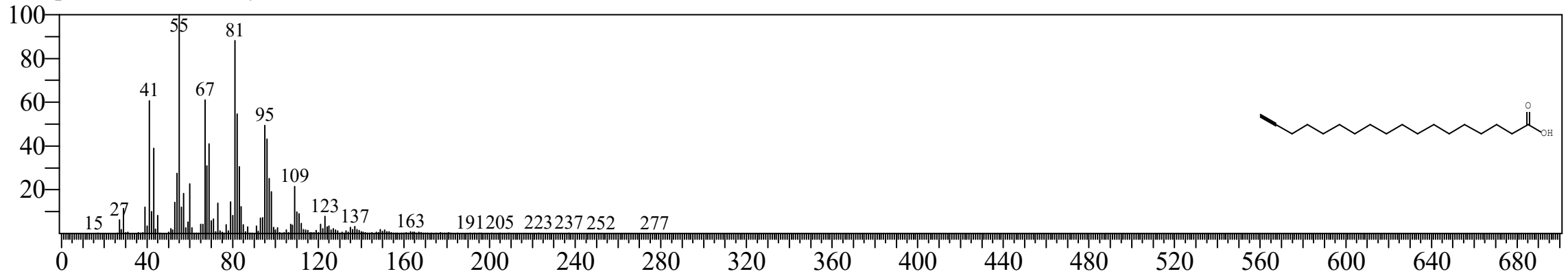

Hit#:3 Entry:214811 Library:WILEY8.LIB
SI:78 Formula:C18H32O2 CAS:60-33-3 MolWeight:280 RetIndex:0
CompName:9,12-OCTADECADIENOIC ACID (Z,Z)- $$ (9E,12E)-9,12-OCTADECADIENOIC ACID # $$ (9E,12E)-9,12-OCTADECADIENOIC ACID

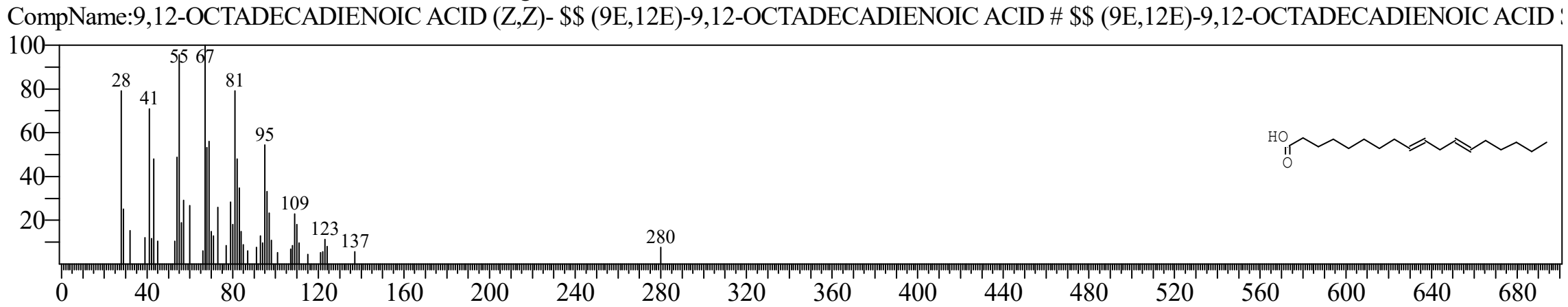

<< Target >>
Line#:135 R.Time:20.080(Scan#:3317) MassPeaks:152
RawMode:Averaged 20.075-20.085(3316-3318) BasePeak:43.05(132712)
BG Mode:Calc. from Peak Group 1 - Event 1 Scan

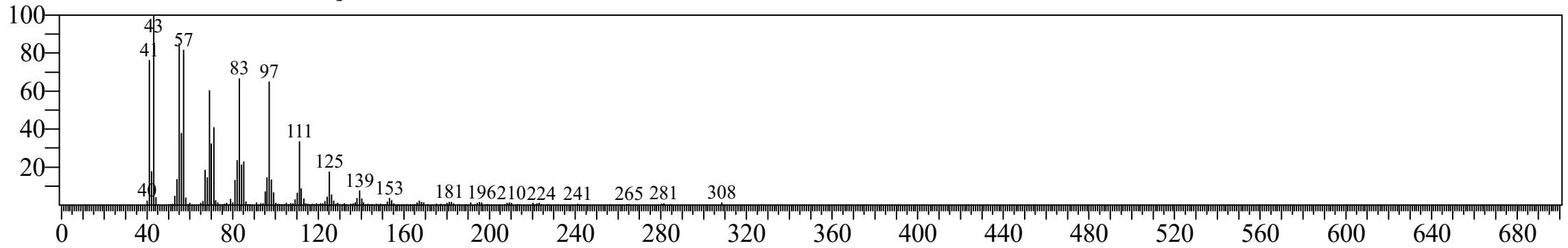

Hit#:1 Entry:25913 Library:NIST14s.lib
SI:97 Formula:C19H38 CAS:18435-45-5 MolWeight:266 RetIndex:1900
CompName:1-Nonadecene $$ Nonadec-1-ene $$

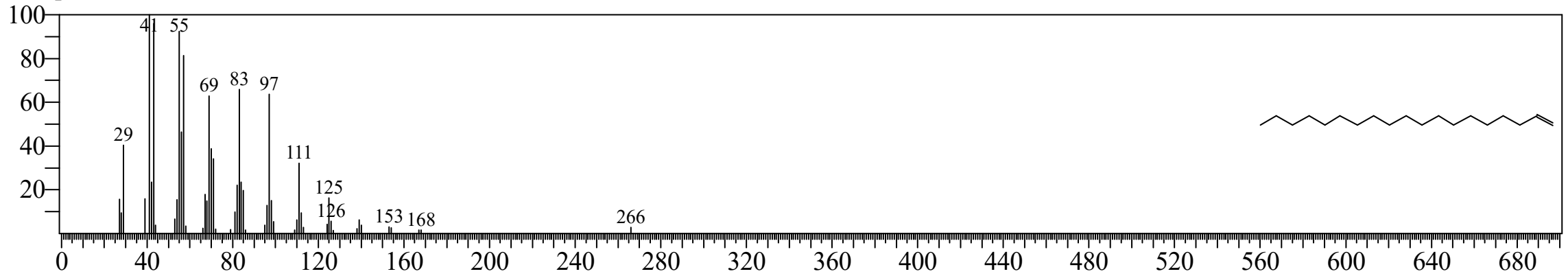

Hit#:2 Entry:28881 Library:NIST14s.lib
SI:97 Formula:C22H44 CAS:1599-67-3 MolWeight:308 RetIndex:2198
CompName:1-Docosene $$ Docos-1-ene $$

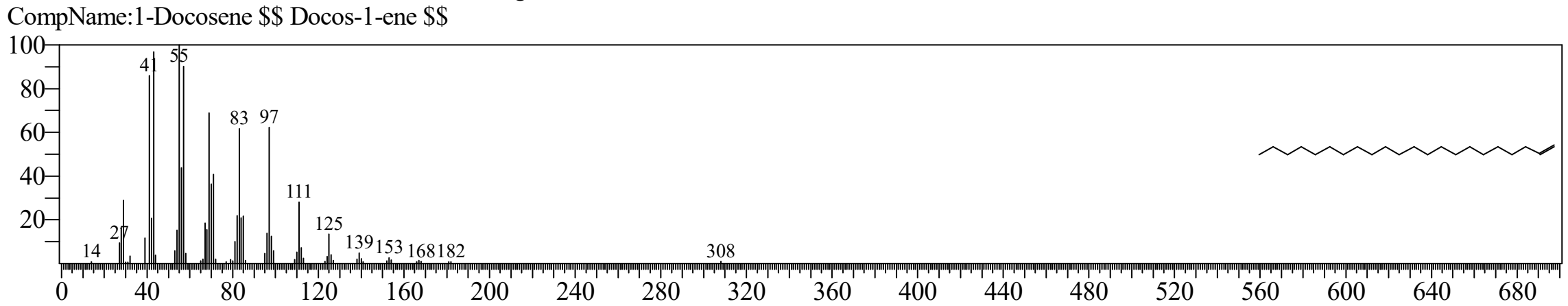

Hit#:3 Entry:270849 Library:WILEY8.LIB
SI:96 Formula:C22H46O CAS:661-19-8 MolWeight:326 RetIndex:0
CompName:1-DOCOSANOL $$ DOCOSAN-1-OL $$ 1-DOCOSANO $$ ABREVA $$ AI3-36489 $$ AIDS-042803 $$ BEHENIC ALCOHOL $$ BEHEN

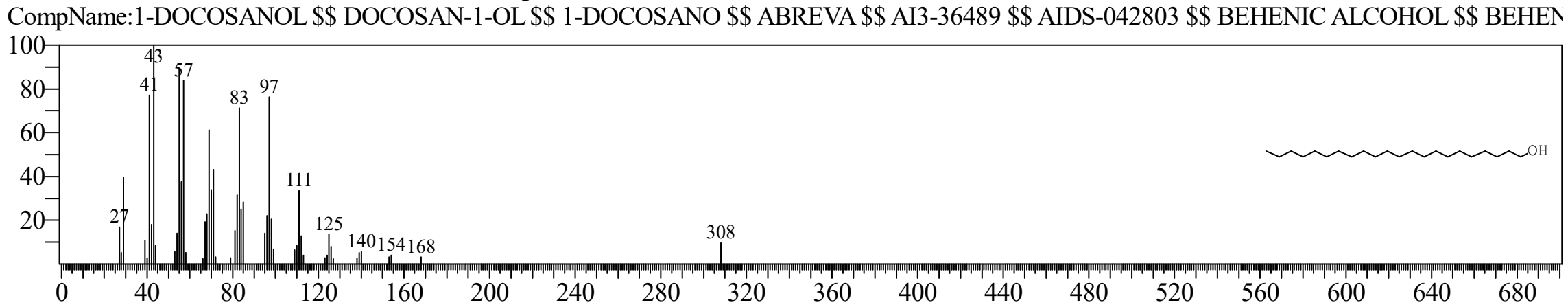

<< Target >>
Line#:136 R.Time:20.235(Scan#:3348) MassPeaks:97
RawMode:Averaged 20.230-20.240(3347-3349) BasePeak:43.05(6480)
BG Mode:Calc. from Peak Group 1 - Event 1 Scan

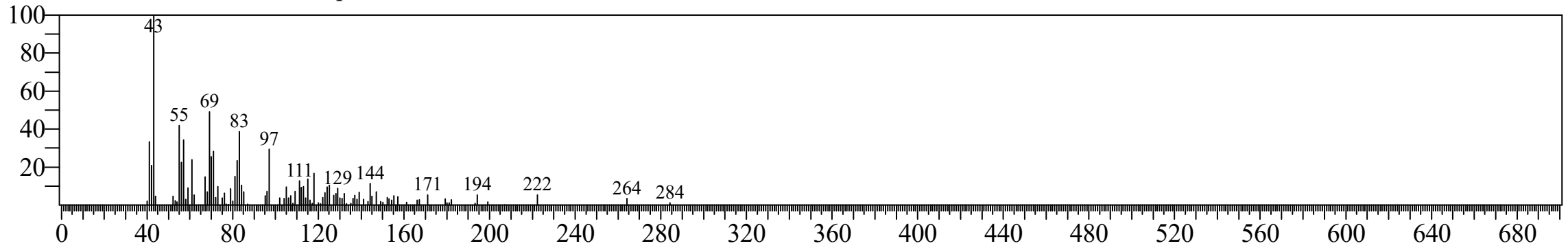

Hit#:1 Entry:129668 Library:NIST14.lib
SI:80 Formula:C19H38O2 CAS:822-20-8 MolWeight:298 RetIndex:2077
CompName:Heptadecyl acetate $$ 1-Heptadecanol, acetate $$ n-Heptadecyl acetate $$ Heptadecanyl acetate $$ 1-Heptadecyl acetate $$

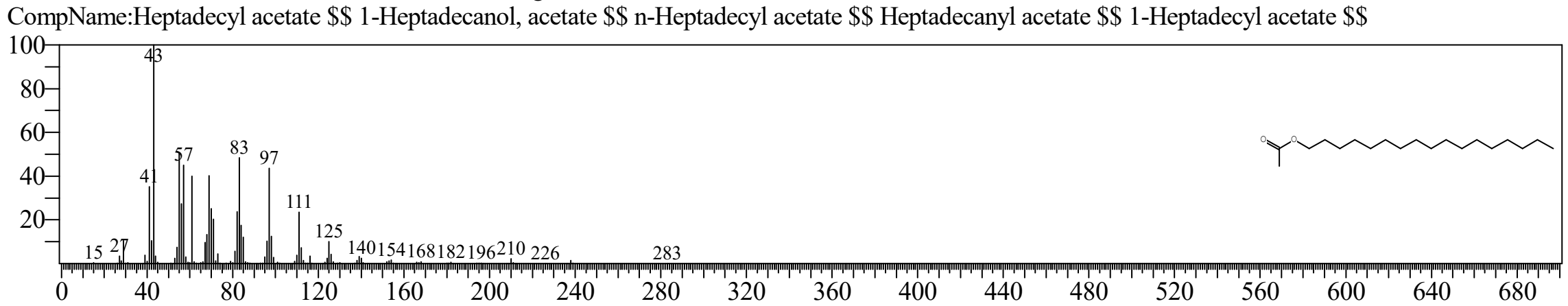

Hit#:2 Entry:80625 Library:NIST14.lib
SI:80 Formula:C15H30O2 CAS:0-00-0 MolWeight:242 RetIndex:1680
CompName:Tridecyl acetate $$ n-Tridecyl acetate $$ Acetic acid, tridecyl ester $$

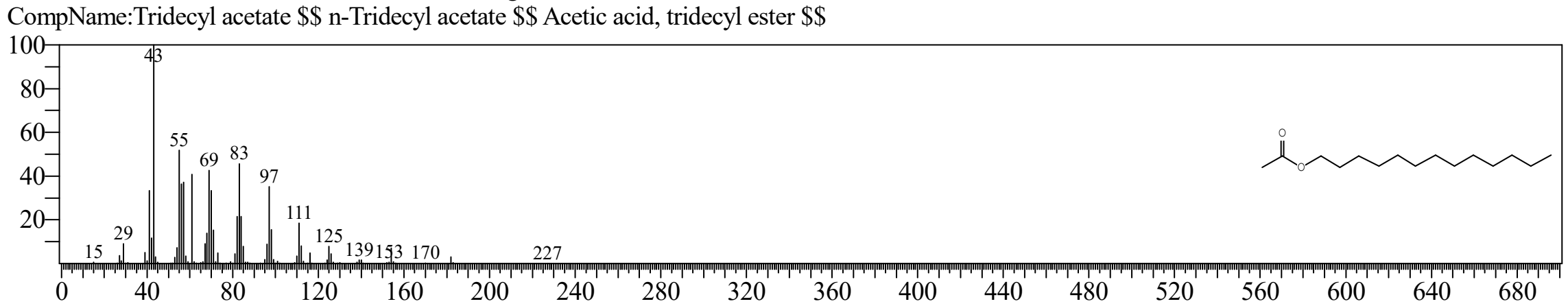

Hit#:3 Entry:29925 Library:NIST14s.lib
SI:80 Formula:C21H42O2 CAS:1577-43-1 MolWeight:326 RetIndex:2276
CompName:1-Acetoxynonadecane $$ Nonadecyl acetate # $$

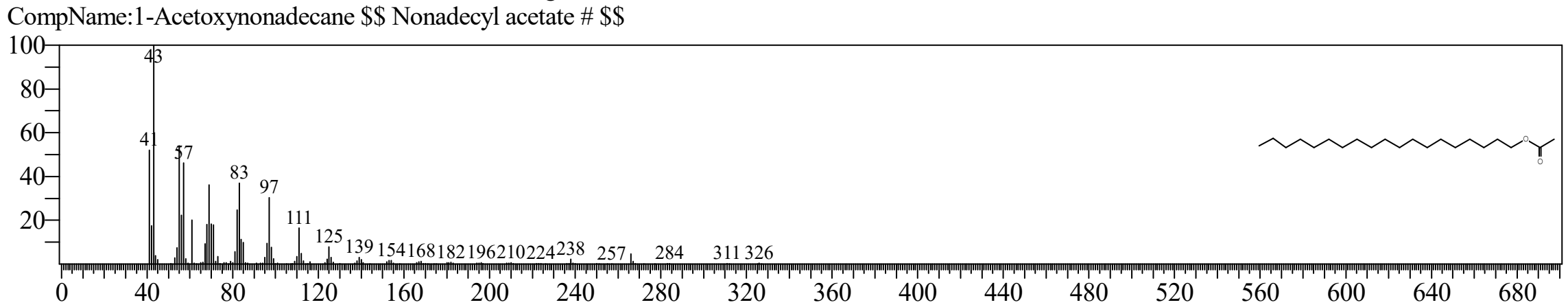

<< Target >>
Line#:137   R.Time:20.325(Scan#:3366) MassPeaks:136
RawMode:Averaged 20.320-20.330(3365-3367) BasePeak:43.05(49111)
BG Mode:Calc. from Peak Group 1 - Event 1 Scan

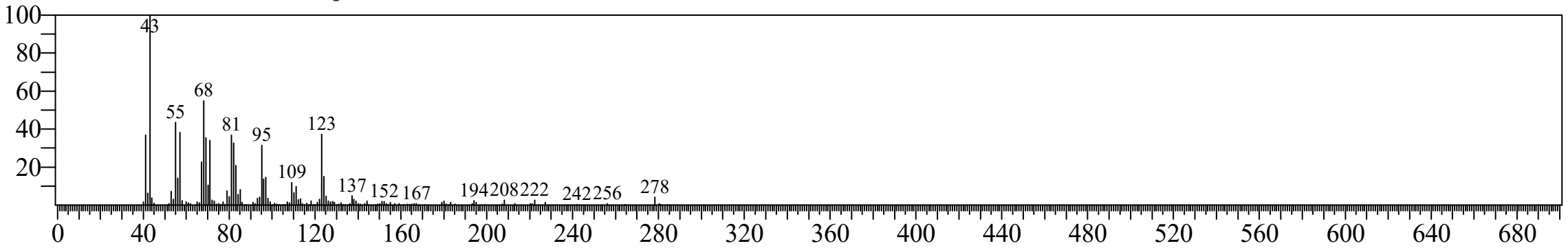

Hit#:1 Entry:165113 Library:NIST14.lib
SI:89 Formula:C22H42O2 CAS:0-00-0 MolWeight:338 RetIndex:2089
CompName:Isophytol, acetate

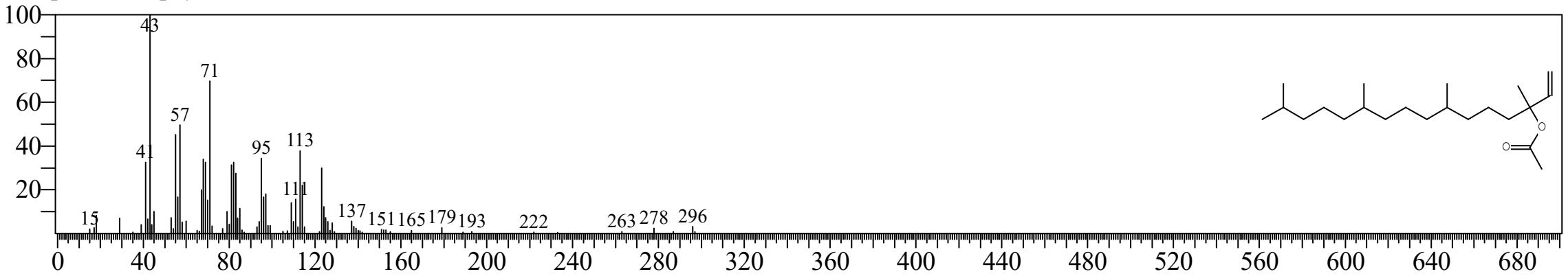

Hit#:2 Entry:235608 Library:WILEY8.LIB
SI:88 Formula:C20H40O CAS:0-00-0 MolWeight:296 RetIndex:0
CompName:2-HEXADECEN-1-OL, 3,7,11,15-TETRAMETHYL-, [R-[R*,R*-(E)]]- (T-PHYTOL) $$ 3,7,11,15-TETRAMETHYL-2-HEXADECEN-1-OL

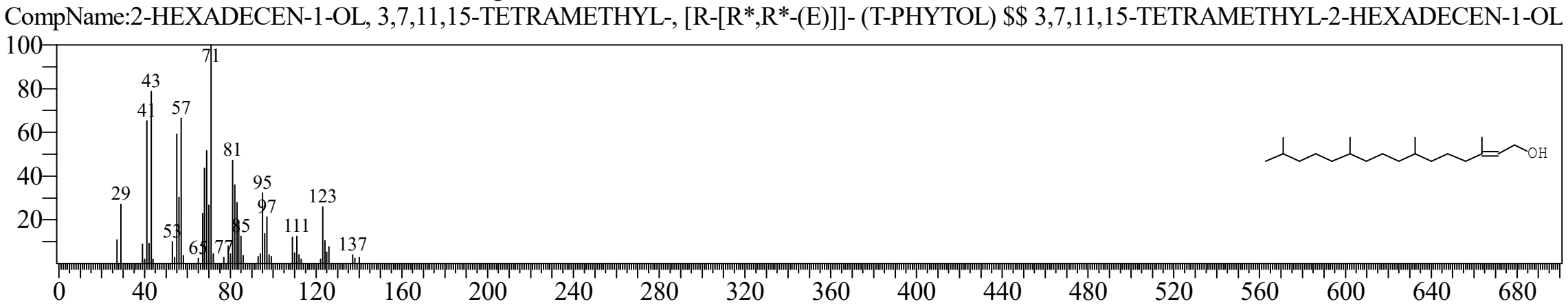

Hit#:3 Entry:28152 Library:NIST14s.lib
SI:88 Formula:C20H40O CAS:150-86-7 MolWeight:296 RetIndex:2045
CompName:Phytol $$ 2-Hexadecen-1-ol, 3,7,11,15-tetramethyl-, [R-[R*,R*-(E)]]- $$ trans-Phytol $$ 3,7,11,15-Tetramethyl-2-hexadecen-1-ol-, (2E,7R,11R

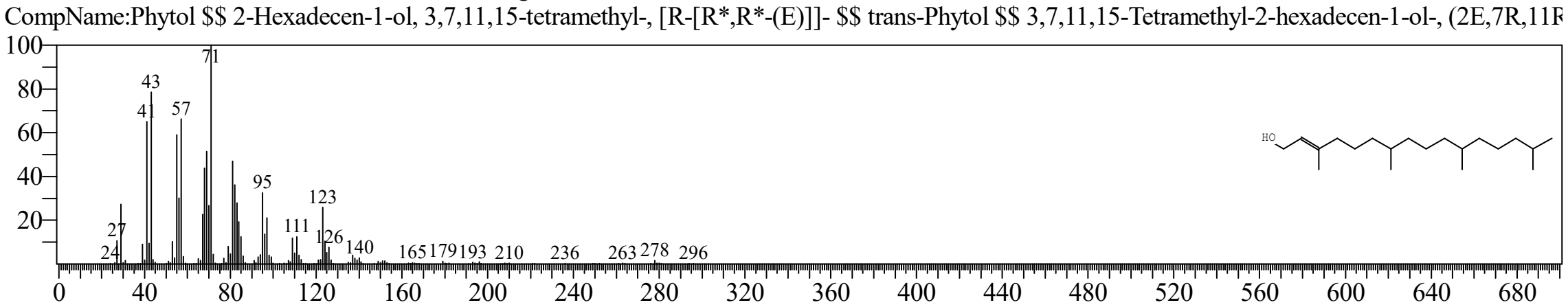

<< Target >>
Line#:138   R.Time:20.485(Scan#:3398) MassPeaks:117
RawMode:Averaged 20.480-20.490(3397-3399) BasePeak:195.10(4031)
BG Mode:Calc. from Peak Group 1 - Event 1 Scan

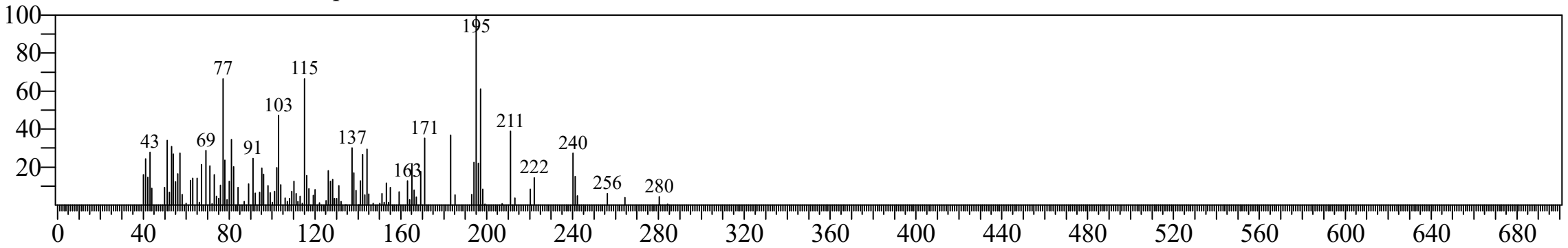

Hit#:1 Entry:161848 Library:WILEY8.LIB
SI:51 Formula:C17H20O CAS:0-00-0 MolWeight:240 RetIndex:0
CompName:1-ETHOXY-7-(2-PHENYLETHENYLIDENE)BICYCLO[4.1.0]HEPTANE $$ 1-ETHOXY-7-PHENYLVINYLIDENE-BICYCLO[4.1.0]HEP

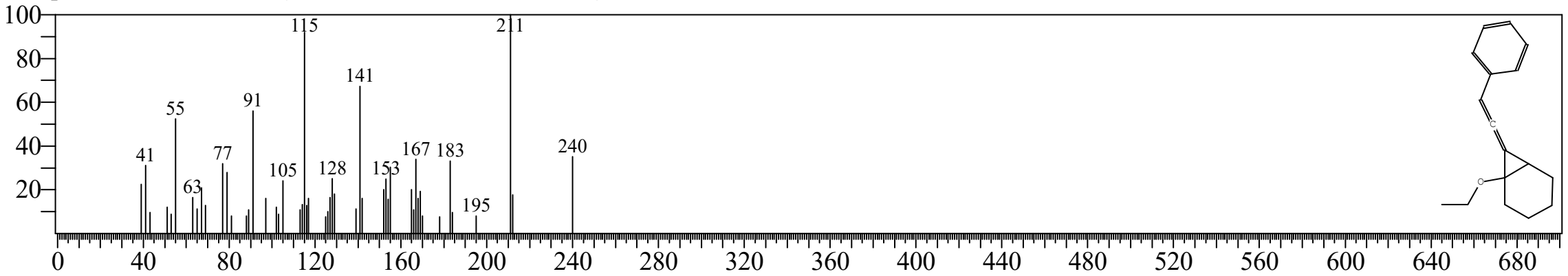

Hit#:2 Entry:79106 Library:NIST14.lib
SI:51 Formula:C17H20O CAS:0-00-0 MolWeight:240 RetIndex:0
CompName:1-Ethoxy-7-phenylvinylidene-bicyclo[4.1.0]heptane $$ 1-Ethoxy-7-(2-phenylethenylidene)bicyclo[4.1.0]heptane # $$

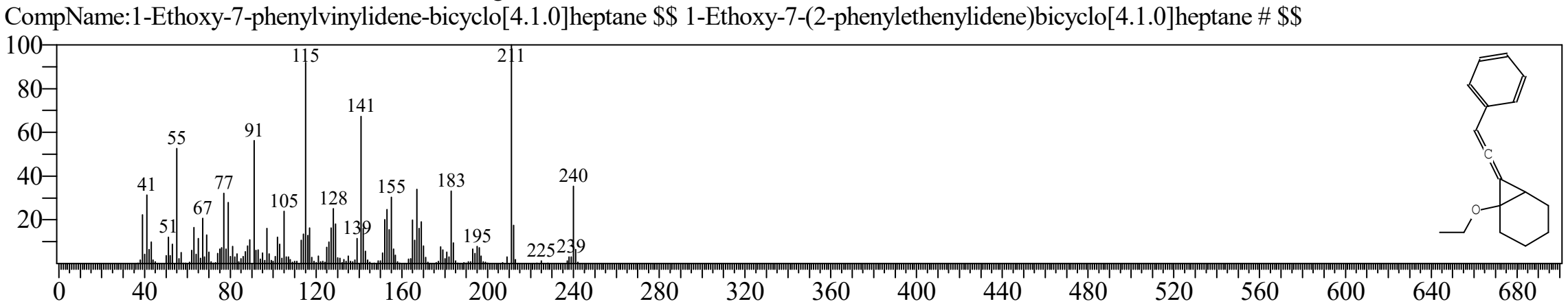

Hit#:3 Entry:161847 Library:WILEY8.LIB
SI:50 Formula:C17H20O CAS:0-00-0 MolWeight:240 RetIndex:0
CompName:1-ETHOXY-7-(PHENYLETHYNYL)BICYCLO[4.1.0]HEPTANE $$ 1-ETHOXY-7-PHENYLETHYNYL-BICYCLO[4.1.0]HEPTANE $$ ET

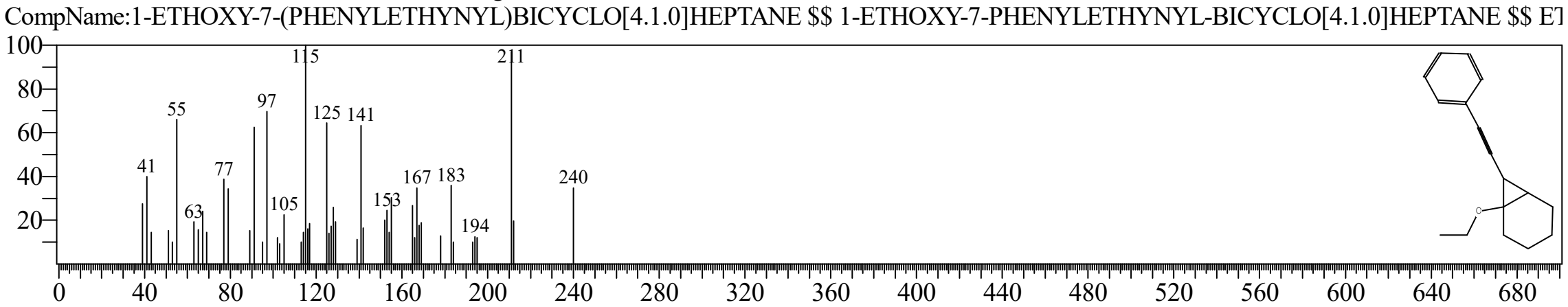

<< Target >>
Line#:139 R.Time:20.620(Scan#:3425) MassPeaks:104
RawMode:Averaged 20.615-20.625(3424-3426) BasePeak:43.05(3305)
BG Mode:Calc. from Peak Group 1 - Event 1 Scan

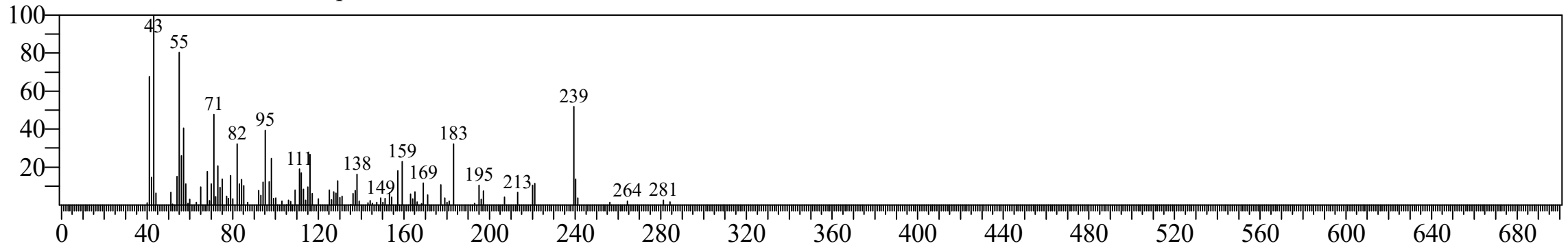

Hit#:1 Entry:238294 Library:NIST14.lib
SI:69 Formula:C35H68O5 CAS:761-35-3 MolWeight:568 RetIndex:4013
CompName:Hexadecanoic acid, 1-(hydroxymethyl)-1,2-ethanediyl ester $$ Palmitin, 1,2-di- $$ Dipalmitin $$ Glycerol 1,2-dipalmitate $$ 1,2-Dipalmitin $$

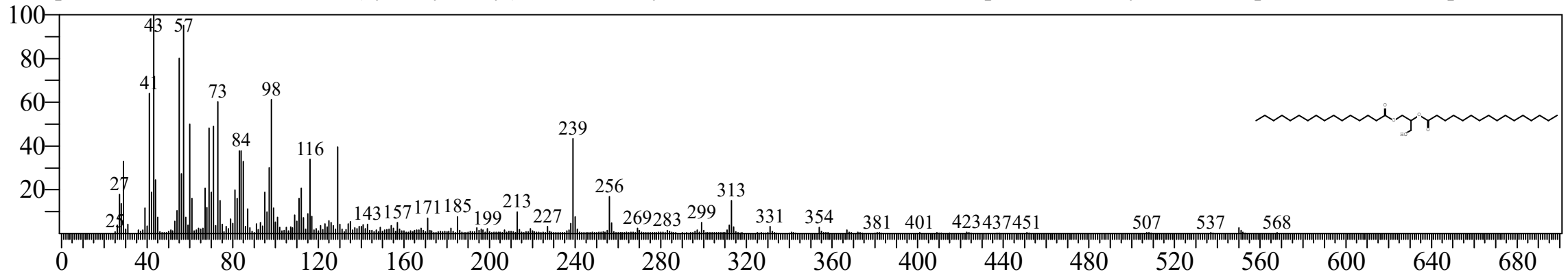

Hit#:2 Entry:386726 Library:WILEY8.LIB
SI:69 Formula:C35H68O5 CAS:761-35-3 MolWeight:568 RetIndex:0
CompName:HEXADECANOIC ACID, 1-(HYDROXYMETHYL)-1,2-ETHANEDIYL ESTER $$ 2-HYDROXY-1-[(PALMITOYLOXY)METHYL]ETHY

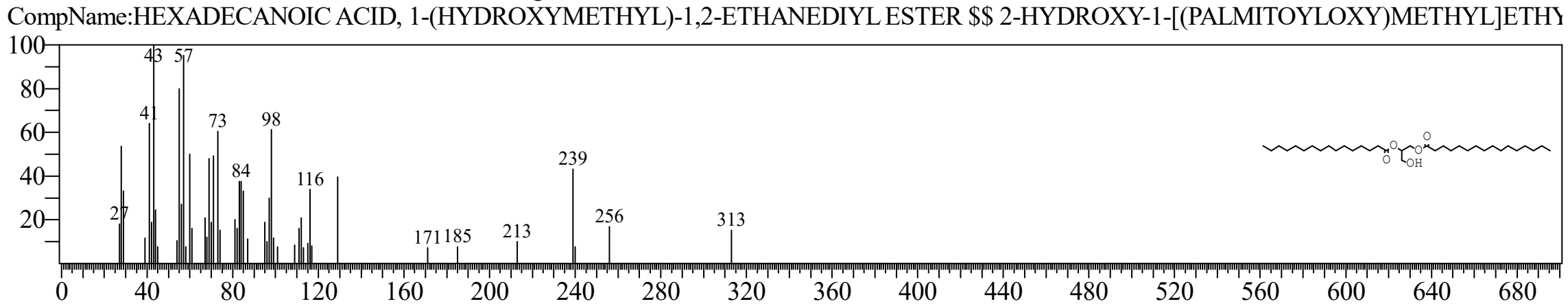

Hit#:3 Entry:386724 Library:WILEY8.LIB
SI:69 Formula:C35H68O5 CAS:502-52-3 MolWeight:568 RetIndex:0
CompName:HEXADECANOIC ACID, 2-HYDROXY-1,3-PROPANEDIYL ESTER $$ 2-HYDROXY-3-(PALMITOYLOXY)PROPYL PALMITATE # $$

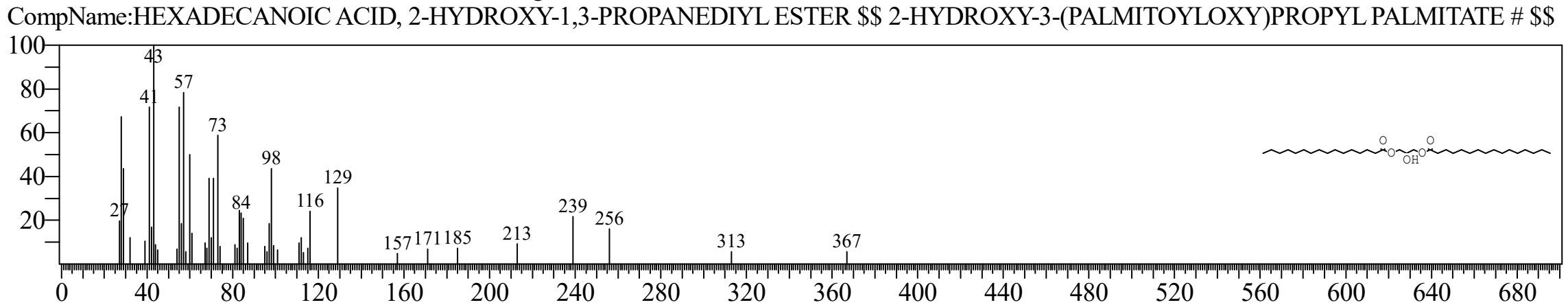

<< Target >>
Line#:140   R.Time:20.740(Scan#:3449) MassPeaks:119
RawMode:Averaged 20.735-20.745(3448-3450) BasePeak:55.05(14078)
BG Mode:Calc. from Peak Group 1 - Event 1 Scan

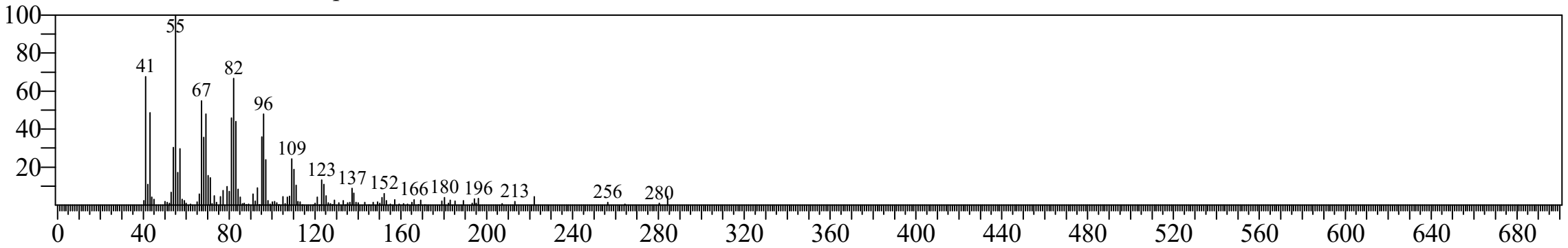

Hit#:1 Entry:199389 Library:WILEY8.LIB
SI:93 Formula:C18H36O CAS:143-28-2 MolWeight:268 RetIndex:0
CompName:9-OCTADECEN-1-OL, (Z)- $$ OCTADEC-9-EN-1-OL $$ (9Z)-9-OCTADECEN-1-OL $$ (Z)-9-OCTADECEN-1-OL $$ (Z)-9-OCTADECEN

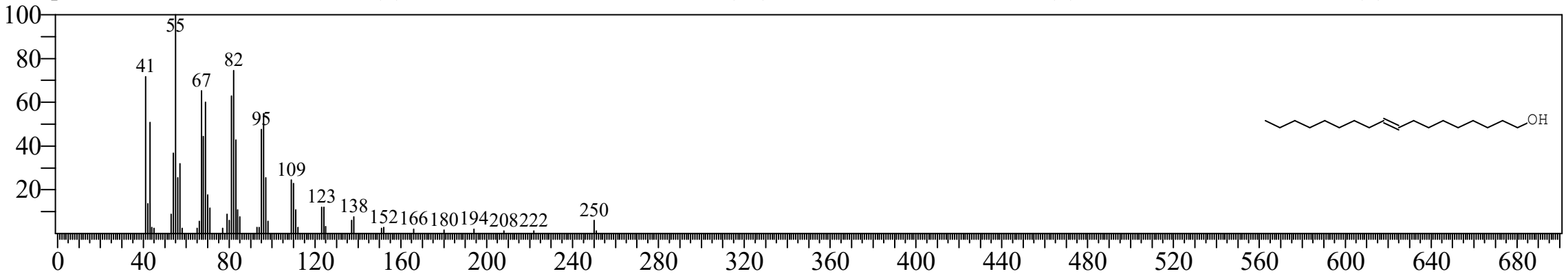

Hit#:2 Entry:127768 Library:NIST14.lib
SI:93 Formula:C20H40O CAS:112248-30-3 MolWeight:296 RetIndex:2260
CompName:Eicosen-1-ol, cis-9- $$ (9Z)-9-Icosen-1-ol # $$

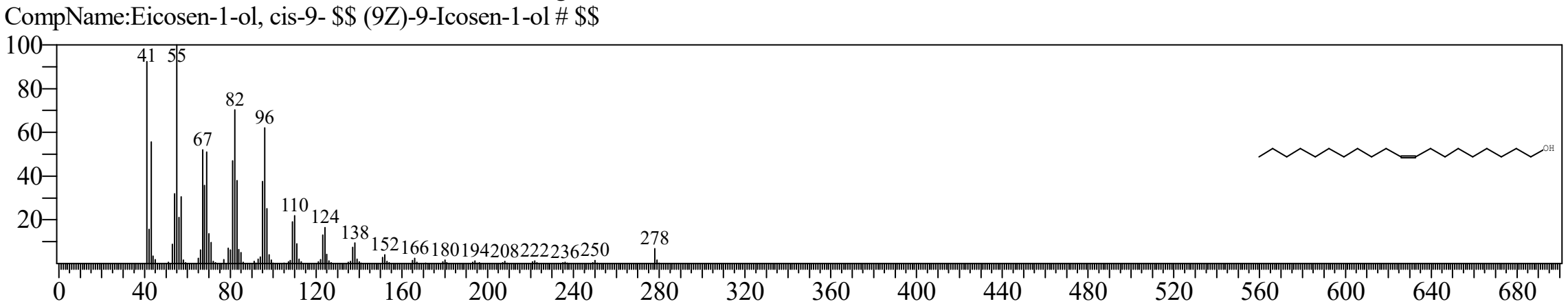

Hit#:3 Entry:26081 Library:NIST14s.lib
SI:92 Formula:C18H36O CAS:506-42-3 MolWeight:268 RetIndex:2061
CompName:9-Octadecen-1-ol, (E)- $$ trans-9-Octadecen-1-ol $$ Elaidyl alcohol $$ 9-Octadecen-1-ol, trans- $$ (9E)-9-Octadecen-1-ol # $$

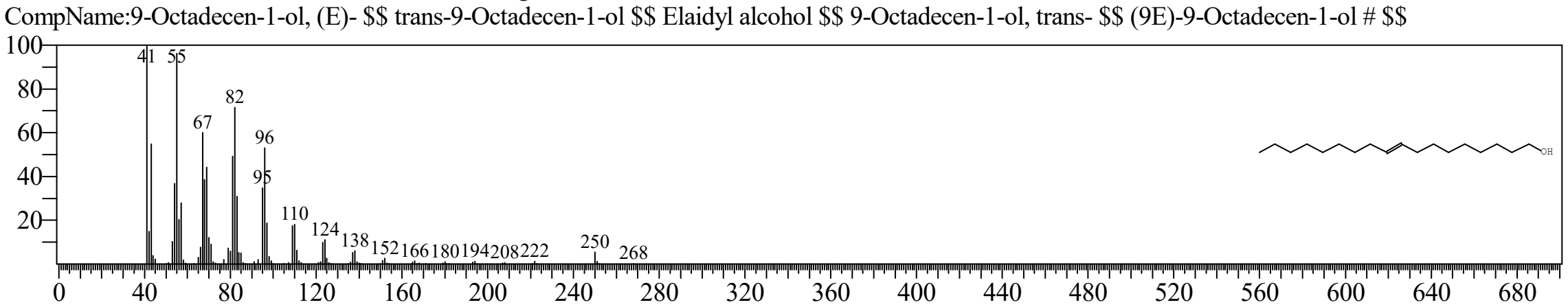

<< Target >>
Line#:141 R.Time:20.860(Scan#:3473) MassPeaks:77
RawMode:Averaged 20.855-20.865(3472-3474) BasePeak:69.05(1281)
BG Mode:Calc. from Peak Group 1 - Event 1 Scan

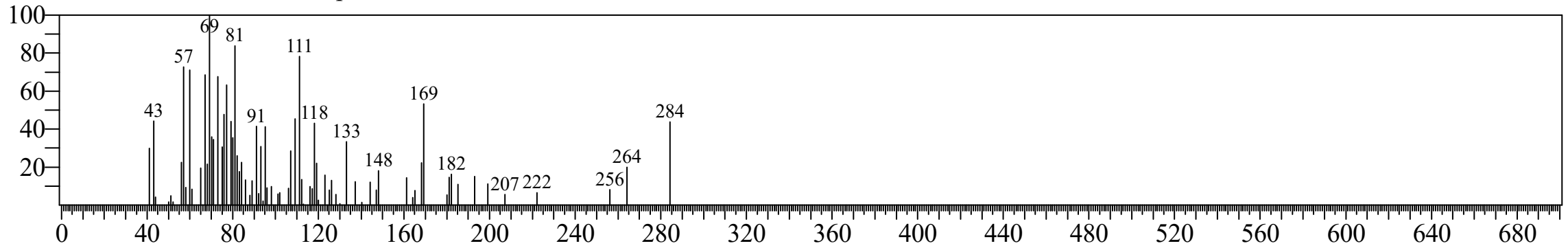

Hit#:1 Entry:335263 Library:WILEY8.LIB
SI:62 Formula:C28H48O CAS:22599-96-8 MolWeight:400 RetIndex:0
CompName:CHOLESTAN-3-OL, 2-METHYLENE-, (3.BETA.,5.ALPHA.)- $$ 2-METHYLENECHOLESTAN-3-OL # $$ 2-METHYLENECHOLESTAN

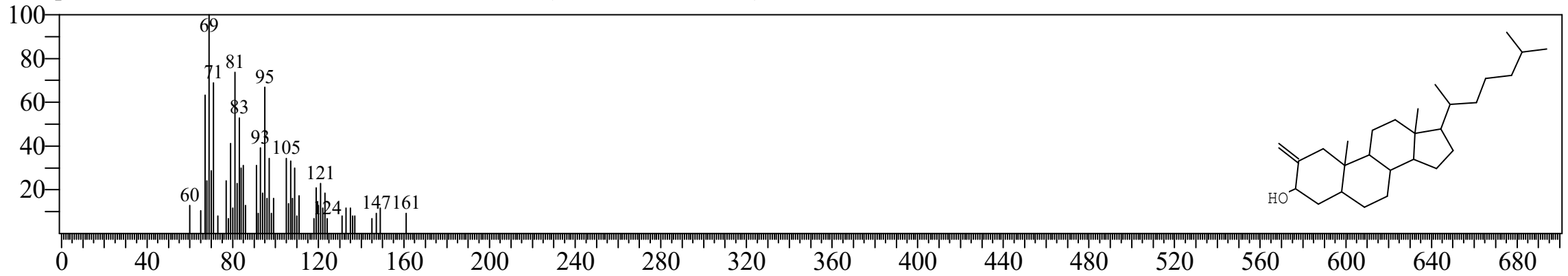

Hit#:2 Entry:135938 Library:WILEY8.LIB
SI:62 Formula:C15H26O CAS:142-50-7 MolWeight:222 RetIndex:0
CompName:1,6,10-DODECATRIEN-3-OL, 3,7,11-TRIMETHYL-, [S-(Z)]- $$ (6Z)-3,7,11-TRIMETHYL-1,6,10-DODECATRIEN-3-OL # $$ (+)-NEROL

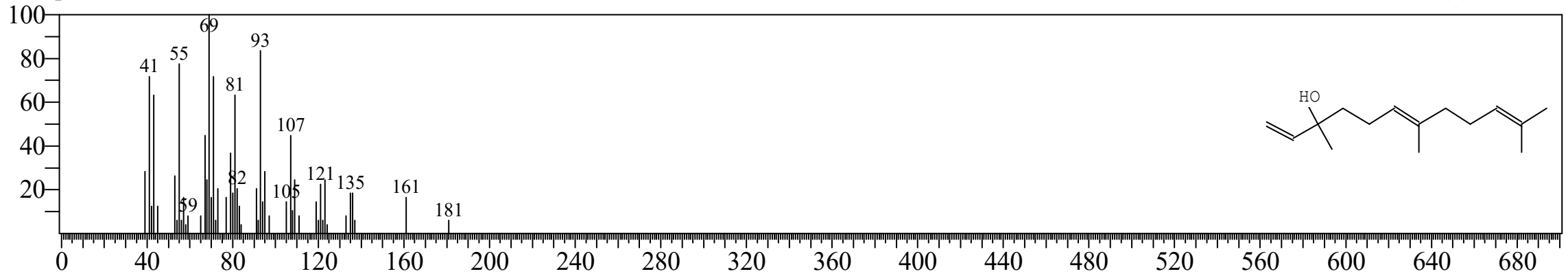

Hit#:3 Entry:46777 Library:WILEY8.LIB
SI:61 Formula:C10H18O CAS:89-79-2 MolWeight:154 RetIndex:0
CompName:CYCLOHEXANOL, 5-METHYL-2-(1-METHYLETHENYL)-, [1R-(1.ALPHA.,2.BETA.,5.ALPHA.)]- $$ 2-ISOPROPENYL-5-METHYLCY

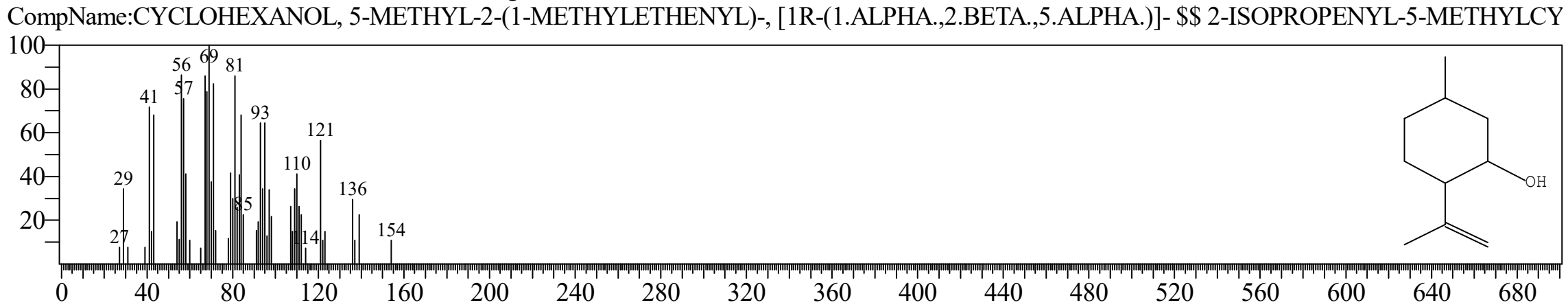

<< Target >>
Line#:142 R.Time:20.970(Scan#:3495) MassPeaks:189
RawMode:Averaged 20.965-20.975(3494-3496) BasePeak:91.05(77217)
BG Mode:Calc. from Peak Group 1 - Event 1 Scan

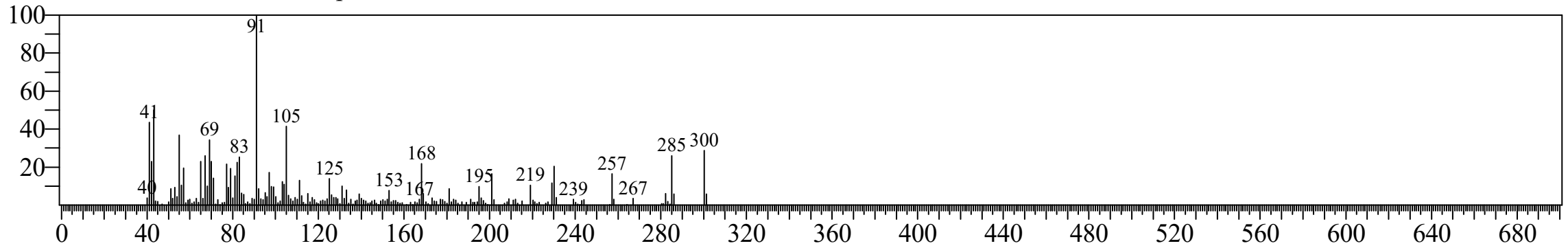

Hit#:1 Entry:325704 Library:WILEY8.LIB
SI:69 Formula:C28H50 CAS:55334-72-0 MolWeight:386 RetIndex:0
CompName:BENZENE, (2-DECYLDODECYL)- $$ (2-DECYLDODECYL)BENZENE # $$ (2-DECYLDODECYL)BENZENE $$ (2-DECYLDODECYI

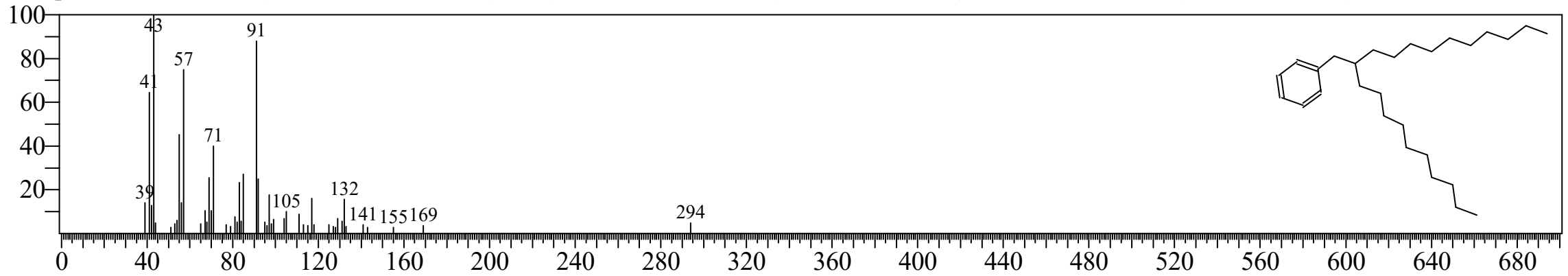

Hit#:2 Entry:185555 Library:WILEY8.LIB
SI:69 Formula:C16H22N2O CAS:339320-81-9 MolWeight:258 RetIndex:0
CompName:N'-[(1Z)-2-ETHYLCYCLOPENTYLIDENE]-3-PHENYLPROPANOHYDRAZIDE # $$ N'-[(1Z)-2-ETHYLCYCLOPENTYLIDENE]-3-PHE

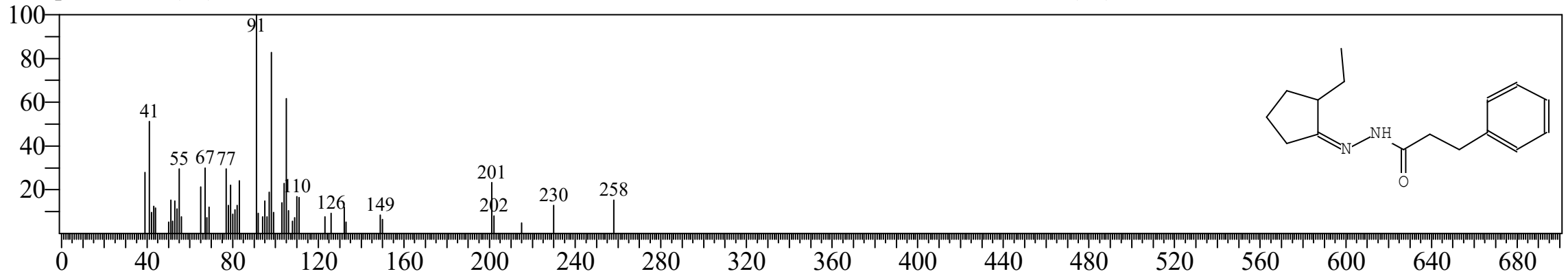

Hit#:3 Entry:94207 Library:NIST14.lib
SI:69 Formula:C16H22N2O CAS:339320-81-9 MolWeight:258 RetIndex:2324
CompName:Propanehydrazide, 3-phenyl-N2-(2-ethylcyclopentylideno)- $$ N'-[(1Z)-2-Ethylcyclopentylidene]-3-phenylpropanohydrazide # $$

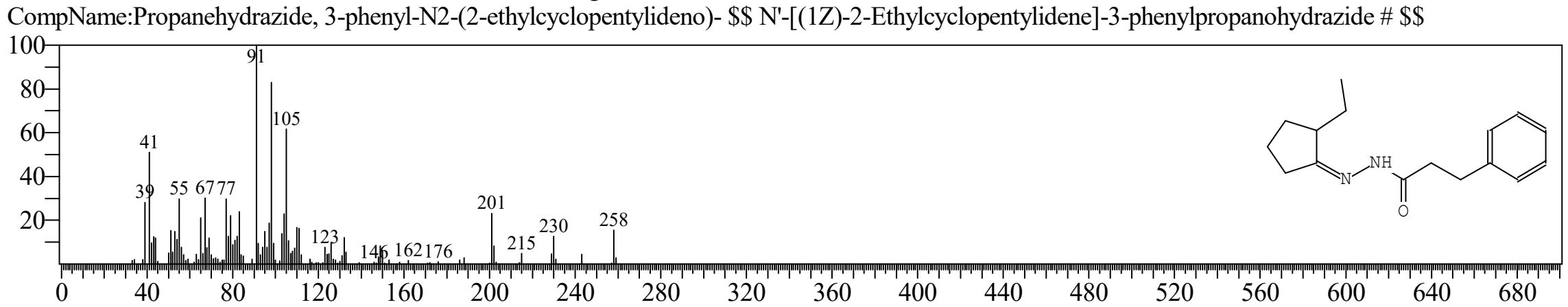

<< Target >>
Line#:143   R.Time:21.190(Scan#:3539) MassPeaks:105
RawMode:Averaged 21.185-21.195(3538-3540) BasePeak:91.05(3199)
BG Mode:Calc. from Peak Group 1 - Event 1 Scan

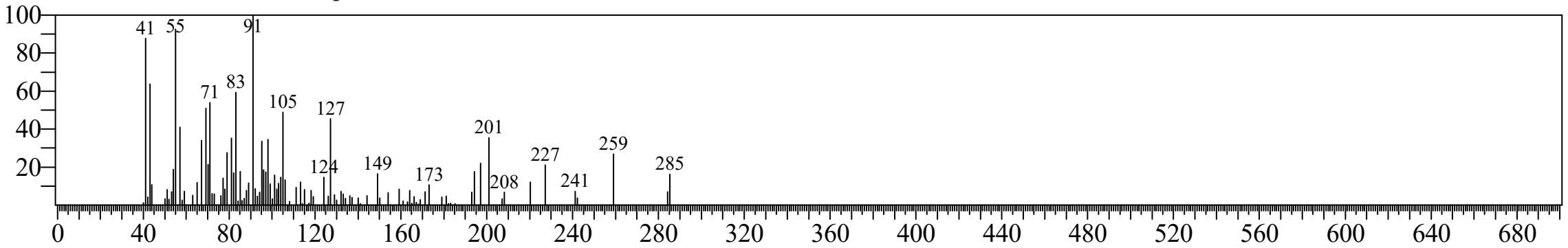

Hit#:1 Entry:232225 Library:WILEY8.LIB
SI:68 Formula:C16H32Cl2 CAS:7735-39-9 MolWeight:294 RetIndex:0
CompName:1,16-DICHLOROHEXADECANE $$ 1,16-DICHLOROHEXADECANE # $$ HEXADECANE, 1,16-DICHLORO-

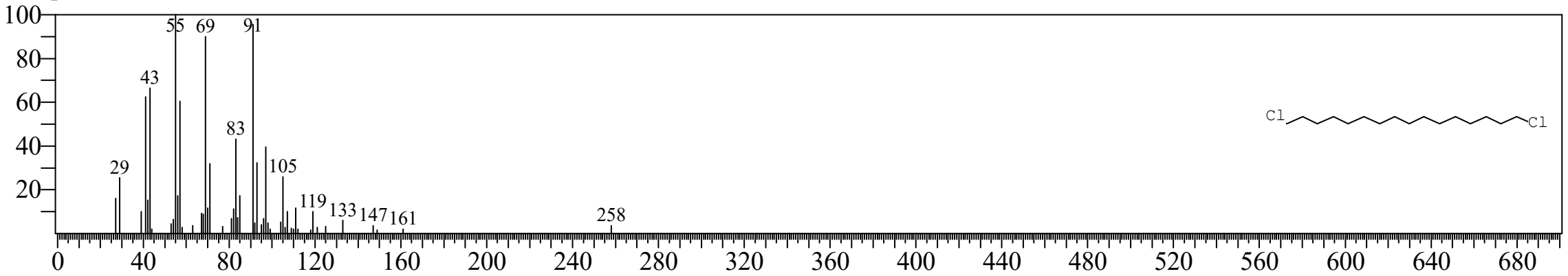

Hit#:2 Entry:245531 Library:WILEY8.LIB
SI:68 Formula:C21H38N CAS:6004-24-6 MolWeight:304 RetIndex:0
CompName:PYRIDINIUM, 1-HEXADECYL-, CHLORIDE, MONOHYDRATE $$ 1-HEXADECYLPYRIDINIUM CHLORIDE MONOHYDRATE $$ C

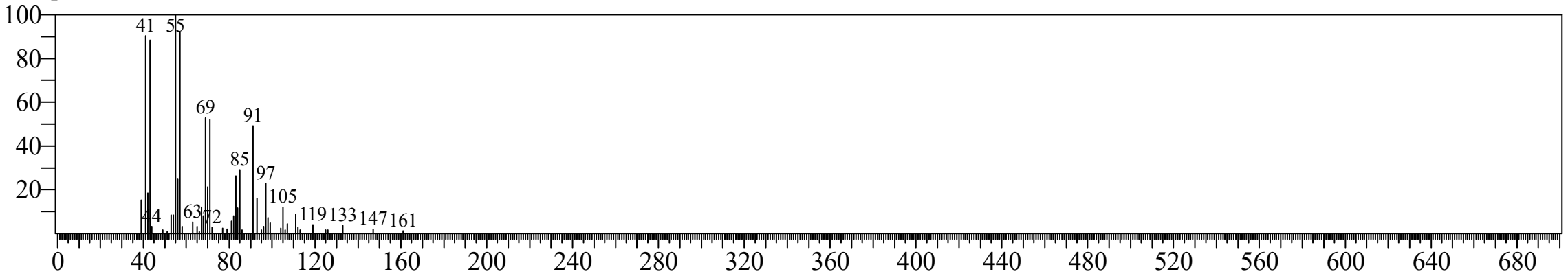

Hit#:3 Entry:125598 Library:NIST14.lib
SI:68 Formula:C16H32Cl2 CAS:7735-39-9 MolWeight:294 RetIndex:2062
CompName:Hexadecane, 1,16-dichloro- $$ 1,16-Dichlorohexadecane # $$

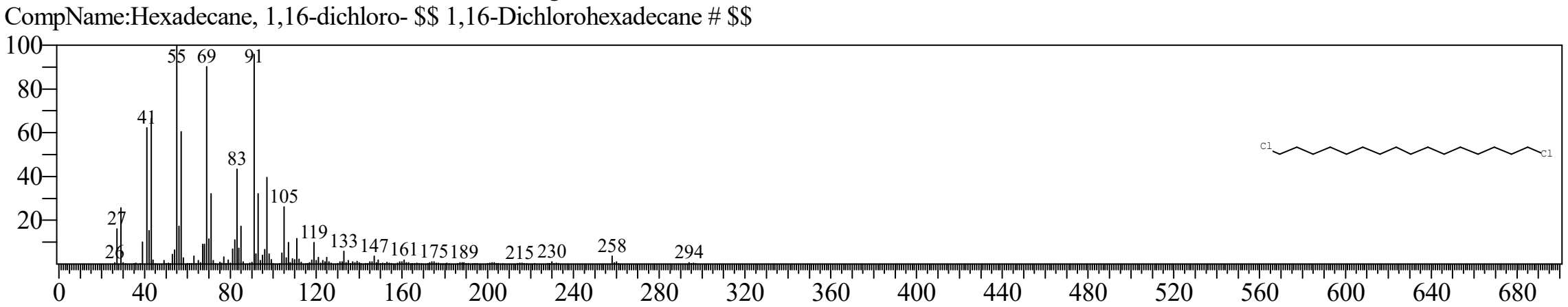

<< Target >>
Line#:144   R.Time:21.235(Scan#:3548)   MassPeaks:115
RawMode:Averaged 21.230-21.240(3547-3549) BasePeak:205.20(6792)
BG Mode:Calc. from Peak Group 1 - Event 1 Scan

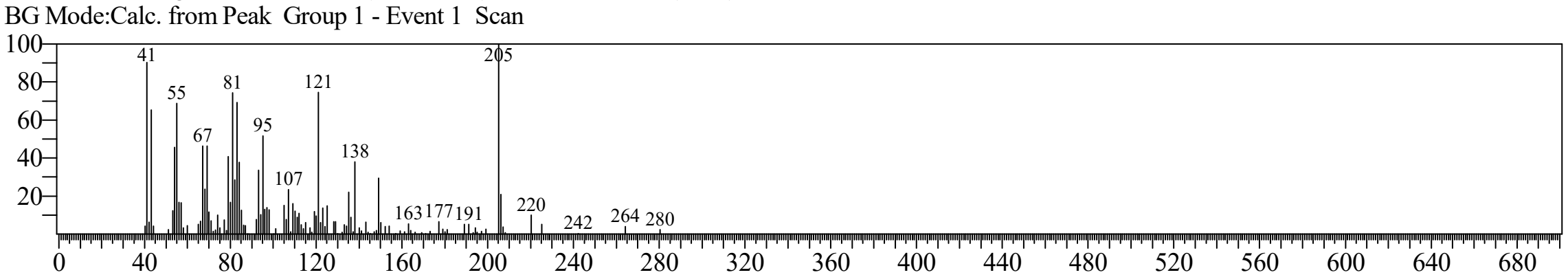

Hit#:1 Entry:77346 Library:NIST14.lib
SI:79 Formula:C16H30O CAS:56797-40-1 MolWeight:238 RetIndex:1808
CompName:7-Hexadecenal, (Z)- $$ (Z)-7-Hexadecenal $$ Z-7-Hexadecenal $$ (7Z)-7-Hexadecenal # $$

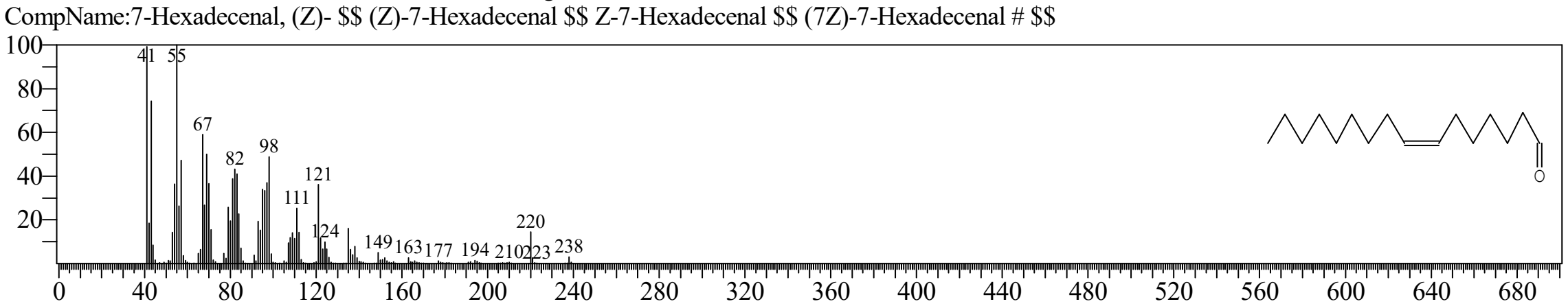

Hit#:2 Entry:23433 Library:NIST14s.lib
SI:78 Formula:C16H30O CAS:56219-04-6 MolWeight:238 RetIndex:1808
CompName:cis-9-Hexadecenal $$ 9-Hexadecenal, (Z)- $$ (Z)-9-Hexadecenal $$ Z-9-Hexadecenal $$ (9Z)-9-Hexadecenal # $$

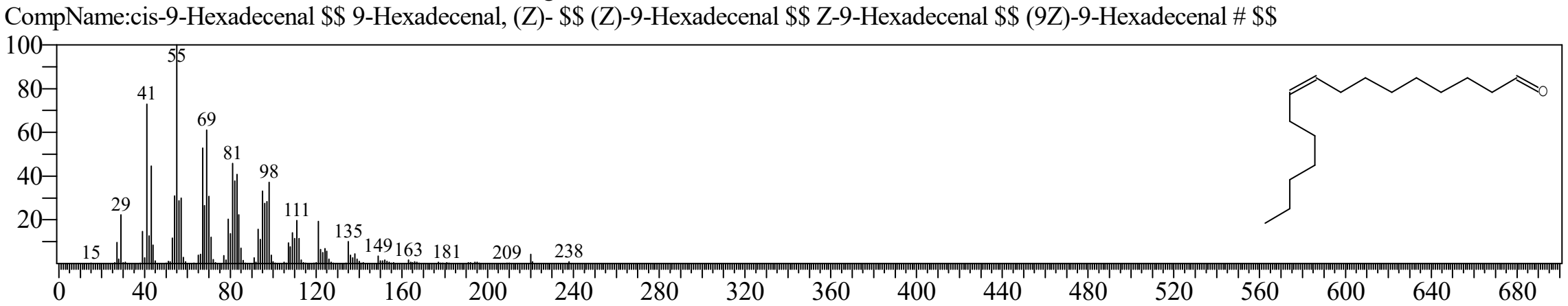

Hit#:3 Entry:77352 Library:NIST14.lib
SI:78 Formula:C16H30O CAS:56219-04-6 MolWeight:238 RetIndex:1808
CompName:cis-9-Hexadecenal $$ 9-Hexadecenal, (Z)- $$ (Z)-9-Hexadecenal $$ Z-9-Hexadecenal $$ (9Z)-9-Hexadecenal # $$

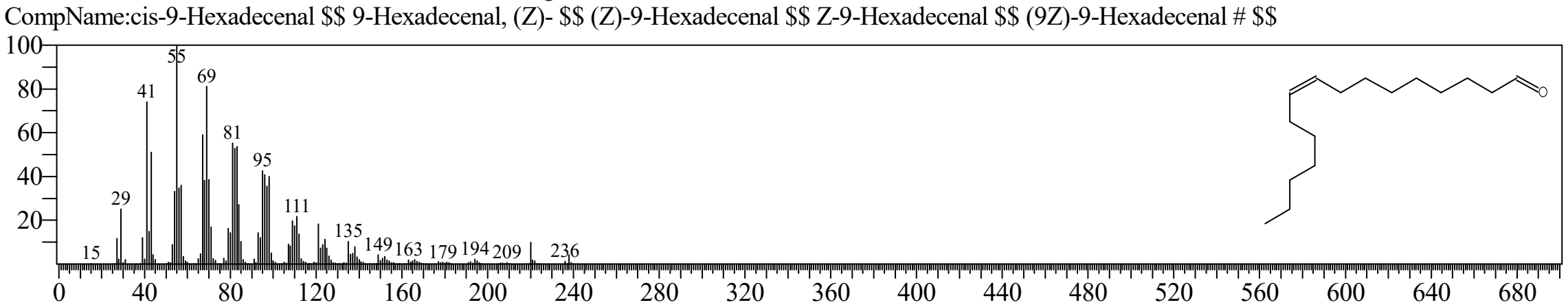

<< Target >>
Line#:145 R.Time:21.385(Scan#:3578) MassPeaks:113
RawMode:Averaged 21.380-21.390(3577-3579) BasePeak:205.20(10218)
BG Mode:Calc. from Peak Group 1 - Event 1 Scan

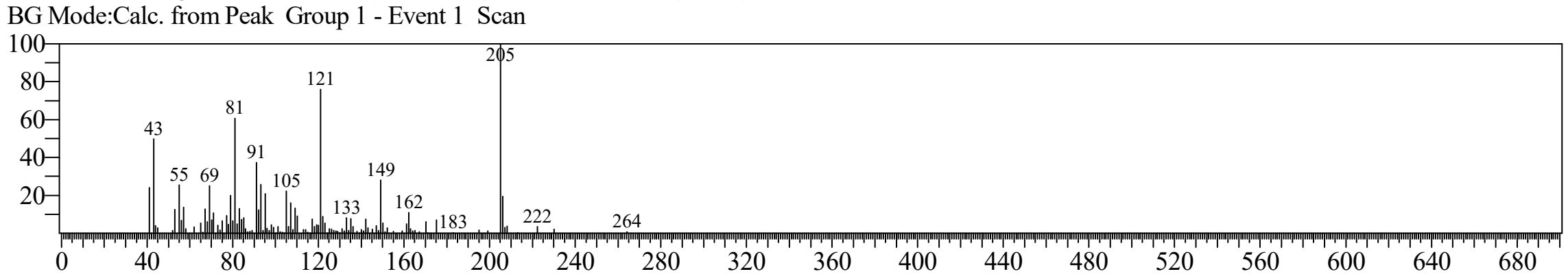

Hit#:1 Entry:341216 Library:WILEY8.LIB
SI:74 Formula:C30H50 CAS:0-00-0 MolWeight:410 RetIndex:0
CompName:2,6,6,9,2',6',6',9'-OCTAMETHYL-[8,8']BI[TRICYCLO[5.4.0.0 2,9]UNDECYL]

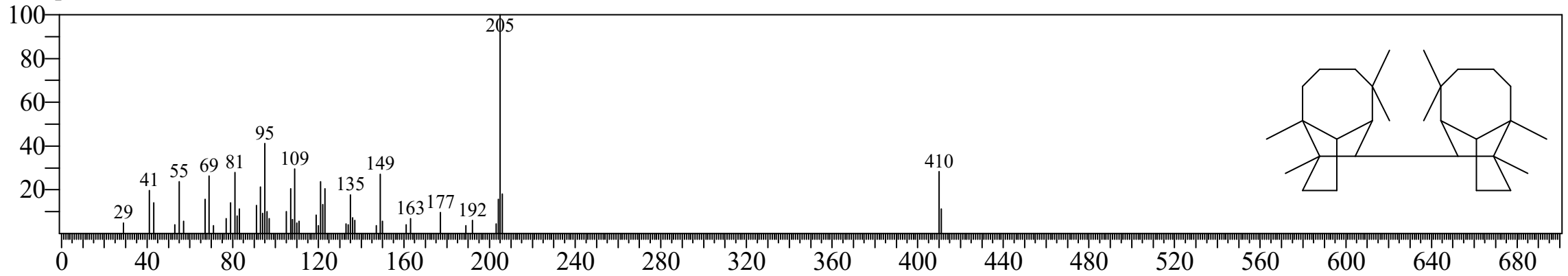

Hit#:2 Entry:210644 Library:NIST14.lib
SI:74 Formula:C30H50 CAS:0-00-0 MolWeight:410 RetIndex:2589
CompName:2,6,6,9,2',6',6',9'-Octamethyl-[8,8']bi[tricyclo[5.4.0.0(2,9)]undecyl]

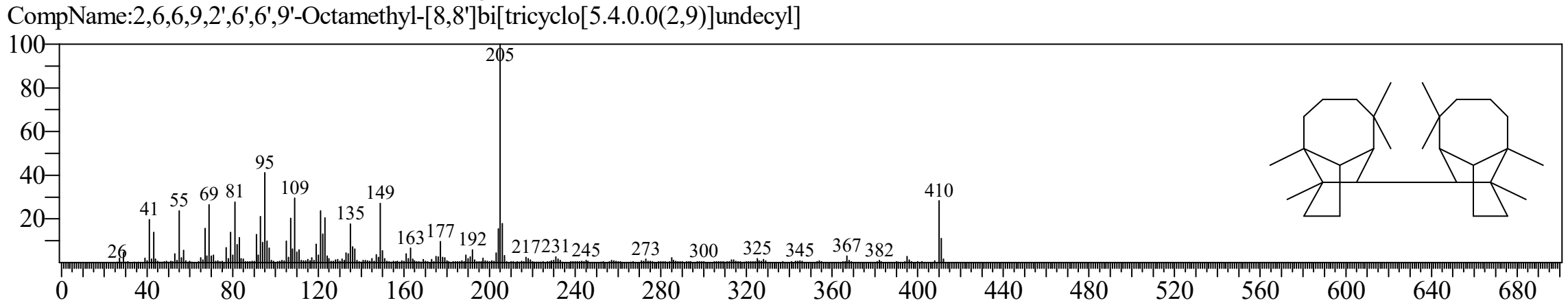

Hit#:3 Entry:75642 Library:NIST14.lib
SI:73 Formula:C16H28O CAS:137235-47-3 MolWeight:236 RetIndex:1721
CompName:2-Hydroxymethyl-2,6,8,8-tetramethyltricyclo[5.2.2.0(1,6)]undecane

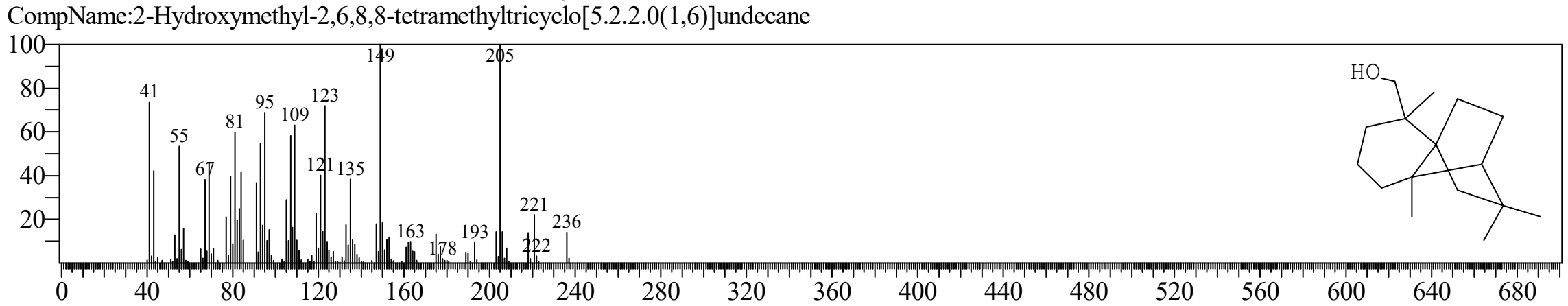

<< Target >>
Line#:146   R.Time:21.550(Scan#:3611) MassPeaks:133
RawMode:Averaged 21.545-21.555(3610-3612) BasePeak:99.05(56768)
BG Mode:Calc. from Peak Group 1 - Event 1 Scan

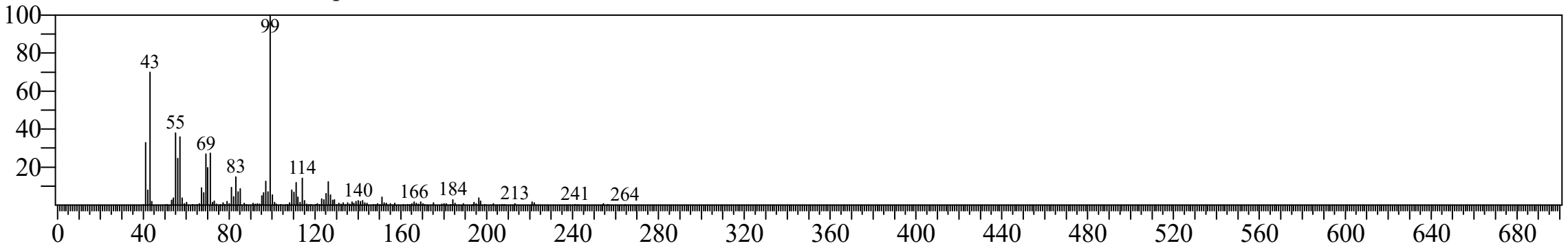

Hit#:1 Entry:152777 Library:NIST14.lib
SI:91 Formula:C21H40O2 CAS:96168-15-9 MolWeight:324 RetIndex:2258
CompName:4,8,12,16-Tetramethylheptadecan-4-olide $$ 5-Methyl-5-(4,8,12-trimethyltridecyl)dihydro-2(3H)-furanone # $$

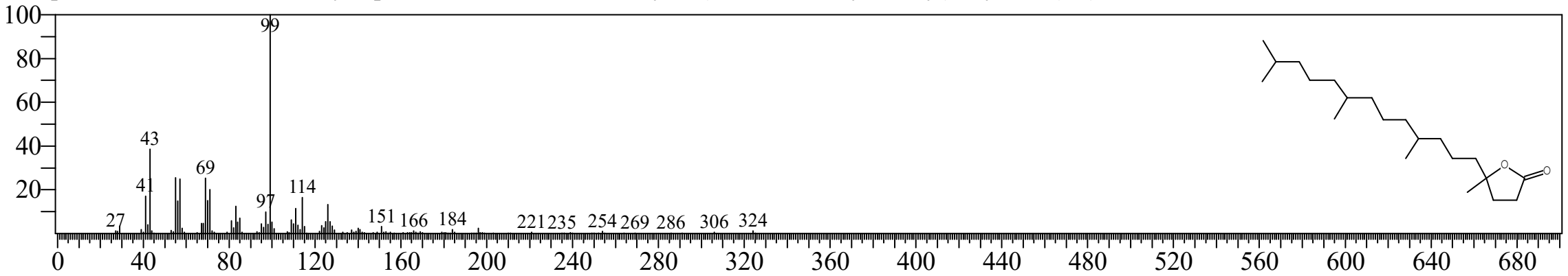

Hit#:2 Entry:29836 Library:NIST14s.lib
SI:89 Formula:C21H40O2 CAS:96168-15-9 MolWeight:324 RetIndex:2258
CompName:4,8,12,16-Tetramethylheptadecan-4-olide $$ 5-Methyl-5-(4,8,12-trimethyltridecyl)dihydro-2(3H)-furanone # $$

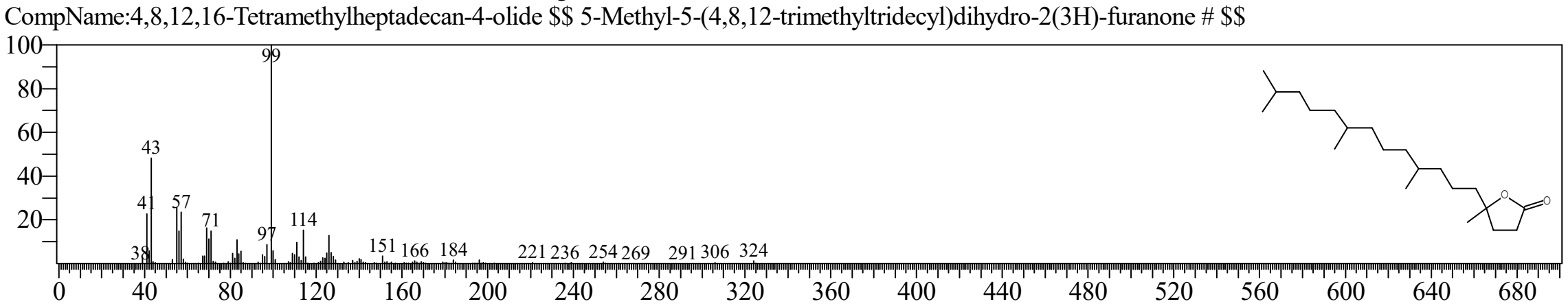

Hit#:3 Entry:167037 Library:NIST14.lib
SI:85 Formula:C22H44O2 CAS:0-00-0 MolWeight:340 RetIndex:2311
CompName:Hexanoic acid, 4-hexadecyl ester $$ 1-Propyltridecyl hexanoate # $$

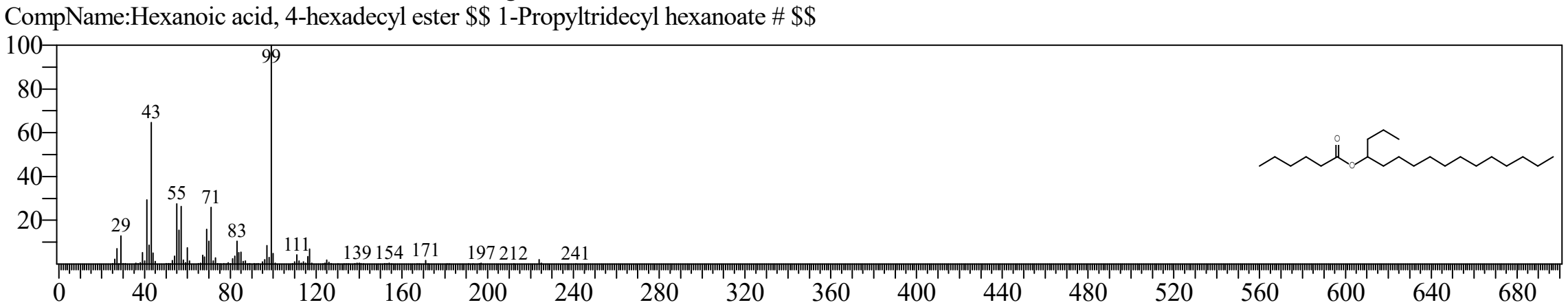

<< Target >>
Line#:147   R.Time:21.620(Scan#:3625) MassPeaks:87
RawMode:Averaged 21.615-21.625(3624-3626) BasePeak:69.05(2377)
BG Mode:Calc. from Peak Group 1 - Event 1 Scan

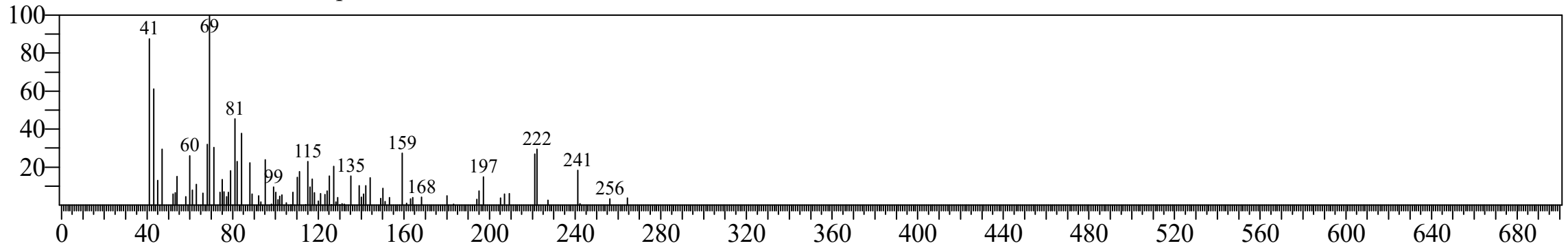

Hit#:1 Entry:101376 Library:WILEY8.LIB
SI:58 Formula:C12H22O2 CAS:7367-84-2 MolWeight:198 RetIndex:0
CompName:4-DECENOIC ACID, ETHYL ESTER, (Z)- $$ 4-DECENOIC ACID, ETHYL ESTER, CIS- $$ ETHYL (4Z)-4-DECENOATE # $$ EINECS 2

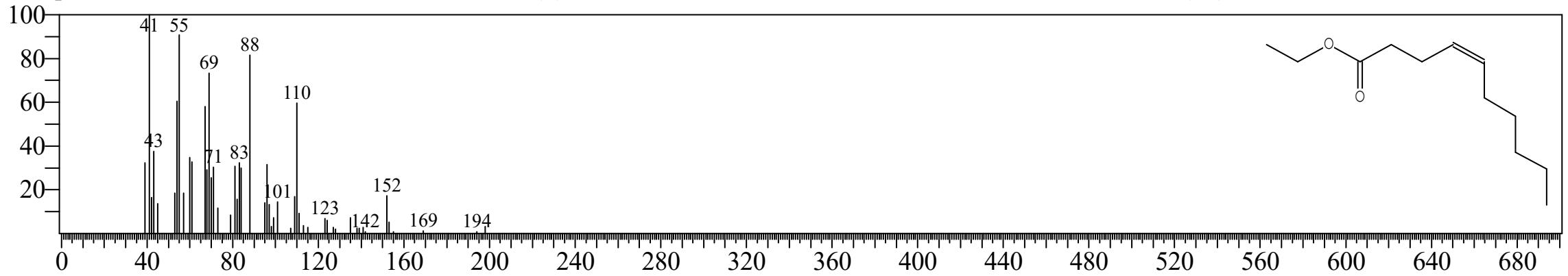

Hit#:2 Entry:18146 Library:NIST14s.lib
SI:58 Formula:C12H22O2 CAS:7367-84-2 MolWeight:198 RetIndex:1389
CompName:4-Decenoic acid, ethyl ester, (Z)- $$ Ethyl cis-4-decenoate $$ Ethyl (4Z)-4-decenoate $$ Ethyl (Z)-4-decenoate $$

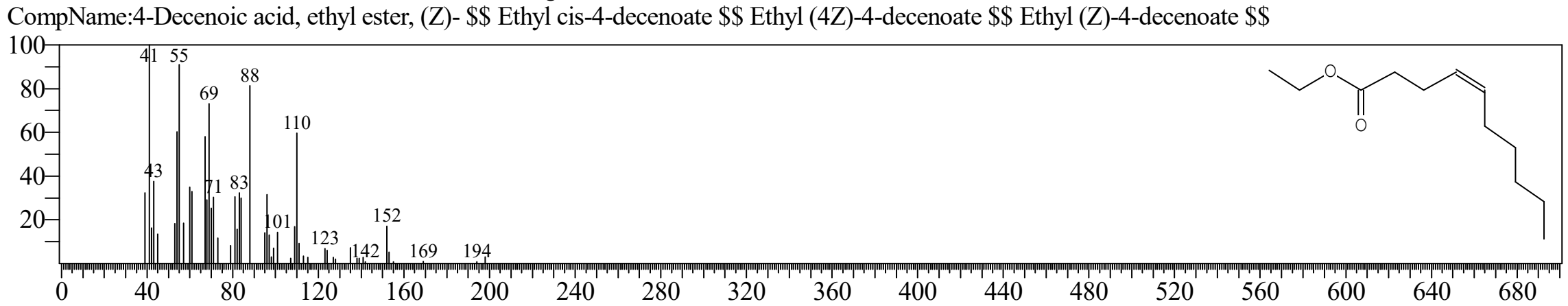

Hit#:3 Entry:67672 Library:NIST14.lib
SI:57 Formula:C14H26O2 CAS:0-00-0 MolWeight:226 RetIndex:1605
CompName:Hexanoic acid, 2-ethylcyclohexyl ester $$ 2-Ethylcyclohexyl hexanoate # $$

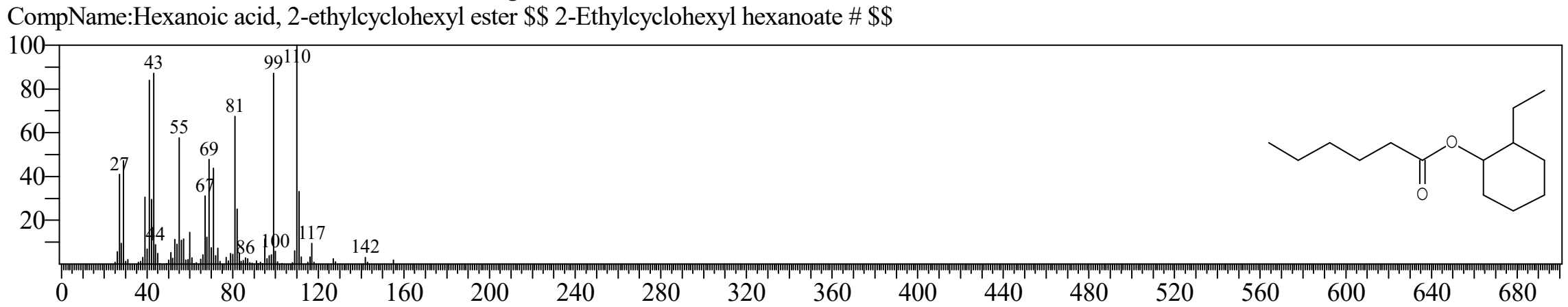

<< Target >>
Line#:148 R.Time:21.675(Scan#:3636) MassPeaks:93
RawMode:Averaged 21.670-21.680(3635-3637) BasePeak:121.10(2181)
BG Mode:Calc. from Peak Group 1 - Event 1 Scan

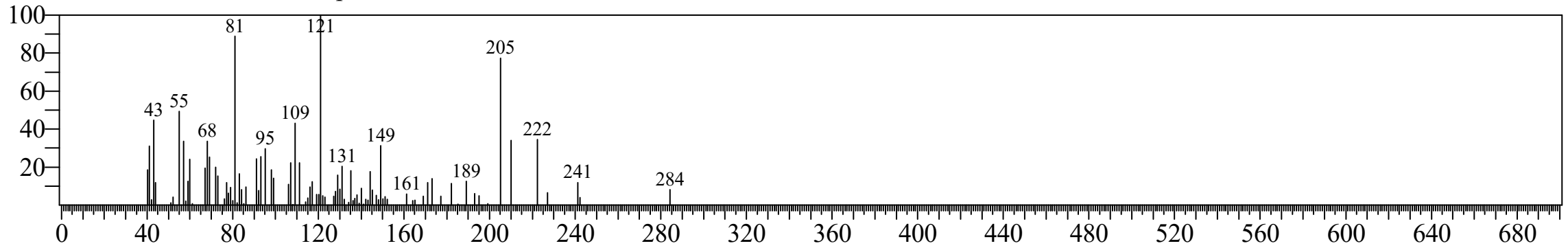

Hit#:1 Entry:160106 Library:NIST14.lib
SI:60 Formula:C21H32O3 CAS:14364-16-0 MolWeight:332 RetIndex:2206
CompName:Methyl steviol $$ Methyl 13-hydroxykaur-16-en-18-oate # $$

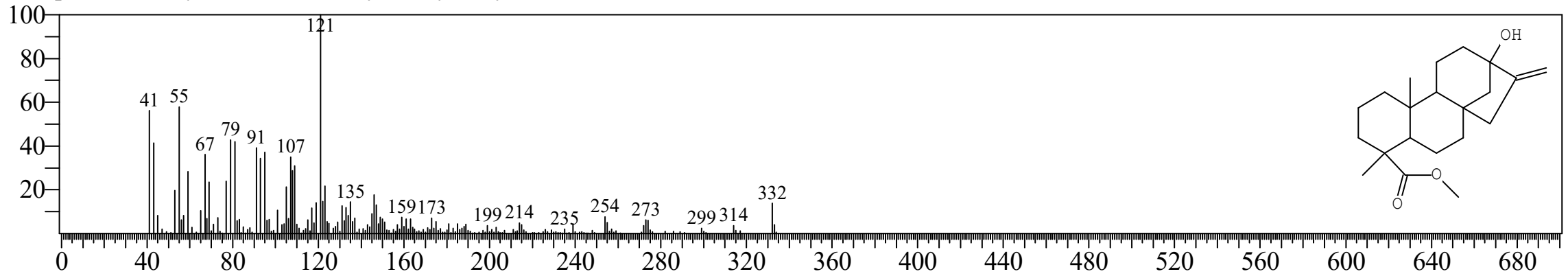

Hit#:2 Entry:342509 Library:WILEY8.LIB
SI:60 Formula:C30H52 CAS:26266-08-0 MolWeight:412 RetIndex:0
CompName:TETRACOSAPENTAENE, 2,6,10,15,19,23-HEXAMETHYL- $$ 2,6,10,15,19,23-HEXAMETHYLTETRACOSA-2,6,10,14,18-PENTAENE

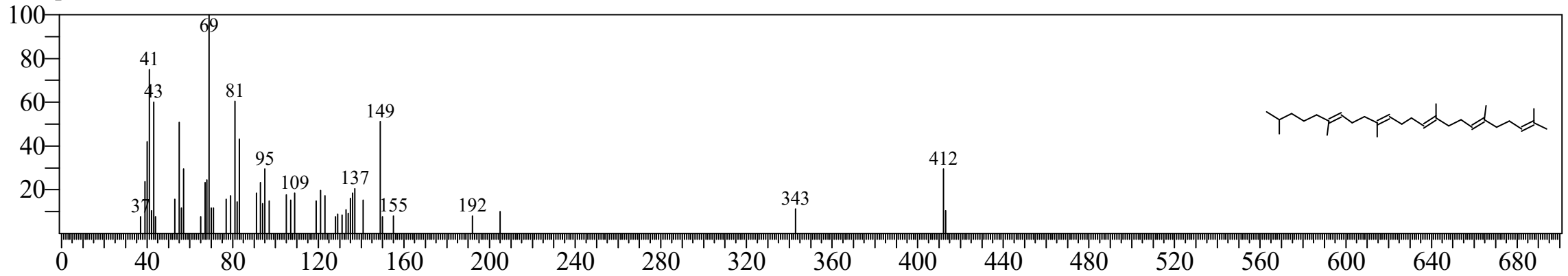

Hit#:3 Entry:182221 Library:NIST14.lib
SI:59 Formula:C22H32O4 CAS:0-00-0 MolWeight:360 RetIndex:2743
CompName:12-Hydroxy-3-keto-bisnor-4-cholenic acid $$ 4-(12-Hydroxy-3-oxogon-4-en-17-yl)pentanoic acid # $$

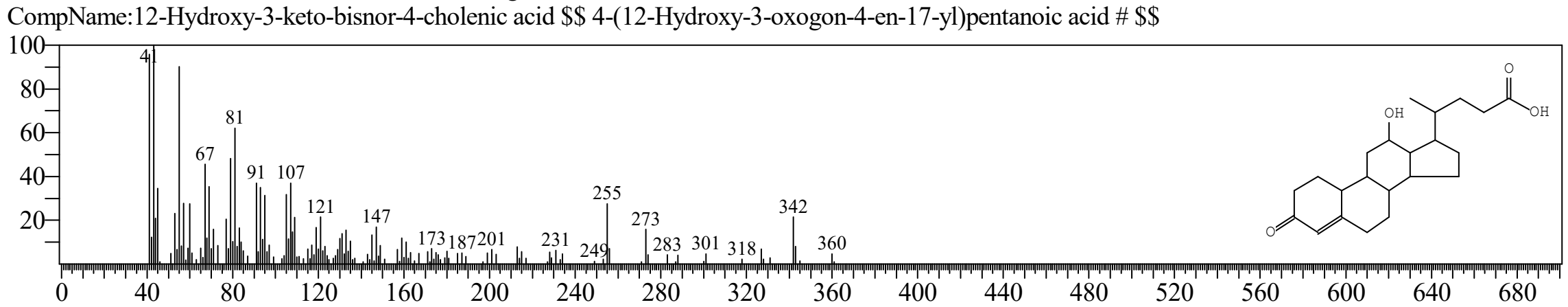

<< Target >>
Line#:149   R.Time:21.775(Scan#:3656) MassPeaks:158
RawMode:Averaged 21.770-21.780(3655-3657) BasePeak:43.05(95023)
BG Mode:Calc. from Peak Group 1 - Event 1 Scan

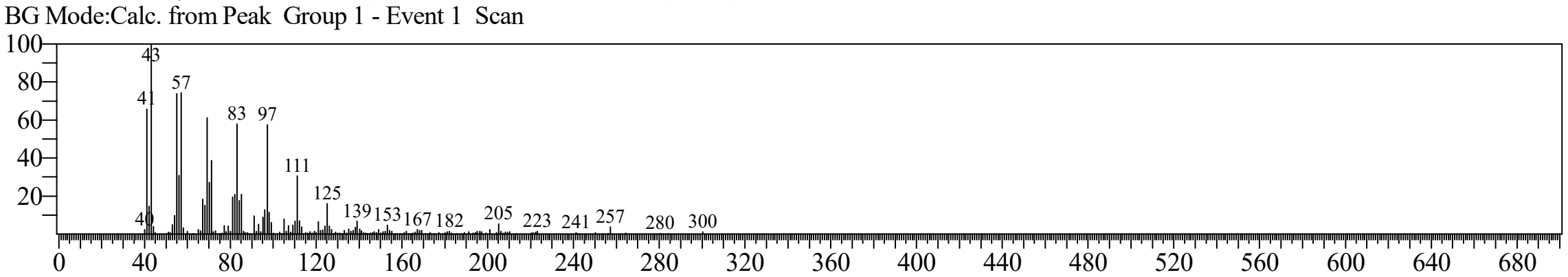

Hit#:1 Entry:307926 Library:WILEY8.LIB
SI:93 Formula:C26H52 CAS:18835-33-1 MolWeight:364 RetIndex:0
CompName:1-HEXACOSENE $$ AI3-10513 $$ EINECS 242-615-9 $$ HEXACOSENE

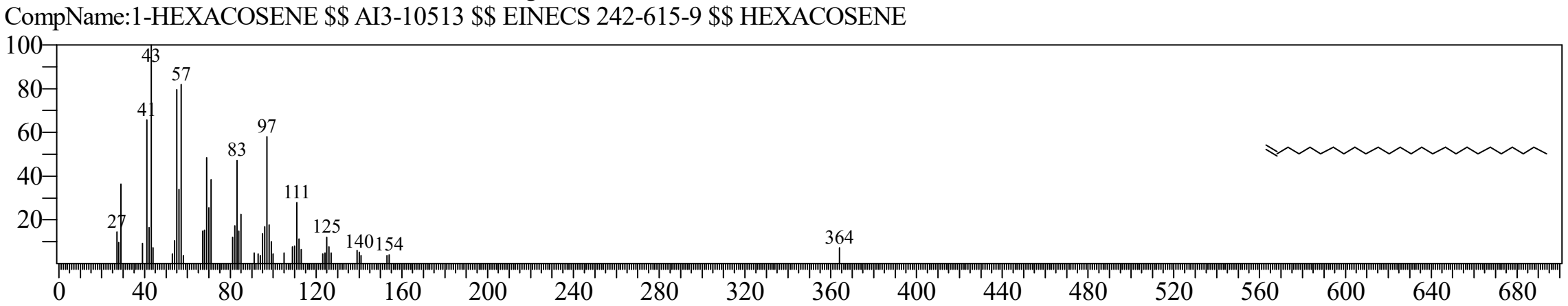

Hit#:2 Entry:185088 Library:NIST14.lib
SI:93 Formula:C26H52 CAS:18835-33-1 MolWeight:364 RetIndex:2596
CompName:1-Hexacosene

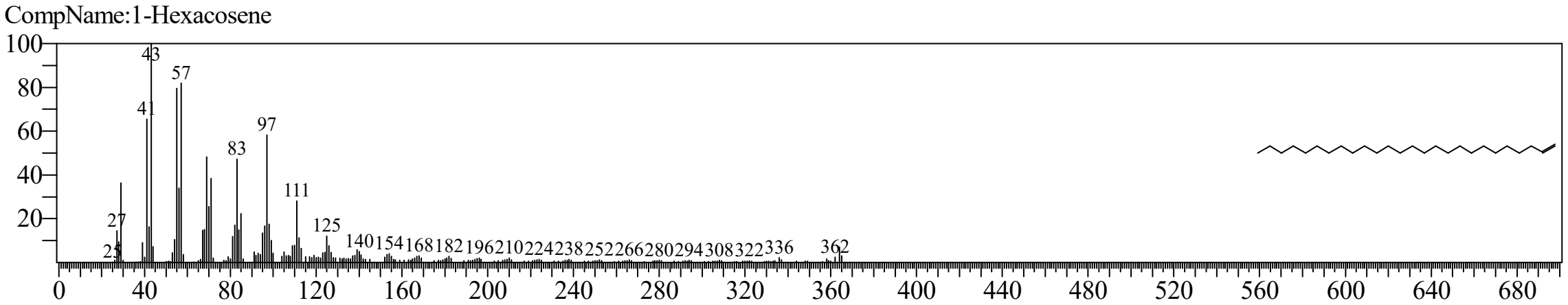

Hit#:3 Entry:28281 Library:NIST14s.lib
SI:93 Formula:C20H42O CAS:629-96-9 MolWeight:298 RetIndex:2252
CompName:1-Eicosanol $$ n-Eicosanol $$ n-1-Eicosanol $$ Arachic alcohol $$ Arachidic alcohol $$ Arachidyl alcohol $$ Eicosyl alcohol $$ 1-Icosanol $$

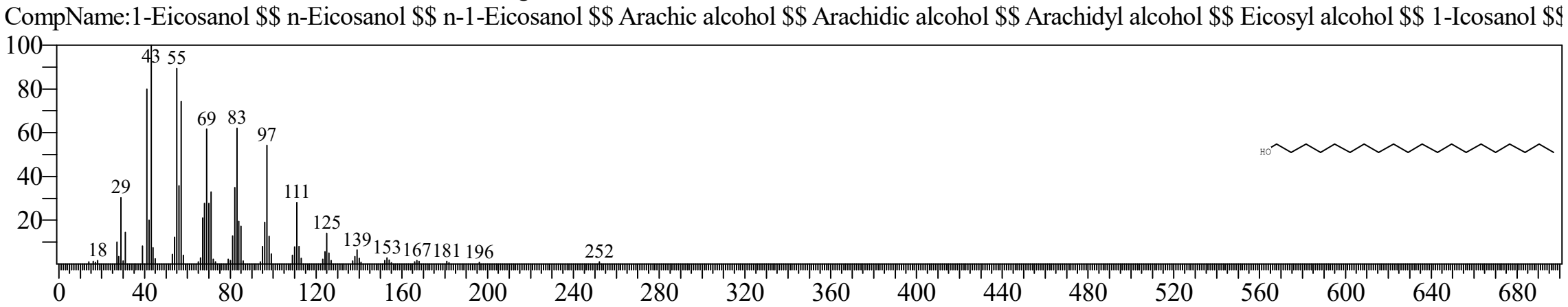

<< Target >>
Line#:150 R.Time:21.910(Scan#:3683) MassPeaks:82
RawMode:Averaged 21.905-21.915(3682-3684) BasePeak:43.05(3835)
BG Mode:Calc. from Peak Group 1 - Event 1 Scan

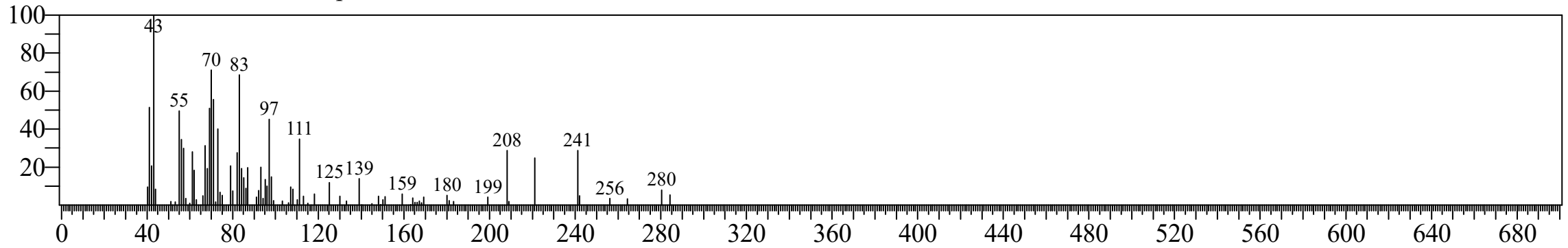

Hit#:1 Entry:144653 Library:WILEY8.LIB
SI:78 Formula:C14H28O2 CAS:112-66-3 MolWeight:228 RetIndex:0
CompName:ACETIC ACID, DODECYL ESTER $$ DODECYL ACETATE $$ 1-DODECANOL ACETATE $$ 1-DODECYL ACETATE $$ ACETATE C-

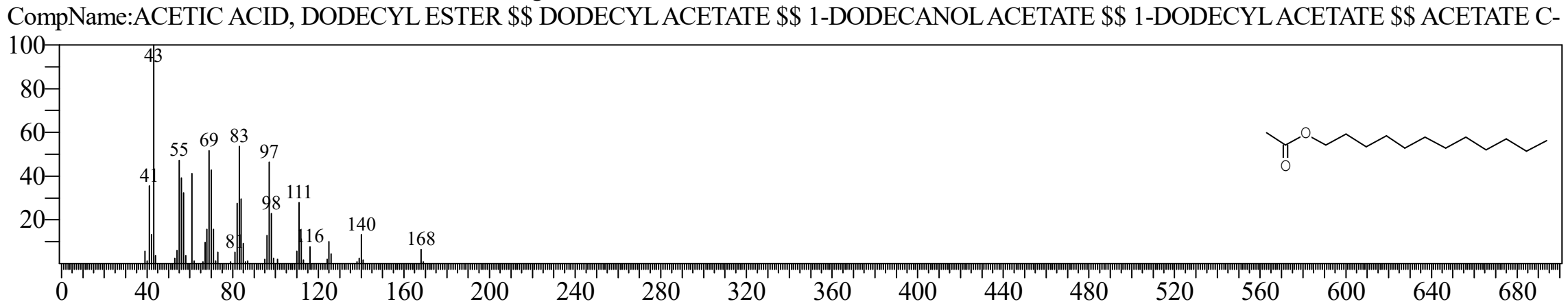

Hit#:2 Entry:104626 Library:NIST14.lib
SI:76 Formula:C17H34O2 CAS:629-58-3 MolWeight:270 RetIndex:1878
CompName:1-Pentadecanol acetate $$ Pentadecyl acetate # $$ Pentadecyl acetate $$ n-Pentadecyl acetate $$ Acetic acid, pentadecyl ester $$

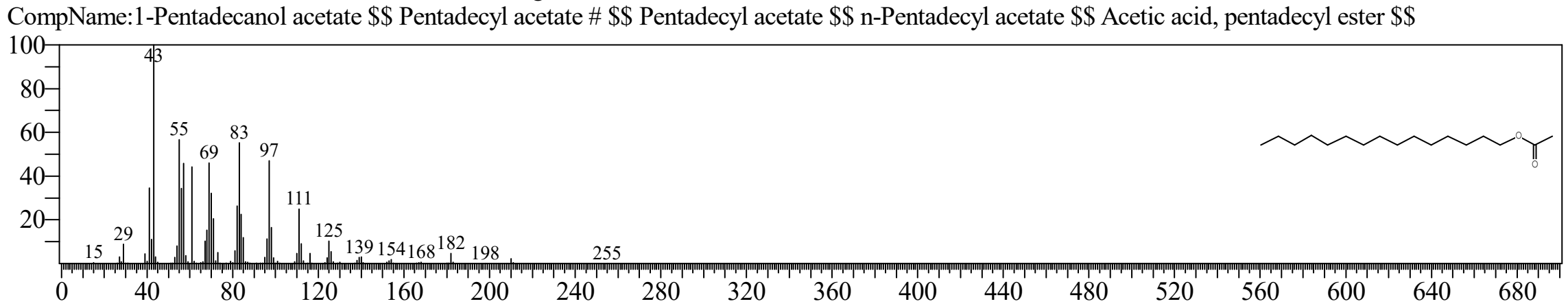

Hit#:3 Entry:22414 Library:NIST14s.lib
SI:76 Formula:C14H28O2 CAS:112-66-3 MolWeight:228 RetIndex:1580
CompName:Lauryl acetate $$ Dodecan-1-yl acetate $$ Dodecyl alcohol, acetate $$ 1-Dodecanol acetate $$ n-Dodecyl ethanoate $$ Dodecanol acetate $$ D

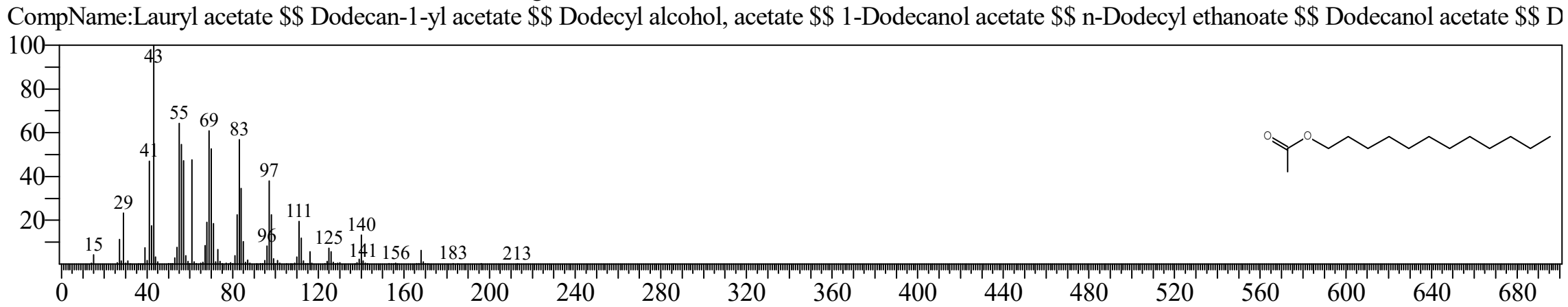

<< Target >>
Line#:151   R.Time:22.050(Scan#:3711) MassPeaks:196
RawMode:Averaged 22.045-22.055(3710-3712) BasePeak:298.15(44980)
BG Mode:Calc. from Peak Group 1 - Event 1 Scan

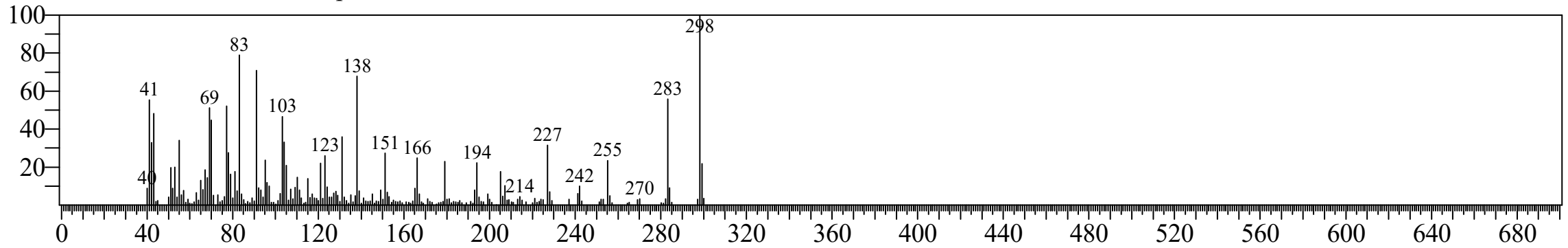

Hit#:1 Entry:129513 Library:NIST14.lib
SI:92 Formula:C18H18O4 CAS:0-00-0 MolWeight:298 RetIndex:2443
CompName:2,3-Dihydro-5-hydroxy-6,8,8-trimethyl-2-phenyl-4H-1-benzopyran-4,7(8H)-dione

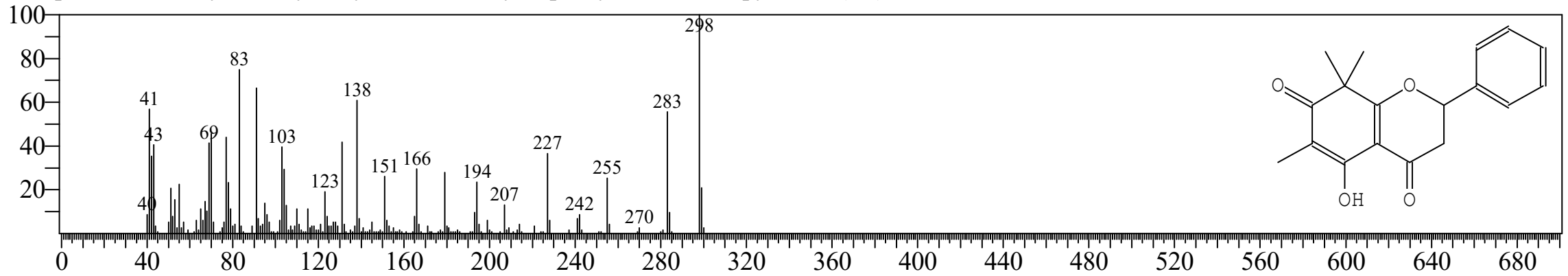

Hit#:2 Entry:24618 Library:NIST14.lib
SI:62 Formula:C11H18O CAS:54345-62-9 MolWeight:166 RetIndex:1125
CompName:1-Oxaspiro[2.5]octane, 2,4,4-trimethyl-8-methylene- $$ 2,4,4-Trimethyl-8-methylene-1-oxaspiro[2.5]octane # $$

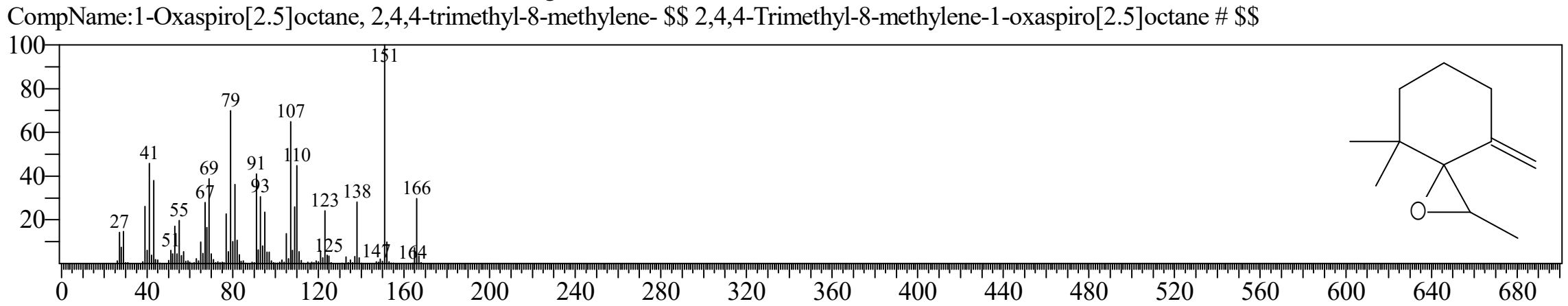

Hit#:3 Entry:60683 Library:WILEY8.LIB
SI:62 Formula:C11H18O CAS:54345-62-9 MolWeight:166 RetIndex:0
CompName:2,4,4-TRIMETHYL-8-METHYLENE-1-OXA-SPIRO[2.5]OCTANE $$ 2,4,4-TRIMETHYL-8-METHYLENE-1-OXASPIRO[2.5]OCTANE

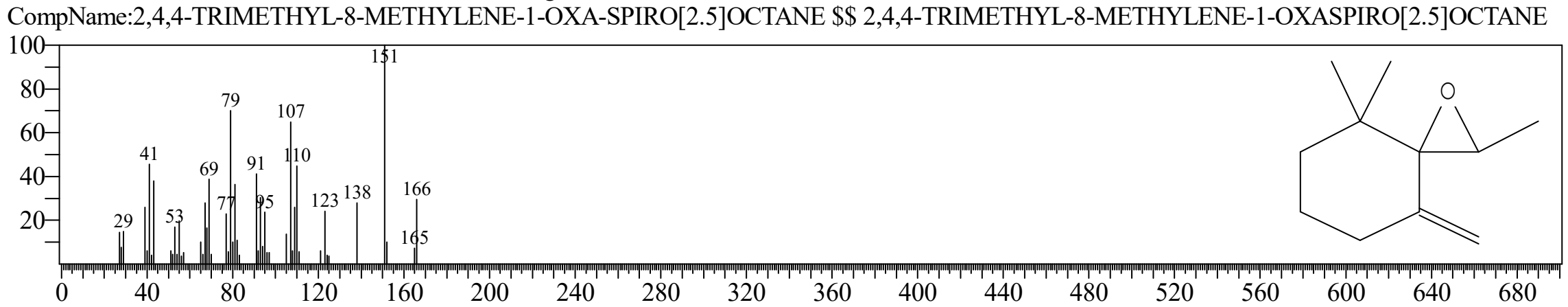

<< Target >>
Line#:152   R.Time:22.145(Scan#:3730) MassPeaks:232
RawMode:Averaged 22.140-22.150(3729-3731) BasePeak:270.15(1795059)
BG Mode:Calc. from Peak Group 1 - Event 1 Scan

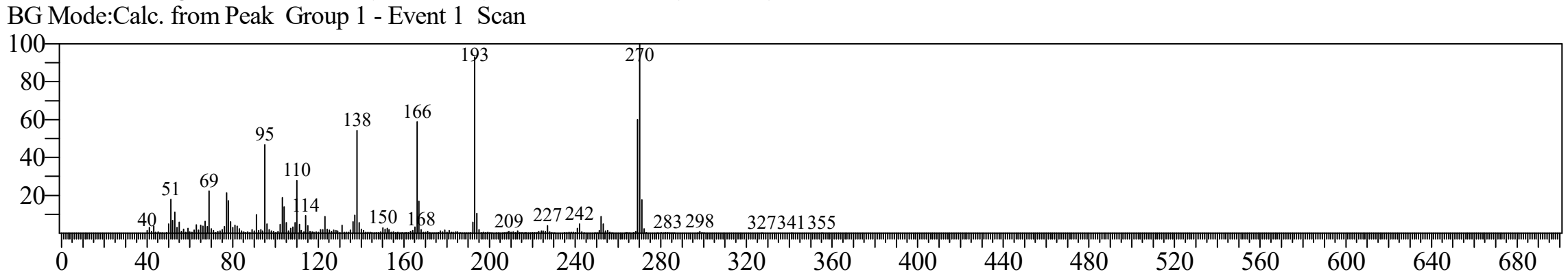

Hit#:1 Entry:104412 Library:NIST14.lib
SI:95 Formula:C16H14O4 CAS:75291-74-6 MolWeight:270 RetIndex:0
CompName:5-hydroxy-7-methoxyflavanone

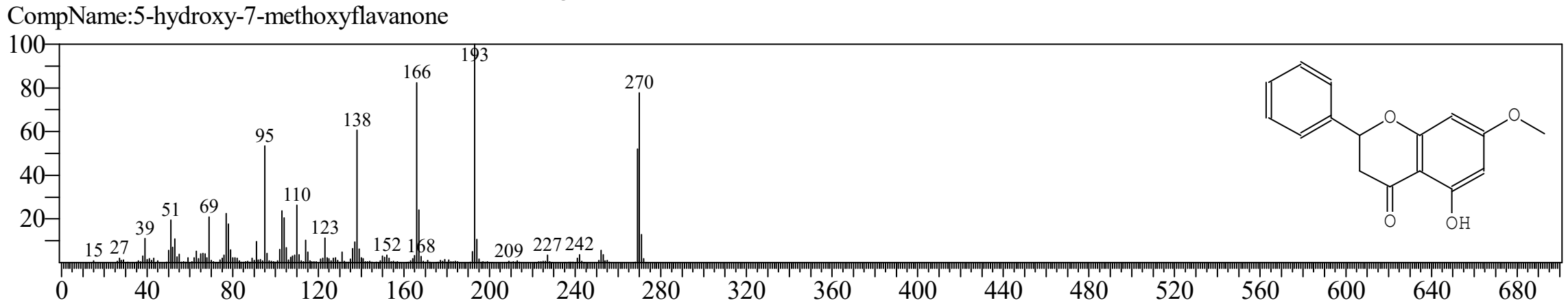

Hit#:2 Entry:104428 Library:NIST14.lib
SI:93 Formula:C16H14O4 CAS:18956-15-5 MolWeight:270 RetIndex:2440
CompName:2-Propen-1-one, 1-(2,6-dihydroxy-4-methoxyphenyl)-3-phenyl-, (E)- $$ Chalcone, 2',6'-dihydroxy-4'-methoxy- $$ Pinostrobin chalcone $$ 2-Pr

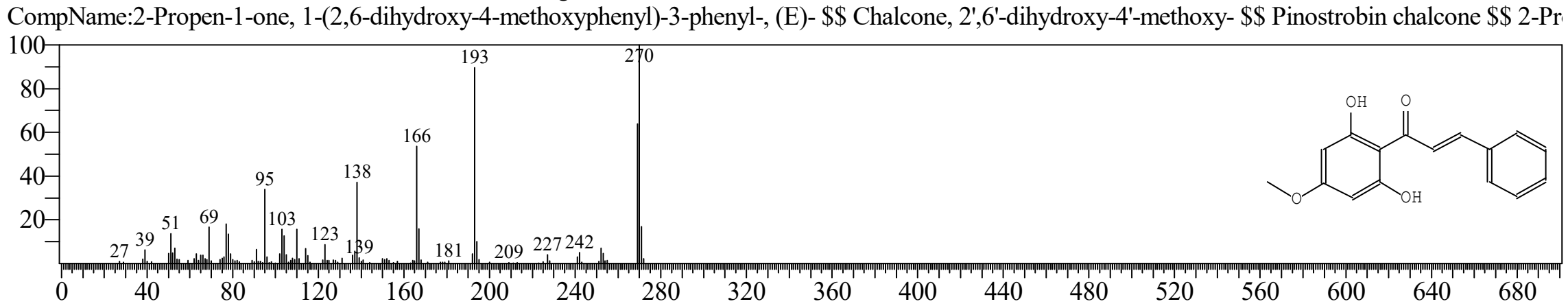

Hit#:3 Entry:104427 Library:NIST14.lib
SI:90 Formula:C16H14O4 CAS:480-37-5 MolWeight:270 RetIndex:0
CompName:4H-1-Benzopyran-4-one, 2,3-dihydro-5-hydroxy-7-methoxy-2-phenyl-, (S)- $$ 5-Hydroxy-7-methoxyflavanone $$ Pinostrobin $$ Flavanone, 5-

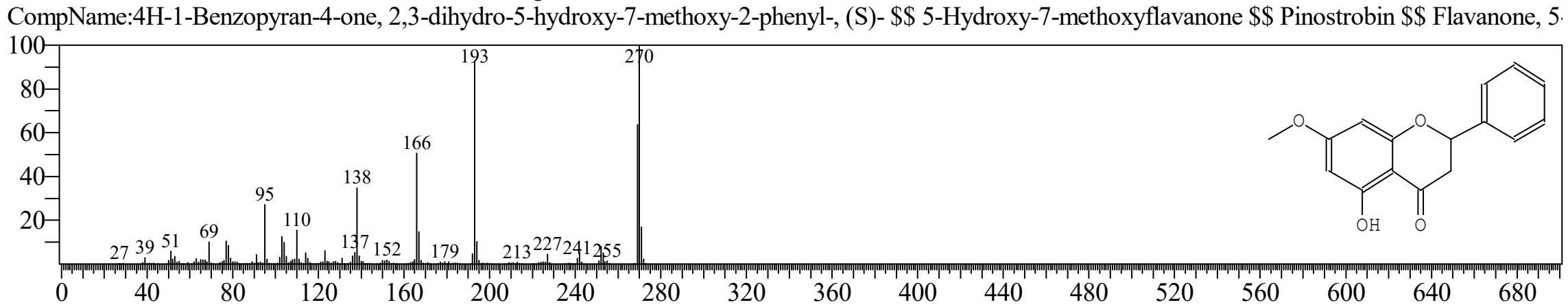

<< Target >>
Line#:153   R.Time:22.360(Scan#:3773) MassPeaks:130
RawMode:Averaged 22.355-22.365(3772-3774) BasePeak:121.10(5861)
BG Mode:Calc. from Peak Group 1 - Event 1 Scan

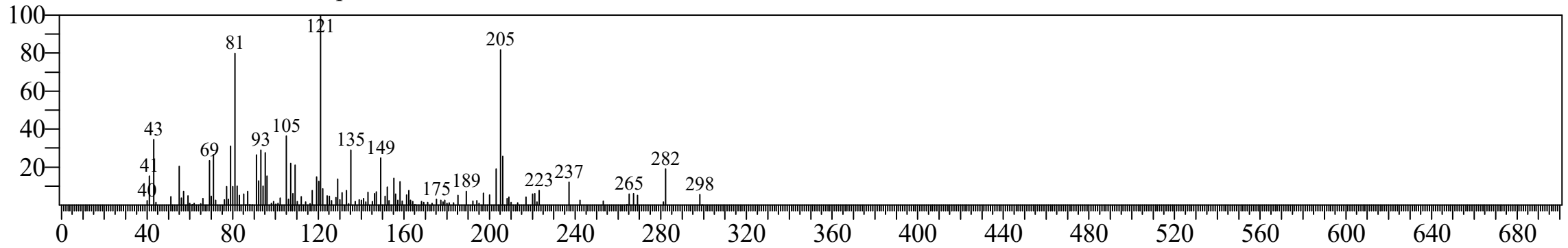

Hit#:1 Entry:64366 Library:NIST14.lib
SI:69 Formula:C15H26O CAS:481-34-5 MolWeight:222 RetIndex:1580
CompName:.alpha.-Cadinol $$ 4-Isopropyl-1,6-dimethyl-1,2,3,4,4a,7,8,8a-octahydro-1-naphthalenol # $$ (1R,4S,4aR,8aR)-4-Isopropyl-1,6-dimethyl-1,2,3,

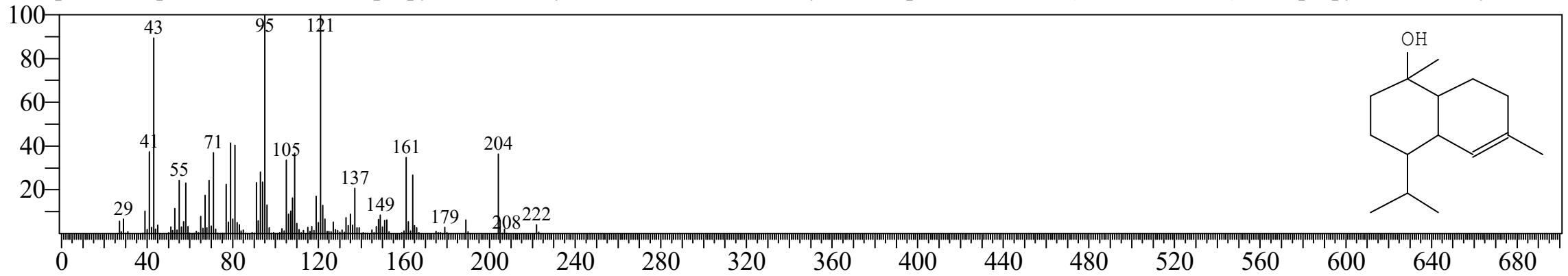

Hit#:2 Entry:64398 Library:NIST14.lib
SI:69 Formula:C15H26O CAS:0-00-0 MolWeight:222 RetIndex:1630
CompName:gleenol

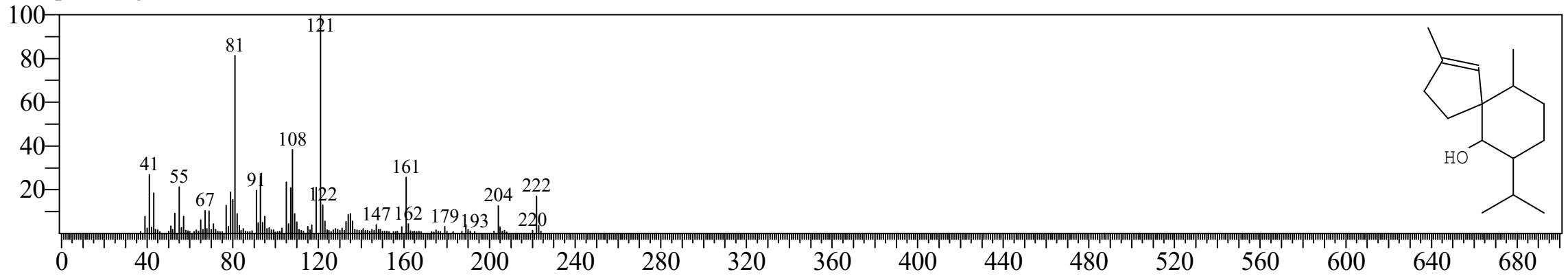

Hit#:3 Entry:341216 Library:WILEY8.LIB
SI:68 Formula:C30H50 CAS:0-00-0 MolWeight:410 RetIndex:0
CompName:2,6,6,9,2',6',6',9'-OCTAMETHYL-[8,8']BI[TRICYCLO[5.4.0.0 2,9]UNDECYL]

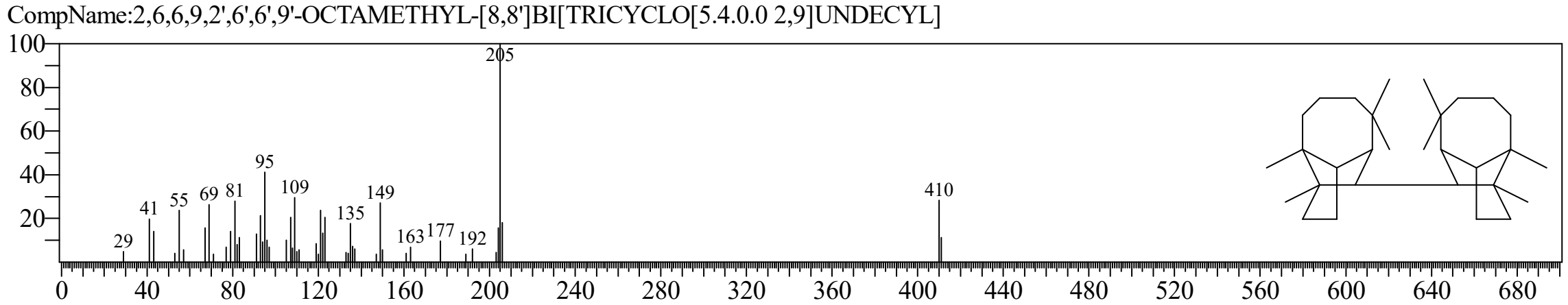

<< Target >>
Line#:154   R.Time:22.440(Scan#:3789) MassPeaks:98
RawMode:Averaged 22.435-22.445(3788-3790) BasePeak:81.05(4650)
BG Mode:Calc. from Peak Group 1 - Event 1 Scan

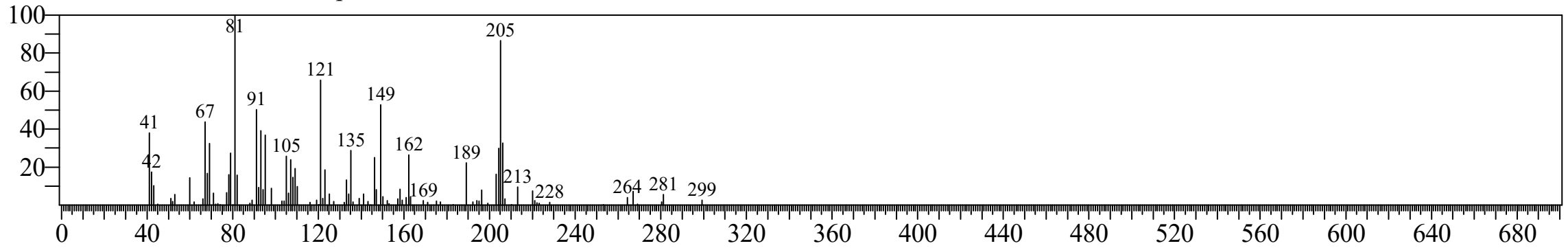

Hit#:1 Entry:276356 Library:WILEY8.LIB
SI:72 Formula:C15H25I CAS:0-00-0 MolWeight:332 RetIndex:0
CompName:<NO NAME>

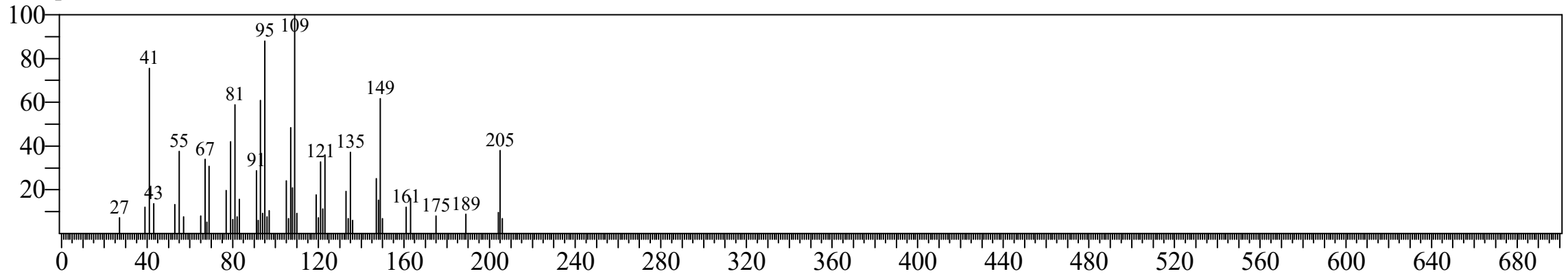

Hit#:2 Entry:159448 Library:NIST14.lib
SI:72 Formula:C15H25I CAS:0-00-0 MolWeight:332 RetIndex:1831
CompName:6.beta.Bicyclo[4.3.0]nonane, 5.beta.-iodomethyl-1.beta.-isopropenyl-4.alpha.,5.alpha.-dimethyl-, $$ 7-(Iodomethyl)-3a-isopropenyl-6,7-dimethy

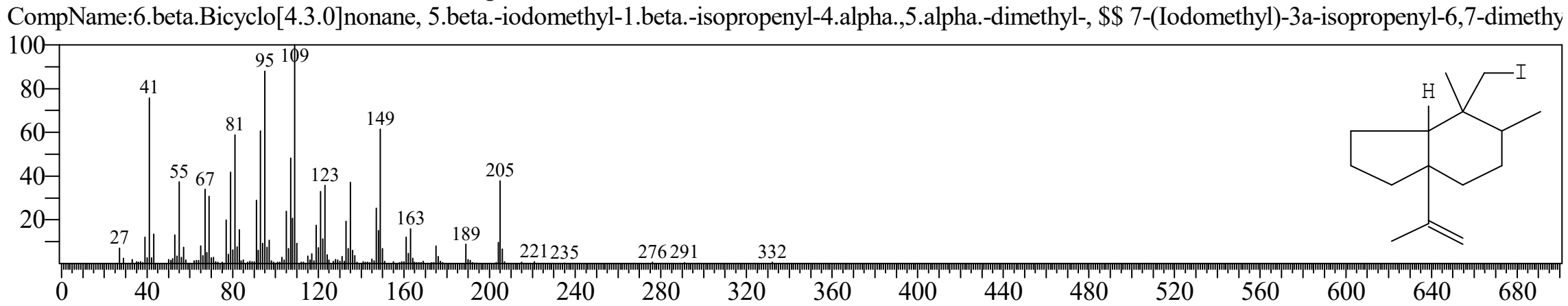

Hit#:3 Entry:274951 Library:WILEY8.LIB
SI:69 Formula:C21H30OS CAS:0-00-0 MolWeight:330 RetIndex:0
CompName:(7A-ISOPROPENYL-4,5-DIMETHYLOCTAHYDRO-1H-INDEN-4-YL)METHYL PHENYL SULFOXIDE $$ 3A-ISOPROPENYL-6,7-DIM

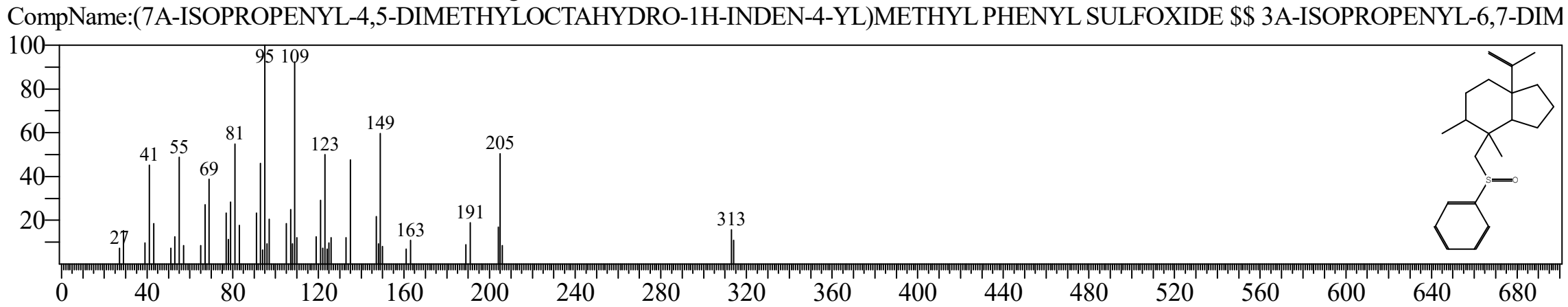

<< Target >>
Line#:155   R.Time:22.590(Scan#:3819) MassPeaks:136
RawMode:Averaged 22.585-22.595(3818-3820) BasePeak:43.05(10152)
BG Mode:Calc. from Peak Group 1 - Event 1 Scan

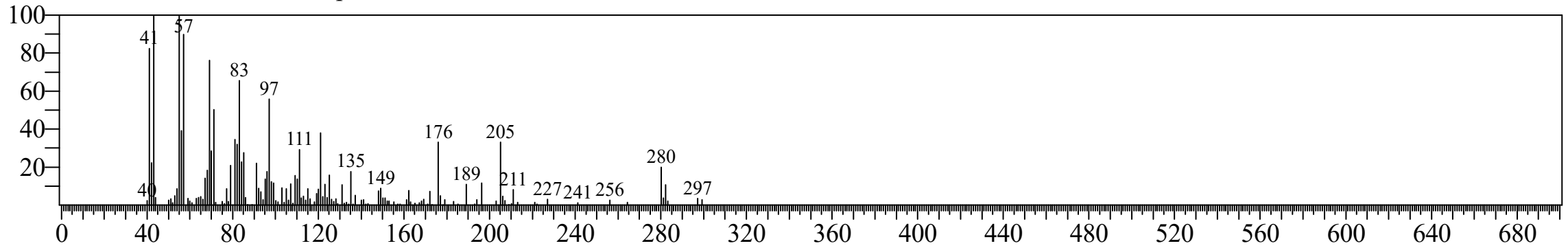

Hit#:1 Entry:183235 Library:NIST14.lib
SI:81 Formula:C18H35BrO2 CAS:5454-48-8 MolWeight:362 RetIndex:2274
CompName:Bromoacetic acid, hexadecyl ester $$ Hexadecyl bromoacetate # $$

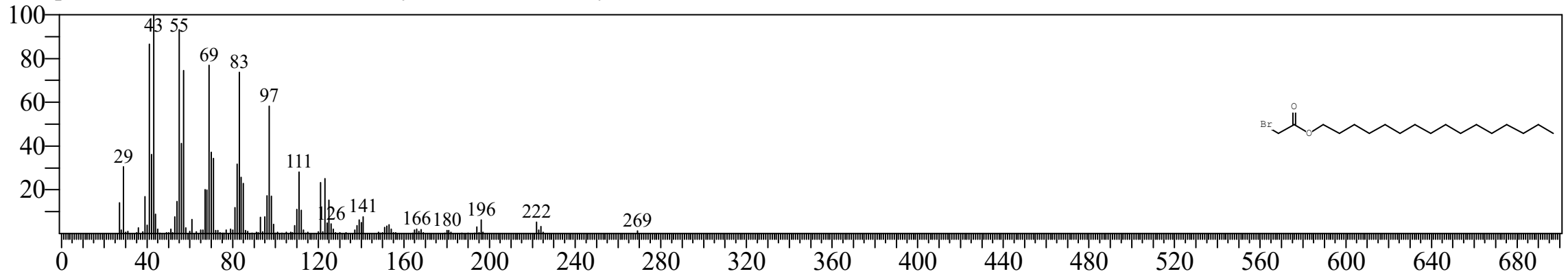

Hit#:2 Entry:200895 Library:NIST14.lib
SI:81 Formula:C20H39BrO2 CAS:18992-03-5 MolWeight:390 RetIndex:2473
CompName:Bromoacetic acid, octadecyl ester $$ Octadecyl bromoacetate # $$

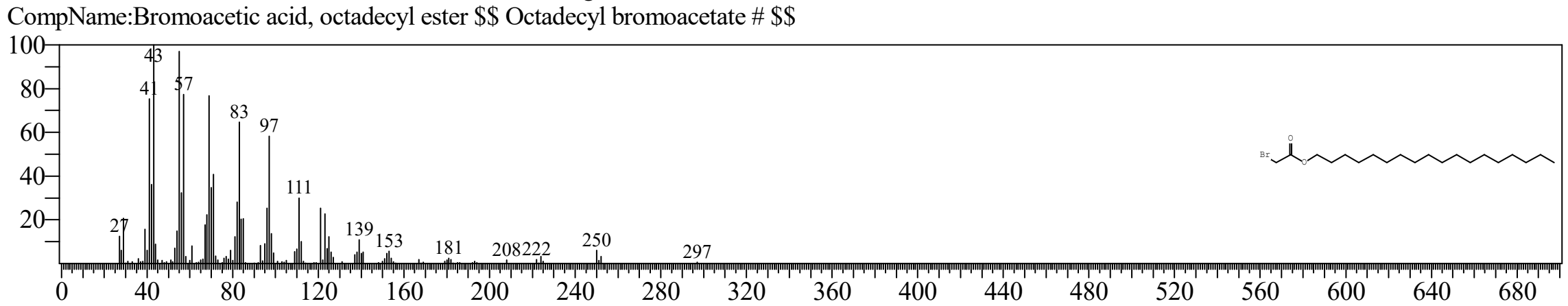

Hit#:3 Entry:194365 Library:NIST14.lib
SI:80 Formula:C25H46O2 CAS:0-00-0 MolWeight:378 RetIndex:0
CompName:Undec-10-ynoic acid, tetradecyl ester

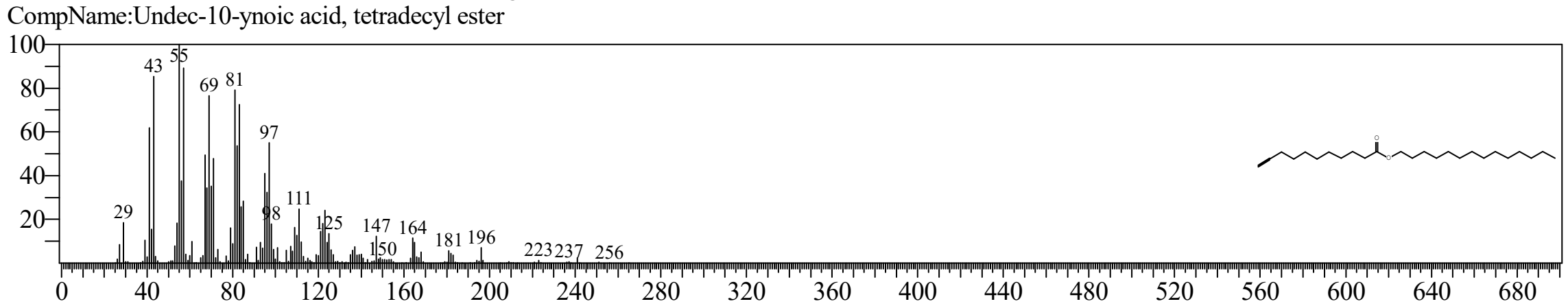

<< Target >>
Line#:156   R.Time:22.675(Scan#:3836) MassPeaks:159
RawMode:Averaged 22.670-22.680(3835-3837) BasePeak:205.20(10541)
BG Mode:Calc. from Peak Group 1 - Event 1 Scan

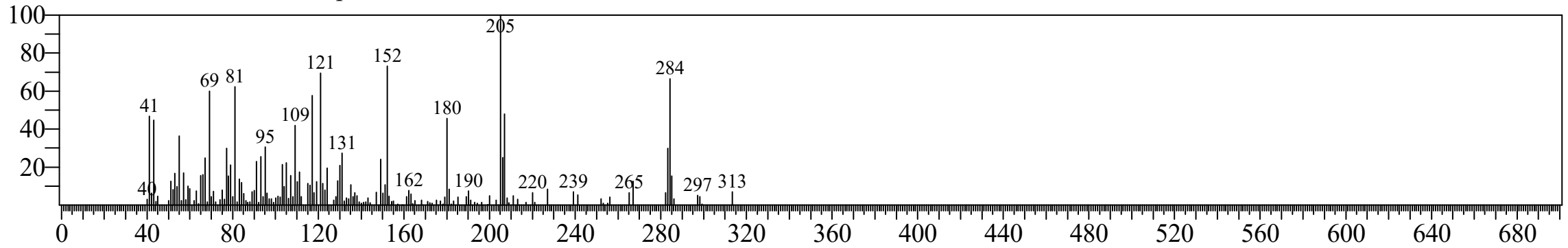

Hit#:1 Entry:85418 Library:NIST14.lib
SI:63 Formula:C15H20O3 CAS:65563-75-9 MolWeight:248 RetIndex:1690
CompName:Alantolactone, 4.alpha.,4A.alpha.-epoxy- $$ Naphtho[2,3-b]furan-2-one, perhydro-4,4a-epoxy-3-methylene-5,8a-dimethyl- $$ 2,5a-Dimethyl-9-

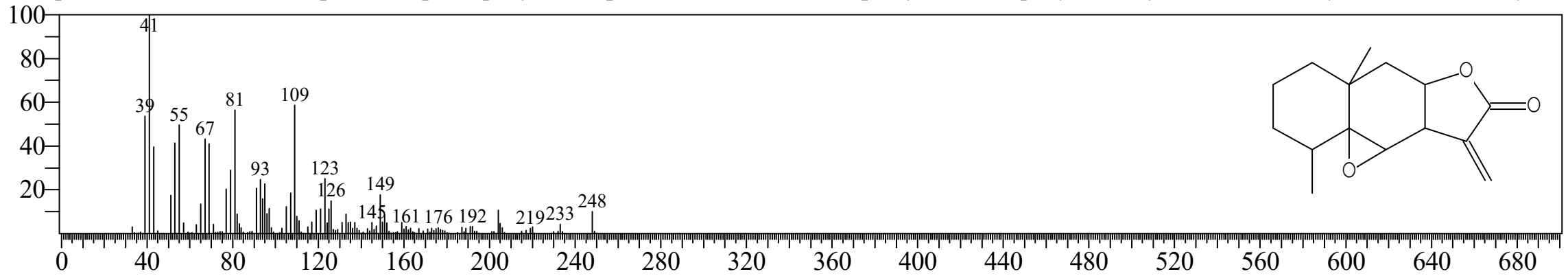

Hit#:2 Entry:171792 Library:WILEY8.LIB
SI:63 Formula:C15H20O3 CAS:65563-75-9 MolWeight:248 RetIndex:0
CompName:2,5A-DIMETHYL-9-METHYLENEOCTAHYDRO-2H-OXIRENO[2',3':4,4A]NAPHTHO[2,3-B]FURAN-8(9H)-ONE # $$ 2,5A-DIMETHY

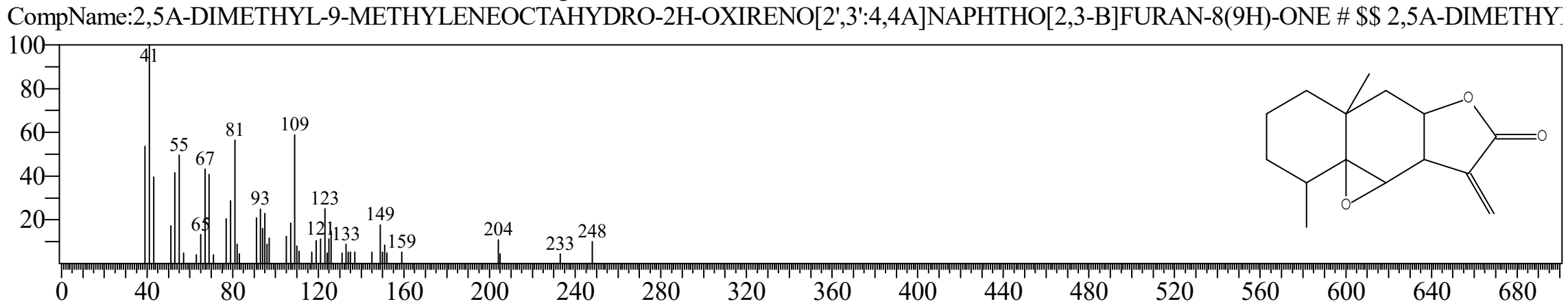

Hit#:3 Entry:75642 Library:NIST14.lib
SI:61 Formula:C16H28O CAS:137235-47-3 MolWeight:236 RetIndex:1721
CompName:2-Hydroxymethyl-2,6,8,8-tetramethyltricyclo[5.2.2.0(1,6)]undecane

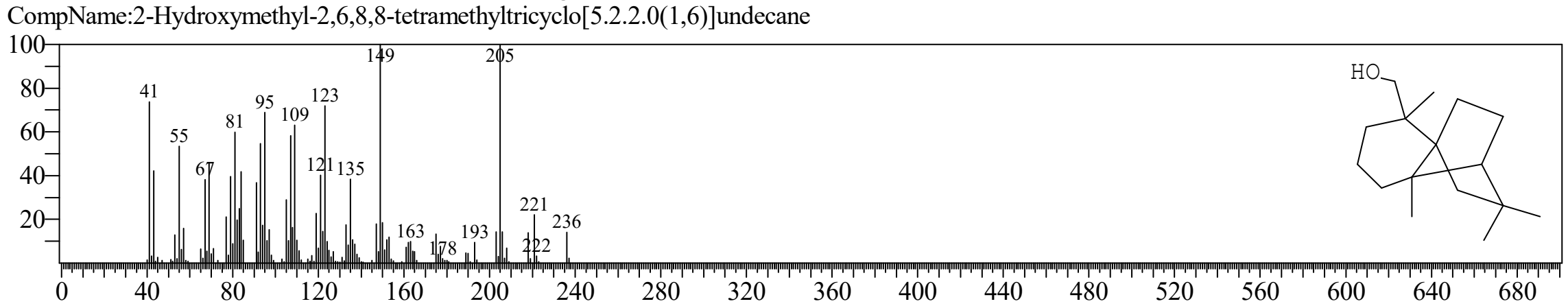

<< Target >>
Line#:157 R.Time:22.750(Scan#:3851) MassPeaks:136
RawMode:Averaged 22.745-22.755(3850-3852) BasePeak:205.20(11532)
BG Mode:Calc. from Peak Group 1 - Event 1 Scan

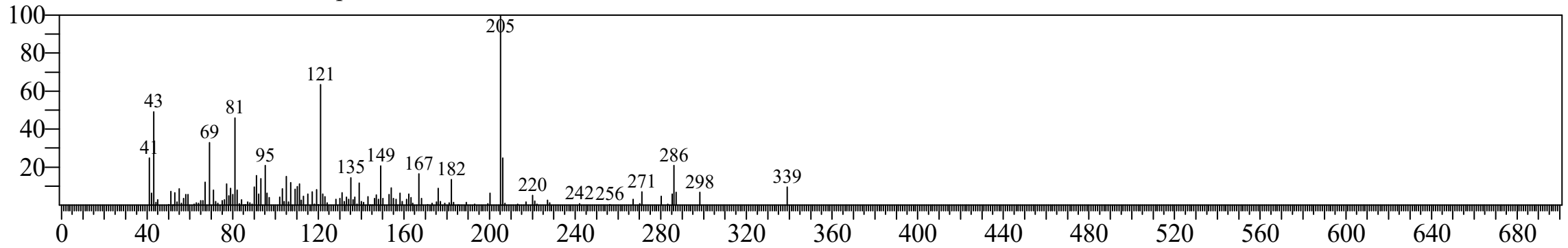

Hit#:1 Entry:341216 Library:WILEY8.LIB
SI:66 Formula:C30H50 CAS:0-00-0 MolWeight:410 RetIndex:0
CompName:2,6,6,9,2',6',6',9'-OCTAMETHYL-[8,8']BI[TRICYCLO[5.4.0.0 2,9]UNDECYL]

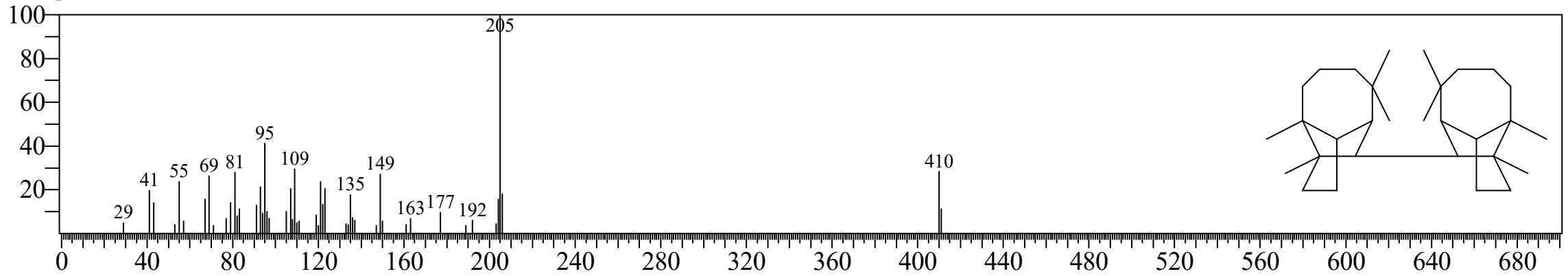

Hit#:2 Entry:210644 Library:NIST14.lib
SI:66 Formula:C30H50 CAS:0-00-0 MolWeight:410 RetIndex:2589
CompName:2,6,6,9,2',6',6',9'-Octamethyl-[8,8']bi[tricyclo[5.4.0.0(2,9)]undecyl]

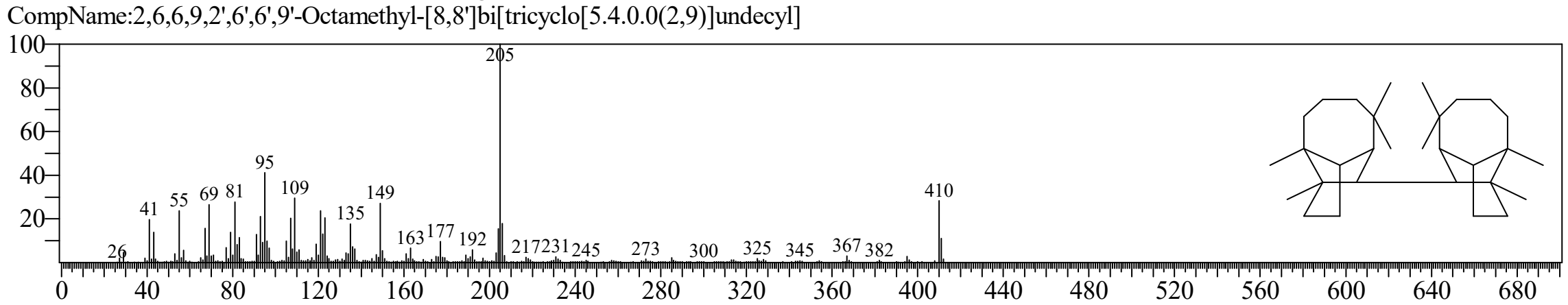

Hit#:3 Entry:188196 Library:WILEY8.LIB
SI:63 Formula:C17H24O2 CAS:89902-28-3 MolWeight:260 RetIndex:0
CompName:2(5H)-FURANONE, 4-METHYL-3,5,5-TRIS(2-METHYL-2-PROPENYL)- $$ 4-METHYL-3,5,5-TRIS(2-METHYL-2-PROPENYL)-2(5H)-I

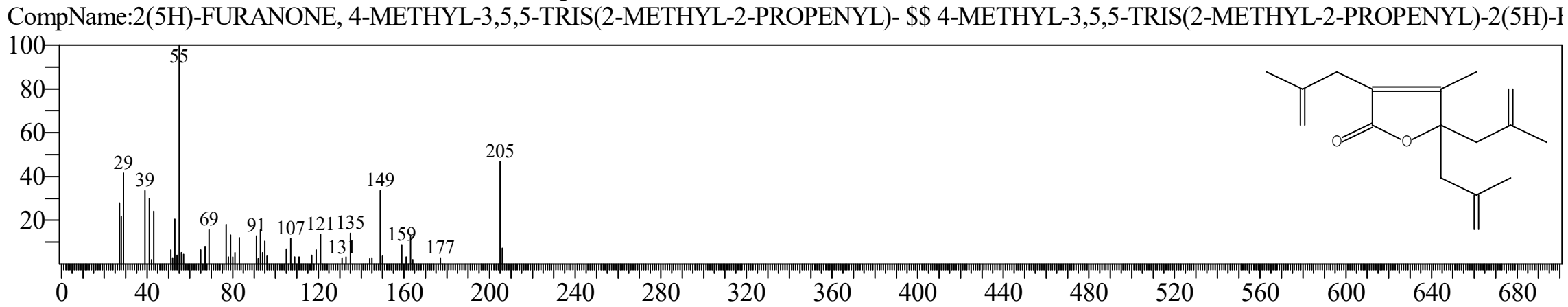

<< Target >>
Line#:158 R.Time:22.840(Scan#:3869) MassPeaks:171
RawMode:Averaged 22.835-22.845(3868-3870) BasePeak:166.10(21397)
BG Mode:Calc. from Peak Group 1 - Event 1 Scan

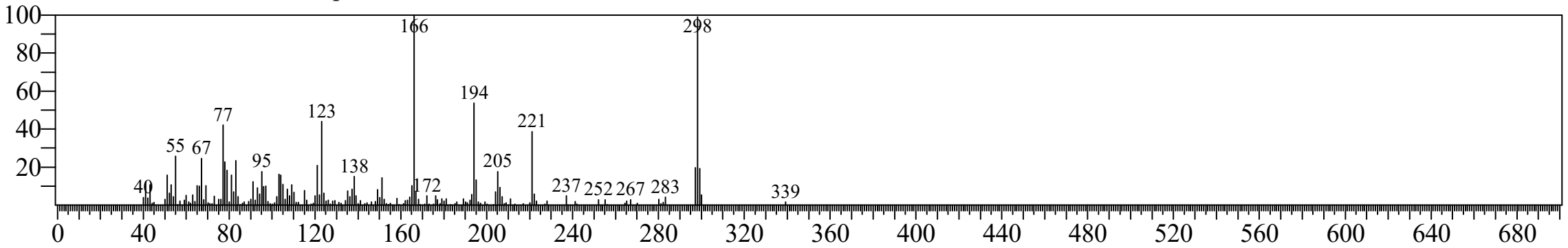

Hit#:1 Entry:59903 Library:WILEY8.LIB
SI:62 Formula:C10H14O2 CAS:42199-01-9 MolWeight:166 RetIndex:0
CompName:5H-INDEN-5-ONE, 1,2,3,6,7,7A-HEXAHYDRO-1-HYDROXY-7A-METHYL-, CIS- $$ 1.BETA.-HYDROXY-7A.BETA.-METHYL-2,3,7,7/

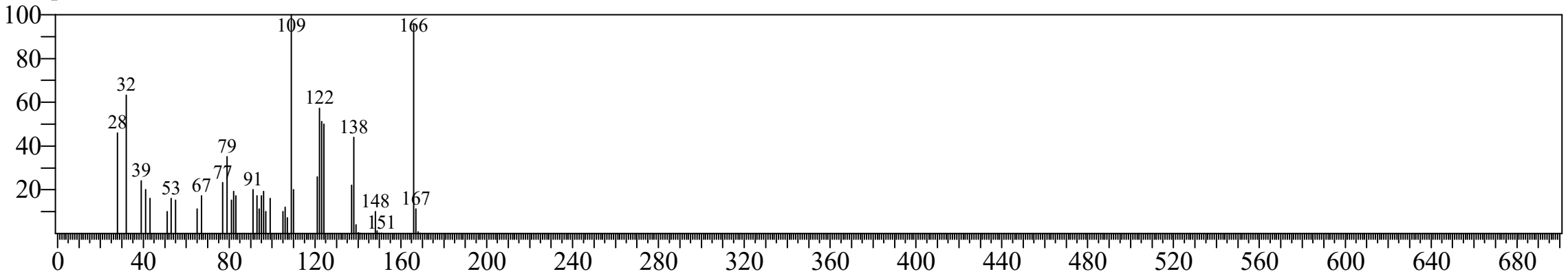

Hit#:2 Entry:95738 Library:WILEY8.LIB
SI:62 Formula:C12H18O2 CAS:0-00-0 MolWeight:194 RetIndex:0
CompName:8A-ETHYLHEXAHYDRO-1,8(2H,5H)-NAPHTHALENEDIONE

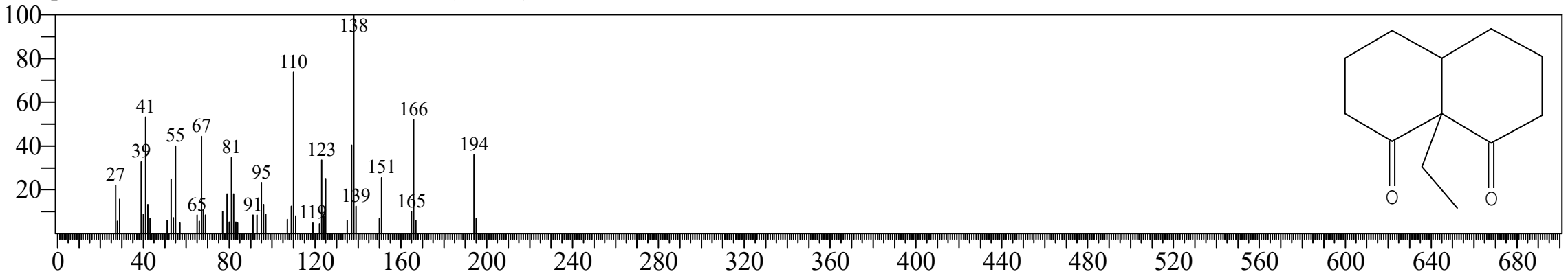

Hit#:3 Entry:42710 Library:NIST14.lib
SI:62 Formula:C12H18O2 CAS:0-00-0 MolWeight:194 RetIndex:1617
CompName:1,8-Naphthalenedione, 8a-ethylperhydro $$ 8a-Ethylhexahydro-1,8(2H,5H)-naphthalenedione # $$

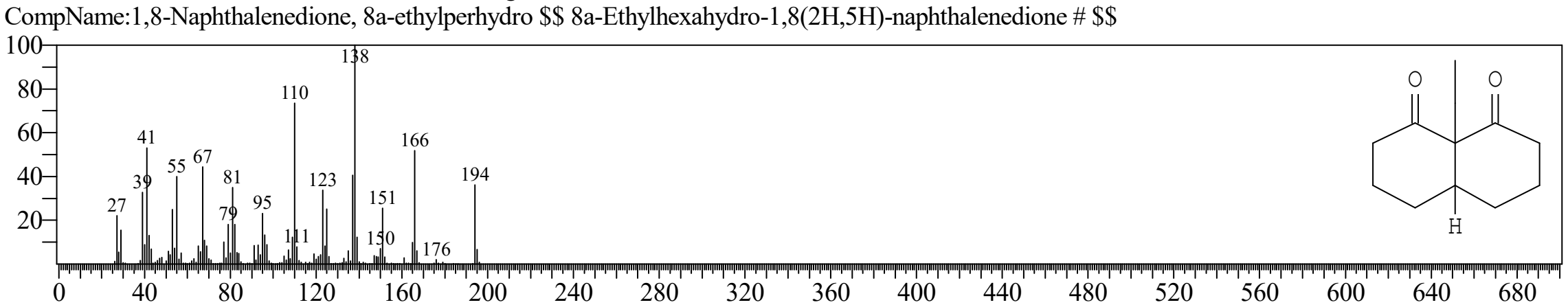

<< Target >>
Line#:159 R.Time:22.955(Scan#:3892) MassPeaks:250
RawMode:Averaged 22.950-22.960(3891-3893) BasePeak:284.15(2240222)
BG Mode:Calc. from Peak Group 1 - Event 1 Scan

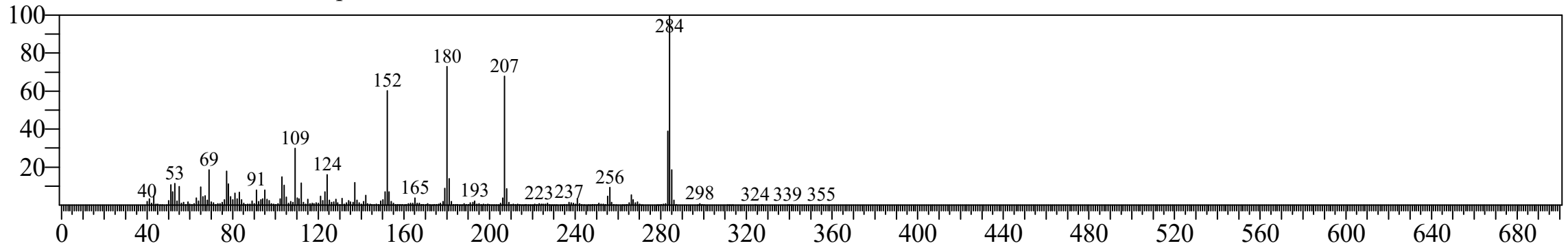

Hit#:1 Entry:116883 Library:NIST14.lib
SI:74 Formula:C17H16O4 CAS:1036-72-2 MolWeight:284 RetIndex:2340
CompName:4H-1-Benzopyran-4-one, 2,3-dihydro-5,7-dimethoxy-2-phenyl- $$ Flavanone, 5,7-dimethoxy- $$ 5,7-Dimethoxyflavanone $$ 5,7-Dimethoxy-2-

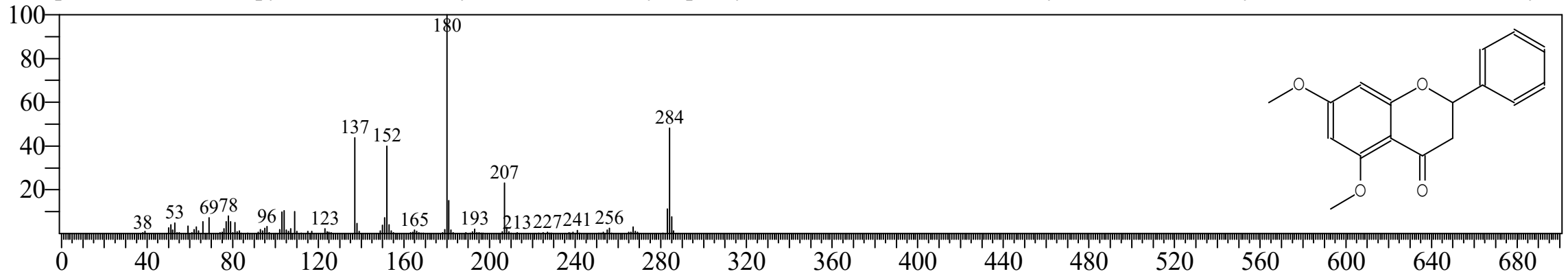

Hit#:2 Entry:116888 Library:NIST14.lib
SI:70 Formula:C17H16O4 CAS:1775-97-9 MolWeight:284 RetIndex:0
CompName:(E)-1-(2-Hydroxy-4,6-dimethoxyphenyl)-3-phenylprop-2-en-1-one $$ 2-Propen-1-one, 1-(2-hydroxy-4,6-dimethoxyphenyl)-3-phenyl-, (2E)- $$

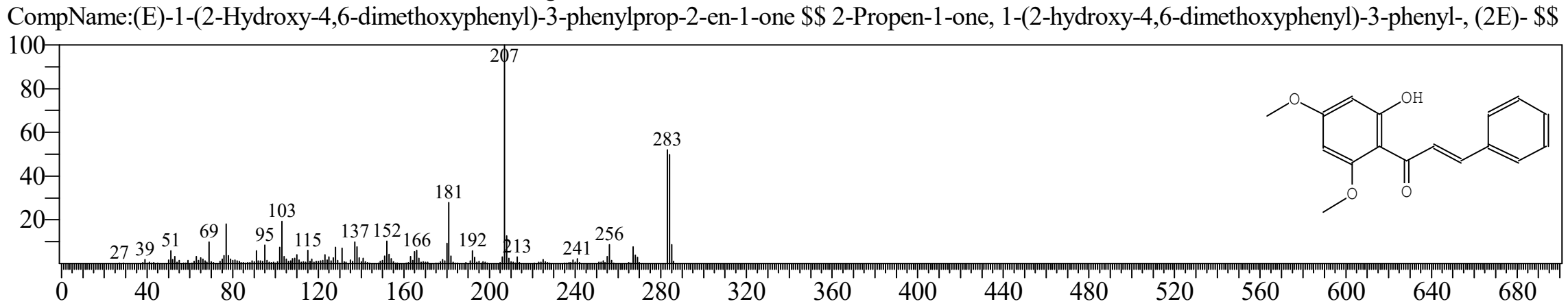

Hit#:3 Entry:219860 Library:WILEY8.LIB
SI:66 Formula:C17H16O4 CAS:1036-72-2 MolWeight:284 RetIndex:0
CompName:4H-1-BENZOPYRAN-4-ONE, 2,3-DIHYDRO-5,7-DIMETHOXY-2-PHENYL- $$ 5,7-DIMETHOXY-2-PHENYL-2,3-DIHYDRO-4H-CHRO

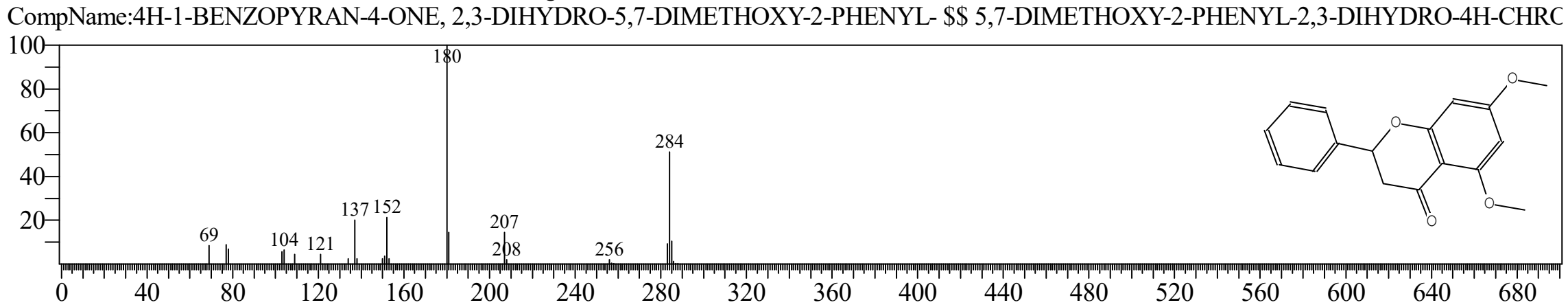

<< Target >>
Line#:160   R.Time:23.140(Scan#:3929) MassPeaks:166
RawMode:Averaged 23.135-23.145(3928-3930) BasePeak:195.10(4574663)
BG Mode:Calc. from Peak Group 1 - Event 1 Scan

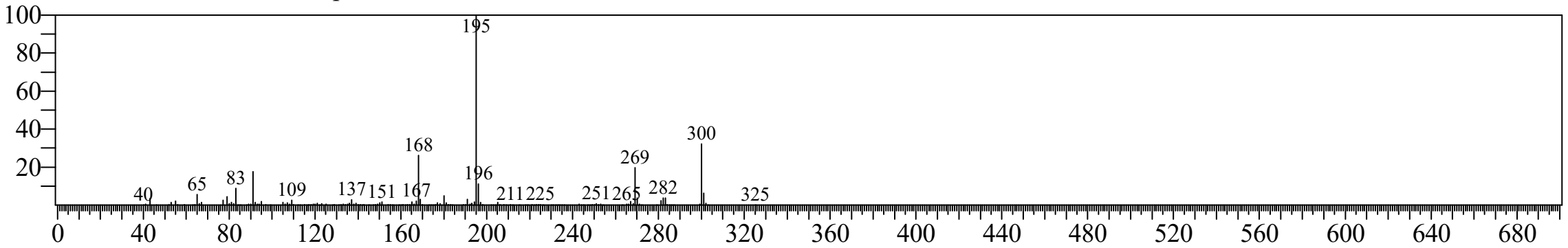

Hit#:1 Entry:240417 Library:WILEY8.LIB
SI:72 Formula:C20H16N2O CAS:1235-28-5 MolWeight:300 RetIndex:0
CompName:1H-BENZIMIDAZOL-2-YL(DIPHENYL)METHANOL # $$ (1H-BENZOIMIDAZOL-2-YL)-DIPHENYL-METHANOL $$ 1H-BENZIMID

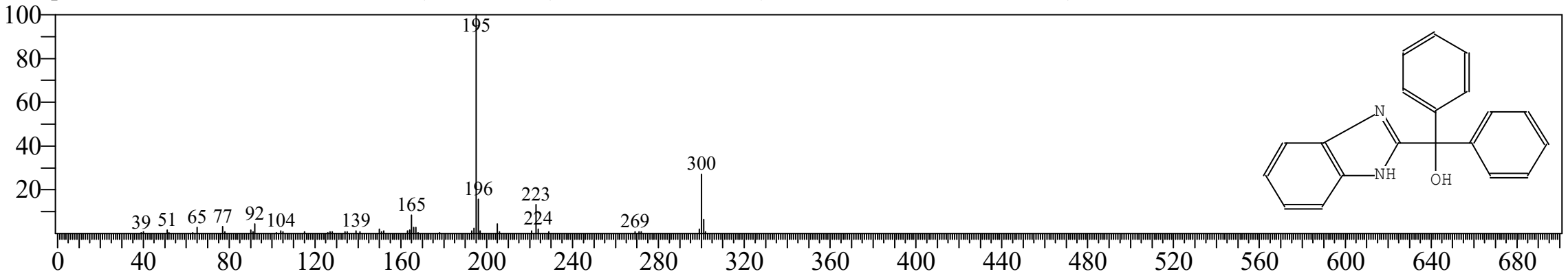

Hit#:2 Entry:131658 Library:NIST14.lib
SI:72 Formula:C20H16N2O CAS:1235-28-5 MolWeight:300 RetIndex:2799
CompName:(1H-Benzoimidazol-2-yl)-diphenyl-methanol $$ 1H-Benzimidazol-2-yl(diphenyl)methanol # $$

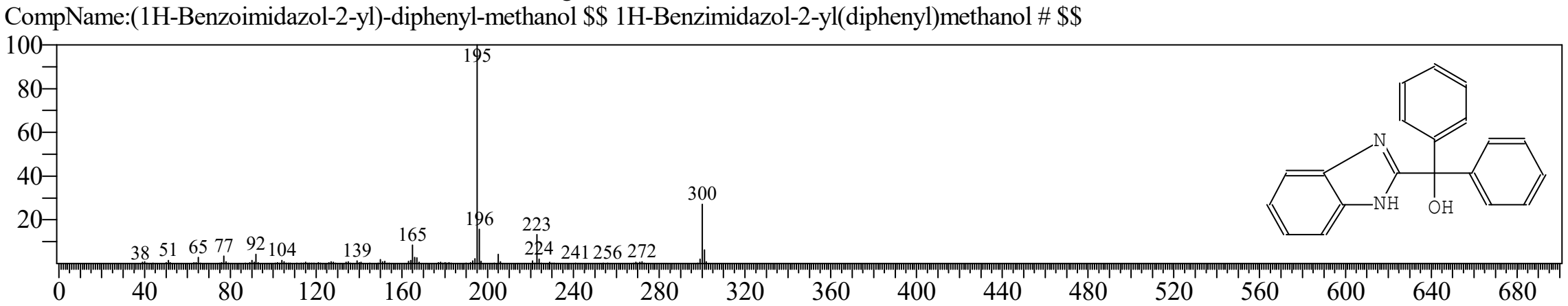

Hit#:3 Entry:239764 Library:WILEY8.LIB
SI:70 Formula:C16H20N4O2 CAS:0-00-0 MolWeight:300 RetIndex:0
CompName:1,3-DIMETHYL-6-(1-PHENYLETHYL)-5,6,7,8-TETRAHYDROPYRIMIDO[4,5-D]PYRIMIDINE-2,4(1H,3H)-DIONE $$ 1H-PYRIMIDO[

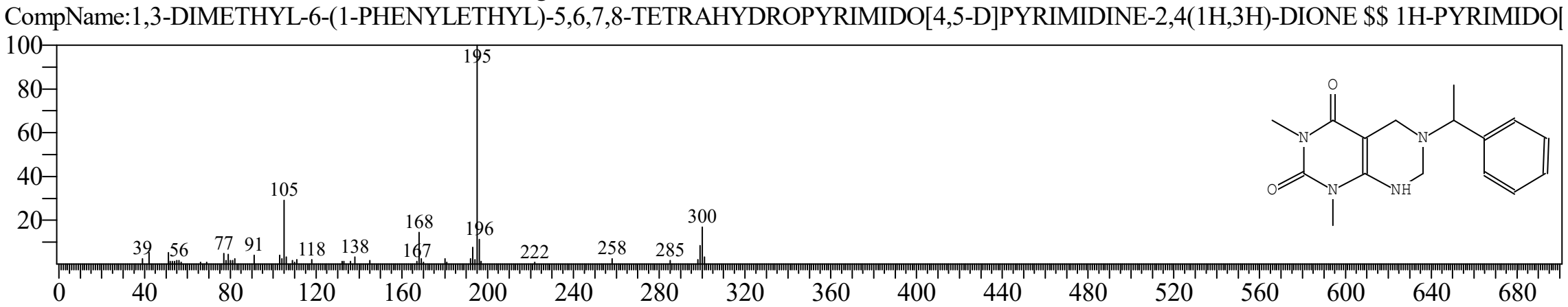

<< Target >>
Line#:161   R.Time:23.450(Scan#:3991) MassPeaks:116
RawMode:Averaged 23.445-23.455(3990-3992) BasePeak:256.10(2147373)
BG Mode:Calc. from Peak Group 1 - Event 1 Scan

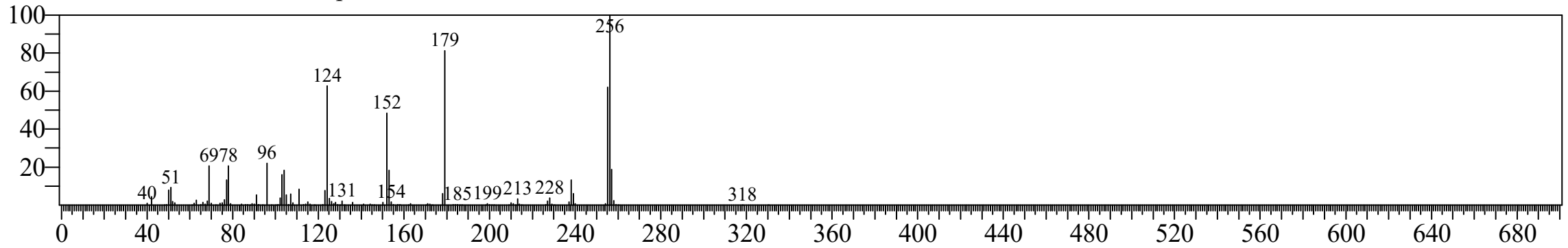

Hit#:1 Entry:25088 Library:NIST14s.lib
SI:93 Formula:C15H12O4 CAS:480-39-7 MolWeight:256 RetIndex:2403
CompName:4H-1-Benzopyran-4-one, 2,3-dihydro-5,7-dihydroxy-2-phenyl-, (S)- $$ Dihydrochrysin $$ Galangin flavanone $$ Pinocembrin $$ 4H-1-Benzop

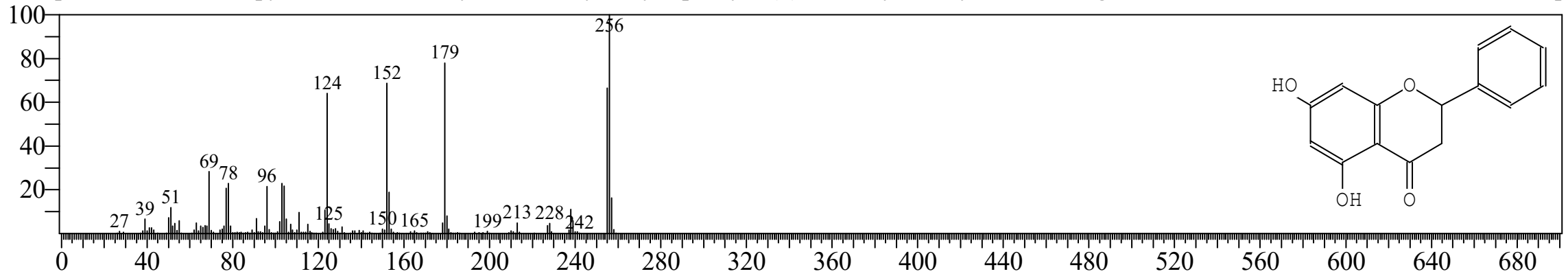

Hit#:2 Entry:92205 Library:NIST14.lib
SI:90 Formula:C15H12O4 CAS:480-39-7 MolWeight:256 RetIndex:2403
CompName:4H-1-Benzopyran-4-one, 2,3-dihydro-5,7-dihydroxy-2-phenyl-, (S)- $$ Dihydrochrysin $$ Galangin flavanone $$ Pinocembrin $$ 4H-1-Benzop

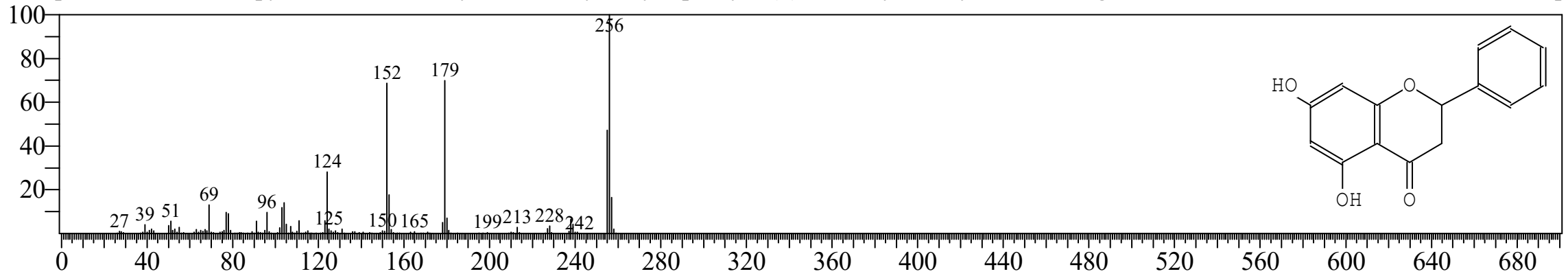

Hit#:3 Entry:182626 Library:WILEY8.LIB
SI:79 Formula:C15H12O4 CAS:480-39-7 MolWeight:256 RetIndex:0
CompName:4H-1-BENZOPYRAN-4-ONE, 2,3-DIHYDRO-5,7-DIHYDROXY-2-PHENYL-, (S)- $$ 5,7-DIHYDROXY-2-PHENYL-2,3-DIHYDRO-4H-C

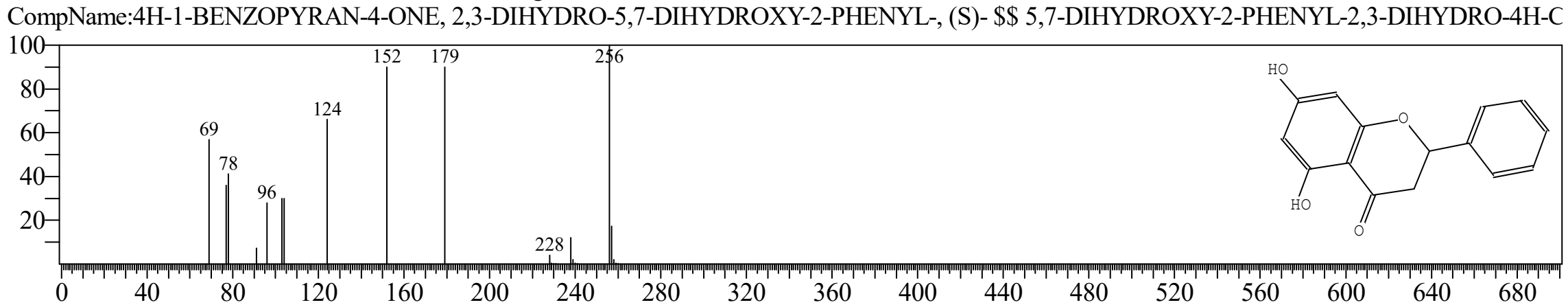

<< Target >>
Line#:162 R.Time:23.605(Scan#:4022) MassPeaks:140
RawMode:Averaged 23.600-23.610(4021-4023) BasePeak:284.15(939600)
BG Mode:Calc. from Peak Group 1 - Event 1 Scan

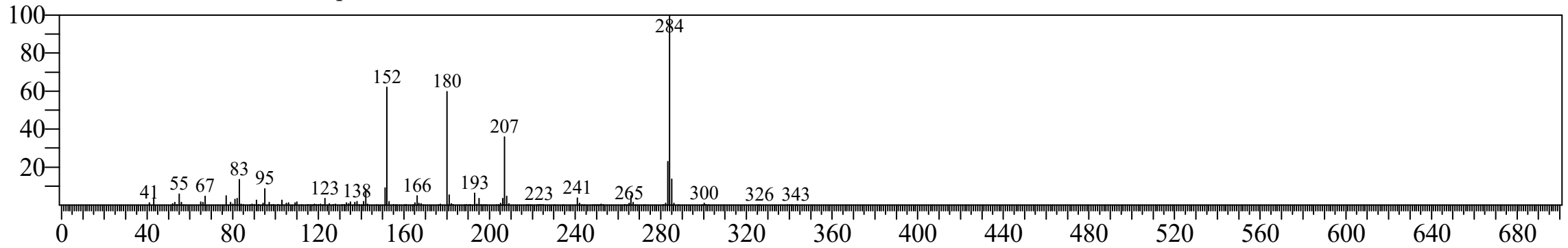

Hit#:1 Entry:219869 Library:WILEY8.LIB
SI:73 Formula:C17H16O4 CAS:56297-79-1 MolWeight:284 RetIndex:0
CompName:5,7-DIHYDROXY-6,8-DIMETHYL-2-PHENYL-2,3-DIHYDRO-4H-CHROMEN-4-ONE # $$ 4H-1-BENZOPYRAN-4-ONE, 2,3-DIHYDRO

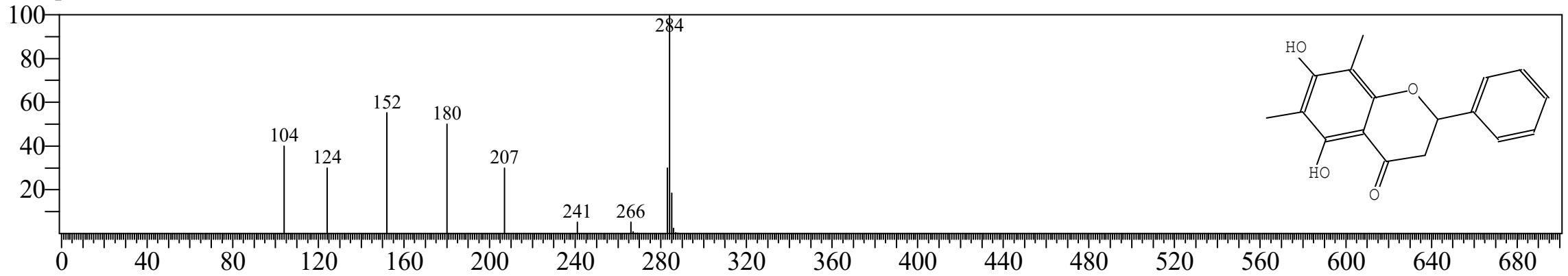

Hit#:2 Entry:116899 Library:NIST14.lib
SI:71 Formula:C17H16O4 CAS:56297-79-1 MolWeight:284 RetIndex:2629
CompName:4H-1-Benzopyran-4-one, 2,3-dihydro-5,7-dihydroxy-6,8-dimethyl-2-phenyl-, (S)- $$ 5,7-Dihydroxy-6,8-dimethyl-2-phenyl-2,3-dihydro-4H-chro

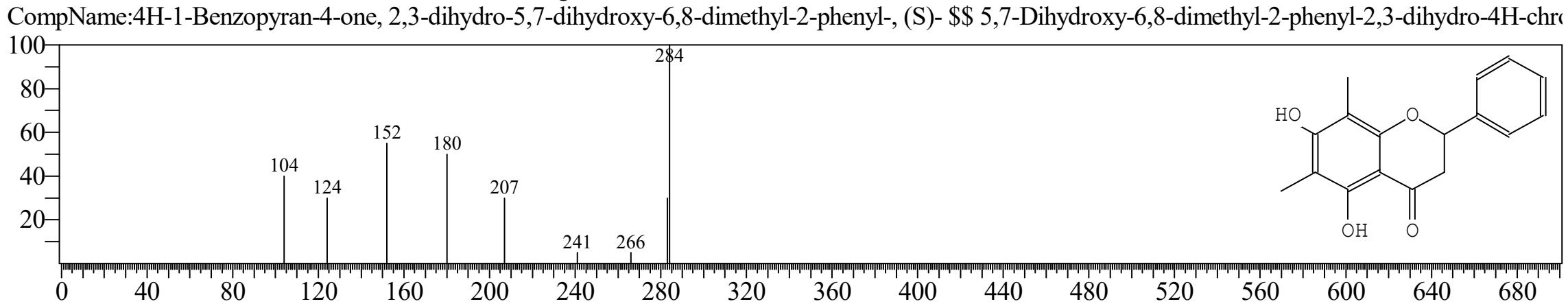

Hit#:3 Entry:116883 Library:NIST14.lib
SI:70 Formula:C17H16O4 CAS:1036-72-2 MolWeight:284 RetIndex:2340
CompName:4H-1-Benzopyran-4-one, 2,3-dihydro-5,7-dimethoxy-2-phenyl- $$ Flavanone, 5,7-dimethoxy- $$ 5,7-Dimethoxyflavanone $$ 5,7-Dimethoxy-2-

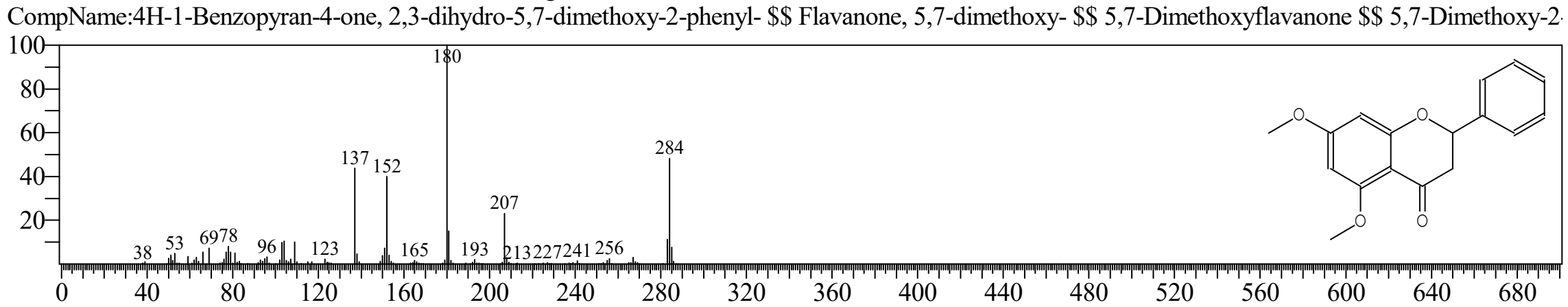

<< Target >>
Line#:163   R.Time:23.815(Scan#:4064) MassPeaks:153
RawMode:Averaged 23.810-23.820(4063-4065) BasePeak:270.10(2517153)
BG Mode:Calc. from Peak Group 1 - Event 1 Scan

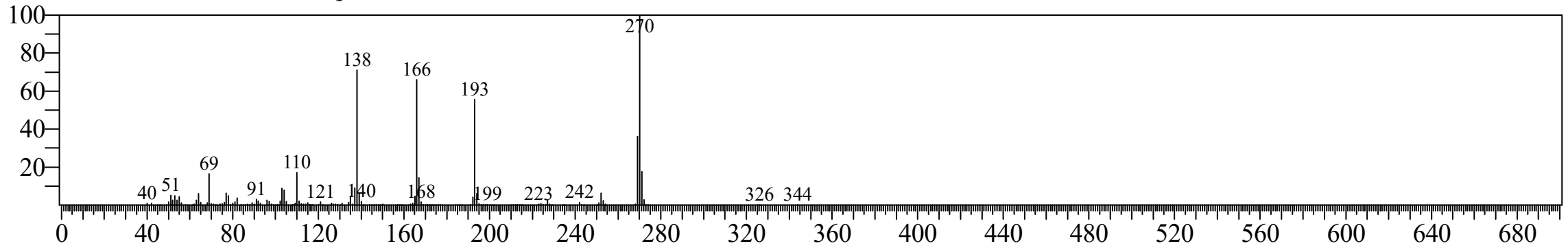

Hit#:1 Entry:104427 Library:NIST14.lib
SI:83 Formula:C16H14O4 CAS:480-37-5 MolWeight:270 RetIndex:0
CompName:4H-1-Benzopyran-4-one, 2,3-dihydro-5-hydroxy-7-methoxy-2-phenyl-, (S)- $$ 5-Hydroxy-7-methoxyflavanone $$ Pinostrobin $$ Flavanone, 5-

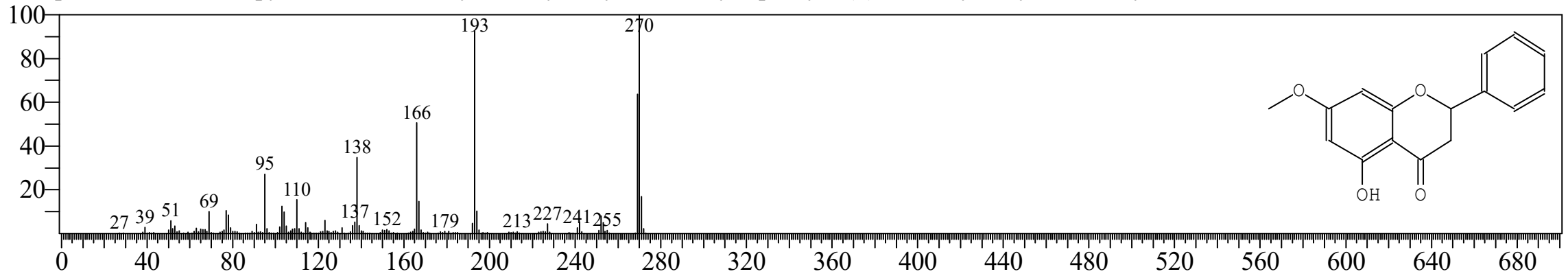

Hit#:2 Entry:104428 Library:NIST14.lib
SI:81 Formula:C16H14O4 CAS:18956-15-5 MolWeight:270 RetIndex:2440
CompName:2-Propen-1-one, 1-(2,6-dihydroxy-4-methoxyphenyl)-3-phenyl-, (E)- $$ Chalcone, 2',6'-dihydroxy-4'-methoxy- $$ Pinostrobin chalcone $$ 2-Pr

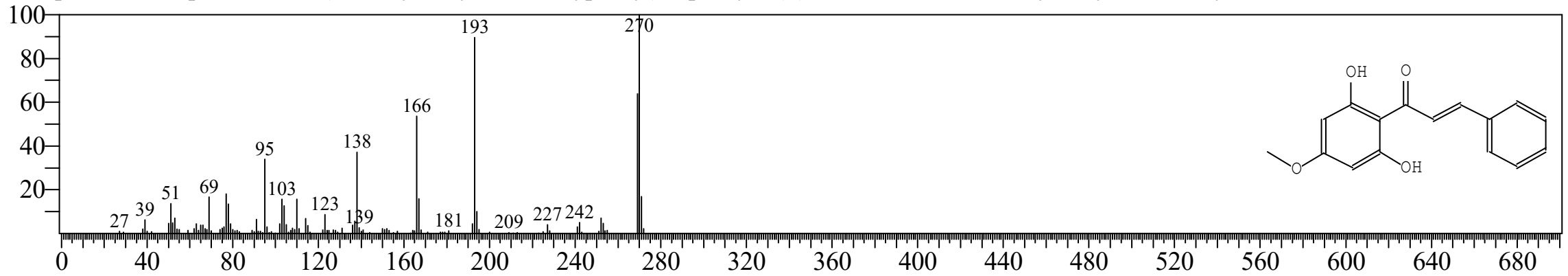

Hit#:3 Entry:26232 Library:NIST14s.lib
SI:80 Formula:C16H14O4 CAS:18956-15-5 MolWeight:270 RetIndex:2440
CompName:2-Propen-1-one, 1-(2,6-dihydroxy-4-methoxyphenyl)-3-phenyl-, (E)- $$ Chalcone, 2',6'-dihydroxy-4'-methoxy- $$ Pinostrobin chalcone $$ 2-Pr

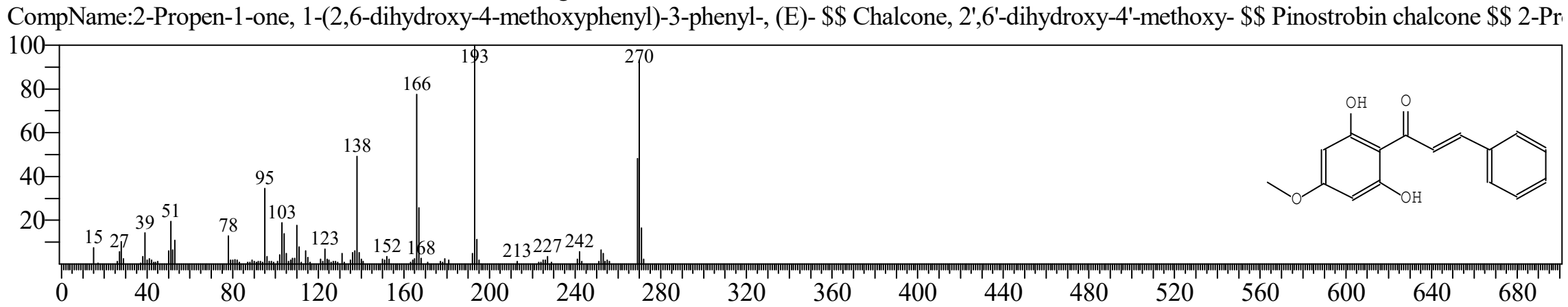

<< Target >>
Line#:164   R.Time:23.935(Scan#:4088) MassPeaks:172
RawMode:Averaged 23.930-23.940(4087-4089) BasePeak:284.15(2151562)
BG Mode:Calc. from Peak Group 1 - Event 1 Scan

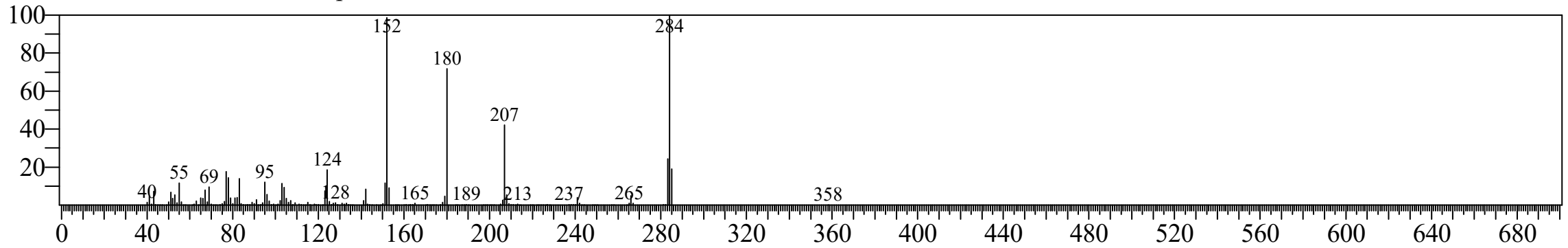

Hit#:1 Entry:219869 Library:WILEY8.LIB
SI:70 Formula:C17H16O4 CAS:56297-79-1 MolWeight:284 RetIndex:0
CompName:5,7-DIHYDROXY-6,8-DIMETHYL-2-PHENYL-2,3-DIHYDRO-4H-CHROMEN-4-ONE # $$ 4H-1-BENZOPYRAN-4-ONE, 2,3-DIHYDRO

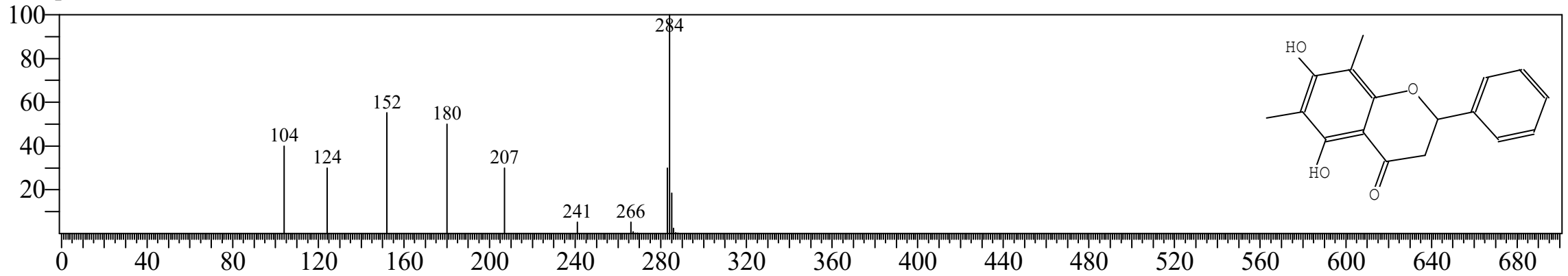

Hit#:2 Entry:116883 Library:NIST14.lib
SI:70 Formula:C17H16O4 CAS:1036-72-2 MolWeight:284 RetIndex:2340
CompName:4H-1-Benzopyran-4-one, 2,3-dihydro-5,7-dimethoxy-2-phenyl- $$ Flavanone, 5,7-dimethoxy- $$ 5,7-Dimethoxyflavanone $$ 5,7-Dimethoxy-2-

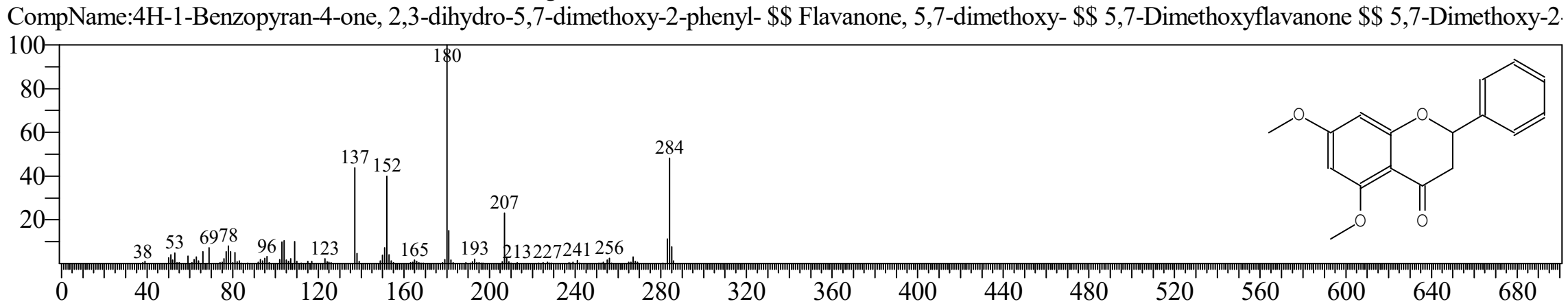

Hit#:3 Entry:116899 Library:NIST14.lib
SI:68 Formula:C17H16O4 CAS:56297-79-1 MolWeight:284 RetIndex:2629
CompName:4H-1-Benzopyran-4-one, 2,3-dihydro-5,7-dihydroxy-6,8-dimethyl-2-phenyl-, (S)- $$ 5,7-Dihydroxy-6,8-dimethyl-2-phenyl-2,3-dihydro-4H-chro

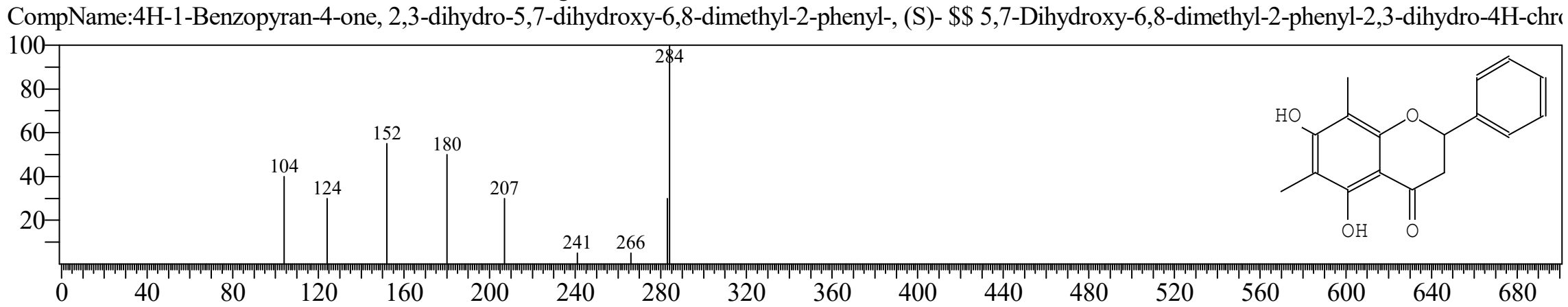

<< Target >>
Line#:165 R.Time:24.355(Scan#:4172) MassPeaks:256
RawMode:Averaged 24.350-24.360(4171-4173) BasePeak:194.10(2208816)
BG Mode:Calc. from Peak Group 1 - Event 1 Scan

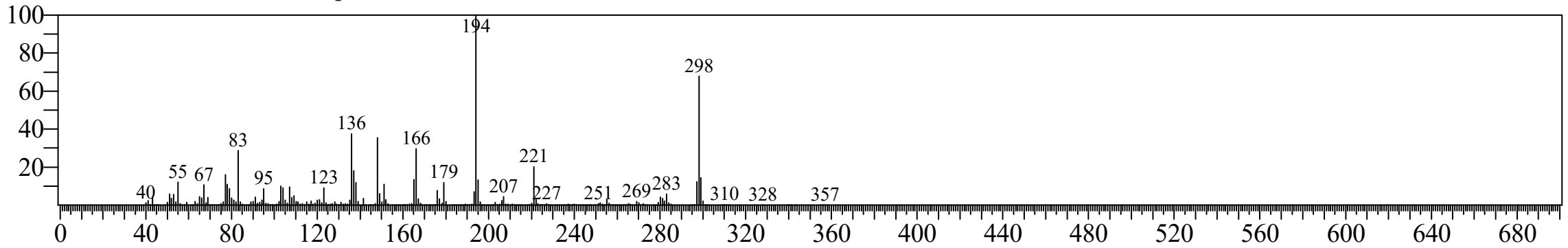

Hit#:1 Entry:237401 Library:WILEY8.LIB
SI:66 Formula:C17H18N2OS CAS:0-00-0 MolWeight:298 RetIndex:0
CompName:1H-BENZOIMIDAZOLE, 5-ETHOXY-2-PHENETHYLSULFANYL- $$ 5-ETHOXY-2-[(2-PHENYLETHYL)SULFANYL]-1H-BENZIMIDA

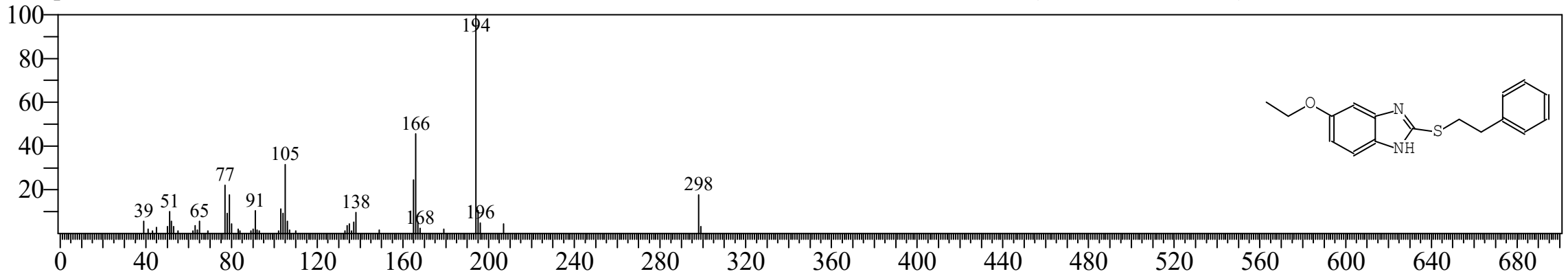

Hit#:2 Entry:129231 Library:NIST14.lib
SI:66 Formula:C17H18N2OS CAS:0-00-0 MolWeight:298 RetIndex:2673
CompName:1H-Benzoimidazole, 5-ethoxy-2-phenethylsulfanyl-

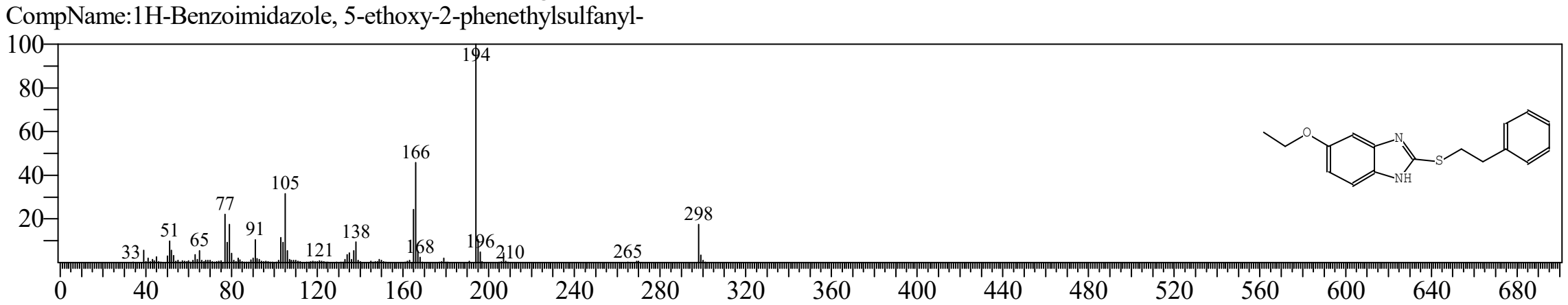

Hit#:3 Entry:95081 Library:WILEY8.LIB
SI:60 Formula:C11H14O3 CAS:50505-61-8 MolWeight:194 RetIndex:0
CompName:2,5-DIMETHOXY-4-ETHYLBENZALDEHYDE

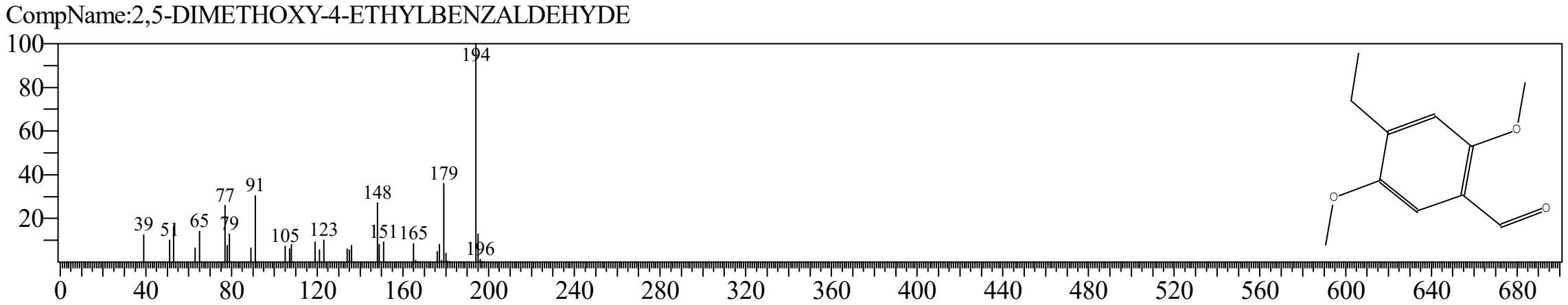

<< Target >>
Line#:166   R.Time:24.650(Scan#:4231) MassPeaks:153
RawMode:Averaged 24.645-24.655(4230-4232) BasePeak:181.05(5120980)
BG Mode:Calc. from Peak Group 1 - Event 1 Scan

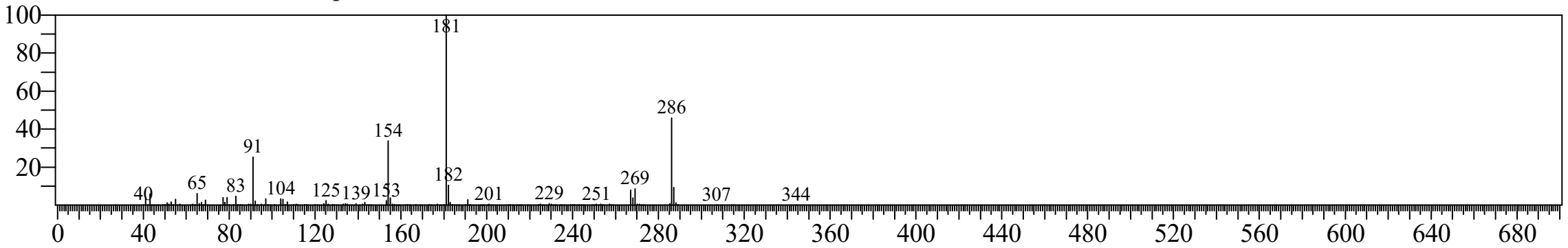

Hit#:1 Entry:221625 Library:WILEY8.LIB
SI:65 Formula:C12H11FO3S2 CAS:121031-80-9 MolWeight:286 RetIndex:0
CompName:SPIRO[4H-1-BENZOPYRAN-4,2'-[1,3]DITHIAN]-5'-ONE, 6-FLUORO-2,3-DIHYDRO-, 1'-OXIDE $$ 6-FLUORO-2,3-DIHYDROSPIRO{2

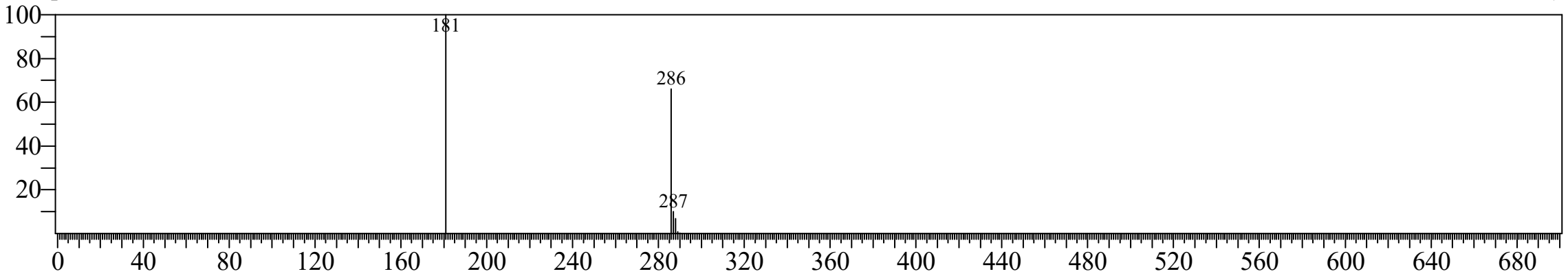

Hit#:2 Entry:65688 Library:NIST14.lib
SI:64 Formula:C12H16O4 CAS:519-40-4 MolWeight:224 RetIndex:1971
CompName:Aspidinol $$ 1-(2,6-Dihydroxy-4-methoxy-3-methylphenyl)-1-butanone $$

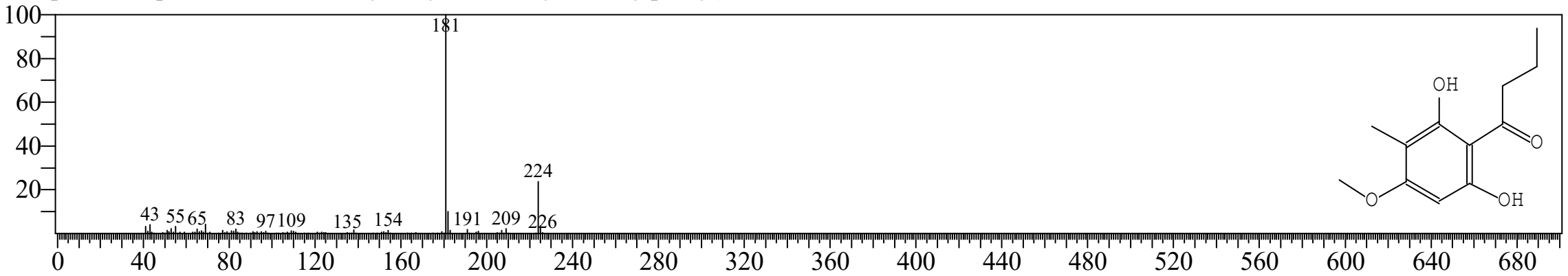

Hit#:3 Entry:34696 Library:NIST14.lib
SI:63 Formula:C12H10N2 CAS:0-00-0 MolWeight:182 RetIndex:1521
CompName:1,1-Di(pyrid-4-yl)ethylene

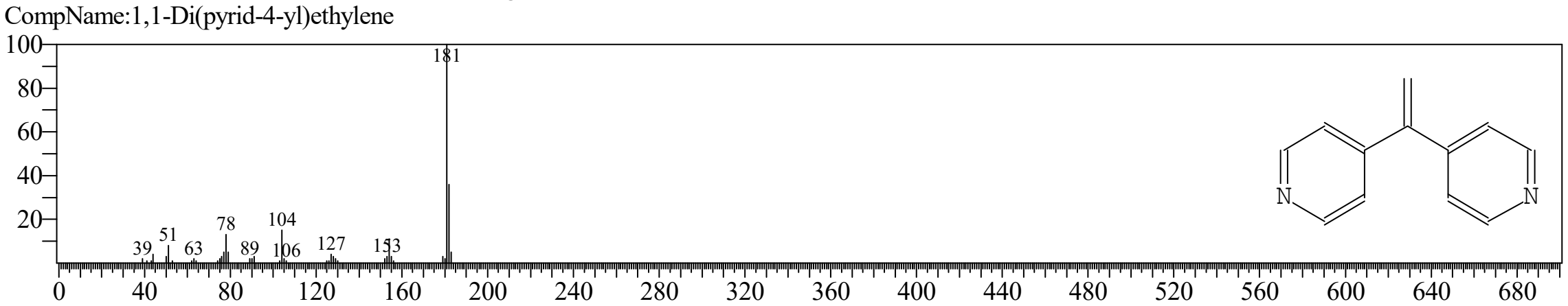

<< Target >>
Line#:167   R.Time:24.870(Scan#:4275) MassPeaks:239
RawMode:Averaged 24.865-24.875(4274-4276) BasePeak:298.15(1626994)
BG Mode:Calc. from Peak Group 1 - Event 1 Scan

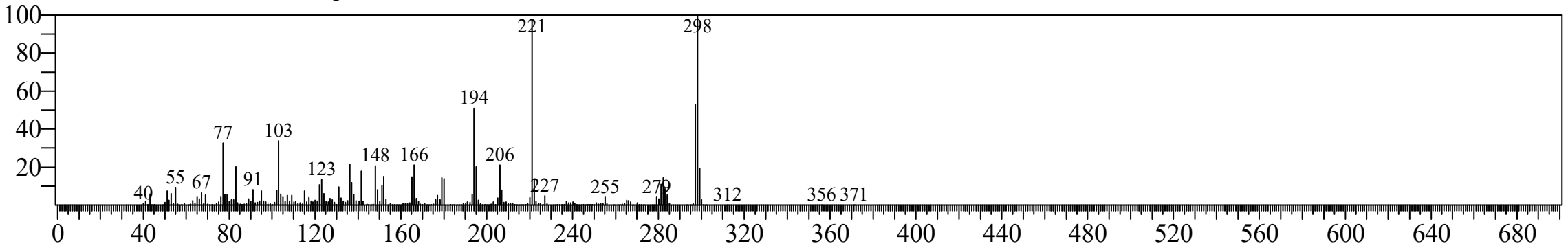

Hit#:1 Entry:129513 Library:NIST14.lib
SI:59 Formula:C18H18O4 CAS:0-00-0 MolWeight:298 RetIndex:2443
CompName:2,3-Dihydro-5-hydroxy-6,8,8-trimethyl-2-phenyl-4H-1-benzopyran-4,7(8H)-dione

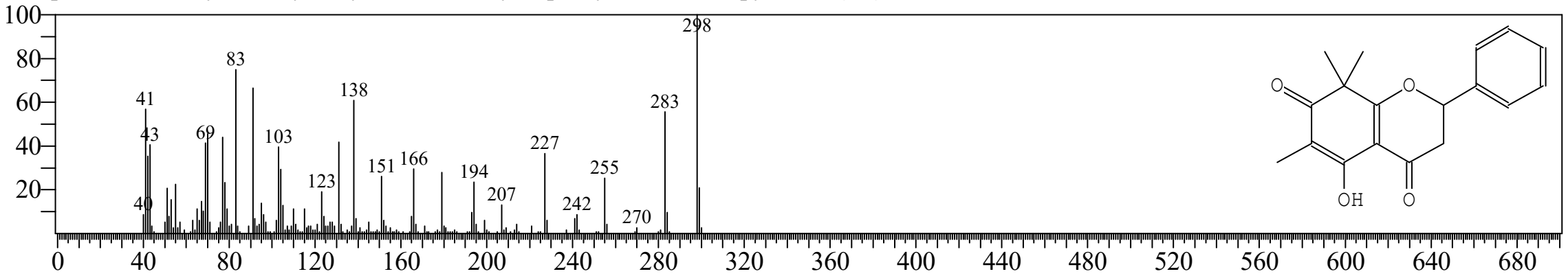

Hit#:2 Entry:28287 Library:NIST14s.lib
SI:57 Formula:C21H18N2 CAS:742-01-8 MolWeight:298 RetIndex:2604
CompName:1H-Pyrazole, 4,5-dihydro-1,3,5-triphenyl- $$ 1,3,5-Triphenyl-4,5-dihydro-1H-pyrazole # $$

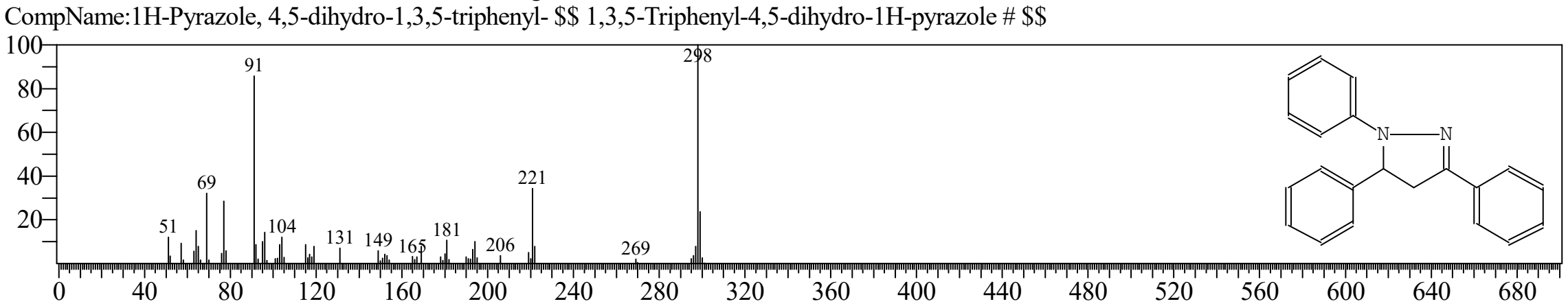

Hit#:3 Entry:129800 Library:NIST14.lib
SI:57 Formula:C21H18N2 CAS:742-01-8 MolWeight:298 RetIndex:2604
CompName:1H-Pyrazole, 4,5-dihydro-1,3,5-triphenyl- $$ 1,3,5-Triphenyl-4,5-dihydro-1H-pyrazole # $$

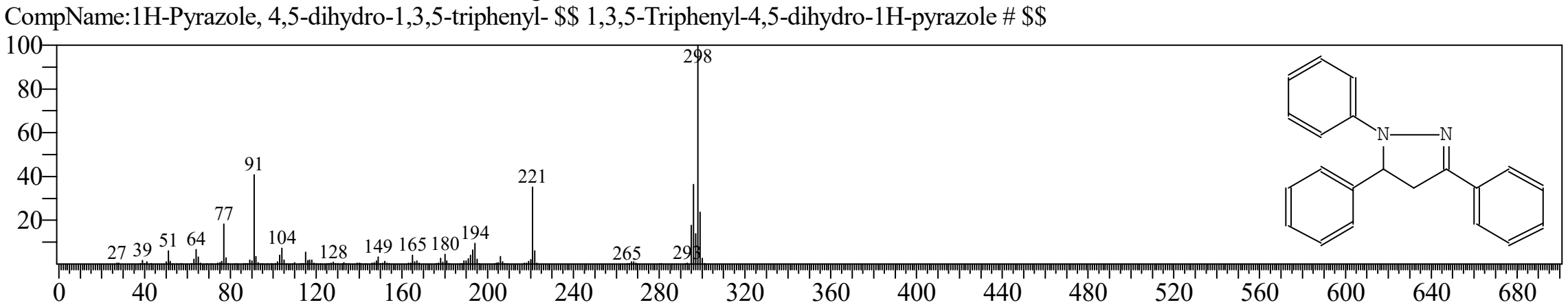

<< Target >>
Line#:168 R.Time:25.270(Scan#:4355) MassPeaks:288
RawMode:Averaged 25.265-25.275(4354-4356) BasePeak:207.05(2449234)
BG Mode:Calc. from Peak Group 1 - Event 1 Scan

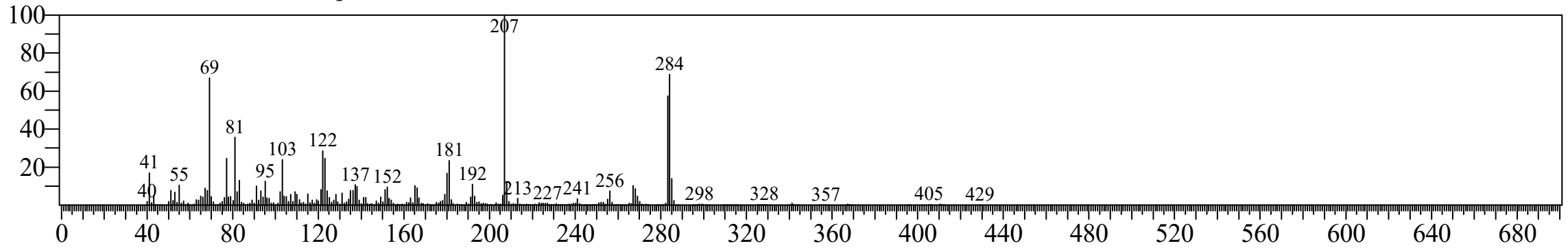

Hit#:1 Entry:116888 Library:NIST14.lib
SI:75 Formula:C17H16O4 CAS:1775-97-9 MolWeight:284 RetIndex:0
CompName:(E)-1-(2-Hydroxy-4,6-dimethoxyphenyl)-3-phenylprop-2-en-1-one $$ 2-Propen-1-one, 1-(2-hydroxy-4,6-dimethoxyphenyl)-3-phenyl-, (2E)- $$

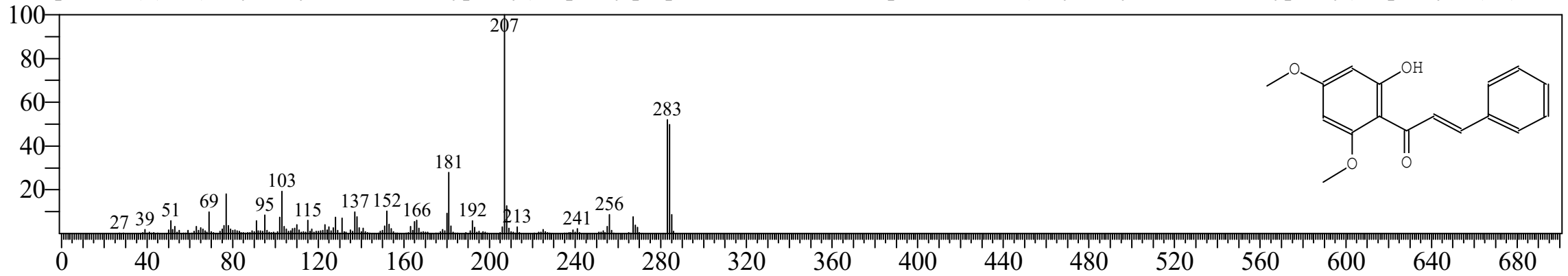

Hit#:2 Entry:116883 Library:NIST14.lib
SI:59 Formula:C17H16O4 CAS:1036-72-2 MolWeight:284 RetIndex:2340
CompName:4H-1-Benzopyran-4-one, 2,3-dihydro-5,7-dimethoxy-2-phenyl- $$ Flavanone, 5,7-dimethoxy- $$ 5,7-Dimethoxyflavanone $$ 5,7-Dimethoxy-2-

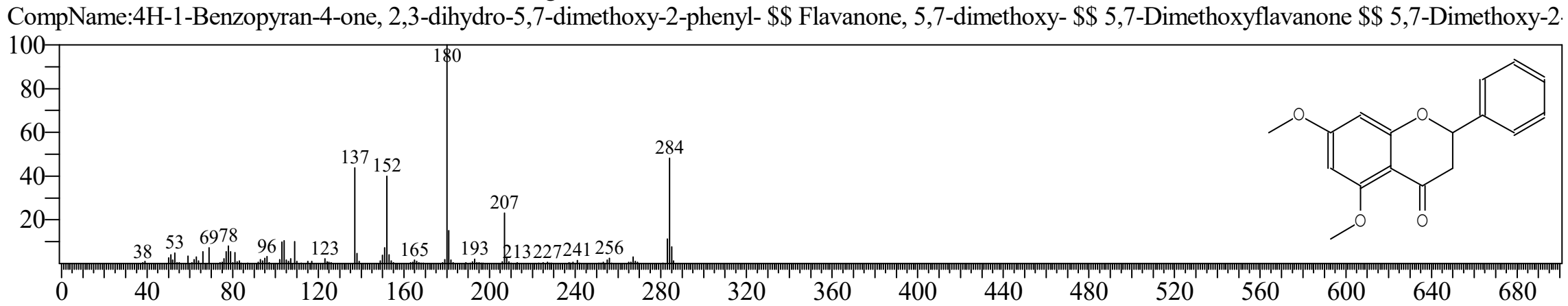

Hit#:3 Entry:281313 Library:WILEY8.LIB
SI:58 Formula:C21H36O3 CAS:0-00-0 MolWeight:336 RetIndex:0
CompName:2-HEPTYL-3-(7-CARBOMETHOXYHEPTYL)-CYCLOPENT-2-ENONE

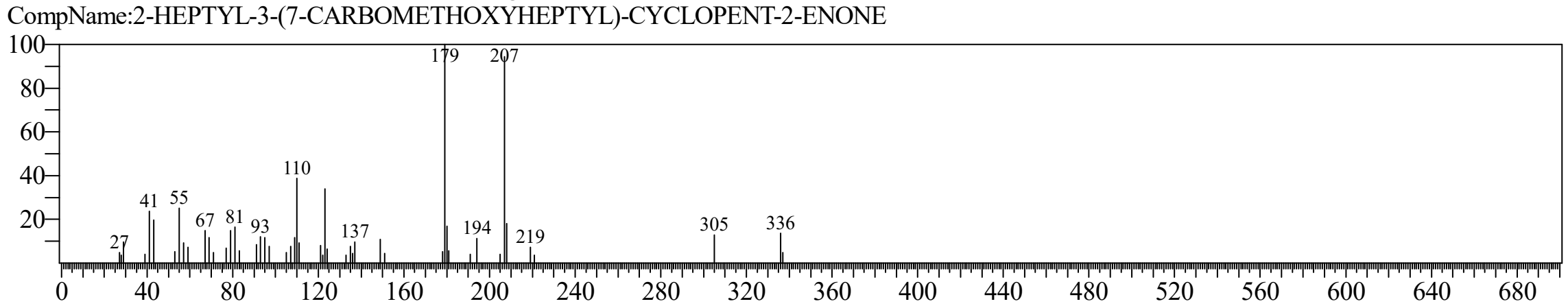

<< Target >>
Line#:169   R.Time:25.625(Scan#:4426) MassPeaks:212
RawMode:Averaged 25.620-25.630(4425-4427) BasePeak:419.40(37097)
BG Mode:Calc. from Peak Group 1 - Event 1 Scan

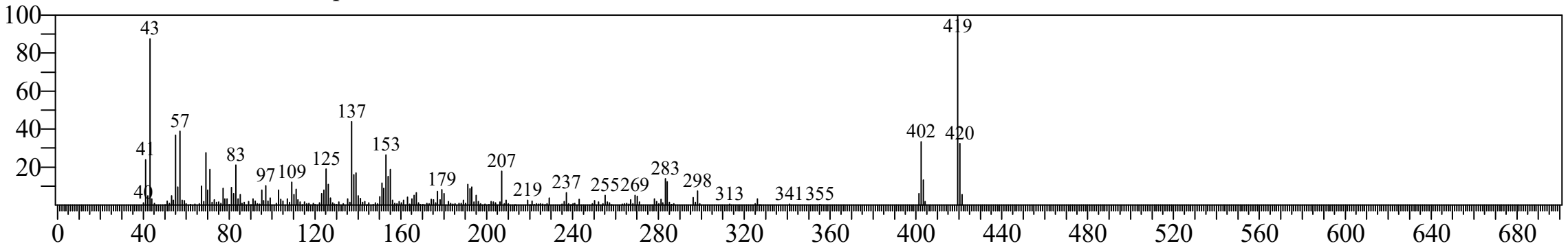

Hit#:1 Entry:226606 Library:NIST14.lib
SI:82 Formula:C29H50O4 CAS:601490-41-9 MolWeight:462 RetIndex:0
CompName:.alpha.-Tocospiro B $$ (2R,5R,9S)-9-Acetyl-9-hydroxy-2,7,8-trimethyl-2-((4R,8R)-4,8,12-trimethyltridecyl)-1-oxaspiro[4.4]non-7-en-6-one $$

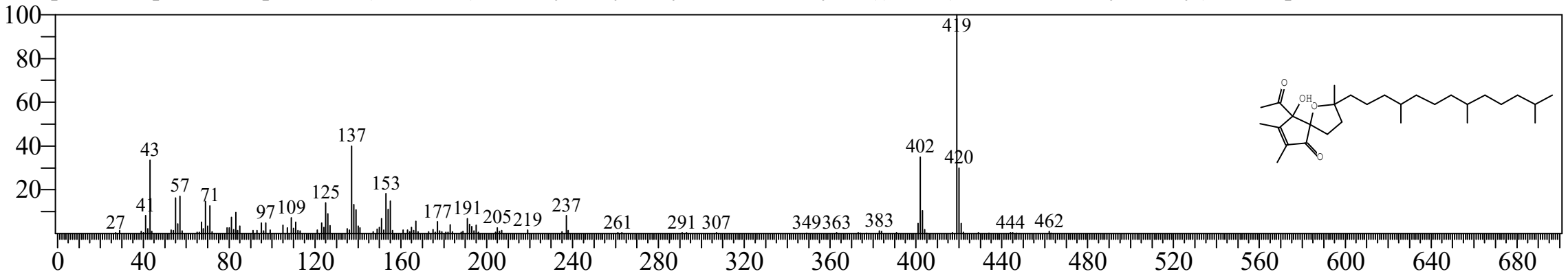

Hit#:2 Entry:226605 Library:NIST14.lib
SI:81 Formula:C29H50O4 CAS:601490-40-8 MolWeight:462 RetIndex:0
CompName:.alpha.-Tocospiro A $$ 1-Oxaspiro[4.4]non-7-en-6-one, 9-acetyl-9-hydroxy-2,7,8-trimethyl-2-[(4R,8R)-4,8,12-trimethyltridecyl]-, (2S,5R,9S)- $

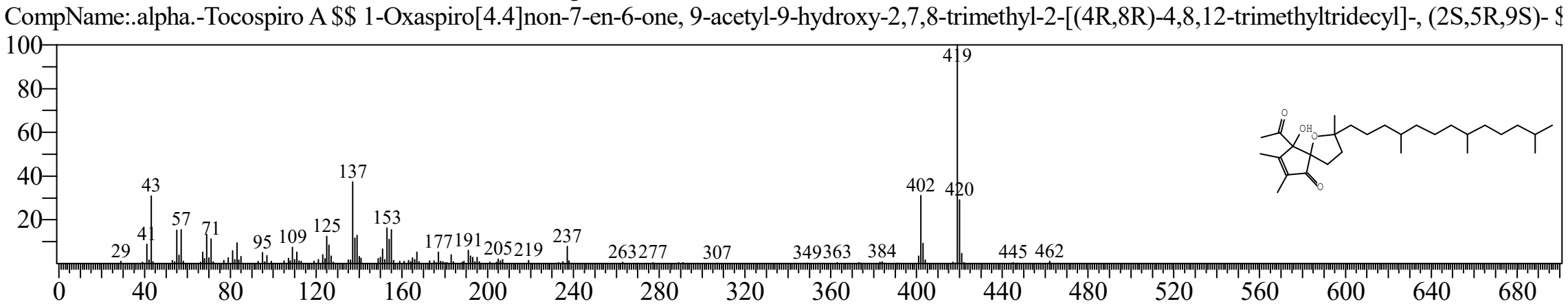

Hit#:3 Entry:46628 Library:WILEY8.LIB
SI:61 Formula:C10H18O CAS:54244-89-2 MolWeight:154 RetIndex:0
CompName:FURAN, 4,5-DIETHYL-2,3-DIHYDRO-2,3-DIMETHYL- $$ 4,5-DIETHYL-2,3-DIMETHYL-2,3-DIHYDROFURAN # $$ 2,3-DIMETHYL-

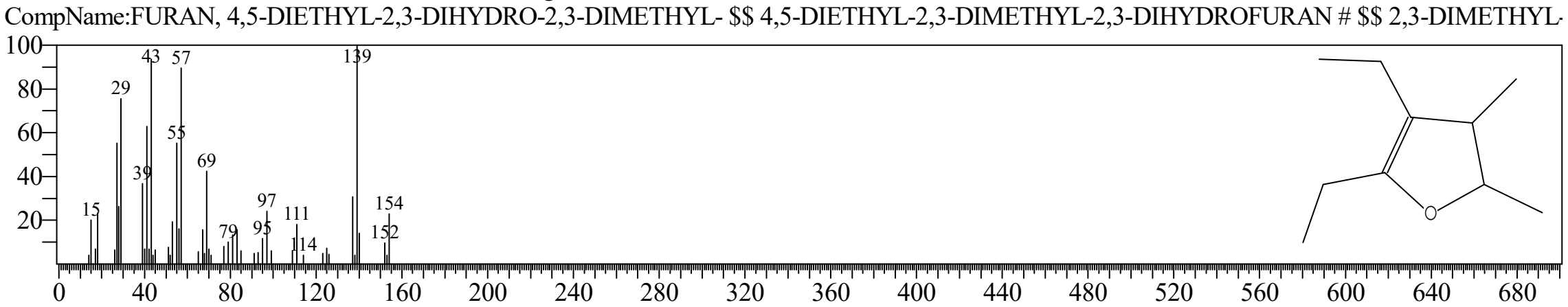

<< Target >>
Line#:170   R.Time:25.815(Scan#:4464) MassPeaks:260
RawMode:Averaged 25.810-25.820(4463-4465) BasePeak:57.10(310545)
BG Mode:Calc. from Peak Group 1 - Event 1 Scan

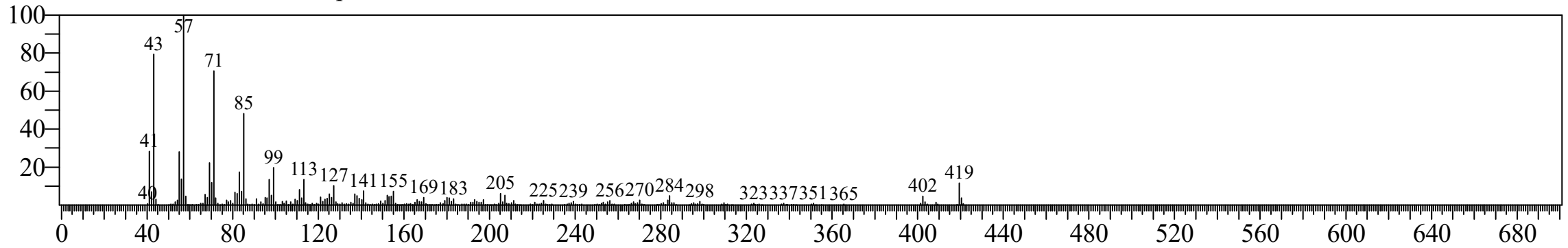

Hit#:1 Entry:340092 Library:WILEY8.LIB
SI:89 Formula:C29H60 CAS:630-03-5 MolWeight:408 RetIndex:0
CompName:CELIDONIOL, DEOXY- $$ NONACOSANE $$ AI3-36284 $$ EINECS 211-126-2 $$ N-NONACOSANE

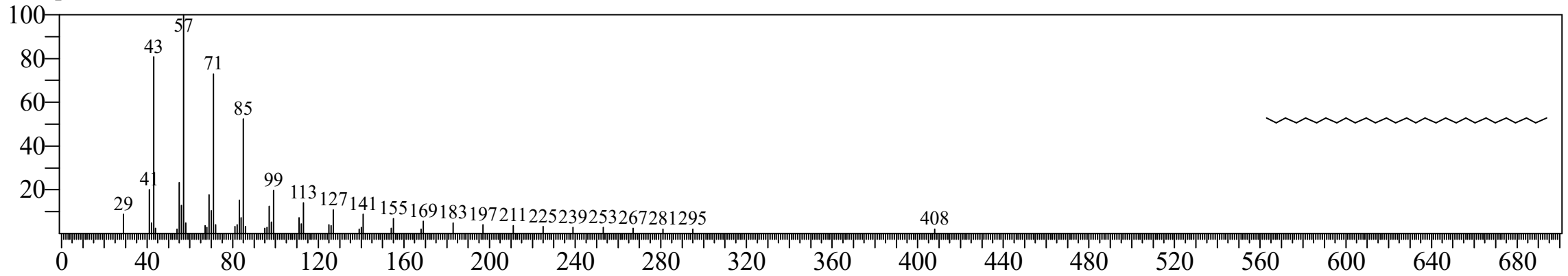

Hit#:2 Entry:32561 Library:NIST14s.lib
SI:89 Formula:C29H60 CAS:630-03-5 MolWeight:408 RetIndex:2904
CompName:Nonacosane $$ n-Nonacosane $$

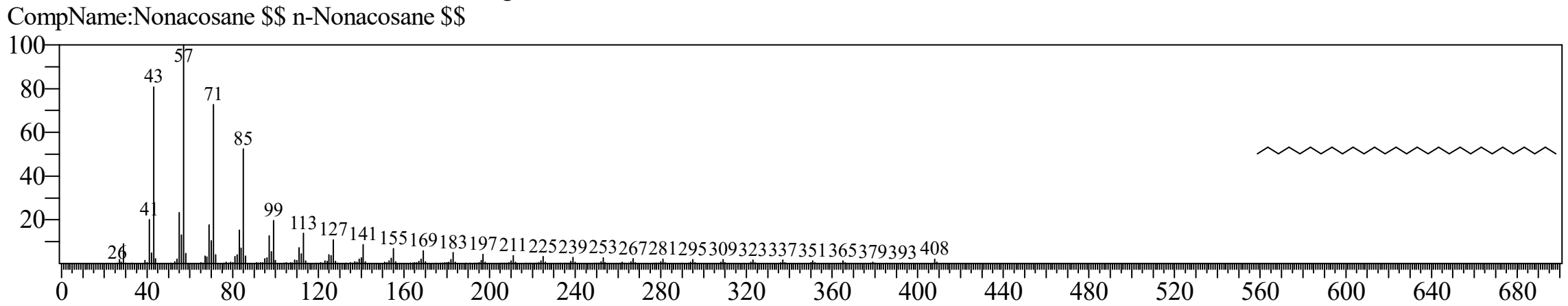

Hit#:3 Entry:321023 Library:WILEY8.LIB
SI:89 Formula:C27H56 CAS:593-49-7 MolWeight:380 RetIndex:0
CompName:HEPTACOSANE $$ AI3-36283 $$ EINECS 209-792-4 $$ N-HEPTACOSANE

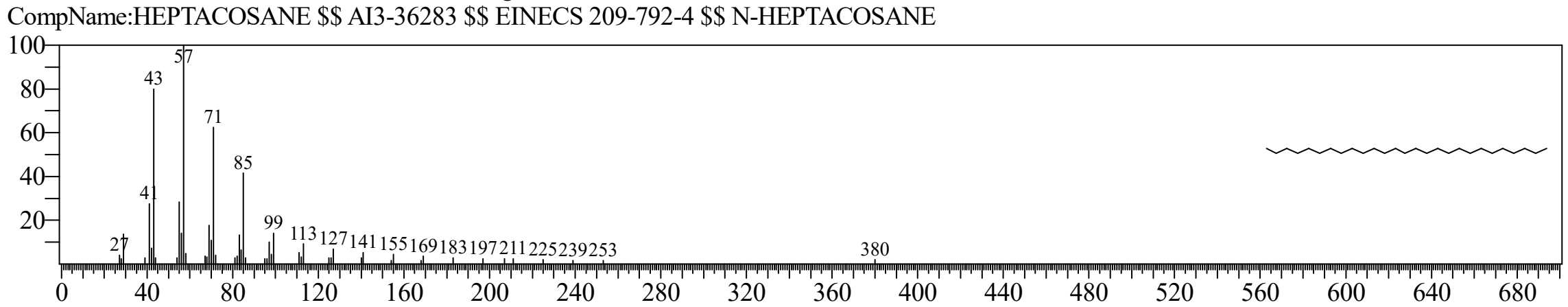

Supplement: Supplementary file 1 [file DataSheet1.pdf]
