# Supplementary material for: Integrative network pharmacology and molecular modeling approaches reveal the therapeutic potential of Syzygium samarangense phytocompounds against diabetes retinopathy
Source: Front Bioinform. 2026 Aug 18;6:1884743. doi: 10.3389/fbinf.2026.1884743 (PMC13529976; doi:10.3389/fbinf.2026.1884743)
Supplement: Supplementary file 2 [file Supplementaryfile1.docx]

**Figure S1:** Temporal evolution of hydrogen bond and hydrophobic contact interactions for SS4–ALB and SS5–SRC complexes during 100 ns MD simulation.
